# Different Occupations Associated with Amyotrophic Lateral Sclerosis: Is Diesel Exhaust the Link?

**DOI:** 10.1371/journal.pone.0080993

**Published:** 2013-11-11

**Authors:** Roger Pamphlett, Anna Rikard-Bell

**Affiliations:** The Stacey Motor Neuron Disease Laboratory, Department of Pathology, Sydney Medical School, the University of Sydney, Sydney, New South Wales, Australia; University of Oxford, United Kingdom

## Abstract

The cause of sporadic amyotrophic lateral sclerosis (SALS) remains unknown. We attempted to find out if occupational exposure to toxicants plays a part in the pathogenesis of this disease. In an Australia-wide case-control study we compared the lifetime occupations of 611 SALS and 775 control individuals. Occupations were coded using country-specific as well as international classifications. The risk of SALS for each occupation was calculated with odds ratios using logistic regression. In addition, the literature was searched for possible toxicant links between our findings and previously-reported occupational associations with SALS. Male occupations in our study that required lower skills and tasks tended to have increased risks of SALS, and conversely, those occupations that required higher skills and tasks had decreased risks of SALS. Of all the occupations, only truck drivers, where exposure to diesel exhaust is common, maintained an increased risk of SALS throughout all occupational groups. Another large case-control study has also found truck drivers to be at risk of SALS, and almost two-thirds of occupations, as well as military duties, that have previously been associated with SALS have potential exposure to diesel exhaust. In conclusion, two of the largest case-control studies of SALS have now found that truck drivers have an increased risk of SALS. Since exposure to diesel exhaust is common in truck drivers, as well as in other occupations that have been linked to SALS, exposure to this toxicant may underlie some of the occupations that are associated with SALS.

## Introduction

In the great majority of patients with sporadic amyotrophic lateral sclerosis (SALS) the cause of the disease remains unknown, and the proportion of environmental or genetic factors that underlie SALS continues to be debated [1]. Recent findings that the sporadic form of ALS may be even more common than was previously thought [2] have again focussed interest on the possibility that environmental agents play a part in the pathogenesis of the disease. Occupation has been widely used as a surrogate for environmental exposure to toxicants (for a review of occupational studies in ALS, see Sutedja et al. (2009) [3]), since few individuals are likely to be aware of particular toxicants they have been exposed to during their working lives. 

A feature of some previous studies of occupations in SALS has been that only the main (typically the longest-held) occupation of the individual has been considered [4-6], though some have used all lifetime occupations [7-10]. It could be argued that all such studies should take lifetime occupations into account, since even time spent in a relatively short-lasting work environment that exposes an individual to a toxicant could be relevant to the later appearance of a disease. For example, a short exposure to asbestos fibres can lead to mesothelioma many years after asbestos exposure. Using all occupations may be especially applicable to SALS, since a short exposure to a neurotoxicant such as inorganic mercury results in the toxicant remaining within motor neurons indefinitely in both animals [11] and humans [12]. 

Occupations can be coded for epidemiological studies using either country-specific codes (such as the Australian and New Zealand Classification of Occupations, ANZSCO) or the International Standard Classification of Occupations (ISCO). ISCO is useful since it allows comparisons of studies performed in different countries, but this classification considers occupations in groups only and does not extend to individual occupations, as does a classification such as ANZSCO. 

Although a large number of occupations have been reported to be associated with SALS [6], no toxicant exposure that is present in the majority of these occupations, and that has the potential to cause SALS, has been proposed. Our study, looking at lifetime occupations in people with SALS and controls, has found that truck drivers in particular have an increased risk of SALS. We suggest that a plausible link between a number of occupational findings in SALS is exposure to diesel exhaust.

## Methods

### Ethics statement

Individuals gave written informed consent before providing the questionnaire data. The research protocol (X11-0383) was approved by the Human Research Ethics Committee of the Sydney South West Area Health Service.

### Study population

Notices were placed in the newsletters of all Australian state-based ALS Associations asking for self-reported questionnaires from patients with ALS, as well as from partners, friends of people with ALS, and Rotary Club members. Recruitment was from April 2000 to June 2011. Inclusion criteria were patients with sporadic ALS (SALS, cases) and people unrelated to SALS patients who did not have ALS (controls); all were white Australian residents who were above the age of 24 y at the time of survey. For patients, the questionnaire could be completed at any time after the diagnosis of SALS had been made.

### Cases and controls

Cases were chosen from patients with SALS who had completed the questionnaire where clinical notes and the results of electrophysiological and imaging investigations were available from treating neurologists. Patients were classified as having ALS if they fulfilled the probable or definite revised El Escorial criteria for ALS [13]. The outcome was defined as a diagnosis of SALS or a normal control.

### Classification of occupations

Individuals were asked to state the title of each occupation they had ever had, from the age of 18 years on, that lasted for 6 months or more. Occupations were coded into groups, according to similarities in skills and tasks performed, firstly using the 2009 revised 2006 edition of ANZSCO, a five-number code which has 5 groups: Major (8 titles), Sub-Major (43 titles), Minor (97 titles), Unit (358 titles) and Occupation (998 titles) (see Classification S1). Secondly, occupations were classified using the 2008 ISCO, a four-number code which has 4 groups: Major (10 titles), Sub-Major (28 titles), Minor (146 titles) and Unit (390 titles). The ANSCO classification is roughly equivalent to the ISCO, but with an additional detailed Group 5 that codes for specific occupational titles. See Figure S1, for a comparison of group numbers in ANZSCO and ISCO, as well as Figure S2, for an example of how an occupation (in this instance, a sonographer) is coded in ANZSCO and ISCO.

Neither classification has a code for ‘domestic duties’ or similar responses. Unique codes were therefore added in ANZSCO (code 811312) into the Unit Group: Domestic Cleaners, and in ISCO (code 9113) into the Minor Group: Domestic, Hotel and Office Cleaners and Helpers, since domestic duties often include cleaning. We acknowledge, of course, that domestic duties entail much wider roles, and that this is a compromise code. Responses such as ‘public servant’ that apply to no specific occupation were coded as unclassifiable and were not included in the statistical analyses. If an individual entered no classifiable occupation they were removed from the analysis.

Because men and women in this cohort had different spectrums of occupations, all analyses were undertaken on male and female groups separately. An attempt was made to gauge the diligence of individuals in completing the questionnaire by comparing the average number of occupations that were listed by cases and controls.

### Statistical methods

#### Initial data handling

Data were entered into a Filemaker Pro 12 database (FileMaker Inc., Santa Clara CA) and then transferred to an Excel file, where unclassifiable occupations were removed. 

#### Removal of repeat occupations

In the questionnaire, occupations were listed in the chronological order in which they were undertaken, so individuals could repeat an occupation later on in their working life. To remove the possibility of obtaining duplicate responses, RStudio v0.96.316 was used to construct comma-separated values (.csv) files with True (never employed in that group) or False (ever been employed in that group) values, for each male/female, ANZSCO/ISCO, and occupational group combination, resulting in 10 files for ANZSCO and 8 for ISCO. An example of the R script for the Male ISCO Minor Group is given in Methods S1. 

#### Testing potential confounders

Each .csv file was imported into SPSS v20 (SPSS Inc., Chicago IL) and unconditional logistic regression was undertaken for each occupational title to test for potential SALS confounders of age [1] and smoking [14]. Smoking was found not to be a confounder since it did not change the ORs by more than 15% [15], so it was removed from further analysis. Age was found to be a confounder in females and was therefore retained in female regression analyses. 

#### Logistic regression for odds ratios

Logistic regression was used to calculate odds ratios (ORs), and corresponding 95% confidence intervals (CIs) and two-tailed p values (with p values <0.05 considered significant). Statistical results were reported only in groups which contained 5 or more cases and controls [16]. In occupational titles with fewer than 5 SALS or control individuals the percentages of cases and controls were calculated to give an indication of their relative frequencies.

### Risk of SALS in occupational groups

The occupational titles within the groups of ANZSCO and ISCO are ranked hierarchically, based on skills and tasks, so the risk of SALS was able to be related to these ranks of occupational skills and tasks. This was undertaken in males in the 8 titles in The ANZSCO Major Group and in 9 of the 10 titles in The ISCO Major Group (ISCO Title 0 was not used since this is a non-hierarchical military service group). Females were not compared in this ranking analysis since the numbers of females in some of the Major Groups were limited.

### Environmental exposures in occupations at-risk for SALS

Environmental exposures associated with the occupation found to be most at risk of SALS were compared with exposures associated with other at-risk occupations in the study, to see if any exposure was common to a number of occupations. The literature was also searched to see if any SALS at-risk occupations identified in this study had been reported to be associated with SALS in previous studies.

## Results

### Cases and controls

Completed questionnaires were received from 611 SALS patients (379 male, 232 female) and 775 controls (377 male, 398 female). The numbers of individuals who had classifiable ANZSCO occupations are listed in Table 1, together with the number of classifiable and unclassifiable occupations, mean number and range of classifiable occupations per individual, age of onset of SALS, site of onset of SALS, and the duration of the disease at the time the questionnaire was filled in (see Table S1, for the slightly different ISCO results). The response rate for returning the questionnaire was similar in both SALS patients and controls: of 647 SALS patients who gave blood for DNA analysis, 611 (94.4%) completed the questionnaire, and of 824 control patients who gave blood 775 (94.1%) completed the questionnaire.

**Table 1:** Male and female ANZSCO occupational questionnaire responses.

	SALS *N* (%) [mean] {SD} range	Control *N* (%) [mean] {SD} range
*Males*		
Individuals who completed a questionnaire	379 (100)	377 (100)
Individuals who had ≥1 classifiable occupation	372 (98.2)	363 (96.3)
Ages of individuals with ≥1 classifiable occupation	[61.5] {11.4} 30 to 90	[61.4] {12.2} 24 to 94
Individuals who had no classifiable occupation	7 (1.8)	14 (3.7)
Occupations listed	1181 (100)	1013 (100)
Classifiable occupations	1075 (91.8)	899 (89.9)
Classifiable occupations per individual	[2.7] 1 to 14	[2.4] 1 to 11
Age of SALS onset y	[61.9]{11.4}28 to 90	NA
Site of SALS onset	U (39), L (35), B (25), R (1)	NA
SALS duration y	[1.3]{1.5} 0.5 to 18	NA
Unclassifiable occupations	106 (8.2) 0 to 4	114 (10.1) 0 to 4
*Females*		
Individuals who completed a questionnaire	232 (100)	398 (100)
Individuals who had ≥1 classifiable occupation	228 (98.3)	389 (97.7)
Ages of individuals with ≥1 classifiable occupation	[65.0] {11.2} 27 to 100	[58.5] {11.1} 28 to 86
Individuals who had no classifiable occupation	4 (1.7)	9 (2.3)
Occupations listed	611 (100)	1054 (100)
Classifiable occupations	558 (91.3)	1003 (95.2)
Classifiable occupations per individual	[2.7] 1 to 7	[2.7] 1 to 11
Unclassifiable occupations	53 (8.7) 0 to 4	51 (4.8) 0 to 4
Age of ALS onset y	[61.7] {11.8} 26 to 99	NA
Site of ALS onset	U (27), L (35), B (38), R (0)	NA
ALS duration y	[1.3]{1.5} 0.5 to 13	NA

The similar results for ISCO are shown in Table S1.

ALS duration: duration of ALS between diagnosis and filling in the questionnaire. U: upper limb, L: lower limb, B: bulbar, R: respiratory. NA: not applicable.

### Diligence in completing the questionnaire

The mean number of occupations per person was similar in SALS patient and control groups (Table 1). It therefore appears that SALS patients and controls put in a similar degree of effort in answering this question, and by extrapolation suggests that a similar amount of effort was put into answering the entire questionnaire.

### Risk of SALS in occupations

#### A. Occupations with 5 or more individuals

Odds ratios, confidence intervals, and p-values for all occupational titles where 5 or more SALS and control individuals were present are listed for both ANZSCO and ISCO in Table S2. Those occupational titles with statistically-significant ORs for SALS are listed below. 

1. Major Group

*Males*

In ANZSCO, Professionals (OR 0.59) had a decreased risk of SALS. Technicians and Trade Workers (OR 1.47), Machinery Operators and Drivers (OR 1.96), and Labourers (OR 1.72) had increased risks of SALS. 

In ISCO, Managers (OR 0.67) and Professionals (OR 0.56) had decreased risks of SALS. Machinery Operators and Drivers (OR 1.59), Labourers (OR 1.68), and Elementary Occupations (OR 2.07) had increased risks of SALS.

**Figure 1:**
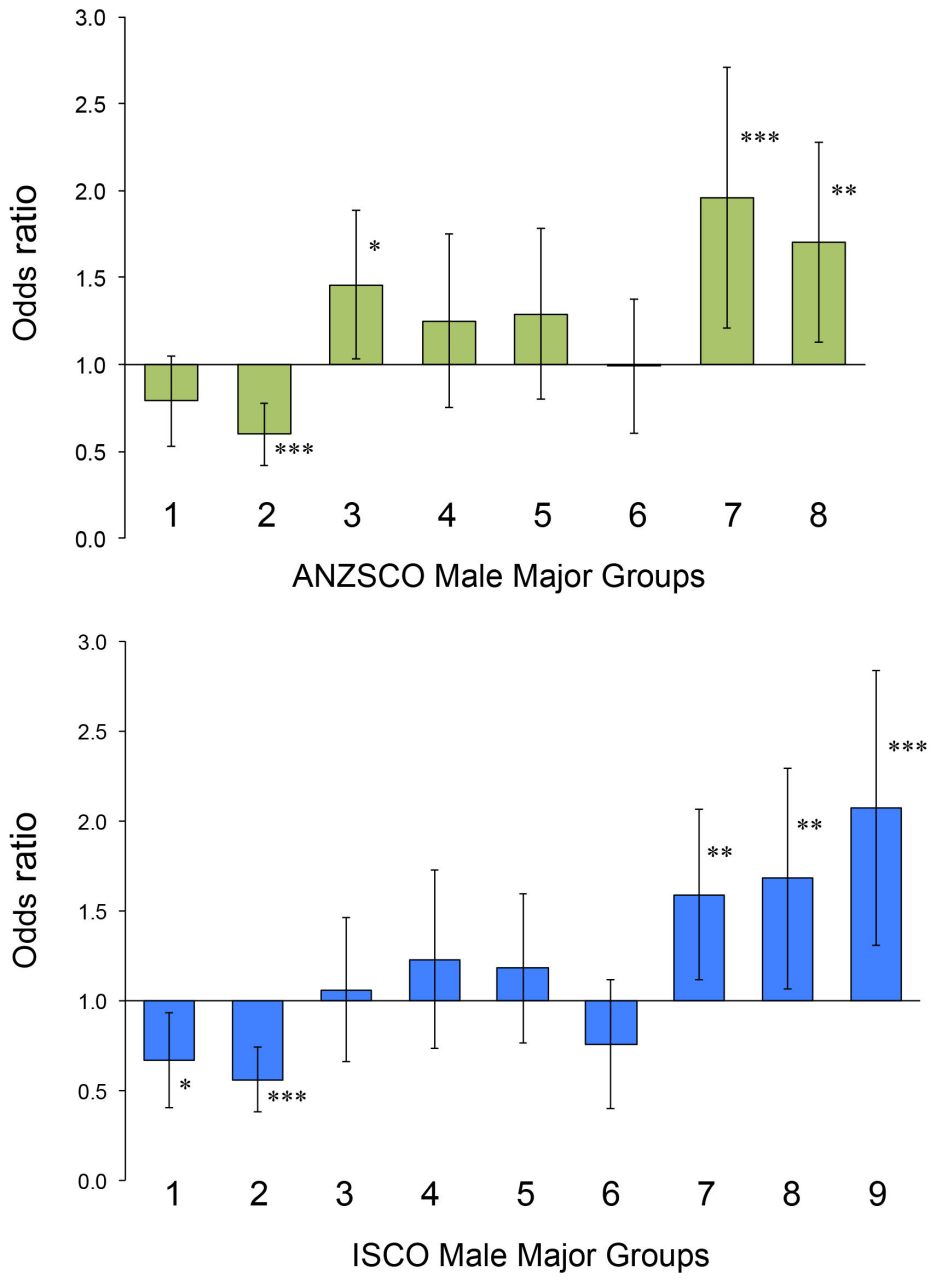
The risk of SALS in male Major Occupational Groups. In both ANZSCO and ISCO classifications, occupations with higher skills/tasks (on the left of the graphs) tended to have lower risks of SALS, and those with lower skills/tasks (on the right of the graphs) higher risks of SALS. 1 = Managers, 2 = Professionals, 3 = Technicians and Trade Workers, 4 = Community and Personal Service Workers, 5 = Clerical and Administrative Workers, 6 = Sales Workers, 7 = Machinery Operators and Drivers, 8 = Labourers, 9 = Elementary Occupations. Bars = 95% confidence internals. * = p <0.05, ** = p<0.01, *** = p<0.001.

The trend for ANZSCO and ISCO male Major Group occupations which require higher skills and tasks to have a reduced risk of SALS, and conversely those occupations requiring lower skills and tasks to have an increased risks of SALS, can be seen in Figure 1.

*Females*

In both ANZSCO and ISCO, no ORs for these occupational titles reached significance. 

2. Submajor Group

*Males*

In ANZSCO, Chief Executives, General Managers and Legislators (OR 0.32), and Business, Human Resource and Marketing Professionals (OR 0.41) had decreased risks of SALS. Electrotechnology and Telecommunications Trades Workers (OR 1.95), Road and Rail Drivers (OR 2.05), Construction and Mining Labourers (OR 2.50), Farm, Forestry and Garden Workers (OR 1.98), and Other Labourers (OR 2.10) had increased risks of SALS. 

In ISCO, Business and Administration Professionals (OR 0.46), and Information and Communications Technology Professionals (OR 0.28) had decreased risks of SALS. Personal Service Workers (OR 3.18), Building and Related Trades Workers, excluding Electricians (OR 1.79), Electrical and Electronic Trades Workers (OR 2.10), Stationary Plant and Machine Operators (OR 1.60), Drivers and Mobile Plant Operators (OR 1.78), Agricultural, Forestry and Fishery Labourers (OR 2.17) and Labourers in Mining, Construction, Manufacturing and Transport (OR 1.91) had increased risks of SALS.

*Females*

In both ANZSCO and ISCO, no ORs for these occupational titles reached significance. 

3. Minor Group

*Males*

In ANZSCO, Chief Executives, General Managers and Legislators (OR 0.32) had decreased risks of SALS. Fabrication Engineering Trades Workers (OR 2.92), Truck Drivers (OR 2.27), Construction and Mining Labourers (OR 2.50), Food Process Workers (OR 3.19), Farm, Forestry and Garden Workers (OR 1.98), and Miscellaneous Labourers (OR 2.32) had increased risks of SALS. 

In ISCO, Finance Professionals (OR 0.43) had a decreased risk of SALS. Building Frame and Related Trades Workers (OR 2.11), Heavy Truck and Bus Drivers (OR 1.99), Agricultural, Forestry and Fishery Labourers (OR 2.18), and Mining and Construction Labourers (OR 2.47) had increased risks of SALS.

*Females*

Female Cooks (OR 3.65) in ISCO had increased risks of SALS. No statistically-significant ORs for these occupational titles were found in ANZSCO.

4. Unit Group

*Males*

In ANZSCO, Accountants (OR 0.32) had a decreased risk of SALS, while Truck Drivers (OR 2.26) had an increased risk of SALS. 

In ISCO, Accountants (OR 0.31) had a decreased risk of SALS, while Heavy Truck and Lorry Drivers (OR 2.10), and Building Construction Labourers (OR 3.31) had increased risks of SALS. 

*Females*

Cooks had an increased risk of SALS in both ANZSCO (OR 3.64) and ISCO (OR 3.65).

5. Occupation Group (ANZSCO only)

*Males*

In ANZSCO, Truck Drivers had an increased risk of SALS, with 30 SALS (8.1%) and 13 control (3.6%) individuals being truck drivers (OR 2.36, 95% CI 1.21 to 4.59, p = 0.012).

*Females*

No statistically-significant female ORs were found in these occupational titles.

#### B. Occupations with fewer than 5 individuals

The relative frequency of occupational titles with fewer than 5 SALS or control individuals are available in Spreadsheet S1 for males, and Spreadsheet S2 for females. The percentage difference between SALS and controls for each ANZSCO occupation can be seen in ascending or descending order by sorting the Frequency Difference column on the Excel spreadsheets; negative values indicate decreased frequencies of SALS, and positive values increased frequencies of SALS. 

In males, occupations that appeared to be particularly more frequent for SALS were Metal Fabricator, Cook, Builder's Labourer, Delivery Driver and Fencer; those particularly less frequent for SALS were Accountant, Chief Executive or General Manager, and Sales and Marketing Manager. In females, occupations that were particularly more frequent for SALS were Retail Manager, Cook, and Fruit or Nut Picker; those particularly less frequent for SALS were Bar Attendant, and Nanny.

### Environmental exposures in occupations at-risk for SALS

#### Current study

Truck driving in males was the only occupation to retain a statistically significant increased risk of SALS down to the ANZSCO individual Occupation Group. This was therefore the occupation considered to be most at risk of SALS. Table 2 summarises the OR statistics in occupational groups containing truck drivers. 

**Table 2:** SALS risk in ANZSCO male truck drivers.

Group	Code	Occupational title	SALS N (%)	Controls N (%)	OR	95% CI of OR	p value
Major	7	Machinery Operators and Drivers	95 (25.5)	54 (14.9)	1.96	1.35 to 2.84	<0.001
Submajor	73	Road and Rail Drivers	51 (13.7)	26 (7.2)	2.05	1.25 to 3.37	0.005
Minor	733	Truck Drivers	31 (8.3)	14 (3.9)	2.26	1.18 to 4.32	0.014
Unit	7331	Truck Drivers	31 (8.3)	14 (3.9)	2.26	1.18 to 4.32	0.014
Occupation	733111	Truck Driver (General)	30 (8.1)	13 (3.6)	2.36	1.21 to 4.59	0.012

All groups in which truck drivers appear, even in the most detailed group with the lowest number of subjects, are associated with a risk of SALS. OR: odds ratio, CI: confidence interval.

Diesel exhaust exposure was considered to be the most likely environmental factor in truck drivers that would predispose them to SALS (see Discussion). The literature was therefore searched on the other occupations that had an increased risk of SALS in this study to see if these occupations also had potential exposure to diesel exhaust. Of these 16 other occupations and occupational groups, 11 (69%) have been reported to have possible occupational exposure to diesel exhaust (Table 3). One (6%, Cooks) had potential exposure to particulates in air but only occasionally to diesel itself. Four occupational titles (25%) included too many and varied occupation types (e.g., Labourers) to assess for environmental exposures.

**Table 3:** Occupations (ANZSCO group 5) and other occupational groups (ANZSCO groups 1-4) at risk of SALS in the present study, with reported diesel exhaust exposures.

Occupation with increased SALS risk (present study)	ANZSCO groups	Reported diesel exhaust exposure in occupations in the present study	Occupation with increased SALS risk (past studies)
Truck Drivers	5,4,3	Truck drivers [19,21-24]	Truck drivers [17]
Cooks (female)	4	Diesel cook stoves	NA
Farm, Forestry and Garden Workers	3,2	Farm equipment [23]	Farmers [4,51,52]
Construction and Mining Labourers	3,2	Construction equipment [23,24]; heavy equipment operators [23]; mining [19,24]; mining [53]; coal mining [54]	Construction workers (excluding supervisors) [10]; metal exposure [46]
Fabrication Engineering Trades Workers	3	Heavy equipment operators [23,24]	NA
Miscellaneous Labourers	3	Too broad to assess	NA
Labourers in Mining, Construction, Manufacturing and Transport	2	Mining [19,24]; construction equipment [23]; heavy equipment operators [23]	Construction workers (excluding supervisors) [10]; metal exposure [46]
Agricultural, Forestry and Fishery Labourers	2	Farm equipment [23]; marine equipment [23,24]	Farmers [4,51,52]
Stationary Plant and Machine Operators	2	Heavy equipment operators [23]	Precision production [10]; machine workers [4]; machine operators [7]
Drivers and Mobile Plant Operators	2	Motor vehicles [23,24]; heavy equipment operators [23,24]	Bus drivers [4]
Road and Rail Drivers (*includes truck drivers*)	2	Motor vehicles [23,24]; railway locomotives [23,24]	Truck drivers [17]; bus drivers [4]
Building and Related Trades Workers	2	Construction equipment [23]	NA
Electrotechnology and Telecommunications Trades Workers	2	Too broad to assess	Electrical workers [55]
Other Labourers	2	Too broad to assess	
Machinery Operators and Drivers (*includes truck drivers*)	1	Motor vehicles [23]	Machine workers [4]; machine operators [7]
Labourers	1	Too broad to assess	NA

The column on the right refers to past studies of associations of these occupations with SALS. NA: not applicable.

#### Previous studies

Of the 11 occupational titles in this study with potential exposure to diesel exhaust, 10 (91%) have been reported in previous epidemiological studies to be at increased risk of SALS (Table 3).

## Discussion

Two major findings have come from this study of lifetime occupations as potential risk factors for SALS. Firstly, occupations requiring higher skills and tasks tended to have decreased risks of SALS, whereas occupations requiring lower skills and tasks tended to have increased risks of the disease. Secondly, the majority of occupations with significantly increased risks of SALS, and in particular truck drivers, were likely to be exposed to diesel exhaust during their working hours. Of note, occupations with decreased risks of SALS, being mostly managerial and indoors, would be less likely to be exposed to diesel exhaust.

The stand-out occupation in this study as regards an association with SALS was truck driving. This was the only occupation to remain positively associated with SALS down to the smaller numbers of individuals who were present in the most detailed ANZSCO occupational group. Of interest, a 1980 study of 504 World War II veterans with ALS and 504 matched military controls also found that truck (and tractor) driving was the only individual occupation that had excess numbers of ALS patients [17]. Truck drivers are exposed to a number of potentially injurious environmental agents [18], but the most studied of these has been exposure to diesel exhaust [19-25]. The predominance of diesel use in trucks can be seen in figures from the Australian Bureau of Statistics Motor Vehicle Census for 2012 (www.abs.gov.au/ausstats/abs@.nsf/mf/9309.0), which shows that the percentage of passenger vehicles using diesel was 7%, light commercial vehicles 44%, campervans 62%, buses 76%, non-freight carrying trucks 83%, light rigid trucks 91%, and articulated trucks 98%.

Factors contributing to the level of diesel exhaust exposure of truck drivers are whether windows are open or closed (with higher exposure when windows are open), warmer compared to cooler weather (with warmer weather increasing the exposure) and exposure during daytime city driving (when exposure is higher than during long-haul driving in the evening on suburban or rural roads) [24]. The age of the truck also contributes to the exposure, since fuel exhaust can leak through the rubber seals of the driver compartment in older trucks [22]. 

Diesel exhaust is composed of a complex mixture of gases and particulates [20,24]. A number of components of the exhaust, such as formaldehyde, benzene, hexane, and styrene may be involved in the pathogenesis of SALS [25]. Formaldehyde exposure has been associated with a risk of SALS in two studies [26,27]. Derivatives of both benzene and hexane cause neurofilamentous proximal axonal swellings in rodents [28], and proximal axonal swellings filled with neurofilaments have long been recognised as a pathological feature of ALS [29]. Some of these toxic agents may interact with genetic variants in people who are susceptible to SALS. For example, the cytotoxicity of the glutathione-depleting chemical styrene oxide varies with low or high superoxide dismutase 1 (SOD1) activity [30], and *SOD1* mutations are one cause of familial ALS [31]. The potential neurotoxicity of a large number of components of hydrocarbon fuels has been reviewed by Ritchie et al. (2001) [20]

Experimental studies indicate that diesel exhaust can have deleterious effects on the CNS, including upregulation of the proinflammatory cytokine TNF-alpha [32] which can enhance motor neuron susceptibility to the excitotoxins [33] that are suspected to play a part in ALS [34], and upregulation of microglia [35] which also occurs in ALS [36]. Evidence concerning air pollution-induced neuroinflammation and alterations to the blood-brain barrier has been reviewed by Genc et al. (2012) [37].

Truck drivers were not the only workers in this study who had an increased SALS risk combined with a potential exposure to diesel exhaust. Eleven other occupations and occupational groups at risk of SALS took place in workplaces where diesel exhaust has been reported to be a potential hazard, and ten of these have previously been associated with SALS. Although Truck Drivers dominate the numbers in their respective ANZSCO Occupation, Unit, and Minor Groups, they are only a moderate part of The Submajor Group, and a small part of the Major Group. However, the p values for the odds ratio of the latter two occupations were smaller than the p values of the occupational titles that contain Truck Drivers alone (Table 2), suggesting that non-truck driver occupations within these titles also have an increased risk of SALS, possibility because related occupations in these groups are also likely to have exposure to diesel exhaust. 

Diesel exhaust is not the only occupational hazard that truck drivers are exposed to. They are also prone to sleep apnoea [18], which can lead to intermittent hypoxia. Low ambient oxygen levels have been suggested to be a possible reason for an increased incidence of SALS in airline pilots [38], though exposure to jet fuel, or to diesel fuel used in aircraft refuelling trucks, could also be a reason for this increased risk [24]. However, in none of the other at-risk occupations in our study was there any indication that intermittent hypoxia is part of the workplace environment. It has been suggested that intermittent hypoxia could be a cause of the increased risk of SALS reported in fire fighters [39], but this increased risk could equally be due to diesel exhaust from fire trucks or inhalation of particulate matter from smoke exposure. Obesity is another disorder seen often in truck drivers [18], but in SALS an inverse relationship applies, with patients usually having a lower premorbid body mass index than controls [40].

Occupational exposure to diesel and other hydrocarbon fuel exhaust could explain the puzzling increase in SALS in military personnel. Three US studies have reported a doubling of SALS incidence after military service [5,41,42], and military personnel who saw active service had an increased risk of SALS compared to those who did not [42]. All service branches (army, navy and air force) had the increased SALS risk, despite being engaged in different duties, suggesting some common factor underlay the increased risk. Although two of the studies involved Persian Gulf war veterans [41,42], the third reported on servicemen before 1990 [5], which suggests that the SALS risk is not related to any particular war zone, but to the experience of war itself. One review of hydrocarbon fuel exposure, which paid particular attention to military uses, emphasised the widespread use of diesel and other hydrocarbon fuels in all service branches of the military during combat [20]. As well as the use of these fuels to power land, sea and air military vehicles, a number of unique applications lead to increased hydrocarbon fuel exposure. These include desert sand suppression with jet fuel during the Persian Gulf war (with rapid vaporisation of the fuel at the high local temperature and entry deep into the lungs when combined with fine desert sand), use of diesel fuel aerosols to visually obscure troops and equipment, diesel fuelling of unvented military tent heaters, dermal exposure to hydrocarbon fuels due to contamination of shower water, and the use of jet fuel as aircraft heat sinks [20]. Apart from accidental spills, possibly the greatest respiratory exposure to hydrocarbon fuel is suffered by military fuel workers operating without respiratory protection below the open access hatches of aircraft fuel tanks [20]. It is therefore of interest that the deployed:non-deployed risk ratio for SALS after the Persian Gulf war was higher in air force personnel than in other service branches [42]. Unfortunately, neither ANZSCO nor ISCO has adequate classifications for active military service, so this could not be assessed in the present study.

Exposure to diesel exhaust could explain the observation that SALS is more common in higher, compared to lower, population densities [43], because diesel fume exposure is higher in inner city areas compared to suburban or rural areas [21]. On the other hand, the incidence of SALS is almost identical in men and women, with only a slight male predominance in most studies [44], which argues against a link to diesel exhaust, since most of the occupations exposed to diesel exhaust are undertaken by men. Another contrary finding is that the use of diesel engines has been increasing over time in many countries. For example, heavy trucks in the US switched to diesel engines in the 1950s [24], and in Australia the number of registered diesel motor vehicles has been increasing, with the latest Australian Bureau of Statistics figures showing an increase of 61% from 2007 to 2012. Most investigators, however, now agree that the incidence of SALS remains stable over time [44], which would not be expected if an increasingly common environmental toxicant was playing a part in SALS. This is a complex issue, however, since although diesel fuel use has been increasing, the types of diesel fuels have changed over time [20], and more efficient filters are now being used in diesel-powered vehicles. 

As regards methodology, advantages of this study were that: (1) this is the largest case-control study of occupation in SALS to date; (2) controls were closely matched, limiting the likelihood of type I statistical errors; (3) two occupational classifications were used, which allowed classification of individual occupations, as well as allowing comparisons with other international studies; (4) an accurate diagnosis of SALS could be made from detailed neurological reports; and (5) having access to lifetime occupations allowed consideration of all (not only ‘usual’) occupations as potential risk factors. 

Limitations of the study were that: (1) no corrections were made for multiple testing. We acknowledge that with this large number of occupations some will reach statistical significance by chance only. Rather than risk discarding potentially important results by correcting for multiple testing, however, we chose to examine all occupations that had significant associations with SALS for plausible biological reasons that could underlie these associations [45]; (2) females had fewer types of occupation, so numbers of individuals were limited in some female groups; (3) domestic duties were listed as an occupation by many (mostly female) individuals, and we had to assume that certain duties were undertaken as part of these duties to fit the classification codes (see Methods); (4) a number of individuals listed occupations that could not be classified into either of the two systems. The percentage of these was low and similar in cases and controls, and this factor was therefore unlikely to affect the results; (5) neither ANZSCO or ISCO could assess military duties in detail. For example, neither active military service, nor whether service was in the army, navy or air force, could be determined; (6) a difference in levels of education between patients and controls could lead to differences in the way the questionnaire was completed. However, as has been noted before in relation to this cohort [46], in Australia the level of literacy is high (http://www.abs.gov.au/ausstats), with free compulsory education until the age of 17 years. Close matching of cases and controls (e.g., partners) would add to the likelihood of similar education and social levels. Furthermore, a subset of ALS patients and controls from this cohort has previously been shown to have similar levels of education [46]. Finally, occupation itself is a major indicator of socioeconomic status [47] so when one is comparing disease rates between occupations they are likely to be from similar socioeconomic levels; (7) Voluntary participation in filling in the questionnaire could cause selection bias by favouring patients who were keen to find the underlying cause of their disease, and thus might be more prone to recall bias. No correction could be made for this, since no information was available on the size of the potential patient population for this study (no Australia-wide study of ALS incidence and prevalence has been published) [46].

Many people are exposed to environmental toxicants such as diesel exhaust, yet few get SALS, so simple exposure alone is unlikely to lead to the disease. Individual genetic susceptibility to one or more of the many toxicants in hydrocarbon fuels has been postulated as a reason why only some people exposed to these fuels suffer neurotoxic effects [20]. It has recently been suggested that stress increases the uptake of toxicants into human corticomotor neurons, and that this might lead to SALS [48]. Many of the occupations in this study that had an increased risk of SALS, as well as exposure to diesel exhaust, have been reported to be stressful, including truck driving [49]. A study examining stressors that occur at the same time as exposure to diesel exhaust could be one future avenue to explore in the search for underlying causes of SALS. Another possible study would be to assess early-life exposure to diesel exhaust as a possible risk factor for the later onset of SALS. This could be done by looking at the occupations of parents whose offspring later get SALS. Of interest, an association between parental occupational exposure to diesel exhaust and the onset of childhood brain tumours has recently been described [50].

In conclusion, two of the largest case-control studies of SALS have now found that truck drivers have a uniquely increased risk of the disease. Exposure to diesel exhaust is common in truck drivers, and exposure to this toxicant, as well as other hydrocarbon fuels, may underlie some of the epidemiological findings related to SALS. 

## Supporting Information

Figure S1 **The numbers of occupational titles in the hierarchical groups in the ANZSCO and ISCO classifications.**
(TIF)

Figure S2 **A flow-chart showing how one occupation, a sonographer, is coded in the ANZSCO and ISCO classifications.**
(TIF)

Methods S1 **An example of the R script for the Male ISCO Minor Group, used to prevent repeated occupations in individuals from being counted more than once.**
(DOCX)

Spreadsheet S1 **The relative frequency of male occupational titles with fewer than 5 SALS or control individuals.**
(XLS)

Spreadsheet S2 **The relative frequency of female occupational titles with fewer than 5 SALS or control individuals.**
(XLS)

Table S1 **Male and female ISCO occupational questionnaire responses.**
(DOC)

Table S2 **Odds ratios, confidence intervals, and p-values for all ANZSCO and ISCO occupational titles where 5 or more SALS and control individuals were present.**
(DOC)

Classification S1 **ANZSCO occupational codes.**
(PDF)
