# Supplementary figures and images for: Different Occupations Associated with Amyotrophic Lateral Sclerosis: Is Diesel Exhaust the Link?
Source: PLoS One. 2013 Nov 11;8(11):e80993. doi: 10.1371/journal.pone.0080993 (PMC3823610; doi:10.1371/journal.pone.0080993)

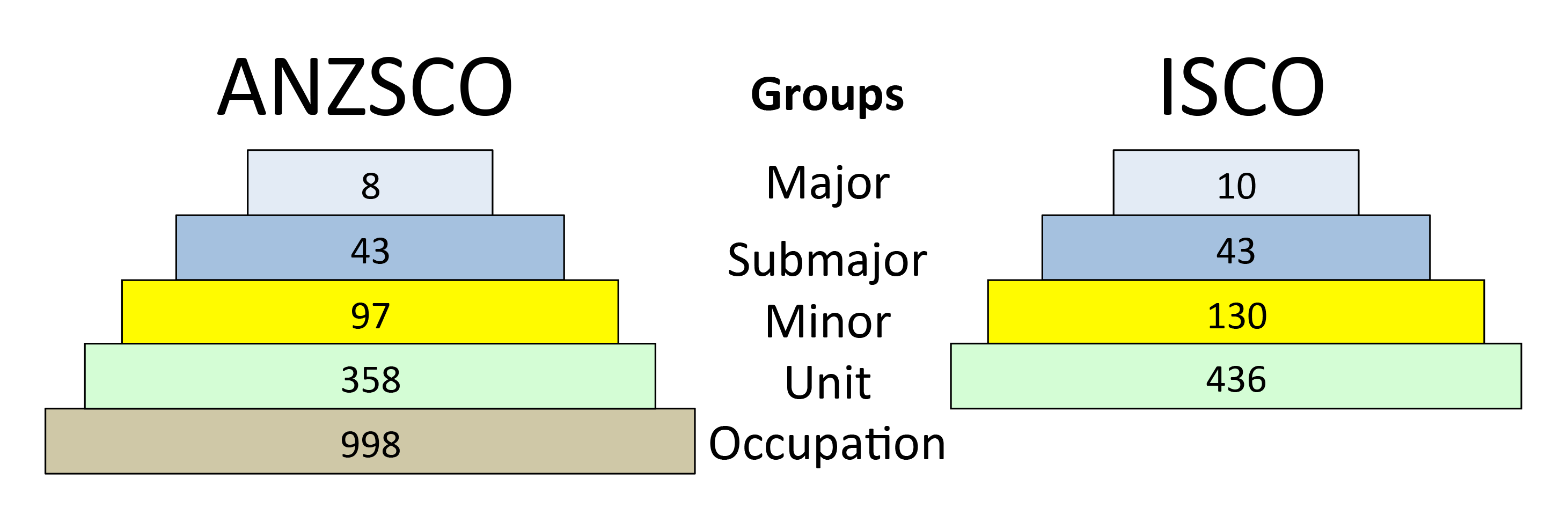

Supplement: Figure S1 — The numbers of occupational titles in the hierarchical groups in the ANZSCO and ISCO classifications. (TIF) [file pone.0080993.s001.tif]

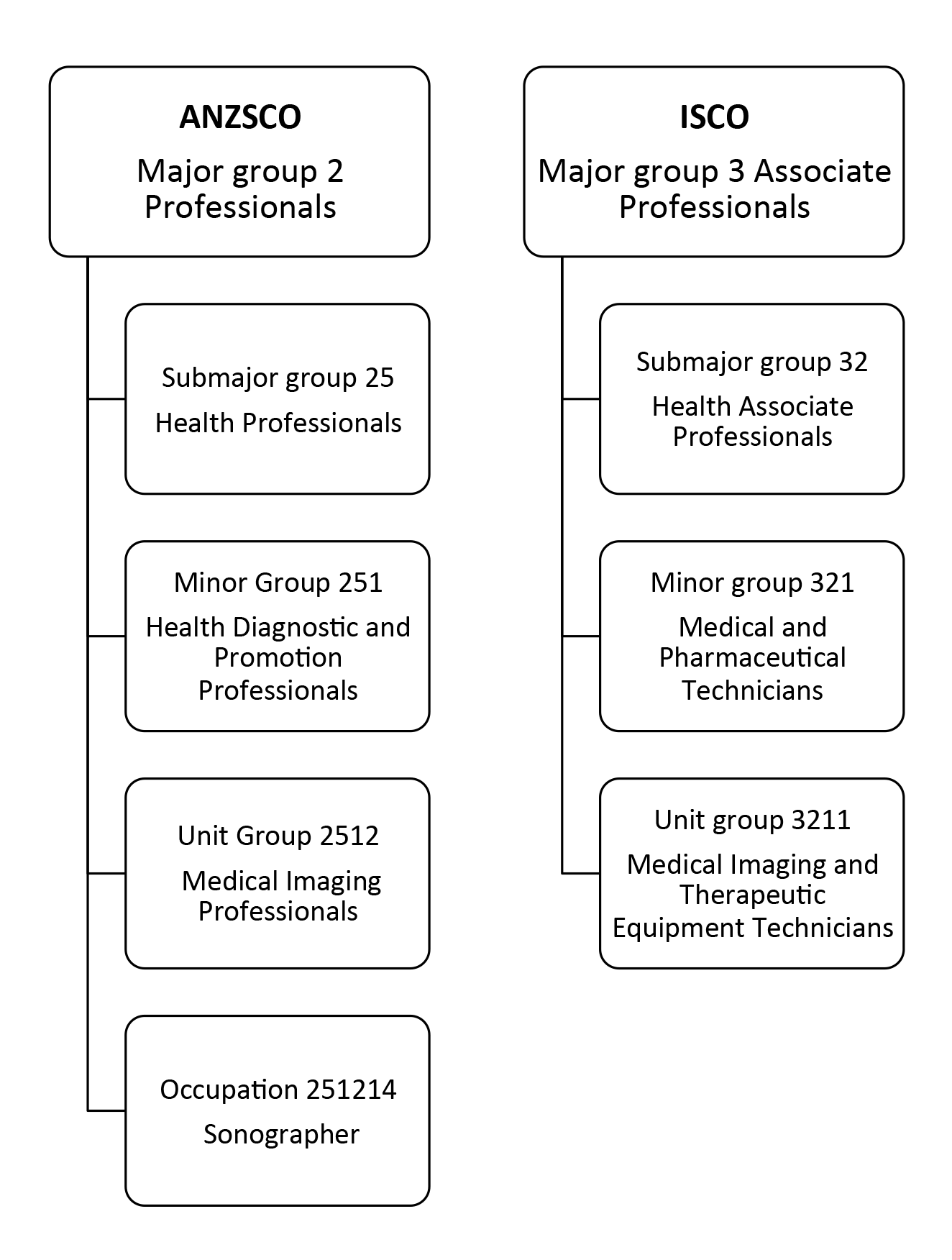

Supplement: Figure S2 — A flow-chart showing how one occupation, a sonographer, is coded in the ANZSCO and ISCO classifications. (TIF) [file pone.0080993.s002.tif]
