# Supplementary material for: Different Occupations Associated with Amyotrophic Lateral Sclerosis: Is Diesel Exhaust the Link?
Source: PLoS One. 2013 Nov 11;8(11):e80993. doi: 10.1371/journal.pone.0080993 (PMC3823610; doi:10.1371/journal.pone.0080993)
Supplement: Methods S1 — An example of the R script for the Male ISCO Minor Group, used to prevent repeated occupations in individuals from being counted more than once. (DOCX) [file pone.0080993.s003.docx]

**Methods S1**

**R script to remove repeat occupations in Male ISCO Minor Group**

stack.occupation<-function(groups.list,dat){

temp.matrix<-matrix(ncol=length(groups.list)+7,nrow=length(unique(dat$ID.no.Bank)))

data.frame.names<-c("id","age","SMND Type",groups.list)

output<-as.data.frame(temp.matrix)

names(output)<-data.frame.names

id.list<-unique(dat$ID.no.Bank)

output$id<-id.list

for(i in 1:nrow(output)){

temp.subset<-subset(dat,ID.no.Bank==id.list[i])

output$age[i]<-unique(temp.subset$Age.at.blood.sampling)

output$"SMND Type"[i]<-unique(temp.subset$Group.III.SMND.types)

for(j in 1:length(groups.list)){

col.index<-which(data.frame.names==groups.list[j])

output[i,col.index]<-ifelse(length(grep(groups.list[j],temp.subset$MAJ.GROUP))>0,T,F)

}

}

return(output)

}

dat<-read.csv("//Users//annarikard-bell//Documents//DATA//INT Male Minor 091012.csv",header=T)

dat$Group.III.SMND.types<-as.character(dat$Group.III.SMND.types)

dat$MIN.GROUP<-as.character(dat$MAJ.GROUP)

#MINOR GROUP INDEX

groups.list<-c("121","132","141","142","214","216","241","265","311","332","333","352","411","421","431","513","522","541","711","712","713","721","723","732","741","751","752","811","816","817","832","833","921","931","962")

flat.pack.occupation<-stack.occupation(groups.list,dat)

write.csv(flat.pack.occupation,"//Users//annarikard-bell//Documents//DATA//201012//INT Minor 2010122.csv",row.names=F)
