# Supplementary material for: Different Occupations Associated with Amyotrophic Lateral Sclerosis: Is Diesel Exhaust the Link?
Source: PLoS One. 2013 Nov 11;8(11):e80993. doi: 10.1371/journal.pone.0080993 (PMC3823610; doi:10.1371/journal.pone.0080993)
Supplement: Table S1 — Male and female ISCO occupational questionnaire responses. (DOC) [file pone.0080993.s006.doc]

**Table S1.** Occupation and age details of ISCO males and females.

|  | SALS  N (%) [mean] {SD} range | Control  N (%) [mean] {SD} range |
| --- | --- | --- |
| *Males* |  |  |
| Individuals who completed a questionnaire | 379 (100) | 377 (100) |
| Individuals who had ≥1 classifiable occupation | 372 (98.2) | 361 (95.8) |
| Ages of individuals with ≥1 classifiable occupation | [61.5] {11.4} 30 to 90 | [62.5] {12.6} 27 to 94 |
| Individual who had no classifiable occupation | 7 (1.8) | 15 (4.0) |
| Occupations listed | 1182 (100) | 1016 (100) |
| Classifiable occupations | 1168 (98.8) | 993 (97.7) |
| Classifiable occupations per individual | [3.1] 1 to 14 | [2.8] 1 to 11 |
| Unclassifiable occupations | 14 (1.2) 0 to 2 | 23 (2.3) 0 to 3 |
| *Females* |  |  |
| Individuals who completed a questionnaire | 232 (100) | 398 (100) |
| Individuals who had ≥1 classifiable occupation | 228 (98.3) | 390 (98.0) |
| Ages of individuals with ≥1 classifiable occupation | [65.0] {11.2} 27 to 100 | [58.5] {11.1} 28 to 86 |
| Individual who had no classifiable occupation | 4 (1.7) | 3 (0.8) |
| Occupations listed | 611 (100) | 1071 (100) |
| Classifiable occupations | 606 (99.2) | 1065 (99.4) |
| Classifiable occupations per individual | [2.7] 1 to 8 | [2.7] 1 to 11 |
| Unclassifiable occupations | 5 (0.8) 0 to 1 | 6 (0.6) 0 to 1 |
