# Supplementary material for: Different Occupations Associated with Amyotrophic Lateral Sclerosis: Is Diesel Exhaust the Link?
Source: PLoS One. 2013 Nov 11;8(11):e80993. doi: 10.1371/journal.pone.0080993 (PMC3823610; doi:10.1371/journal.pone.0080993)
Supplement: Table S2 — Odds ratios, confidence intervals, and p-values for all ANZSCO and ISCO occupational titles where 5 or more SALS and control individuals were present. (DOC) [file pone.0080993.s007.doc]

**Table 2:** Risk of SALS in occupations having 5 or more SALS and control individuals. Numbers between ANZSCO and ISCO vary slightly because of differences in the coding criteria. Occupations with significant odds ratios (ORs) are highlighted in yellow. Female OR = odds ratio adjusted for age, nec = not elsewhere classified. CI = confidence internal. * = p < 0.05, ** = p < 0.01, *** = p <0.001.

1a Male Major Groups

| ANZSCO Male Major Group | | SALS *N* (%) | Controls *N* (%) | OR | 95% CI of OR | p value |
| --- | --- | --- | --- | --- | --- | --- |
| Code | Title | 372 (100) | 362 (100) |
| 1 | Managers | 91 (24.5) | 106 (29.3) | 0.78 | 0.56 to 1.09 | 0.141 |
| 2 | Professionals | 115 (30.9) | 156 (43.1) | 0.59 | 0.44 to 0.80 | <0.001*** |
| 3 | Technicians and Trade Workers | 181 (48.7) | 142 (39.2) | 1.47 | 1.10 to 1.97 | 0.010* |
| 4 | Community and Personal Service Workers | 68 (18.3) | 55 (15.2) | 1.25 | 0.85 to 1.84 | 0.264 |
| 5 | Clerical and Administrative Workers | 78 (21.0) | 62 (17.1) | 1.28 | 0.89 to 1.86 | 0.186 |
| 6 | Sales Workers | 65 (17.5) | 64 (17.7) | 0.99 | 0.67 to 1.44 | 0.941 |
| 7 | Machinery Operators and Drivers | 95 (25.5) | 54 (14.9) | 1.96 | 1.35 to 2.84 | <0.001*** |
| 8 | Labourers | 118 (31.7) | 77 (21.3) | 1.72 | 1.23 to 2.40 | 0.001** |

| ISCO Male Major Group | | SALS patients *N* (%) | Controls *N* (%) | OR | 95% CI of OR | p value |
| --- | --- | --- | --- | --- | --- | --- |
| Code | Title | 372 (100) | 361 (100) |
| 1 | Managers | 53 (14.2) | 72 (19.9) | 0.67 | 0.45 to 0.98 | 0.041* |
| 2 | Professionals | 102 (27.4) | 145 (40.2) | 0.56 | 0.41 to 0.77 | <0.001*** |
| 3 | Technicians and Trade Workers | 70 (18.8) | 65 (18.0) | 1.06 | 0.73 to 1.53 | 0.777 |
| 4 | Community and Personal Service Workers | 66 (17.7) | 54 (15.0) | 1.23 | 0.83 to 1.82 | 0.309 |
| 5 | Clerical and Administrative Workers | 91 (24.5) | 78 (21.6) | 1.18 | 0.83 to 1.66 | 0.359 |
| 6 | Sales Workers | 38 (10.2) | 47 (13.0) | 0.76 | 0.48 to 1.20 | 0.237 |
| 7 | Machinery Operators and Drivers | 177 (47.6) | 131 (36.3) | 1.59 | 1.19 to 2.14 | 0.002** |
| 8 | Labourers | 96 (25.8) | 62 (17.2) | 1.68 | 1.17 to 2.40 | 0.005** |
| 9 | Elementary Occupations | 104 (28.0) | 57 (15.8) | 2.07 | 1.44 to 2.97 | <0.001*** |
| 0 | Armed Forces Occupations | 102 (27.4) | 92 (25.5) | 1.11 | 0.80 to 1.52 | 0.553 |

1b Female Major Groups

| ANZSCO Female Major Groups | | SALS *N* (%) | Controls *N* (%) | OR | 95% CI of OR | p value |
| --- | --- | --- | --- | --- | --- | --- |
| Code | Title | 228 (100) | 389 (100) |
| 1 | Managers | 87 (38.2) | 156 (40.1) | 0.93 | 0.66 to 1.32 | 0.674 |
| 2 | Professionals | 153 (67.1) | 297 (76.3) | 0.73 | 0.50 to 1.06 | 0.097 |
| 3 | Technicians and Trade Workers | 85 (37.3) | 134 (34.4) | 1.32 | 0.92 to 1.89 | 0.131 |
| 4 | Community and Personal Service Workers | 89 (39.0) | 170 (43.7) | 1.00 | 0.71 to 1.42 | 0.992 |
| 5 | Clerical and Administrative Workers | 147 (64.5) | 259 (66.6) | 1.02 | 0.71 to 1.45 | 0.937 |
| 6 | Sales Workers | 88 (38.6) | 165 (42.4) | 0.91 | 0.64 to 1.28 | 0.572 |
| 7 | Machinery Operators and Drivers | 16 (7.0) | 25 (6.4) | 0.93 | 0.48 to 1.82 | 0.837 |
| 8 | Labourers | 77 (33.8) | 128 (32.9) | 1.05 | 0.73 to 1.50 | 0.802 |

| ISCO Female Major Groups | | SALS *N* (%) | Controls *N* (%) | OR | 95% CI of OR | p value |
| --- | --- | --- | --- | --- | --- | --- |
| Code | Title | 228 (100) | 390 (100) |
| 1 | Managers | 22 (5.6) | 42 (11.2) | 1.20 | 0.67 to 2.13 | 0.540 |
| 2 | Professionals | 56 (14.2) | 109 (29) | 0.96 | 0.65 to 1.41 | 0.822 |
| 3 | Technicians and Trade Workers | 33 (8.4) | 83 (22.1) | 0.74 | 0.47 to 1.17 | 0.198 |
| 4 | Community and Personal Service Workers | 109 (27.7) | 196 (52.1) | 0.91 | 0.65 to 1.28 | 0.576 |
| 5 | Clerical and Administrative Workers | 93 (23.7) | 162 (43.1) | 1.12 | 0.79 to 1.58 | 0.530 |
| 6 | Sales Workers | 12 (3.1) | 16 (4.3) | 1.12 | 0.51 to 2.47 | 0.779 |
| 7 | Machinery Operators and Drivers | 19 (4.8) | 26 (6.9) | 1.12 | 0.59 to 2.12 | 0.730 |
| 8 | Labourers | 28 (7.1) | 31 (8.2) | 1.53 | 0.88 to 2.67 | 0.135 |
| 9 | Elementary Occupations | 66 (16.8) | 108 (28.7) | 1.07 | 0.73 to 1.56 | 0.724 |
| 0 | Armed Forces Occupations | 40 (10.2) | 48 (12.8) | 1.56 | 0.96 to 2.55 | 0.075 |

2a Male Submajor Groups

| ANZSCO Male Submajor Groups | | SALS patients *N* (%) | Controls *N* (%) | OR | 95% CI of OR | p value |
| --- | --- | --- | --- | --- | --- | --- |
| Code | Title | 372 (100) | 362 (100) |
| 11 | Chief Executive, General Managers and Legislators | 5 (1.3) | 15 (4.1) | 0.32 | 0.11 to 0.88 | <0.001*** |
| 12 | Farmers and Farm Managers | 33 (8.9) | 40 (11.0) | 0.78 | 0.48 to 1.27 | 0.325 |
| 13 | Specialist Managers | 27 (7.3) | 37 (10.2) | 0.69 | 0.41 to 1.16 | 0.157 |
| 14 | Hospitality, Retail and Service Managers | 28 (7.5) | 18 (5.0) | 1.56 | 0.85 to 2.87 | 0.156 |
| 21 | Arts and Media Professionals | 12 (3.2) | 13 (3.6) | 0.90 | 0.40 to 1.99 | 0.785 |
| 22 | Business, Human Resource and Marketing Professionals | 18 (4.8) | 40 (11.0) | 0.41 | 0.23 to 0.73 | 0.002** |
| 23 | Design, Engineering, Science and Transport Professionals | 49 (13.2) | 61 (16.9) | 0.75 | 0.5 to 1.13 | 0.164 |
| 24 | Education Professionals | 26 (7.0) | 23 (6.4) | 1.11 | 0.62 to 1.98 | 0.730 |
| 25 | Health Professionals | 11 (3.0) | 14 (3.9) | 0.76 | 0.34 to 1.69 | 0.498 |
| 26 | ICT Professionals | 9 (2.4) | 18 (5.0) | 0.47 | 0.21 to 1.07 | 0.072 |
| 27 | Legal, Social and Welfare Professionals | 5 (1.3) | 12 (3.3) | 0.40 | 0.14 to 1.14 | 0.086 |
| 31 | Engineering, ICT and Science Technicians | 19 (5.1) | 21 (5.8) | 0.87 | 0.46 to 1.65 | 0.679 |
| 32 | Automotive and Engineering Trades Workers | 59 (15.9) | 53 (14.6) | 1.10 | 0.74 to 1.64 | 0.646 |
| 33 | Construction Trades Workers | 48 (12.9) | 31 (8.6) | 1.58 | 0.98 to 2.55 | 0.059 |
| 34 | Electrotechnology and Telecommunications Trades Workers | 27 (7.3) | 14 (3.9) | 1.95 | 1.00 to 3.77 | 0.049* |
| 35 | Food Trades Workers | 19 (5.1) | 10 (2.8) | 1.90 | 0.87 to 4.13 | 0.108 |
| 36 | Skilled Animal and Horticultural Workers | 15 (4.0) | 10 (2.8) | 1.48 | 0.66 to 3.34 | 0.346 |
| 39 | Other Technicians and Trades Workers | 26 (7.0) | 25 (6.9) | 1.01 | 0.57 to 1.79 | 0.965 |
| 41 | Health and Welfare Support Workers | 6 (1.6) | 5 (1.4) | 1.17 | 0.35 to 3.87 | 0.796 |
| 43 | Hospitality Workers | 18 (4.8) | 8 (2.2) | 2.25 | 0.97 to 5.24 | 0.060 |
| 44 | Protective Services Workers | 35 (9.4) | 35 (9.7) | 0.97 | 0.59 to 1.59 | 0.905 |
| 45 | Sports and Personal Service Workers | 9 (2.4) | 6 (1.7) | 1.47 | 0.52 to 4.18 | 0.468 |
| 55 | Numerical Clerks | 19 (5.1) | 18 (5.0) | 1.03 | 0.53 to 1.99 | 0.933 |
| 56 | Clerical and Office Support Workers | 30 (8.1) | 21 (5.8) | 1.42 | 0.80 to 2.54 | 0.230 |
| 59 | Other Clerical and Administrative Workers | 7 (1.9) | 14 (3.9) | 0.48 | 0.19 to 1.20 | 0.114 |
| 61 | Sales Representatives and Agents | 17 (4.6) | 18 (5.0) | 0.92 | 0.46 to 1.81 | 0.798 |
| 62 | Sales Assistants and Salespersons | 50 (13.4) | 47 (13.0) | 1.04 | 0.68 to 1.60 | 0.855 |
| 71 | Machine and Stationary Plant Operators | 35 (9.4) | 23 (6.4) | 1.53 | 0.89 to 2.65 | 0.127 |
| 72 | Mobile Plant Operators | 6 (1.6) | 5 (1.4) | 1.17 | 0.35 to 3.87 | 0.796 |
| 73 | Road and Rail Drivers | 51 (13.7) | 26 (7.2) | 2.05 | 1.25 to 3.37 | 0.005** |
| 74 | Storepersons | 15 (4.0) | 6 (1.7) | 2.49 | 0.96 to 6.50 | 0.062 |
| 81 | Cleaners and Laundry Workers | 16 (4.3) | 12 (3.3) | 1.31 | 0.61 to 2.81 | 0.487 |
| 82 | Construction and Mining Labourers | 34 (9.1) | 14 (3.9) | 2.50 | 1.32 to 4.74 | 0.005** |
| 83 | Factory Process Workers | 40 (10.8) | 25 (6.9) | 1.62 | 0.96 to 2.74 | 0.069 |
| 84 | Farm, Forestry and Garden Workers | 33 (8.9) | 17 (4.7) | 1.98 | 1.08 to 3.61 | 0.027* |
| 89 | Other Labourers | 25 (6.7) | 12 (3.3) | 2.10 | 1.04 to 4.25 | 0.039* |

| ISCO Male Submajor Groups | | SALS *N* (%) | Controls *N* (%) | OR | 95% CI of OR | p value |
| --- | --- | --- | --- | --- | --- | --- |
| Code | Title | 372 (100) | 361 (100) |
| 11 | Chief Executives, senior officials and legislators | 7 (1.9) | 12 (3.3) | 0.56 | 0.22 to 1.43 | 0.225 |
| 12 | Administrative and commercial managers | 8 (2.2) | 15 (4.2) | 0.51 | 0.21 to 1.21 | 0.126 |
| 13 | Production and specialized services managers | 12 (3.2) | 19 (5.3) | 0.60 | 0.29 to 1.26 | 0.175 |
| 14 | Hospitality, retail and other services managers | 27 (7.3) | 17 (4.7) | 1.58 | 0.85 to 2.96 | 0.149 |
| 21 | Science and engineering professionals | 40 (10.8) | 54 (15.0) | 0.69 | 0.44 to 1.06 | 0.090 |
| 22 | Health professionals | 10 (2.7) | 14 (3.9) | 0.69 | 0.30 to 1.56 | 0.368 |
| 23 | Teaching professionals | 27 (7.3) | 26 (7.2) | 1.01 | 0.58 to 1.76 | 0.977 |
| 24 | Business and administration professionals | 15 (4.0) | 30 (8.3) | 0.46 | 0.25 to 0.88 | 0.018* |
| 25 | Information and communications technology professionals | 5 (1.3) | 17 (4.7) | 0.28 | 0.10 to 0.76 | 0.012* |
| 26 | Legal, social and cultural professionals | 13 (3.5) | 26 (7.2) | 0.47 | 0.24 to 0.92 | 0.029* |
| 31 | Science and engineering associate professionals | 19 (5.1) | 16 (4.4) | 1.16 | 0.59 to 2.29 | 0.668 |
| 32 | Health associate professionals | 9 (2.4) | 7 (1.9) | 1.25 | 0.46 to 3.40 | 0.657 |
| 33 | Business and administration associate professionals | 22 (5.9) | 25 (6.9) | 0.85 | 0.47 to 1.53 | 0.577 |
| 34 | Legal, social and cultural and related associate professionals | 11 (3.0) | 9 (2.5) | 1.19 | 0.49 to 2.91 | 0.700 |
| 35 | Information and communications technicians | 11 (3.0) | 9 (2.5) | 1.19 | 0.49 to 2.91 | 0.700 |
| 41 | General keyboard clerks | 18 (4.8) | 13 (3.6) | 1.36 | 0.66 to 2.82 | 0.407 |
| 42 | Customer services clerks | 11 (3.0) | 11 (3.0) | 0.97 | 0.42 to 2.27 | 0.943 |
| 43 | Numerical and material recording clerks | 10 (2.7) | 12 (3.3) | 0.80 | 0.34 to 1.88 | 0.615 |
| 51 | Personal service workers | 28 (7.5) | 9 (2.5) | 3.18 | 1.48 to 6.85 | 0.003** |
| 52 | Sales workers | 61 (16.4) | 59 (16.3) | 1.00 | 0.68 to 1.49 | 0.984 |
| 54 | Protective services workers | 11 (3.0) | 14 (3.9) | 0.76 | 0.34 to 1.69 | 0.493 |
| 61 | Market-oriented skilled agricultural workers | 36 (9.7) | 46 (12.7) | 0.73 | 0.46 to 1.17 | 0.189 |
| 71 | Building and related trades workers, excluding electricians | 63 (16.9) | 37 (10.2) | 1.79 | 1.16 to 2.76 | 0.009** |
| 72 | Metal, machinery and related trades workers | 68 (18.3) | 58 (16.1) | 1.17 | 0.80 to 1.72 | 0.427 |
| 73 | Handicraft and printing workers | 6 (1.6) | 10 (2.8) | 0.58 | 0.21 to 1.60 | 0.290 |
| 74 | Electrical and electronic trades workers | 27 (7.3) | 13 (3.6) | 2.10 | 1.06 to 4.13 | 0.033* |
| 75 | Food processing, wood working, garment and other craft and related trades workers | 32 (8.6) | 25 (6.9) | 1.27 | 0.73 to 2.18 | 0.398 |
| 81 | Stationary plant and machine operators | 53 (14.2) | 34 (9.4) | 1.60 | 1.01 to 2.53 | 0.045* |
| 83 | Drivers and mobile plant operators | 55 (14.8) | 32 (8.9) | 1.78 | 1.12 to 2.83 | 0.014* |
| 91 | Cleaners and helpers | 15 (4.0) | 10 (2.8) | 1.48 | 0.65 to 3.33 | 0.349 |
| 92 | Agricultural, forestry and fishery labourers | 38 (10.2) | 18 (5.0) | 2.17 | 1.21 to 3.88 | 0.009** |
| 93 | Labourers in mining, construction, manufacturing and transport | 48 (12.9) | 26 (7.2) | 1.91 | 1.16 to 3.15 | 0.011** |
| 96 | Refuse workers and other elementary workers | 19 (5.1) | 10 (2.8) | 1.89 | 0.87 to 4.12 | 0.110 |
| 83 | Factory Process Workers | 40 (10.8) | 25 (6.9) | 1.62 | 0.96 to 2.74 | 0.069 |
| 84 | Farm, Forestry and Garden Workers | 33 (8.9) | 17 (4.7) | 1.98 | 1.08 to 3.61 | 0.027* |
| 89 | Other Labourers | 25 (6.7) | 12 (3.3) | 2.10 | 1.04 to 4.25 | 0.039* |

Female Submajor Groups

| ANZSCO Female Submajor Groups | | SALS *N* (%) | Controls *N* (%) | OR | 95% CI of OR | p value |
| --- | --- | --- | --- | --- | --- | --- |
| Code | Title | 228 (100) | 389 (100) |
| 12 | Farmers and Farm Managers | 8 (3.5) | 15 (3.9) | 0.74 | 0.30 to 1.82 | 0.512 |
| 13 | Specialist Managers | 6 (2.6) | 16 (4.1) | 1.05 | 0.39 to 2.79 | 0.929 |
| 14 | Hospitality, Retail and Service Managers | 15 (6.6) | 18 (4.6) | 1.41 | 0.67 to 2.95 | 0.365 |
| 22 | Business, Human Resource and Marketing Professionals | 11 (4.8) | 13 (3.3) | 1.71 | 0.72 to 4.05 | 0.224 |
| 24 | Education Professionals | 24 (10.5) | 55 (14.1) | 0.77 | 0.46 to 1.30 | 0.331 |
| 25 | Health Professionals | 33 (14.5) | 62 (15.9) | 0.91 | 0.57 to 1.46 | 0.693 |
| 35 | Food Trades Workers | 8 (3.5) | 10 (2.6) | 1.95 | 0.73 to 5.21 | 0.185 |
| 39 | Other Technicians and Trades Workers | 22 (9.6) | 23 (5.9) | 1.48 | 0.79 to 2.80 | 0.224 |
| 42 | Carers and Aides | 20 (8.8) | 41 (10.5) | 0.99 | 0.55 to 1.76 | 0.959 |
| 43 | Hospitality Workers | 17 (7.5) | 36 (9.3) | 1.10 | 0.58 to 2.08 | 0.772 |
| 45 | Sports and Personal Service Workers | 8 (3.5) | 14 (3.6) | 1.32 | 0.53 to 3.32 | 0.550 |
| 52 | Personal Assistants and Secretaries | 43 (18.9) | 73 (18.8) | 1.04 | 0.67 to 1.60 | 0.869 |
| 53 | General Clerical Workers | 10 (4.4) | 17 (4.4) | 0.83 | 0.37 to 1.88 | 0.652 |
| 55 | Numerical Clerks | 17 (7.5) | 33 (8.5) | 0.91 | 0.49 to 1.72 | 0.778 |
| 56 | Clerical and Office Support Workers | 40 (17.5) | 65 (16.7) | 1.10 | 0.70 to 1.72 | 0.681 |
| 59 | Other Clerical and Administrative Workers | 6 (2.6) | 7 (1.8) | 1.94 | 0.59 to 6.36 | 0.273 |
| 62 | Sales Assistants and Salespersons | 48 (21.1) | 106 (27.2) | 0.73 | 0.49 to 1.08 | 0.116 |
| 63 | Sales Support Workers | 5 (2.2) | 12 (3.1) | 0.72 | 0.24 to 2.15 | 0.551 |
| 71 | Machine and Stationary Plant Operators | 12 (5.3) | 9 (2.3) | 1.70 | 0.70 to 4.14 | 0.245 |
| 81 | Cleaners and Laundry Workers | 51 (22.4) | 86 (22.1) | 0.92 | 0.61 to 1.39 | 0.924 |
| 83 | Factory Process Workers | 21 (9.2) | 24 (6.2) | 1.62 | 0.86 to 3.06 | 0.135 |
| 84 | Farm, Forestry and Garden Workers | 14 (6.1) | 14 (3.6) | 1.89 | 0.84 to 4.24 | 0.122 |
| 85 | Food Preparation Assistants | 6 (2.6) | 7 (1.8) | 2.51 | 0.78 to 8.03 | 0.122 |

| ISCO Female Submajor Groups | | SALS *N* (%) | Controls *N* (%) | OR | 95% CI of OR | p value |
| --- | --- | --- | --- | --- | --- | --- |
| Code | Title | 228 (100) | 390 (100) |
| 12 | Administrative and commercial managers | 6 (2.6) | 15 (3.8) | 1.17 | 0.43 to 3.15 | 0.760 |
| 14 | Hospitality, retail and other services managers | 16 (7.0) | 19 (4.9) | 1.43 | 0.68 to 2.98 | 0.345 |
| 22 | Health professionals | 19 (8.3) | 31 (7.9) | 1.27 | 0.68 to 2.38 | 0.457 |
| 23 | Teaching professionals | 25 (11.0) | 53 (13.6) | 0.84 | 0.5 to 1.41 | 0.510 |
| 24 | Business and administration professionals | 7 (3.1) | 8 (2.1) | 1.85 | 0.63 to 5.46 | 0.265 |
| 26 | Legal, social and cultural professionals | 8 (3.5) | 22 (5.6) | 0.65 | 0.27 to 1.53 | 0.322 |
| 32 | Health associate professionals | 22 (9.6) | 60 (15.4) | 0.63 | 0.37 to 1.07 | 0.086 |
| 33 | Business and administration associate professionals | 5 (2.2) | 11 (2.8) | 1.26 | 0.41 to 3.88 | 0.687 |
| 41 | General keyboard clerks | 78 (34.2) | 137 (35.1) | 0.96 | 0.67 to 1.38 | 0.842 |
| 42 | Customer services clerks | 12 (5.3) | 33 (8.5) | 0.69 | 0.34 to 1.40 | 0.304 |
| 43 | Numerical and material recording clerks | 8 (3.5) | 17 (4.4) | 0.76 | 0.31 to 1.86 | 0.553 |
| 51 | Personal service workers | 37 (16.2) | 64 (16.4) | 1.24 | 0.77 to 1.99 | 0.375 |
| 52 | Sales workers | 51 (22.4) | 112 (28.7) | 0.75 | 0.50 to 1.11 | 0.149 |
| 53 | Personal care workers | 18 (7.9) | 28 (7.2) | 1.37 | 0.72 to 2.59 | 0.341 |
| 61 | Market-oriented skilled agricultural workers | 11 (4.8) | 15 (3.8) | 1.20 | 0.54 to 2.67 | 0.663 |
| 75 | Food processing, wood working, garment and other craft and related trades workers | 13 (5.7) | 14 (3.6) | 1.26 | 0.57 to 2.81 | 0.572 |
| 81 | Stationary plant and machine operators | 24 (10.5) | 26 (6.7) | 1.53 | 0.84 to 2.79 | 0.168 |
| 91 | Cleaners and helpers | 49 (21.5) | 83 (21.3) | 0.94 | 0.62 to 1.42 | 0.751 |
| 92 | Agricultural, forestry and fishery labourers | 14 (6.1) | 15 (3.8) | 1.90 | 0.85 to 4.23 | 0.117 |
| 93 | Labourers in mining, construction, manufacturing and transport | 5 (2.2) | 8 (2.1) | 0.96 | 0.29 to 3.15 | 0.947 |
| 94 | Food preparation assistants | 7 (3.1) | 7 (1.8) | 2.98 | 0.97 to 9.13 | 0.056 |
| 96 | Refuse workers and other elementary workers | 5 (2.2) | 5 (1.3) | 1.88 | 0.50 to 7.02 | 0.350 |

3a Male Minor Groups

| ANZSCO Male Minor Groups | | SALS *N* (%) | Controls *N* (%) | OR | 95% CI of OR | p value |
| --- | --- | --- | --- | --- | --- | --- |
| Code | Title | 372 (100) | 362 (100) |
| 111 | Chief Executives, General Managers and Legislators | 5 (1.3) | 15 (4.1) | 0.32 | 0.11 to 0.88 | <0.001*** |
| 121 | Farmers and Farm Managers | 33 (8.9) | 40 (11) | 0.78 | 0.48 to 1.27 | 0.325 |
| 133 | Construction, Distribution and Production Managers | 19 (5.1) | 19 (5.2) | 0.97 | 0.51 to 1.87 | 0.931 |
| 142 | Retail Managers | 12 (3.2) | 10 (2.8) | 1.17 | 0.50 to 2.75 | 0.713 |
| 149 | Miscellaneous Hospitality, Retail and Service Managers | 5 (1.3) | 5 (1.4) | 0.97 | 0.28 to 3.39 | 0.965 |
| 211 | Arts Professionals | 9 (2.4) | 10 (2.8) | 0.87 | 0.35 to 2.17 | 0.770 |
| 221 | Accountants, Auditors and Company Secretaries | 5 (1.3) | 15 (4.1) | 0.32 | 0.11 to 0.88 | 0.027* |
| 222 | Financial Brokers and Dealers, and Investment Advisers | 5 (1.3) | 12 (3.3) | 0.40 | 0.14 to 1.14 | 0.086 |
| 224 | Information and Organisation Professionals | 5 (1.3) | 6 (1.7) | 0.81 | 0.25 to 2.67 | 0.727 |
| 231 | Air and Marine Transport Professionals | 9 (2.4) | 7 (1.9) | 1.26 | 0.46 to 3.41 | 0.653 |
| 232 | Architects, Designers, Planners and Surveyors | 8 (2.2) | 10 (2.8) | 0.77 | 0.30 to 1.98 | 0.593 |
| 233 | Engineering Professionals | 27 (7.3) | 33 (9.1) | 0.78 | 0.46 to 1.33 | 0.359 |
| 234 | Natural and Physical Science Professionals | 6 (1.6) | 12 (3.3) | 0.48 | 0.18 to 1.29 | 0.144 |
| 241 | School Teachers | 19 (5.1) | 13 (3.6) | 1.45 | 0.70 to 2.97 | 0.317 |
| 242 | Tertiary Education Professionals | 6 (1.6) | 10 (2.8) | 0.58 | 0.21 to 1.61 | 0.292 |
| 263 | ICT Network and Support Professionals | 6 (1.6) | 5 (1.4) | 1.17 | 0.35 to 3.87 | 0.796 |
| 312 | Building and Engineering Technicians | 12 (3.2) | 15 (4.1) | 0.77 | 0.36 to 1.67 | 0.510 |
| 313 | ICT and Telecommunications Technicians | 6 (1.6) | 6 (1.7) | 0.97 | 0.31 to 3.04 | 0.962 |
| 321 | Automotive Electricians and Mechanics | 17 (4.6) | 17 (4.7) | 0.97 | 0.49 to 1.93 | 0.935 |
| 322 | Fabrication Engineering Trades Workers | 23 (6.2) | 8 (2.2) | 2.92 | 1.29 to 6.61 | 0.010* |
| 323 | Mechanical Engineering Trades Workers | 16 (4.3) | 27 (7.5) | 0.56 | 0.30 to 1.05 | 0.072 |
| 331 | Bricklayers and Carpenters and Joiners | 23 (6.2) | 12 (3.3) | 1.92 | 0.94 to 3.92 | 0.073 |
| 332 | Floor Finishers and Painting Trades Workers | 11 (3.0) | 7 (1.9) | 1.55 | 0.59 to 4.03 | 0.374 |
| 334 | Plumbers | 11 (3.0) | 8 (2.2) | 1.35 | 0.54 to 3.39 | 0.525 |
| 341 | Electricians | 14 (3.8) | 7 (1.9) | 1.98 | 0.79 to 4.97 | 0.144 |
| 342 | Electronics and Telecommunications Trades Workers | 14 (3.8) | 8 (2.2) | 1.73 | 0.72 to 4.18 | 0.222 |
| 351 | Food Trades Workers | 19 (5.1) | 10 (2.8) | 1.90 | 0.87 to 4.13 | 0.108 |
| 362 | Horticultural Trades Workers | 9 (2.4) | 9 (2.5) | 0.97 | 0.38 to 2.49 | 0.953 |
| 392 | Printing Trades Workers | 6 (1.6) | 5 (1.4) | 1.17 | 0.35 to 3.87 | 0.796 |
| 394 | Wood Trades Workers | 6 (1.6) | 6 (1.7) | 0.97 | 0.31 to 3.04 | 0.962 |
| 411 | Health and Welfare Support Workers | 6 (1.6) | 5 (1.4) | 1.17 | 0.35 to 3.87 | 0.796 |
| 431 | Hospitality Workers | 18 (4.8) | 8 (2.2) | 2.25 | 0.97 to 5.24 | 0.060 |
| 441 | Defence Force Members, Fire Fighters and Police | 32 (8.6) | 33 (9.1) | 0.94 | 0.56 to 1.56 | 0.806 |
| 552 | Financial and Insurance Clerks | 15 (4.0) | 13 (3.6) | 1.13 | 0.53 to 2.41 | 0.755 |
| 561 | Clerical and Office Support Workers | 30 (8.1) | 21 (5.8) | 1.42 | 0.80 to 2.54 | 0.230 |
| 591 | Logistics Clerks | 5 (1.3) | 9 (2.5) | 0.53 | 0.18 to 1.61 | 0.265 |
| 611 | Insurance Agents and Sales Representatives | 12 (3.2) | 12 (3.3) | 0.97 | 0.43 to 2.19 | 0.946 |
| 612 | Real Estate Sales Agents | 5 (1.3) | 6 (1.7) | 0.81 | 0.25 to 2.67 | 0.727 |
| 621 | Sales Assistants and Salespersons | 50 (13.4) | 47 (13.0) | 1.04 | 0.68 to 1.60 | 0.855 |
| 711 | Machine Operators | 17 (4.6) | 11 (3.0) | 1.53 | 0.71 to 3.31 | 0.282 |
| 712 | Stationary Plant Operators | 20 (5.4) | 12 (3.3) | 1.66 | 0.80 to 3.44 | 0.176 |
| 721 | Mobile Plant Operators | 6 (1.6) | 5 (1.4) | 1.17 | 0.35 to 3.87 | 0.796 |
| 731 | Automobile, Bus and Rail Drivers | 22 (5.9) | 12 (3.3) | 1.83 | 0.89 to 3.76 | 0.098 |
| 733 | Truck Drivers | 31 (8.3) | 14 (3.9) | 2.26 | 1.18 to 4.32 | 0.014* |
| 741 | Storepersons | 15 (4.0) | 6 (1.7) | 2.49 | 0.96 to 6.50 | 0.062 |
| 811 | Cleaners and Laundry Workers | 16 (4.3) | 12 (3.3) | 1.31 | 0.61 to 2.81 | 0.487 |
| 821 | Construction and Mining Labourers | 34 (9.1) | 14 (3.9) | 2.50 | 1.32 to 4.74 | 0.005** |
| 830 | Factory Process Workers | 8 (2.2) | 8 (2.2) | 0.97 | 0.36 to 2.62 | 0.956 |
| 831 | Food Process Workers | 19 (5.1) | 6 (1.7) | 3.19 | 1.26 to 8.09 | 0.014* |
| 839 | Miscellaneous Factory Process Workers | 15 (4.0) | 10 (2.8) | 1.48 | 0.66 to 3.34 | 0.346 |
| 841 | Farm, Forestry and Garden Workers | 33 (8.9) | 17 (4.7) | 1.98 | 1.08 to 3.61 | 0.027* |
| 899 | Miscellaneous Labourers | 23 (6.2) | 10 (2.8) | 2.32 | 1.09 to 4.95 | 0.029* |

| ISCO Male Minor Groups | | SALS *N* (%) | Controls *N* (%) | OR | 95% CI of OR | p value |
| --- | --- | --- | --- | --- | --- | --- |
| Code | Title | 372 (100) | 361 (100) |
| 121 | Business services and administration managers | 5 (1.3) | 5 (1.4) | 0.98 | 0.28 to 3.40 | 0.969 |
| 132 | Manufacturing, mining, construction and distribution managers | 5 (1.3) | 9 (2.5) | 0.54 | 0.18 to 1.62 | 0.268 |
| 141 | Hotel and restaurant managers | 10 (2.7) | 4 (1.1) | 2.48 | 0.77 to 7.98 | 0.128 |
| 142 | Retail and wholesale trade managers | 12 (3.2) | 10 (2.8) | 1.18 | 0.50 to 2.76 | 0.708 |
| 214 | Engineering professionals (excluding electrotechnology) | 23 (6.2) | 29 (8.0) | 0.76 | 0.43 to 1.34 | 0.341 |
| 216 | Architects, planners, surveyors and designers | 9 (2.4) | 12 (3.3) | 0.73 | 0.30 to 1.74 | 0.473 |
| 241 | Finance professionals | 9 (2.4) | 20 (5.5) | 0.43 | 0.19 to 0.95 | 0.036* |
| 265 | Creative and performing artists | 7 (1.9) | 10 (2.8) | 0.68 | 0.26 to 1.80 | 0.434 |
| 311 | Physical and engineering science technicians | 9 (2.4) | 8 (2.2) | 1.10 | 0.42 to 2.88 | 0.846 |
| 332 | Sales and purchasing agents and brokers | 11 (3.0) | 13 (3.6) | 0.82 | 0.36 to 1.86 | 0.634 |
| 333 | Business services agents | 7 (1.9) | 7 (1.9) | 0.98 | 0.34 to 2.81 | 0.963 |
| 352 | Telecommunications and broadcasting technicians | 8 (2.2) | 7 (1.9) | 1.12 | 0.40 to 3.12 | 0.831 |
| 411 | General office clerks | 18 (4.8) | 12 (3.3) | 1.49 | 0.71 to 3.13 | 0.296 |
| 421 | Tellers, money collectors and related clerks | 11 (3.0) | 9 (2.5) | 1.20 | 0.49 to 2.93 | 0.691 |
| 431 | Numerical clerks | 7 (1.9) | 10 (2.8) | 0.68 | 0.26 to 1.80 | 0.434 |
| 513 | Waiters and bartenders | 13 (3.5) | 5 (1.4) | 2.59 | 0.92 to 7.35 | 0.073 |
| 522 | Shop salespersons | 50 (13.4) | 56 (15.5) | 0.85 | 0.56 to 1.29 | 0.444 |
| 541 | Protective services workers | 11 (3.0) | 14 (3.9) | 0.76 | 0.34 to 1.70 | 0.502 |
| 711 | Building frame and related trades workers | 35 (9.4) | 17 (4.7) | 2.11 | 1.16 to 3.85 | 0.014* |
| 712 | Building finishers and related trades workers | 19 (5.1) | 14 (3.9) | 1.34 | 0.66 to 2.72 | 0.414 |
| 713 | Painters, building structure cleaners and related trades workers | 12 (3.2) | 9 (2.5) | 1.31 | 0.55 to 3.15 | 0.545 |
| 721 | Sheet and structural metal workers, moulders and welders and related workers | 30 (8.1) | 26 (7.2) | 1.14 | 0.66 to 1.96 | 0.645 |
| 723 | Machinery mechanics and repairers | 37 (9.9) | 25 (6.9) | 1.49 | 0.88 to 2.54 | 0.138 |
| 732 | Printing trades workers | 5 (1.3) | 5 (1.4) | 0.98 | 0.28 to 3.40 | 0.969 |
| 741 | Electrical equipment installers and repairers | 20 (5.4) | 10 (2.8) | 2.01 | 0.93 to 4.35 | 0.078 |
| 751 | Food processing and related trades workers | 16 (4.3) | 10 (2.8) | 1.59 | 0.71 to 3.54 | 0.260 |
| 752 | Wood treaters, cabinet-makers and related trades workers | 6 (1.6) | 6 (1.7) | 0.98 | 0.31 to 3.05 | 0.966 |
| 811 | Mining and mineral processing plant operators | 14 (3.8) | 7 (1.9) | 1.99 | 0.79 to 4.99 | 0.143 |
| 816 | Food and related products machine operators | 13 (3.5) | 6 (1.7) | 2.16 | 0.81 to 5.73 | 0.124 |
| 817 | Wood processing and papermaking plant operators | 8 (2.2) | 10 (2.8) | 0.78 | 0.30 to 1.99 | 0.597 |
| 832 | Car, van and motorcycle drivers | 16 (4.3) | 8 (2.2) | 1.99 | 0.84 to 4.72 | 0.116 |
| 833 | Heavy truck and bus drivers | 29 (7.8) | 18 (5.0) | 1.99 | 1.11 to 3.58 | 0.022* |
| 921 | Agricultural, forestry and fishery labourers | 38 (10.2) | 18 (5.0) | 2.18 | 1.22 to 3.90 | 0.008** |
| 931 | Mining and construction labourers | 29 (7.8) | 12 (3.3) | 2.47 | 1.24 to 4.93 | 0.010* |
| 962 | Other elementary workers | 17 (4.6) | 9 (2.5) | 1.69 | 0.76 to 3.74 | 0.196 |

3b Female Minor Groups

| ANZSCO Female Minor Groups | | SALS *N* (%) | Controls *N* (%) | OR | 95% CI of OR | p value |
| --- | --- | --- | --- | --- | --- | --- |
| Code | Title | 228 (100) | 389 (100) |
| 121 | Farmers and Farm Managers | 5 (2.2) | 8 (2.1) | 0.74 | 0.30 to 1.82 | 0.740 |
| 141 | Accommodation and Hospitality Managers | 7 (3.1) | 12 (3.1) | 0.91 | 0.34 to 2.43 | 0.849 |
| 241 | School Teachers | 20 (8.8) | 47 (12.1) | 0.72 | 0.41 to 1.26 | 0.246 |
| 254 | Midwifery and Nursing Professionals | 28 (12.3) | 53 (13.6) | 0.90 | 0.54 to 1.49 | 0.670 |
| 351 | Food Trades Workers | 8 (3.5) | 10 (2.6) | 1.95 | 0.73 to 5.21 | 0.185 |
| 393 | Textile, Clothing and Footwear Trades Workers | 12 (5.3) | 10 (2.6) | 1.67 | 0.69 to 4.07 | 0.259 |
| 421 | Child Carers | 10 (4.4) | 13 (3.3) | 1.78 | 0.74 to 4.28 | 0.196 |
| 422 | Education Aides | 9 (3.9) | 7 (1.8) | 2.76 | 0.98 to 7.81 | 0.056 |
| 423 | Personal Carers and Assistants | 6 (2.6) | 23 (5.9) | 0.53 | 0.21 to 1.34 | 0.177 |
| 431 | Hospitality Workers | 17 (7.5) | 39 (10.0) | 1.10 | 0.58 to 2.08 | 0.772 |
| 451 | Personal Service and Travel Workers | 7 (3.1) | 10 (2.6) | 1.42 | 0.52 to 3.91 | 0.492 |
| 521 | Personal Assistants and Secretaries | 43 (18.9) | 74 (19.0) | 1.04 | 0.67 to 1.60 | 0.869 |
| 532 | Keyboard Operators | 10 (4.4) | 17 (4.4) | 0.83 | 0.37 to 1.88 | 0.652 |
| 551 | Accounting Clerks and Bookkeepers | 7 (3.1) | 14 (3.6) | 0.73 | 0.28 to 1.87 | 0.507 |
| 552 | Financial and Insurance Clerks | 10 (4.4) | 23 (5.9) | 0.89 | 0.40 to 1.98 | 0.771 |
| 561 | Clerical and Office Support Workers | 40 (17.5) | 66 (17) | 1.10 | 0.70 to 1.72 | 0.681 |
| 621 | Sales Assistants and Salespersons | 47 (20.6) | 108 (27.8) | 0.70 | 0.47 to 1.05 | 0.087 |
| 711 | Machine Operators | 12 (5.3) | 9 (2.3) | 1.70 | 0.70 to 4.14 | 0.245 |
| 811 | Cleaners and Laundry Workers | 51 (22.4) | 86 (22.1) | 0.92 | 0.61 to 1.39 | 0.706 |
| 841 | Farm, Forestry and Garden Workers | 14 (6.1) | 14 (3.6) | 1.89 | 0.84 to 4.24 | 0.122 |
| 851 | Food Preparation Assistants | 7 (3.1) | 7 (1.8) | 2.93 | 0.96 to 8.98 | 0.059 |

| ISCO Female Minor Groups | | SALS *N* (%) | Controls *N* (%) | OR | 95% CI of OR | p value |
| --- | --- | --- | --- | --- | --- | --- |
| Code | Title | 228 (100) | 390 (100) |
| 141 | Hotel and restaurant managers | 7 (3.1) | 9 (2.3) | 1.22 | 0.43 to 3.48 | 0.707 |
| 142 | Retail and wholesale trade managers | 8 (3.5) | 6 (1.5) | 2.79 | 0.89 to 8.79 | 0.080 |
| 222 | Nursing and midwifery professionals | 14 (6.1) | 21 (5.4) | 1.28 | 0.62 to 2.65 | 0.502 |
| 234 | Primary school and early childhood teachers | 8 (3.5) | 6 (1.5) | 2.21 | 0.73 to 6.67 | 0.161 |
| 322 | Nursing and midwifery associate professionals | 16 (7.0) | 42 (10.8) | 0.63 | 0.34 to 1.17 | 0.147 |
| 411 | General office clerks | 39 (17.1) | 59 (15.1) | 1.23 | 0.78 to 1.95 | 0.373 |
| 412 | Secretaries (general) | 40 (17.5) | 74 (19.0) | 0.89 | 0.58 to 1.39 | 0.621 |
| 413 | Keyboard operators | 10 (4.4) | 17 (4.4) | 0.83 | 0.37 to 1.88 | 0.654 |
| 421 | Tellers, money collectors and related clerks | 10 (4.4) | 21 (5.4) | 0.91 | 0.41 to 2.03 | 0.811 |
| 431 | Numerical clerks | 7 (3.1) | 16 (4.1) | 0.71 | 0.28 to 1.81 | 0.471 |
| 512 | Cooks | 7 (3.1) | 5 (1.3) | 3.65 | 1.09 to 12.28 | 0.036* |
| 513 | Waiters and bartenders | 15 (6.6) | 38 (9.7) | 0.93 | 0.48 to 1.80 | 0.831 |
| 514 | Hairdressers, beauticians and related workers | 7 (3.1) | 12 (3.1) | 1.19 | 0.43 to 3.30 | 0.736 |
| 522 | Shop salespersons | 44 (19.3) | 99 (25.4) | 0.72 | 0.47 to 1.09 | 0.117 |
| 524 | Other sales workers | 5 (2.2) | 9 (2.3) | 1.43 | 0.43 to 4.74 | 0.555 |
| 531 | Child care workers and teachers' aides | 16 (7.0) | 18 (4.6) | 1.89 | 0.92 to 3.88 | 0.084 |
| 753 | Garment and related trades workers | 9 (3.9) | 8 (2.1) | 1.57 | 0.57 to 4.28 | 0.384 |
| 815 | Textile, fur and leather products machine operators | 8 (3.5) | 6 (1.5) | 1.66 | 0.56 to 4.96 | 0.363 |
| 911 | Domestic, hotel and office cleaners and helpers | 42 (18.4) | 68 (17.4) | 0.92 | 0.59 to 1.43 | 0.915 |
| 921 | Agricultural, forestry and fishery labourers | 14 (6.1) | 15 (3.8) | 1.89 | 0.85 to 4.2 | 0.119 |
| 941 | Food preparation assistants | 7 (3.1) | 6 (1.5) | 4.12 | 1.22 to 13.93 | 0.023 |

4a Male Unit Groups

| ANZSCO Male Unit Groups | | SALS *N* (%) | Controls *N* (%) | OR | 95% CI of OR | p value |
| --- | --- | --- | --- | --- | --- | --- |
| Code | Title | 372 (100) | 362 (100) |
| 1213 | Livestock Farmers | 7 (1.9) | 6 (1.7) | 1.14 | 0.38 to 3.42 | 0.818 |
| 1331 | Construction Managers | 14 (3.8) | 8 (2.2) | 1.73 | 0.72 to 4.18 | 0.222 |
| 1421 | Retail Managers | 12 (3.2) | 10 (2.8) | 1.17 | 0.50 to 2.75 | 0.713 |
| 2211 | Accountants | 5 (1.3) | 15 (4.1) | 0.32 | 0.11 to 0.88 | 0.027* |
| 2312 | Marine Transport Professionals | 6 (1.6) | 6 (1.7) | 0.97 | 0.31 to 3.04 | 0.962 |
| 2332 | Civil Engineering Professionals | 7 (1.9) | 5 (1.4) | 1.37 | 0.43 to 4.35 | 0.594 |
| 2631 | Computer Network Professionals | 5 (1.3) | 5 (1.4) | 0.97 | 0.28 to 3.39 | 0.965 |
| 3132 | Telecommunications Technical Specialists | 5 (1.3) | 5 (1.4) | 0.97 | 0.28 to 3.39 | 0.965 |
| 3212 | Motor Mechanics | 16 (4.3) | 15 (4.1) | 1.04 | 0.51 to 2.14 | 0.916 |
| 3232 | Metal Fitters and Machinists | 14 (3.8) | 23 (6.4) | 0.58 | 0.29 to 1.14 | 0.113 |
| 3312 | Carpenters and Joiners | 18 (4.8) | 8 (2.2) | 2.25 | 0.97 to 5.24 | 0.060 |
| 3322 | Painting Trades Workers | 9 (2.4) | 6 (1.7) | 1.47 | 0.52 to 4.18 | 0.468 |
| 3411 | Electricians | 14 (3.8) | 7 (1.9) | 1.98 | 0.79 to 4.97 | 0.144 |
| 3341 | Plumbers | 11 (3.0) | 8 (2.2) | 1.35 | 0.54 to 3.39 | 0.525 |
| 3512 | Butchers and Smallgoods Makers | 9 (2.4) | 5 (1.4) | 1.77 | 0.59 to 5.33 | 0.310 |
| 3622 | Gardeners | 6 (1.6) | 5 (1.4) | 1.17 | 0.35 to 3.87 | 0.796 |
| 3941 | Cabinetmakers | 5 (1.3) | 5 (1.4) | 0.97 | 0.28 to 3.39 | 0.965 |
| 4411 | Defence Force Members, Fire Fighters and Police | 27 (7.3) | 26 (7.2) | 1.01 | 0.58 to 1.77 | 0.968 |
| 4413 | Police | 7 (1.9) | 5 (1.4) | 1.37 | 0.43 to 4.35 | 0.594 |
| 5521 | Bank Workers | 11 (3.0) | 9 (2.5) | 1.20 | 0.49 to 2.92 | 0.696 |
| 5612 | Couriers and Postal Deliverers | 8 (2.2) | 5 (1.4) | 1.57 | 0.51 to 4.84 | 0.433 |
| 5619 | Other Clerical and Office Support Workers | 18 (4.8) | 12 (3.3) | 1.48 | 0.70 to 3.13 | 0.300 |
| 5911 | Purchasing and Supply Logistics Clerks | 5 (1.3) | 6 (1.7) | 0.81 | 0.25 to 2.67 | 0.727 |
| 6113 | Sales Representatives | 9 (2.4) | 5 (1.4) | 1.77 | 0.59 to 5.33 | 0.310 |
| 6121 | Real Estate Sales Agents | 5 (1.3) | 6 (1.7) | 0.81 | 0.24 to 2.67 | 0.727 |
| 6213 | Motor Vehicle and Vehicle Parts Salespersons | 6 (1.6) | 5 (1.4) | 1.17 | 0.35 to 3.87 | 0.796 |
| 6219 | Other Sales Assistants and Salespersons | 36 (9.7) | 36 (9.9) | 0.97 | 0.60 to 1.58 | 0.903 |
| 7122 | Drillers, Miners and Shot Firers | 13 (3.5) | 6 (1.7) | 2.15 | 0.81 to 5.72 | 0.125 |
| 7311 | Automobile Drivers | 12 (3.2) | 6 (1.7) | 1.98 | 0.73 to 5.33 | 0.177 |
| 7331 | Truck Drivers | 31 (8.3) | 14 (3.9) | 2.26 | 1.18 to 4.32 | 0.014* |
| 7411 | Storepersons | 15 (4.0) | 6 (1.7) | 2.49 | 0.96 to 6.5 | 0.062 |
| 8216 | Other Cleaners | 8 (2.2) | 5 (1.4) | 1.57 | 0.51 to 4.84 | 0.433 |
| 8311 | Food and Drink Factory Workers | 11 (3.0) | 4 (1.1) | 2.73 | 0.86 to 8.65 | 0.088 |
| 8394 | Timber and Wood Process Workers | 8 (2.2) | 7 (1.9) | 1.12 | 0.40 to 3.11 | 0.836 |
| 8416 | Mixed Crop and Livestock Farm Workers | 19 (5.1) | 9 (2.5) | 2.11 | 0.94 to 4.73 | 0.069 |
| 8994 | Motor Vehicle Parts and Accessories Fitters | 9 (2.4) | 5 (1.4) | 1.77 | 0.59 to 5.33 | 0.310 |

| ISCO Male Unit Groups | | SALS *N* (%) | Controls *N* (%) | OR | 95% CI of OR | p value |
| --- | --- | --- | --- | --- | --- | --- |
| Code | Title | 372 (100) | 361 (100) |
| 1420 | Retail and wholesale trade managers | 12 (3.2) | 10 (2.8) | 1.17 | 0.50 to 2.74 | 0.718 |
| 2142 | Civil engineers | 7 (1.9) | 6 (1.7) | 1.14 | 0.38 to 3.41 | 0.822 |
| 2165 | Cartographers and surveyors | 7 (1.9) | 7 (1.9) | 0.97 | 0.34 to 2.79 | 0.955 |
| 2411 | Accountants | 5 (1.3) | 15 (4.2) | 0.31 | 0.11 to 0.87 | 0.027* |
| 3118 | Draughtspersons | 5 (1.3) | 5 (1.4) | 0.97 | 0.28 to 3.38 | 0.962 |
| 3334 | Real estate agents and property managers | 5 (1.3) | 6 (1.7) | 0.81 | 0.24 to 2.67 | 0.724 |
| 4110 | General office clerks | 18 (4.8) | 12 (3.3) | 1.48 | 0.70 to 3.12 | 0.304 |
| 4211 | Bank tellers and related clerks | 10 (2.7) | 9 (2.5) | 1.08 | 0.43 to 2.69 | 0.868 |
| 5223 | Shop sales assistants | 48 (12.9) | 51 (14.1) | 0.90 | 0.59 to 1.38 | 0.628 |
| 5412 | Police officers | 6 (1.6) | 6 (1.7) | 0.97 | 0.31 to 3.04 | 0.958 |
| 6121 | Livestock and dairy producers | 7 (1.9) | 6 (1.7) | 1.14 | 0.38 to 3.41 | 0.822 |
| 7111 | House builders | 10 (2.7) | 5 (1.4) | 1.97 | 0.67 to 5.81 | 0.221 |
| 7115 | Carpenters and joiners | 18 (4.8) | 8 (2.2) | 2.24 | 0.96 to 5.23 | 0.061 |
| 7126 | Plumbers and pipe fitters | 12 (3.2) | 8 (2.2) | 1.47 | 0.59 to 3.64 | 0.404 |
| 7131 | Painters and related workers | 9 (2.4) | 6 (1.7) | 1.47 | 0.52 to 4.16 | 0.472 |
| 7231 | Motor vehicle mechanics and repairers | 34 (9.1) | 24 (6.6) | 1.41 | 0.82 to 2.43 | 0.213 |
| 7411 | Building and related electricians | 11 (3.0) | 7 (1.9) | 1.54 | 0.59 to 4.02 | 0.377 |
| 7511 | Butchers, fishmongers and related food preparers | 12 (3.2) | 5 (1.4) | 2.37 | 0.83 to 6.81 | 0.108 |
| 7522 | Cabinet-makers and related workers | 5 (1.3) | 6 (1.7) | 0.81 | 0.24 to 2.67 | 0.724 |
| 8111 | Miners and quarriers | 13 (3.5) | 5 (1.4) | 2.58 | 0.91 to 7.31 | 0.075 |
| 8160 | Food and related products machine operators | 13 (3.5) | 6 (1.7) | 2.14 | 0.81 to 5.70 | 0.127 |
| 8172 | Wood processing plant operators | 8 (2.2) | 5 (1.4) | 1.57 | 0.51 to 4.83 | 0.578 |
| 8322 | Car, taxi and van drivers | 15 (4.0) | 7 (1.9) | 2.13 | 0.86 to 5.27 | 0.104 |
| 8332 | Heavy truck and lorry drivers | 31 (8.3) | 15 (4.2) | 2.10 | 1.11 to 3.95 | 0.022* |
| 9213 | Mixed crop and livestock farm labourers | 19 (5.1) | 9 (2.5) | 2.11 | 0.94 to 4.72 | 0.071 |
| 9214 | Garden and horticultural labourers | 9 (2.4) | 7 (1.9) | 1.25 | 0.46 to 3.40 | 0.657 |
| 9313 | Building construction labourers | 29 (7.8) | 9 (2.5) | 3.31 | 1.54 to 7.09 | 0.002** |
| 9621 | Messengers, package deliverers and luggage porters | 13 (3.5) | 7 (1.9) | 1.83 | 0.72 to 4.64 | 0.203 |

4b Female Unit Groups

| ANZSCO Female Unit Groups | | SALS *N* (%) | | Controls *N* (%) | | OR | | 95% CI of OR | | p value | |
| --- | --- | --- | --- | --- | --- | --- | --- | --- | --- | --- | --- |
| Code | Title | | 228 (100) | | 389 (100) | |  | |  | |  |
| 1411 | Café and Restaurant Managers | | 5 (2.2) | | 8 (2.1) | | 0.96 | | 0.30 to 3.13 | | 0.949 |
| 2544 | Registered Nurses | | 12 (5.3) | | 20 (5.1) | | 1.75 | | 0.45 to 6.76 | | 0.415 |
| 3514 | Cooks | | 7 (3.1) | | 5 (1.3) | | 3.64 | | 1.08 to 12.23 | | 0.037* |
| 3932 | Clothing Trades Workers | | 9 (3.9) | | 10 (2.6) | | 1.19 | | 0.46 to 3.06 | | 0.724 |
| 4211 | Child Carers | | 10 (4.4) | | 13 (3.3) | | 1.78 | | 0.74 to 0.43 | | 0.196 |
| 4221 | Education Aides | | 9 (3.9) | | 7 (1.8) | | 2.76 | | 0.98 to 7.81 | | 0.056 |
| 4315 | Waiters | | 9 (3.9) | | 17 (4.4) | | 1.19 | | 0.50 to 2.84 | | 0.697 |
| 5212 | Secretaries | | 42 (18.4) | | 71 (18.3) | | 1.03 | | 0.66 to 1.59 | | 0.912 |
| 5321 | Keyboard Operators | | 10 (4.4) | | 17 (4.4) | | 0.83 | | 0.37 to 1.88 | | 0.652 |
| 5521 | Bank Workers | | 10 (4.4) | | 21 (5.4) | | 0.91 | | 0.40 to 2.02 | | 0.807 |
| 5619 | Other Clerical and Office Support Workers | | 36 (15.8) | | 59 (15.2) | | 1.10 | | 0.69 to 1.76 | | 0.685 |
| 6219 | Other Sales Assistants and Salespersons | | 44 (19.3) | | 92 (23.7) | | 0.76 | | 0.50 to 1.15 | | 0.196 |
| 8112 | Commercial Cleaners | | 9 (3.9) | | 8 (2.1) | | 1.83 | | 0.68 to 4.96 | | 0.233 |
| 8113 | Domestic Cleaners | | 33 (14.5) | | 60 (15.4) | | 0.76 | | 0.47 to 1.24 | | 0.272 |
| 8416 | Mixed Crop and Livestock Farm Workers | | 6 (2.6) | | 7 (1.8) | | 1.16 | | 0.37 to 3.63 | | 0.803 |

| ISCO Female Unit Groups | | SALS *N* (%) | Controls *N* (%) | OR | 95% CI of OR | p value |
| --- | --- | --- | --- | --- | --- | --- |
| Code | Title | 228 (100) | 390 (100) |
| 1412 | Restaurant Managers | 5 (2.2) | 8 (2.1) | 1.04 | 0.32 to 3.38 | 0.947 |
| 1420 | Retail and Wholesale Trade Managers | 8 (3.5) | 6 (1.5) | 2.79 | 0.89 to 8.79 | 0.080 |
| 2221 | Nursing Professionals | 13 (5.7) | 20 (5.1) | 1.29 | 0.61 to 2.73 | 0.507 |
| 3221 | Nursing Associate Professionals | 16 (7.0) | 42 (10.8) | 0.63 | 0.34 to 1.17 | 0.147 |
| 4110 | General Office Clerks | 39 (17.1) | 59 (15.1) | 1.23 | 0.78 to 1.95 | 0.373 |
| 4120 | Secretaries (General) | 40 (17.5) | 74 (19) | 0.89 | 0.58 to 1.39 | 0.621 |
| 4131 | Typists and Word Processing Operators | 7 (3.1) | 15 (3.8) | 0.58 | 0.23 to 1.47 | 0.253 |
| 4211 | Bank Tellers and Related Clerks | 10 (4.4) | 21 (5.4) | 0.91 | 0.41 to 2.03 | 0.811 |
| 4311 | Accounting and bookkeeping clerks | 5 (2.2) | 15 (3.8) | 0.49 | 0.17 to 1.40 | 0.183 |
| 5120 | Cooks | 7 (3.1) | 5 (1.3) | 3.65 | 1.09 to 12.28 | 0.036* |
| 5131 | Waiters | 12 (5.3) | 29 (7.4) | 0.95 | 0.45 to 1.98 | 0.882 |
| 5223 | Shop Sales Assistants | 43 (18.9) | 96 (24.6) | 0.70 | 0.46 to 1.07 | 0.096 |
| 5311 | Child Care Workers | 11 (4.8) | 13 (3.3) | 1.97 | 0.84 to 4.63 | 0.120 |
| 5312 | Teachers Aides | 6 (2.6) | 6 (1.5) | 1.76 | 0.55 to 5.64 | 0.341 |
| 7531 | Tailors, Dressmakers , Furriers and Hatters | 7 (3.1) | 8 (2.1) | 1.02 | 0.35 to 2.92 | 0.976 |
| 9112 | Cleaners and Helpers in Offices, Hotels and Other Establishments | 10 (4.4) | 9 (2.3) | 1.85 | 0.72 to 4.75 | 0.199 |
| 9113 | Domestic Duties | 28 (12.3) | 57 (14.6) | 0.67 | 0.40 to 1.11 | 0.121 |
| 9213 | Mixed Crop and Livestock Farm Labourers | 6 (2.6) | 7 (1.8) | 1.16 | 0.64 to 3.63 | 0.802 |

5a Male Occupation Groups (ANZSCO only)

| ANZSCO Male Occupation Groups | | SALS *N* (%) | Controls *N* (%) | OR | 95% CI of OR | p value |
| --- | --- | --- | --- | --- | --- | --- |
| Code | Title | 372 (100) | 362 (100) |
| 133112 | Project Builder | 13 (3.5) | 6 (1.7) | 2.15 | 0.81 to 5.72 | 0.125 |
| 142111 | Retail Manager (General) | 11 (3.0) | 10 (2.8) | 1.07 | 0.45 to 2.56 | 0.874 |
| 233211 | Civil Engineer | 6 (1.6) | 5 (1.4) | 1.17 | 0.35 to 3.87 | 0.796 |
| 321211 | Motor Mechanic (General) | 16 (4.3) | 15 (4.1) | 1.04 | 0.51 to 2.14 | 0.916 |
| 323211 | Fitter (General) | 5 (1.3) | 9 (2.5) | 0.53 | 0.18 to 1.61 | 0.265 |
| 323212 | Fitter and Turner | 7 (1.9) | 12 (3.3) | 0.56 | 0.22 to 1.44 | 0.228 |
| 331212 | Carpenter | 15 (4.0) | 7 (1.9) | 2.13 | 0.86 to 5.29 | 0.103 |
| 332211 | Painting Trades Worker | 9 (2.4) | 6 (1.7) | 1.47 | 0.52 to 4.18 | 0.468 |
| 334111 | Plumber (General) | 6 (1.6) | 8 (2.2) | 0.73 | 0.25 to 2.11 | 0.556 |
| 341111 | Electrician (General) | 14 (3.8) | 7 (1.9) | 1.98 | 0.79 to 4.97 | 0.144 |
| 351211 | Butcher or Small Goods Maker | 9 (2.4) | 5 (1.4) | 1.77 | 0.59 to 5.33 | 0.310 |
| 394111 | Cabinetmaker | 5 (1.3) | 5 (1.4) | 0.97 | 0.28 to 3.39 | 0.965 |
| 441111 | Defence Force Member- Other Ranks | 27 (7.3) | 26 (7.2) | 1.01 | 0.58 to 1.77 | 0.968 |
| 441312 | Police Officer | 7 (1.9) | 5 (1.4) | 1.37 | 0.43 to 4.35 | 0.594 |
| 552111 | Bank Worker | 11 (3.0) | 9 (2.5) | 1.20 | 0.49 to 2.92 | 0.696 |
| 561999 | Clerical and Office Support Workers nec | 18 (4.8) | 12 (3.3) | 1.48 | 0.70 to 3.13 | 0.300 |
| 621999 | Sales Assistants and Salespersons nec | 36 (9.7) | 36 (9.9) | 0.97 | 0.60 to 1.58 | 0.903 |
| 712212 | Miner | 13 (3.5) | 5 (1.4) | 2.59 | 0.91 to 7.33 | 0.074 |
| 731112 | Taxi Driver | 10 (2.7) | 5 (1.4) | 1.97 | 0.67 to 5.83 | 0.219 |
| 733111 | Truck Driver (General) | 30 (8.1) | 13 (3.6) | 2.36 | 1.21 to 4.59 | 0.012* |
| 741111 | Storeperson | 15 (4.0) | 6 (1.7) | 2.49 | 0.96 to 6.50 | 0.062 |
| 821611 | Railway Track Worker | 8 (2.2) | 5 (1.4) | 1.57 | 0.51 to 4.84 | 0.433 |
| 839412 | Sawmill or Timber Yard Worker | 8 (2.2) | 5 (1.4) | 1.57 | 0.51 to 4.84 | 0.433 |
| 841611 | Mixed Crop and Livestock Farm Worker | 19 (5.1) | 9 (2.5) | 2.11 | 0.94 to 4.73 | 0.069 |

5 b Female Occupation Groups (ANZSCO only)

| ANZSCO Female Occupation Groups | | SALS *N* (%) | Controls *N* (%) | OR | 95% CI of OR | p value |
| --- | --- | --- | --- | --- | --- | --- |
| Code | Title | 372 (100) | 362 (100) |
| 141111 | Café or Restaurant Manager | 5 (2.2) | 8 (2.1) | 1.04 | 0.32 to 3.38 | 0.949 |
| 393213 | Dressmaker or Tailor | 8 (3.5) | 6 (1.5) | 1.56 | 0.52 to 4.67 | 0.430 |
| 422116 | Teachers Aide | 7 (3.1) | 6 (1.5) | 2.06 | 0.67 to 6.61 | 0.207 |
| 431511 | Waiter | 9 (3.9) | 17 (4.4) | 1.19 | 0.50 to 2.84 | 0.697 |
| 521211 | Secretary (General) | 42 (18.4) | 71 (18.3) | 1.03 | 0.66 to 1.59 | 0.912 |
| 552111 | Bank Worker | 10 (4.4) | 21 (5.4) | 0.91 | 0.40 to 2.02 | 0.807 |
| 561999 | Clerical and Office Support Workers nec | 36 (15.8) | 58 (14.9) | 1.12 | 0.70 to 1.8 | 0.631 |
| 621999 | Sales Assistants and Salespersons nec | 44 (19.3) | 91 (23.4) | 0.77 | 0.51 to 1.17 | 0.222 |
| 811211 | Commercial Cleaner | 9 (3.9) | 8 (2.1) | 1.83 | 0.68 to 4.96 | 0.233 |
| 811312 | Domestic Duties | 28 (12.3) | 57 (14.7) | 0.67 | 0.40 to 1.11 | 0.120 |
| 841611 | Mixed Crop and Livestock Farm Worker | 6 (2.6) | 7 (1.8) | 1.16 | 0.37 to 3.63 | 0.803 |
