# Supplementary material for: Different Occupations Associated with Amyotrophic Lateral Sclerosis: Is Diesel Exhaust the Link?
Source: PLoS One. 2013 Nov 11;8(11):e80993. doi: 10.1371/journal.pone.0080993 (PMC3823610; doi:10.1371/journal.pone.0080993)
Supplement: Classification S1 — ANZSCO occupational codes. (PDF) [file pone.0080993.s008.pdf]

Australian Bureau of Statistics

Statistics New Zealand
TATAURANGA AOTEAROA

Australian and New Zealand Standard Classification of Occupations

First Edition

ANZSCO

ABS cat. no. 1220.0

ANZSCO - Australian and New Zealand Standard Classification of Occupations

First Edition

Dennis Trewin
Australian Statistician
Australian Bureau of Statistics

Brian Pink
Government Statistician
Statistics New Zealand

AUSTRALIAN BUREAU OF STATISTICS / STATISTICS NEW ZEALAND

EMBARGO: 11.30AM (CANBERRA TIME) MON 11 SEP 2006

ABS Catalogue No. 1220.0

ISBN 0 642 23200 8

© Commonwealth of Australia 2006

This work is copyright. Apart from any use as permitted under the *Copyright Act 1968*, no part may be reproduced by any process without prior written permission from the Commonwealth. Requests and inquiries concerning reproduction and rights in this publication should be addressed to The Manager, Intermediary Management, Australian Bureau of Statistics, Locked Bag 10, Belconnen ACT 2616, by telephone (02) 6252 6998, fax (02) 6252 7102, or email: <intermediary.management@abs.gov.au>.
In all cases the ABS must be acknowledged as the source when reproducing or quoting any part of an ABS publication or other product.

© Crown Copyright New Zealand 2006

Statistics New Zealand gives no warranty that the information or data supplied contains no errors. However, all care and diligence has been used in processing, analysing and extracting the information. Statistics New Zealand shall not be liable for any loss or damage suffered by the customer consequent upon the use directly, or indirectly, of the information supplied in this product.

Any table or other material published in this report may be reproduced and published without further licence, provided that it does not purport to be published under government authority and that acknowledgement is made of this source.

Produced by the Australian Bureau of Statistics

INQUIRIES

- For further information about this classification, contact the ABS National Information and Referral Service on 1300 135 070 or Statistics NZ's Information Centre on 0508 525 525.

CONTENTS

page

CONTENTS

Preface v
Abbreviations vi

INTRODUCTION

Overview 1
Background to the development of ANZSCO 1
Purpose of this publication 2
Coding occupation information 2
Alternative views 2
Updates to ANZSCO 3

CONCEPTUAL BASIS OF ANZSCO

Scope of the classification 4
Overview of ANZSCO structure 4
Conceptual model 4
Underlying concepts 6
Design constraints 9

RELATIONSHIP TO OTHER OCCUPATION CLASSIFICATIONS

Comparison between ANZSCO, ASCO Second Edition and NZSCO 1999 11
Main differences between ANZSCO and ASCO Second Edition 11
Main differences between ANZSCO and NZSCO 1999 13
Comparability with ISCO 14
Correspondences 15

CLASSIFICATION STRUCTURE

Profile and summary of ANZSCO structure 17
Standard code scheme 18
Explanatory notes 20
Major Groups 21
Major and Sub-Major Groups 22
Major, Sub-Major and Minor Groups 23
Major, Sub-Major, Minor and Unit Groups 26
Major, Sub-Major, Minor, Unit Groups and Occupations 35

DEFINITIONS

Interpreting ANZSCO occupation definitions 68
Format of the definitions 68
Major Group 1 Managers 70
Major Group 2 Professionals 140
Major Group 3 Technicians and Trades Workers 335
Major Group 4 Community and Personal Service Workers 472
Major Group 5 Clerical and Administrative Workers 545
Major Group 6 Sales Workers 612

CONTENTS *continued*

page

DEFINITIONS *continued*

Major Group 7 Machinery Operators and Drivers 644
Major Group 8 Labourers 695

APPENDIXES

A Summary correspondence between ANZSCO and ASCO Second Edition and NZSCO 1999 778
B Australian Qualifications Framework 811
C New Zealand Register of Quality Assured Qualifications 813
D Employability skills 814

INDEX

Index of principal titles, alternative titles and specialisations 815

PREFACE

The Australian and New Zealand Standard Classification of Occupations (ANZSCO) has been developed for use in the collection, analysis and dissemination of occupation statistics in Australia and New Zealand.

ANZSCO was developed jointly by the Australian Bureau of Statistics (ABS), Statistics New Zealand (Statistics NZ) and the Australian Government Department of Employment and Workplace Relations (DEWR) to improve the comparability of occupation statistics between the two countries and the rest of the world.

The past decade has seen ongoing structural change in the Australian and New Zealand labour markets with strong employment growth in areas such as health, community services, business services, tourism and hospitality, and retail. Occupations have become more specialised and new occupations have emerged and evolved, particularly in information technology, communications and health services.

An up-to-date occupational classification is an essential response to the changing labour market. ANZSCO will assist the many enterprises, education and training bodies, government agencies, and industry and professional organisations to understand and adapt to emerging occupational requirements.

It is important that ANZSCO provides a means of comparing Australian and New Zealand occupation statistics with international statistics. The International Standard Classification for Occupations (ISCO-88), which is under review and will become ISCO-08, forms the basis for such comparisons. Correspondence tables between ANZSCO and ISCO-08 will be provided when ISCO-08 is available in 2008.

In the development of the classification many individuals, government and private organisations, professional associations, industry training bodies and unions were consulted. We gratefully acknowledge the assistance provided by many individuals and organisations.

In particular, we would like to thank the members of the Australian and New Zealand Reference Groups and the New Zealand Statistical Advisory Group who provided direction and guidance to the project team in developing ANZSCO.

We encourage use of the classification to improve the comparability and usefulness of occupation statistics across Australia and New Zealand.

Dennis Trewin
Australian Statistician
Australian Bureau of Statistics

Brian Pink
Government Statistician
Statistics New Zealand

September 2006

ABBREVIATIONS

**ABS**	Australian Bureau of Statistics
**ANZSCO**	Australian and New Zealand Standard Classification of Occupations
**AQF**	Australian Qualifications Framework
**ASCO**	Australian Standard Classification of Occupations
**Aus**	Australia
**DEST**	Australian Government Department of Education, Science and Training
**DEWR**	Australian Government Department of Employment and Workplace Relations
**ICT**	information and communication technology
**ILO**	International Labour Organization
**ISCO**	International Standard Classification of Occupations
**nec**	not elsewhere classified
**nfd**	not further defined
**NZ**	New Zealand
**NZ Register**	New Zealand Register of Quality Assured Qualifications
**NZSCO**	New Zealand Standard Classification of Occupations
**Statistics NZ**	Statistics New Zealand

INTRODUCTION

OVERVIEW

The Australian and New Zealand Standard Classification of Occupations (ANZSCO) is the product of a development program undertaken jointly by a project team from the Australian Bureau of Statistics (ABS), Statistics New Zealand (Statistics NZ) and the Australian Government Department of Employment and Workplace Relations (DEWR) for use in the collection, publication and analysis of occupation statistics.

ANZSCO provides a basis for the standardised collection, analysis and dissemination of occupation data for Australia and New Zealand. The use of ANZSCO will result in improved comparability of occupation statistics produced by the two countries.

ANZSCO replaces the Australian Standard Classification of Occupations (ASCO) Second Edition and the New Zealand Standard Classification of Occupations (NZSCO) 1999 used in Australia and New Zealand, respectively. ANZSCO is intended to provide an integrated framework for storing, organising and reporting occupation-related information in both statistical and client-oriented applications, such as matching job seekers to job vacancies and providing career information.

From 2006, ANZSCO will be used in ABS and Statistics NZ censuses and surveys where occupation data are collected. ANZSCO will also be progressively introduced into administrative by-product collections such as births, deaths, marriages and divorces.

This publication is designed to assist users who wish to understand the detailed structure and content of ANZSCO, and will aid in interpreting statistics classified to ANZSCO. A range of supporting materials, including detailed correspondence tables and alternative views of ANZSCO, will be released on the ABS and Statistics NZ web sites.

The introductory sections of this publication provide a detailed explanation of the conceptual basis of ANZSCO and describe some of the principal differences between ANZSCO and ASCO Second Edition and NZSCO 1999. The main part of this publication explains the code structure, details the classification structure, outlines the format of the ANZSCO occupation and group definitions and provides definitions for the 8 major groups, 43 sub-major groups, 97 minor groups, 358 unit groups and 998 occupations which comprise ANZSCO. A summary correspondence table (concordance) between ANZSCO, ASCO Second Edition and NZSCO 1999 is also included.

The number of occupations identified in ANZSCO is a net increase of 12 occupations compared to ASCO Second Edition and 433 occupations compared to NZSCO 1999.

BACKGROUND TO THE DEVELOPMENT OF ANZSCO

In support of the Australia-New Zealand Closer Economic Relations Trade Agreement, the ABS and Statistics NZ have a policy of working towards developing harmonised statistical classifications.

The benefits of developing a joint occupation classification were noted as being the ability to produce a more up-to-date, relevant and conceptually sound classification, and the improved capacity for analysis of trans-Tasman labour market data.

The development of ANZSCO commenced in 2002 as a joint project between the ABS, Statistics NZ and the Australian Government Department of Employment and Workplace Relations (DEWR).

BACKGROUND TO THE DEVELOPMENT OF ANZSCO *continued*

Formal consultations with stakeholders in Australia and New Zealand were undertaken between 2002 and 2005. These consultations informed stakeholders of progress and sought their views on a number of key issues affecting the overall design and structure of ANZSCO.

Early in the development of ANZSCO, it was necessary to agree on a common basis for harmonising the classification across the two countries recognising that some restructuring, expansion and contraction of the existing classifications would be necessary. For Australian users, a high level of comparability was maintained with ASCO Second Edition, by minimising the extent to which ASCO Second Edition unit groups were split and re-aggregated when designing ANZSCO unit groups.

However, for New Zealand users, moving from NZSCO 1999 to ANZSCO will involve structural change as well as a significant increase in the number of occupations. It is acknowledged that a consequence of this will be a time series break from NZSCO 1999 at all levels of the classification.

PURPOSE OF THIS PUBLICATION

This publication is a reference document intended to provide a detailed account of the content and structure of ANZSCO and to assist the interpretation of statistics classified to it. It is not intended as a means of assigning information about particular jobs to ANZSCO classes.

Care needs to taken when assigning information about particular jobs to ANZSCO classes because the same job titles can be used in different industries to describe different occupations (e.g. business analyst). Additionally, the titles used in ANZSCO are not an exhaustive list of all titles used by people to describe an occupation (e.g. brickie).

CODING OCCUPATION INFORMATION

To consistently and reliably allocate occupation information, such as responses from statistical collections, to any level of the ANZSCO structure, the minimum information required is occupation title and task. Restricted use can also be made of industry and employer information when it is available.

To enable easier and faster coding of occupation information, the ABS and Statistics NZ have developed their own Windows-based coding systems. These coding systems are based on an index (or codefile) of responses given in ABS and Statistics NZ collections and are rule-based to ensure that coding is performed in an accurate, consistent and efficient manner. Primary importance is given to the occupation title. Extensive use is also made of main tasks performed in the job.

Further information on the ABS and Statistics NZ coding systems can be obtained from the ABS National Information and Referral Service on 1300 135 070 or Statistics NZ's Information Centre on 0508 525 525.

ALTERNATIVE VIEWS

This publication provides the main classification structure of ANZSCO which should be used for output covering the whole labour force.

At times, it may be useful to look at alternative (or thematic) views of specific groups of occupations on the basis of the main goods and services produced or provided by an employee. Such alternative views span different parts of ANZSCO.

ALTERNATIVE VIEWS *continued*

Alternative views are seen to be a useful adjunct to the main ANZSCO structure. Therefore, a set of alternative views has been developed to facilitate meaningful and consistent comparison of employment in various 'industry' sectors between different data sources and across time.

At this stage, alternative views have been developed for agriculture, health, culture and leisure, hospitality and tourism, and information and communication technology (ICT).

The alternative views will be available from the ABS and Statistics NZ web sites.

UPDATES TO ANZSCO

An important consideration when developing a statistical classification is the need to build in sufficient robustness to allow for long-term usage. This robustness facilitates meaningful time series analysis of data assigned to that classification.

It is recognised that, for non-statistical uses of ANZSCO, there is a need for a classification which reflects the contemporary labour markets in Australia and New Zealand. To meet this need, minor updates to ANZSCO will be considered every two to three years.

To minimise disruption to time series data, updates will only be made at the occupation and possibly the unit group level. Updates will take the form of including newly emerging occupations and/or unit groups, merging declining occupations and/or unit groups with other occupations and/or unit groups, or changing titles of occupations and/or unit groups.

CONCEPTUAL BASIS OF ANZSCO

SCOPE OF THE CLASSIFICATION

The scope of ANZSCO is all occupations and jobs in the Australian and New Zealand labour markets undertaken for pay or profit, including jobs occupied by people working for themselves.

ANZSCO is not designed to cover work not undertaken for pay or profit, for example voluntary work. However, this does not preclude ANZSCO from describing such activities.

Occupations that are wholly illegal in New Zealand and all States and Territories of Australia are excluded from ANZSCO.

OVERVIEW OF ANZSCO STRUCTURE

The structure of ANZSCO has five hierarchical levels - major group, sub-major group, minor group, unit group and occupation. The categories at the most detailed level of the classification are termed 'occupations'. These are grouped together to form 'unit groups', which in turn are grouped into 'minor groups'. Minor groups are aggregated to form 'sub-major groups' which in turn are aggregated at the highest level to form 'major groups'.

These are the same hierarchical levels that are used in ASCO Second Edition and NZSCO 1999.

CONCEPTUAL MODEL

ANZSCO is a skill-based classification used to classify all occupations and jobs in the Australian and New Zealand labour markets.

To do this, ANZSCO identifies a set of occupations covering all jobs in the Australian and New Zealand labour markets, defines these occupations according to their attributes and groups them on the basis of their similarity into successively broader categories for statistical and other types of analysis. The individual objects classified in ANZSCO are jobs.

In ANZSCO, occupations are organised into progressively larger groups on the basis of their similarities in terms of both skill level and skill specialisation.

The conceptual model adopted for ANZSCO uses a combination of skill level and skill specialisation as criteria to design major groups which are meaningful and useful for most purposes. The eight major groups are formed by grouping together sub-major groups using aspects of both skill level and skill specialisation. In designing the major groups, intuitive appeal and usefulness in both statistical and administrative applications were also important considerations.

The skill level criterion is applied as rigorously as possible at the second level of the classification, the sub-major group level, together with a finer application of skill specialisation than that applied at the major group level. Each sub-major group is made up of a number of minor groups.

Minor groups are distinguished from each other mainly on the basis of a finer application of skill specialisation than that applied at the sub-major group level. Within minor groups, unit groups are distinguished from each other on the basis of skill specialisation and, where necessary, skill level.

CONCEPTUAL MODEL *continued*

Virtually all unit groups are at one skill level. There are only eight unit groups which contain occupations at more than one skill level. In all but two of these unit groups, the vast majority of jobs classified to the unit group are at one skill level only. Data stored at unit group level can therefore be aggregated by skill level with a high degree of validity.

Within unit groups, the distinction between occupations amounts to differences between tasks performed in occupations. All occupations are at one skill level.

As a result, data classified at the major group level will provide only a broad indication of skill level. Data at the sub-major group level will provide a satisfactory indication of skill level for many analytical purposes. Data classified at the unit group level will provide an accurate indication of skill level. Unit groups can, therefore, be aggregated by skill level to provide an indicative measure of occupations classified by skill level.

The distinctions between the various levels of ANZSCO are summarised in the following paragraphs.

MAJOR GROUP

Major groups are:

- the broadest level of ANZSCO
- formed using a combination of skill level and skill specialisation to create groups which are meaningful and useful for most (statistical and administrative) purposes.

SUB-MAJOR GROUP

Sub-major groups are:

- subdivisions of the major groups
- distinguished from other sub-major groups in the same major group on the basis of skill level and a broad application of skill specialisation.

MINOR GROUP

Minor groups are:

- subdivisions of the sub-major groups
- distinguished from other minor groups in the same sub-major group mainly on the basis of a less broad application of skill specialisation.

UNIT GROUP

Unit groups are:

- subdivisions of the minor groups
- distinguished from other unit groups in the same minor group on the basis of a finer application of skill specialisation and, where necessary, skill level.

OCCUPATION

Occupations are:

- the most detailed level of ANZSCO
- subdivisions of the unit groups
- distinguished from other occupations in the same unit group on the basis of detailed skill specialisation
- sets of jobs which involve the performance of a common set of tasks.

UNDERLYING CONCEPTS

As noted in Conceptual model, the framework adopted for ANZSCO is based on the concepts of 'job' and 'occupation'. The concept of 'occupation' is based on the concepts of 'skill level' and 'skill specialisation'.

Understanding these concepts is fundamental to understanding ANZSCO. These concepts are defined in the following sections.

The concept of job

A 'job' is defined as a set of tasks designed to be performed by one person for an employer (including self-employment) in return for payment or profit. Individual persons are classified by occupation through their relationship to a past, present or future job.

Any particular job will typically involve an individual working for a particular employer and undertaking a particular set of tasks. People working for themselves are considered as having a job and belonging to the labour force.

The concept of occupation

The categories at the most detailed level of the ANZSCO structure are called 'occupations'. An 'occupation' is defined as a set of jobs that require the performance of similar or identical sets of tasks. As it is rare for two actual jobs to have identical sets of tasks, in practical terms, an 'occupation' is a set of jobs whose main tasks are characterised by a high degree of similarity.

The similarity of tasks is defined in ANZSCO as a function of the level and specialisation of skill required to perform those tasks. Skill is defined as the ability to competently perform the tasks associated with an occupation.

It follows that ANZSCO classifies occupations according to two criteria - skill level and skill specialisation.

The concept of skill level

In ANZSCO, skill level is defined as a function of the range and complexity of the set of tasks performed in a particular occupation. The greater the range and complexity of the set of tasks, the greater the skill level of an occupation.

Skill level is measured operationally by:

- the level or amount of formal education and training
- the amount of previous experience in a related occupation, and
- the amount of on-the-job training

required to competently perform the set of tasks required for that occupation.

In general, the greater the range and complexity of the set of tasks involved, the greater the amount of formal education and training, previous experience and on-the-job training required to competently perform the set of tasks for that occupation.

Formal education and training refers to the level and amount of education and training required for competent performance of the tasks required in an occupation. It is measured in terms of educational qualifications as set out in the Australian Qualifications Framework (AQF) and the New Zealand Register of Quality Assured Qualifications (NZ Register). Details of the AQF and the NZ Register, as they were at the time of the development of ANZSCO, can be found at Appendixes B and C, respectively.

The concept of skill level continued

Previous experience refers to the time spent gaining work experience in related occupations or activities required for the competent performance of the tasks in an occupation. It is measured in months or years.

On-the-job training refers to the amount of training required after commencing work in an occupation for competent performance of the tasks in that occupation. It is measured in months or years, and may be undertaken at the same time as formal training.

ANZSCO does not measure the skill level of an individual, rather it refers to the level of skill that is typically required to competently perform the tasks of a particular occupation. Skill level is an attribute of occupations, not of individuals in the labour force or of particular jobs. It is irrelevant whether a particular individual working in a job in a particular occupation has a certain amount of training or a particular level of competence or not.

For example, a person who spreads mortar and lays bricks for a living has the occupation Bricklayer, regardless of whether he or she is an exceptionally competent bricklayer with many years of experience and post-trade qualifications, or an inexperienced bricklayer with no formal qualifications and a low level of competence. The skill level of the occupation Bricklayer is determined on the basis of that typically required for competent performance.

ANZSCO assigns occupations to one of five skill levels. In determining the skill level of each occupation in ANZSCO, advice was sought from employers, industry training bodies, professional organisations and others to ensure that the information is as accurate and meaningful as possible. The determination of boundaries between skill levels is based on the following definitions.

SKILL LEVEL 1

Occupations at Skill Level 1 have a level of skill commensurate with a bachelor degree or higher qualification. At least five years of relevant experience may substitute for the formal qualification. In some instances relevant experience and/or on-the-job training may be required in addition to the formal qualification.

SKILL LEVEL 2

Occupations at Skill Level 2 have a level of skill commensurate with one of the following:

- NZ Register Diploma or
- AQF Associate Degree, Advanced Diploma or Diploma.

At least three years of relevant experience may substitute for the formal qualifications listed above. In some instances relevant experience and/or on-the-job training may be required in addition to the formal qualification.

SKILL LEVEL 3

Occupations at Skill Level 3 have a level of skill commensurate with one of the following:

- NZ Register Level 4 qualification
- AQF Certificate IV or
- AQF Certificate III including at least two years of on-the-job training.

The concept of skill level continued

SKILL LEVEL 3 *continued*

At least three years of relevant experience may substitute for the formal qualifications listed above. In some instances relevant experience and/or on-the-job training may be required in addition to the formal qualification.

SKILL LEVEL 4

Occupations at Skill Level 4 have a level of skill commensurate with one of the following:

- NZ Register Level 2 or 3 qualification or
- AQF Certificate II or III.

At least one year of relevant experience may substitute for the formal qualifications listed above. In some instances relevant experience may be required in addition to the formal qualification.

SKILL LEVEL 5

Occupations at Skill Level 5 have a level of skill commensurate with one of the following:

- NZ Register Level 1 qualification
- AQF Certificate I or
- compulsory secondary education.

For some occupations a short period of on-the-job training may be required in addition to or instead of the formal qualification.

In some instances, no formal qualification or on-the-job training may be required.

The concept of skill specialisation

Skill specialisation is defined as a function of:

- field of knowledge required
- tools and equipment used
- materials worked on, and
- goods or services produced or provided.

Field of knowledge required refers to the subject matter knowledge that is essential for satisfactory performance of the tasks of an occupation.

Tools and equipment used includes all forms of plant, machinery, computer-based equipment or hand tools used in the performance of the tasks, as well as intellectual tools such as personal interaction, and art or design techniques.

- The term plant is used to describe mobile or stationary equipment which is large in size, performs several related functions, and is usually controlled by an internally located operator.
- The term machinery is used to describe stationary equipment which is not as large as plant, performs one processing function and is usually controlled by an externally located operator.
- The term hand tools is used to describe equipment which is small enough to be moved by one person.

Materials worked on refers to materials of both a tangible and abstract nature which are extracted, processed, transformed, refined or fabricated as an essential part of the tasks performed. Examples of materials worked on include wood, metal, livestock, accounting data, text, people and organisations.

The concept of skill specialisation continued

Goods or services produced or provided refers to the end product of the performance of the tasks of an occupation including physical goods, personal or other services, or abstract goods such as a software application or statistical information.

Employability skills

In developing the skill specialisation criteria for ANZSCO, employability skills were considered as a possible additional dimension of skill specialisation. There are two facets to employability skills, personal attributes such as loyalty, commitment and motivation, and generic skills, including communication, team work and problem-solving. Employers are increasingly using employability skills in conjunction with technical or job-specific skills when assessing the suitability of an individual for a particular occupation. Since these employability skills are applicable to most occupations, it was decided not to include them as classification criteria for ANZSCO.

Further discussion on employability skills can be found in Appendix D.

DESIGN CONSTRAINTS

One of the primary purposes of a statistical classification is to provide a simplification of the real world, which is a useful framework for collecting and analysing data from both statistical and administrative collections. To achieve this end, there are a number of practical considerations which impinged on the design of ANZSCO.

Statistical balance

As a general principle, a classification used for the dissemination of statistics should not have categories at the same level in its hierarchy which are too disparate in their population size. That is, similar numbers of real world entities should be classified to each category at a particular level. This approach serves to minimise large variations in standard errors and the suppression of cells in statistical tables at particular levels of the structure when using output from sample surveys. It also allows the classification to be used effectively for the cross-tabulation of aggregate data.

Categories which have been defined to reflect the real world, however, will not always be statistically balanced. To force categories to conform to size limitations would mean that the categories would not always be meaningful or useful.

In developing ANZSCO, a balance between these competing requirements was sought. The following minimum and maximum size guidelines were considered in designing the categories at each level of ANZSCO. For inclusion in ANZSCO, a category ideally fitted within the range listed below for either Australia or New Zealand.

	Australia	*New Zealand*
Major Group	500,000 to 1,500,000	100,000 to 300,000
Sub-Major Group	100,000 to 300,000	30,000 to 100,000
Minor Group	50,000 to 150,000	10,000 to 30,000
Unit Group	5,000 to 30,000	3,000 to 10,000
Occupation	300 to 10,000	100 to 5,000

Some exceptions, however, were made for occupations, or groups of occupations, of particular strategic or labour market significance.

Statistical feasibility

Another consideration affecting the design of ANZSCO was the statistical feasibility of the categories. That is, whether it is possible to accurately and consistently distinguish between the categories in the classification on the basis of responses to questions which can reasonably be asked in statistical collections such as population censuses and labour force surveys.

Time series comparability with ASCO Second Edition

Early in the development of ANZSCO, it was decided to try to maintain comparability, as far as practical, with ASCO Second Edition at the unit group level. This was achieved by minimising the extent to which ASCO Second Edition unit groups were split and re-aggregated when designing ANZSCO unit groups.

It was acknowledged that doing so would result in a major time series break from NZSCO 1999 for New Zealand users at all levels of the classification.

COMPARISON BETWEEN ANZSCO, ASCO SECOND EDITION AND NZSCO 1999

This section provides a broad comparison between ANZSCO and ASCO Second Edition and NZSCO 1999. The detailed relationship between ANZSCO, ASCO Second Edition and NZSCO 1999 will be explored in the correspondence tables (concordances) between ANZSCO and ASCO Second Edition, and ANZSCO and NZSCO 1999. See Correspondences.

Number of categories in classification structures

The following table indicates the number of categories at each level for the respective classifications.

Hierarchical Level	*ANZSCO*	*ASCO Second Edition*	*NZSCO 1999*
Major Group	8	9	9
Sub-Major Group	43	35	25
Minor Group	97	81	99
Unit Group	358	340	260
Occupation	998	986	565

Comparison between ANZSCO, ASCO Second Edition and NZSCO 1999 Major Groups

The following table compares the major group titles for ANZSCO, ASCO Second Edition and NZSCO 1999. Note that this table does not provide a correspondence between the three classifications.

ANZSCO	*ASCO Second Edition*	*NZSCO 1999*
1 Managers	1 Managers and Administrators	1 Legislators, Administrators and Managers
2 Professionals	2 Professionals	2 Professionals
3 Technicians and Trades Workers	3 Associate Professionals	3 Technicians and Associate Professionals
4 Community and Personal Service Workers	4 Tradespersons and Related Workers	4 Clerks
5 Clerical and Administrative Workers	5 Advanced Clerical and Service Workers	5 Service and Sales Workers
6 Sales Workers	6 Intermediate Clerical, Sales and Service Workers	6 Agriculture and Fishery Workers
7 Machinery Operators and Drivers	7 Intermediate Production and Transport Workers	7 Trades Workers
8 Labourers	8 Elementary Clerical, Sales and Service Workers	8 Plant and Machine Operators and Assemblers
	9 Labourers and Related Workers	9 Elementary Occupations

The full list of codes and titles for all groups in ASCO Second Edition can be found at <http://www.abs.gov.au>.

The full list of codes and titles for all groups in NZSCO 1999 can be found at <http://www.stats.govt.nz/statistical-methods/classifications/default.htm>.

MAIN DIFFERENCES BETWEEN ANZSCO AND ASCO SECOND EDITION

While the classification criteria for ANZSCO are essentially the same as those used for ASCO Second Edition, the application of the skill level criterion has changed. In ASCO Second Edition, the skill level required for entry to an occupation was considered. In

MAIN DIFFERENCES BETWEEN ANZSCO AND ASCO SECOND EDITION *continued*

ANZSCO, it is the skill level required for competent performance of the set of tasks associated with the occupation which is used.

The use of both skill level and skill specialisation to define the major groups in ANZSCO compared to just skill level in ASCO Second Edition has resulted in major groups which are more intuitively appealing and useful than their ASCO Second Edition predecessors. Accordingly, there have been changes to the way some occupations are classified in ANZSCO.

Associate Professionals

This ASCO Second Edition major group has been discontinued in ANZSCO. These occupations have been spread across a number of ANZSCO major groups. The major changes are:

Science, Engineering and Related Associate Professionals, ASCO Second Edition Sub-Major Group 31, are classified to ANZSCO Major Group 3 Technicians and Trades Workers, Sub-Major Group 31 Engineering, ICT and Science Technicians.

Finance Associate Professionals, ASCO Second Edition Minor Group 321, are classified to ANZSCO Major Group 2 Professionals, Minor Group 222 Financial Brokers and Dealers, and Investment Advisers.

Office Managers, ASCO Second Edition Unit Group 3291, and Project and Program Administrators, ASCO Second Edition Unit Group 3292, are classified to ANZSCO Major Group 5 Clerical and Administrative Workers, Sub-Major group 511 Office Managers and Program Administrators.

Managing Supervisors (Sales and Service), ASCO Second Edition Sub-Major Group 33, are mainly classified to ANZSCO Major Group 1 Managers, Sub-Major Group 14 Hospitality, Retail and Service Managers.

Health and Welfare Associate Professionals, ASCO Second Edition Sub-Major Group 34, are classified to ANZSCO Major Group 4 Community and Personal Service Workers, Sub-Major Group 41 Health and Welfare Support Workers.

Clerical, sales and service occupations

ASCO Second Edition Major Group 5 Advanced Clerical and Service Workers, Major Group 6 Intermediate Clerical, Sales and Service Workers, and Major Group 8 Elementary Clerical, Sales and Service Workers have been disaggregated and reorganised in ANZSCO into three intuitively appealing major groups.

There is now one major group which covers all clerical occupations - Major Group 5 Clerical and Administrative Workers, one major group which covers all sales occupations - Major Group 6 Sales Workers, and one major group which covers all service workers - Major Group 4 Community and Personal Service Workers.

Information and communication technology (ICT) occupations

ANZSCO identifies a greater number of ICT unit groups and occupations compared to ASCO Second Edition.

In ANZSCO, there are 13 ICT unit groups and 36 ICT occupations. This compares to three unit groups and nine occupations in ASCO Second Edition.

MAIN DIFFERENCES BETWEEN ANZSCO AND NZSCO 1999

While the classification criteria for ANZSCO are essentially the same as those used for NZSCO 1999, ANZSCO provides a greater definition of skill level, and the application of the skill level criterion is more rigorous compared to NZSCO 1999. The structure and conceptual basis of NZSCO 1999 was closer to that of the International Standard Classification of Occupations (ISCO) than ANZSCO.

The introduction of ANZSCO has resulted in there being more occupations than contained in NZSCO 1999. It has also meant major changes to the way some occupations were classified in NZSCO 1999. Some of the major changes are listed below.

Agriculture and Fishery Workers

This NZSCO 1999 major group has been discontinued in ANZSCO. These occupations have been spread across a number of ANZSCO major groups. The major changes are:

Farmers and farm managers are classified to ANZSCO Major Group 1 Managers, Sub-Major Group 12 Farmers and Farm Managers.

Farm and forestry workers are classified to ANZSCO Major Group 8 Labourers, Sub-Major Group 84 Farm, Forestry and Garden Workers.

Gardeners, greenkeepers and nurserypersons are classified to ANZSCO Major Group 3 Technicians and Trades Workers, Minor Group 362 Horticultural Trades Workers.

Gardening and nursery labourers are classified to ANZSCO Major Group 8 Labourers, Sub-Major Group 84 Farm, Forestry and Garden Workers.

Associate Professionals

This NZSCO 1999 major group has been discontinued in ANZSCO. These occupations have been spread across a number of ANZSCO major groups. The major changes are:

Physical Science and Engineering Associate Professionals, NZSCO 1999 Sub-Major Group 31, are mostly classified to ANZSCO Major Group 3 Technicians and Trades Workers, Sub-Major Group 31 Engineering, ICT and Science Technicians.

Ship and Aircraft Controllers and Technicians, NZSCO 1999 Minor Group 314, are classified to ANZSCO Major Group 2 Professionals, Minor Group 231 Air and Marine Transport Professionals.

Life Science Technicians and Related Workers, NZSCO 1999 Minor Group 321, are classified to ANZSCO Major Group 3 Technicians and Trades Workers, Sub-Major Group 31 Engineering, ICT and Science Technicians.

Health Associate Professionals, NZSCO 1999 Minor Group 322, and Nursing Associate Professionals, NZSCO 1999 Minor Group 323, are classified to ANZSCO Major Group 2 Professionals, Sub-Major Group 25 Health Professionals, and Major Group 4 Community and Personal Service Workers, Sub-Major Group 41 Health and Welfare Support Workers.

Associate Professionals continued

Writers, Artists, Entertainment and Sports Associate Professionals, NZSCO 1999 Minor Group 336, are classified to ANZSCO Major Group 2 Professionals, Sub-Major Group 21 Arts and Media Professionals, and Major Group 4 Community and Personal Service Workers, Sub-Major Group 45 Sports and Personal Service Workers.

Sales and service occupations

NZSCO 1999 Major Group 5 Service and Sales Workers has been disaggregated and reorganised in ANZSCO into two major groups.

There is now one major group which covers all sales occupations - Major Group 6 Sales Workers, and one major group which covers all service workers - Major Group 4 Community and Personal Service Workers.

Information and communication technology (ICT) occupations

ANZSCO identifies a greater number of ICT unit groups and occupations compared to NZSCO 1999.

In ANZSCO, there are 13 ICT unit groups and 36 ICT occupations. This compares to two minor groups, three unit groups and seven occupations in NZSCO 1999.

COMPARABILITY WITH ISCO

ISCO was developed by the International Labour Organization (ILO). ISCO was first issued in 1958 and revised versions were produced in 1968 and 1988. The main aims of ISCO are to provide a basis for international comparisons of occupation statistics between member countries and to provide a conceptual model for the development of national occupation classifications.

The current edition, ISCO-88, uses skill level and skill specialisation as criteria in the conceptual framework for the classification. Four broad skill levels are used, defined in terms of the educational categories and levels that appear in the International Standard Classification of Education 1976, published by the United Nations Educational, Scientific and Cultural Organization.

Comparison between ANZSCO and ISCO-88

The following table indicates the number of categories at each level of ANZSCO and ISCO-88.

Hierarchical Level	*ANZSCO*	*ISCO-88*
Major Group	8	10
Sub-Major Group	43	28
Minor Group	97	116
Unit Group	358	390
Occupation	998	–

The occupation level is not defined in ISCO-88, as it is expected that individual countries will develop this level of detail to suit their requirements.

The following table compares the major group titles for ANZSCO and ISCO-88. Note that this table does not provide a correspondence between the two classifications.

Comparison between ANZSCO and ISCO-88 continued

ANZSCO	*ISCO-88*
1 Managers	1 Legislators, Senior Officials and Managers
2 Professionals	2 Professionals
3 Technicians and Trades Workers	3 Technicians and Associate Professionals
4 Community and Personal Service Workers	4 Clerks
5 Clerical and Administrative Workers	5 Service Workers and Shop and Market Sales Workers
6 Sales Workers	6 Skilled Agricultural and Fishery Workers
7 Machinery Operators and Drivers	7 Craft and Related Trades Workers
8 Labourers	8 Plant and Machine Operators and Assemblers
	9 Elementary Occupations
	0 Armed Forces

The ten major groups in ISCO-88 are broadly similar to the eight ANZSCO major groups. The most significant differences at major group level are:

ISCO-88 identifies Skilled Agricultural and Fishery Workers as a separate major group (Major Group 6), whereas ANZSCO includes Farmers and Farm Managers as a sub-major group in Major Group 1 Managers; Skilled Animal and Horticultural Workers as a sub-major group in Major Group 3 Technicians and Trades Workers; and Farm, Forestry and Garden Workers as a sub-major group in Major Group 8 Labourers.

ISCO-88 Major Group 3 Technicians and Associate Professionals (and equivalent major groups in ASCO Second Edition and NZSCO 1999) has no equivalent in ANZSCO.

A major group of Community and Personal Service Workers has been introduced for ANZSCO and has no equivalent in ISCO-88.

In ISCO-88, jobs held by members of the armed forces are included in Major Group 0 Armed Forces. In ANZSCO, jobs held by members of the armed forces are classified with their civilian equivalents, where these exist, or to a number of defence force specific occupations.

ISCO-88 is currently being reviewed by the ILO.

CORRESPONDENCES

ANZSCO and ASCO Second Edition and NZSCO 1999

Detailed correspondence tables (concordances) have been developed between ANZSCO and ASCO Second Edition, and ANZSCO and NZSCO 1999. These correspondence tables show where one-to-one relationships exist between the occupations in ASCO Second Edition or NZSCO 1999 and ANZSCO, and where they do not.

The correspondence tables will be available in electronic format from the ABS and Statistics NZ web sites.

A summary correspondence table is included at Appendix A of this publication. This correspondence provides an overview of the relationship between ANZSCO and ASCO Second Edition and NZSCO 1999.

These correspondences are a conceptual link between ANZSCO and the classifications it replaces; ASCO Second Edition and NZSCO 1999.

ANZSCO and ASCO Second Edition and NZSCO 1999 continued

In Australia, a detailed link file will be produced following completion of data processing of the 2006 Census of Population and Housing showing the numerical/proportional relationship between the categories of ASCO Second Edition and ANZSCO. A number of other ABS collections, including the Labour Force Survey, will be dual-coded to both ASCO Second Edition and ANZSCO to assist in maintaining time series between the two classifications.

In New Zealand, the 2006 Census of Population and Dwellings was dual-coded to both NZSCO 1999 and ANZSCO. A number of other Statistics NZ collections, including the Household Labour Force Survey, will be dual-coded to both NZSCO 1999 and ANZSCO to assist in maintaining time series between the two classifications.

ANZSCO and ISCO

Detailed correspondences between ANZSCO and ISCO-88 will be available in electronic format from the ABS and Statistics NZ web sites from early 2007. The correspondence between ANZSCO and ISCO-08 will be developed once ISCO-08 is released.

PROFILE AND SUMMARY OF ANZSCO STRUCTURE

The structure of ANZSCO has five hierarchical levels - major group, sub-major group, minor group, unit group and occupation. The categories at the most detailed level of the classification are termed 'occupations'. These are grouped together to form 'unit groups', which in turn are grouped into 'minor groups'. Minor groups are aggregated to form 'sub-major groups' which in turn are aggregated at the highest level to form 'major groups'.

These are the same hierarchical levels that are used in ASCO Second Edition and NZSCO 1999.

The following is a profile of the ANZSCO structure. The complete listing of the major, sub-major, minor and unit groups and occupations follows later in this chapter.

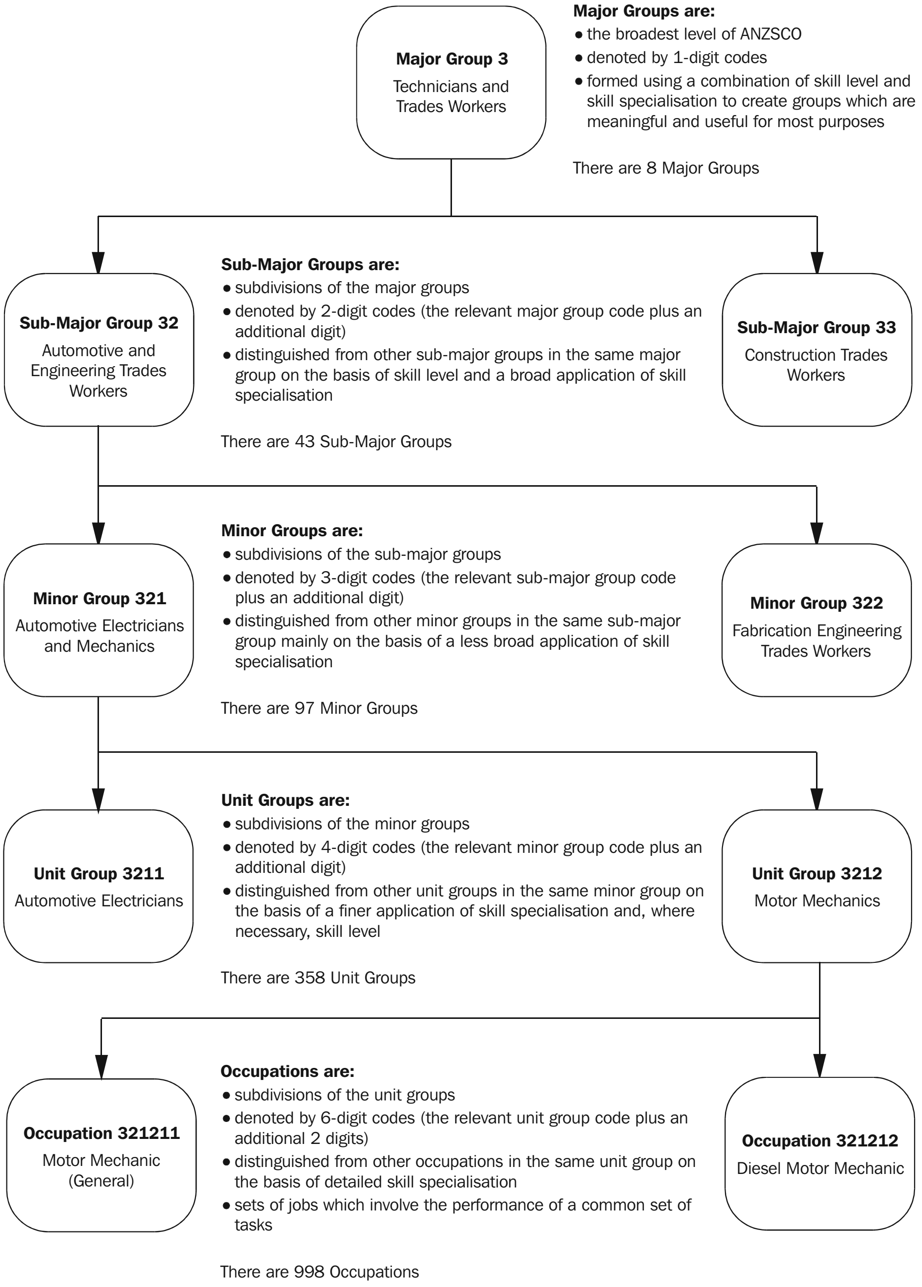

PROFILE AND SUMMARY OF ANZSCO STRUCTURE *continued*

Each major group comprises a different number of sub-major, minor and unit groups and occupations. The following table illustrates the distribution of these categories between the major groups.

Major Group	*Sub-Major Groups*	*Minor Groups*	*Unit Groups*	*Occupations*
1 Managers	4	11	38	95
2 Professionals	7	23	100	310
3 Technicians and Trades Workers	7	21	66	174
4 Community and Personal Service Workers	5	9	36	101
5 Clerical and Administrative Workers	7	12	33	80
6 Sales Workers	3	5	19	37
7 Machinery Operators and Drivers	4	7	22	76
8 Labourers	6	9	44	125

STANDARD CODE SCHEME

One, two, three, four and six-digit codes are assigned to the major, sub-major, minor and unit groups, and occupations respectively.

Within each major group, the sub-major groups are ordered firstly by skill level and then alphabetically. Residual 'other' sub-major groups are listed last. Sub-major groups comprising occupations at multiple skill levels have been ordered firstly on the basis of their highest predominant skill level, then alphabetically.

Within each sub-major group, the minor groups are ordered alphabetically, with the exception of residual 'miscellaneous' minor groups which are listed last. Similarly, within each minor group, the unit groups are ordered alphabetically, with the exception of the residual 'other' unit groups which are listed last.

The occupations within each unit group are essentially in alphabetical order, with the exception of 'general' occupations which are listed first, and residual 'not elsewhere classified' (nec) categories which are listed last. This ordering is more expedient than necessary and it is not considered that the addition of any new occupations, which may disrupt this ordering, will affect the usefulness of the classification.

The occupational profile of Australia and New Zealand is likely to change over time due to factors such as technological change and changes in the industrial profile of Australia and New Zealand. Therefore, from time to time, it may be necessary to add or delete occupations from the list of occupations separately identified in ANZSCO.

If it becomes necessary to identify an additional unit group or occupation, it will be allocated the next available four or six-digit code in the numerical sequence of codes of the minor or unit group to which it is being added. Similarly, if a unit group or occupation ceases to have sufficient numbers of persons employed to justify it continuing to be separately identified in the classification and it is consequently deleted from the classification, its code would not be reallocated as this would be likely to cause confusion with time series data.

It should be noted that the separately identified occupations are not allocated codes ending with the digits '0' or '9'. These are special purpose codes used to denote supplementary or operational (not further defined) codes in the case of '0' and residual (not elsewhere classified) categories in the case of '9' (see Supplementary or operational codes and Codes reserved for residual categories).

STANDARD CODE SCHEME *continued*

The ANZSCO code scheme is devised so that any future changes to the classification structure can be easily accommodated. However, in order that the classification remains a standard, users should not make arbitrary changes to the structure. Rather, they should contact the ABS or Statistics NZ and identify any apparent problems they encounter in the course of implementation, data collection or data analysis. ANZSCO will be revised at a suitable time so that all users continue to use the standard classification.

Codes reserved for residual categories

For each unit group of the classification structure, a six-digit code, consisting of the four digits of the unit group followed by the digits '99', is reserved as a residual 'not elsewhere classified' (nec) category. All occupations which are not separately identified in the classification structure are included in the 'nec' category of the unit group to which they relate. Residual categories are only identified in the classification structure if they are needed. ANZSCO currently identifies 77 'nec' categories.

The decision to include particular occupations in an 'nec' category rather than as substantive categories is based on their lack of numerical significance in Australia or New Zealand.

For each minor group, codes are reserved for residual categories at the unit group level. These codes consist of the minor group code followed by '9'. These categories are termed 'Other' and consist of separately identified occupations which do not fit into any of the unit groups contained within the minor group, on the basis of the classification criteria. The classification contains 21 'other' categories at the unit group level.

For each sub-major group, codes are also reserved for residual categories at the minor group level. These codes consist of the sub-major group code followed by '9'. These categories are termed 'Miscellaneous' and consist of separately identified unit groups which do not fit into any of the minor groups contained within the sub-major group, on the basis of the classification criteria. The classification contains eight 'miscellaneous' categories at the minor group level.

For each major group, codes are reserved for residual categories at the sub-major group level. These codes consist of the major group code followed by '9'. These categories are termed 'Other' and consist of separately identified minor groups which do not fit into any of the sub-major groups contained within the major group, on the basis of the classification criteria. The classification contains three 'other' categories at the sub-major group level.

It should be noted that residual categories are part of the ANZSCO structure. They should not be created or used merely to 'dump' responses that cannot be coded to any separately identified category in the classification because of insufficient detail in the response. See Supplementary or operational codes.

Supplementary or operational codes

Supplementary or operational codes are used in statistical collections to process inadequately described responses or for responses which are outside of the scope of the classification.

In Australia, these codes are of two types:

- six digit codes ending in two, three, four or five zeros; and
- six digit codes commencing with one zero.

Supplementary or operational codes continued

Codes ending in zero are described as 'not further defined' (nfd) codes and are used to code responses which cannot be coded to the occupation level of the classification, but which can be coded to a higher level of the classification structure.

For example, responses which cannot be identified as relating directly to a particular occupation category, but which are known to be within the range of occupations within a particular unit group are coded to that unit group. Such responses are allocated an nfd code consisting of the four-digit code of the unit group followed by '00'. For instance, the response 'Internal Medicine Specialist' does not contain sufficient information to be coded directly to any particular occupation category, but it can be coded to Unit Group 2533 Internal Medicine Specialist, which encompasses all internal medicine specialists. It is thus allocated the code 253300 Internal Medicine Specialists, nfd.

Codes commencing with zero are used to process responses which do not provide sufficient information to be coded to any level of the structure. They are also used to process responses such as 'housewife', 'pensioner' and 'student', which are not covered by the current definition of the labour force (see *Standards for Labour Force Statistics, ABS cat. no. 1288.0*). The standard set of such codes used in the ANZSCO Coding Index is available on request from the ABS.

Other codes commencing with zero may be defined by users to facilitate the processing and storage of data, when data sets coded to ANZSCO contain records for entities outside the scope of ANZSCO. For example, occupational activities which are wholly illegal in New Zealand and all States and Territories of Australia are excluded from ANZSCO.

In New Zealand, codes commencing with the digits '99' are used as supplementary or operational codes.

- The code '997000' is used for legitimate/valid responses, such as 'Public servant', which cannot be coded to any single occupation category because there is insufficient supporting information to accurately code to a specific category. This code is called 'Response Unidentifiable'.
- The code '999000' is used for responses, such as 'housewife', 'pensioner' or 'student', which are not covered by the current definition of the labour force. This code is called 'Response Outside Scope'.
- The code '999999' is used for non-response. This code is called 'Not Stated'.

It should be noted that supplementary or operational codes are not part of the classification structure. They exist for operational reasons only, and no data would be coded to them if sufficiently detailed responses or responses within the scope of the classification were obtained in all instances.

EXPLANATORY NOTES

The following tables detail the structure of ANZSCO at each, descending level of the classification. They also show the relationship between the groups and skill level.

The first three tables (Major Groups, Major and Sub-Major Groups, and Major, Sub-Major and Minor Groups) show the predominant skill levels applying to each group. Skill levels which apply to only a few occupations in each group are not shown.

EXPLANATORY NOTES
continued

For the two remaining tables (Major, Sub-Major, Minor and Unit Groups, and Major, Sub-Major, Minor and Unit Groups and Occupations) all skill levels applying to each group are shown.

A definitive list of all skill levels applying to each group in the classification is found in the definition for that group. See Definitions.

MAJOR GROUPS

		Predominant Skill Levels
1	Managers	1,2
2	Professionals	1
3	Technicians and Trades Workers	2,3
4	Community and Personal Service Workers	2,3,4,5
5	Clerical and Administrative Workers	2,3,4,5
6	Sales Workers	2,3,4,5
7	Machinery Operators and Drivers	4
8	Labourers	4,5

MAJOR AND SUB–MAJOR GROUPS

		Predominant Skill Level(s)
**1**	**MANAGERS**	
11	Chief Executives, General Managers and Legislators	1
12	Farmers and Farm Managers	1
13	Specialist Managers	1
14	Hospitality, Retail and Service Managers	2
**2**	**PROFESSIONALS**	
21	Arts and Media Professionals	1
22	Business, Human Resource and Marketing Professionals	1
23	Design, Engineering, Science and Transport Professionals	1
24	Education Professionals	1
25	Health Professionals	1
26	ICT Professionals	1
27	Legal, Social and Welfare Professionals	1
**3**	**TECHNICIANS AND TRADES WORKERS**	
31	Engineering, ICT and Science Technicians	2
32	Automotive and Engineering Trades Workers	3
33	Construction Trades Workers	3
34	Electrotechnology and Telecommunications Trades Workers	3
35	Food Trades Workers	2,3
36	Skilled Animal and Horticultural Workers	3
39	Other Technicians and Trades Workers	3
**4**	**COMMUNITY AND PERSONAL SERVICE WORKERS**	
41	Health and Welfare Support Workers	2
42	Carers and Aides	4
43	Hospitality Workers	4,5
44	Protective Service Workers	2,3,4,5
45	Sports and Personal Service Workers	3,4
**5**	**CLERICAL AND ADMINISTRATIVE WORKERS**	
51	Office Managers and Program Administrators	2
52	Personal Assistants and Secretaries	3
53	General Clerical Workers	4
54	Inquiry Clerks and Receptionists	4
55	Numerical Clerks	4
56	Clerical and Office Support Workers	5
59	Other Clerical and Administrative Workers	3,4
**6**	**SALES WORKERS**	
61	Sales Representatives and Agents	3,4
62	Sales Assistants and Salespersons	5
63	Sales Support Workers	5
**7**	**MACHINERY OPERATORS AND DRIVERS**	
71	Machine and Stationary Plant Operators	4
72	Mobile Plant Operators	4
73	Road and Rail Drivers	4
74	Storepersons	4
**8**	**LABOURERS**	
81	Cleaners and Laundry Workers	5
82	Construction and Mining Labourers	4,5
83	Factory Process Workers	4,5
84	Farm, Forestry and Garden Workers	5
85	Food Preparation Assistants	5
89	Other Labourers	5

MAJOR, SUB–MAJOR AND MINOR GROUPS

			Predominant Skill Level(s)
**1**		**MANAGERS**	
**11**		**Chief Executives, General Managers and Legislators**	
	111	Chief Executives, General Managers and Legislators	1
**12**		**Farmers and Farm Managers**	
	121	Farmers and Farm Managers	1
**13**		**Specialist Managers**	
	131	Advertising and Sales Managers	1
	132	Business Administration Managers	1
	133	Construction, Distribution and Production Managers	1
	134	Education, Health and Welfare Services Managers	1
	135	ICT Managers	1
	139	Miscellaneous Specialist Managers	1
**14**		**Hospitality, Retail and Service Managers**	
	141	Accommodation and Hospitality Managers	2
	142	Retail Managers	2
	149	Miscellaneous Hospitality, Retail and Service Managers	2
**2**		**PROFESSIONALS**	
**21**		**Arts and Media Professionals**	
	211	Arts Professionals	1
	212	Media Professionals	1
**22**		**Business, Human Resource and Marketing Professionals**	
	221	Accountants, Auditors and Company Secretaries	1
	222	Financial Brokers and Dealers, and Investment Advisers	1,2
	223	Human Resource and Training Professionals	1
	224	Information and Organisation Professionals	1
	225	Sales, Marketing and Public Relations Professionals	1
**23**		**Design, Engineering, Science and Transport Professionals**	
	231	Air and Marine Transport Professionals	1
	232	Architects, Designers, Planners and Surveyors	1
	233	Engineering Professionals	1
	234	Natural and Physical Science Professionals	1
**24**		**Education Professionals**	
	241	School Teachers	1
	242	Tertiary Education Teachers	1
	249	Miscellaneous Education Professionals	1
**25**		**Health Professionals**	
	251	Health Diagnostic and Promotion Professionals	1
	252	Health Therapy Professionals	1
	253	Medical Practitioners	1
	254	Midwifery and Nursing Professionals	1
**26**		**ICT Professionals**	
	261	Business and Systems Analysts, and Programmers	1
	262	Database and Systems Administrators, and ICT Security Specialists	1
	263	ICT Network and Support Professionals	1
**27**		**Legal, Social and Welfare Professionals**	
	271	Legal Professionals	1
	272	Social and Welfare Professionals	1
**3**		**TECHNICIANS AND TRADES WORKERS**	
**31**		**Engineering, ICT and Science Technicians**	
	311	Agricultural, Medical and Science Technicians	2
	312	Building and Engineering Technicians	2
	313	ICT and Telecommunications Technicians	2
**32**		**Automotive and Engineering Trades Workers**	
	321	Automotive Electricians and Mechanics	3
	322	Fabrication Engineering Trades Workers	3
	323	Mechanical Engineering Trades Workers	3
	324	Panelbeaters, and Vehicle Body Builders, Trimmers and Painters	3

MAJOR, SUB–MAJOR AND MINOR GROUPS *continued*

		Predominant Skill Level(s)
**3**	**TECHNICIANS AND TRADES WORKERS** *cont.*	
**33**	**Construction Trades Workers**	
331	Bricklayers, and Carpenters and Joiners	3
332	Floor Finishers and Painting Trades Workers	3
333	Glaziers, Plasterers and Tilers	3
334	Plumbers	3
**34**	**Electrotechnology and Telecommunications Trades Workers**	
341	Electricians	3
342	Electronics and Telecommunications Trades Workers	3
**35**	**Food Trades Workers**	
351	Food Trades Workers	3
**36**	**Skilled Animal and Horticultural Workers**	
361	Animal Attendants and Trainers, and Shearers	3
362	Horticultural Trades Workers	3
**39**	**Other Technicians and Trades Workers**	
391	Hairdressers	3
392	Printing Trades Workers	3
393	Textile, Clothing and Footwear Trades Workers	3
394	Wood Trades Workers	3
399	Miscellaneous Technicians and Trades Workers	3
**4**	**COMMUNITY AND PERSONAL SERVICE WORKERS**	
**41**	**Health and Welfare Support Workers**	
411	Health and Welfare Support Workers	2
**42**	**Carers and Aides**	
421	Child Carers	4
422	Education Aides	4
423	Personal Carers and Assistants	4
**43**	**Hospitality Workers**	
431	Hospitality Workers	4,5
**44**	**Protective Service Workers**	
441	Defence Force Members, Fire Fighters and Police	2,3
442	Prison and Security Officers	4,5
**45**	**Sports and Personal Service Workers**	
451	Personal Service and Travel Workers	4
452	Sports and Fitness Workers	3,4
**5**	**CLERICAL AND ADMINISTRATIVE WORKERS**	
**51**	**Office Managers and Program Administrators**	
511	Contract, Program and Project Administrators	2
512	Office and Practice Managers	2
**52**	**Personal Assistants and Secretaries**	
521	Personal Assistants and Secretaries	3
**53**	**General Clerical Workers**	
531	General Clerks	4
532	Keyboard Operators	4
**54**	**Inquiry Clerks and Receptionists**	
541	Call or Contact Centre Information Clerks	4
542	Receptionists	4
**55**	**Numerical Clerks**	
551	Accounting Clerks and Bookkeepers	4
552	Financial and Insurance Clerks	4
**56**	**Clerical and Office Support Workers**	
561	Clerical and Office Support Workers	5
**59**	**Other Clerical and Administrative Workers**	
591	Logistics Clerks	4
599	Miscellaneous Clerical and Administrative Workers	3,4

MAJOR, SUB–MAJOR AND MINOR GROUPS *continued*

		Predominant Skill Level(s)
**6**	**SALES WORKERS**	
**61**	**Sales Representatives and Agents**	
611	Insurance Agents and Sales Representatives	4
612	Real Estate Sales Agents	3
**62**	**Sales Assistants and Salespersons**	
621	Sales Assistants and Salespersons	5
**63**	**Sales Support Workers**	
631	Checkout Operators and Office Cashiers	5
639	Miscellaneous Sales Support Workers	5
**7**	**MACHINERY OPERATORS AND DRIVERS**	
**71**	**Machine and Stationary Plant Operators**	
711	Machine Operators	4
712	Stationary Plant Operators	4
**72**	**Mobile Plant Operators**	
721	Mobile Plant Operators	4
**73**	**Road and Rail Drivers**	
731	Automobile, Bus and Rail Drivers	4
732	Delivery Drivers	4
733	Truck Drivers	4
**74**	**Storepersons**	
741	Storepersons	4
**8**	**LABOURERS**	
**81**	**Cleaners and Laundry Workers**	
811	Cleaners and Laundry Workers	5
**82**	**Construction and Mining Labourers**	
821	Construction and Mining Labourers	4,5
**83**	**Factory Process Workers**	
831	Food Process Workers	4,5
832	Packers and Product Assemblers	5
839	Miscellaneous Factory Process Workers	4,5
**84**	**Farm, Forestry and Garden Workers**	
841	Farm, Forestry and Garden Workers	5
**85**	**Food Preparation Assistants**	
851	Food Preparation Assistants	5
**89**	**Other Labourers**	
891	Freight Handlers and Shelf Fillers	5
899	Miscellaneous Labourers	4,5

MAJOR, SUB–MAJOR, MINOR AND UNIT GROUPS

Code	Title	Skill Level(s)
1	**MANAGERS**	
11	**Chief Executives, General Managers and Legislators**	
111	Chief Executives, General Managers and Legislators	
1111	Chief Executives and Managing Directors	1
1112	General Managers	1
1113	Legislators	1
12	**Farmers and Farm Managers**	
121	Farmers and Farm Managers	
1211	Aquaculture Farmers	1
1212	Crop Farmers	1
1213	Livestock Farmers	1
1214	Mixed Crop and Livestock Farmers	1
13	**Specialist Managers**	
131	Advertising and Sales Managers	
1311	Advertising and Sales Managers	1
132	Business Administration Managers	
1321	Corporate Services Managers	1
1322	Finance Managers	1
1323	Human Resource Managers	1
1324	Policy and Planning Managers	1
1325	Research and Development Managers	1
133	Construction, Distribution and Production Managers	
1331	Construction Managers	1
1332	Engineering Managers	1
1333	Importers, Exporters and Wholesalers	1
1334	Manufacturers	1
1335	Production Managers	1
1336	Supply and Distribution Managers	1
134	Education, Health and Welfare Services Managers	
1341	Child Care Centre Managers	1
1342	Health and Welfare Services Managers	1
1343	School Principals	1
1344	Other Education Managers	1
135	ICT Managers	
1351	ICT Managers	1
139	Miscellaneous Specialist Managers	
1391	Commissioned Officers (Management)	1
1392	Senior Non-commissioned Defence Force Members	1
1399	Other Specialist Managers	1
14	**Hospitality, Retail and Service Managers**	
141	Accommodation and Hospitality Managers	
1411	Cafe and Restaurant Managers	2
1412	Caravan Park and Camping Ground Managers	2
1413	Hotel and Motel Managers	2
1414	Licensed Club Managers	2
1419	Other Accommodation and Hospitality Managers	2
142	Retail Managers	
1421	Retail Managers	2
149	Miscellaneous Hospitality, Retail and Service Managers	
1491	Amusement, Fitness and Sports Centre Managers	2
1492	Call or Contact Centre and Customer Service Managers	2
1493	Conference and Event Organisers	2
1494	Transport Services Managers	2
1499	Other Hospitality, Retail and Service Managers	2

MAJOR, SUB–MAJOR, MINOR AND UNIT GROUPS *continued*

		Skill Level(s)
**2**	**PROFESSIONALS**	
**21**	**Arts and Media Professionals**	
211	Arts Professionals	
2111	Actors, Dancers and Other Entertainers	1
2112	Music Professionals	1
2113	Photographers	1
2114	Visual Arts and Crafts Professionals	1
212	Media Professionals	
2121	Artistic Directors, and Media Producers and Presenters	1
2122	Authors, and Book and Script Editors	1
2123	Film, Television, Radio and Stage Directors	1
2124	Journalists and Other Writers	1
**22**	**Business, Human Resource and Marketing Professionals**	
221	Accountants, Auditors and Company Secretaries	
2211	Accountants	1
2212	Auditors, Company Secretaries and Corporate Treasurers	1
222	Financial Brokers and Dealers, and Investment Advisers	
2221	Financial Brokers	2
2222	Financial Dealers	1
2223	Financial Investment Advisers and Managers	1
223	Human Resource and Training Professionals	
2231	Human Resource Professionals	1
2232	ICT Trainers	1
2233	Training and Development Professionals	1
224	Information and Organisation Professionals	
2241	Actuaries, Mathematicians and Statisticians	1
2242	Archivists, Curators and Records Managers	1
2243	Economists	1
2244	Intelligence and Policy Analysts	1
2245	Land Economists and Valuers	1
2246	Librarians	1
2247	Management and Organisation Analysts	1
2249	Other Information and Organisation Professionals	1
225	Sales, Marketing and Public Relations Professionals	
2251	Advertising and Marketing Professionals	1
2252	ICT Sales Professionals	1
2253	Public Relations Professionals	1
2254	Technical Sales Representatives	1
**23**	**Design, Engineering, Science and Transport Professionals**	
231	Air and Marine Transport Professionals	
2311	Air Transport Professionals	1
2312	Marine Transport Professionals	1
232	Architects, Designers, Planners and Surveyors	
2321	Architects and Landscape Architects	1
2322	Cartographers and Surveyors	1
2323	Fashion, Industrial and Jewellery Designers	1
2324	Graphic and Web Designers, and Illustrators	1
2325	Interior Designers	1
2326	Urban and Regional Planners	1
233	Engineering Professionals	
2331	Chemical and Materials Engineers	1
2332	Civil Engineering Professionals	1
2333	Electrical Engineers	1
2334	Electronics Engineers	1
2335	Industrial, Mechanical and Production Engineers	1
2336	Mining Engineers	1
2339	Other Engineering Professionals	1

MAJOR, SUB–MAJOR, MINOR AND UNIT GROUPS *continued*

Code	Title	Skill Level(s)
**2**	**PROFESSIONALS** *cont.*	
**23**	**Design, Engineering, Science and Transport Professionals** *cont.*	
234	Natural and Physical Science Professionals	
2341	Agricultural and Forestry Scientists	1
2342	Chemists, and Food and Wine Scientists	1
2343	Environmental Scientists	1
2344	Geologists and Geophysicists	1
2345	Life Scientists	1
2346	Medical Laboratory Scientists	1
2347	Veterinarians	1
2349	Other Natural and Physical Science Professionals	1
**24**	**Education Professionals**	
241	School Teachers	
2411	Early Childhood (Pre-primary School) Teachers	1
2412	Primary School Teachers	1
2413	Middle School Teachers (Aus) / Intermediate School Teachers (NZ)	1
2414	Secondary School Teachers	1
2415	Special Education Teachers	1
242	Tertiary Education Teachers	
2421	University Lecturers and Tutors	1
2422	Vocational Education Teachers (Aus) / Polytechnic Teachers (NZ)	1
249	Miscellaneous Education Professionals	
2491	Education Advisers and Reviewers	1
2492	Private Tutors and Teachers	1
2493	Teachers of English to Speakers of Other Languages	1
**25**	**Health Professionals**	
251	Health Diagnostic and Promotion Professionals	
2511	Dietitians	1
2512	Medical Imaging Professionals	1
2513	Occupational and Environmental Health Professionals	1
2514	Optometrists and Orthoptists	1
2515	Pharmacists	1
2519	Other Health Diagnostic and Promotion Professionals	1
252	Health Therapy Professionals	
2521	Chiropractors and Osteopaths	1
2522	Complementary Health Therapists	1
2523	Dental Practitioners	1
2524	Occupational Therapists	1
2525	Physiotherapists	1
2526	Podiatrists	1
2527	Speech Professionals and Audiologists	1
253	Medical Practitioners	
2531	Generalist Medical Practitioners	1
2532	Anaesthetists	1
2533	Internal Medicine Specialists	1
2534	Psychiatrists	1
2535	Surgeons	1
2539	Other Medical Practitioners	1
254	Midwifery and Nursing Professionals	
2541	Midwives	1
2542	Nurse Educators and Researchers	1
2543	Nurse Managers	1
2544	Registered Nurses	1
**26**	**ICT Professionals**	
261	Business and Systems Analysts, and Programmers	
2611	ICT Business and Systems Analysts	1
2612	Multimedia Specialists and Web Developers	1
2613	Software and Applications Programmers	1
262	Database and Systems Administrators, and ICT Security Specialists	
2621	Database and Systems Administrators, and ICT Security Specialists	1

MAJOR, SUB–MAJOR, MINOR AND UNIT GROUPS *continued*

		Skill Level(s)
**2**	**PROFESSIONALS** *cont.*	
**26**	**ICT Professionals** *cont.*	
263	ICT Network and Support Professionals	
2631	Computer Network Professionals	1
2632	ICT Support and Test Engineers	1
2633	Telecommunications Engineering Professionals	1
**27**	**Legal, Social and Welfare Professionals**	
271	Legal Professionals	
2711	Barristers	1
2712	Judicial and Other Legal Professionals	1
2713	Solicitors	1
272	Social and Welfare Professionals	
2721	Counsellors	1
2722	Ministers of Religion	1
2723	Psychologists	1
2724	Social Professionals	1
2725	Social Workers	1
2726	Welfare, Recreation and Community Arts Workers	1
**3**	**TECHNICIANS AND TRADES WORKERS**	
**31**	**Engineering, ICT and Science Technicians**	
311	Agricultural, Medical and Science Technicians	
3111	Agricultural Technicians	2
3112	Medical Technicians	2
3113	Primary Products Inspectors	2
3114	Science Technicians	2
312	Building and Engineering Technicians	
3121	Architectural, Building and Surveying Technicians	2
3122	Civil Engineering Draftspersons and Technicians	2
3123	Electrical Engineering Draftspersons and Technicians	2
3124	Electronic Engineering Draftspersons and Technicians	2
3125	Mechanical Engineering Draftspersons and Technicians	2
3126	Safety Inspectors	2
3129	Other Building and Engineering Technicians	2
313	ICT and Telecommunications Technicians	
3131	ICT Support Technicians	2
3132	Telecommunications Technical Specialists	2
**32**	**Automotive and Engineering Trades Workers**	
321	Automotive Electricians and Mechanics	
3211	Automotive Electricians	3
3212	Motor Mechanics	3
322	Fabrication Engineering Trades Workers	
3221	Metal Casting, Forging and Finishing Trades Workers	3
3222	Sheetmetal Trades Workers	3
3223	Structural Steel and Welding Trades Workers	3
323	Mechanical Engineering Trades Workers	
3231	Aircraft Maintenance Engineers	3
3232	Metal Fitters and Machinists	3
3233	Precision Metal Trades Workers	3
3234	Toolmakers and Engineering Patternmakers	3
324	Panelbeaters, and Vehicle Body Builders, Trimmers and Painters	
3241	Panelbeaters	3
3242	Vehicle Body Builders and Trimmers	3
3243	Vehicle Painters	3
**33**	**Construction Trades Workers**	
331	Bricklayers, and Carpenters and Joiners	
3311	Bricklayers and Stonemasons	3
3312	Carpenters and Joiners	3

MAJOR, SUB–MAJOR, MINOR AND UNIT GROUPS *continued*

		Skill Level(s)
**3**	**TECHNICIANS AND TRADES WORKERS** *cont.*	
**33**	**Construction Trades Workers** *cont.*	
332	Floor Finishers and Painting Trades Workers	
3321	Floor Finishers	3
3322	Painting Trades Workers	3
333	Glaziers, Plasterers and Tilers	
3331	Glaziers	3
3332	Plasterers	3
3333	Roof Tilers	3
3334	Wall and Floor Tilers	3
334	Plumbers	
3341	Plumbers	3
**34**	**Electrotechnology and Telecommunications Trades Workers**	
341	Electricians	
3411	Electricians	3
342	Electronics and Telecommunications Trades Workers	
3421	Airconditioning and Refrigeration Mechanics	3
3422	Electrical Distribution Trades Workers	3
3423	Electronics Trades Workers	3
3424	Telecommunications Trades Workers	3
**35**	**Food Trades Workers**	
351	Food Trades Workers	
3511	Bakers and Pastrycooks	3
3512	Butchers and Smallgoods Makers	3
3513	Chefs	2
3514	Cooks	3
**36**	**Skilled Animal and Horticultural Workers**	
361	Animal Attendants and Trainers, and Shearers	
3611	Animal Attendants and Trainers	3
3612	Shearers	3
3613	Veterinary Nurses	3
362	Horticultural Trades Workers	
3621	Florists	3
3622	Gardeners	3
3623	Greenkeepers	3
3624	Nurserypersons	3
**39**	**Other Technicians and Trades Workers**	
391	Hairdressers	
3911	Hairdressers	3
392	Printing Trades Workers	
3921	Binders, Finishers and Screen Printers	3
3922	Graphic Pre-press Trades Workers	3
3923	Printers	3
393	Textile, Clothing and Footwear Trades Workers	
3931	Canvas and Leather Goods Makers	3
3932	Clothing Trades Workers	3
3933	Upholsterers	3
394	Wood Trades Workers	
3941	Cabinetmakers	3
3942	Wood Machinists and Other Wood Trades Workers	3
399	Miscellaneous Technicians and Trades Workers	
3991	Boat Builders and Shipwrights	3
3992	Chemical, Gas, Petroleum and Power Generation Plant Operators	3
3993	Gallery, Library and Museum Technicians	2
3994	Jewellers	3
3995	Performing Arts Technicians	3
3996	Signwriters	3
3999	Other Miscellaneous Technicians and Trades Workers	2,3

MAJOR, SUB–MAJOR, MINOR AND UNIT GROUPS *continued*

		Skill Level(s)
4	**COMMUNITY AND PERSONAL SERVICE WORKERS**	
41	**Health and Welfare Support Workers**	
411	Health and Welfare Support Workers	
4111	Ambulance Officers and Paramedics	2
4112	Dental Hygienists, Technicians and Therapists	2
4113	Diversional Therapists	2
4114	Enrolled and Mothercraft Nurses	2
4115	Indigenous Health Workers	2
4116	Massage Therapists	2
4117	Welfare Support Workers	2
42	**Carers and Aides**	
421	Child Carers	
4211	Child Carers	4
422	Education Aides	
4221	Education Aides	4
423	Personal Carers and Assistants	
4231	Aged and Disabled Carers	4
4232	Dental Assistants	4
4233	Nursing Support and Personal Care Workers	4
4234	Special Care Workers	4
43	**Hospitality Workers**	
431	Hospitality Workers	
4311	Bar Attendants and Baristas	4
4312	Cafe Workers	5
4313	Gaming Workers	4
4314	Hotel Service Managers	3
4315	Waiters	4
4319	Other Hospitality Workers	5
44	**Protective Service Workers**	
441	Defence Force Members, Fire Fighters and Police	
4411	Defence Force Members - Other Ranks	3
4412	Fire and Emergency Workers	3
4413	Police	2
442	Prison and Security Officers	
4421	Prison Officers	4
4422	Security Officers and Guards	3,4,5
45	**Sports and Personal Service Workers**	
451	Personal Service and Travel Workers	
4511	Beauty Therapists	4
4512	Driving Instructors	3
4513	Funeral Workers	2,3
4514	Gallery, Museum and Tour Guides	4
4515	Personal Care Consultants	4
4516	Tourism and Travel Advisers	4
4517	Travel Attendants	3
4518	Other Personal Service Workers	4,5
452	Sports and Fitness Workers	
4521	Fitness Instructors	4
4522	Outdoor Adventure Guides	4
4523	Sports Coaches, Instructors and Officials	2,3
4524	Sportspersons	3
5	**CLERICAL AND ADMINISTRATIVE WORKERS**	
51	**Office Managers and Program Administrators**	
511	Contract, Program and Project Administrators	
5111	Contract, Program and Project Administrators	2
512	Office and Practice Managers	
5121	Office Managers	2
5122	Practice Managers	2

MAJOR, SUB–MAJOR, MINOR AND UNIT GROUPS *continued*

Code			Title	Skill Level(s)
5			**CLERICAL AND ADMINISTRATIVE WORKERS** *cont.*	
52			**Personal Assistants and Secretaries**	
	521		Personal Assistants and Secretaries	
		5211	Personal Assistants	3
		5212	Secretaries	3
53			**General Clerical Workers**	
	531		General Clerks	
		5311	General Clerks	4
	532		Keyboard Operators	
		5321	Keyboard Operators	4
54			**Inquiry Clerks and Receptionists**	
	541		Call or Contact Centre Information Clerks	
		5411	Call or Contact Centre Workers	3,4
		5412	Inquiry Clerks	4
	542		Receptionists	
		5421	Receptionists	4
55			**Numerical Clerks**	
	551		Accounting Clerks and Bookkeepers	
		5511	Accounting Clerks	4
		5512	Bookkeepers	4
		5513	Payroll Clerks	4
	552		Financial and Insurance Clerks	
		5521	Bank Workers	4
		5522	Credit and Loans Officers	4
		5523	Insurance, Money Market and Statistical Clerks	4
56			**Clerical and Office Support Workers**	
	561		Clerical and Office Support Workers	
		5611	Betting Clerks	5
		5612	Couriers and Postal Deliverers	5
		5613	Filing and Registry Clerks	5
		5614	Mail Sorters	5
		5615	Survey Interviewers	5
		5616	Switchboard Operators	5
		5619	Other Clerical and Office Support Workers	5
59			**Other Clerical and Administrative Workers**	
	591		Logistics Clerks	
		5911	Purchasing and Supply Logistics Clerks	4
		5912	Transport and Despatch Clerks	4
	599		Miscellaneous Clerical and Administrative Workers	
		5991	Conveyancers and Legal Executives	2
		5992	Court and Legal Clerks	3
		5993	Debt Collectors	4
		5994	Human Resource Clerks	4
		5995	Inspectors and Regulatory Officers	4
		5996	Insurance Investigators, Loss Adjusters and Risk Surveyors	3
		5997	Library Assistants	4
		5999	Other Miscellaneous Clerical and Administrative Workers	4
6			**SALES WORKERS**	
61			**Sales Representatives and Agents**	
	611		Insurance Agents and Sales Representatives	
		6111	Auctioneers, and Stock and Station Agents	3
		6112	Insurance Agents	3
		6113	Sales Representatives	4
	612		Real Estate Sales Agents	
		6121	Real Estate Sales Agents	2,3

MAJOR, SUB–MAJOR, MINOR AND UNIT GROUPS *continued*

				Skill Level(s)
**6**			**SALES WORKERS** *cont.*	
**62**			**Sales Assistants and Salespersons**	
	621		Sales Assistants and Salespersons	
		6211	Sales Assistants (General)	5
		6212	ICT Sales Assistants	5
		6213	Motor Vehicle and Vehicle Parts Salespersons	4
		6214	Pharmacy Sales Assistants	5
		6215	Retail Supervisors	4
		6216	Service Station Attendants	5
		6217	Street Vendors and Related Salespersons	5
		6219	Other Sales Assistants and Salespersons	5
**63**			**Sales Support Workers**	
	631		Checkout Operators and Office Cashiers	
		6311	Checkout Operators and Office Cashiers	5
	639		Miscellaneous Sales Support Workers	
		6391	Models and Sales Demonstrators	5
		6392	Retail and Wool Buyers	3
		6393	Telemarketers	5
		6394	Ticket Salespersons	5
		6395	Visual Merchandisers	4
		6399	Other Sales Support Workers	5
**7**			**MACHINERY OPERATORS AND DRIVERS**	
**71**			**Machine and Stationary Plant Operators**	
	711		Machine Operators	
		7111	Clay, Concrete, Glass and Stone Processing Machine Operators	4
		7112	Industrial Spraypainters	4
		7113	Paper and Wood Processing Machine Operators	4
		7114	Photographic Developers and Printers	4
		7115	Plastics and Rubber Production Machine Operators	4
		7116	Sewing Machinists	4
		7117	Textile and Footwear Production Machine Operators	4
		7119	Other Machine Operators	4
	712		Stationary Plant Operators	
		7121	Crane, Hoist and Lift Operators	4
		7122	Drillers, Miners and Shot Firers	4
		7123	Engineering Production Systems Workers	4
		7129	Other Stationary Plant Operators	4
**72**			**Mobile Plant Operators**	
	721		Mobile Plant Operators	
		7211	Agricultural, Forestry and Horticultural Plant Operators	4
		7212	Earthmoving Plant Operators	4
		7213	Forklift Drivers	4
		7219	Other Mobile Plant Operators	4
**73**			**Road and Rail Drivers**	
	731		Automobile, Bus and Rail Drivers	
		7311	Automobile Drivers	4
		7312	Bus and Coach Drivers	4
		7313	Train and Tram Drivers	4
	732		Delivery Drivers	
		7321	Delivery Drivers	4
	733		Truck Drivers	
		7331	Truck Drivers	4
**74**			**Storepersons**	
	741		Storepersons	
		7411	Storepersons	4

MAJOR, SUB–MAJOR, MINOR AND UNIT GROUPS *continued*

Code	Title	Skill Level(s)
**8**	**LABOURERS**	
**81**	**Cleaners and Laundry Workers**	
811	Cleaners and Laundry Workers	
8111	Car Detailers	5
8112	Commercial Cleaners	5
8113	Domestic Cleaners	5
8114	Housekeepers	5
8115	Laundry Workers	5
8116	Other Cleaners	5
**82**	**Construction and Mining Labourers**	
821	Construction and Mining Labourers	
8211	Building and Plumbing Labourers	5
8212	Concreters	5
8213	Fencers	4
8214	Insulation and Home Improvement Installers	4
8215	Paving and Surfacing Labourers	5
8216	Railway Track Workers	4
8217	Structural Steel Construction Workers	4
8219	Other Construction and Mining Labourers	5
**83**	**Factory Process Workers**	
831	Food Process Workers	
8311	Food and Drink Factory Workers	5
8312	Meat Boners and Slicers, and Slaughterers	4
8313	Meat, Poultry and Seafood Process Workers	5
832	Packers and Product Assemblers	
8321	Packers	5
8322	Product Assemblers	5
839	Miscellaneous Factory Process Workers	
8391	Metal Engineering Process Workers	5
8392	Plastics and Rubber Factory Workers	5
8393	Product Quality Controllers	4
8394	Timber and Wood Process Workers	5
8399	Other Factory Process Workers	5
**84**	**Farm, Forestry and Garden Workers**	
841	Farm, Forestry and Garden Workers	
8411	Aquaculture Workers	5
8412	Crop Farm Workers	5
8413	Forestry and Logging Workers	4
8414	Garden and Nursery Labourers	5
8415	Livestock Farm Workers	5
8416	Mixed Crop and Livestock Farm Workers	5
8419	Other Farm, Forestry and Garden Workers	4,5
**85**	**Food Preparation Assistants**	
851	Food Preparation Assistants	
8511	Fast Food Cooks	5
8512	Food Trades Assistants	5
8513	Kitchenhands	5
**89**	**Other Labourers**	
891	Freight Handlers and Shelf Fillers	
8911	Freight and Furniture Handlers	5
8912	Shelf Fillers	5
899	Miscellaneous Labourers	
8991	Caretakers	5
8992	Deck and Fishing Hands	4
8993	Handypersons	5
8994	Motor Vehicle Parts and Accessories Fitters	4
8995	Printing Assistants and Table Workers	4
8996	Recycling and Rubbish Collectors	5
8997	Vending Machine Attendants	5
8999	Other Miscellaneous Labourers	5

MAJOR, SUB–MAJOR, MINOR, UNIT GROUPS AND OCCUPATIONS

Code	Title	Skill Level
1	**MANAGERS**	
11	**Chief Executives, General Managers and Legislators**	
111	**Chief Executives, General Managers and Legislators**	
1111	Chief Executives and Managing Directors	
111111	Chief Executive or Managing Director	1
1112	General Managers	
111211	Corporate General Manager	1
111212	Defence Force Senior Officer	1
1113	Legislators	
111311	Local Government Legislator	1
111312	Member of Parliament	1
111399	Legislators nec	1
12	**Farmers and Farm Managers**	
121	**Farmers and Farm Managers**	
1211	Aquaculture Farmers	
121111	Aquaculture Farmer	1
1212	Crop Farmers	
121211	Cotton Grower	1
121212	Flower Grower	1
121213	Fruit or Nut Grower	1
121214	Grain, Oilseed or Pasture Grower (Aus) / Field Crop Grower (NZ)	1
121215	Grape Grower	1
121216	Mixed Crop Farmer	1
121217	Sugar Cane Grower	1
121218	Turf Grower	1
121221	Vegetable Grower (Aus) / Market Gardener (NZ)	1
121299	Crop Farmers nec	1
1213	Livestock Farmers	
121311	Apiarist	1
121312	Beef Cattle Farmer	1
121313	Dairy Cattle Farmer	1
121314	Deer Farmer	1
121315	Goat Farmer	1
121316	Horse Breeder	1
121317	Mixed Livestock Farmer	1
121318	Pig Farmer	1
121321	Poultry Farmer	1
121322	Sheep Farmer	1
121399	Livestock Farmers nec	1
1214	Mixed Crop and Livestock Farmers	
121411	Mixed Crop and Livestock Farmer	1
13	**Specialist Managers**	
131	**Advertising and Sales Managers**	
1311	Advertising and Sales Managers	
131111	Advertising and Public Relations Manager	1
131112	Sales and Marketing Manager	1
132	**Business Administration Managers**	
1321	Corporate Services Managers	
132111	Corporate Services Manager	1
1322	Finance Managers	
132211	Finance Manager	1
1323	Human Resource Managers	
132311	Human Resource Manager	1
1324	Policy and Planning Managers	
132411	Policy and Planning Manager	1
1325	Research and Development Managers	
132511	Research and Development Manager	1

MAJOR, SUB–MAJOR, MINOR, UNIT GROUPS AND OCCUPATIONS *continued*

Code	Title	Skill Level
**1**	**MANAGERS** *cont.*	
**13**	**Specialist Managers** *cont.*	
**133**	**Construction, Distribution and Production Managers**	
1331	Construction Managers	
133111	Construction Project Manager	1
133112	Project Builder	1
1332	Engineering Managers	
133211	Engineering Manager	1
1333	Importers, Exporters and Wholesalers	
133311	Importer or Exporter	1
133312	Wholesaler	1
1334	Manufacturers	
133411	Manufacturer	1
1335	Production Managers	
133511	Production Manager (Forestry)	1
133512	Production Manager (Manufacturing)	1
133513	Production Manager (Mining)	1
1336	Supply and Distribution Managers	
133611	Supply and Distribution Manager	1
**134**	**Education, Health and Welfare Services Managers**	
1341	Child Care Centre Managers	
134111	Child Care Centre Manager	1
1342	Health and Welfare Services Managers	
134211	Medical Administrator (Aus) / Medical Superintendent (NZ)	1
134212	Nursing Clinical Director	1
134213	Primary Health Organisation Manager	1
134214	Welfare Centre Manager	1
134299	Health and Welfare Services Managers nec	1
1343	School Principals	
134311	School Principal	1
1344	Other Education Managers	
134411	Faculty Head	1
134412	Regional Education Manager	1
134499	Education Managers nec	1
**135**	**ICT Managers**	
1351	ICT Managers	
135111	Chief Information Officer	1
135112	ICT Project Manager	1
135199	ICT Managers nec	1
**139**	**Miscellaneous Specialist Managers**	
1391	Commissioned Officers (Management)	
139111	Commissioned Defence Force Officer	1
139112	Commissioned Fire Officer	1
139113	Commissioned Police Officer	1
1392	Senior Non-commissioned Defence Force Members	
139211	Senior Non-commissioned Defence Force Member	1
1399	Other Specialist Managers	
139911	Arts Administrator or Manager	1
139912	Environmental Manager	1
139913	Laboratory Manager	1
139914	Quality Assurance Manager	1
139915	Sports Administrator	1
139999	Specialist Managers nec	1

MAJOR, SUB–MAJOR, MINOR, UNIT GROUPS AND OCCUPATIONS *continued*

Code	Title	Skill Level
1	**MANAGERS** *cont.*	
14	**Hospitality, Retail and Service Managers**	
141	**Accommodation and Hospitality Managers**	
1411	Cafe and Restaurant Managers	
141111	Cafe or Restaurant Manager	2
1412	Caravan Park and Camping Ground Managers	
141211	Caravan Park and Camping Ground Manager	2
1413	Hotel and Motel Managers	
141311	Hotel or Motel Manager	2
1414	Licensed Club Managers	
141411	Licensed Club Manager	2
1419	Other Accommodation and Hospitality Managers	
141911	Bed and Breakfast Operator	2
141999	Accommodation and Hospitality Managers nec	2
142	**Retail Managers**	
1421	Retail Managers	
142111	Retail Manager (General)	2
142112	Antique Dealer	2
142113	Betting Agency Manager	2
142114	Hair or Beauty Salon Manager	2
142115	Post Office Manager	2
142116	Travel Agency Manager	2
149	**Miscellaneous Hospitality, Retail and Service Managers**	
1491	Amusement, Fitness and Sports Centre Managers	
149111	Amusement Centre Manager	2
149112	Fitness Centre Manager	2
149113	Sports Centre Manager	2
1492	Call or Contact Centre and Customer Service Managers	
149211	Call or Contact Centre Manager	2
149212	Customer Service Manager	2
1493	Conference and Event Organisers	
149311	Conference and Event Organiser	2
1494	Transport Services Managers	
149411	Fleet Manager	2
149412	Railway Station Manager	2
149413	Transport Company Manager	2
1499	Other Hospitality, Retail and Service Managers	
149911	Boarding Kennel or Cattery Operator	2
149912	Cinema or Theatre Manager	2
149913	Facilities Manager	2
149914	Financial Institution Branch Manager	2
149999	Hospitality, Retail and Service Managers nec	2

MAJOR, SUB–MAJOR, MINOR, UNIT GROUPS AND OCCUPATIONS *continued*

		Skill Level
**2**	**PROFESSIONALS**	
**21**	**Arts and Media Professionals**	
**211**	**Arts Professionals**	
2111	Actors, Dancers and Other Entertainers	
211111	Actor	1
211112	Dancer or Choreographer	1
211113	Entertainer or Variety Artist	1
211199	Actors, Dancers and Other Entertainers nec	1
2112	Music Professionals	
211211	Composer	1
211212	Music Director	1
211213	Musician (Instrumental)	1
211214	Singer	1
211299	Music Professionals nec	1
2113	Photographers	
211311	Photographer	1
2114	Visual Arts and Crafts Professionals	
211411	Painter (Visual Arts)	1
211412	Potter or Ceramic Artist	1
211413	Sculptor	1
211499	Visual Arts and Crafts Professionals nec	1
**212**	**Media Professionals**	
2121	Artistic Directors, and Media Producers and Presenters	
212111	Artistic Director	1
212112	Media Producer (excluding Video)	1
212113	Radio Presenter	1
212114	Television Presenter	1
2122	Authors, and Book and Script Editors	
212211	Author	1
212212	Book or Script Editor	1
2123	Film, Television, Radio and Stage Directors	
212311	Art Director (Film, Television or Stage)	1
212312	Director (Film, Television, Radio or Stage)	1
212313	Director of Photography	1
212314	Film and Video Editor	1
212315	Program Director (Television or Radio)	1
212316	Stage Manager	1
212317	Technical Director	1
212318	Video Producer	1
212399	Film, Television, Radio and Stage Directors nec	1
2124	Journalists and Other Writers	
212411	Copywriter	1
212412	Newspaper or Periodical Editor	1
212413	Print Journalist	1
212414	Radio Journalist	1
212415	Technical Writer	1
212416	Television Journalist	1
212499	Journalists and Other Writers nec	1
**22**	**Business, Human Resource and Marketing Professionals**	
**221**	**Accountants, Auditors and Company Secretaries**	
2211	Accountants	
221111	Accountant (General)	1
221112	Management Accountant	1
221113	Taxation Accountant	1
2212	Auditors, Company Secretaries and Corporate Treasurers	
221211	Company Secretary	1
221212	Corporate Treasurer	1
221213	External Auditor	1
221214	Internal Auditor	1

MAJOR, SUB–MAJOR, MINOR, UNIT GROUPS AND OCCUPATIONS *continued*

Code	Title	Skill Level
**2**	**PROFESSIONALS** *cont.*	
**22**	**Business, Human Resource and Marketing Professionals** *cont.*	
**222**	**Financial Brokers and Dealers, and Investment Advisers**	
2221	Financial Brokers	
222111	Commodities Trader	2
222112	Finance Broker	2
222113	Insurance Broker	2
222199	Financial Brokers nec	2
2222	Financial Dealers	
222211	Financial Market Dealer	1
222212	Futures Trader	1
222213	Stockbroking Dealer	1
222299	Financial Dealers nec	1
2223	Financial Investment Advisers and Managers	
222311	Financial Investment Adviser	1
222312	Financial Investment Manager	1
**223**	**Human Resource and Training Professionals**	
2231	Human Resource Professionals	
223111	Human Resource Adviser	1
223112	Recruitment Consultant	1
223113	Workplace Relations Adviser	1
2232	ICT Trainers	
223211	ICT Trainer	1
2233	Training and Development Professionals	
223311	Training and Development Professional	1
**224**	**Information and Organisation Professionals**	
2241	Actuaries, Mathematicians and Statisticians	
224111	Actuary	1
224112	Mathematician	1
224113	Statistician	1
2242	Archivists, Curators and Records Managers	
224211	Archivist	1
224212	Gallery or Museum Curator	1
224213	Health Information Manager	1
224214	Records Manager	1
2243	Economists	
224311	Economist	1
2244	Intelligence and Policy Analysts	
224411	Intelligence Officer	1
224412	Policy Analyst	1
2245	Land Economists and Valuers	
224511	Land Economist	1
224512	Valuer	1
2246	Librarians	
224611	Librarian	1
2247	Management and Organisation Analysts	
224711	Management Consultant	1
224712	Organisation and Methods Analyst	1
2249	Other Information and Organisation Professionals	
224911	Electorate Officer	1
224912	Liaison Officer	1
224913	Migration Agent (Aus) / Immigration Consultant (NZ)	1
224914	Patents Examiner	1
224999	Information and Organisation Professionals nec	1

MAJOR, SUB–MAJOR, MINOR, UNIT GROUPS AND OCCUPATIONS *continued*

Code	Title	Skill Level
**2**	**PROFESSIONALS** *cont.*	
**22**	**Business, Human Resource and Marketing Professionals** *cont.*	
**225**	**Sales, Marketing and Public Relations Professionals**	
2251	Advertising and Marketing Professionals	
225111	Advertising Specialist	1
225112	Market Research Analyst	1
225113	Marketing Specialist	1
2252	ICT Sales Professionals	
225211	ICT Account Manager	1
225212	ICT Business Development Manager	1
225213	ICT Sales Representative	1
2253	Public Relations Professionals	
225311	Public Relations Professional	1
2254	Technical Sales Representatives	
225411	Sales Representative (Industrial Products)	1
225412	Sales Representative (Medical and Pharmaceutical Products)	1
225499	Technical Sales Representatives nec	1
**23**	**Design, Engineering, Science and Transport Professionals**	
**231**	**Air and Marine Transport Professionals**	
2311	Air Transport Professionals	
231111	Aeroplane Pilot	1
231112	Air Traffic Controller	1
231113	Flying Instructor	1
231114	Helicopter Pilot	1
231199	Air Transport Professionals nec	1
2312	Marine Transport Professionals	
231211	Master Fisher	1
231212	Ship's Engineer	1
231213	Ship's Master	1
231214	Ship's Officer	1
231215	Ship's Surveyor	1
231299	Marine Transport Professionals nec	1
**232**	**Architects, Designers, Planners and Surveyors**	
2321	Architects and Landscape Architects	
232111	Architect	1
232112	Landscape Architect	1
2322	Cartographers and Surveyors	
232211	Cartographer	1
232212	Surveyor	1
2323	Fashion, Industrial and Jewellery Designers	
232311	Fashion Designer	1
232312	Industrial Designer	1
232313	Jewellery Designer	1
2324	Graphic and Web Designers, and Illustrators	
232411	Graphic Designer	1
232412	Illustrator	1
232413	Multimedia Designer	1
232414	Web Designer	1
2325	Interior Designers	
232511	Interior Designer	1
2326	Urban and Regional Planners	
232611	Urban and Regional Planner	1
**233**	**Engineering Professionals**	
2331	Chemical and Materials Engineers	
233111	Chemical Engineer	1
233112	Materials Engineer	1

MAJOR, SUB–MAJOR, MINOR, UNIT GROUPS AND OCCUPATIONS *continued*

		Skill Level
**2**	**PROFESSIONALS** *cont.*	
**23**	**Design, Engineering, Science and Transport Professionals** *cont.*	
**233**	**Engineering Professionals** *cont.*	
2332	Civil Engineering Professionals	
233211	Civil Engineer	1
233212	Geotechnical Engineer	1
233213	Quantity Surveyor	1
233214	Structural Engineer	1
233215	Transport Engineer	1
2333	Electrical Engineers	
233311	Electrical Engineer	1
2334	Electronics Engineers	
233411	Electronics Engineers	1
2335	Industrial, Mechanical and Production Engineers	
233511	Industrial Engineer	1
233512	Mechanical Engineer	1
233513	Production or Plant Engineer	1
2336	Mining Engineers	
233611	Mining Engineer (excluding Petroleum)	1
233612	Petroleum Engineer	1
2339	Other Engineering Professionals	
233911	Aeronautical Engineer	1
233912	Agricultural Engineer	1
233913	Biomedical Engineer	1
233914	Engineering Technologist	1
233915	Environmental Engineer	1
233916	Naval Architect (Aus) / Marine Designer (NZ)	1
233999	Engineering Professionals nec	1
**234**	**Natural and Physical Science Professionals**	
2341	Agricultural and Forestry Scientists	
234111	Agricultural Consultant	1
234112	Agricultural Scientist	1
234113	Forester (Aus) / Forest Scientist (NZ)	1
2342	Chemists, and Food and Wine Scientists	
234211	Chemist	1
234212	Food Technologist	1
234213	Wine Maker	1
2343	Environmental Scientists	
234311	Conservation Officer	1
234312	Environmental Consultant	1
234313	Environmental Research Scientist	1
234314	Park Ranger	1
234399	Environmental Scientists nec	1
2344	Geologists and Geophysicists	
234411	Geologist	1
234412	Geophysicist	1
2345	Life Scientists	
234511	Life Scientist (General)	1
234512	Anatomist or Physiologist	1
234513	Biochemist	1
234514	Biotechnologist	1
234515	Botanist	1
234516	Marine Biologist	1
234517	Microbiologist	1
234518	Zoologist	1
234599	Life Scientists nec	1
2346	Medical Laboratory Scientists	
234611	Medical Laboratory Scientist	1

MAJOR, SUB–MAJOR, MINOR, UNIT GROUPS AND OCCUPATIONS *continued*

Code	Title	Skill Level
**2**	**PROFESSIONALS** *cont.*	
**23**	**Design, Engineering, Science and Transport Professionals** *cont.*	
**234**	**Natural and Physical Science Professionals** *cont.*	
2347	Veterinarians	
234711	Veterinarian	1
2349	Other Natural and Physical Science Professionals	
234911	Conservator	1
234912	Metallurgist	1
234913	Meteorologist	1
234914	Physicist	1
234999	Natural and Physical Science Professionals nec	1
**24**	**Education Professionals**	
**241**	**School Teachers**	
2411	Early Childhood (Pre-primary School) Teachers	
241111	Early Childhood (Pre-primary School) Teacher	1
241112	Kaiako Kōhanga Reo (Māori Language Nest Teacher)	1
2412	Primary School Teachers	
241211	Kaiako Kura Kaupapa Māori (Māori-medium Primary School Teacher)	1
241212	Pouako Kura Kaupapa Māori (Māori-medium Primary School Senior Teacher)	1
241213	Primary School Teacher	1
2413	Middle School Teachers (Aus) / Intermediate School Teachers (NZ)	
241311	Middle School Teacher (Aus) / Intermediate School Teacher (NZ)	1
2414	Secondary School Teachers	
241411	Secondary School Teacher	1
2415	Special Education Teachers	
241511	Special Needs Teacher	1
241512	Teacher of the Hearing Impaired	1
241513	Teacher of the Sight Impaired	1
241599	Special Education Teachers nec	1
**242**	**Tertiary Education Teachers**	
2421	University Lecturers and Tutors	
242111	University Lecturer	1
242112	University Tutor	1
2422	Vocational Education Teachers (Aus) / Polytechnic Teachers (NZ)	
242211	Vocational Education Teacher (Aus) / Polytechnic Teacher (NZ)	1
**249**	**Miscellaneous Education Professionals**	
2491	Education Advisers and Reviewers	
249111	Education Adviser	1
249112	Education Reviewer	1
2492	Private Tutors and Teachers	
249211	Art Teacher (Private Tuition)	1
249212	Dance Teacher (Private Tuition)	1
249213	Drama Teacher (Private Tuition)	1
249214	Music Teacher (Private Tuition)	1
249299	Private Tutors and Teachers nec	1
2493	Teachers of English to Speakers of Other Languages	
249311	Teacher of English to Speakers of Other Languages	1
**25**	**Health Professionals**	
**251**	**Health Diagnostic and Promotion Professionals**	
2511	Dietitians	
251111	Dietitian	1
2512	Medical Imaging Professionals	
251211	Medical Diagnostic Radiographer	1
251212	Medical Radiation Therapist	1
251213	Nuclear Medicine Technologist	1
251214	Sonographer	1

MAJOR, SUB–MAJOR, MINOR, UNIT GROUPS AND OCCUPATIONS *continued*

		Skill Level
**2**	**PROFESSIONALS** *cont.*	
**25**	**Health Professionals** *cont.*	
**251**	**Health Diagnostic and Promotion Professionals** *cont.*	
2513	Occupational and Environmental Health Professionals	
251311	Environmental Health Officer	1
251312	Occupational Health and Safety Adviser	1
2514	Optometrists and Orthoptists	
251411	Optometrist	1
251412	Orthoptist	1
2515	Pharmacists	
251511	Hospital Pharmacist	1
251512	Industrial Pharmacist	1
251513	Retail Pharmacist	1
2519	Other Health Diagnostic and Promotion Professionals	
251911	Health Promotion Officer	1
251912	Orthotist or Prosthetist	1
251999	Health Diagnostic and Promotion Professionals nec	1
**252**	**Health Therapy Professionals**	
2521	Chiropractors and Osteopaths	
252111	Chiropractor	1
252112	Osteopath	1
2522	Complementary Health Therapists	
252211	Acupuncturist	1
252212	Homoeopath	1
252213	Naturopath	1
252214	Traditional Chinese Medicine Practitioner	1
252215	Traditional Māori Health Practitioner	1
252299	Complementary Health Therapists nec	1
2523	Dental Practitioners	
252311	Dental Specialist	1
252312	Dentist	1
2524	Occupational Therapists	
252411	Occupational Therapist	1
2525	Physiotherapists	
252511	Physiotherapist	1
2526	Podiatrists	
252611	Podiatrist	1
2527	Speech Professionals and Audiologists	
252711	Audiologist	1
252712	Speech Pathologist (Aus) / Speech Language Therapist (NZ)	1
**253**	**Medical Practitioners**	
2531	Generalist Medical Practitioners	
253111	General Medical Practitioner	1
253112	Resident Medical Officer	1
2532	Anaesthetists	
253211	Anaesthetist	1

MAJOR, SUB–MAJOR, MINOR, UNIT GROUPS AND OCCUPATIONS *continued*

Code	Title	Skill Level
**2**	**PROFESSIONALS** *cont.*	
**25**	**Health Professionals** *cont.*	
**253**	**Medical Practitioners** *cont.*	
2533	Internal Medicine Specialists	
253311	Specialist Physician (General Medicine)	1
253312	Cardiologist	1
253313	Clinical Haematologist	1
253314	Clinical Oncologist	1
253315	Endocrinologist	1
253316	Gastroenterologist	1
253317	Intensive Care Specialist	1
253318	Neurologist	1
253321	Paediatrician	1
253322	Renal Medicine Specialist	1
253323	Rheumatologist	1
253324	Thoracic Medicine Specialist	1
253399	Internal Medicine Specialists nec	1
2534	Psychiatrists	
253411	Psychiatrist	1
2535	Surgeons	
253511	Surgeon (General)	1
253512	Cardiothoracic Surgeon	1
253513	Neurosurgeon	1
253514	Orthopaedic Surgeon	1
253515	Otorhinolaryngologist	1
253516	Paediatric Surgeon	1
253517	Plastic and Reconstructive Surgeon	1
253518	Urologist	1
253521	Vascular Surgeon	1
2539	Other Medical Practitioners	
253911	Dermatologist	1
253912	Emergency Medicine Specialist	1
253913	Obstetrician and Gynaecologist	1
253914	Ophthalmologist	1
253915	Pathologist	1
253916	Radiologist	1
253999	Medical Practitioners nec	1
**254**	**Midwifery and Nursing Professionals**	
2541	Midwives	
254111	Midwife	1
2542	Nurse Educators and Researchers	
254211	Nurse Educator	1
254212	Nurse Researcher	1
2543	Nurse Managers	
254311	Nurse Manager	1
2544	Registered Nurses	
254411	Nurse Practitioner	1
254412	Registered Nurse (Aged Care)	1
254413	Registered Nurse (Child and Family Health)	1
254414	Registered Nurse (Community Health)	1
254415	Registered Nurse (Critical Care and Emergency)	1
254416	Registered Nurse (Developmental Disability)	1
254417	Registered Nurse (Disability and Rehabilitation)	1
254418	Registered Nurse (Medical)	1
254421	Registered Nurse (Medical Practice)	1
254422	Registered Nurse (Mental Health)	1
254423	Registered Nurse (Perioperative)	1
254424	Registered Nurse (Surgical)	1
254499	Registered Nurses nec	1

MAJOR, SUB–MAJOR, MINOR, UNIT GROUPS AND OCCUPATIONS *continued*

		Skill Level
**2**	**PROFESSIONALS** *cont.*	
**26**	**ICT Professionals**	
**261**	**Business and Systems Analysts, and Programmers**	
2611	ICT Business and Systems Analysts	
261111	ICT Business Analyst	1
261112	Systems Analyst	1
2612	Multimedia Specialists and Web Developers	
261211	Multimedia Specialist	1
261212	Web Developer	1
2613	Software and Applications Programmers	
261311	Analyst Programmer	1
261312	Developer Programmer	1
261313	Software Engineer	1
261399	Software and Applications Programmers nec	1
**262**	**Database and Systems Administrators, and ICT Security Specialists**	
2621	Database and Systems Administrators, and ICT Security Specialists	
262111	Database Administrator	1
262112	ICT Security Specialist	1
262113	Systems Administrator	1
**263**	**ICT Network and Support Professionals**	
2631	Computer Network Professionals	
263111	Computer Network and Systems Engineer	1
263112	Network Administrator	1
263113	Network Analyst	1
2632	ICT Support and Test Engineers	
263211	ICT Quality Assurance Engineer	1
263212	ICT Support Engineer	1
263213	ICT Systems Test Engineer	1
263299	ICT Support and Test Engineers nec	1
2633	Telecommunications Engineering Professionals	
263311	Telecommunications Engineer	1
263312	Telecommunications Network Engineer	1
**27**	**Legal, Social and Welfare Professionals**	
**271**	**Legal Professionals**	
2711	Barristers	
271111	Barrister	1
2712	Judicial and Other Legal Professionals	
271211	Judge	1
271212	Magistrate	1
271213	Tribunal Member	1
271299	Judicial and Other Legal Professionals nec	1
2713	Solicitors	
271311	Solicitor	1
**272**	**Social and Welfare Professionals**	
2721	Counsellors	
272111	Careers Counsellor	1
272112	Drug and Alcohol Counsellor	1
272113	Family and Marriage Counsellor	1
272114	Rehabilitation Counsellor	1
272115	Student Counsellor	1
272199	Counsellors nec	1
2722	Ministers of Religion	
272211	Minister of Religion	1

MAJOR, SUB–MAJOR, MINOR, UNIT GROUPS AND OCCUPATIONS *continued*

		Skill Level
**2**	**PROFESSIONALS** *cont.*	
**27**	**Legal, Social and Welfare Professionals** *cont.*	
**272**	**Social and Welfare Professionals** *cont.*	
2723	Psychologists	
272311	Clinical Psychologist	1
272312	Educational Psychologist	1
272313	Organisational Psychologist	1
272314	Psychotherapist	1
272399	Psychologists nec	1
2724	Social Professionals	
272411	Historian	1
272412	Interpreter	1
272413	Translator	1
272499	Social Professionals nec	1
2725	Social Workers	
272511	Social Worker	1
2726	Welfare, Recreation and Community Arts Workers	
272611	Community Arts Worker	1
272612	Recreation Officer (Aus) / Recreation Coordinator (NZ)	1
272613	Welfare Worker	1

MAJOR, SUB–MAJOR, MINOR, UNIT GROUPS AND OCCUPATIONS *continued*

		Skill Level
**3**	**TECHNICIANS AND TRADES WORKERS**	
**31**	**Engineering, ICT and Science Technicians**	
**311**	**Agricultural, Medical and Science Technicians**	
3111	Agricultural Technicians	
311111	Agricultural Technician	2
3112	Medical Technicians	
311211	Anaesthetic Technician	2
311212	Cardiac Technician	2
311213	Medical Laboratory Technician	2
311214	Operating Theatre Technician	2
311215	Pharmacy Technician	2
311299	Medical Technicians nec	2
3113	Primary Products Inspectors	
311311	Fisheries Officer	2
311312	Meat Inspector	2
311313	Quarantine Officer	2
311399	Primary Products Inspectors nec	2
3114	Science Technicians	
311411	Chemistry Technician	2
311412	Earth Science Technician	2
311413	Life Science Technician	2
311414	School Laboratory Technician	2
311499	Science Technicians nec	2
**312**	**Building and Engineering Technicians**	
3121	Architectural, Building and Surveying Technicians	
312111	Architectural Draftsperson	2
312112	Building Associate	2
312113	Building Inspector	2
312114	Construction Estimator	2
312115	Plumbing Inspector	2
312116	Surveying or Cartographic Technician	2
3122	Civil Engineering Draftspersons and Technicians	
312211	Civil Engineering Draftsperson	2
312212	Civil Engineering Technician	2
3123	Electrical Engineering Draftspersons and Technicians	
312311	Electrical Engineering Draftsperson	2
312312	Electrical Engineering Technician	2
3124	Electronic Engineering Draftspersons and Technicians	
312411	Electronic Engineering Draftsperson	2
312412	Electronic Engineering Technician	2
3125	Mechanical Engineering Draftspersons and Technicians	
312511	Mechanical Engineering Draftsperson	2
312512	Mechanical Engineering Technician	2
3126	Safety Inspectors	
312611	Safety Inspector	2
3129	Other Building and Engineering Technicians	
312911	Maintenance Planner	2
312912	Metallurgical or Materials Technician	2
312913	Mine Deputy	2
312999	Building and Engineering Technicians nec	2
**313**	**ICT and Telecommunications Technicians**	
3131	ICT Support Technicians	
313111	Hardware Technician	2
313112	ICT Customer Support Officer	2
313113	Web Administrator	2
313199	ICT Support Technicians nec	2

MAJOR, SUB–MAJOR, MINOR, UNIT GROUPS AND OCCUPATIONS *continued*

Code	Title	Skill Level
**3**	**TECHNICIANS AND TRADES WORKERS** *cont.*	
**31**	**Engineering, ICT and Science Technicians** *cont.*	
**313**	**ICT and Telecommunications Technicians** *cont.*	
3132	Telecommunications Technical Specialists	
313211	Radiocommunications Technician	2
313212	Telecommunications Field Engineer	2
313213	Telecommunications Network Planner	2
313214	Telecommunications Technical Officer or Technologist	2
**32**	**Automotive and Engineering Trades Workers**	
**321**	**Automotive Electricians and Mechanics**	
3211	Automotive Electricians	
321111	Automotive Electrician	3
3212	Motor Mechanics	
321211	Motor Mechanic (General)	3
321212	Diesel Motor Mechanic	3
321213	Motorcycle Mechanic	3
321214	Small Engine Mechanic	3
**322**	**Fabrication Engineering Trades Workers**	
3221	Metal Casting, Forging and Finishing Trades Workers	
322111	Blacksmith	3
322112	Electroplater	3
322113	Farrier	3
322114	Metal Casting Trades Worker	3
322115	Metal Polisher	3
3222	Sheetmetal Trades Workers	
322211	Sheetmetal Trades Worker	3
3223	Structural Steel and Welding Trades Workers	
322311	Metal Fabricator	3
322312	Pressure Welder	3
322313	Welder (First Class) (Aus) / Welder (NZ)	3
**323**	**Mechanical Engineering Trades Workers**	
3231	Aircraft Maintenance Engineers	
323111	Aircraft Maintenance Engineer (Avionics)	3
323112	Aircraft Maintenance Engineer (Mechanical)	3
323113	Aircraft Maintenance Engineer (Structures)	3
3232	Metal Fitters and Machinists	
323211	Fitter (General)	3
323212	Fitter and Turner	3
323213	Fitter-Welder	3
323214	Metal Machinist (First Class)	3
323215	Textile, Clothing and Footwear Mechanic	3
323299	Metal Fitters and Machinists nec	3
3233	Precision Metal Trades Workers	
323311	Engraver	3
323312	Gunsmith	3
323313	Locksmith	3
323314	Precision Instrument Maker and Repairer	3
323315	Saw Maker and Repairer	3
323316	Watch and Clock Maker and Repairer	3
3234	Toolmakers and Engineering Patternmakers	
323411	Engineering Patternmaker	3
323412	Toolmaker	3
**324**	**Panelbeaters, and Vehicle Body Builders, Trimmers and Painters**	
3241	Panelbeaters	
324111	Panelbeater	3

MAJOR, SUB–MAJOR, MINOR, UNIT GROUPS AND OCCUPATIONS *continued*

		Skill Level
3	**TECHNICIANS AND TRADES WORKERS** *cont.*	
32	**Automotive and Engineering Trades Workers** *cont.*	
324	**Panelbeaters, and Vehicle Body Builders, Trimmers and Painters** *cont.*	
3242	Vehicle Body Builders and Trimmers	
324211	Vehicle Body Builder	3
324212	Vehicle Trimmer	3
3243	Vehicle Painters	
324311	Vehicle Painter	3
33	**Construction Trades Workers**	
331	**Bricklayers, and Carpenters and Joiners**	
3311	Bricklayers and Stonemasons	
331111	Bricklayer	3
331112	Stonemason	3
3312	Carpenters and Joiners	
331211	Carpenter and Joiner	3
331212	Carpenter	3
331213	Joiner	3
332	**Floor Finishers and Painting Trades Workers**	
3321	Floor Finishers	
332111	Floor Finisher	3
3322	Painting Trades Workers	
332211	Painting Trades Worker	3
333	**Glaziers, Plasterers and Tilers**	
3331	Glaziers	
333111	Glazier	3
3332	Plasterers	
333211	Fibrous Plasterer	3
333212	Solid Plasterer	3
3333	Roof Tilers	
333311	Roof Tiler	3
3334	Wall and Floor Tilers	
333411	Wall and Floor Tiler	3
334	**Plumbers**	
3341	Plumbers	
334111	Plumber (General)	3
334112	Airconditioning and Mechanical Services Plumber	3
334113	Drainer (Aus) / Drainlayer (NZ)	3
334114	Gasfitter	3
334115	Roof Plumber	3
34	**Electrotechnology and Telecommunications Trades Workers**	
341	**Electricians**	
3411	Electricians	
341111	Electrician (General)	3
341112	Electrician (Special Class)	3
341113	Lift Mechanic	3
342	**Electronics and Telecommunications Trades Workers**	
3421	Airconditioning and Refrigeration Mechanics	
342111	Airconditioning and Refrigeration Mechanic	3
3422	Electrical Distribution Trades Workers	
342211	Electrical Linesworker (Aus) / Electrical Line Mechanic (NZ)	3
342212	Technical Cable Jointer	3

MAJOR, SUB–MAJOR, MINOR, UNIT GROUPS AND OCCUPATIONS *continued*

Code	Title	Skill Level
**3**	**TECHNICIANS AND TRADES WORKERS** *cont.*	
**34**	**Electrotechnology and Telecommunications Trades Workers** *cont.*	
**342**	**Electronics and Telecommunications Trades Workers** *cont.*	
3423	Electronics Trades Workers	
342311	Business Machine Mechanic	3
342312	Communications Operator	3
342313	Electronic Equipment Trades Worker	3
342314	Electronic Instrument Trades Worker (General)	3
342315	Electronic Instrument Trades Worker (Special Class)	3
3424	Telecommunications Trades Workers	
342411	Cabler (Data and Telecommunications)	3
342412	Telecommunications Cable Jointer	3
342413	Telecommunications Linesworker (Aus) / Telecommunications Line Mechanic (NZ)	3
342414	Telecommunications Technician	3
**35**	**Food Trades Workers**	
**351**	**Food Trades Workers**	
3511	Bakers and Pastrycooks	
351111	Baker	3
351112	Pastrycook	3
3512	Butchers and Smallgoods Makers	
351211	Butcher or Smallgoods Maker	3
3513	Chefs	
351311	Chef	2
3514	Cooks	
351411	Cook	3
**36**	**Skilled Animal and Horticultural Workers**	
**361**	**Animal Attendants and Trainers, and Shearers**	
3611	Animal Attendants and Trainers	
361111	Dog Handler or Trainer	3
361112	Horse Trainer	3
361113	Pet Groomer	3
361114	Zookeeper	3
361199	Animal Attendants and Trainers nec	3
3612	Shearers	
361211	Shearer	3
3613	Veterinary Nurses	
361311	Veterinary Nurse	3
**362**	**Horticultural Trades Workers**	
3621	Florists	
362111	Florist	3
3622	Gardeners	
362211	Gardener (General)	3
362212	Arborist	3
362213	Landscape Gardener	3
3623	Greenkeepers	
362311	Greenkeeper	3
3624	Nurserypersons	
362411	Nurseryperson	3
**39**	**Other Technicians and Trades Workers**	
**391**	**Hairdressers**	
3911	Hairdressers	
391111	Hairdresser	3

MAJOR, SUB–MAJOR, MINOR, UNIT GROUPS AND OCCUPATIONS *continued*

Code	Title	Skill Level
**3**	**TECHNICIANS AND TRADES WORKERS** *cont.*	
**39**	**Other Technicians and Trades Workers** *cont.*	
**392**	**Printing Trades Workers**	
3921	Binders, Finishers and Screen Printers	
392111	Binder and Finisher	3
392112	Screen Printer	3
3922	Graphic Pre-press Trades Workers	
392211	Graphic Pre-press Trades Worker	3
3923	Printers	
392311	Printing Machinist	3
392312	Small Offset Printer	3
**393**	**Textile, Clothing and Footwear Trades Workers**	
3931	Canvas and Leather Goods Makers	
393111	Canvas Goods Maker	3
393112	Leather Goods Maker	3
393113	Sail Maker	3
393114	Shoemaker	3
3932	Clothing Trades Workers	
393211	Apparel Cutter	3
393212	Clothing Patternmaker	3
393213	Dressmaker or Tailor	3
393299	Clothing Trades Workers nec	3
3933	Upholsterers	
393311	Upholsterer	3
**394**	**Wood Trades Workers**	
3941	Cabinetmakers	
394111	Cabinetmaker	3
3942	Wood Machinists and Other Wood Trades Workers	
394211	Furniture Finisher	3
394212	Picture Framer	3
394213	Wood Machinist	3
394214	Wood Turner	3
394299	Wood Machinists and Other Wood Trades Workers nec	3
**399**	**Miscellaneous Technicians and Trades Workers**	
3991	Boat Builders and Shipwrights	
399111	Boat Builder and Repairer	3
399112	Shipwright	3
3992	Chemical, Gas, Petroleum and Power Generation Plant Operators	
399211	Chemical Plant Operator	3
399212	Gas or Petroleum Operator	3
399213	Power Generation Plant Operator	3
3993	Gallery, Library and Museum Technicians	
399311	Gallery or Museum Technician	2
399312	Library Technician	2
3994	Jewellers	
399411	Jeweller	3
3995	Performing Arts Technicians	
399511	Broadcast Transmitter Operator	3
399512	Camera Operator (Film, Television or Video)	3
399513	Light Technician	3
399514	Make Up Artist	3
399515	Musical Instrument Maker or Repairer	3
399516	Sound Technician	3
399517	Television Equipment Operator	3
399599	Performing Arts Technicians nec	3
3996	Signwriters	
399611	Signwriter	3

MAJOR, SUB–MAJOR, MINOR, UNIT GROUPS AND OCCUPATIONS *continued*

Code	Title	*Skill Level*
**3**	**TECHNICIANS AND TRADES WORKERS** *cont.*	
**39**	**Other Technicians and Trades Workers** *cont.*	
**399**	**Miscellaneous Technicians and Trades Workers** *cont.*	
3999	Other Miscellaneous Technicians and Trades Workers	
399911	Diver	3
399912	Interior Decorator	2
399913	Optical Dispenser (Aus) / Dispensing Optician (NZ)	3
399914	Optical Mechanic	3
399915	Photographer's Assistant	3
399916	Plastics Technician	3
399917	Wool Classer	3
399999	Technicians and Trades Workers nec	3

MAJOR, SUB–MAJOR, MINOR, UNIT GROUPS AND OCCUPATIONS *continued*

Code	Title	Skill Level
**4**	**COMMUNITY AND PERSONAL SERVICE WORKERS**	
**41**	**Health and Welfare Support Workers**	
**411**	**Health and Welfare Support Workers**	
4111	Ambulance Officers and Paramedics	
411111	Ambulance Officer	2
411112	Intensive Care Ambulance Paramedic (Aus) / Ambulance Paramedic (NZ)	2
4112	Dental Hygienists, Technicians and Therapists	
411211	Dental Hygienist	2
411212	Dental Prosthetist	2
411213	Dental Technician	2
411214	Dental Therapist	2
4113	Diversional Therapists	
411311	Diversional Therapist	3
4114	Enrolled and Mothercraft Nurses	
411411	Enrolled Nurse	2
411412	Mothercraft Nurse	2
4115	Indigenous Health Workers	
411511	Aboriginal and Torres Strait Islander Health Worker	2
411512	Kaiāwhina (Hauora) (Māori Health Assistant)	2
4116	Massage Therapists	
411611	Massage Therapist	2
4117	Welfare Support Workers	
411711	Community Worker	2
411712	Disabilities Services Officer	2
411713	Family Support Worker	2
411714	Parole or Probation Officer	2
411715	Residential Care Officer	2
411716	Youth Worker	2
**42**	**Carers and Aides**	
**421**	**Child Carers**	
4211	Child Carers	
421111	Child Care Worker	4
421112	Family Day Care Worker	4
421113	Nanny	4
421114	Out of School Hours Care Worker	4
**422**	**Education Aides**	
4221	Education Aides	
422111	Aboriginal and Torres Strait Islander Education Worker	4
422112	Integration Aide	4
422113	Kaiāwhina Kōhanga Reo (Māori Language Nest Assistant)	4
422114	Kaiāwhina Kura Kaupapa Māori (Māori-medium School Assistant)	4
422115	Preschool Aide	4
422116	Teachers' Aide	4
**423**	**Personal Carers and Assistants**	
4231	Aged and Disabled Carers	
423111	Aged or Disabled Carer	4
4232	Dental Assistants	
423211	Dental Assistant	4
4233	Nursing Support and Personal Care Workers	
423311	Hospital Orderly	4
423312	Nursing Support Worker	4
423313	Personal Care Assistant	4
423314	Therapy Aide	4
4234	Special Care Workers	
423411	Child or Youth Residential Care Assistant	4
423412	Hostel Parent	4
423413	Refuge Worker	4

MAJOR, SUB–MAJOR, MINOR, UNIT GROUPS AND OCCUPATIONS *continued*

		Skill Level
**4**	**COMMUNITY AND PERSONAL SERVICE WORKERS** *cont.*	
**43**	**Hospitality Workers**	
**431**	**Hospitality Workers**	
4311	Bar Attendants and Baristas	
431111	Bar Attendant	4
431112	Barista	4
4312	Cafe Workers	
431211	Cafe Worker	5
4313	Gaming Workers	
431311	Gaming Worker	4
4314	Hotel Service Managers	
431411	Hotel Service Manager	3
4315	Waiters	
431511	Waiter	4
4319	Other Hospitality Workers	
431911	Bar Useful or Busser	5
431912	Doorperson or Luggage Porter	5
431999	Hospitality Workers nec	5
**44**	**Protective Service Workers**	
**441**	**Defence Force Members, Fire Fighters and Police**	
4411	Defence Force Members - Other Ranks	
441111	Defence Force Member - Other Ranks	3
4412	Fire and Emergency Workers	
441211	Emergency Service Worker	3
441212	Fire Fighter	3
4413	Police	
441311	Detective	2
441312	Police Officer	2
**442**	**Prison and Security Officers**	
4421	Prison Officers	
442111	Prison Officer	4
4422	Security Officers and Guards	
442211	Alarm, Security or Surveillance Monitor	5
442212	Armoured Car Escort	5
442213	Crowd Controller	4
442214	Private Investigator	4
442215	Retail Loss Prevention Officer	5
442216	Security Consultant	3
442217	Security Officer	5
442299	Security Officers and Guards nec	5
**45**	**Sports and Personal Service Workers**	
**451**	**Personal Service and Travel Workers**	
4511	Beauty Therapists	
451111	Beauty Therapist	4
4512	Driving Instructors	
451211	Driving Instructor	3
4513	Funeral Workers	
451311	Funeral Director	2
451399	Funeral Workers nec	3
4514	Gallery, Museum and Tour Guides	
451411	Gallery or Museum Guide	4
451412	Tour Guide	4
4515	Personal Care Consultants	
451511	Natural Remedy Consultant	4
451512	Weight Loss Consultant	4

MAJOR, SUB–MAJOR, MINOR, UNIT GROUPS AND OCCUPATIONS *continued*

Code	Title	Skill Level
**4**	**COMMUNITY AND PERSONAL SERVICE WORKERS** *cont.*	
**45**	**Sports and Personal Service Workers** *cont.*	
**451**	**Personal Service and Travel Workers** *cont.*	
4516	Tourism and Travel Advisers	
451611	Tourist Information Officer	4
451612	Travel Consultant	4
4517	Travel Attendants	
451711	Flight Attendant	3
451799	Travel Attendants nec	3
4518	Other Personal Service Workers	
451811	Civil Celebrant	4
451812	Hair or Beauty Salon Assistant	5
451813	Sex Worker or Escort	5
451899	Personal Service Workers nec	4
**452**	**Sports and Fitness Workers**	
4521	Fitness Instructors	
452111	Fitness Instructor	4
4522	Outdoor Adventure Guides	
452211	Bungy Jump Master	4
452212	Fishing Guide	4
452213	Hunting Guide	4
452214	Mountain or Glacier Guide	4
452215	Outdoor Adventure Instructor	4
452216	Trekking Guide	4
452217	Whitewater Rafting Guide	4
452299	Outdoor Adventure Guides nec	4
4523	Sports Coaches, Instructors and Officials	
452311	Diving Instructor (Open Water)	3
452312	Gymnastics Coach or Instructor	3
452313	Horse Riding Coach or Instructor	3
452314	Snowsport Instructor	3
452315	Swimming Coach or Instructor	3
452316	Tennis Coach	3
452317	Other Sports Coach or Instructor	3
452318	Dog or Horse Racing Official	3
452321	Sports Development Officer	2
452322	Sports Umpire	3
452323	Other Sports Official	3
4524	Sportspersons	
452411	Footballer	3
452412	Golfer	3
452413	Jockey	3
452414	Lifeguard	3
452499	Sportspersons nec	3

MAJOR, SUB–MAJOR, MINOR, UNIT GROUPS AND OCCUPATIONS *continued*

		Skill Level
**5**	**CLERICAL AND ADMINISTRATIVE WORKERS**	
**51**	**Office Managers and Program Administrators**	
**511**	**Contract, Program and Project Administrators**	
5111	Contract, Program and Project Administrators	
511111	Contract Administrator	2
511112	Program or Project Administrator	2
**512**	**Office and Practice Managers**	
5121	Office Managers	
512111	Office Manager	2
5122	Practice Managers	
512211	Health Practice Manager	2
512299	Practice Managers nec	2
**52**	**Personal Assistants and Secretaries**	
**521**	**Personal Assistants and Secretaries**	
5211	Personal Assistants	
521111	Personal Assistant	3
5212	Secretaries	
521211	Secretary (General)	3
521212	Legal Secretary	3
**53**	**General Clerical Workers**	
**531**	**General Clerks**	
5311	General Clerks	
531111	General Clerk	4
**532**	**Keyboard Operators**	
5321	Keyboard Operators	
532111	Data Entry Operator	4
532112	Machine Shorthand Reporter	4
532113	Word Processing Operator	4
**54**	**Inquiry Clerks and Receptionists**	
**541**	**Call or Contact Centre Information Clerks**	
5411	Call or Contact Centre Workers	
541111	Call or Contact Centre Team Leader	3
541112	Call or Contact Centre Operator	4
5412	Inquiry Clerks	
541211	Inquiry Clerk	4
**542**	**Receptionists**	
5421	Receptionists	
542111	Receptionist (General)	4
542112	Admissions Clerk	4
542113	Hotel or Motel Receptionist	4
542114	Medical Receptionist	4
**55**	**Numerical Clerks**	
**551**	**Accounting Clerks and Bookkeepers**	
5511	Accounting Clerks	
551111	Accounts Clerk	4
551112	Cost Clerk	4
5512	Bookkeepers	
551211	Bookkeeper	4
5513	Payroll Clerks	
551311	Payroll Clerk	4
**552**	**Financial and Insurance Clerks**	
5521	Bank Workers	
552111	Bank Worker	4
5522	Credit and Loans Officers	
552211	Credit or Loans Officer	4

MAJOR, SUB–MAJOR, MINOR, UNIT GROUPS AND OCCUPATIONS *continued*

Code	Title	Skill Level
5	**CLERICAL AND ADMINISTRATIVE WORKERS** *cont.*	
55	**Numerical Clerks** *cont.*	
552	**Financial and Insurance Clerks** *cont.*	
5523	Insurance, Money Market and Statistical Clerks	
552311	Bookmaker	4
552312	Insurance Consultant	4
552313	Money Market Clerk	4
552314	Statistical Clerk	4
56	**Clerical and Office Support Workers**	
561	**Clerical and Office Support Workers**	
5611	Betting Clerks	
561111	Betting Agency Counter Clerk	5
561112	Bookmaker's Clerk	5
561113	Telephone Betting Clerk	5
561199	Betting Clerks nec	5
5612	Couriers and Postal Deliverers	
561211	Courier	5
561212	Postal Delivery Officer	5
5613	Filing and Registry Clerks	
561311	Filing or Registry Clerk	5
5614	Mail Sorters	
561411	Mail Clerk	5
561412	Postal Sorting Officer	5
5615	Survey Interviewers	
561511	Survey Interviewer	5
5616	Switchboard Operators	
561611	Switchboard Operator	5
5619	Other Clerical and Office Support Workers	
561911	Classified Advertising Clerk	5
561912	Meter Reader	5
561913	Parking Inspector	5
561999	Clerical and Office Support Workers nec	5
59	**Other Clerical and Administrative Workers**	
591	**Logistics Clerks**	
5911	Purchasing and Supply Logistics Clerks	
591111	Order Clerk	4
591112	Production Clerk	4
591113	Purchasing Officer	4
591114	Sales Clerk	4
591115	Stock Clerk	4
591116	Warehouse Administrator	4
5912	Transport and Despatch Clerks	
591211	Despatching and Receiving Clerk	4
591212	Import-Export Clerk	4
599	**Miscellaneous Clerical and Administrative Workers**	
5991	Conveyancers and Legal Executives	
599111	Conveyancer	2
599112	Legal Executive	2
5992	Court and Legal Clerks	
599211	Clerk of Court	3
599212	Court Bailiff or Sheriff (Aus) / Court Collections Officer (NZ)	3
599213	Court Orderly (Aus) / Court Registry Officer (NZ)	3
599214	Law Clerk	3
599215	Trust Officer	3
5993	Debt Collectors	
599311	Debt Collector	4

MAJOR, SUB–MAJOR, MINOR, UNIT GROUPS AND OCCUPATIONS *continued*

		Skill Level
**5**	**CLERICAL AND ADMINISTRATIVE WORKERS** *cont.*	
**59**	**Other Clerical and Administrative Workers** *cont.*	
**599**	**Miscellaneous Clerical and Administrative Workers** *cont.*	
5994	Human Resource Clerks	
599411	Human Resources Clerk	4
5995	Inspectors and Regulatory Officers	
599511	Customs Officer	4
599512	Immigration Officer	4
599513	Motor Vehicle Licence Examiner	4
599514	Noxious Weeds and Pest Inspector	4
599515	Social Security Assessor	4
599516	Taxation Inspector	4
599517	Train Examiner	4
599518	Transport Operations Inspector	4
599521	Water Inspector	4
599599	Inspectors and Regulatory Officers nec	4
5996	Insurance Investigators, Loss Adjusters and Risk Surveyors	
599611	Insurance Investigator	3
599612	Insurance Loss Adjuster	3
599613	Insurance Risk Surveyor	3
5997	Library Assistants	
599711	Library Assistant	4
5999	Other Miscellaneous Clerical and Administrative Workers	
599911	Coding Clerk	4
599912	Production Assistant (Film, Television, Radio or Stage)	4
599913	Proof Reader	4
599914	Radio Despatcher	4
599999	Clerical and Administrative Workers nec	4

MAJOR, SUB–MAJOR, MINOR, UNIT GROUPS AND OCCUPATIONS *continued*

		Skill Level
6	SALES WORKERS	
61	Sales Representatives and Agents	
611	**Insurance Agents and Sales Representatives**	
6111	Auctioneers, and Stock and Station Agents	
611111	Auctioneer	3
611112	Stock and Station Agent	3
6112	Insurance Agents	
611211	Insurance Agent	3
6113	Sales Representatives	
611311	Sales Representative (Building and Plumbing Supplies)	4
611312	Sales Representative (Business Services)	4
611313	Sales Representative (Motor Vehicle Parts and Accessories)	4
611314	Sales Representative (Personal and Household Goods)	4
611399	Sales Representatives nec	4
612	**Real Estate Sales Agents**	
6121	Real Estate Sales Agents	
612111	Business Broker	3
612112	Property Manager	3
612113	Real Estate Agency Principal (Aus) / Real Estate Agency Licensee (NZ)	2
612114	Real Estate Agent	3
612115	Real Estate Representative	3
62	Sales Assistants and Salespersons	
621	**Sales Assistants and Salespersons**	
6211	Sales Assistants (General)	
621111	Sales Assistant (General)	5
6212	ICT Sales Assistants	
621211	ICT Sales Assistant	5
6213	Motor Vehicle and Vehicle Parts Salespersons	
621311	Motor Vehicle or Caravan Salesperson	4
621312	Motor Vehicle Parts Interpreter (Aus) / Automotive Parts Salesperson (NZ)	4
6214	Pharmacy Sales Assistants	
621411	Pharmacy Sales Assistant	5
6215	Retail Supervisors	
621511	Retail Supervisor	4
6216	Service Station Attendants	
621611	Service Station Attendant	5
6217	Street Vendors and Related Salespersons	
621711	Cash Van Salesperson	5
621712	Door-to-door Salesperson	5
621713	Street Vendor	5
6219	Other Sales Assistants and Salespersons	
621911	Materials Recycler	5
621912	Rental Salesperson	5
621999	Sales Assistants and Salespersons nec	5
63	Sales Support Workers	
631	**Checkout Operators and Office Cashiers**	
6311	Checkout Operators and Office Cashiers	
631111	Checkout Operator	5
631112	Office Cashier	5
639	**Miscellaneous Sales Support Workers**	
6391	Models and Sales Demonstrators	
639111	Model	5
639112	Sales Demonstrator	5
6392	Retail and Wool Buyers	
639211	Retail Buyer	3
639212	Wool Buyer	3

MAJOR, SUB–MAJOR, MINOR, UNIT GROUPS AND OCCUPATIONS *continued*

		Skill Level
**6**	**SALES WORKERS** *cont.*	
**63**	**Sales Support Workers** *cont.*	
**639**	**Miscellaneous Sales Support Workers** *cont.*	
6393	Telemarketers	
639311	Telemarketer	5
6394	Ticket Salespersons	
639411	Ticket Seller	5
639412	Transport Conductor	5
6395	Visual Merchandisers	
639511	Visual Merchandiser	4
6399	Other Sales Support Workers	
639911	Other Sales Support Worker	5

MAJOR, SUB–MAJOR, MINOR, UNIT GROUPS AND OCCUPATIONS *continued*

Code	Title	Skill Level
**7**	**MACHINERY OPERATORS AND DRIVERS**	
**71**	**Machine and Stationary Plant Operators**	
**711**	**Machine Operators**	
7111	Clay, Concrete, Glass and Stone Processing Machine Operators	
711111	Clay Products Machine Operator	4
711112	Concrete Products Machine Operator	4
711113	Glass Production Machine Operator	4
711114	Stone Processing Machine Operator	4
711199	Clay, Concrete, Glass and Stone Processing Machine Operators nec	4
7112	Industrial Spraypainters	
711211	Industrial Spraypainter	4
7113	Paper and Wood Processing Machine Operators	
711311	Paper Products Machine Operator	4
711312	Wood Processing Machine Operator	4
7114	Photographic Developers and Printers	
711411	Photographic Developer and Printer	4
7115	Plastics and Rubber Production Machine Operators	
711511	Plastic Cablemaking Machine Operator	4
711512	Plastic Compounding and Reclamation Machine Operator	4
711513	Plastics Fabricator or Welder	4
711514	Plastics Production Machine Operator (General)	4
711515	Reinforced Plastic and Composite Production Worker	4
711516	Rubber Production Machine Operator	4
711599	Plastics and Rubber Production Machine Operators nec	4
7116	Sewing Machinists	
711611	Sewing Machinist	4
7117	Textile and Footwear Production Machine Operators	
711711	Footwear Production Machine Operator	4
711712	Hide and Skin Processing Machine Operator	4
711713	Knitting Machine Operator	4
711714	Textile Dyeing and Finishing Machine Operator	4
711715	Weaving Machine Operator	4
711716	Yarn Carding and Spinning Machine Operator	4
711799	Textile and Footwear Production Machine Operators nec	4
7119	Other Machine Operators	
711911	Chemical Production Machine Operator	4
711912	Motion Picture Projectionist	4
711913	Sand Blaster	4
711914	Sterilisation Technician	4
711999	Machine Operators nec	4
**712**	**Stationary Plant Operators**	
7121	Crane, Hoist and Lift Operators	
712111	Crane, Hoist or Lift Operator	4
7122	Drillers, Miners and Shot Firers	
712211	Driller	4
712212	Miner	4
712213	Shot Firer	4
7123	Engineering Production Systems Workers	
712311	Engineering Production Systems Worker	4

MAJOR, SUB–MAJOR, MINOR, UNIT GROUPS AND OCCUPATIONS *continued*

		Skill Level
**7**	**MACHINERY OPERATORS AND DRIVERS** *cont.*	
**71**	**Machine and Stationary Plant Operators** *cont.*	
**712**	**Stationary Plant Operators** *cont.*	
7129	Other Stationary Plant Operators	
712911	Boiler or Engine Operator	4
712912	Bulk Materials Handling Plant Operator	4
712913	Cement Production Plant Operator	4
712914	Concrete Batching Plant Operator	4
712915	Concrete Pump Operator	4
712916	Paper and Pulp Mill Operator	4
712917	Railway Signal Operator	4
712918	Train Controller	4
712921	Waste Water or Water Plant Operator	4
712922	Weighbridge Operator	4
712999	Stationary Plant Operators nec	4
**72**	**Mobile Plant Operators**	
**721**	**Mobile Plant Operators**	
7211	Agricultural, Forestry and Horticultural Plant Operators	
721111	Agricultural and Horticultural Mobile Plant Operator	4
721112	Logging Plant Operator	4
7212	Earthmoving Plant Operators	
721211	Earthmoving Plant Operator (General)	4
721212	Backhoe Operator	4
721213	Bulldozer Operator	4
721214	Excavator Operator	4
721215	Grader Operator	4
721216	Loader Operator	4
7213	Forklift Drivers	
721311	Forklift Driver	4
7219	Other Mobile Plant Operators	
721911	Aircraft Baggage Handler and Airline Ground Crew	4
721912	Linemarker	4
721913	Paving Plant Operator	4
721914	Railway Track Plant Operator	4
721915	Road Roller Operator	4
721916	Streetsweeper Operator	4
721999	Mobile Plant Operators nec	4
**73**	**Road and Rail Drivers**	
**731**	**Automobile, Bus and Rail Drivers**	
7311	Automobile Drivers	
731111	Chauffeur	4
731112	Taxi Driver	4
731199	Automobile Drivers nec	4
7312	Bus and Coach Drivers	
731211	Bus Driver	4
731212	Charter and Tour Bus Driver	4
731213	Passenger Coach Driver	4
7313	Train and Tram Drivers	
731311	Train Driver	4
731312	Tram Driver	4
**732**	**Delivery Drivers**	
7321	Delivery Drivers	
732111	Delivery Driver	4

MAJOR, SUB–MAJOR, MINOR, UNIT GROUPS AND OCCUPATIONS *continued*

Code	Title	*Skill Level*
**7**	**MACHINERY OPERATORS AND DRIVERS** *cont.*	
**73**	**Road and Rail Drivers** *cont.*	
**733**	**Truck Drivers**	
7331	Truck Drivers	
733111	Truck Driver (General)	4
733112	Aircraft Refueller	4
733113	Furniture Removalist	4
733114	Tanker Driver	4
733115	Tow Truck Driver	4
**74**	**Storepersons**	
**741**	**Storepersons**	
7411	Storepersons	
741111	Storeperson	4

MAJOR, SUB–MAJOR, MINOR, UNIT GROUPS AND OCCUPATIONS *continued*

Code	Title	Skill Level
**8**	**LABOURERS**	
**81**	**Cleaners and Laundry Workers**	
**811**	**Cleaners and Laundry Workers**	
8111	Car Detailers	
811111	Car Detailer	5
8112	Commercial Cleaners	
811211	Commercial Cleaner	5
8113	Domestic Cleaners	
811311	Domestic Cleaner	5
8114	Housekeepers	
811411	Commercial Housekeeper	5
811412	Domestic Housekeeper	5
8115	Laundry Workers	
811511	Laundry Worker (General)	5
811512	Drycleaner	5
811513	Ironer or Presser	5
8116	Other Cleaners	
811611	Carpet Cleaner	5
811612	Window Cleaner	5
811699	Cleaners nec	5
**82**	**Construction and Mining Labourers**	
**821**	**Construction and Mining Labourers**	
8211	Building and Plumbing Labourers	
821111	Builder's Labourer	5
821112	Drainage, Sewerage and Stormwater Labourer	5
821113	Earthmoving Labourer	5
821114	Plumber's Assistant	5
8212	Concreters	
821211	Concreter	5
8213	Fencers	
821311	Fencer	4
8214	Insulation and Home Improvement Installers	
821411	Building Insulation Installer	4
821412	Home Improvement Installer	4
8215	Paving and Surfacing Labourers	
821511	Paving and Surfacing Labourer	5
8216	Railway Track Workers	
821611	Railway Track Worker	4
8217	Structural Steel Construction Workers	
821711	Construction Rigger	4
821712	Scaffolder	4
821713	Steel Fixer	4
821714	Structural Steel Erector	4
8219	Other Construction and Mining Labourers	
821911	Crane Chaser	5
821912	Driller's Assistant	5
821913	Lagger	5
821914	Mining Support Worker	5
821915	Surveyor's Assistant	5

MAJOR, SUB–MAJOR, MINOR, UNIT GROUPS AND OCCUPATIONS *continued*

		Skill Level
**8**	**LABOURERS** *cont.*	
**83**	**Factory Process Workers**	
**831**	**Food Process Workers**	
8311	Food and Drink Factory Workers	
831111	Baking Factory Worker	5
831112	Brewery Worker	5
831113	Confectionery Maker	5
831114	Dairy Products Maker	5
831115	Fruit and Vegetable Factory Worker	5
831116	Grain Mill Worker	5
831117	Sugar Mill Worker	5
831118	Winery Cellar Hand	5
831199	Food and Drink Factory Workers nec	5
8312	Meat Boners and Slicers, and Slaughterers	
831211	Meat Boner and Slicer	4
831212	Slaughterer	4
8313	Meat, Poultry and Seafood Process Workers	
831311	Meat Process Worker	5
831312	Poultry Process Worker	5
831313	Seafood Process Worker	5
**832**	**Packers and Product Assemblers**	
8321	Packers	
832111	Chocolate Packer	5
832112	Container Filler	5
832113	Fruit and Vegetable Packer	5
832114	Meat Packer	5
832115	Seafood Packer	5
832199	Packers nec	5
8322	Product Assemblers	
832211	Product Assembler	5
**839**	**Miscellaneous Factory Process Workers**	
8391	Metal Engineering Process Workers	
839111	Metal Engineering Process Worker	5
8392	Plastics and Rubber Factory Workers	
839211	Plastics Factory Worker	5
839212	Rubber Factory Worker	5
8393	Product Quality Controllers	
839311	Product Examiner	4
839312	Product Grader	4
839313	Product Tester	4
8394	Timber and Wood Process Workers	
839411	Paper and Pulp Mill Worker	5
839412	Sawmill or Timber Yard Worker	5
839413	Wood and Wood Products Factory Worker	5
8399	Other Factory Process Workers	
839911	Cement and Concrete Plant Worker	5
839912	Chemical Plant Worker	5
839913	Clay Processing Factory Worker	5
839914	Fabric and Textile Factory Worker	5
839915	Footwear Factory Worker	5
839916	Glass Processing Worker	5
839917	Hide and Skin Processing Worker	5
839999	Factory Process Workers nec	5
**84**	**Farm, Forestry and Garden Workers**	
**841**	**Farm, Forestry and Garden Workers**	
8411	Aquaculture Workers	
841111	Aquaculture Worker	5

MAJOR, SUB–MAJOR, MINOR, UNIT GROUPS AND OCCUPATIONS *continued*

Code	Title	Skill Level
8	**LABOURERS** *cont.*	
84	**Farm, Forestry and Garden Workers** *cont.*	
841	**Farm, Forestry and Garden Workers** *cont.*	
8412	Crop Farm Workers	
841211	Fruit or Nut Farm Worker	5
841212	Fruit or Nut Picker	5
841213	Grain, Oilseed or Pasture Farm Worker (Aus) / Field Crop Farm Worker (NZ)	5
841214	Vegetable Farm Worker (Aus) / Market Garden Worker (NZ)	5
841215	Vegetable Picker	5
841216	Vineyard Worker	5
841299	Crop Farm Workers nec	5
8413	Forestry and Logging Workers	
841311	Forestry Worker	4
841312	Logging Assistant	4
841313	Tree Faller	4
8414	Garden and Nursery Labourers	
841411	Garden Labourer	5
841412	Horticultural Nursery Assistant	5
8415	Livestock Farm Workers	
841511	Beef Cattle Farm Worker	5
841512	Dairy Cattle Farm Worker	5
841513	Mixed Livestock Farm Worker	5
841514	Poultry Farm Worker	5
841515	Sheep Farm Worker	5
841516	Stablehand	5
841517	Wool Handler	5
841599	Livestock Farm Workers nec	5
8416	Mixed Crop and Livestock Farm Workers	
841611	Mixed Crop and Livestock Farm Worker	5
8419	Other Farm, Forestry and Garden Workers	
841911	Hunter-Trapper	5
841912	Pest or Weed Controller	4
841999	Farm, Forestry and Garden Workers nec	5
85	**Food Preparation Assistants**	
851	**Food Preparation Assistants**	
8511	Fast Food Cooks	
851111	Fast Food Cook	5
8512	Food Trades Assistants	
851211	Pastrycook's Assistant	5
851299	Food Trades Assistants nec	5
8513	Kitchenhands	
851311	Kitchenhand	5
89	**Other Labourers**	
891	**Freight Handlers and Shelf Fillers**	
8911	Freight and Furniture Handlers	
891111	Freight Handler (Rail or Road)	5
891112	Truck Driver's Offsider	5
891113	Waterside Worker	5
8912	Shelf Fillers	
891211	Shelf Filler	5
899	**Miscellaneous Labourers**	
8991	Caretakers	
899111	Caretaker	5
8992	Deck and Fishing Hands	
899211	Deck Hand	4
899212	Fishing Hand	4

MAJOR, SUB–MAJOR, MINOR, UNIT GROUPS AND OCCUPATIONS *continued*

		Skill Level
**8**	**LABOURERS** *cont.*	
**89**	**Other Labourers** *cont.*	
**899**	**Miscellaneous Labourers** *cont.*	
8993	Handypersons	
899311	Handyperson	5
8994	Motor Vehicle Parts and Accessories Fitters	
899411	Motor Vehicle Parts and Accessories Fitter (General)	4
899412	Autoglazier	4
899413	Exhaust and Muffler Fitter	4
899414	Radiator Fitter	4
899415	Tyre Fitter	4
8995	Printing Assistants and Table Workers	
899511	Printer's Assistant	4
899512	Printing Table Worker	4
8996	Recycling and Rubbish Collectors	
899611	Recycling or Rubbish Collector	5
8997	Vending Machine Attendants	
899711	Vending Machine Attendant	5
8999	Other Miscellaneous Labourers	
899911	Bicycle Mechanic	5
899912	Car Park Attendant	5
899913	Crossing Supervisor	5
899914	Electrical or Telecommunications Trades Assistant	5
899915	Leaflet or Newspaper Deliverer	5
899916	Mechanic's Assistant	5
899917	Railways Assistant	5
899918	Sign Erector	5
899921	Ticket Collector or Usher	5
899922	Trolley Collector	5
899999	Labourers nec	5

INTERPRETING ANZSCO OCCUPATION DEFINITIONS

ANZSCO is primarily a statistical classification designed to aggregate and organise data collected about jobs or individuals. The classification definitions are based on the skill level and specialisation usually necessary to perform the tasks of the specific occupation, or of most occupations in the group. The definitions and skill level statements apply to the occupation and not persons working in the occupation. The allocation of a particular occupation to a particular skill level should be seen as indicative only and should not be used prescriptively.

The definitional material describing each occupation is intended primarily as an aid to interpreting occupation statistics classified to ANZSCO. The descriptions are, therefore, only a guide to the tasks undertaken and skills involved in various occupations and are not a definitive statement of what is required.

FORMAT OF THE DEFINITIONS

This publication contains definitions for the major, sub-major, minor and unit groups, and all occupations in ANZSCO. The format of the definitions may vary slightly between the hierarchical levels, but all contain similar elements.

Major, sub-major, minor and unit group definitions

The elements of major, sub-major, minor and unit group definitions are as follows:

- Main heading - consists of the level in the ANZSCO hierarchy (e.g. minor group), the code, and the title of the group in that order.
- Lead statement - describes the main activities undertaken in the group and, indirectly, the group's boundaries.
- Inclusion and exclusion statements - are included only where necessary to avoid potential for confusion and to clarify the scope of the group.
- Task list - a representative list of the principal or indicative tasks undertaken in the group. Because of the disparate nature of the occupations contained in 'miscellaneous' and 'other' groups, no task lists are provided for these groups.
- Skill level statement - specifies the requirements for competent performance of occupations in the group, expressed in terms of the amount of formal education and training, previous experience and on-the-job training. Any special requirements, such as registration or licensing, are indicated in this statement.
- List of lower categories - lists the categories (code and title) in the hierarchical level immediately below the group being defined.

The elements of major, sub-major, minor and unit group definitions are presented in the order shown above.

Occupation definitions

The elements of occupation definitions are as follows:

- Code - the numerical representation of the occupation.

Occupation definitions continued

- Principal title - the title which best describes the particular occupation. It is generally the most commonly used title, although there are exceptions in cases where the most commonly used title is too broad or too narrow in meaning for the purposes of the ANZSCO occupation, or where occupations of different content are usually known by the same title.
  A small number of occupations are known by different titles in Australia and New Zealand. In these cases, a dual title, which notes the country-specific titles, has been used to describe the occupation, e.g. 'Migration Agent (Aus) / Immigration Consultant (NZ)'.
- Alternative title - any commonly used alternative title (or titles) for the occupation. These alternative titles have the same meaning as the principal title but may be less commonly used.
- Lead statement - describes the nature of the occupation, summarising the main activities undertaken and, indirectly, the occupation's boundaries. Any special requirements, such as registration or licensing, are indicated in this statement.
- Skill level - the number equating to the ANZSCO skill level of the occupation.
- Specialisation titles - any commonly used titles which refer to a subset of jobs belonging to the occupation designated in the principal title. These jobs involve the performance of specialised tasks rather than the broader range of tasks usually performed in the occupation.

The elements of occupation definitions are presented in the order shown above.

MAJOR GROUP 1 MANAGERS

MANAGERS plan, organise, direct, control, coordinate and review the operations of government, commercial, agricultural, industrial, non-profit and other organisations, and departments.

Indicative Skill Level:
Most occupations in this major group have a level of skill commensurate with the qualifications and experience outlined below.

In Australia:

Bachelor degree or higher qualification. At least five years of relevant experience may substitute for the formal qualification (ANZSCO Skill Level 1); or

AQF Associate Degree, Advanced Diploma or Diploma, or at least three years of relevant experience (ANZSCO Skill Level 2)

In New Zealand:

Bachelor degree or higher qualification. At least five years of relevant experience may substitute for the formal qualification (ANZSCO Skill Level 1); or

NZ Register Diploma, or at least three years of relevant experience (ANZSCO Skill Level 2)

In some instances relevant experience and/or on-the-job training may be required in addition to the formal qualification.

Tasks Include:

- setting the overall direction and objectives of organisations and departments within organisations
- formulating, administering and reviewing policy and legislation to ensure organisational and departmental objectives are met
- directing and coordinating the allocation of assets and resources
- directing, controlling and coordinating the activities of organisations and departments, either personally or through senior subordinate staff
- monitoring and evaluating overall organisational and departmental performance, and adjusting policies, rules and regulations to ensure objectives are met
- representing the organisation at official occasions, in negotiations, at conventions, seminars, public hearings and forums, and liaising between areas of responsibility

Occupations in this major group are classified into the following sub-major groups:

11 Chief Executives, General Managers and Legislators
12 Farmers and Farm Managers
13 Specialist Managers
14 Hospitality, Retail and Service Managers

SUB-MAJOR GROUP 11 CHIEF EXECUTIVES, GENERAL MANAGERS AND LEGISLATORS

CHIEF EXECUTIVES, GENERAL MANAGERS AND LEGISLATORS plan, organise, direct, control and review the overall operations of organisations and their major programs, and represent constituencies in parliaments and local government authorities.

Indicative Skill Level:

In Australia and New Zealand:

Most occupations in this sub-major group have a level of skill commensurate with a bachelor degree or higher qualification. At least five years of relevant experience may substitute for the formal qualification. In some instances relevant experience and/or on-the-job training may be required in addition to the formal qualification (ANZSCO Skill Level 1).

Tasks Include:

- determining and setting the overall direction and objectives of organisations
- determining and formulating policies, rules and regulations to govern the overall operations of organisations
- directing and controlling the overall operations of organisations to ensure objectives are met
- monitoring and evaluating the overall performance of organisations and adjusting policies, rules and regulations to ensure objectives are met
- representing the organisation at official occasions, in negotiations, at conventions, seminars, public hearings and forums, and liaising between areas of responsibility
- determining, planning and formulating government policies, laws, rules and regulations
- investigating matters of concern to the public and electorate, and proposing government action

Occupations in this sub-major group are classified into the following minor group:

111 Chief Executives, General Managers and Legislators

MINOR GROUP 111 CHIEF EXECUTIVES, GENERAL MANAGERS AND LEGISLATORS

CHIEF EXECUTIVES, GENERAL MANAGERS AND LEGISLATORS plan, organise, direct, control and review the overall operations of organisations and their major programs, and represent constituencies in parliaments and local government authorities.

Indicative Skill Level:
In Australia and New Zealand:

Most occupations in this minor group have a level of skill commensurate with a bachelor degree or higher qualification. At least five years of relevant experience may substitute for the formal qualification. In some instances relevant experience and/or on-the-job training may be required in addition to the formal qualification (ANZSCO Skill Level 1).

Tasks Include:
- determining and setting the overall direction and objectives of organisations
- determining and formulating policies, rules and regulations to govern the overall operations of organisations
- directing and controlling the overall operations of organisations to ensure objectives are met
- monitoring and evaluating the overall performance of organisations and adjusting policies, rules and regulations to ensure objectives are met
- representing the organisation at official occasions, in negotiations, at conventions, seminars, public hearings and forums, and liaising between areas of responsibility
- determining, planning and formulating government policies, laws, rules and regulations
- investigating matters of concern to the public and electorate, and proposing government action

Occupations in this minor group are classified into the following unit groups:

1111 Chief Executives and Managing Directors
1112 General Managers
1113 Legislators

UNIT GROUP 1111 CHIEF EXECUTIVES AND MANAGING DIRECTORS

CHIEF EXECUTIVES AND MANAGING DIRECTORS determine, formulate and review the general policy programs and the overall direction of organisations within the framework established by boards of directors and similar governing bodies.

Indicative Skill Level:

In Australia and New Zealand:

Most occupations in this unit group have a level of skill commensurate with a bachelor degree or higher qualification. At least five years of relevant experience may substitute for the formal qualification. In some instances relevant experience and/or on-the-job training may be required in addition to the formal qualification (ANZSCO Skill Level 1).

Tasks Include:

- determining objectives, strategies, policies and programs for organisations
- providing overall direction and management to organisations
- authorising material, human and financial resources to implement organisational policies and programs
- monitoring and evaluating performance of organisations against organisational objectives and strategies
- consulting with senior subordinate staff and reviewing recommendations and reports
- preparing, or arranging for the preparation of, reports, budgets and forecasts and presenting them to governing bodies
- representing the organisation at official occasions, in negotiations, at conventions, seminars, public hearings and forums, and liaising between areas of responsibility
- selecting and approving the selection of senior staff
- ensuring the organisation complies with company laws and other relevant legislation

Occupation:

111111 Chief Executive or Managing Director

111111 CHIEF EXECUTIVE OR MANAGING DIRECTOR

Alternative Title:

Chief Executive Officer

Determines, formulates and reviews the general policy program and the overall direction of an organisation within the framework established by a board of directors or similar governing body.

Skill Level: 1

Specialisations:

Director-General
Executive Director
Secretary (Government Department) (Aus)

UNIT GROUP 1112 GENERAL MANAGERS

GENERAL MANAGERS plan, organise, direct, control and review the day-to-day operations and major functions of commercial, industrial, government and defence organisations through departmental managers and subordinate executives.

Indicative Skill Level:

In Australia and New Zealand:

Most occupations in this unit group have a level of skill commensurate with a bachelor degree or higher qualification. At least five years of relevant experience may substitute for the formal qualification. In some instances relevant experience and/or on-the-job training may be required in addition to the formal qualification (ANZSCO Skill Level 1).

Tasks Include:

- planning policy, and setting standards and objectives for organisations
- providing day-to-day direction and management of organisations, and directing and endorsing policy to fulfil objectives, achieve specific goals, and maximise profit and efficiency
- assessing changing situations and responding accordingly by issuing commands and directives to subordinate staff
- consulting with immediate subordinates and departmental heads on matters such as methods of operation, equipment requirements, finance, sales and human resources
- authorising the funding of major policy implementation programs
- representing the organisation at official occasions, in negotiations, at conventions, seminars, public hearings and forums, and liaising between areas of responsibility
- preparing, or arranging for the preparation of, reports, budgets and forecasts, and presenting them to governing bodies
- selecting and managing the performance of senior staff
- may undertake responsibility for some or all of accounting, sales, marketing, human resources and other specialist operations

Occupations:

111211 Corporate General Manager
111212 Defence Force Senior Officer

111211 CORPORATE GENERAL MANAGER

Alternative Title:

Chief Operating Officer

Plans, organises, directs, controls and reviews the day-to-day operations and major functions of a commercial, industrial, governmental or other organisation through departmental managers and subordinate executives.

Skill Level: 1

Specialisations:

Assistant Commissioner (Police)
Hospital Administrator
Managing Editor
Trade Union Secretary

UNIT GROUP 1112 GENERAL MANAGERS *continued*

111212 DEFENCE FORCE SENIOR OFFICER

Alternative Titles:

Air Force Senior Officer
Army Senior Officer
Navy Senior Officer

Plans, organises, directs, controls and reviews the day-to-day operations and major functions of organisational units of the Australian or New Zealand Defence Forces through subordinate officers.

Skill Level: 1

Specialisations:

Air Chief Marshal (Air Force)
Air Commodore (Air Force)
Air Marshal (Air Force)
Air Vice Marshal (Air Force)
Group Captain (Air Force)
Wing Commander (Air Force)
Brigadier (Army)
Colonel (Army)
General (Army)
Lieutenant Colonel (Army)
Lieutenant General (Army)
Major General (Army)
Admiral (Navy)
Captain (Navy)
Commander (Navy)
Commodore (Navy)
Rear Admiral (Navy)
Vice Admiral (Navy)

UNIT GROUP 1113 LEGISLATORS

LEGISLATORS represent the interests of people in constituencies as elected members of national, state and territory parliaments and local government authorities.

Indicative Skill Level:

In Australia and New Zealand:

Most occupations in this unit group have a level of skill commensurate with a bachelor degree or higher qualification. At least five years of relevant experience may substitute for the formal qualification. In some instances relevant experience and/or on-the-job training may be required in addition to the formal qualification (ANZSCO Skill Level 1).

Tasks Include:

- developing national, state, territory or local government policy, and formulating, amending and repealing legislation and by-laws
- helping constituents with a range of problems particularly with regard to matters concerning government and local agencies
- investigating matters of concern to the public and to particular persons and groups
- introducing proposals for government action and representing public and electoral interests
- issuing policy directions to government departments and exercising control over local government authorities
- attending community functions and meetings of local groups to provide service to the community, gauge public opinion and provide information on government plans
- may serve as a member on parliamentary committees and inquiries
- may present petitions on behalf of concerned groups

Occupations:

111311 Local Government Legislator
111312 Member of Parliament
111399 Legislators nec

111311 LOCAL GOVERNMENT LEGISLATOR

Alternative Titles:

Alderman (Aus)
Councillor

Represents the interests of people in a constituency as their elected member of a local government authority.

Skill Level: 1

Specialisations:

Community Board Member (NZ)
Mayor

UNIT GROUP 1113 LEGISLATORS *continued*

111312 MEMBER OF PARLIAMENT

Alternative Title:
- Parliamentarian

Represents the interests of people in a constituency as their elected member to a national, state or territory parliament.

Skill Level: 1

Specialisations:
- Chief Minister (Aus)
- Government Minister
- Member of the Legislative Assembly (Aus)
- Member of the Legislative Council (Aus)
- Premier (Aus)
- Prime Minister
- Senator (Aus)

111399 LEGISLATORS NEC

This occupation group covers Legislators not elsewhere classified.

Skill Level: 1

Occupations in this group include:
- Aboriginal Community Council Member (Aus)
- Councillor, Aboriginal Land Council (Aus)

SUB-MAJOR GROUP 12 FARMERS AND FARM MANAGERS

FARMERS AND FARM MANAGERS plan, organise, control, coordinate and perform farming operations in agricultural establishments to grow crops, and breed and raise livestock, and fish and other aquatic life.

Indicative Skill Level:
In Australia and New Zealand:

Most occupations in this sub-major group have a level of skill commensurate with a bachelor degree or higher qualification. At least five years of relevant experience may substitute for the formal qualification. In some instances relevant experience and/or on-the-job training may be required in addition to the formal qualification (ANZSCO Skill Level 1).

Tasks Include:

- planning and coordinating the operation of hatcheries and crop production
- breeding and raising livestock and aquatic stock
- monitoring and maintaining the health of stock
- identifying and controlling environmental toxins, weeds, pests and diseases
- organising and conducting farming operations such as maintaining buildings, water supply systems and equipment
- managing business capital, monitoring market activity and planning production to meet contract requirements and market demand

Occupations in this sub-major group are classified into the following minor group:

121 Farmers and Farm Managers

MINOR GROUP 121 FARMERS AND FARM MANAGERS

FARMERS AND FARM MANAGERS plan, organise, control, coordinate and perform farming operations in agricultural establishments to grow crops, and breed and raise livestock, and fish and other aquatic life.

Indicative Skill Level:
In Australia and New Zealand:

Most occupations in this minor group have a level of skill commensurate with a bachelor degree or higher qualification. At least five years of relevant experience may substitute for the formal qualification. In some instances relevant experience and/or on-the-job training may be required in addition to the formal qualification (ANZSCO Skill Level 1).

Tasks Include:

- planning and coordinating the operation of hatcheries and crop production
- breeding and raising livestock and aquatic stock
- monitoring and maintaining the health of stock
- identifying and controlling environmental toxins, weeds, pests and diseases
- organising and conducting farming operations such as maintaining buildings, water supply systems and equipment
- managing business capital, monitoring market activity and planning production to meet contract requirements and market demand

Occupations in this minor group are classified into the following unit groups:

1211 Aquaculture Farmers
1212 Crop Farmers
1213 Livestock Farmers
1214 Mixed Crop and Livestock Farmers

UNIT GROUP 1211 AQUACULTURE FARMERS

AQUACULTURE FARMERS plan, organise, control, coordinate and perform farming operations to breed and raise fish and other aquatic stock.

Indicative Skill Level:

In Australia and New Zealand:

Most occupations in this unit group have a level of skill commensurate with a bachelor degree or higher qualification. At least five years of relevant experience may substitute for the formal qualification. In some instances relevant experience and/or on-the-job training may be required in addition to the formal qualification (ANZSCO Skill Level 1).

Tasks Include:

- planning and coordinating the operation of hatcheries to produce fish fry, seed oysters, crayfish, marron and prawns taking into account environmental and market factors
- monitoring the environment to maintain optimal growing conditions
- identifying and controlling environmental toxins and diseases
- monitoring stock growth rates to determine when to harvest
- transporting fish, crayfish, marron, prawns and sticks of seed oysters to new tanks, ponds, cages and floating net pens
- directing and overseeing the harvesting, grading and packaging of fish, oysters and other aquatic stock
- organising the sale, purchase and transportation of fish stock
- maintaining and evaluating records of farming activities, monitoring market activity and planning production accordingly
- managing business capital including budgeting, taxation, debt and loan management
- may select, train and supervise staff and contractors

Occupation:

121111 Aquaculture Farmer

121111 AQUACULTURE FARMER

Alternative Title:

Seafood Farmer

Plans, organises, controls, coordinates and performs farming operations to breed and raise fish and other aquatic stock.

Skill Level: 1

Specialisations:

Fish Farmer
Hatchery Manager (Fish)
Mussel Farmer
Oyster Farmer

UNIT GROUP 1212 CROP FARMERS

CROP FARMERS plan, organise, control, coordinate and perform farming operations to grow crops.

Indicative Skill Level:

In Australia and New Zealand:

Most occupations in this unit group have a level of skill commensurate with a bachelor degree or higher qualification. At least five years of relevant experience may substitute for the formal qualification. In some instances relevant experience and/or on-the-job training may be required in addition to the formal qualification (ANZSCO Skill Level 1).

Tasks Include:

- planning and coordinating the production and marketing of crops, such as grain, cotton, sugar cane, fruit and nuts, vegetables, turf and flowers, from soil preparation to harvest taking into account environmental and market factors
- selecting and planting seeds, seedlings and bulbs, and grafting new varieties to root stocks
- maintaining crop production by cultivating, de-budding and pruning, and maintaining optimal growing conditions
- organising and conducting farming operations, such as collecting, storing, grading and packaging produce, and organising the sale, purchase and despatch of produce
- directing and overseeing general farming activities such as fertilising and pest and weed control
- maintaining farm buildings, fences, equipment and water supply systems
- maintaining and evaluating records of farming activities, monitoring market activity, and planning crop preparation and production to meet contract requirements and market demand
- managing business capital including budgeting, taxation, debt and loan management
- may select, train and supervise staff and contractors

Occupations:

121211 Cotton Grower
121212 Flower Grower
121213 Fruit or Nut Grower
121214 Grain, Oilseed or Pasture Grower (Aus) / Field Crop Grower (NZ)
121215 Grape Grower
121216 Mixed Crop Farmer
121217 Sugar Cane Grower
121218 Turf Grower
121221 Vegetable Grower (Aus) / Market Gardener (NZ)
121299 Crop Farmers nec

121211 COTTON GROWER

Plans, organises, controls, coordinates and performs farming operations to grow cotton.

Skill Level: 1

UNIT GROUP 1212 CROP FARMERS *continued*

121212 FLOWER GROWER

Alternative Title:
Floriculturist

Plans, organises, controls, coordinates and performs planting, cultivating and harvesting activities to grow flowering and foliage plants.

Skill Level: 1

Specialisation:
Market Gardener (Flowers) (Aus)

121213 FRUIT OR NUT GROWER

Alternative Title:
Fruit or Nut Farm Manager

Plans, organises, controls, coordinates and performs farming operations to grow fruit and nuts.

Skill Level: 1

Specialisation:
Market Gardener (Fruit) (Aus)
Olive Grower
Orchardist

121214 GRAIN, OILSEED OR PASTURE GROWER (AUS) / FIELD CROP GROWER (NZ)

Alternative Title:
Grain, Oilseed or Pasture Farm Manager (Aus)

Plans, organises, controls, coordinates and performs farming operations to grow grain, oilseed, protein and pasture crops.

Skill Level: 1

Specialisations:
Lucerne Farmer
Soybean Grower (Aus)
Wheat Farmer

121215 GRAPE GROWER

Plans, organises, controls, coordinates and performs farming operations to grow table or wine grapes.

Skill Level: 1

Specialisation:
Viticulturist

121216 MIXED CROP FARMER

Plans, organises, controls, coordinates and performs farming operations to grow a variety of crops.

Skill Level: 1

UNIT GROUP 1212 CROP FARMERS *continued*

121217 SUGAR CANE GROWER

Alternative Title:

Sugar Cane Farm Manager

Plans, organises, controls, coordinates and performs farming operations to grow sugar cane.

Skill Level: 1

121218 TURF GROWER

Alternative Title:

Turf Farmer

Plans, organises, controls, coordinates and performs farming operations to grow turf.

Skill Level: 1

121221 VEGETABLE GROWER (AUS) / MARKET GARDENER (NZ)

Alternative Title:

Vegetable Farm Manager

Plans, organises, controls, coordinates and performs farming, greenhouse and market garden operations to grow vegetables.

Skill Level: 1

Specialisation:

Market Gardener (Vegetables) (Aus)

121299 CROP FARMERS NEC

This occupation group covers Crop Farmers not elsewhere classified.

Skill Level: 1

Occupations in this group include:

Coffee Grower (Aus)
Duboisia Farmer (Aus)
Ginger Farmer
Hop Farmer
Tea Tree Farmer
Tobacco Grower
Tree Farmer

UNIT GROUP 1213 LIVESTOCK FARMERS

LIVESTOCK FARMERS plan, organise, control, coordinate and perform farming operations to breed and raise livestock.

Indicative Skill Level:

In Australia and New Zealand:

Most occupations in this unit group have a level of skill commensurate with a bachelor degree or higher qualification. At least five years of relevant experience may substitute for the formal qualification. In some instances relevant experience and/or on-the-job training may be required in addition to the formal qualification (ANZSCO Skill Level 1).

Tasks Include:

- breeding and raising livestock for the production of honey, meat, milk, skins, eggs and wool
- monitoring and maintaining the health and condition of livestock
- providing pastures and fodder to maintain appropriate nutritional levels
- moving livestock to optimise feeding opportunities
- organising and conducting farming operations such as catching, drenching and milking livestock, sterilising machines, and collecting, grading and packaging produce
- directing and overseeing general farming activities such as maintaining pens, sheds and cages, fertilising, controlling pests and weeds, and growing fodder
- maintaining fences, equipment and water supply systems
- organising the sale, purchase and transportation of livestock and produce
- maintaining and evaluating records of farming activities, monitoring market activity and planning production accordingly
- managing business capital including budgeting, taxation, debt and loan management
- may select, train and supervise staff and contractors

Occupations:

121311 Apiarist
121312 Beef Cattle Farmer
121313 Dairy Cattle Farmer
121314 Deer Farmer
121315 Goat Farmer
121316 Horse Breeder
121317 Mixed Livestock Farmer
121318 Pig Farmer
121321 Poultry Farmer
121322 Sheep Farmer
121399 Livestock Farmers nec

121311 APIARIST

Alternative Title:

Beekeeper

Plans, organises, controls, coordinates and operates apiaries to produce honey, queen bee pollen, beeswax and royal jelly, breed queen bees and pollinate crops.

Skill Level: 1

UNIT GROUP 1213 LIVESTOCK FARMERS *continued*

121312 BEEF CATTLE FARMER

Alternative Titles:
Beef Cattle Farm Manager
Beef Cattle Grazier

Plans, organises, controls, coordinates and performs farming operations to breed and raise beef cattle for meat and breeding stock.

Skill Level: 1

Specialisation:
Stud Beef Cattle Farmer

121313 DAIRY CATTLE FARMER

Alternative Title:
Dairy Cattle Farm Manager

Plans, organises, controls, coordinates and performs farming operations to breed and raise dairy cattle for milk, meat and breeding stock.

Skill Level: 1

Specialisations:
Share Dairy Farmer (Aus) / Sharemilker (NZ)
Stud Dairy Cattle Farmer

121314 DEER FARMER

Plans, organises, controls, coordinates and performs farming operations to breed and raise deer for meat, velvet, hides and breeding stock.

Skill Level: 1

121315 GOAT FARMER

Plans, organises, controls, coordinates and performs farming operations to breed and raise goats for fibre, milk, meat and breeding stock.

Skill Level: 1

121316 HORSE BREEDER

Alternative Title:
Horse Stud Manager

Plans, organises, controls, coordinates and performs farming operations to breed and raise horses for competition, dressage, eventing, showjumping, riding for pleasure and working.

Skill Level: 1

Specialisation:
Stud Master/Mistress

UNIT GROUP 1213 LIVESTOCK FARMERS *continued*

121317 MIXED LIVESTOCK FARMER

Plans, organises, controls, coordinates and performs farming operations to breed and raise a variety of livestock.

Skill Level: 1

121318 PIG FARMER

Alternative Title:
Pig Farm Manager

Plans, organises, controls, coordinates and performs farming operations to breed and raise pigs for meat and breeding stock.

Skill Level: 1

Specialisation:
Pig Breeder

121321 POULTRY FARMER

Alternative Title:
Poultry Farm Manager

Plans, organises, controls, coordinates and performs farming operations to breed and raise chickens, turkeys, ducks and other poultry for eggs, meat and breeding stock.

Skill Level: 1

Specialisations:
Chicken Meat Producer
Egg Producer
Hatchery Manager (Poultry)

121322 SHEEP FARMER

Alternative Title:
Sheep Farm Manager

Plans, organises, controls, coordinates and performs farming operations to breed and raise sheep for wool, meat and breeding stock.

Skill Level: 1

Specialisations:
Stud Sheep Farmer
Wool Grower

UNIT GROUP 1213 LIVESTOCK FARMERS *continued*

121399 LIVESTOCK FARMERS NEC

This occupation group covers Livestock Farmers not elsewhere classified.

Skill Level: 1

Occupations in this group include:

- Alpaca Farmer
- Crocodile Farmer (Aus)
- Emu Farmer (Aus)
- Llama Farmer
- Ostrich Farmer

UNIT GROUP 1214 MIXED CROP AND LIVESTOCK FARMERS

MIXED CROP AND LIVESTOCK FARMERS plan, organise, control, coordinate and perform farming operations to both grow crops and to breed and raise livestock.

Indicative Skill Level:

In Australia and New Zealand:

Most occupations in this unit group have a level of skill commensurate with a bachelor degree or higher qualification. At least five years of relevant experience may substitute for the formal qualification. In some instances relevant experience and/or on-the-job training may be required in addition to the formal qualification (ANZSCO Skill Level 1).

Tasks Include:

- planning and coordinating the production and marketing of crops and livestock
- breeding and raising livestock for the production of meat, wool, skins, milk or eggs, and growing grain, seed crops, vegetables, fruit or nuts
- monitoring and maintaining the health and condition of livestock
- providing pastures and fodder to maintain appropriate nutritional levels
- selecting and purchasing seed for planting, planning and implementing breeding programs
- directing and overseeing general farming activities such as fertilising, and pest and weed control
- maintaining farm buildings, fences, equipment and water supply systems
- organising the sale, purchase and transportation of livestock and produce
- maintaining and evaluating records of farming activities, monitoring market activity and planning production accordingly
- managing business capital, monitoring market activity and planning production accordingly
- may select, train and supervise staff and contractors

Occupation:

121411 Mixed Crop and Livestock Farmer

121411 MIXED CROP AND LIVESTOCK FARMER

Alternative Title:

Farmer and Grazier

Plans, organises, controls, coordinates and performs farming operations to both grow crops and to breed and raise livestock.

Skill Level: 1

Specialisations:

Cattle and Wheat Farmer
Sheep and Wheat Farmer

SUB-MAJOR GROUP 13 SPECIALIST MANAGERS

SPECIALIST MANAGERS plan, organise, direct, control and coordinate special functions within organisations such as advertising and sales, financial, human resources, production and distribution, education, health and welfare, and ICT.

Indicative Skill Level:

In Australia and New Zealand:

Most occupations in this sub-major group have a level of skill commensurate with a bachelor degree or higher qualification. At least five years of relevant experience may substitute for the formal qualification. In some instances relevant experience and/or on-the-job training may be required in addition to the formal qualification (ANZSCO Skill Level 1).

Tasks Include:

- developing, implementing and monitoring strategies, policies and plans for their area of control
- establishing and directing operational and administrative procedures
- directing and coordinating the allocation of resources
- monitoring work progress and performance, and adjusting processes and resources to keep goals on track
- controlling budget planning and report preparation, and monitoring and controlling expenditure for their area of control
- controlling selection, training and performance of staff
- representing the organisation in negotiations, and at conventions, seminars, public hearings and forums

Occupations in this sub-major group are classified into the following minor groups:

131 Advertising and Sales Managers
132 Business Administration Managers
133 Construction, Distribution and Production Managers
134 Education, Health and Welfare Services Managers
135 ICT Managers
139 Miscellaneous Specialist Managers

MINOR GROUP 131 ADVERTISING AND SALES MANAGERS

ADVERTISING AND SALES MANAGERS plan, organise, direct, control and coordinate advertising, public relations, sales and marketing activities within organisations.

Indicative Skill Level:
In Australia and New Zealand:

Most occupations in this minor group have a level of skill commensurate with a bachelor degree or higher qualification. At least five years of relevant experience may substitute for the formal qualification. In some instances relevant experience and/or on-the-job training may be required in addition to the formal qualification (ANZSCO Skill Level 1).

Tasks Include:

- formulating and implementing policies and plans for advertising, public relations, sales and marketing in consultation with other Managers
- directing the development of initiatives for new products, marketing and advertising campaigns
- organising and controlling sales activities by setting product mix, geographical sales areas and customer service standards
- directing merchandising methods and distribution policy by coordinating the work of salespersons, and organising agents and distributors
- directing sales methods and arrangements by setting prices and credit arrangements

Occupations in this minor group are classified into the following unit group:

1311 Advertising and Sales Managers

UNIT GROUP 1311 ADVERTISING AND SALES MANAGERS

ADVERTISING AND SALES MANAGERS plan, organise, direct, control and coordinate advertising, public relations, sales and marketing activities within organisations.

Indicative Skill Level:

In Australia and New Zealand:

Most occupations in this unit group have a level of skill commensurate with a bachelor degree or higher qualification. At least five years of relevant experience may substitute for the formal qualification. In some instances relevant experience and/or on-the-job training may be required in addition to the formal qualification (ANZSCO Skill Level 1).

Tasks Include:

- formulating and implementing policies and plans for advertising, public relations, sales and marketing in consultation with other Managers
- directing the development of initiatives for new products, marketing and advertising campaigns
- organising and controlling sales activities by setting product mix, geographical sales areas and customer service standards
- directing merchandising methods and distribution policy by coordinating the work of salespersons, and organising agents and distributors
- directing sales methods and arrangements by setting prices and credit arrangements

Occupations:

131111 Advertising and Public Relations Manager
131112 Sales and Marketing Manager

131111 ADVERTISING AND PUBLIC RELATIONS MANAGER

Plans, organises, directs, controls and coordinates the advertising and public relations activities within an organisation.

Skill Level: 1

Specialisations:

Community Relations Manager
Corporate Relations Manager
Public Affairs Manager
Relationship Manager

131112 SALES AND MARKETING MANAGER

Plans, organises, directs, controls and coordinates the sales and marketing activities within an organisation.

Skill Level: 1

ICT Business Development Managers are excluded from this occupation. ICT Business Development Managers are included in Unit Group 2252 ICT Sales Professionals, in Occupation 225212 ICT Business Development Manager.

Specialisations:

Business Development Manager
Market Research Manager

MINOR GROUP 132 BUSINESS ADMINISTRATION MANAGERS

BUSINESS ADMINISTRATION MANAGERS plan, organise, direct, control and coordinate the corporate, financial, human resource, policy, planning, research and development activities and guidelines within organisations.

Indicative Skill Level:

In Australia and New Zealand:

Most occupations in this minor group have a level of skill commensurate with a bachelor degree or higher qualification. At least five years of relevant experience may substitute for the formal qualification. In some instances relevant experience and/or on-the-job training may be required in addition to the formal qualification (ANZSCO Skill Level 1).

Tasks Include:

- formulating and administering policy advice and strategic planning
- establishing and directing operational and administrative procedures
- implementing, monitoring and evaluating budgetary and accounting strategies and policies
- providing advice to senior Managers and board members on strategic, policy and program and legislative issues
- ensuring compliance with relevant legislation, regulations and standards
- controlling selection, training and performance of staff
- representing the organisation in negotiations, and at conventions, seminars, public hearings and forums

Occupations in this minor group are classified into the following unit groups:

1321 Corporate Services Managers
1322 Finance Managers
1323 Human Resource Managers
1324 Policy and Planning Managers
1325 Research and Development Managers

UNIT GROUP 1321 CORPORATE SERVICES MANAGERS

CORPORATE SERVICES MANAGERS plan, organise, direct, control and coordinate the overall administration of organisations.

Indicative Skill Level:

In Australia and New Zealand:

Most occupations in this unit group have a level of skill commensurate with a bachelor degree or higher qualification. At least five years of relevant experience may substitute for the formal qualification. In some instances relevant experience and/or on-the-job training may be required in addition to the formal qualification (ANZSCO Skill Level 1).

Tasks Include:

- providing high level administrative, strategic planning and operational support, research and advice to senior management on administrative matters such as staff management, financial planning, facility management and information services
- developing and managing the organisation's administrative, financial, physical and staff resources
- developing and implementing administrative, financial and operational procedural statements and guidelines for use by staff in the organisation
- analysing complex resource management issues and initiatives that affect the organisation, and preparing associated reports, correspondence and submissions
- providing information and support for the preparation of financial reports and budgets
- leading, managing and developing administrative staff to ensure smooth business operations and the provision of accurate and timely information
- representing the organisation in negotiations, and at conventions, seminars, public hearings and forums, and promoting existing and new programs and policies

Occupation:

132111 Corporate Services Manager

132111 CORPORATE SERVICES MANAGER

Alternative Titles:

Administration Manager

Business Services Manager

Plans, organises, directs, controls and coordinates the overall administration of an organisation.

Skill Level: 1

UNIT GROUP 1322 FINANCE MANAGERS

FINANCE MANAGERS plan, organise, direct, control and coordinate the financial and accounting activities within organisations.

Indicative Skill Level:

In Australia and New Zealand:

Most occupations in this unit group have a level of skill commensurate with a bachelor degree or higher qualification. At least five years of relevant experience may substitute for the formal qualification. In some instances relevant experience and/or on-the-job training may be required in addition to the formal qualification (ANZSCO Skill Level 1).

Tasks Include:

- determining, implementing, monitoring, reviewing and evaluating budgetary and accounting strategies, policies and plans in consultation with other Managers
- providing financial information and interpreting the implications for business performance and funding needs
- coordinating the development, implementation and monitoring of accounting systems
- directing the preparation of financial reports summarising and forecasting the organisation's financial position such as income statements, balance sheets and analyses of future earnings and income
- assessing capital finance proposals and the financial status of operational projects
- advising on investment strategies, sources of funds and the distribution of earnings
- delivering long range profit forecasts, budgeting and financial reporting
- ensuring compliance with financial legislation and standards

Occupation:

132211 Finance Manager

132211 FINANCE MANAGER

Alternative Titles:

Chief Financial Officer
Finance Director
Financial Controller

Plans, organises, directs, controls and coordinates the financial and accounting activities within an organisation.

Skill Level: 1

UNIT GROUP 1323 HUMAN RESOURCE MANAGERS

HUMAN RESOURCE MANAGERS plan, organise, direct, control and coordinate the human resource and workplace relations activities within organisations.

Indicative Skill Level:

In Australia and New Zealand:

Most occupations in this unit group have a level of skill commensurate with a bachelor degree or higher qualification. At least five years of relevant experience may substitute for the formal qualification. In some instances relevant experience and/or on-the-job training may be required in addition to the formal qualification (ANZSCO Skill Level 1).

Tasks Include:

- determining, implementing, monitoring, reviewing and evaluating human resource management strategies, policies and plans to meet business needs
- advising and assisting other Managers in applying sound recruitment and selection practices, and appropriate induction, training and development programs
- developing and implementing performance management systems to plan, appraise and improve individual and team performance
- representing the organisation in negotiations with unions and employees to determine remuneration and other conditions of employment
- developing and implementing occupational health and safety programs and equal employment opportunity programs, and ensuring compliance with related statutory requirements
- overseeing the application of redundancy and other employee retrenchment policies
- monitoring employment costs and productivity levels
- may train and advise other Managers in personnel and workplace relations matters

Occupation:

132311 Human Resource Manager

132311 HUMAN RESOURCE MANAGER

Alternative Title:

Personnel and Employee Relations Manager

Plans, organises, directs, controls and coordinates the human resource and workplace relations activities within an organisation.

Skill Level: 1

Specialisations:

Occupational Health and Safety Manager
Training and Development Manager
Workplace Relations Manager

UNIT GROUP 1324 POLICY AND PLANNING MANAGERS

POLICY AND PLANNING MANAGERS plan, organise, direct, control and coordinate policy advice and strategic planning within organisations.

Indicative Skill Level:

In Australia and New Zealand:

Most occupations in this unit group have a level of skill commensurate with a bachelor degree or higher qualification. At least five years of relevant experience may substitute for the formal qualification. In some instances relevant experience and/or on-the-job training may be required in addition to the formal qualification (ANZSCO Skill Level 1).

Tasks Include:

- developing, implementing and monitoring strategic plans, programs, policies, processes, systems and procedures to achieve goals, objectives and work standards
- developing, implementing, administering and participating in policy research and analysis
- coordinating the implementation of policies and practices
- establishing activity measures and measurements of accountability
- overseeing and participating in the development of policy documents and reports
- consulting with and providing expert advice to government officials and board members on policy, program and legislative issues
- representing the organisation in negotiations, and at conventions, seminars, public hearings and forums convened to discuss policy issues

Occupation:

132411 Policy and Planning Manager

132411 POLICY AND PLANNING MANAGER

Alternative Title:

Public Policy Manager

Plans, organises, directs, controls and coordinates policy advice and strategic planning within an organisation.

Skill Level: 1

Specialisations:

Corporate Planning Manager
Policy Development Manager
Strategic Planning Manager

UNIT GROUP 1325 RESEARCH AND DEVELOPMENT MANAGERS

RESEARCH AND DEVELOPMENT MANAGERS plan, organise, direct, control and coordinate research and development activities within organisations.

Indicative Skill Level:

In Australia and New Zealand:

Most occupations in this unit group have a level of skill commensurate with a bachelor degree or higher qualification. At least five years of relevant experience may substitute for the formal qualification. In some instances relevant experience and/or on-the-job training may be required in addition to the formal qualification (ANZSCO Skill Level 1).

Tasks Include:

- determining, implementing and monitoring research and development strategies, policies and plans
- developing and implementing research projects, priorities and targets to support commercial and policy developments
- leading major research projects and coordinating activities of other research workers
- assessing the benefits and monitoring the costs and effectiveness of research and development activities
- interpreting results of research projects and recommending associated product and service development innovations
- providing advice on research and development options available to the organisation
- monitoring leading-edge developments in relevant disciplines and assessing implications for the organisation
- may publish results of significant research projects

Occupation:

132511 Research and Development Manager

132511 RESEARCH AND DEVELOPMENT MANAGER

Plans, organises, directs, controls and coordinates research and development activities within an organisation.

Skill Level: 1

MINOR GROUP 133 CONSTRUCTION, DISTRIBUTION AND PRODUCTION MANAGERS

CONSTRUCTION, DISTRIBUTION AND PRODUCTION MANAGERS plan, organise, direct, control and coordinate building and construction, engineering, importing, exporting and wholesaling, manufacturing, production, supply and distribution activities within organisations.

Indicative Skill Level:

In Australia and New Zealand:

Most occupations in this minor group have a level of skill commensurate with a bachelor degree or higher qualification. At least five years of relevant experience may substitute for the formal qualification. In some instances relevant experience and/or on-the-job training may be required in addition to the formal qualification (ANZSCO Skill Level 1).

Tasks Include:

- identifying business opportunities, and developing and implementing business plans and marketing, operating, human resource, pricing and credit policies and procedures
- establishing project schedules and budgets
- coordinating labour resources and procurement and delivery of materials, plant and equipment
- negotiating contracts with suppliers and customers to meet resource, cost and quality requirements
- monitoring costs and work progress and performance, and adjusting processes and resources to ensure goals are met
- directing the preparation of production records and reports

Occupations in this minor group are classified into the following unit groups:

1331 Construction Managers
1332 Engineering Managers
1333 Importers, Exporters and Wholesalers
1334 Manufacturers
1335 Production Managers
1336 Supply and Distribution Managers

UNIT GROUP 1331 CONSTRUCTION MANAGERS

CONSTRUCTION MANAGERS plan, organise, direct, control and coordinate the construction of civil engineering projects, buildings and dwellings, and the physical and human resources involved in building and construction.

Indicative Skill Level:

In Australia and New Zealand:

Most occupations in this unit group have a level of skill commensurate with a bachelor degree or higher qualification. At least five years of relevant experience may substitute for the formal qualification. In some instances relevant experience and/or on-the-job training may be required in addition to the formal qualification (ANZSCO Skill Level 1).

Registration or licensing is required.

Tasks Include:

- interpreting architectural drawings and specifications
- coordinating labour resources, and procurement and delivery of materials, plant and equipment
- consulting with Architects, Engineering Professionals and other professionals, and Technical and Trades Workers
- negotiating with building owners, property developers and subcontractors involved in the construction process to ensure projects are completed on time and within budget
- preparing tenders and contract bids
- operating and implementing coordinated work programs for sites
- ensuring adherence to building legislation and standards of performance, quality, cost and safety
- arranging submission of plans to local authorities
- building under contract, or subcontracting specialised building services
- overseeing the standard and progress of subcontractors' work
- arranging building inspections by local authorities

Occupations:

133111 Construction Project Manager
133112 Project Builder

133111 CONSTRUCTION PROJECT MANAGER

Alternative Title:

Building and Construction Manager

Plans, organises, directs, controls and coordinates construction of civil engineering and building projects, and the physical and human resources involved in the construction process. Registration or licensing is required.

Skill Level: 1

133112 PROJECT BUILDER

Alternative Title:

Professional Builder

Plans, organises, directs, controls and coordinates the construction, alteration and renovation of dwellings and other buildings, and the physical and human resources involved in the building process. Registration or licensing is required.

Skill Level: 1

UNIT GROUP 1332 ENGINEERING MANAGERS

ENGINEERING MANAGERS plan, organise, direct, control and coordinate the engineering and technical operations of organisations.

Indicative Skill Level:

In Australia and New Zealand:

Most occupations in this unit group have a level of skill commensurate with a bachelor degree or higher qualification. At least five years of relevant experience may substitute for the formal qualification. In some instances relevant experience and/or on-the-job training may be required in addition to the formal qualification (ANZSCO Skill Level 1).

Tasks Include:

- determining, implementing and monitoring engineering strategies, policies and plans
- interpreting plans, drawings and specifications, and providing advice on engineering methods and procedures to achieve construction and production requirements
- establishing project schedules and budgets
- ensuring conformity with specifications and plans, and with laws, regulations and safety standards
- ensuring engineering standards of quality, cost, safety, timeliness and performance are observed
- overseeing maintenance requirements to optimise efficiency
- liaising with marketing, research and manufacturing managers regarding engineering aspects of new construction and product design
- may contribute to research and development projects

Occupation:

133211 Engineering Manager

133211 ENGINEERING MANAGER

Plans, organises, directs, controls and coordinates the engineering and technical operations of an organisation.

Skill Level: 1

UNIT GROUP 1333 IMPORTERS, EXPORTERS AND WHOLESALERS

IMPORTERS, EXPORTERS AND WHOLESALERS plan, organise, direct, control and coordinate the operations of importing, exporting and wholesaling establishments.

Indicative Skill Level:

In Australia and New Zealand:

Most occupations in this unit group have a level of skill commensurate with a bachelor degree or higher qualification. At least five years of relevant experience may substitute for the formal qualification. In some instances relevant experience and/or on-the-job training may be required in addition to the formal qualification (ANZSCO Skill Level 1).

Tasks Include:

- identifying local and overseas business opportunities
- developing and implementing business plans, and marketing, operating, human resource, pricing and credit policies and procedures
- determining the mix of products and services to be provided and negotiating conditions of trade
- liaising with local and overseas suppliers and distributors about orders and products
- researching regulatory and statutory requirements affecting the importing, exporting, wholesaling and distribution of goods
- monitoring business performance and preparing estimates, financial statements and reports of operations
- appointing agents and distributors
- arranging the shipping of goods into and out of the country
- overseeing the display and sale of merchandise and preparation of product information for customer service staff and customers
- implementing after-sales service procedures

Occupations:

133311 Importer or Exporter
133312 Wholesaler

133311 IMPORTER OR EXPORTER

Plans, organises, directs, controls and coordinates the operations of an importing or exporting establishment.

Skill Level: 1

133312 WHOLESALER

Plans, organises, directs, controls and coordinates the operations of a wholesale trading establishment.

Skill Level: 1

UNIT GROUP 1334 MANUFACTURERS

MANUFACTURERS plan, organise, direct, control and coordinate the operations of small manufacturing establishments.

Indicative Skill Level:

In Australia and New Zealand:

Most occupations in this unit group have a level of skill commensurate with a bachelor degree or higher qualification. At least five years of relevant experience may substitute for the formal qualification. In some instances relevant experience and/or on-the-job training may be required in addition to the formal qualification (ANZSCO Skill Level 1).

Tasks Include:

- identifying business opportunities, devising new manufacturing processes and determining products to be manufactured
- developing business plans and implementing operational, marketing, human resource and pricing procedures
- researching and implementing regulatory and statutory requirements affecting manufacturing operations and the environment
- directing the activities of production, warehouse, distribution and other operating units
- maintaining quality control systems for manufacturing, waste disposal, delivery and other procedures
- coordinating orders for raw materials, supplies and equipment, and arranging packaging, delivery and wholesaling of products
- overseeing the coordination of after-sales service
- overseeing the provision of quotes for the manufacture of specialised goods and arranging contracts with customers
- may devise and oversee the implementation of production run schedules

Occupation:

133411 Manufacturer

133411 MANUFACTURER

Plans, organises, directs, controls and coordinates the operations of a small manufacturing establishment.

Skill Level: 1

UNIT GROUP 1335 PRODUCTION MANAGERS

PRODUCTION MANAGERS plan, organise, direct, control and coordinate the production activities of forestry, manufacturing and mining organisations including physical and human resources.

Indicative Skill Level:

In Australia and New Zealand:

Most occupations in this unit group have a level of skill commensurate with a bachelor degree or higher qualification. At least five years of relevant experience may substitute for the formal qualification. In some instances relevant experience and/or on-the-job training may be required in addition to the formal qualification (ANZSCO Skill Level 1).

Tasks Include:

- determining, implementing and monitoring production strategies, policies and plans
- planning details of production activities in terms of output quality and quantity, cost, time available and labour requirements
- controlling the operation of production plant and quality procedures through planning of maintenance, designation of operating hours and supply of parts and tools
- monitoring production output and costs, and adjusting processes and resources to minimise costs
- informing other Managers about production matters
- overseeing the acquisition and installation of new plant and equipment
- directing research into production methods, and recommending and implementing initiatives
- controlling the preparation of production records and reports
- coordinating the implementation of occupational health and safety requirements
- directing staff activities and monitoring their performance

Occupations:

133511 Production Manager (Forestry)
133512 Production Manager (Manufacturing)
133513 Production Manager (Mining)

133511 PRODUCTION MANAGER (FORESTRY)

Alternative Title:

Forest Manager

Plans, organises, directs, controls and coordinates the production activities of a forestry operation including physical and human resources.

Skill Level: 1

Specialisations:

Forest Logistics Manager (NZ)
Harvest Manager (Forestry)
Operations Manager (Forestry)

UNIT GROUP 1335 PRODUCTION MANAGERS *continued*

133512 PRODUCTION MANAGER (MANUFACTURING)

Plans, organises, directs, controls and coordinates the manufacturing activities of an organisation including physical and human resources.

Skill Level: 1

Specialisations:

- Operations Manager (Production)
- Plant Manager (Manufacturing) (Aus)
- Works Manager (Manufacturing) (Aus)

133513 PRODUCTION MANAGER (MINING)

Alternative Titles:

- Mine Manager
- Mine Superintendent

Plans, organises, directs, controls and coordinates the production activities of a mining operation including physical and human resources.

Skill Level: 1

Specialisation:

- Quarry Manager

UNIT GROUP 1336 SUPPLY AND DISTRIBUTION MANAGERS

SUPPLY AND DISTRIBUTION MANAGERS plan, organise, direct, control and coordinate the supply, storage and distribution of goods produced by organisations.

Indicative Skill Level:

In Australia and New Zealand:

Most occupations in this unit group have a level of skill commensurate with a bachelor degree or higher qualification. At least five years of relevant experience may substitute for the formal qualification. In some instances relevant experience and/or on-the-job training may be required in addition to the formal qualification (ANZSCO Skill Level 1).

Tasks Include:

- determining, implementing and monitoring purchasing, storage and distribution strategies, policies and plans
- preparing and implementing plans to maintain required stock levels at minimum cost
- negotiating contracts with suppliers to meet quality, cost and delivery requirements
- monitoring and reviewing storage and inventory systems to meet supply requirements and control stock levels
- operating recording systems to track all movements of supplies and finished goods, and ensuring re-ordering and re-stocking at optimal times
- liaising with other departments and customers concerning requirements for outward goods and associated forwarding transportation
- overseeing the recording of purchase, storage and distribution transactions
- directing staff activities and monitoring their performance

Occupation:

133611 Supply and Distribution Manager

133611 SUPPLY AND DISTRIBUTION MANAGER

Plans, organises, directs, controls and coordinates the supply, storage and distribution of goods produced by an organisation.

Skill Level: 1

Specialisations:

Logistics Manager
Logistics Officer (Air Force)
Ordnance Corps Officer (Army)
Supply Chain Manager
Supply Officer (Navy)
Transport Corps Officer (Army)

MINOR GROUP 134 EDUCATION, HEALTH AND WELFARE SERVICES MANAGERS

EDUCATION, HEALTH AND WELFARE SERVICES MANAGERS plan, organise, direct, control and coordinate the provision of childcare, health, welfare and education services.

Indicative Skill Level:

In Australia and New Zealand:

Most occupations in this minor group have a level of skill commensurate with a bachelor degree or higher qualification. At least five years of relevant experience may substitute for the formal qualification. In some instances relevant experience and/or on-the-job training may be required in addition to the formal qualification (ANZSCO Skill Level 1).

Tasks Include:

- formulating policies and plans for the operation of childcare, health, welfare and educational establishments, and setting standards and objectives
- developing and implementing programs and services to meet the needs of clients
- directing and coordinating the allocation of resources
- liaising with parents, boards, funding bodies, the community and educational institutions to discuss areas of cooperation and coordination
- directing administrative operations such as budget planning, report preparation, monitoring and controlling expenditure, and student admissions
- selecting, developing and managing the performance of staff
- preparing, or arranging for the preparation of, reports, budgets and forecasts
- representing the organisation in negotiations, and at conventions, seminars, public hearings and forums

Occupations in this minor group are classified into the following unit groups:

1341 Child Care Centre Managers
1342 Health and Welfare Services Managers
1343 School Principals
1344 Other Education Managers

UNIT GROUP 1341 CHILD CARE CENTRE MANAGERS

CHILD CARE CENTRE MANAGERS plan, organise, direct, control and coordinate the activities of childcare centres and services including physical and human resources.

Indicative Skill Level:

In Australia and New Zealand:

Most occupations in this unit group have a level of skill commensurate with a bachelor degree or higher qualification. At least five years of relevant experience may substitute for the formal qualification. In some instances relevant experience and/or on-the-job training may be required in addition to the formal qualification (ANZSCO Skill Level 1).

Tasks Include:

- developing and implementing programs to enhance the physical, social, emotional and intellectual development of young children
- providing care for children in before-school, after-school, day, and vacation care centres
- directing and supervising Child Carers in providing care and supervision for young children
- ensuring the centre is a safe area for children, staff and visitors
- complying with relevant government requirements and standards
- liaising with parents
- maintaining records and accounts for the centre
- recruiting staff and coordinating professional development

Occupation:

134111 Child Care Centre Manager

134111 CHILD CARE CENTRE MANAGER

Alternative Titles:

Child Care Centre Director
Child Care Coordinator

Plans, organises, directs, controls and coordinates the activities of a childcare centre or service including physical and human resources.

Skill Level: 1

UNIT GROUP 1342 HEALTH AND WELFARE SERVICES MANAGERS

HEALTH AND WELFARE SERVICES MANAGERS plan, organise, direct, control and coordinate the professional and administrative aspects of health and welfare programs and services.

Nurse Managers are excluded from this unit group. Nurse Managers are included in Unit Group 2543 Nurse Managers.

Indicative Skill Level:

In Australia and New Zealand:

Most occupations in this unit group have a level of skill commensurate with a bachelor degree or higher qualification and at least five years of relevant experience. In some instances relevant experience may substitute for the formal qualification (ANZSCO Skill Level 1).

Registration or licensing may be required.

Tasks Include:

- providing overall direction and management for the service, facility, organisation or centre
- developing, implementing and monitoring procedures, policies and standards for medical, nursing, allied health and administrative staff
- coordinating and administering health and welfare programs and clinical services
- monitoring and evaluating resources devoted to health, welfare, recreation, housing, employment, training and other community facilities and centres
- controlling administrative operations such as budget planning, report preparation, expenditure on supplies, equipment and services
- liaising with other health and welfare providers, boards and funding bodies to discuss areas of health and welfare service cooperation and coordination
- advising government bodies about measures to improve health and welfare services and facilities
- representing the organisation in negotiations, and at conventions, seminars, public hearings and forums
- controlling selection, training and supervision of staff

Occupations:

134211 Medical Administrator (Aus) / Medical Superintendent (NZ)
134212 Nursing Clinical Director
134213 Primary Health Organisation Manager
134214 Welfare Centre Manager
134299 Health and Welfare Services Managers nec

134211 MEDICAL ADMINISTRATOR (AUS) / MEDICAL SUPERINTENDENT (NZ)

Alternative Title:

Medical Manager

Plans, organises, directs, controls and coordinates medical programs and clinical services in a hospital or other health service facility, maintains standards of medical care, provides leadership to ensure an appropriately skilled medical workforce, and contributes to health service planning.

Skill Level: 1

Specialisations:

Director of Clinical Services
Director of Medical Services

UNIT GROUP 1342 HEALTH AND WELFARE SERVICES MANAGERS *continued*

134212 NURSING CLINICAL DIRECTOR

Alternative Titles:
- Director of Nursing
- Senior Nurse Manager

Plans, organises, directs, controls and coordinates nursing programs and clinical services in a hospital, aged care or other health service facility, maintains standards of nursing care, provides leadership to ensure an appropriately skilled nursing and midwifery workforce, and contributes to health service planning. Registration or licensing may be required.

Skill Level: 1

Specialisations:
- Assistant Director of Nursing
- Deputy Director of Nursing
- Executive Director of Nursing

134213 PRIMARY HEALTH ORGANISATION MANAGER

Plans, organises, directs, controls and coordinates a primary health organisation that provides a broad range of out-of-hospital health services.

Skill Level: 1

134214 WELFARE CENTRE MANAGER

Alternative Title:
- Welfare Project Manager

Plans, organises, directs, controls and coordinates a centre, program or project concerned with social welfare support.

Skill Level: 1

134299 HEALTH AND WELFARE SERVICES MANAGERS NEC

This occupation group covers Health and Welfare Services Managers not elsewhere classified.

Skill Level: 1

Occupations in this group include:
- Director of Pharmacy
- Director of Physiotherapy Services
- Director of Speech Pathology
- Manager of Allied Health Services
- Medical Corps Officer (Army)

UNIT GROUP 1343 SCHOOL PRINCIPALS

SCHOOL PRINCIPALS plan, organise, direct, control and coordinate the educational and administrative aspects of primary, middle or intermediate, and secondary schools including physical and human resources.

Indicative Skill Level:

In Australia and New Zealand:

Most occupations in this unit group have a level of skill commensurate with a bachelor degree or higher qualification and at least five years of relevant experience (ANZSCO Skill Level 1).

Registration or licensing is required.

Tasks Include:

- determining educational programs based on curricula set by educational authorities
- implementing systems and procedures to monitor school performance and student enrolments
- directing administrative and clerical activities concerning student admissions and educational services
- preparing school budgets and maintaining budgetary controls
- promoting the educational program, and representing schools in the wider community
- supervising the maintenance of school facilities
- enforcing a disciplinary code to create a safe and conducive environment for students and teachers
- organising and implementing methods of raising additional funds in conjunction with parent groups
- controlling selection, training and supervision of staff
- may teach students

Occupation:

134311 School Principal

134311 SCHOOL PRINCIPAL

Alternative Title:

Headmaster/mistress

Plans, organises, directs, controls and coordinates the educational and administrative aspects of primary, middle or intermediate and secondary schools including physical and human resources. Registration or licensing is required.

Skill Level: 1

Specialisations:

Middle or Intermediate School Principal
Primary School Principal
Secondary School Principal

UNIT GROUP 1344 OTHER EDUCATION MANAGERS

OTHER EDUCATION MANAGERS plan, organise, direct, control and coordinate educational policy, and provide advice and educational and administrative support to staff and students in educational institutions.

Indicative Skill Level:

In Australia and New Zealand:

Most occupations in this unit group have a level of skill commensurate with a bachelor degree or higher qualification and at least five years of relevant experience. In some instances relevant experience may substitute for the formal qualification (ANZSCO Skill Level 1).

Tasks Include:

- coordinating the educational, administrative and financial affairs of an educational institution or department within the institution
- researching, developing, implementing, reviewing and evaluating educational and administrative policy
- liaising between educational institutions, parents and the wider community
- providing advice on policy and procedures to staff and students
- consulting with academic and administrative staff to coordinate educational programs
- identifying and addressing present and future needs for student and staff development
- researching educational systems and monitoring and evaluating new developments
- researching and reporting on students' needs arising from curriculum implementation
- developing and delivering training programs for teachers

Occupations:

134411 Faculty Head
134412 Regional Education Manager
134499 Education Managers nec

134411 FACULTY HEAD

Plans, organises, directs, controls and coordinates the educational and administrative aspects of a department, faculty or school within a university or other tertiary education institution.

Skill Level: 1

Specialisations:

Dean (University)
Head Teacher (TAFE) (Aus)

134412 REGIONAL EDUCATION MANAGER

Plans, organises, directs, controls and coordinates educational approaches and policy, and curriculum resources and development for preschool, primary, middle or intermediate school, secondary, TAFE or polytechnic teachers and administrators.

Skill Level: 1

Specialisation:

Schools Director

UNIT GROUP 1344 OTHER EDUCATION MANAGERS *continued*

134499 EDUCATION MANAGERS NEC

This occupation group covers Education Managers not elsewhere classified.

Skill Level: 1

Occupations in this group include:

- Polytechnic Registrar (NZ)
- Project Coordinator (Education)
- TAFE Registrar (Aus)
- University Registrar

MINOR GROUP 135 ICT MANAGERS

ICT MANAGERS plan, organise, direct, control and coordinate the acquisition, development, maintenance and use of computer and telecommunication systems within organisations.

Indicative Skill Level:

In Australia and New Zealand:

Most occupations in this minor group have a level of skill commensurate with a bachelor degree or higher qualification. At least five years of relevant experience may substitute for the formal qualification. In some instances relevant experience and/or on-the-job training may be required in addition to the formal qualification (ANZSCO Skill Level 1).

Tasks Include:

- analysing information needs and specifying technology to meet those needs
- formulating and directing information and communication technology (ICT) strategies, policies and plans
- directing the selection and installation of ICT resources and the provision of user training
- directing ICT operations and setting priorities between system developments, maintenance and operations
- overseeing the security of ICT systems

Occupations in this minor group are classified into the following unit group:

1351 ICT Managers

UNIT GROUP 1351 ICT MANAGERS

ICT MANAGERS plan, organise, direct, control and coordinate the acquisition, development, maintenance and use of computer and telecommunication systems within organisations.

Indicative Skill Level:

In Australia and New Zealand:

Most occupations in this unit group have a level of skill commensurate with a bachelor degree or higher qualification. At least five years of relevant experience may substitute for the formal qualification. In some instances relevant experience and/or on-the-job training may be required in addition to the formal qualification (ANZSCO Skill Level 1).

Tasks Include:

- analysing information needs and specifying technology to meet those needs
- formulating and directing information and communication technology (ICT) strategies, policies and plans
- directing the selection and installation of ICT resources and the provision of user training
- directing ICT operations and setting priorities between system developments, maintenance and operations
- overseeing the security of ICT systems

Occupations:

135111 Chief Information Officer
135112 ICT Project Manager
135199 ICT Managers nec

135111 CHIEF INFORMATION OFFICER

Alternative Title:

Chief Technology Officer

Plans, organises, directs, controls and coordinates the ICT strategies, plans and operations of an organisation to ensure the ICT infrastructure supports the organisation's overall operations and priorities.

Skill Level: 1

135112 ICT PROJECT MANAGER

Plans, organises, directs, controls and coordinates quality accredited ICT projects. Accountable for day-to-day operations of resourcing, scheduling, prioritisation and task coordination, and meeting project milestones, objectives and deliverables within agreed timeframes and budgets.

Skill Level: 1

Specialisation:

ICT Development Manager

135199 ICT MANAGERS NEC

This occupation group covers ICT Managers not elsewhere classified.

Skill Level: 1

Occupations in this group include:

Internet Service Provider
Network Manager

MINOR GROUP 139 MISCELLANEOUS SPECIALIST MANAGERS

This minor group covers Specialist Managers not elsewhere classified.

It includes Commissioned Officers (Management), and Senior Non-commissioned Defence Force Members.

Indicative Skill Level:
In Australia and New Zealand:

Most occupations in this minor group have a level of skill commensurate with a bachelor degree or higher qualification. At least five years of relevant experience may substitute for the formal qualification. In some instances relevant experience and/or on-the-job training may be required in addition to the formal qualification (ANZSCO Skill Level 1).

Occupations in this minor group are classified into the following unit groups:

1391 Commissioned Officers (Management)
1392 Senior Non-commissioned Defence Force Members
1399 Other Specialist Managers

UNIT GROUP 1391 COMMISSIONED OFFICERS (MANAGEMENT)

COMMISSIONED OFFICERS (MANAGEMENT) provide high level management to support the running of organisational, geographical and operational units and sections within the defence forces and fire and police services.

Indicative Skill Level:

In Australia and New Zealand:

Most occupations in this unit group have a level of skill commensurate with a bachelor degree or higher qualification. At least five years of relevant experience may substitute for the formal qualification. In some instances relevant experience and/or on-the-job training may be required in addition to the formal qualification (ANZSCO Skill Level 1).

Tasks Include:

- establishing administrative and operational procedures by taking account of the organisation's operating environment
- making policy decisions and accepting responsibility for operations, performance of staff, achievement of targets and adherence to budgets, standards and procedures
- establishing lines of control and delegating responsibilities to subordinate staff
- representing the organisation in dealings with other organisations and the public
- controlling the collection and interpretation of management information to monitor performance
- controlling the use of, and accounting for, the assets and facilities of the organisation
- preparing budgets and other management plans
- preparing reports, authorising the release of information, and handling public relations activities

Occupations:

139111 Commissioned Defence Force Officer
139112 Commissioned Fire Officer
139113 Commissioned Police Officer

UNIT GROUP 1391 COMMISSIONED OFFICERS (MANAGEMENT) *continued*

139111 COMMISSIONED DEFENCE FORCE OFFICER

Provides high level management to support the running of an organisational unit within the Australian or New Zealand Defence Forces.

Skill Level: 1

This occupation includes the following ranks:

Air Force: Flight Lieutenant, Flying Officer, Pilot Officer, Squadron Leader

Army: Captain, Lieutenant, Major, Second Lieutenant

Navy: Lieutenant, Lieutenant Commander, Midshipman, Sub Lieutenant

This occupation excludes Commissioned Defence Force Officers performing duties for which there is a civilian equivalent. These officers are included with the civilian occupation, for example, Nursing Officers are included in Occupation 254499 Registered Nurses nec.

Specialisations:

- Air Defence Officer (Air Force) (Aus)
- Ground Defence Officer (Air Force) (Aus)
- Military Police Officer (Air Force) (NZ)
- Security Police Officer (Air Force) (Aus)
- Armoured Corps Officer (Army)
- Artillery Officer (Army)
- General Service Officer (Army) (Aus)
- Infantry Officer (Army)
- Military Police Officer (Army)
- Special Service Officer (Army) (Aus)
- Military Police Officer (Navy) (NZ)
- Observer (Navy)

139112 COMMISSIONED FIRE OFFICER

Provides high level management to support the running of a geographical or operational section of a fire service.

Skill Level: 1

Specialisations:

- Fire Investigator
- Inspector (Fire Services)

139113 COMMISSIONED POLICE OFFICER

Provides high level management to support the running of a geographical or operational section of a police service.

Skill Level: 1

UNIT GROUP 1392 SENIOR NON-COMMISSIONED DEFENCE FORCE MEMBERS

SENIOR NON-COMMISSIONED DEFENCE FORCE MEMBERS implement and enforce directives of commissioned officers of the Australian and New Zealand Defence Forces.

Indicative Skill Level:

In Australia and New Zealand:

Most occupations in this unit group have a level of skill commensurate with a bachelor degree or higher qualification. At least five years of relevant experience may substitute for the formal qualification. In some instances relevant experience and/or on-the-job training may be required in addition to the formal qualification (ANZSCO Skill Level 1).

Tasks Include:

- supervising a small group of subordinates
- maintaining discipline of subordinates
- monitoring the morale, welfare and behaviour of subordinates, and advising commanding officers accordingly
- instructing subordinates in dress, deportment, demeanour, behaviour, drill and devotion to duty to maintain military standards
- conducting military drills and inspections
- teaching military traditions and customs

Occupation:

139211 Senior Non-commissioned Defence Force Member

139211 SENIOR NON-COMMISSIONED DEFENCE FORCE MEMBER

Implements and enforces directives of commissioned officers of the Australian or New Zealand Defence Forces.

Skill Level: 1

This occupation includes the following ranks:

Air Force: Flight Sergeant, Sergeant, Warrant Officer, Warrant Officer of the Air Force

Army: Regimental Sergeant Major of the Army, Sergeant, Warrant Officer Class 1, Warrant Officer Class 2

Navy: Chief Petty Officer, Petty Officer, Warrant Officer, Warrant Officer of the Navy

UNIT GROUP 1399 OTHER SPECIALIST MANAGERS

This unit group covers Specialist Managers not elsewhere classified.

It includes Arts Administrators or Managers, Environmental Managers, Laboratory Managers, Quality Assurance Managers and Sports Administrators.

Indicative Skill Level:
In Australia and New Zealand:

Most occupations in this unit group have a level of skill commensurate with a bachelor degree or higher qualification. At least five years of relevant experience may substitute for the formal qualification. In some instances relevant experience and/or on-the-job training may be required in addition to the formal qualification (ANZSCO Skill Level 1).

Occupations:

139911 Arts Administrator or Manager
139912 Environmental Manager
139913 Laboratory Manager
139914 Quality Assurance Manager
139915 Sports Administrator
139999 Specialist Managers nec

139911 ARTS ADMINISTRATOR OR MANAGER

Plans, organises, directs, controls, coordinates and promotes artistic and cultural policies, programs, projects and services.

Skill Level: 1

Specialisations:

Art Gallery Director
Community Arts Centre Manager
Cultural Centre Manager

139912 ENVIRONMENTAL MANAGER

Plans, organises, directs, controls and coordinates the development and implementation of an environmental management system within an organisation by identifying, solving and alleviating environmental issues, such as pollution and waste treatment, in compliance with environmental legislation and to ensure corporate sustainable development.

Skill Level: 1

139913 LABORATORY MANAGER

Plans, organises, directs, controls and coordinates the operations of a research or production laboratory.

Skill Level: 1

UNIT GROUP 1399 OTHER SPECIALIST MANAGERS *continued*

139914 QUALITY ASSURANCE MANAGER

Alternative Title:
Quality Facilitator

Plans, organises, directs, controls and coordinates the deployment of quality systems and certification processes within an organisation.

Skill Level: 1

Specialisation:
Quality Certification Manager (NZ)

139915 SPORTS ADMINISTRATOR

Plans, organises, directs, controls, coordinates and promotes sport and recreational activities, and develops related policies.

Skill Level: 1

139999 SPECIALIST MANAGERS NEC

This occupation group covers Specialist Managers not elsewhere classified.

Skill Level: 1

Occupations in this group include:
Airport Manager
Ambassador
Archbishop
Bishop
Harbour Master

SUB-MAJOR GROUP 14 HOSPITALITY, RETAIL AND SERVICE MANAGERS

HOSPITALITY, RETAIL AND SERVICE MANAGERS organise and control the operations of establishments which provide accommodation, hospitality, retail and other services.

Indicative Skill Level:
Most occupations in this sub-major group have a level of skill commensurate with the qualifications and experience outlined below.

In Australia:
AQF Associate Degree, Advanced Diploma or Diploma (ANZSCO Skill Level 2)

In New Zealand:
NZ Register Diploma (ANZSCO Skill Level 2)

At least three years of relevant experience may substitute for the formal qualifications listed above. In some instances relevant experience and/or on-the-job training may be required in addition to the formal qualification.

Tasks Include:
- planning and organising special functions, sporting, gaming and entertainment activities and the range and mix of products, stock levels and service standards
- promoting and selling goods and services
- observing liquor, gaming, health and other laws and regulations
- developing and reviewing policies, programs and procedures concerning customer relations and goods and services provided
- promoting facilities, conferences, conventions and trade shows to potential customers
- organising the purchase and maintenance of transport vehicles, equipment and fuel, and transporting goods
- controlling the selection, training and supervision of staff
- ensuring compliance with occupational health and safety regulations

Occupations in this sub-major group are classified into the following minor groups:
141 Accommodation and Hospitality Managers
142 Retail Managers
149 Miscellaneous Hospitality, Retail and Service Managers

MINOR GROUP 141 ACCOMMODATION AND HOSPITALITY MANAGERS

ACCOMMODATION AND HOSPITALITY MANAGERS organise and control the operations of establishments which provide accommodation and hospitality services.

Indicative Skill Level:
Most occupations in this minor group have a level of skill commensurate with the qualifications and experience outlined below.

In Australia:
AQF Associate Degree, Advanced Diploma or Diploma (ANZSCO Skill Level 2)

In New Zealand:
NZ Register Diploma (ANZSCO Skill Level 2)

At least three years of relevant experience may substitute for the formal qualifications listed above. In some instances relevant experience and/or on-the-job training may be required in addition to the formal qualification.

Tasks Include:

- planning and organising special functions, sporting, gaming and entertainment activities
- directing and overseeing reservation, reception, room service and housekeeping activities
- observing liquor, gaming, health and other laws and regulations
- monitoring quality at all stages of preparation and presentation of food and services
- controlling the selection, training and supervision of staff
- ensuring compliance with occupational health and safety regulations

Occupations in this minor group are classified into the following unit groups:

1411 Cafe and Restaurant Managers
1412 Caravan Park and Camping Ground Managers
1413 Hotel and Motel Managers
1414 Licensed Club Managers
1419 Other Accommodation and Hospitality Managers

UNIT GROUP 1411 CAFE AND RESTAURANT MANAGERS

CAFE AND RESTAURANT MANAGERS organise and control the operations of cafes, restaurants and related establishments to provide dining and catering services.

Indicative Skill Level:
Most occupations in this unit group have a level of skill commensurate with the qualifications and experience outlined below.

In Australia:
AQF Associate Degree, Advanced Diploma or Diploma (ANZSCO Skill Level 2)

In New Zealand:
NZ Register Diploma (ANZSCO Skill Level 2)

At least three years of relevant experience may substitute for the formal qualifications listed above. In some instances relevant experience and/or on-the-job training may be required in addition to the formal qualification.

Tasks Include:

- planning menus in consultation with Chefs
- planning and organising special functions
- arranging the purchasing and pricing of goods according to budget
- maintaining records of stock levels and financial transactions
- ensuring dining facilities comply with health regulations and are clean, functional and of suitable appearance
- conferring with customers to assess their satisfaction with meals and service
- selecting, training and supervising waiting and kitchen staff
- may take reservations, greet guests and assist in taking orders

Occupation:
141111 Cafe or Restaurant Manager

141111 CAFE OR RESTAURANT MANAGER

Alternative Titles:
Food and Beverage Manager
Restaurateur

Organises and controls the operations of a cafe, restaurant or related establishment to provide dining and catering services.

Skill Level: 2

Specialisations:
Bistro Manager
Canteen Manager
Caterer
Internet Cafe Manager
Mess Supervisor
Steward (Navy)

UNIT GROUP 1412 CARAVAN PARK AND CAMPING GROUND MANAGERS

CARAVAN PARK AND CAMPING GROUND MANAGERS organise and control the operations of caravan parks and camping grounds to provide accommodation and leisure services.

Indicative Skill Level:
Most occupations in this unit group have a level of skill commensurate with the qualifications and experience outlined below.

In Australia:
AQF Associate Degree, Advanced Diploma or Diploma (ANZSCO Skill Level 2)

In New Zealand:
NZ Register Diploma (ANZSCO Skill Level 2)

At least three years of relevant experience may substitute for the formal qualifications listed above. In some instances relevant experience and/or on-the-job training may be required in addition to the formal qualification.

Registration or licensing may be required.

Tasks Include:
- taking reservations, registering guests, assigning accommodation and collecting payments
- arranging cleaning and maintenance of caravans, cabins, amenities blocks, recreation facilities and grounds
- informing guests of local tourist attractions and recreational facilities
- attending to complaints lodged by customers
- liaising with local government to ensure compliance with laws, regulations and ordinances
- may operate an on-site shop, cafe or restaurant for guests
- may sign, supervise and enforce terms of tenancy agreements in accordance with legislation and may obtain enforcement orders where tenants are in breach of requirements
- may act as licensee for the purchase and sale of liquor

Occupation:
141211 Caravan Park and Camping Ground Manager

141211 CARAVAN PARK AND CAMPING GROUND MANAGER

Organises and controls the operations of a caravan park and camping ground to provide accommodation and leisure services. Registration or licensing may be required.

Skill Level: 2

UNIT GROUP 1413 HOTEL AND MOTEL MANAGERS

HOTEL AND MOTEL MANAGERS organise and control the operations of hotels and motels to provide guest accommodation, meals and other services.

Indicative Skill Level:
Most occupations in this unit group have a level of skill commensurate with the qualifications and experience outlined below.

In Australia:
AQF Associate Degree, Advanced Diploma or Diploma (ANZSCO Skill Level 2)

In New Zealand:
NZ Register Diploma (ANZSCO Skill Level 2)

At least three years of relevant experience may substitute for the formal qualifications listed above. In some instances relevant experience and/or on-the-job training may be required in addition to the formal qualification.

Registration or licensing may be required.

Tasks Include:
- directing and overseeing reservation, reception, room service and housekeeping activities
- supervising security arrangements, and garden and property maintenance
- planning and supervising bar, restaurant, function and conference activities
- observing liquor, gaming, and other laws and regulations
- assessing and reviewing customer satisfaction
- overseeing accounting and purchasing activities
- ensuring compliance with occupational health and safety regulations
- may provide guests with local tourism information, and arrange tours and transportation

Occupation:
141311 Hotel or Motel Manager

141311 HOTEL OR MOTEL MANAGER

Alternative Titles:
Hotelier
Publican

Organises and controls the operations of a hotel or motel to provide guest accommodation, meals and other services. Registration or licensing may be required.

Skill Level: 2

Specialisations:
Duty Manager (Hotel)
Resort Manager

UNIT GROUP 1414 LICENSED CLUB MANAGERS

LICENSED CLUB MANAGERS organise and control the operations of licensed clubs to provide food, beverages, gaming, entertainment, sporting and other amenities for members.

Indicative Skill Level:
Most occupations in this unit group have a level of skill commensurate with the qualifications and experience outlined below.

In Australia:
AQF Associate Degree, Advanced Diploma or Diploma (ANZSCO Skill Level 2)

In New Zealand:
NZ Register Diploma (ANZSCO Skill Level 2)

At least three years of relevant experience may substitute for the formal qualifications listed above. In some instances relevant experience and/or on-the-job training may be required in addition to the formal qualification.

Registration or licensing is required.

Tasks Include:
- planning and supervising bar, restaurant and function services
- planning, booking and supervising sporting, gaming and entertainment activities
- supervising security arrangements and property maintenance
- arranging member subscriptions
- observing liquor, gaming, health and other laws and regulations
- ensuring compliance with occupational health and safety regulations
- compiling and organising distribution of newsletters and other information to keep members informed of forthcoming events and facilities available
- assessing and reviewing member satisfaction and preferences
- liaising with community groups sponsored and assisted by the club
- selecting, training and supervising staff

Occupation:
141411 Licensed Club Manager

141411 LICENSED CLUB MANAGER

Alternative Title:
Club Licensee

Organises and controls the operations of a licensed club to provide food, beverages, gaming, entertainment, sporting and other amenities for members. Registration or licensing is required.

Skill Level: 2

Specialisations:
Gaming Manager
Nightclub Manager

UNIT GROUP 1419 OTHER ACCOMMODATION AND HOSPITALITY MANAGERS

This unit group covers Accommodation and Hospitality Managers not elsewhere classified.

It includes Bed and Breakfast Operators.

Indicative Skill Level:
Most occupations in this unit group have a level of skill commensurate with the qualifications and experience outlined below.

In Australia:
AQF Associate Degree, Advanced Diploma or Diploma (ANZSCO Skill Level 2)

In New Zealand:
NZ Register Diploma (ANZSCO Skill Level 2)

At least three years of relevant experience may substitute for the formal qualifications listed above. In some instances relevant experience and/or on-the-job training may be required in addition to the formal qualification.

Registration or licensing may be required.

Occupations:
141911 Bed and Breakfast Operator
141999 Accommodation and Hospitality Managers nec

141911 BED AND BREAKFAST OPERATOR

Organises and controls the operations of a bed and breakfast to provide a short term, highly personalised accommodation and leisure service for guests including breakfast. Ensures guests' needs, wants and comfort are satisfied during their stay. Registration or licensing may be required.

Skill Level: 2

141999 ACCOMMODATION AND HOSPITALITY MANAGERS NEC

This occupation group covers Accommodation and Hospitality Managers not elsewhere classified. Registration or licensing may be required.

Skill Level: 2

Occupations in this group include:
Backpackers Manager
Boarding House Manager
Casino Duty Manager
Guest House Manager
Hostel Manager
Reception Centre Manager
Retirement Village Manager

MINOR GROUP 142 RETAIL MANAGERS

RETAIL MANAGERS organise and control the operations of establishments which provide retail services.

Indicative Skill Level:
Most occupations in this minor group have a level of skill commensurate with the qualifications and experience outlined below.

In Australia:
AQF Associate Degree, Advanced Diploma or Diploma (ANZSCO Skill Level 2)

In New Zealand:
NZ Register Diploma (ANZSCO Skill Level 2)

At least three years of relevant experience may substitute for the formal qualifications listed above. In some instances relevant experience and/or on-the-job training may be required in addition to the formal qualification.

Tasks Include:
- determining product mix, stock levels and service standards
- formulating and implementing purchasing and marketing policies, and setting prices
- promoting and advertising the establishment's goods and services
- selling goods and services to customers and advising them on product use
- maintaining records of stock levels and financial transactions
- undertaking budgeting for the establishment
- controlling selection, training and supervision of staff
- ensuring compliance with occupational health and safety regulations

Occupations in this minor group are classified into the following unit group:
1421 Retail Managers

UNIT GROUP 1421 RETAIL MANAGERS

RETAIL MANAGERS organise and control the operations of establishments which provide retail services.

Indicative Skill Level:
Most occupations in this unit group have a level of skill commensurate with the qualifications and experience outlined below.

In Australia:
AQF Associate Degree, Advanced Diploma or Diploma (ANZSCO Skill Level 2)

In New Zealand:
NZ Register Diploma (ANZSCO Skill Level 2)

At least three years of relevant experience may substitute for the formal qualifications listed above. In some instances relevant experience and/or on-the-job training may be required in addition to the formal qualification.

Registration or licensing may be required.

Tasks Include:
- determining product mix, stock levels and service standards
- formulating and implementing purchasing and marketing policies, and setting prices
- promoting and advertising the establishment's goods and services
- selling goods and services to customers and advising them on product use
- maintaining records of stock levels and financial transactions
- undertaking budgeting for the establishment
- controlling selection, training and supervision of staff
- ensuring compliance with occupational health and safety regulations

Occupations:
142111 Retail Manager (General)
142112 Antique Dealer
142113 Betting Agency Manager
142114 Hair or Beauty Salon Manager
142115 Post Office Manager
142116 Travel Agency Manager

142111 RETAIL MANAGER (GENERAL)

Alternative Titles:
Retail Store Manager
Shop Manager

Organises and controls the operations of a retail trading establishment.

Retail Pharmacists are excluded from this occupation. Retail Pharmacists are included in Unit Group 2515 Pharmacists, in Occupation 251513 Retail Pharmacist.

Skill Level: 2

Specialisations:
Newsagent
Snack Bar Manager

UNIT GROUP 1421 RETAIL MANAGERS *continued*

142112 ANTIQUE DEALER

Buys and sells antiques such as furniture, art, jewellery and china. May also clean, restore and value antiques. Registration or licensing may be required.

Skill Level: 2

142113 BETTING AGENCY MANAGER

Organises and controls the operations of a branch of a betting agency. Registration or licensing is required.

Skill Level: 2

142114 HAIR OR BEAUTY SALON MANAGER

Organises and controls the operations of a hairdressing or beauty salon. Registration or licensing may be required.

Skill Level: 2

142115 POST OFFICE MANAGER

Organises and controls the operations of a post office.

Skill Level: 2

142116 TRAVEL AGENCY MANAGER

Organises and controls the operations of a travel agency. Registration or licensing may be required.

Skill Level: 2

MINOR GROUP 149 MISCELLANEOUS HOSPITALITY, RETAIL AND SERVICE MANAGERS

This minor group covers Hospitality, Retail and Service Managers not elsewhere classified.

It includes Amusement, Fitness and Sports Centre Managers, Call or Contact Centre and Customer Service Managers, Conference and Event Organisers, and Transport Services Managers.

Indicative Skill Level:
Most occupations in this minor group have a level of skill commensurate with the qualifications and experience outlined below.

In Australia:
AQF Associate Degree, Advanced Diploma or Diploma (ANZSCO Skill Level 2)

In New Zealand:
NZ Register Diploma (ANZSCO Skill Level 2)

At least three years of relevant experience may substitute for the formal qualifications listed above. In some instances relevant experience and/or on-the-job training may be required in addition to the formal qualification.

Occupations in this minor group are classified into the following unit groups:
1491 Amusement, Fitness and Sports Centre Managers
1492 Call or Contact Centre and Customer Service Managers
1493 Conference and Event Organisers
1494 Transport Services Managers
1499 Other Hospitality, Retail and Service Managers

UNIT GROUP 1491 AMUSEMENT, FITNESS AND SPORTS CENTRE MANAGERS

AMUSEMENT, FITNESS AND SPORTS CENTRE MANAGERS organise, control and promote the activities, facilities and resources of amusement, fitness and sports centres.

Indicative Skill Level:
Most occupations in this unit group have a level of skill commensurate with the qualifications and experience outlined below.

In Australia:
AQF Associate Degree, Advanced Diploma or Diploma (ANZSCO Skill Level 2)

In New Zealand:
NZ Register Diploma (ANZSCO Skill Level 2)

At least three years of relevant experience may substitute for the formal qualifications listed above. In some instances relevant experience and/or on-the-job training may be required in addition to the formal qualification.

Tasks Include:
- planning and organising the range and mix of entertainment, attractions, amusement machines and fitness programs to be offered by the centre
- organising publicity to promote facilities and attract clients
- scheduling games and competitions
- selecting, training and supervising staff
- ensuring facilities are properly maintained and conform to safety standards
- may undertake coaching, fitness instruction and training of clients
- may plan and organise catering facilities

Occupations:
149111 Amusement Centre Manager
149112 Fitness Centre Manager
149113 Sports Centre Manager

149111 AMUSEMENT CENTRE MANAGER

Alternative Title:
Entertainment Centre Manager

Organises, controls and promotes the activities, facilities and resources of an amusement centre, showground or theme park.

Skill Level: 2

Specialisations:
Bridge Club Manager
Fairground Operator
Video Arcade Manager

149112 FITNESS CENTRE MANAGER

Organises, controls and promotes the activities, facilities and resources of a fitness centre. May coach, instruct and train clients.

Skill Level: 2

UNIT GROUP 1491 AMUSEMENT, FITNESS AND SPORTS CENTRE MANAGERS *continued*

149113 SPORTS CENTRE MANAGER

Organises, controls and promotes the activities, facilities and resources of a sports centre.

Skill Level: 2

Specialisations:

- Aquatic Centre Manager
- Golf Course Manager
- Indoor Sports Centre Manager
- Squash Centre Manager
- Stadium Manager
- Tennis Centre Manager
- Ten Pin Bowling Centre Manager

UNIT GROUP 1492 CALL OR CONTACT CENTRE AND CUSTOMER SERVICE MANAGERS

CALL OR CONTACT CENTRE AND CUSTOMER SERVICE MANAGERS organise and control the operations of call or contact centres, review customer services, and maintain sound customer relations.

Indicative Skill Level:
Most occupations in this unit group have a level of skill commensurate with the qualifications and experience outlined below.

In Australia:
AQF Associate Degree, Advanced Diploma or Diploma (ANZSCO Skill Level 2)

In New Zealand:
NZ Register Diploma (ANZSCO Skill Level 2)

At least three years of relevant experience may substitute for the formal qualifications listed above. In some instances relevant experience and/or on-the-job training may be required in addition to the formal qualification.

Tasks Include:
- developing and reviewing policies, programs and procedures concerning customer relations and goods and services provided
- ensuring operational efficiency within a call centre
- providing direction and feedback to team members and assisting with recruitment
- managing, motivating and developing staff providing customer services
- planning and implementing after-sales services to follow up customer satisfaction, ensure performance of goods purchased, and modify and improve services provided
- liaising with other organisational units, service agents and customers to identify and respond to customer expectations
- may work in a call centre

Occupations:
149211 Call or Contact Centre Manager
149212 Customer Service Manager

149211 CALL OR CONTACT CENTRE MANAGER

Organises and controls the operations of a call or contact centre. May work in a call centre.

Skill Level: 2

149212 CUSTOMER SERVICE MANAGER

Alternative Titles:
Client Service Manager
Service Manager

Plans, administers and reviews customer services and after-sales services, and maintains sound customer relations.

Skill Level: 2

UNIT GROUP 1493 CONFERENCE AND EVENT ORGANISERS

CONFERENCE AND EVENT ORGANISERS organise and coordinate services for conferences, events, functions, banquets and seminars.

Indicative Skill Level:
Most occupations in this unit group have a level of skill commensurate with the qualifications and experience outlined below.

In Australia:
AQF Associate Degree, Advanced Diploma or Diploma (ANZSCO Skill Level 2)

In New Zealand:
NZ Register Diploma (ANZSCO Skill Level 2)

At least three years of relevant experience may substitute for the formal qualifications listed above. In some instances relevant experience and/or on-the-job training may be required in addition to the formal qualification.

Tasks Include:
- promoting conferences, conventions and trade shows to potential customers
- responding to inquiries concerning services provided and costs for room and equipment hire, catering and related services
- meeting with clients to discuss their needs and outlining package options to meet these needs
- arranging and coordinating services, such as conference facilities, catering, signage, displays, audiovisual equipment, accommodation, transport and social events, for participants
- organising registration of participants
- negotiating the type and costs of services to be provided within budget
- overseeing work by contractors and reporting on variations to work orders

Occupation:
149311 Conference and Event Organiser

149311 CONFERENCE AND EVENT ORGANISER

Alternative Title:
Event Management Consultant

Organises and coordinates services for conferences, events, functions, banquets and seminars.

Skill Level: 2

Specialisations:
Event Planner
Exhibition Organiser
Wedding Coordinator

UNIT GROUP 1494 TRANSPORT SERVICES MANAGERS

TRANSPORT SERVICES MANAGERS organise and control the buying and selling of vehicles for rental agencies and coordinates the leasing of vehicles, the operations of railway stations, and the operations of enterprises that operate fleets of vehicles to transport goods and passengers.

Indicative Skill Level:
Most occupations in this unit group have a level of skill commensurate with the qualifications and experience outlined below.

In Australia:
AQF Associate Degree, Advanced Diploma or Diploma (ANZSCO Skill Level 2)

In New Zealand:
NZ Register Diploma (ANZSCO Skill Level 2)

At least three years of relevant experience may substitute for the formal qualifications listed above. In some instances relevant experience and/or on-the-job training may be required in addition to the formal qualification.

Registration or licensing may be required.

Tasks Include:
- organising the purchase and maintenance of transport vehicles, equipment and fuel
- liaising with clients to determine requirements and providing customers with advice and information regarding vehicle type, purchase or hire rates and obligations and handling complaints
- receiving orders and bookings, and planning and implementing transportation schedules
- ensuring goods are stored and transported in conditions that will maintain their quality
- arranging collection and delivery of vehicles and goods
- maintaining business records and preparing operational statements and reports
- coordinating activities associated with the arrival, departure, loading and unloading of trains
- ensuring compliance with occupational health and safety regulations

Occupations:
149411 Fleet Manager
149412 Railway Station Manager
149413 Transport Company Manager

149411 FLEET MANAGER

Organises and controls the buying and selling of vehicles for rental agencies and coordinates the leasing of vehicles. Registration or licensing may be required.

Skill Level: 2

149412 RAILWAY STATION MANAGER

Organises and controls the operations of a railway station.

Skill Level: 2

UNIT GROUP 1494 TRANSPORT SERVICES MANAGERS *continued*

149413 TRANSPORT COMPANY MANAGER

Organises and controls the operations of an enterprise that operates a fleet of vehicles to transport goods and passengers. Registration or licensing may be required.

Skill Level: 2

Specialisations:
- Bus Company Manager
- Car Rental Agency Manager

UNIT GROUP 1499 OTHER HOSPITALITY, RETAIL AND SERVICE MANAGERS

This unit group covers Hospitality, Retail and Service Managers not elsewhere classified.

It includes Boarding Kennel or Cattery Operators, Cinema or Theatre Managers, Facilities Managers and Financial Institution Branch Managers.

Indicative Skill Level:
Most occupations in this unit group have a level of skill commensurate with the qualifications and experience outlined below.

In Australia:
AQF Associate Degree, Advanced Diploma or Diploma (ANZSCO Skill Level 2)

In New Zealand:
NZ Register Diploma (ANZSCO Skill Level 2)

At least three years of relevant experience may substitute for the formal qualifications listed above. In some instances relevant experience and/or on-the-job training may be required in addition to the formal qualification.

Registration or licensing may be required.

Occupations:
149911 Boarding Kennel or Cattery Operator
149912 Cinema or Theatre Manager
149913 Facilities Manager
149914 Financial Institution Branch Manager
149999 Hospitality, Retail and Service Managers nec

149911 BOARDING KENNEL OR CATTERY OPERATOR

Organises and controls the operations of an establishment which offers temporary boarding for dogs and cats.

Skill Level: 2

149912 CINEMA OR THEATRE MANAGER

Organises and controls the operations of a cinema or theatre. Registration or licensing may be required.

Skill Level: 2

149913 FACILITIES MANAGER

Alternative Title:
Building Manager

Organises, controls and coordinates the strategic and operational management of facilities in a public or private organisation.

Skill Level: 2

Specialisation:
Shopping Centre Manager

UNIT GROUP 1499 OTHER HOSPITALITY, RETAIL AND SERVICE MANAGERS *continued*

149914 FINANCIAL INSTITUTION BRANCH MANAGER

Organises and controls the general operational activities of a branch of a bank, building society, credit union or similar financial institution.

Skill Level: 2

Specialisations:
- Bank Manager
- Building Society Manager
- Credit Union Manager

149999 HOSPITALITY, RETAIL AND SERVICE MANAGERS NEC

This occupation group covers Hospitality, Retail and Service Managers not elsewhere classified. Registration or licensing may be required.

Skill Level: 2

Occupations in this group include:
- Abattoir Manager
- Brothel Keeper
- Equipment Hire Manager
- Laundrette Owner
- Marina Manager
- Nursing Agency Manager
- Taxi Proprietor
- Weight Loss Centre Manager

MAJOR GROUP 2 PROFESSIONALS

PROFESSIONALS perform analytical, conceptual and creative tasks through the application of theoretical knowledge and experience in the fields of the arts, media, business, design, engineering, the physical and life sciences, transport, education, health, information and communication technology, the law, social sciences and social welfare.

Indicative Skill Level:
Most occupations in this major group have a level of skill commensurate with the qualifications and experience outlined below.

In Australia:

Bachelor degree or higher qualification. At least five years of relevant experience may substitute for the formal qualification (ANZSCO Skill Level 1); or

AQF Associate Degree, Advanced Diploma or Diploma, or at least three years of relevant experience (ANZSCO Skill Level 2)

In New Zealand:

Bachelor degree or higher qualification. At least five years of relevant experience may substitute for the formal qualification (ANZSCO Skill Level 1); or

NZ Register Diploma, or at least three years of relevant experience (ANZSCO Skill Level 2).

In some instances relevant experience and/or on-the-job training may be required in addition to the formal qualification.

Some occupations, such as those in Sub-Major Group 21 Arts and Media Professionals, require high levels of creative talent or personal commitment and interest as well as, or in place of, formal qualifications or experience.

Tasks Include:

- communicating ideas through language, printed and electronic media, and artistic media including the visual and performing arts
- analysing, planning, developing and implementing programs and solutions to resolve business and economic problems
- providing services in financial accounting, human resource development, publicity and marketing, and the efficient operation of organisations
- flying aircraft, and controlling and directing the operation of ships, boats and marine equipment
- conducting and analysing research to extend the body of knowledge in the field of the sciences and developing techniques to apply this knowledge
- designing products, buildings and other physical structures, and engineering systems
- researching and developing curricula, and teaching students in a range of educational settings
- designing, implementing, testing and maintaining technologies and services that enable information to be accessed, stored, manipulated, processed, and disseminated
- identifying, treating, and advising on, health, social, and personal issues
- advising clients on legal matters

Occupations in this major group are classified into the following sub-major groups:

21 Arts and Media Professionals

22 Business, Human Resource and Marketing Professionals

23 Design, Engineering, Science and Transport Professionals

24 Education Professionals

25 Health Professionals

26 ICT Professionals

27 Legal, Social and Welfare Professionals

SUB-MAJOR GROUP 21 ARTS AND MEDIA PROFESSIONALS

ARTS AND MEDIA PROFESSIONALS communicate ideas, impressions and factual information through printed, electronic, visual and performance media, and produce, direct and present film, television, radio and stage productions.

Indicative Skill Level:

In Australia and New Zealand:

Most occupations in this sub-major group have a level of skill commensurate with a bachelor degree or higher qualification. At least five years of relevant experience may substitute for the formal qualification. In some instances relevant experience and/or on-the-job training may be required in addition to the formal qualification. Some occupations in this sub-major group require high levels of creative talent or personal commitment and interest as well as, or in place of, formal qualifications or experience (ANZSCO Skill Level 1).

Tasks Include:

- conceiving and developing ideas, styles, characters, emotions and themes and translating them through artistic presentation
- composing music and dance routines and creating visual art forms such as paintings, pictures and sculptures
- writing, reviewing and editing journalistic copy, literary works, musical compositions, and film, television and radio scripts
- presenting information and producing, directing and performing various kinds of entertainment

Occupations in this sub-major group are classified into the following minor groups:

211 Arts Professionals

212 Media Professionals

MINOR GROUP 211 ARTS PROFESSIONALS

ARTS PROFESSIONALS communicate ideas, impressions and facts in a range of media to achieve particular effects, and interpret compositions such as a musical scores and scripts for performance.

Indicative Skill Level:

In Australia and New Zealand:

Most occupations in this minor group have a level of skill commensurate with a bachelor degree or higher qualification. At least five years of relevant experience may substitute for the formal qualification. In some instances relevant experience and/or on-the-job training may be required in addition to the formal qualification. Some occupations in this minor group require high levels of creative talent or personal commitment and interest as well as, or in place of, formal qualifications or experience (ANZSCO Skill Level 1).

Tasks Include:

- conceiving and developing ideas, styles, characters, emotions and themes and translating them through artistic presentation
- reading scripts and undertaking research to gain understanding of parts, themes and characterisations
- composing music and dance routines and creating visual forms such as paintings, pictures and sculptures
- learning lines, parts, cues, dance routines and music
- performing music, dances, dramatic roles and other parts for audience entertainment
- transporting and setting up photographic equipment, and taking photographs

Occupations in this minor group are classified into the following unit groups:

2111 Actors, Dancers and Other Entertainers
2112 Music Professionals
2113 Photographers
2114 Visual Arts and Crafts Professionals

UNIT GROUP 2111 ACTORS, DANCERS AND OTHER ENTERTAINERS

ACTORS, DANCERS AND OTHER ENTERTAINERS entertain by portraying roles in productions, performing and composing dances, and performing a variety of other acts.

Indicative Skill Level:

In Australia and New Zealand:

Most occupations in this unit group have a level of skill commensurate with a bachelor degree or higher qualification. At least five years of relevant experience may substitute for the formal qualification. Some occupations in this unit group require high levels of creative talent or personal commitment and interest as well as, or in place of, formal qualifications or experience (ANZSCO Skill Level 1).

Tasks Include:

- reading scripts and undertaking research to gain understanding of parts, themes and characterisations
- learning lines and cues, rehearsing parts, and applying vocal and movement skills to the development of characterisation
- preparing for performances through rehearsals under the instruction and guidance of production directors
- acting parts and portraying roles as developed in rehearsals in film, television, radio and stage productions
- practising dance routines and interpreting the choreographic content of the production
- performing dances for audience entertainment, coordinating body movements and facial expression, usually with musical accompaniment
- composing and notating ballet compositions and other dance routines
- creating and performing individual performance routines
- rehearsing, auditioning and travelling between entertainment venues

Occupations:

211111 Actor
211112 Dancer or Choreographer
211113 Entertainer or Variety Artist
211199 Actors, Dancers and Other Entertainers nec

211111 ACTOR

Entertains by portraying roles in film, television, radio and stage productions. This occupation requires high levels of creative talent or personal commitment and interest as well as, or in place of, formal qualifications or experience.

Skill Level: 1

Specialisations:

Mime Artist
Voice-over Artist

UNIT GROUP 2111 ACTORS, DANCERS AND OTHER ENTERTAINERS *continued*

211112 DANCER OR CHOREOGRAPHER

Entertains by performing dances, or creates dance compositions. This occupation requires high levels of creative talent or personal commitment and interest as well as, or in place of, formal qualifications or experience.

Skill Level: 1

Specialisations:

- Ballet Dancer
- Contemporary or Modern Dancer
- Exotic Dancer

211113 ENTERTAINER OR VARIETY ARTIST

Entertains by performing a variety of acts using a mix of acting, singing, dance and movement skills. This occupation requires high levels of creative talent or personal commitment and interest as well as, or in place of, formal qualifications or experience.

Skill Level: 1

Specialisations:

- Busker
- Circus Artist
- Clown
- Comedian
- Magician/Illusionist
- Puppeteer
- Ventriloquist

211199 ACTORS, DANCERS AND OTHER ENTERTAINERS NEC

This occupation group covers Actors, Dancers and Other Entertainers not elsewhere classified. This occupation group requires high levels of creative talent or personal commitment and interest as well as, or in place of, formal qualifications or experience.

Skill Level: 1

Occupations in this group include:

- Disc Jockey (Nightclub)
- Motivational Speaker
- Performance Artist
- Public Speaker
- Stunt Performer

UNIT GROUP 2112 MUSIC PROFESSIONALS

MUSIC PROFESSIONALS write, arrange, orchestrate, conduct and perform musical compositions.

Indicative Skill Level:
In Australia and New Zealand:

Most occupations in this unit group have a level of skill commensurate with a bachelor degree or higher qualification. At least five years of relevant experience may substitute for the formal qualification. Some occupations in this unit group require high levels of creative talent or personal commitment and interest as well as, or in place of, formal qualifications or experience (ANZSCO Skill Level 1).

Tasks Include:

- creating melodic, harmonic and rhythmic structures to express ideas and emotions in musical form
- translating ideas and concepts into standard musical signs and symbols for reproduction and performance
- undertaking research and liaising with clients when composing musical backing for television commercials, popular recordings, and radio, television and film productions
- auditioning and selecting musicians and Singers
- selecting music for performances and assigning instrumental parts to musicians
- directing musical groups at rehearsals and performances to achieve desired effects such as tonal and harmonic balance, rhythm and tempo
- studying and rehearsing repertoire and musical scores prior to performances
- playing music in recital, as an accompanist, or as a member of an orchestra, band or other musical group, from score and by memory
- performing music and songs according to interpretation, direction and style of presentation, using highly developed aural skills to reproduce music

Occupations:

211211 Composer
211212 Music Director
211213 Musician (Instrumental)
211214 Singer
211299 Music Professionals nec

211211 COMPOSER

Writes new and rearranges existing musical compositions such as songs, operas, symphonies, musical scores and advertising jingles. This occupation requires high levels of creative talent or personal commitment and interest as well as, or in place of, formal qualifications or experience.

Skill Level: 1

Specialisations:

Orchestrator
Music Arranger
Songwriter

UNIT GROUP 2112 MUSIC PROFESSIONALS *continued*

211212 MUSIC DIRECTOR

Conducts choirs, orchestras, bands, ensembles, opera companies and musical performances. This occupation requires high levels of creative talent or personal commitment and interest as well as, or in place of, formal qualifications or experience.

Skill Level: 1

Specialisations:

- Band Leader
- Choral Director
- Orchestra Conductor

211213 MUSICIAN (INSTRUMENTAL)

Alternative Title:

- Instrumentalist

Entertains by playing one or more musical instruments. This occupation requires high levels of creative talent or personal commitment and interest as well as, or in place of, formal qualifications or experience.

Skill Level: 1

Specialisations:

- Drummer
- Guitarist
- Pianist
- Violinist

211214 SINGER

Alternative Title:

- Vocalist

Entertains by singing songs. This occupation requires high levels of creative talent or personal commitment and interest as well as, or in place of, formal qualifications or experience.

Skill Level: 1

Specialisations:

- Band Singer
- Chorister
- Commercial Singer (Advertising)
- Jazz Singer
- Opera Singer
- Pop Singer
- Rock Singer

UNIT GROUP 2112 MUSIC PROFESSIONALS *continued*

211299 MUSIC PROFESSIONALS NEC

This occupation group covers Music Professionals not elsewhere classified.

Skill Level: 1

Occupations in this group include:

- Ethnomusicologist
- Music Copyist
- Music Researcher

UNIT GROUP 2113 PHOTOGRAPHERS

PHOTOGRAPHERS operate still cameras to take photographs.

Indicative Skill Level:

In Australia and New Zealand:

Most occupations in this unit group have a level of skill commensurate with a bachelor degree or higher qualification. At least five years of relevant experience may substitute for the formal qualification. The occupation in this unit group requires high levels of creative talent or personal commitment and interest as well as, or in place of, formal qualifications or experience (ANZSCO Skill Level 1).

Tasks Include:

- consulting with clients to determine objectives of photographic assignments
- studying requirements of assignment and selecting type of camera, film, filter and lighting
- working from a studio, and transporting and setting up equipment at assigned locations
- measuring light levels and determining exposure
- adjusting camera angles, aperture settings and subjects to achieve desired composition
- adjusting lighting and filters to accentuate highlights and colours
- mixing photographic chemicals, processing film and developing prints
- manipulating images using digital imaging techniques and creative darkroom techniques such as adjusting exposure and development times
- may make enlarged prints, and mount and frame prints

Occupation:

211311 Photographer

211311 PHOTOGRAPHER

Operates a still camera to take photographs. This occupation requires high levels of creative talent or personal commitment and interest as well as, or in place of, formal qualifications or experience.

Skill Level: 1

Specialisations:

Fashion Photographer
Landscape Photographer
News Photographer
Photographic Artist
Portrait Photographer
Sports Photographer
Technical Photographer

UNIT GROUP 2114 VISUAL ARTS AND CRAFTS PROFESSIONALS

VISUAL ARTS AND CRAFTS PROFESSIONALS create visual two- and three-dimensional concepts and forms through painting, drawing, carving, sculpting, modelling, printmaking, video, multimedia, found objects, ephemera, soundscapes and other media to communicate concepts and ideas.

Indicative Skill Level:

In Australia and New Zealand:

Most occupations in this unit group have a level of skill commensurate with a bachelor degree or higher qualification. At least five years of relevant experience may substitute for the formal qualification. Some occupations in this unit group require high levels of creative talent or personal commitment and interest as well as, or in place of, formal qualifications or experience (ANZSCO Skill Level 1).

Tasks Include:

- conceiving and developing ideas, designs and styles for paintings, drawings, pottery pieces and sculptures
- arranging objects, positioning models, and selecting landscapes and other visual forms according to chosen subject matter
- selecting artistic media, method and materials
- applying media to surfaces using appropriate techniques
- moulding clay into functional and artistic articles by wheel-throwing, moulding and hand-building
- mixing glazing materials and applying glazes to dried pottery by dipping and painting
- placing finished and decorated pieces in kilns
- sketching designs of proposed sculptures, and making wax and plaster models
- devising forms from metal using welding and metalworking equipment, and from stone using masonry tools
- carving and forming materials to desired shape using hand and power tools

Occupations:

211411 Painter (Visual Arts)
211412 Potter or Ceramic Artist
211413 Sculptor
211499 Visual Arts and Crafts Professionals nec

211411 PAINTER (VISUAL ARTS)

Alternative Title:

Painter (Artistic)

Conceives and creates visual art forms, such as pictures, abstract designs and similar compositions, to communicate impressions and ideas. This occupation requires high levels of creative talent or personal commitment and interest as well as, or in place of, formal qualifications or experience.

Skill Level: 1

211412 POTTER OR CERAMIC ARTIST

Conceives and creates functional or artistic articles by moulding clay, glass and other fusible materials using hand-building and wheel techniques. This occupation requires high levels of creative talent or personal commitment and interest as well as, or in place of, formal qualifications or experience.

Skill Level: 1

UNIT GROUP 2114 VISUAL ARTS AND CRAFTS PROFESSIONALS *continued*

211413 SCULPTOR

Conceives and creates three-dimensional forms to communicate impressions and ideas by carving or modelling materials, such as wood, stone, clay and metal, or assembling found and manufactured materials. This occupation requires high levels of creative talent or personal commitment and interest as well as, or in place of, formal qualifications or experience.

Skill Level: 1

211499 VISUAL ARTS AND CRAFTS PROFESSIONALS NEC

This occupation group covers Visual Arts and Crafts Professionals not elsewhere classified. This occupation group requires high levels of creative talent or personal commitment and interest as well as, or in place of, formal qualifications or experience.

Skill Level: 1

Occupations in this group include:

- Ephemeral Artist
- Leadlighter
- Multimedia Artist
- New Media Artist
- Textile Artist

MINOR GROUP 212 MEDIA PROFESSIONALS

MEDIA PROFESSIONALS direct and produce film, television, radio and stage productions, present programs on radio and television, write and edit literary works and scripts, and research, write and edit news reports, articles and material for advertising.

Indicative Skill Level:

In Australia and New Zealand:

Most occupations in this minor group have a level of skill commensurate with a bachelor degree or higher qualification. At least five years of relevant experience may substitute for the formal qualification. In some instances relevant experience and/or on-the-job training may be required in addition to the formal qualification (ANZSCO Skill Level 1).

Tasks Include:

- managing artistic and media productions to meet quality, cost and timing specifications
- studying scripts and scenarios to determine theme and setting, and assessing staging, production and technical requirements
- preparing and presenting news, sports and other information, conducting interviews and introducing music, guests and special events on radio and television
- writing literary works for publication and scripts for film, television, radio and stage productions
- gathering facts about newsworthy events, and writing news reports, commentaries and feature stories for presentation in print and electronic media
- reviewing written material to ensure consistency in style, grammar and content, and coherence of story

Occupations in this minor group are classified into the following unit groups:

2121 Artistic Directors, and Media Producers and Presenters
2122 Authors, and Book and Script Editors
2123 Film, Television, Radio and Stage Directors
2124 Journalists and Other Writers

UNIT GROUP 2121 ARTISTIC DIRECTORS, AND MEDIA PRODUCERS AND PRESENTERS

ARTISTIC DIRECTORS, AND MEDIA PRODUCERS AND PRESENTERS plan, administer and review activities concerned with producing artistic media, determine artistic policies for performing arts organisations, prepare and present news, sports and other information, and conduct interviews and introduce music, performances and special events on radio and television.

Indicative Skill Level:

In Australia and New Zealand:

Most occupations in this unit group have a level of skill commensurate with a bachelor degree or higher qualification. At least five years of relevant experience may substitute for the formal qualification. Artistic Directors and Media Producers (excluding Video) require relevant experience in addition to the formal qualification. Radio and Television Presenters require high levels of creative talent or personal commitment and interest as well as, or in place of, formal qualifications and experience (ANZSCO Skill Level 1).

Tasks Include:

- managing artistic and media productions to meet quality, cost and timing specifications
- formulating and developing organisations' artistic policies and selecting and planning organisations' artistic programs
- hiring and managing artistic staff
- controlling the use of media production facilities such as studios and editing equipment, stage and film equipment and rehearsal time
- directing the formulation of media production strategies, policies and plans
- introducing programs, music, entertainment items, guests and celebrities
- preparing and reading news bulletins, making special announcements and providing commentary for sports and other events
- presenting opinions on sports, politics, and social and economic matters
- researching, investigating and compiling stories and programs

Occupations:

212111 Artistic Director
212112 Media Producer (excluding Video)
212113 Radio Presenter
212114 Television Presenter

212111 ARTISTIC DIRECTOR

Determines and oversees implementation of artistic policies for a performing arts organisation such as a theatre company, dance company, music ensemble, festival or venue.

Skill Level: 1

UNIT GROUP 2121 ARTISTIC DIRECTORS, AND MEDIA PRODUCERS AND PRESENTERS *continued*

212112 MEDIA PRODUCER (EXCLUDING VIDEO)

Plans, administers and reviews activities concerned with publishing, or the production of films, television or radio programs, theatre, music, festivals or other artistic activities.

Skill Level: 1

Specialisations:

- Executive Producer
- Film Producer
- Stage Producer
- Television Producer

212113 RADIO PRESENTER

Alternative Title:

- Radio Announcer

Prepares and presents news, sports or other information, conducts interviews, and introduces music, performances and special events on radio. This occupation requires high levels of creative talent or personal commitment and interest as well as, or in place of, formal qualifications and experience.

Skill Level: 1

Specialisations:

- Disc Jockey (Radio)
- Talkback Host
- Tourism Radio Presenter

212114 TELEVISION PRESENTER

Alternative Titles:

- Commentator (Television)
- Compere (Television)

Prepares and presents news, sports or other information, conducts interviews, and introduces music, performances and special events on television. This occupation requires high levels of creative talent or personal commitment and interest as well as, or in place of, formal qualifications and experience.

Skill Level: 1

UNIT GROUP 2122 AUTHORS, AND BOOK AND SCRIPT EDITORS

AUTHORS, AND BOOK AND SCRIPT EDITORS write, edit and evaluate literary works for publication and scripts for film, television, radio and stage productions.

Indicative Skill Level:
In Australia and New Zealand:

Most occupations in this unit group have a level of skill commensurate with a bachelor degree or higher qualification. At least five years of relevant experience may substitute for the formal qualification. Some occupations in this unit group require high levels of creative talent or personal commitment and interest as well as, or in place of, formal qualifications or experience (ANZSCO Skill Level 1).

Tasks Include:

- creating and developing ideas and themes for written works, such as novels, plays, musicals, screen productions, educational texts, information texts and multimedia products
- researching subject matter through original and secondary materials, interviews and other media
- planning, organising and writing material
- reviewing and evaluating manuscripts of novels, biographies, short stories, poems, educational texts and other books, and ensuring coherence of style and development of theme, plot and characterisation
- advising publishers about potential of works for publication and conditions of publication contract
- negotiating publication details such as royalties, publication dates and numbers of copies to be printed
- reviewing and assessing stories and other material for film, television, radio and stage productions
- directing the preparation of scripts to be read by announcers to introduce and connect parts of musicals, news, sports and special events programs

Occupations:

212211 Author
212212 Book or Script Editor

212211 AUTHOR

Produces literary or other written work for publication or performance. This occupation requires high levels of creative talent or personal commitment and interest as well as, or in place of, formal qualifications and experience.

Skill Level: 1

Specialisations:

Novelist
Playwright
Poet
Screenwriter
Script Writer

212212 BOOK OR SCRIPT EDITOR

Evaluates manuscripts of books or scripts to determine suitability for publication or production, and edits and supervises material in preparation for publication or for production on film, television, radio or stage.

Skill Level: 1

Specialisations:

Dramaturge
Script Coordinator

UNIT GROUP 2123 FILM, TELEVISION, RADIO AND STAGE DIRECTORS

FILM, TELEVISION, RADIO AND STAGE DIRECTORS direct the artistic and production aspects of film, television, radio and stage productions.

Indicative Skill Level:

In Australia and New Zealand:

Most occupations in this unit group have a level of skill commensurate with a bachelor degree or higher qualification. At least five years of relevant experience may substitute for the formal qualification. Some occupations in this unit group require high levels of creative talent or personal commitment and interest as well as, or in place of, formal qualifications or experience (ANZSCO Skill Level 1).

Tasks Include:

- studying scripts and scenarios to determine theme and setting
- assessing locations and staging requirements for productions in association with specialist designers
- overseeing creative aspects of film, television, radio and stage productions
- determining lighting, film, shutter angles, filter factors, camera distance, depth of field and focus, angles of view and other variables to achieve desired mood and effect
- viewing film and video tape to evaluate and select scenes and determine which scenes need to be re-shot
- planning and organising the preparation and presentation of programs
- supervising the positioning of scenery, props and lighting and sound equipment
- assessing technical requirements of productions by studying scripts and discussing program content, set locations and stage directions with production team
- creating, planning, writing scripts for, recording, videotaping and editing programs

Occupations:

212311 Art Director (Film, Television or Stage)
212312 Director (Film, Television, Radio or Stage)
212313 Director of Photography
212314 Film and Video Editor
212315 Program Director (Television or Radio)
212316 Stage Manager
212317 Technical Director
212318 Video Producer
212399 Film, Television, Radio and Stage Directors nec

212311 ART DIRECTOR (FILM, TELEVISION OR STAGE)

Alternative Title:

Production Designer

Plans, organises and controls artistic aspects of film, television or stage productions. This occupation requires high levels of creative talent or personal commitment and interest as well as, or in place of, formal qualifications and experience.

Skill Level: 1

UNIT GROUP 2123 FILM, TELEVISION, RADIO AND STAGE DIRECTORS *continued*

212312 DIRECTOR (FILM, TELEVISION, RADIO OR STAGE)

Interprets and approves selection of scripts, and directs and instructs cast and crew during filming, recording or performance of productions. This occupation requires high levels of creative talent or personal commitment and interest as well as, or in place of, formal qualifications and experience.

Skill Level: 1

212313 DIRECTOR OF PHOTOGRAPHY

Alternative Title:
Cinematographer

Plans, directs and coordinates filming to control the quality and style of photography in films or videos.

Skill Level: 1

212314 FILM AND VIDEO EDITOR

Makes and implements editorial decisions regarding mood, pace and climax of films, television programs, video productions or commercials.

Skill Level: 1

212315 PROGRAM DIRECTOR (TELEVISION OR RADIO)

Compiles and directs programs for television or radio.

Skill Level: 1

212316 STAGE MANAGER

Plans, organises, supervises and coordinates the activities of workers responsible for placing sets and properties, and operating lighting and sound equipment as part of film, television or stage productions.

Skill Level: 1

212317 TECHNICAL DIRECTOR

Alternative Title:
Technical Producer

Controls the quality of pictures and sound for television or radio programs by directing technical teams and planning and organising technical facilities.

Skill Level: 1

212318 VIDEO PRODUCER

Creates films, television programs, video productions or commercials by filming, adding sound and editing in digital or analogue format. This occupation requires high levels of creative talent or personal commitment and interest as well as, or in place of, formal qualifications and experience.

Skill Level: 1

UNIT GROUP 2123 FILM, TELEVISION, RADIO AND STAGE DIRECTORS *continued*

212399 FILM, TELEVISION, RADIO AND STAGE DIRECTORS NEC

This occupation group covers Film, Television, Radio and Stage Directors not elsewhere classified.

Skill Level: 1

Occupations in this group include:

- Casting Director
- Lighting Director
- Location Manager (Film or Television)

UNIT GROUP 2124 JOURNALISTS AND OTHER WRITERS

JOURNALISTS AND OTHER WRITERS research and compile news stories, write and edit news reports, commentaries and feature stories for presentation in print and electronic media, and compose written material to advertise goods and services.

Indicative Skill Level:

In Australia and New Zealand:

Most occupations in this unit group have a level of skill commensurate with a bachelor degree or higher qualification. At least five years of relevant experience may substitute for the formal qualification. In some instances relevant experience and/or on-the-job training may be required in addition to the formal qualification (ANZSCO Skill Level 1).

Tasks Include:

- determining advertising approach by consulting clients and management, and studying products to establish principal selling features
- writing advertisements for press, radio, television, cinema screens, billboards, catalogues and shop displays
- making decisions about the specific content of publications in conjunction with other senior editors and in accordance with editorial policies and guidelines
- reviewing copy for publication to ensure conformity with accepted rules of grammar, style and format, coherence of story, and accuracy, legality and probity of content
- collecting and analysing facts about newsworthy events from interviews, printed matter, investigations and observations
- writing news reports, commentaries, articles and feature stories for newspapers, magazines, journals, television and radio on topics of public interest
- researching and writing technical, information-based material and documentation for manuals, text books, handbooks and multimedia products
- critically discussing daily news topics in the editorial columns of newspapers and reviewing books, films and plays

Occupations:

212411 Copywriter
212412 Newspaper or Periodical Editor
212413 Print Journalist
212414 Radio Journalist
212415 Technical Writer
212416 Television Journalist
212499 Journalists and Other Writers nec

212411 COPYWRITER

Designs and composes written material to advertise products and services.

Skill Level: 1

UNIT GROUP 2124 JOURNALISTS AND OTHER WRITERS *continued*

212412 NEWSPAPER OR PERIODICAL EDITOR

Alternative Title:

Associate Editor

Plans and directs editing of a publication, such as a newspaper, magazine or journal, in accordance with editorial policies and guidelines and accepted rules of grammar, style and format prior to printing and distribution.

Skill Level: 1

Specialisations:

Features Editor
News Editor
Pictures Editor
Subeditor
Web Site/Blog Editor

212413 PRINT JOURNALIST

Collects and analyses facts about newsworthy events by interview, investigation and observation and writes stories for newspapers, magazines or journals.

Skill Level: 1

Specialisations:

Columnist
Feature Writer
Leader Writer
Newspaper Reporter

212414 RADIO JOURNALIST

Alternative Title:

Radio Reporter

Collects and analyses facts about newsworthy events by interview, investigation and observation and writes stories for radio news or current affairs programs.

Skill Level: 1

212415 TECHNICAL WRITER

Researches and writes technical information-based material and documentation for articles, manuals, text books, handbooks, or multimedia products, usually for education or corporate purposes.

Skill Level: 1

212416 TELEVISION JOURNALIST

Alternative Title:

Television Reporter

Collects and analyses facts about newsworthy events by interview, investigation and observation and writes stories for television news or current affairs programs.

Skill Level: 1

UNIT GROUP 2124 JOURNALISTS AND OTHER WRITERS *continued*

212499 JOURNALISTS AND OTHER WRITERS NEC

This occupation group covers Journalists and Other Writers not elsewhere classified.

Skill Level: 1

Occupations in this group include:

- Blogger
- Critic
- Editorial Assistant
- Essayist

SUB-MAJOR GROUP 22 BUSINESS, HUMAN RESOURCE AND MARKETING PROFESSIONALS

BUSINESS, HUMAN RESOURCE AND MARKETING PROFESSIONALS perform analytical, conceptual and practical tasks to provide services in financial accounting and transaction matters, human resource development, public relations and marketing, and conduct studies of the economy, organisational structures, methods and systems.

Indicative Skill Level:
Most occupations in this sub-major group have a level of skill commensurate with the qualifications and experience outlined below.

In Australia:

Bachelor degree or higher qualification. At least five years of relevant experience may substitute for the formal qualification (ANZSCO Skill Level 1); or

AQF Associate Degree, Advanced Diploma or Diploma, or at least three years of relevant experience (ANZSCO Skill Level 2)

In New Zealand:

Bachelor degree or higher qualification. At least five years of relevant experience may substitute for the formal qualification (ANZSCO Skill Level 1); or

NZ Register Diploma, or at least three years of relevant experience (ANZSCO Skill Level 2)

In some instances relevant experience and/or on-the-job training may be required in addition to the formal qualification.

Tasks Include:

- collecting, analysing and interpreting information on the financial viability, cost structures and trading effectiveness of organisations
- conducting audits, preparing financial statements and controlling treasury systems for organisations
- developing and reviewing financial plans and strategies, executing buy and sell orders, and negotiating the purchase and sale of commodities
- developing, implementing and evaluating staff recruitment, training and development programs
- researching, developing and implementing marketing and public relations campaigns
- studying and developing methods and policies to improve and promote government and business operations and effectiveness
- developing and managing record and archival systems for retention and destruction of legal, administrative, evidential, historical and other records

Occupations in this sub-major group are classified into the following minor groups:

221 Accountants, Auditors and Company Secretaries
222 Financial Brokers and Dealers, and Investment Advisers
223 Human Resource and Training Professionals
224 Information and Organisation Professionals
225 Sales, Marketing and Public Relations Professionals

MINOR GROUP 221 ACCOUNTANTS, AUDITORS AND COMPANY SECRETARIES

ACCOUNTANTS, AUDITORS AND COMPANY SECRETARIES plan and provide accounting, financial auditing and treasury valuation services and systems to individuals and organisations, and plan and review legislative compliance activities.

Indicative Skill Level:

In Australia and New Zealand:

Most occupations in this minor group have a level of skill commensurate with a bachelor degree or higher qualification. At least five years of relevant experience may substitute for the formal qualification. In some instances relevant experience and/or on-the-job training may be required in addition to the formal qualification (ANZSCO Skill Level 1).

Tasks Include:

- collecting, analysing and interpreting information on the financial status, cost structures and trading effectiveness of organisations
- devising, re-organising and establishing budgetary cost control and other accounting systems such as computer-based systems
- conducting audits and investigations and preparing financial statements and reports for management, shareholders, and statutory and other bodies
- controlling treasury and treasury systems and establishing and reviewing risk management objectives and treasury policies
- arranging and giving notice of meetings of directors and shareholders
- advising organisations' governing boards on matters concerning compliance with stock exchange listing rules, relevant legislation and corporation practice

Occupations in this minor group are classified into the following unit groups:

2211 Accountants
2212 Auditors, Company Secretaries and Corporate Treasurers

UNIT GROUP 2211 ACCOUNTANTS

ACCOUNTANTS plan and provide accounting systems and services relating to taxation and the financial dealings of organisations and individuals, and advise on associated record-keeping and compliance requirements.

Indicative Skill Level:

In Australia and New Zealand:

Most occupations in this unit group have a level of skill commensurate with a bachelor degree or higher qualification. In some instances relevant experience and/or on-the-job training may be required in addition to the formal qualification (ANZSCO Skill Level 1).

Registration or licensing may be required.

Tasks Include:

- assisting in formulating budgetary and accounting policies
- preparing financial statements for presentation to boards of directors, management, shareholders, and governing and statutory bodies
- conducting financial investigations, preparing reports, undertaking audits and advising on matters such as the purchase and sale of businesses, mergers, capital financing, suspected fraud, insolvency and taxation
- examining operating costs and organisations' income and expenditure
- providing assurance about the accuracy of information contained in financial reports and their compliance with statutory requirements
- providing financial and taxation advice on business structures, plans and operations
- preparing taxation returns for individuals and organisations
- liaising with financial institutions and brokers to establish funds management arrangements
- introducing and maintaining accounting systems, and advising on the selection and application of computer-based accounting systems
- maintaining internal control systems
- may appraise cash flow and financial risk of capital investment projects

Occupations:

221111 Accountant (General)
221112 Management Accountant
221113 Taxation Accountant

221111 ACCOUNTANT (GENERAL)

Plans and provides systems and services relating to the financial dealings of organisations and individuals, and advises on associated record-keeping and compliance requirements. Registration or licensing is required.

Skill Level: 1

Specialisations:

Financial Analyst
Insolvency Practitioner

UNIT GROUP 2211 ACCOUNTANTS *continued*

221112 MANAGEMENT ACCOUNTANT

Alternative Titles:

Cost Accountant

Financial Accountant

Plans, reviews and administers accounting systems and procedures, analyses the financial information needs of organisations, provides advice on financial planning and risk management, and provides management with reports to assist in decision-making. May provide insight into cost performance and support the implementation of benchmarking and improvement initiatives. Registration or licensing is required.

Skill Level: 1

221113 TAXATION ACCOUNTANT

Alternative Titles:

Taxation Agent

Taxation Consultant

Analyses, reports and provides advice on taxation issues to organisations or individuals, prepares taxation returns and reports, and handles disputes with taxation authorities. Registration or licensing may be required.

Skill Level: 1

UNIT GROUP 2212 AUDITORS, COMPANY SECRETARIES AND CORPORATE TREASURERS

AUDITORS, COMPANY SECRETARIES AND CORPORATE TREASURERS conduct audits of accounting systems, procedures and financial statements, manage corporate funding and financial risk, and administer and review corporate compliance activities.

Indicative Skill Level:

In Australia and New Zealand:

Most occupations in this unit group have a level of skill commensurate with a bachelor degree or higher qualification. In some instances relevant experience and/or on-the-job training may be required in addition to the formal qualification. In the case of Corporate Treasurers and Company Secretaries, at least five years of relevant experience may substitute for the formal qualification (ANZSCO Skill Level 1).

Registration or licensing may be required.

Tasks Include:

- arranging, giving notice of and attending meetings of directors and shareholders
- advising organisations' governing boards on matters concerning compliance with stock exchange listing rules, relevant legislation and corporation practice
- supervising organisations' share capital by preparing documents and share issues, and handling share transfers
- controlling treasury and treasury systems and establishing and reviewing risk management objectives and treasury policies
- identifying, managing and reporting on financial risks
- assisting with equity management, debt management, securities and taxation planning issues
- collecting, analysing and interpreting information on the financial standing, cost structures and trading effectiveness of organisations
- devising, re-organising and establishing budgetary cost control and other accounting systems such as computer-based systems
- conducting audits and investigations and preparing financial statements and reports for management, shareholders, and governing and statutory bodies
- evaluating the cost effectiveness and risks of operational processes, activities, policies and systems
- reporting to management on the existence and effectiveness of the system of internal controls
- establishing audit objectives, and designing and implementing audit methodologies, processes and audit report criteria

Occupations:

221211 Company Secretary
221212 Corporate Treasurer
221213 External Auditor
221214 Internal Auditor

221211 COMPANY SECRETARY

Plans, administers and reviews corporate compliance activities and effective practice concerning company board meetings and shareholdings, ensuring all business matters and transactions are managed and implemented as directed by the board.

Skill Level: 1

UNIT GROUP 2212 AUDITORS, COMPANY SECRETARIES AND CORPORATE TREASURERS *continued*

221212 CORPORATE TREASURER

Alternative Title:

Financial Risk Manager

Manages corporate funding, liquidity and financial risk associated with the profitable development and operation of an organisation. May be involved in acquisitions, disposals and joint ventures. Registration or licensing may be required.

Skill Level: 1

221213 EXTERNAL AUDITOR

Designs and operates information and reporting systems, procedures and controls to meet external financial reporting requirements. Registration or licensing is required.

Skill Level: 1

221214 INTERNAL AUDITOR

Alternative Title:

Audit Officer

Examines, verifies, evaluates and reports on financial, operational and managerial processes, systems and outcomes to ensure financial and operational integrity and compliance, and assists in business process reviews, risk assessments, developing deliverables and reporting progress against outcomes. Registration or licensing is required.

Skill Level: 1

MINOR GROUP 222 FINANCIAL BROKERS AND DEALERS, AND INVESTMENT ADVISERS

FINANCIAL BROKERS AND DEALERS, AND INVESTMENT ADVISERS conduct financial market transactions on behalf of clients, sell loans and insurance, buy and sell commodities, offer financial investment advice and plans, and develop and manage financial plans for individuals and organisations.

Indicative Skill Level:
Most occupations in this minor group have a level of skill commensurate with the qualifications and experience outlined below.

In Australia:

Bachelor degree or higher qualification. At least five years of relevant experience may substitute for the formal qualification (ANZSCO Skill Level 1); or

AQF Associate Degree, Advanced Diploma or Diploma, or at least three years of relevant experience (ANZSCO Skill Level 2)

In New Zealand:

Bachelor degree or higher qualification. At least five years of relevant experience may substitute for the formal qualification (ANZSCO Skill Level 1); or

NZ Register Diploma, or at least three years of relevant experience (ANZSCO Skill Level 2)

In some instances relevant experience and/or on-the-job training may be required in addition to the formal qualification.

Tasks Include:
- obtaining information on securities, market conditions, government regulations and financial circumstances of clients
- executing buy and sell orders in the market place on behalf of clients, and offering advice on financial matters such as stocks and bonds, market conditions and the history and prospects of corporations
- recording and transmitting buy and sell orders, and calculating and recording costs of transactions
- arranging insurance, home loan mortgages and other types of finance for clients through banks, lenders, financiers and insurance companies
- developing lists of investments for clients, and reviewing and revising investment plans based on modified needs and changes in markets
- negotiating the purchase and sale of commodities such as grains, wool, minerals and metals
- interviewing prospective clients to determine financial status and objectives, discussing their financial options and developing financial plans and strategies
- managing funds raised from personal superannuation saving policies and unit trusts

Occupations in this minor group are classified into the following unit groups:

2221 Financial Brokers
2222 Financial Dealers
2223 Financial Investment Advisers and Managers

UNIT GROUP 2221 FINANCIAL BROKERS

FINANCIAL BROKERS operate as independent agents to facilitate the trading of commodities and arrange insurance and loans of money on behalf of clients.

Indicative Skill Level:
Most occupations in this unit group have a level of skill commensurate with the qualifications and experience outlined below.

In Australia:
AQF Associate Degree, Advanced Diploma or Diploma (ANZSCO Skill Level 2)

In New Zealand:
NZ Register Diploma (ANZSCO Skill Level 2)

At least three years of relevant experience may substitute for the formal qualifications listed above. In some instances relevant experience and/or on-the-job training may be required in addition to the formal qualification.

Registration or licensing may be required.

Tasks Include:

- monitoring commodity prices, trends and other factors affecting the supply and demand for commodities
- negotiating the purchase and sale of commodities such as grains, wool, minerals and metals
- determining the specific financial and insurance requirements of clients, and researching and reviewing available finance and insurance products for suitability to meet clients' requirements
- analysing clients' financial status, discussing financial options and developing financial strategies
- recommending loan combinations that meet clients' needs
- interviewing prospective clients to explain insurance policy conditions, risks covered, premium rates and benefits, and to make recommendations on the amount and type of cover
- arranging insurance, home loan mortgages and other types of finance for clients through banks, lenders, financiers and insurance companies
- preparing documents which set out the conditions of finance, repayments and loan periods
- identifying and advising on significant risk changes to clients' insurance
- broking complex and commercial leases, equipment finance, commercial finance, project finance and finance for property developers

Occupations:
222111 Commodities Trader
222112 Finance Broker
222113 Insurance Broker
222199 Financial Brokers nec

UNIT GROUP 2221 FINANCIAL BROKERS *continued*

222111 COMMODITIES TRADER

Alternative Title:

Commodities Broker

Operates as an independent agent to bring together buyers and sellers of commodities, negotiates private sales and arranges sales through established market places.

Skill Level: 2

Specialisations:

Energy Trader
Grain Buyer
Livestock Trader
Media Buyer
Wool Broker

222112 FINANCE BROKER

Operates as an independent agent in the course of financial negotiations and arranges loans of money on behalf of clients. Registration or licensing is required.

Skill Level: 2

Specialisations:

Lease Broker
Mortgage Broker

222113 INSURANCE BROKER

Operates as an independent agent to sell life, fire, accident, industrial or other forms of insurance for a range of insurance companies. Registration or licensing may be required.

Skill Level: 2

222199 FINANCIAL BROKERS NEC

This occupation group covers Financial Brokers not elsewhere classified. Registration or licensing may be required.

Skill Level: 2

Occupations in this group include:

Investment Broker

UNIT GROUP 2222 FINANCIAL DEALERS

FINANCIAL DEALERS conduct financial market transactions on behalf of clients.

Indicative Skill Level:

In Australia and New Zealand:

Most occupations in this unit group have a level of skill commensurate with a bachelor degree or higher qualification. At least five years of relevant experience may substitute for the formal qualification. In some instances relevant experience and/or on-the-job training may be required in addition to the formal qualification (ANZSCO Skill Level 1).

Registration or licensing may be required.

Tasks Include:

- obtaining information on securities, market conditions, government regulations and financial circumstances of clients
- interpreting data from securities reports, financial periodicals and stock-quotation viewer screens
- analysing financial markets and financial market products
- providing information and offering advice on financial market matters, market conditions and the history and prospects of corporations
- executing buy and sell orders in the market place on behalf of clients
- relaying trade information to clients such as the number of contracts bought and sold and the price
- monitoring futures prices and market changes, and bidding for commodity futures contracts
- recording and transmitting buy and sell orders
- calculating and recording costs of transactions

Occupations:

222211 Financial Market Dealer
222212 Futures Trader
222213 Stockbroking Dealer
222299 Financial Dealers nec

222211 FINANCIAL MARKET DEALER

Alternative Title:

Money Market Dealer

Buys and sells securities within financial markets, and trades and distributes financial securities on behalf of financial institutions. Registration or licensing is required.

Skill Level: 1

Specialisations:

Derivatives Dealer
Fixed Interest Dealer
Foreign Exchange Dealer
Securities Dealer

222212 FUTURES TRADER

Buys and sells commodity futures on behalf of clients. Registration or licensing is required.

Skill Level: 1

UNIT GROUP 2222 FINANCIAL DEALERS *continued*

222213 STOCKBROKING DEALER

Alternative Titles:
- Sharebroker
- Stockbroker

Buys and sells stocks and bonds on behalf of clients. Registration or licensing is required.

Skill Level: 1

Specialisation:
- Trading Floor Operator (Stock Exchange)

222299 FINANCIAL DEALERS NEC

This occupation group covers Financial Dealers not elsewhere classified. Registration or licensing may be required.

Skill Level: 1

Occupations in this group include:
- Equities Analyst
- Investment Dealer

UNIT GROUP 2223 FINANCIAL INVESTMENT ADVISERS AND MANAGERS

FINANCIAL INVESTMENT ADVISERS AND MANAGERS develop financial plans for individuals and organisations, and invest and manage funds on their behalf.

Indicative Skill Level:

In Australia and New Zealand:

Most occupations in this unit group have a level of skill commensurate with a bachelor degree or higher qualification. At least five years of relevant experience may substitute for the formal qualification. In some instances relevant experience and/or on-the-job training may be required in addition to the formal qualification (ANZSCO Skill Level 1).

Registration or licensing may be required.

Tasks Include:

- interviewing prospective clients to determine financial status and objectives, discussing financial options and developing financial plans and investment strategies
- monitoring investment performance, and reviewing and revising investment plans based on modified needs and changes in markets
- recommending and arranging insurance cover for clients
- arranging to buy and sell stocks and bonds for clients
- advising on investment strategies, sources of funds and the distribution of earnings
- setting financial objectives, and developing and implementing strategies for achieving the financial objectives
- managing funds raised from personal superannuation savings policies and unit trusts
- assisting in meeting superannuation compliance requirements
- directing the collection of financial, accounting and investment information and the preparation of budgets, reports, forecasts and statutory returns
- may refer clients to other organisations to obtain services outlined in financial plans

Occupations:

222311 Financial Investment Adviser
222312 Financial Investment Manager

222311 FINANCIAL INVESTMENT ADVISER

Alternative Title:

Financial Planning Adviser

Develops and implements financial plans for individuals or organisations, and advises on investment strategies and their taxation implications, securities, insurance, pension plans and real estate. Registration or licensing may be required.

Skill Level: 1

UNIT GROUP 2223 FINANCIAL INVESTMENT ADVISERS AND MANAGERS *continued*

222312 FINANCIAL INVESTMENT MANAGER

Alternative Title:

Portfolio Manager

Invests and manages sums of money and assets on behalf of others over an agreed period of time, in order to generate income and profit. Registration or licensing may be required.

Skill Level: 1

Specialisations:

Superannuation Funds Manager

Unit Trust Manager

MINOR GROUP 223 HUMAN RESOURCE AND TRAINING PROFESSIONALS

HUMAN RESOURCE AND TRAINING PROFESSIONALS plan, develop, implement and evaluate staff recruitment, retention, training and development programs, assist in resolving disputes by advising on workplace relations policies and problems, and represent industrial, commercial, union, employer and other parties in negotiations.

Indicative Skill Level:

In Australia and New Zealand:

Most occupations in this minor group have a level of skill commensurate with a bachelor degree or higher qualification. At least five years of relevant experience may substitute for the formal qualification. In some instances relevant experience and/or on-the-job training may be required in addition to the formal qualification (ANZSCO Skill Level 1).

Tasks Include:

- arranging for advertising of job vacancies, interviewing and testing of applicants, and selection of staff
- providing advice and information to management on human resource policies and procedures, staff performance and misconduct matters
- undertaking negotiations on terms and conditions of employment, and examining and resolving disputes and grievances
- studying and interpreting legislation, awards and agreements, wage payment systems, and dispute settlement procedures
- developing, planning and formulating workplace relations policies and programs, and procedures for their implementation
- identifying and determining the need for training, through identifying competencies and defining training requirements in the work environment
- designing, developing and assessing staff training and development

Occupations in this minor group are classified into the following unit groups:

2231 Human Resource Professionals

2232 ICT Trainers

2233 Training and Development Professionals

UNIT GROUP 2231 HUMAN RESOURCE PROFESSIONALS

HUMAN RESOURCE PROFESSIONALS plan, develop, implement and evaluate staff recruitment, assist in resolving disputes by advising on workplace matters, and represent industrial, commercial, union, employer and other parties in negotiations on issues such as enterprise bargaining, rates of pay and conditions of employment.

Indicative Skill Level:
In Australia and New Zealand:

Most occupations in this unit group have a level of skill commensurate with a bachelor degree or higher qualification. At least five years of relevant experience may substitute for the formal qualification. In some instances relevant experience and/or on-the-job training may be required in addition to the formal qualification (ANZSCO Skill Level 1).

Tasks Include:

- arranging for advertising of job vacancies, interviewing and testing of applicants, and selection of staff
- maintaining personnel records and associated human resource information systems
- providing advice and information to management on workplace relations policies and procedures, staff performance and disciplinary matters
- arranging the induction of staff and providing information on conditions of service, salaries and promotional opportunities
- receiving and recording job vacancy information from employers such as details about job description, wages and conditions of employment
- providing information on current job vacancies in the organisation to employers and job seekers
- undertaking negotiations on terms and conditions of employment, and examining and resolving disputes and grievances
- studying and interpreting legislation, awards, collective agreements and employment contracts, wage payment systems and dispute settlement procedures
- developing, planning and formulating enterprise agreements or collective contracts such as productivity-based wage adjustment procedures, workplace relations policies and programs, and procedures for their implementation
- overseeing the formation and conduct of workplace consultative committees and employee participation initiatives

Occupations:

223111 Human Resource Adviser
223112 Recruitment Consultant
223113 Workplace Relations Adviser

223111 HUMAN RESOURCE ADVISER

Alternative Titles:

Human Resource Consultant
Personnel Officer

Provides staffing and personnel administration services in support of an organisation's human resource policies and programs.

Skill Level: 1

Specialisation:

Workforce Planning Analyst

UNIT GROUP 2231 HUMAN RESOURCE PROFESSIONALS *continued*

223112 RECRUITMENT CONSULTANT

Alternative Title:

Employment Consultant (Aus)

Interviews applicants to determine their job requirements and suitability for particular jobs, and assists employers to find suitable staff.

Skill Level: 1

Specialisations:

Casting Agent
Literary Agent

223113 WORKPLACE RELATIONS ADVISER

Alternative Title:

Industrial Relations Officer

Assists in resolving disputes by advising on workplace relations policies and problems, and representing industrial, commercial, union, employer or other parties in negotiations on rates of pay and conditions of employment.

Skill Level: 1

Specialisations:

Trade Union Official
Union Organiser

UNIT GROUP 2232 ICT TRAINERS

ICT TRAINERS analyse and evaluate information-based system training needs and objectives, and develop, schedule and conduct ICT-based system training programs and courses.

Indicative Skill Level:

In Australia and New Zealand:

Most occupations in this unit group have a level of skill commensurate with a bachelor degree or higher qualification. At least five years of relevant experience and/or relevant vendor certification may substitute for the formal qualification. In some instances relevant experience and/or on-the-job training may be required in addition to the formal qualification (ANZSCO Skill Level 1).

Tasks Include:

- identifying technical training needs and requirements of individuals and organisations
- setting human resource development objectives and evaluating learning outcomes
- preparing and developing instructional training material and aids such as handbooks, visual aids, online tutorials, demonstration models, and supporting training reference documentation
- designing, coordinating, scheduling and conducting ICT training and development programs that can be delivered in the form of individual and group instruction, and facilitating workshops, meetings, demonstrations and conferences
- liaising with external training providers to arrange delivery of specific training and development programs
- promoting internal and external training and development, and evaluating these promotional activities
- monitoring and performing ongoing evaluation and assessment of training quality and effectiveness, and reviewing and modifying training objectives, methods and course deliverables
- gathering, investigating and researching background materials to gain a full understanding of the ICT subject matter and systems
- keeping up-to-date with new product version releases, advances in programming languages, application development software, and general information technology trends
- writing end user products and materials such as user training, tutorial and instruction manuals, online help, and operating and maintenance instructions

Occupation:

223211 ICT Trainer

223211 ICT TRAINER

Alternative Title:

ICT Educator

Analyses and evaluates information-based system training needs and objectives, and develops, schedules and conducts ICT-based system training programs and courses.

Skill Level: 1

Specialisation:

Software Trainer

UNIT GROUP 2233 TRAINING AND DEVELOPMENT PROFESSIONALS

TRAINING AND DEVELOPMENT PROFESSIONALS plan, develop, implement and evaluate training and development programs to ensure management and staff acquire the skills and develop the competencies required by organisations to meet organisational objectives.

Indicative Skill Level:

In Australia and New Zealand:

Most occupations in this unit group have a level of skill commensurate with a bachelor degree or higher qualification. At least five years of relevant experience may substitute for the formal qualification. In some instances relevant experience and/or on-the-job training may be required in addition to the formal qualification (ANZSCO Skill Level 1).

Tasks Include:

- identifying training needs and requirements of individuals and organisations
- setting human resource development objectives and evaluating learning outcomes
- preparing and developing instructional training material and aids such as handbooks, visual aids, online tutorials, demonstration models, and supporting training reference documentation
- designing, coordinating, scheduling and conducting training and development programs that can be delivered in the form of individual and group instruction, and facilitating workshops, meetings, demonstrations and conferences
- liaising with external training providers to arrange delivery of specific training and development programs
- promoting internal and external training and development, and evaluating these promotional activities
- monitoring and performing ongoing evaluation and assessment of training quality and effectiveness, and reviewing and modifying training objectives, methods and course deliverables
- gathering, investigating and researching background materials to gain an understanding of various subject matters and systems
- advising management on the development and placement of staff, and providing career counselling for employees

Occupation:

223311 Training and Development Professional

223311 TRAINING AND DEVELOPMENT PROFESSIONAL

Alternative Title:

Training Officer

Plans, develops, implements and evaluates training and development programs to ensure management and staff acquire the skills and develop the competencies required by an organisation to meet organisational objectives.

Skill Level: 1

Specialisations:

Education Officer (Air Force and Army)

Training Systems Officer (Navy)

MINOR GROUP 224 INFORMATION AND ORGANISATION PROFESSIONALS

INFORMATION AND ORGANISATION PROFESSIONALS support organisations, government, individuals and the community by analysing, organising and managing information and data, and by providing advice on policy, business and organisational methods, and the value of property and other items.

Indicative Skill Level:

In Australia and New Zealand:

Most occupations in this minor group have a level of skill commensurate with a bachelor degree or higher qualification. At least five years of relevant experience may substitute for the formal qualification. In some instances relevant experience and/or on-the-job training may be required in addition to the formal qualification (ANZSCO Skill Level 1).

Tasks Include:

- applying mathematical, statistical and actuarial principles and techniques to a range of tasks
- developing, organising and maintaining libraries and other information keeping services
- reviewing and analysing economic data and preparing reports
- managing the collection and processing of information and data to produce intelligence, and analysing and advising on policy options
- assessing the value of land, property and other items, and providing advice on the administration, and commercial and operational use of land and property
- studying organisational structures and methods to solve organisational problems and achieve greater efficiency

Occupations in this minor group are classified into the following unit groups:

2241 Actuaries, Mathematicians and Statisticians
2242 Archivists, Curators and Records Managers
2243 Economists
2244 Intelligence and Policy Analysts
2245 Land Economists and Valuers
2246 Librarians
2247 Management and Organisation Analysts
2249 Other Information and Organisation Professionals

UNIT GROUP 2241 ACTUARIES, MATHEMATICIANS AND STATISTICIANS

ACTUARIES, MATHEMATICIANS AND STATISTICIANS develop and apply actuarial, mathematical, statistical and quantitative principles and techniques to solve problems in a range of fields such as business and finance, scientific and social research, and engineering.

Econometricians are excluded from this unit group. Econometricians are included in Unit Group 2243 Economists.

Indicative Skill Level:

In Australia and New Zealand:

Most occupations in this unit group have a level of skill commensurate with a bachelor degree or higher qualification. In some instances relevant experience and/or on-the-job training may be required in addition to the formal qualification (ANZSCO Skill Level 1).

Registration or licensing may be required.

Tasks Include:

- defining, analysing and solving complex financial and business problems relating to areas such as insurance premiums, annuities, superannuation funds, pensions and dividends
- examining financial projections for general insurance companies, finance companies, government and other organisations
- designing new types of policies, assessing risks and analysing investments in life insurance, superannuation funds, health insurance, friendly societies, financial markets and other areas
- formulating mathematical models to simulate processes
- applying models to experimental observations, and adjusting and recasting the models
- using numerical analysis methods to develop algorithms and perform computations
- liaising with management and clients to determine the subject or area to be surveyed or examined
- specifying the data to be collected, and the methodology to be used in collection and analysis
- evaluating and describing the reliability and utility of source information
- analysing and interpreting data, and producing relevant statistics to describe and infer particular trends and patterns

Occupations:

224111 Actuary
224112 Mathematician
224113 Statistician

224111 ACTUARY

Analyses mathematical, statistical, demographic, financial or economic data to predict and assess the long-term risk involved in financial decisions and planning. Registration or licensing is required.

Skill Level: 1

224112 MATHEMATICIAN

Develops and applies mathematical principles and techniques to solve problems in all areas of the sciences, engineering, technology, social sciences, business, industry and commerce.

Skill Level: 1

Specialisation:

Operations Research Analyst

UNIT GROUP 2241 ACTUARIES, MATHEMATICIANS AND STATISTICIANS *continued*

224113 STATISTICIAN

Designs and applies statistical principles and techniques for collecting, organising and interpreting quantifiable data, and uses statistical methodologies to produce statistical reports and analyses for government, commercial and other purposes.

Skill Level: 1

Specialisations:
- Biometrician
- Demographer
- Epidemiologist

UNIT GROUP 2242 ARCHIVISTS, CURATORS AND RECORDS MANAGERS

ARCHIVISTS, CURATORS AND RECORDS MANAGERS develop, maintain, implement and deliver systems for keeping, updating, accessing and preserving records, files, information, historical documents and artefacts.

Indicative Skill Level:

In Australia and New Zealand:

Most occupations in this unit group have a level of skill commensurate with a bachelor degree or higher qualification. At least five years of relevant experience may substitute for the formal qualification. In some instances relevant experience and/or on-the-job training may be required in addition to the formal qualification (ANZSCO Skill Level 1).

Registration or licensing may be required.

Tasks Include:

- evaluating and preserving records for administrative, historical, legal, evidential and other purposes
- preparing record-keeping systems, indexes, guides and procedures for archival research and for the retention and destruction of records
- identifying and classifying specimens and objects, and arranging restoration work
- examining items and arranging examinations to determine condition and authenticity
- designing and revising medical record forms
- managing organisations' central records systems
- analysing the record-keeping needs of organisations, and translating these needs into record management systems
- maintaining computerised and other record management systems and record forms, and advising on their usage
- controlling access to confidential information, and recommending codes of practice and procedures for accessing records
- developing record cataloguing, coding and classification systems, and monitoring their use

Occupations:

224211 Archivist
224212 Gallery or Museum Curator
224213 Health Information Manager
224214 Records Manager

224211 ARCHIVIST

Analyses and documents records, and plans and organises systems and procedures for the safekeeping of records and historically valuable documents.

Skill Level: 1

Specialisations:

Film Archivist
Legal Archivist
Manuscripts Archivist
Parliamentary Archivist

UNIT GROUP 2242 ARCHIVISTS, CURATORS AND RECORDS MANAGERS *continued*

224212 GALLERY OR MUSEUM CURATOR

Plans and organises a gallery or museum collection by drafting collection policies and arranging acquisitions of pieces. Registration or licensing may be required.

Skill Level: 1

224213 HEALTH INFORMATION MANAGER

Alternative Title:
Medical Records Administrator

Plans, develops, implements and manages health information services, such as patient information systems, and clinical and administrative data, to meet the medical, legal, ethical and administrative requirements of health care delivery. Registration or licensing may be required.

Skill Level: 1

Specialisations:
Casemix Coordinator
Clinical Trial Data Manager
Health Data Administrator

224214 RECORDS MANAGER

Designs, implements and administers record systems and related information services, to support efficient access, movement, updating, storage, retention and disposal of files and other organisational records.

Skill Level: 1

Specialisation:
Freedom of Information Officer

UNIT GROUP 2243 ECONOMISTS

ECONOMISTS perform economic research and analysis, develop and apply theories about production and distribution of goods and services and people's spending and financial behaviour, and provide advice to governments and organisations on economic policy issues.

Statisticians are excluded from this unit group. Statisticians are included in Unit Group 2241 Actuaries, Mathematicians and Statisticians.

Indicative Skill Level:
In Australia and New Zealand:
Most occupations in this unit group have a level of skill commensurate with a bachelor degree or higher qualification (ANZSCO Skill Level 1).

Tasks Include:
- analysing interrelationships between economic variables and studying the effects of government fiscal and monetary policies, expenditure, taxation and other budgetary policies on the economy and the community
- researching, analysing and assessing the effects of labour market programs and industry policies and programs on economic growth, welfare, education and training
- investigating international and national economic situations, and particular features such as industries, regions and socioeconomic groups
- studying workplace issues such as enterprise bargaining and wage fixation, and the effect of workplace policies on productivity and economic growth
- analysing trends and advising on economic issues such as taxation levels, prices, employment and unemployment, imports and exports, and interest and exchange rates
- forecasting changes in the economic environment for short-term budgeting, long-term planning and investment evaluation
- formulating recommendations, policies and plans for the economy, corporate strategies and investment, and undertaking feasibility studies for projects
- preparing reports on research findings

Occupation:
224311 Economist

224311 ECONOMIST

Alternative Title:
Economic Analyst

Performs economic research and analysis, develops and applies theories about production and distribution of goods and services and people's spending and financial behaviour, and provides advice to governments and organisations on economic policy issues.

Skill Level: 1

Specialisations:
Agricultural Economist
Econometrician
Economic Forecaster
Environmental Economist
Health Economist
Labour Market Economist
Mineral Economist
Taxation Economist

UNIT GROUP 2244 INTELLIGENCE AND POLICY ANALYSTS

INTELLIGENCE AND POLICY ANALYSTS collect and analyse information and data to produce intelligence and to develop and analyse policies guiding the design, implementation and modification of government and commercial operations and programs.

Indicative Skill Level:

In Australia and New Zealand:

Most occupations in this unit group have a level of skill commensurate with a bachelor degree or higher qualification. At least five years of relevant experience may substitute for the formal qualification. In some instances relevant experience and/or on-the-job training may be required in addition to the formal qualification (ANZSCO Skill Level 1).

Tasks Include:

- determining organisational and client intelligence requirements
- organising, collecting, collating and analysing data, and developing intelligence information such as electronic surveillance
- compiling and disseminating intelligence information using briefings, maps, charts, reports and other methods
- ascertaining the accuracy of data collected and reliability of sources
- conducting threat and risk assessments and developing responses
- liaising and consulting with program administrators and other interested parties to identify policy needs
- reviewing existing policies and legislation to identify anomalies and out-of-date provisions
- researching social, economic and industrial trends, and client expectations of programs and services provided
- formulating and analysing policy options, preparing briefing papers and recommendations for policy changes, and advising on preferred options
- assessing impacts, financial implications, interactions with other programs and political and administrative feasibility of policies

Occupations:

224411 Intelligence Officer
224412 Policy Analyst

224411 INTELLIGENCE OFFICER

Collects and analyses information and data to produce intelligence for an organisation to support planning, operations and human resource functions.

Skill Level: 1

Specialisations:

Criminal Intelligence Analyst
Defence Intelligence Analyst

UNIT GROUP 2244 INTELLIGENCE AND POLICY ANALYSTS *continued*

224412 POLICY ANALYST

Alternative Title:

Policy Adviser

Develops and analyses policies guiding the design, implementation and modification of government or commercial operations and programs.

Skill Level: 1

Specialisation:

Research and Evaluation Analyst (NZ)

UNIT GROUP 2245 LAND ECONOMISTS AND VALUERS

LAND ECONOMISTS AND VALUERS provide advice on the administration and use of land and property, and assess the value of land, property and other items such as commercial equipment and objects of art.

Indicative Skill Level:

In Australia and New Zealand:

Most occupations in this unit group have a level of skill commensurate with a bachelor degree or higher qualification. At least five years of relevant experience may substitute for the formal qualification. In some instances relevant experience and/or on-the-job training may be required in addition to the formal qualification (ANZSCO Skill Level 1).

Registration or licensing may be required.

Tasks Include:

- providing advice on land and property financing and valuation matters
- researching and advising on the administration and use of land and property
- developing and implementing sales and leasing proposals for commercial land and property
- providing asset management services for the administration and use of land and property
- analysing land and property investments
- managing land and property portfolios and commercial property developments
- calculating values by considering market demand, condition of items, future trends and other factors
- examining property, selecting methods of valuation, and submitting written assessments
- giving evidence in legal proceedings, mediating on valuation matters and providing rental determinations for arbitration purposes

Occupations:

224511 Land Economist
224512 Valuer

224511 LAND ECONOMIST

Alternative Title:

Property Economist

Provides advice on the administration and use of land and property.

Skill Level: 1

Specialisation:

Asset Manager (Land and Property)

224512 VALUER

Assesses the value of land, property, commercial equipment, merchandise, personal effects, household goods and objects of art. Registration or licensing may be required.

Skill Level: 1

Specialisations:

Plant and Machinery Valuer
Property Valuer
Real Estate Valuer

UNIT GROUP 2246 LIBRARIANS

LIBRARIANS develop, organise and manage library services such as collections of information, recreational resources and reader information services.

Teacher-Librarians are excluded from this unit group. Teacher-Librarians are included in Minor Group 241 School Teachers.

Indicative Skill Level:
In Australia and New Zealand:
Most occupations in this unit group have a level of skill commensurate with a bachelor degree or higher qualification. In some instances relevant experience and/or on-the-job training may be required in addition to the formal qualification (ANZSCO Skill Level 1).

Registration or licensing may be required.

Tasks Include:
- developing and implementing library and information policies and services
- examining publications and materials, interviewing publishers' representatives, and consulting with others to select library materials
- reviewing, evaluating and modifying services in response to user needs
- providing assistance to clients in accessing library resources
- managing library systems for recording and organising library holdings, acquisitions and purchases, reader registrations and loan transactions, and supervising indexing, filing and retrieval activities
- managing inter-library loan systems and information networks
- undertaking information research activities on behalf of clients
- selecting, ordering, classifying and cataloguing library and information resources
- monitoring collection development and culling programs
- supervising and training other library staff
- may plan and direct library promotion and outreach activities

Occupation:
224611 Librarian

224611 LIBRARIAN

Develops, organises and manages library services such as collections of information, recreational resources and reader information services. Registration or licensing may be required.

Skill Level: 1

Specialisations:
Acquisitions Librarian
Audiovisual Librarian
Bibliographer
Cataloguer
Children's Librarian
Corporate Librarian
Legal Librarian
Multicultural Services Librarian
Parliamentary Librarian
Reference Librarian
Special Librarian
Special Needs Librarian

UNIT GROUP 2247 MANAGEMENT AND ORGANISATION ANALYSTS

MANAGEMENT AND ORGANISATION ANALYSTS assist organisations to achieve greater efficiency and solve organisational problems, and study organisational structures, methods, systems and procedures.

ICT Business Analysts are excluded from this unit group. ICT Business Analysts are included in Unit Group 2611 ICT Business and Systems Analysts.

Indicative Skill Level:
In Australia and New Zealand:

Most occupations in this unit group have a level of skill commensurate with a bachelor degree or higher qualification. At least five years of relevant experience may substitute for the formal qualification. In some instances relevant experience and/or on-the-job training may be required in addition to the formal qualification (ANZSCO Skill Level 1).

Tasks Include:

- assisting and encouraging the development of objectives, strategies and plans aimed at achieving customer satisfaction and the efficient use of organisations' resources
- discussing business and organisational shortcomings with clients
- analysing and evaluating current systems and structures
- discussing current systems with staff and observing systems at all levels of organisation
- directing clients towards more efficient organisation and developing solutions to organisational problems
- undertaking and reviewing work studies by analysing existing and proposed methods and procedures such as administrative and clerical procedures
- recording and analysing organisations' work flow charts, records, reports, manuals and job descriptions
- preparing and recommending proposals to revise methods and procedures, alter work flows, redefine job functions and resolve organisational problems
- assisting in implementing approved recommendations, issuing revised instructions and procedure manuals, and drafting other documentation
- reviewing operating procedures and advising of departures from procedures and standards

Occupations:

224711 Management Consultant
224712 Organisation and Methods Analyst

224711 MANAGEMENT CONSULTANT

Alternative Title:

Business Consultant

Assists organisations to achieve greater efficiency and solve organisational problems.

Skill Level: 1

Specialisation:

Business Analyst

UNIT GROUP 2247 MANAGEMENT AND ORGANISATION ANALYSTS *continued*

224712 ORGANISATION AND METHODS ANALYST

Alternative Title:

Procedures Analyst

Studies organisational structures, methods, systems and procedures.

Skill Level: 1

Specialisations:

Change Management Facilitator
Industry Analyst
Quality Auditor
Skills Auditor

UNIT GROUP 2249 OTHER INFORMATION AND ORGANISATION PROFESSIONALS

This unit group covers Information and Organisation Professionals not elsewhere classified.

It includes Electorate Officers, Liaison Officers, Migration Agents (Aus) / Immigration Consultants (NZ) and Patents Examiners.

Indicative Skill Level:
In Australia and New Zealand:

Most occupations in this unit group have a level of skill commensurate with a bachelor degree or higher qualification. At least five years of relevant experience may substitute for the formal qualification. In some instances relevant experience and/or on-the-job training may be required in addition to the formal qualification (ANZSCO Skill Level 1).

Registration or licensing may be required.

Occupations:

224911 Electorate Officer
224912 Liaison Officer
224913 Migration Agent (Aus) / Immigration Consultant (NZ)
224914 Patents Examiner
224999 Information and Organisation Professionals nec

224911 ELECTORATE OFFICER

Manages the electorate office of a politician, and liaises with constituents and the media on their behalf.

Skill Level: 1

224912 LIAISON OFFICER

Establishes and facilitates communication between different community groups, organisations and governments.

Skill Level: 1

Specialisations:

Aboriginal Liaison Officer
Business Liaison Officer
Community Liaison Officer
Disability Liaison Officer
Police Liaison Officer

224913 MIGRATION AGENT (AUS) / IMMIGRATION CONSULTANT (NZ)

Alternative Title:

Migration Consultant

Provides information and advice to potential migrants, prepares and lodges visa applications, and acts as an intermediary to legally represent clients during visa processing and before review bodies. Liaises with Legal Professionals in relation to judicial review matters. Registration or licensing may be required.

Skill Level: 1

UNIT GROUP 2249 OTHER INFORMATION AND ORGANISATION PROFESSIONALS *continued*

224914 PATENTS EXAMINER

Investigates and reports on patent applications to assess their compliance with the requirements of the Patents Act. Registration or licensing may be required.

Skill Level: 1

224999 INFORMATION AND ORGANISATION PROFESSIONALS NEC

This occupation group includes Information and Organisation Professionals not elsewhere classified.

Skill Level: 1

Occupations in this group include:

- Electoral Officer
- Forms Designer
- Knowledge Manager
- Lobbyist
- Museum Registrar

MINOR GROUP 225 SALES, MARKETING AND PUBLIC RELATIONS PROFESSIONALS

SALES, MARKETING AND PUBLIC RELATIONS PROFESSIONALS plan, develop, coordinate and implement programs of information dissemination to promote organisations, goods and services, and represent companies in selling a range of technical, industrial, medical, pharmaceutical and ICT goods and services.

Indicative Skill Level:

In Australia and New Zealand:

Most occupations in this minor group have a level of skill commensurate with a bachelor degree or higher qualification. At least five years of relevant experience may substitute for the formal qualification. In some instances relevant experience and/or on-the-job training may be required in addition to the formal qualification (ANZSCO Skill Level 1).

Tasks Include:

- commissioning and undertaking market research, analysing the findings, and planning advertising, marketing and public relations activities
- supporting business growth and development through the preparation and execution of marketing objectives, policies and programs
- planning and organising publicity campaigns
- appraising and selecting material submitted by writers, Photographers, Illustrators and others to create favourable publicity
- acquiring and updating knowledge of employers' and competitors' goods and services, and market conditions
- assessing customers' needs and explaining and demonstrating goods and services to them
- visiting regular and prospective client businesses to establish and act on marketing opportunities
- quoting and negotiating prices and credit terms, and completing contracts

Occupations in this minor group are classified into the following unit groups:

2251 Advertising and Marketing Professionals
2252 ICT Sales Professionals
2253 Public Relations Professionals
2254 Technical Sales Representatives

UNIT GROUP 2251 ADVERTISING AND MARKETING PROFESSIONALS

ADVERTISING AND MARKETING PROFESSIONALS develop and coordinate advertising strategies and campaigns, determine the market for new goods and services, and identify and develop market opportunities for new and existing goods and services.

Indicative Skill Level:

In Australia and New Zealand:

Most occupations in this unit group have a level of skill commensurate with a bachelor degree or higher qualification. At least five years of relevant experience may substitute for the formal qualification. In some instances relevant experience and/or on-the-job training may be required in addition to the formal qualification (ANZSCO Skill Level 1).

Tasks Include:

- planning, developing and organising advertising policies and campaigns to support sales objectives
- advising executives and clients on advertising strategies and campaigns to reach target markets, creating consumer awareness and effectively promoting the attributes of goods and services
- coordinating production of advertising campaigns involving specialised activities, such as artwork, copywriting, media scripting, television and film production and media placement, within time and budget constraints
- analysing data regarding consumer patterns and preferences
- interpreting and predicting current and future consumer trends
- researching potential demand and market characteristics for new goods and services and collecting and analysing data and other statistical information
- supporting business growth and development through the preparation and execution of marketing objectives, policies and programs
- commissioning and undertaking market research to identify market opportunities for new and existing goods and services
- advising on all elements of marketing such as product mix, pricing, advertising and sales promotion, selling, and distribution channels

Occupations:

225111 Advertising Specialist
225112 Market Research Analyst
225113 Marketing Specialist

225111 ADVERTISING SPECIALIST

Alternative Titles:

Advertising Account Executive
Advertising Account Manager
Creative Director (Advertising)

Devises and coordinates advertising campaigns which encourage consumers to purchase particular goods or services.

Skill Level: 1

UNIT GROUP 2251 ADVERTISING AND MARKETING PROFESSIONALS *continued*

225112 MARKET RESEARCH ANALYST

Determines the market for new goods and services, develops advertising strategies, and evaluates the best business sites for commercial organisations.

Skill Level: 1

225113 MARKETING SPECIALIST

Alternative Titles:

- Marketing Consultant
- Marketing Coordinator
- Marketing Officer

Identifies market opportunities and advises on the development, coordination and implementation of plans for pricing and promoting an organisation's goods and services.

Skill Level: 1

Specialisations:

- Brand Manager
- Category Manager
- Product Manager
- Sales Promotion Officer

UNIT GROUP 2252 ICT SALES PROFESSIONALS

ICT SALES PROFESSIONALS manage client accounts and represent companies in selling a range of computer hardware, software and other ICT goods and services to industrial, business, professional and other organisations.

Indicative Skill Level:

In Australia and New Zealand:

Most occupations in this unit group have a level of skill commensurate with a bachelor degree or higher qualification. At least five years of relevant experience and/or relevant vendor certification may substitute for the formal qualification. In some instances relevant experience and/or on-the-job training may be required in addition to the formal qualification (ANZSCO Skill Level 1).

Tasks Include:

- compiling lists of prospective client businesses using trade directories and other sources
- acquiring and updating knowledge of employer's and competitors' goods and services, and market conditions
- visiting regular and prospective client businesses to establish and act on selling opportunities
- assessing customers' needs and explaining the goods and services which meet their needs
- promoting employers' ICT goods and services to existing and prospective clients
- quoting and negotiating prices and credit terms, and completing contracts and recording orders
- arranging delivery of goods, installation of equipment and the provision of services
- reporting to sales management on sales made and the marketability of ICT goods and services
- following up with clients to ensure satisfaction with ICT goods and services purchased, arranging modifications and resolving any problems arising
- preparing sales reports, and maintaining and submitting records of business expenses incurred

Occupations:

225211 ICT Account Manager
225212 ICT Business Development Manager
225213 ICT Sales Representative

225211 ICT ACCOUNT MANAGER

Manages sale of computer hardware, software and services to existing account clients and identifies further sales opportunities within these accounts, builds new account clients, manages customer satisfaction and retention, and coordinates the preparation and presentation of ICT sales proposals and tenders.

Skill Level: 1

225212 ICT BUSINESS DEVELOPMENT MANAGER

Identifies and generates new ICT business opportunities to further improve market share and awareness by gaining an understanding of customers' ICT needs and promoting goods and services to these customers. May manage some key customer accounts.

Skill Level: 1

225213 ICT SALES REPRESENTATIVE

Develops and converts sales opportunities into sales of computer hardware, software and ICT services.

Skill Level: 1

UNIT GROUP 2253 PUBLIC RELATIONS PROFESSIONALS

PUBLIC RELATIONS PROFESSIONALS plan, develop, implement and evaluate information and communication strategies that create an understanding and a favourable view of organisations, their goods and services, and their role in the community.

Indicative Skill Level:

In Australia and New Zealand:

Most occupations in this unit group have a level of skill commensurate with a bachelor degree or higher qualification. At least five years of relevant experience may substitute for the formal qualification. In some instances relevant experience and/or on-the-job training may be required in addition to the formal qualification (ANZSCO Skill Level 1).

Tasks Include:

- planning and organising publicity campaigns and communication strategies
- advising executives on the public relations implications of their policies, programs and practices
- preparing and controlling the issue of news and press releases
- undertaking and commissioning public opinion research, analysing the findings and planning public relations and promotional campaigns
- organising special events, seminars, entertainment, competitions and social functions to promote goodwill and favourable publicity
- representing organisations and arranging executive interviews with publicity media
- attending business, social and other functions to promote the organisation
- commissioning and obtaining photographs and other illustrative material
- selecting, appraising and revising material submitted by publicity writers, Photographers, Illustrators and others to create favourable publicity

Occupation:

225311 Public Relations Professional

225311 PUBLIC RELATIONS PROFESSIONAL

Alternative Title:

Corporate Affairs Officer

Plans, develops, implements and evaluates information and communication strategies that create an understanding and a favourable view of an organisation, its goods and services, and its role in the community.

Skill Level: 1

Specialisations:

Māori Liaison Officer (NZ)
Media Liaison Officer
Press Officer
Promotions Officer
Public Affairs Officer
Public Relations Consultant
Public Relations Officer

UNIT GROUP 2254 TECHNICAL SALES REPRESENTATIVES

TECHNICAL SALES REPRESENTATIVES represent companies in selling a range of industrial, medical and pharmaceutical goods and services to industrial, business, professional and other establishments.

Indicative Skill Level:
In Australia and New Zealand:
Most occupations in this unit group have a level of skill commensurate with a bachelor degree or higher qualification. At least five years of relevant experience may substitute for the formal qualification. In some instances relevant experience and/or on-the-job training may be required in addition to the formal qualification (ANZSCO Skill Level 1).

Tasks Include:
- compiling lists of prospective client businesses using directories and other sources
- acquiring and updating knowledge of employers' and competitors' goods and services, and market conditions
- visiting regular and prospective client businesses to establish and act on selling opportunities
- assessing customers' needs and recommending and explaining goods and services to them
- monitoring customers' changing needs and competitor activity, and reporting these developments to sales management
- quoting and negotiating prices and credit terms, and completing contracts and recording orders
- arranging delivery of goods, installation of equipment and the provision of services
- reporting to sales management on sales made and the marketability of goods and services
- following up with clients to ensure satisfaction with goods and services purchased, and resolving any problems arising
- preparing sales reports and maintaining and submitting records of business expenses incurred

Occupations:
225411 Sales Representative (Industrial Products)
225412 Sales Representative (Medical and Pharmaceutical Products)
225499 Technical Sales Representatives nec

225411 SALES REPRESENTATIVE (INDUSTRIAL PRODUCTS)

Represents companies in selling a range of specialised chemicals, machines, manufacturing materials and other industrial supplies.

Skill Level: 1

225412 SALES REPRESENTATIVE (MEDICAL AND PHARMACEUTICAL PRODUCTS)

Represents companies in selling medical, dental and veterinary equipment and supplies, and pharmaceutical products.

Skill Level: 1

Specialisation:
Medical Representative

225499 TECHNICAL SALES REPRESENTATIVES NEC

This occupation group covers Technical Sales Representatives not elsewhere classified.

Skill Level: 1

Occupations in this group include:
Sales Representative (Educational Products and Services)

SUB-MAJOR GROUP 23 DESIGN, ENGINEERING, SCIENCE AND TRANSPORT PROFESSIONALS

DESIGN, ENGINEERING, SCIENCE AND TRANSPORT PROFESSIONALS fly and ensure the safe operation of aircraft, control and manage the operation of ships, boats and marine equipment, design buildings, landscapes and products for manufacture and visual communication, design, plan and organise the testing, construction and maintenance of structures, machines, production systems and plants, and perform analytical, conceptual and practical tasks in relation to the chemical and physical properties of the universe, living organisms, and the environment.

Indicative Skill Level:
In Australia and New Zealand:

Most occupations in this sub-major group have a level of skill commensurate with a bachelor degree or higher qualification. In some instances relevant experience and/or on-the-job training may be required in addition to the formal qualification (ANZSCO Skill Level 1).

Tasks Include:

- flying aircraft, and ensuring the safe and efficient operation of aircraft in flight and on the ground
- controlling and directing the operation of ships, boats and marine equipment to ensure the safe and efficient loading and transport of cargo and passengers
- designing products including furniture, textiles and maps, and physical structures and engineering systems
- conducting research and practical tests to resolve design and operational problems
- interpreting and analysing data from engineering and scientific experiments and tests
- conducting experiments and tests to determine the chemical composition and reactive properties of natural substances and processed materials
- analysing and describing living organisms and their interaction with their environment
- advising on the use of natural resources, and discussing and making recommendations to individuals and groups about variables affecting land use

Occupations in this sub-major group are classified into the following minor groups:

231 Air and Marine Transport Professionals
232 Architects, Designers, Planners and Surveyors
233 Engineering Professionals
234 Natural and Physical Science Professionals

MINOR GROUP 231 AIR AND MARINE TRANSPORT PROFESSIONALS

AIR AND MARINE TRANSPORT PROFESSIONALS fly and navigate aircraft, control air traffic to ensure the safe and efficient operation of aircraft in flight and on the ground, and control and direct the operation of ships, boats and marine equipment.

Indicative Skill Level:

In Australia and New Zealand:

Most occupations in this minor group have a level of skill commensurate with an AQF Diploma or higher qualification. In some instances relevant experience and/or on-the-job training may be required in addition to the formal qualification (ANZSCO Skill Level 1).

Tasks Include:

- flying aircraft in accordance with established air traffic control and aircraft operating procedures
- controlling aircraft movements, and directing aircraft taxiing, take-offs and landings by radio
- monitoring aircraft performance and reporting on mechanical condition of aircraft
- giving in-flight instruction, supervising solo flights, accompanying students on training flights and demonstrating techniques for controlling aircraft
- directing fishing operations by using knowledge about the species sought, fishing areas, seasons and the capabilities of the vessel and crew
- controlling and directing shipping operations to ensure the safe and efficient loading and transport of cargo and passengers
- planning, controlling and coordinating the operational and maintenance requirements of a ship's propulsion and domestic plant and equipment
- examining and approving design plans of hulls and equipment such as main propulsion engines, auxiliary boilers and turbines, electrical power generating plant, refrigeration and airconditioning plant and pumping systems
- monitoring a ship's navigational situation, and supervising a ship's course and speed according to predetermined passage plan and safety

Occupations in this minor group are classified into the following unit groups:

2311 Air Transport Professionals

2312 Marine Transport Professionals

UNIT GROUP 2311 AIR TRANSPORT PROFESSIONALS

AIR TRANSPORT PROFESSIONALS fly and navigate aircraft, control and direct air traffic to ensure the safe and efficient operation of aircraft in flight and on the ground, and instruct students in flying aircraft.

Indicative Skill Level:

In Australia and New Zealand:

Most occupations in this unit group have a level of skill commensurate with an AQF Diploma or higher qualification. In some instances relevant experience and/or on-the job training may be required in addition to the formal qualification. Aeroplane and Helicopter Pilots require a prescribed minimum amount of flying experience in addition to the formal qualification (ANZSCO Skill Level 1).

Registration or licensing is required.

Tasks Include:

- preparing and submitting flight plans giving consideration to factors such as weather conditions and aircraft performance
- flying aircraft in accordance with established air traffic control and aircraft operating procedures
- providing flight information for flight crews and air traffic services staff
- controlling aircraft movements, and directing aircraft taxiing, take-offs and landings by radio
- providing pre-flight briefings and aeronautical information services
- completing cockpit preparations and external inspections to determine that aircraft are acceptable for flight
- monitoring aircraft performance and reporting on mechanical condition
- giving in-flight instruction, supervising solo flights, accompanying students on training flights and demonstrating techniques for controlling aircraft

Occupations:

231111 Aeroplane Pilot
231112 Air Traffic Controller
231113 Flying Instructor
231114 Helicopter Pilot
231199 Air Transport Professionals nec

231111 AEROPLANE PILOT

Flies aeroplanes to transport passengers, mail and freight, or provide agricultural, aerial surveillance or other aviation services. Registration or licensing is required.

Skill Level: 1

231112 AIR TRAFFIC CONTROLLER

Ensures the safe and efficient movement of aircraft in controlled airspace and aerodromes by directing aircraft movements. Registration or licensing is required.

Skill Level: 1

UNIT GROUP 2311 AIR TRANSPORT PROFESSIONALS *continued*

231113 FLYING INSTRUCTOR

Alternative Title:

Ground School Instructor

Teaches the theory and practical skills of flying aircraft. Registration or licensing is required.

Skill Level: 1

Specialisations:

Gliding Pilot Instructor
Helicopter Pilot Instructor

231114 HELICOPTER PILOT

Flies helicopters to transport passengers, mail or freight, or provide agricultural, aviation or aerial surveillance services. Registration or licensing is required.

Skill Level: 1

231199 AIR TRANSPORT PROFESSIONALS NEC

This occupation group covers Air Transport Professionals not elsewhere classified. Registration or licensing is required.

Skill Level: 1

Occupations in this group include:

Aircraft Navigator
Air Observer (Rescue)
Airworthiness Surveyor
Balloonist
Flight Engineer Inspector
Navigator (Air Force)

UNIT GROUP 2312 MARINE TRANSPORT PROFESSIONALS

MARINE TRANSPORT PROFESSIONALS control and manage the operations of ships, boats and marine equipment.

Indicative Skill Level:

In Australia and New Zealand:

Most occupations in this unit group have a level of skill commensurate with an AQF Diploma or higher qualification. Ship's Engineers and Ship's Surveyors require a bachelor degree or higher qualification. In some instances relevant experience and/or on-the-job training may be required in addition to the formal qualification (ANZSCO Skill Level 1).

Registration or licensing is required.

Tasks Include:

- directing fishing operations by using knowledge about the species sought, fishing areas, seasons and the capabilities of the vessel and crew
- directing crew in catching fish, molluscs and crustacea at varying depths using nets, lines, poles, pots and traps
- planning, controlling and coordinating the operational and maintenance requirements of a ship's propulsion and domestic plant and equipment
- operating plant and equipment and performing routine maintenance on ship's systems including mechanical, electrical, hydraulic, pneumatic, steam generating, and fire prevention and control systems
- controlling and directing shipping operations to ensure the safe and efficient loading and transport of cargo and passengers
- ensuring compliance with regulations pertaining to safety at sea and protection of the marine environment
- directing the activities of the deck crew for navigational support tasks, berthing and unberthing, maintenance, cleaning and painting of superstructures, and repair and replacement of defective deck gear and equipment
- navigating a ship by supervising the ship's course and speed according to predetermined passage plans and safety procedures
- examining and approving design plans of hulls and equipment such as main propulsion engines, auxiliary boilers and turbines, electrical power generating plant, refrigeration and airconditioning plant and pumping systems
- conducting periodic surveys throughout a ship's life to ensure standards are maintained

Occupations:

231211 Master Fisher
231212 Ship's Engineer
231213 Ship's Master
231214 Ship's Officer
231215 Ship's Surveyor
231299 Marine Transport Professionals nec

231211 MASTER FISHER

Controls a fishing vessel and fishing operations to catch and preserve fish, crustacea and molluscs. Registration or licensing is required.

Skill Level: 1

UNIT GROUP 2312 MARINE TRANSPORT PROFESSIONALS *continued*

231212 SHIP'S ENGINEER

Alternative Title:
Marine Engineer

Controls and manages the operation and maintenance of a ship's plant and equipment. Registration or licensing is required.

Skill Level: 1

Specialisations:
Mechanical Engineering Officer (Navy)
Weapons Electrical Engineering Officer (Navy)

231213 SHIP'S MASTER

Alternative Title:
Ship's Captain

Controls and manages the operations of a ship or boat. Registration or licensing is required.

Skill Level: 1

Specialisations:
Dredge Master
Hydrofoil Captain
Ship's Pilot
Tug Master

231214 SHIP'S OFFICER

Alternative Title:
Deck Officer

Navigates and controls the safe operation of a ship and supervises and coordinates the activities of deck crew. Registration or licensing is required.

Skill Level: 1

Specialisations:
Navigating Officer (Ship's)
Seaman Officer (Navy)

231215 SHIP'S SURVEYOR

Alternative Title:
Marine Engineer Surveyor

Surveys machines and hulls of ships to ensure they are constructed, equipped and maintained according to safety standards, rules and regulations laid down by marine authorities. Registration or licensing is required.

Skill Level: 1

UNIT GROUP 2312 MARINE TRANSPORT PROFESSIONALS *continued*

231299 MARINE TRANSPORT PROFESSIONALS NEC

This occupation group covers Marine Transport Professionals not elsewhere classified. Registration or licensing is required.

Skill Level: 1

Occupations in this group include:
- Marine Certification and Surveillance Manager
- Marine Safety Officer
- Ship's Purser

MINOR GROUP 232 ARCHITECTS, DESIGNERS, PLANNERS AND SURVEYORS

ARCHITECTS, DESIGNERS, PLANNERS AND SURVEYORS design building exteriors and interiors, and landscapes; conduct survey work to precisely position geographical features, and design, prepare and revise maps; design information for visual communication, publication and display, and products for manufacture; and develop and implement plans and policies for controlling the use of land.

Indicative Skill Level:

In Australia and New Zealand:

Most occupations in this minor group have a level of skill commensurate with a bachelor degree or higher qualification. For some occupations at least five years of relevant experience may substitute for the formal qualification. In some instances relevant experience and/or on-the-job training may be required in addition to the formal qualification. Some occupations in this minor group require high levels of creative talent or personal commitment and interest as well as, or in place of, formal qualifications or experience (ANZSCO Skill Level 1).

Tasks Include:

- obtaining advice from and providing information to clients and management about design, size, materials and costs associated with new buildings and alterations to existing buildings
- consulting with professionals and clients about external area designs, costs and construction
- designing and compiling map manuscripts using digital and graphical source material such as aerial photographs, satellite imagery, survey documents, existing maps and records, and reports and statistics
- planning and designing land subdivision projects and negotiating details with local governments and other authorities
- formulating design concepts for clothing, textiles, industrial, commercial and consumer products, jewellery and building interiors
- undertaking research and analysing functional, spatial, commercial, cultural, safety and aesthetic requirements
- preparing sketches, diagrams, illustrations and samples to communicate design concepts
- determining the objectives and constraints of design briefs by consulting with clients and stakeholders
- compiling and analysing data on economic, legal, political, cultural, demographic, sociological, physical and environmental factors affecting land use

Occupations in this minor group are classified into the following unit groups:

2321 Architects and Landscape Architects
2322 Cartographers and Surveyors
2323 Fashion, Industrial and Jewellery Designers
2324 Graphic and Web Designers, and Illustrators
2325 Interior Designers
2326 Urban and Regional Planners

UNIT GROUP 2321 ARCHITECTS AND LANDSCAPE ARCHITECTS

ARCHITECTS AND LANDSCAPE ARCHITECTS design commercial, industrial, institutional, residential and recreational buildings and landscapes.

Indicative Skill Level:

In Australia and New Zealand:

Most occupations in this unit group have a level of skill commensurate with a bachelor degree or higher qualification. In some instances relevant experience and/or on-the-job training may be required in addition to the formal qualification (ANZSCO Skill Level 1).

Registration or licensing may be required.

Tasks Include:

- obtaining advice from clients and management to determine type, style and size of planned buildings and alterations to existing buildings
- providing information regarding designs, materials and estimated building times
- preparing project documentation, including sketches and scale drawings, and integrating structural, mechanical and aesthetic elements in final designs
- writing specifications and contract documents for use by builders and calling tenders on behalf of clients
- consulting with Professionals and clients about external area designs, costs and construction
- compiling and analysing site and community data about geographical and ecological features, landforms, soils, vegetation, site hydrology, visual characteristics and human-made structures, to formulate land use and development recommendations, and for preparing environmental impact statements
- preparing reports, site plans, working drawings, specifications and cost estimates for land development, showing location and details of proposals, including ground modelling, structures, vegetation and access
- inspecting construction work in progress to ensure compliance with plans, specifications and quality standards

Occupations:

232111 Architect
232112 Landscape Architect

232111 ARCHITECT

Plans and designs buildings, provides concepts, plans, specifications and detailed drawings, negotiates with builders and advises on the procurement of buildings. Registration or licensing is required.

Skill Level: 1

Specialisation:

Conservation or Heritage Architect

232112 LANDSCAPE ARCHITECT

Plans and designs land areas for projects such as open space networks, parks, schools, institutions, roads, external areas for all building types, land subdivisions, and commercial, industrial and residential sites.

Skill Level: 1

UNIT GROUP 2322 CARTOGRAPHERS AND SURVEYORS

CARTOGRAPHERS AND SURVEYORS apply scientific and mathematical principles to design, prepare and revise maps and charts, plan, direct and conduct survey work to determine, delineate, plan and precisely position tracts of land, natural and constructed features, coastlines, marine floors and underground works, and manage related information systems.

Indicative Skill Level:

In Australia and New Zealand:

Most occupations in this unit group have a level of skill commensurate with a bachelor degree or higher qualification. In some instances relevant experience and/or on-the-job training may be required in addition to the formal qualification (ANZSCO Skill Level 1).

Registration or licensing may be required.

Tasks Include:

- designing and compiling map manuscripts using digital and graphical source material, including aerial photographs, satellite imagery, survey documents, existing maps and records, reports and statistics
- advising Surveyors and other professionals on the data requirements for map production, and on the aesthetic, technical and economic considerations of scales, details to be illustrated, place names and reproduction techniques
- supervising and coordinating the work of cartographic technicians in the production and reproduction of maps
- determining the position of points of interest on the earth's surface including marine floors, and preparing the final product data in digital form
- supervising the preparation of plans, maps, charts and drawings to give pictorial representations and managing automated spatial information systems
- undertaking research and development of surveying and photogrammetric measurement systems, cadastral systems and land information systems
- planning and designing land subdivision projects and negotiating details with local governments and other authorities
- advising Architects, Engineering Professionals, environmental and other scientists or other relevant professionals on the technical requirements of surveying, mapping and spatial information systems
- compiling and evaluating data, interpreting codes of practice, and writing reports concerning survey measurement, land use and tenure
- preparing site plans and survey reports required for conveyancing and land ownership matters

Occupations:

232211 Cartographer
232212 Surveyor

232211 CARTOGRAPHER

Alternative Title:

Map Maker

Applies scientific and mathematical principles to design, prepare and revise maps and charts. Registration or licensing may be required.

Skill Level: 1

UNIT GROUP 2322 CARTOGRAPHERS AND SURVEYORS *continued*

232212 SURVEYOR

Alternative Titles:

- Geomatician
- Geomatic Engineer

Plans, directs and conducts survey work to determine, delineate, plan and precisely position tracts of land, natural and constructed features, coastlines, marine floors and underground works, and manages related information systems. Registration or licensing may be required.

Skill Level: 1

Specialisations:

- Cadastral Surveyor
- Geodetic Surveyor
- Hydrographic Survey Operator (Navy)
- Hydrographic Surveyor
- Photogrammetric Surveyor

UNIT GROUP 2323 FASHION, INDUSTRIAL AND JEWELLERY DESIGNERS

FASHION, INDUSTRIAL AND JEWELLERY DESIGNERS plan, design, develop and document products for manufacture and prepare designs and specifications of products for mass, batch and one-off production.

Indicative Skill Level:

In Australia and New Zealand:

Most occupations in this unit group have a level of skill commensurate with a bachelor degree or higher qualification. At least five years of relevant experience may substitute for the formal qualification. In some instances relevant experience and/or on-the-job training may be required in addition to the formal qualification (ANZSCO Skill Level 1).

Tasks Include:

- determining the objectives and constraints of the design brief by consulting with clients and stakeholders
- undertaking product research and analysing functional, commercial, cultural and aesthetic requirements
- formulating design concepts for clothing, textiles, industrial, commercial and consumer products, and jewellery
- preparing sketches, diagrams, illustrations, plans, samples and models to communicate design concepts
- negotiating design solutions with clients, management, and sales and manufacturing staff
- selecting, specifying and recommending functional and aesthetic materials, production methods and finishes for manufacture
- detailing and documenting the selected design for production
- preparing and commissioning prototypes and samples
- supervising the preparation of patterns, programs and tooling, and the manufacture process

Occupations:

232311 Fashion Designer
232312 Industrial Designer
232313 Jewellery Designer

232311 FASHION DESIGNER

Plans, designs and develops clothing, accessories, footwear or other items of personal apparel considering the form and construction of clothing, historical styles and contexts, contemporary and cultural trends, colour, fabric, and decoration, and the techniques and processes available for manufacture.

Skill Level: 1

Specialisations:

Costume Designer
Leisurewear Designer

UNIT GROUP 2323 FASHION, INDUSTRIAL AND JEWELLERY DESIGNERS *continued*

232312 INDUSTRIAL DESIGNER

Alternative Title:
Product Designer

Plans, designs, develops and documents industrial, commercial or consumer products for manufacture with particular emphasis on ergonomic (human) factors, marketing considerations and manufacturability, and prepares designs and specifications of products for mass or batch production.

Skill Level: 1

Specialisations:
Ceramic Designer
Furniture Designer
Glass Designer
Textile Designer

232313 JEWELLERY DESIGNER

Conceptualises and designs prototypes and details for the manufacture of jewellery and objects for personal adornment, such as watches and spectacles, homewares and other objects, such as trophies and silverware, using metals, precious stones, plastics, engraving, casting and fabrication, to develop designs for mass or batch production or one-off commissions.

Skill Level: 1

UNIT GROUP 2324 GRAPHIC AND WEB DESIGNERS, AND ILLUSTRATORS

GRAPHIC AND WEB DESIGNERS, AND ILLUSTRATORS design information for visual and audio communication, publication and display using print, film, electronic, digital and other forms of visual and audio media.

Indicative Skill Level:

In Australia and New Zealand:

Most occupations in this unit group have a level of skill commensurate with a bachelor degree or higher qualification. At least five years of relevant experience may substitute for the formal qualification. In some instances relevant experience and/or on-the-job training may be required in addition to the formal qualification (ANZSCO Skill Level 1).

Tasks Include:

- determining the objectives and constraints of the design brief by consulting with clients and stakeholders
- undertaking research and analysing functional communication requirements
- formulating design concepts for the subject to be communicated
- preparing sketches, diagrams, illustrations and layouts to communicate design concepts
- negotiating design solutions with clients, management, sales and production staff
- selecting, specifying or recommending functional and aesthetic materials and media for publication, delivery or display
- detailing and documenting the selected design for production
- supervising or carrying out production in the chosen media
- may archive information for future client use

Occupations:

232411 Graphic Designer
232412 Illustrator
232413 Multimedia Designer
232414 Web Designer

232411 GRAPHIC DESIGNER

Alternative Title:

Graphic Artist

Plans, designs, develops and prepares information for publication and reproduction using text, symbols, pictures, colours and layout to achieve commercial and communication needs with particular emphasis on tailoring the message for the intended audience.

Skill Level: 1

Specialisations:

Exhibition Designer
Film and Video Graphics Designer
Publication Designer

UNIT GROUP 2324 GRAPHIC AND WEB DESIGNERS, AND ILLUSTRATORS *continued*

232412 ILLUSTRATOR

Plans, designs, develops, and prepares pictures and diagrams to communicate messages, clarify meaning, assist in presentations and illustrate stories, using traditional and digital media such as drawing, painting, drafting, collage, models, photography, and image capture and manipulation software.

Skill Level: 1

Specialisations:

- Animator
- Cartoonist
- Technical Illustrator

232413 MULTIMEDIA DESIGNER

Alternative Titles:

- Digital Media Designer
- Interactive Media Designer

Plans, designs and develops the production of digitally delivered information, promotional content, instructional material and entertainment through online and recorded digital media using static and animated information, text, pictures, video and sound to produce information and entertainment tailored to an intended audience and purpose.

Skill Level: 1

Specialisation:

- Instructional Designer

232414 WEB DESIGNER

Plans, designs, develops and prepares information for Internet publication with particular emphasis on the user interface, ease of navigation and location of information using text, pictures, animation, sound, colours, layout and data sources to deliver information tailored to an intended audience and purpose.

Skill Level: 1

UNIT GROUP 2325 INTERIOR DESIGNERS

INTERIOR DESIGNERS plan, design, detail and supervise the construction of commercial, industrial, retail and residential building interiors to produce an environment tailored to a purpose, with particular emphasis on space creation, space planning, and factors that enhance living and working environments.

Indicative Skill Level:

In Australia and New Zealand:

Most occupations in this unit group have a level of skill commensurate with a bachelor degree or higher qualification. At least five years of relevant experience may substitute for the formal qualification. In some instances relevant experience and/or on-the-job training may be required in addition to the formal qualification (ANZSCO Skill Level 1).

Tasks Include:

- determining the objectives and constraints of the design brief by consulting with clients and stakeholders
- researching and analysing spatial, functional, efficiency, safety and aesthetic requirements
- formulating design concepts for building interiors
- preparing sketches, diagrams, illustrations and plans to communicate design concepts
- negotiating design solutions with clients, management, suppliers and construction staff
- selecting, specifying and recommending functional and aesthetic materials, furniture and products for interiors
- detailing and documenting selected design for construction
- supervising the construction of interiors

Occupation:

232511 Interior Designer

232511 INTERIOR DESIGNER

Plans, designs, details and supervises the construction of commercial, industrial, retail and residential building interiors to produce an environment tailored to a purpose, with particular emphasis on space creation, space planning, and factors that enhance living and working environments.

Skill Level: 1

Specialisations:

Commercial Interior Designer
Environmental Designer
Residential Interior Designer
Retail Interior Designer

UNIT GROUP 2326 URBAN AND REGIONAL PLANNERS

URBAN AND REGIONAL PLANNERS develop and implement plans and policies for the controlled use of urban and rural land, and advise on economic, environmental and social factors affecting land use.

Indicative Skill Level:

In Australia and New Zealand:

Most occupations in this unit group have a level of skill commensurate with a bachelor degree or higher qualification (ANZSCO Skill Level 1).

Tasks Include:

- compiling and analysing data on economic, legal, political, cultural, demographic, sociological, physical and environmental factors affecting land use
- conferring with government authorities, communities, Architects, social scientists, Legal Professionals, and planning, development and environmental specialists
- devising and recommending use and development of land, and presenting narrative and graphic plans, programs and designs to groups and individuals
- advising governments and organisations on urban and regional planning and resource planning
- reviewing and evaluating environmental impact reports
- staying up-to-date with changes in building and zoning codes, regulations and other legal issues
- may serve as mediators in disputes over planning proposals and projects
- may speak at public meetings and appear before government to explain planning proposals

Occupation:

232611 Urban and Regional Planner

232611 URBAN AND REGIONAL PLANNER

Develops and implements plans and policies for the controlled use of urban and rural land, and advises on economic, environmental and social factors affecting land use.

Skill Level: 1

Specialisations:

Land Planner
Resource Management Planner (NZ)
Town Planner
Traffic and Transport Planner

MINOR GROUP 233 ENGINEERING PROFESSIONALS

ENGINEERING PROFESSIONALS design, plan and organise the testing, construction, installation and maintenance of structures, machines and their components, and production systems and plants, and plan production schedules and work procedures to ensure engineering projects are undertaken efficiently and in a cost effective manner.

Indicative Skill Level:

In Australia and New Zealand:

Most occupations in this minor group have a level of skill commensurate with a bachelor degree or higher qualification. In some instances relevant experience and/or on-the-job training may be required in addition to the formal qualification (ANZSCO Skill Level 1).

Tasks Include:

- planning and designing chemical process systems, civil engineering projects, electrical power equipment and facilities, electronic components used in computer and industrial applications, mechanical equipment and systems, mining and drilling operations, and other engineering projects
- drafting and interpreting specifications, drawings and plans, and determining construction methods
- supervising the construction of structures, water and gas supply and transportation systems, and the manufacture, installation, operation and maintenance of equipment, machines and plant
- organising and managing project labour and the delivery of materials, plant and equipment
- estimating total costs and preparing detailed cost plans and estimates as tools for budgetary control
- resolving design and operational problems in the various fields of engineering through the application of engineering technology

Occupations in this minor group are classified into the following unit groups:

2331 Chemical and Materials Engineers
2332 Civil Engineering Professionals
2333 Electrical Engineers
2334 Electronics Engineers
2335 Industrial, Mechanical and Production Engineers
2336 Mining Engineers
2339 Other Engineering Professionals

UNIT GROUP 2331 CHEMICAL AND MATERIALS ENGINEERS

CHEMICAL AND MATERIALS ENGINEERS design and prepare specifications for chemical process systems and the construction and operation of commercial-scale chemical plants, supervise industrial processing and fabrication of products undergoing physical and chemical change, and investigate the properties of metals, ceramics, polymers and other materials and assess and develop their engineering and commercial applications.

Indicative Skill Level:

In Australia and New Zealand:

Most occupations in this unit group have a level of skill commensurate with a bachelor degree or higher qualification. In some instances relevant experience and/or on-the-job training may be required in addition to the formal qualification (ANZSCO Skill Level 1).

Registration or licensing may be required.

Tasks Include:

- preparing designs for chemical process systems and planning control systems for processes such as those used to remove and separate components, effect chemical changes, test and evaluate fuels, transfer heat, and control the storing and handling of solids, liquids and gases
- monitoring the operation and maintenance of equipment to achieve maximum efficiency under safe operating conditions
- ensuring correct materials and equipment are used and that they conform to specifications
- diagnosing malfunctions in chemical plants and instituting remedial action
- studying product utilisation and pollution control problems
- reviewing plans for new products and submitting material selection recommendations in accordance with design specifications and factors such as strength, weight and cost
- planning and implementing laboratory operations to develop new materials and fabrication procedures for new materials to fulfil production cost and performance standards
- conferring with producers of materials, such as metals, ceramics, polymers, cements and elastomers, during the investigation and evaluation of materials suitable for specific product applications
- reviewing product failure data and implementing laboratory tests to establish or reject possible causes, and advising on ways to overcome any problems

Occupations:

233111 Chemical Engineer
233112 Materials Engineer

233111 CHEMICAL ENGINEER

Designs and prepares specifications for chemical process systems and the construction and operation of commercial-scale chemical plants, and supervises industrial processing and fabrication of products undergoing physical and chemical changes. Registration or licensing may be required.

Skill Level: 1

233112 MATERIALS ENGINEER

Investigates the properties of metals, ceramics, polymers and other materials and assesses and develops their engineering and commercial applications. Registration or licensing may be required.

Skill Level: 1

UNIT GROUP 2332 CIVIL ENGINEERING PROFESSIONALS

CIVIL ENGINEERING PROFESSIONALS design, plan, organise and oversee the construction of civil engineering projects such as dams, bridges, pipelines, gas and water supply schemes, sewerage systems, roads, airports and other structures; analyse the likely behaviour of soil and rock when placed under pressure by proposed structures and design structural foundations; analyse the statical properties of all types of structures and test the behaviour and durability of materials used in their construction; plan and develop transportation systems; and estimate and monitor the construction costs of projects.

Indicative Skill Level:

In Australia and New Zealand:

Most occupations in this unit group have a level of skill commensurate with a bachelor degree or higher qualification. In some instances relevant experience and/or on-the-job training may be required in addition to the formal qualification (ANZSCO Skill Level 1).

Registration or licensing may be required.

Tasks Include:

- determining construction methods, materials and quality standards, and drafting and interpreting specifications, drawings, plans, construction methods and procedures
- organising and directing site labour and the delivery of construction materials, plant and equipment, and establishing detailed programs for the coordination of site activities
- obtaining soil and rock samples at different depths across sites and testing samples to determine strength, compressibility and other factors that affect the behaviour of soil and rock when a structure is imposed and determining the safe loading for the soil
- studying architectural and engineering drawings and specifications to estimate total costs, and preparing detailed cost plans and estimates as tools to assist in budgetary control
- monitoring changes to designs, assessing effects on cost, and measuring, valuing and negotiating variations to designs
- analysing structural systems for both static and dynamic loads
- designing structures to ensure they do not collapse, bend, twist or vibrate in undesirable ways
- assessing present and future travel flow patterns taking into account population increase and needs change
- designing the physical aspects of transportation systems such as highways, railroads, urban transit, air transportation, logistical supply systems and their terminals

Occupations:

233211 Civil Engineer
233212 Geotechnical Engineer
233213 Quantity Surveyor
233214 Structural Engineer
233215 Transport Engineer

UNIT GROUP 2332 CIVIL ENGINEERING PROFESSIONALS *continued*

233211 CIVIL ENGINEER

Plans, designs, organises and oversees the construction and operation of dams, bridges, pipelines, gas and water supply schemes, sewerage systems, airports and other civil engineering projects. Registration or licensing may be required.

Skill Level: 1

Specialisations:
- Airfield Engineer Officer (Air Force)
- Hydraulics Engineer

233212 GEOTECHNICAL ENGINEER

Plans, directs and conducts survey work to analyse the likely behaviour of soil and rock when placed under pressure by proposed structures, and designs above and below ground foundations. Registration or licensing may be required.

Skill Level: 1

233213 QUANTITY SURVEYOR

Alternative Titles:
- Building Economist
- Construction Economist

Estimates and monitors construction costs from the project feasibility stage, through tender preparation, to the construction period and beyond. Registration or licensing is required.

Skill Level: 1

233214 STRUCTURAL ENGINEER

Analyses the statical properties of all types of structures, tests the behaviour and durability of materials used in their construction, and designs and supervises the construction of all types of structures. Registration or licensing may be required.

Skill Level: 1

233215 TRANSPORT ENGINEER

Plans and develops transport systems to improve infrastructure efficiency and the cost effectiveness of moving people and freight. Registration or licensing may be required.

Skill Level: 1

Specialisation:
- Roading Engineer (NZ)

UNIT GROUP 2333 ELECTRICAL ENGINEERS

ELECTRICAL ENGINEERS design, develop and supervise the manufacture, installation, operation and maintenance of equipment, machines and systems for the generation, distribution, utilisation and control of electric power.

Indicative Skill Level:

In Australia and New Zealand:

Most occupations in this unit group have a level of skill commensurate with a bachelor degree or higher qualification. In some instances relevant experience and/or on-the-job training may be required in addition to the formal qualification (ANZSCO Skill Level 1).

Registration or licensing may be required.

Tasks Include:

- planning and designing power stations and power generation equipment
- determining the type and arrangement of circuits, transformers, circuit-breakers, transmission lines and other equipment
- developing products such as electric motors, components, equipment and appliances
- interpreting specifications, drawings, standards and regulations relating to electric power equipment and use
- organising and managing resources used in the supply of electrical components, machines, appliances and equipment
- establishing delivery and installation schedules for machines, switchgear, cables and fittings
- supervising the operation and maintenance of power stations, transmission and distribution systems and industrial plants
- designing and installing control and signalling equipment for road, rail and air traffic
- may specialise in research in areas such as power generation and transmission systems, transformers, switchgear and electric motors, telemetry and control systems

Occupation:

233311 Electrical Engineer

233311 ELECTRICAL ENGINEER

Designs, develops and supervises the manufacture, installation, operation and maintenance of equipment, machines and systems for the generation, distribution, utilisation and control of electric power. Registration or licensing may be required.

Skill Level: 1

Specialisations:

Electrical Design Engineer
Railway Signalling Engineer
Signalling and Communications Engineer

UNIT GROUP 2334 ELECTRONICS ENGINEERS

ELECTRONICS ENGINEERS design, develop, adapt, install, test and maintain electronic components, circuits and systems used for computer systems, communication systems, entertainment, transport and other industrial applications.

Indicative Skill Level:

In Australia and New Zealand:

Most occupations in this unit group have a level of skill commensurate with a bachelor degree or higher qualification. In some instances relevant experience and/or on-the-job training may be required in addition to the formal qualification (ANZSCO Skill Level 1).

Registration or licensing may be required.

Tasks Include:

- designing electronic components, circuits and systems used for computer, communication and control systems, and other industrial applications
- designing software, especially embedded software, to be used within such systems
- developing apparatus and procedures to test electronic components, circuits and systems
- supervising installation and commissioning of computer, communication and control systems, and ensuring proper control and protection methods
- establishing and monitoring performance and safety standards and procedures for operation, modification, maintenance and repair of such systems
- designing communications bearers based on wired, optical fibre and wireless communication media
- analysing communications traffic and level of service, and determining the type of installation, location, layout and transmission medium for communication systems
- designing and developing signal processing algorithms and implementing these through appropriate choice of hardware and software

Occupation:

233411 Electronics Engineer

233411 ELECTRONICS ENGINEER

Designs, develops, adapts, installs, tests and maintains electronic components, circuits and systems used for computer systems, communication systems, entertainment, transport and other industrial applications. Registration or licensing may be required.

Skill Level: 1

Specialisation:

Communications Engineer (Army)

UNIT GROUP 2335 INDUSTRIAL, MECHANICAL AND PRODUCTION ENGINEERS

INDUSTRIAL, MECHANICAL AND PRODUCTION ENGINEERS design, organise and oversee the construction, operation and maintenance of mechanical and process plant and installations, establish programs for the coordination of manufacturing activities, and ensure usage of resources is cost effective.

Indicative Skill Level:

In Australia and New Zealand:

Most occupations in this unit group have a level of skill commensurate with a bachelor degree or higher qualification. In some instances relevant experience and/or on-the-job training may be required in addition to the formal qualification (ANZSCO Skill Level 1).

Registration or licensing may be required.

Tasks Include:

- studying functional statements, organisational charts and project information to determine functions and responsibilities of workers and work units and to identify areas of duplication
- establishing work measurement programs and analysing work samples to develop standards for labour utilisation
- analysing workforce utilisation, facility layout, operational data and production schedules and costs to determine optimum worker and equipment efficiencies
- designing mechanical equipment, machines, components, products for manufacture, and plant and systems for construction
- developing specifications for manufacture, and determining materials, equipment, piping, material flows, capacities and layout of plant and systems
- organising and managing project labour and the delivery of materials, plant and equipment
- establishing standards and policies for installation, modification, quality control, testing, inspection and maintenance according to engineering principles and safety regulations
- inspecting plant to ensure optimum performance is maintained
- directing the maintenance of plant buildings and equipment, and coordinating the requirements for new designs, surveys and maintenance schedules

Occupations:

233511 Industrial Engineer
233512 Mechanical Engineer
233513 Production or Plant Engineer

233511 INDUSTRIAL ENGINEER

Investigates and reviews the utilisation of personnel, facilities, equipment and materials, current operational processes and established practices, to recommend improvement in the efficiency of operations in a variety of commercial, industrial and production environments. Registration or licensing may be required.

Skill Level: 1

Specialisation:

Process Engineer (Industrial)

UNIT GROUP 2335 INDUSTRIAL, MECHANICAL AND PRODUCTION ENGINEERS *continued*

233512 MECHANICAL ENGINEER

Plans, designs, organises and oversees the assembly, erection, operation and maintenance of mechanical and process plant and installations. Registration or licensing may be required.

Skill Level: 1

Specialisations:
- Airconditioning Engineer
- Heating and Ventilation Engineer

233513 PRODUCTION OR PLANT ENGINEER

Plans, directs and coordinates the design, construction, modification, continued performance and maintenance of equipment and machines in industrial plants, and the management and planning of manufacturing activities. Registration or licensing may be required.

Skill Level: 1

Specialisation:
- Automation and Control Engineer

UNIT GROUP 2336 MINING ENGINEERS

MINING ENGINEERS plan and direct the engineering aspects of locating and extracting minerals, petroleum and natural gas from the earth.

Indicative Skill Level:
In Australia and New Zealand:

Most occupations in this unit group have a level of skill commensurate with a bachelor degree or higher qualification. In some instances relevant experience and/or on-the-job training may be required in addition to the formal qualification (ANZSCO Skill Level 1).

Registration or licensing may be required.

Tasks Include:

- conducting preliminary surveys of mineral, petroleum and natural gas deposits with prospectors, Geologists, Geophysicists, other mineral scientists and other engineers to determine the resources present, the feasibility of extracting the reserves, and the design and development of the extraction process
- preparing operation and project cost estimates and production schedules, and reporting progress, production and costs compared to budget
- determining the most suitable methods of ore extraction taking account of such factors as depth of overburden, and attitude and physical characteristics of deposits and surrounding strata
- preparing plans for tunnels and chambers, location and construction of mine shafts, layout of mine development and the application of appropriate mining techniques, often using computer modelling
- assessing the natural, technical, financial and safety risks associated with the phases of the project development, construction and operations
- determining the safety of processes, order of extraction and safety of mine walls, evaluating the risk of slippage and advising on the prevention of slippage and rock falls
- planning and coordinating the utilisation of labour and equipment consistent with efficiency targets, statutes, safety guidelines and environmental conditions
- planning and conducting research and providing advice on engineering operations for the exploration, location and extraction of petroleum and natural gas
- determining location for drilling
- deciding on types of derrick and equipment including seabed platforms
- devising methods of controlling the flow of oil and gas from wells

Occupations:

233611 Mining Engineer (excluding Petroleum)
233612 Petroleum Engineer

233611 MINING ENGINEER (EXCLUDING PETROLEUM)

Plans and directs the engineering aspects of locating and extracting minerals from the earth. Registration or licensing may be required.

Skill Level: 1

Specialisation:

Process Engineer (Mining)

UNIT GROUP 2336 MINING ENGINEERS *continued*

233612 PETROLEUM ENGINEER

Plans and directs the engineering aspects of locating and extracting petroleum or natural gas from the earth. Registration or licensing may be required.

Skill Level: 1

Specialisations:
- Mud Engineer
- Petrophysical Engineer

UNIT GROUP 2339 OTHER ENGINEERING PROFESSIONALS

This unit group covers Engineering Professionals not elsewhere classified.

It includes Aeronautical Engineers, Agricultural Engineers, Biomedical Engineers, Engineering Technologists, Environmental Engineers and Naval Architects (Aus) / Marine Designers (NZ).

Indicative Skill Level:
In Australia and New Zealand:

Most occupations in this unit group have a level of skill commensurate with a bachelor degree or higher qualification. In some instances relevant experience and/or on-the-job training may be required in addition to the formal qualification (ANZSCO Skill Level 1).

Registration or licensing may be required.

Occupations:

233911 Aeronautical Engineer
233912 Agricultural Engineer
233913 Biomedical Engineer
233914 Engineering Technologist
233915 Environmental Engineer
233916 Naval Architect (Aus) / Marine Designer (NZ)
233999 Engineering Professionals nec

233911 AERONAUTICAL ENGINEER

Performs and supervises engineering work concerned with the design, development, manufacture, maintenance and modification of aircraft for flight. Registration or licensing may be required.

Skill Level: 1

Specialisations:

Aeronautical Engineering Officer (Navy)
Aerospace Engineer
Aerospace Engineer Officer - Aeronautical (Air Force)
Aerospace Engineer Officer - Armament (Air Force)
Aerospace Engineer Officer - Electronics (Air Force)
Avionics Systems Engineer
Weapons Aeronautical Engineering Officer (Navy)

233912 AGRICULTURAL ENGINEER

Alternative Title:

Natural Resources Engineer

Performs and supervises engineering work related to the use and development of agricultural land, buildings, machines and equipment. Registration or licensing may be required.

Skill Level: 1

UNIT GROUP 2339 OTHER ENGINEERING PROFESSIONALS *continued*

233913 BIOMEDICAL ENGINEER

Applies knowledge and methodology of physics, engineering, mathematics, computing, physical chemistry and materials science to problems in biology and the treatment and prevention of human disease. Registration or licensing may be required.

Skill Level: 1

Specialisations:
- Bioengineer
- Clinical Engineer
- Medical Engineer

233914 ENGINEERING TECHNOLOGIST

Analyses and modifies new and existing engineering technologies and applies them in the testing and implementation of engineering projects. Registration or licensing may be required.

Skill Level: 1

Specialisations:
- Aeronautical Engineering Technologist
- Agricultural Engineering Technologist
- Biomedical Engineering Technologist
- Chemical Engineering Technologist
- Industrial Engineering Technologist
- Mining Engineering Technologist

233915 ENVIRONMENTAL ENGINEER

Assesses the impact on air, water, soil and noise levels in the vicinity of engineering projects, plans and designs equipment and processes for the treatment and safe disposal of waste material, and assesses what may cause problems for the environment in the long-term. Registration or licensing is required.

Skill Level: 1

233916 NAVAL ARCHITECT (AUS) / MARINE DESIGNER (NZ)

Designs and oversees the construction and repair of marine craft and floating structures. Registration or licensing may be required.

Skill Level: 1

233999 ENGINEERING PROFESSIONALS NEC

This occupation group covers Engineering Professionals not elsewhere classified. Registration or licensing may be required.

Skill Level: 1

Occupations in this group include:
- Mechatronics Engineer
- Product Design Engineer

MINOR GROUP 234 NATURAL AND PHYSICAL SCIENCE PROFESSIONALS

NATURAL AND PHYSICAL SCIENCE PROFESSIONALS perform analytical, conceptual and practical tasks in relation to environmental factors and agricultural production, the chemical and physical properties of the universe, the extraction and processing of mineral ores, life forms including the physiology and biochemistry of humans, plants and animals, and disease prevention.

Indicative Skill Level:
In Australia and New Zealand:

Most occupations in this minor group have a level of skill commensurate with a bachelor degree or higher qualification. In some instances relevant experience and/or on-the-job training may be required in addition to the formal qualification (ANZSCO Skill Level 1).

Tasks Include:

- advising farmers on techniques for improving crop and livestock production
- conducting experiments and tests to identify the chemical composition and reactive properties of natural substances and processed materials
- developing conservation and management policies for biological resources, such as fish populations and forests, and establishing standards and developing approaches for the control of pollution and the rehabilitation of areas disturbed by activities such as mining, timber felling and overgrazing
- conducting studies of minerals and the nature and formation of the earth's crust, and carrying out mineral exploration
- studying the forms and structures of organs and tissues of the body by systematic observation, dissection and microscopic examination
- investigating the effects of environmental factors, such as rainfall, temperature, sunlight, soil, topography and disease, on plant and animal growth
- analysing samples of body tissue and fluids to develop techniques to aid in the diagnosis and treatment of diseases
- treating animals medically and surgically, and administering and prescribing drugs, analgesics, and general and local anaesthetics for animals
- conducting experiments to discover and develop industrial, medical and other practical applications of physics
- studying and applying chemical and metallurgical techniques for extracting and refining metallic materials from their ores and concentrates

Occupations in this minor group are classified into the following unit groups:

2341 Agricultural and Forestry Scientists
2342 Chemists, and Food and Wine Scientists
2343 Environmental Scientists
2344 Geologists and Geophysicists
2345 Life Scientists
2346 Medical Laboratory Scientists
2347 Veterinarians
2349 Other Natural and Physical Science Professionals

UNIT GROUP 2341 AGRICULTURAL AND FORESTRY SCIENTISTS

AGRICULTURAL AND FORESTRY SCIENTISTS advise farmers, rural industries and government on aspects of farming, develop techniques for increasing productivity, and study and develop plans and policies for the management of forest areas.

Indicative Skill Level:

In Australia and New Zealand:

Most occupations in this unit group have a level of skill commensurate with a bachelor degree or higher qualification. In some instances relevant experience and/or on-the-job training may be required in addition to the formal qualification (ANZSCO Skill Level 1).

Tasks Include:

- collecting and analysing data and samples of produce, feed, soil and other factors affecting production
- advising Farmers and Farm Managers on techniques for improving the production of crops and livestock, and alternative agricultural options
- advising farmers on issues such as livestock and crop disease, control of pests and weeds, soil improvement, animal husbandry and feeding programs
- studying the environmental factors affecting commercial crop production, pasture growth, animal breeding, and the growth and health of forest trees
- studying the effects of cultivation techniques, soils, insects and plant diseases on animal, crop and forest production
- developing procedures and techniques for solving agricultural problems and improving the efficiency of production
- managing forest resources to maximise their long-term commercial, recreational and environmental benefits for the community
- studying the propagation and culture of forest trees, methods for improving the growth of stock, and the effects of thinning on forest yields
- preparing plans for reafforestation and devising efficient harvesting systems
- investigating, planning and implementing management procedures to cope with the effects of fires, floods, droughts, soil erosion, insect pests and diseases

Occupations:

234111 Agricultural Consultant
234112 Agricultural Scientist
234113 Forester (Aus) / Forest Scientist (NZ)

234111 AGRICULTURAL CONSULTANT

Alternative Title:

Agricultural Adviser

Advises farmers, agricultural businesses, rural industries and government on the production, processing and distribution of farm products.

Skill Level: 1

Specialisations:

Agricultural Extension Officer
Landcare Officer

UNIT GROUP 2341 AGRICULTURAL AND FORESTRY SCIENTISTS *continued*

234112 AGRICULTURAL SCIENTIST

Studies commercial plants, animals and cultivation techniques to enhance the productivity of farms and agricultural industries.

Skill Level: 1

Specialisation:
Agronomist

234113 FORESTER (AUS) / FOREST SCIENTIST (NZ)

Studies, develops and manages forest areas to maintain commercial and recreational uses, conserve flora and fauna, and protect against fire, pests and diseases.

Skill Level: 1

Specialisations:
Forestry Adviser
Forestry Consultant
Silviculturist

UNIT GROUP 2342 CHEMISTS, AND FOOD AND WINE SCIENTISTS

CHEMISTS, AND FOOD AND WINE SCIENTISTS study the chemical and physical properties of substances, develop and monitor chemical processes and production, develop new and improve existing food products, and plan and coordinate the production of wine and spirits.

Indicative Skill Level:

In Australia and New Zealand:

Most occupations in this unit group have a level of skill commensurate with a bachelor degree or higher qualification. In some instances relevant experience and/or on-the-job training may be required in addition to the formal qualification. In the case of Wine Makers, at least five years of experience may substitute for the formal qualification (ANZSCO Skill Level 1).

Tasks Include:

- conducting experiments and tests to identify the chemical composition and reactive properties of natural substances and processed materials
- analysing and conducting research to develop theories, techniques and processes, and testing the reliability of outcomes under different conditions
- developing practical applications of experimental and research findings
- testing food products for flavour, colour, taste, texture and nutritional content
- advising on preserving, processing, packaging, storing and delivering foods
- developing quality control procedures and safety standards for the manufacture of food products
- examining grape samples to assess ripeness, sugar and acid content, and determining suitability for processing
- coordinating winemaking processes, directing workers in testing and crushing grapes, fermenting juices, and fortifying, clarifying, maturing and finishing wines
- blending wines according to formulae and knowledge of winemaking techniques

Occupations:

234211 Chemist
234212 Food Technologist
234213 Wine Maker

234211 CHEMIST

Studies the chemical and physical properties of substances, and develops and monitors chemical processes and production.

Skill Level: 1

Specialisations:

Analytical Chemist
Industrial Chemist

234212 FOOD TECHNOLOGIST

Alternative Title:

Food Scientist

Develops new and improves existing food products, and sets standards for producing, packaging and marketing food.

Skill Level: 1

UNIT GROUP 2342 CHEMISTS, AND FOOD AND WINE SCIENTISTS *continued*

234213 WINE MAKER

Alternative Title:

Oenologist

Plans, supervises and coordinates the production of wine or spirits from selected varieties of grapes.

Skill Level: 1

UNIT GROUP 2343 ENVIRONMENTAL SCIENTISTS

ENVIRONMENTAL SCIENTISTS study, develop, implement and advise on policies and plans for managing and protecting the environment, flora, fauna and other natural resources.

Indicative Skill Level:

In Australia and New Zealand:

Most occupations in this unit group have a level of skill commensurate with a bachelor degree or higher qualification. In some instances relevant experience and/or on-the-job training may be required in addition to the formal qualification (ANZSCO Skill Level 1).

Tasks Include:

- evaluating habitat, wildlife and fisheries needs, and formulating short- and long-term management goals and objectives
- enforcing laws and regulations to conserve and protect fish and wildlife
- carrying out environmental impact assessments for a wide range of development projects
- proposing solutions to address negative environmental impact
- studying the effects of factors, such as terrain, altitude, climatic and environmental change, sources of nutrition, predators and the impacts of humans, on animal and plant life
- studying and analysing pollution, atmospheric conditions, demographic characteristics, ecology, mineral, soil and water samples
- developing conservation and management policies for biological resources, such as fish populations and forests, and establishing standards and developing approaches for the control of pollution and the rehabilitation of areas disturbed by activities such as mining, timber felling and overgrazing
- implementing policies and organising activities in designated parks and other areas to conserve and protect natural and cultural heritage
- participating in management planning by providing environmental information and making inventories of plants, animals and items of cultural and heritage significance

Occupations:

234311 Conservation Officer
234312 Environmental Consultant
234313 Environmental Research Scientist
234314 Park Ranger
234399 Environmental Scientists nec

234311 CONSERVATION OFFICER

Alternative Title:

Environmental Officer

Develops and implements programs and regulations for the protection of fish, wildlife and other natural resources.

Skill Level: 1

Specialisation:

Landcare Facilitator

UNIT GROUP 2343 ENVIRONMENTAL SCIENTISTS *continued*

234312 ENVIRONMENTAL CONSULTANT

Alternative Titles:

- Environmental Adviser
- Environmental Analyst

Analyses and advises on policies guiding the design, implementation and modification of government or commercial environmental operations and programs.

Skill Level: 1

234313 ENVIRONMENTAL RESEARCH SCIENTIST

Alternative Title:

- Environmental Scientist

Studies and develops policies and plans for the control of factors which may produce pollution, imbalance in or degradation of the environment.

Skill Level: 1

Specialisations:

- Air Pollution Analyst
- Ecologist
- Land Degradation Analyst
- Water Quality Analyst

234314 PARK RANGER

Assists in controlling a State or national park, scenic area, historic site, nature reserve, recreation area or conservation reserve in accordance with authorised policies and priorities.

Skill Level: 1

234399 ENVIRONMENTAL SCIENTISTS NEC

This occupation group covers Environmental Scientists not elsewhere classified.

Skill Level: 1

Occupations in this group include:

- Aquaculture Consultant
- Environmental Auditor
- Soil Scientist

UNIT GROUP 2344 GEOLOGISTS AND GEOPHYSICISTS

GEOLOGISTS AND GEOPHYSICISTS study the composition, structure and other physical attributes of the earth, locate and advise on the extraction of minerals, petroleum and ground water, and detect, monitor and forecast seismic, magnetic, electrical, thermal and oceanographic activity.

Geographers are excluded from this unit group. Geographers are included in Unit Group 2724 Social Professionals.

Indicative Skill Level:

In Australia and New Zealand:

Most occupations in this unit group have a level of skill commensurate with a bachelor degree or higher qualification. In some instances relevant experience and/or on-the-job training may be required in addition to the formal qualification (ANZSCO Skill Level 1).

Tasks Include:

- conducting preliminary surveys of mineral, petroleum and natural gas deposits with prospectors, Mining Engineers, Metallurgists, and other mineral scientists and engineers
- preparing and supervising the production of laboratory reports and scientific papers
- conducting studies of the structure, nature and formation of the earth's crust and the minerals contained in it
- studying and dating fossils and rock strata to develop knowledge of the evolution and biology of life forms, and to assess their commercial applications
- studying the effects of natural events, such as erosion, sedimentation, earthquakes and volcanic activity, on the formation of the earth's surface and sea beds
- carrying out exploration to determine the resources present by sampling, examining and analysing geological specimens, rock cores, cuttings and samples using optical, chemical, electronic and mechanical techniques
- conducting surveys of variations in the earth's gravitational and magnetic fields to determine its physical features
- investigating the propagation of seismic waves to determine the structure and stability of the earth's mantle and crust
- studying the causes of earthquakes and other stress states of the earth's crust
- performing laboratory and field studies, and aerial, ground and drill hole surveys

Occupations:

234411 Geologist
234412 Geophysicist

234411 GEOLOGIST

Studies the composition, structure and other physical attributes of the earth to increase scientific knowledge and to develop practical applications in fields such as mineral exploitation, civil engineering, environmental protection and rehabilitation of land after mining.

Skill Level: 1

Specialisations:

Hydrogeologist
Marine Geologist
Palaeontologist

UNIT GROUP 2344 GEOLOGISTS AND GEOPHYSICISTS *continued*

234412 GEOPHYSICIST

Studies the composition, structure and other physical attributes of the earth, locates minerals, petroleum or ground water, and detects, monitors and forecasts seismic, magnetic, electrical, geothermal and oceanographic activity.

Skill Level: 1

Specialisations:
- Physical Oceanographer
- Seismologist

UNIT GROUP 2345 LIFE SCIENTISTS

LIFE SCIENTISTS examine the anatomy, physiology and biochemistry of humans, animals, plants and other living organisms to better understand how living organisms function and interact with each other and the environment in which they live.

Indicative Skill Level:

In Australia and New Zealand:

Most occupations in this unit group have a level of skill commensurate with a bachelor degree or higher qualification. In some instances relevant experience and/or on-the-job training may be required in addition to the formal qualification (ANZSCO Skill Level 1).

Tasks Include:

- designing and conducting experiments, making observations and measurements, researching information, analysing data, preparing or supervising the preparation of laboratory reports and scientific papers, presenting findings at scientific meetings and conferences, and supervising the work of staff
- studying the forms and structures of bodily organs and tissues by systematic observation, dissection and microscopic examination
- investigating the chemical structure and function of living cells and their isolated components, organs and tissues in humans, animals, plants, and micro-organisms
- examining micro-organisms, such as bacteria, fungi, yeast and their enzymes, and using the knowledge gained to create and develop new, and improve existing, products, materials and processes
- investigating the effects of environmental factors, such as rainfall, temperature, sunlight, soil, topography and disease, on plant growth
- planning and undertaking experiments to study, measure and understand marine animals and plants
- studying the growth and characteristics of micro-organisms, such as bacteria, algae and fungi, and the effects they have on plants, animals and humans to develop medical, veterinary, industrial, environmental and other practical applications
- investigating the interrelationships between animals in their natural surroundings, in captivity and in laboratories

Occupations:

234511 Life Scientist (General)
234512 Anatomist or Physiologist
234513 Biochemist
234514 Biotechnologist
234515 Botanist
234516 Marine Biologist
234517 Microbiologist
234518 Zoologist
234599 Life Scientists nec

234511 LIFE SCIENTIST (GENERAL)

Alternative Title:

Biologist (General)

Studies the origin, anatomy, physiology, reproduction and behaviour of living organisms and the ways in which they interact with the environment in which they live.

Skill Level: 1

UNIT GROUP 2345 LIFE SCIENTISTS *continued*

234512 ANATOMIST OR PHYSIOLOGIST

Studies the anatomy and physiology of humans.

Skill Level: 1

Specialisations:
- Embryologist
- Neuroanatomist

234513 BIOCHEMIST

Studies the biochemistry of living organisms and the molecular structure and function of related components.

Skill Level: 1

Specialisations:
- Enzyme Chemist
- Protein Chemist

234514 BIOTECHNOLOGIST

Studies the anatomy, physiology and characteristics of living organisms and isolated biological molecules, and develops new materials for applying to a range of purposes.

Skill Level: 1

Specialisations:
- Cell Geneticist
- Molecular Biologist
- Molecular Geneticist

234515 BOTANIST

Studies the anatomy, physiology, biochemistry and ecology of plants.

Skill Level: 1

Specialisations:
- Plant Morphologist
- Plant Pathologist
- Plant Physiologist
- Plant Taxonomist

234516 MARINE BIOLOGIST

Studies the anatomy, physiology, functions, characteristics, behaviour and environments of all forms of life living in the sea and connected water bodies.

Skill Level: 1

234517 MICROBIOLOGIST

Studies microscopic forms of life such as bacteria, viruses and protozoa.

Skill Level: 1

Specialisation:
- Bacteriologist (Non-medical)

UNIT GROUP 2345 LIFE SCIENTISTS *continued*

234518 ZOOLOGIST

Studies the anatomy, physiology, characteristics, ecology, behaviour and environments of animals.

Skill Level: 1

Specialisations:
- Entomologist
- Mammologist
- Ornithologist

234599 LIFE SCIENTISTS NEC

This occupation group covers Life Scientists not elsewhere classified.

Skill Level: 1

Occupations in this group include:
- Animal Behaviourist
- Parasitologist
- Pharmacologist (Non-clinical)
- Toxicologist

UNIT GROUP 2346 MEDICAL LABORATORY SCIENTISTS

MEDICAL LABORATORY SCIENTISTS conduct medical laboratory tests to assist in the diagnosis, treatment and prevention of disease.

Indicative Skill Level:

In Australia and New Zealand:

Most occupations in this unit group have a level of skill commensurate with a bachelor degree or higher qualification. In some instances relevant experience and/or on-the-job training may be required in addition to the formal qualification (ANZSCO Skill Level 1).

Tasks Include:

- preparing tissue sections for microscopic examination
- examining and analysing samples to study the effects of microbial infections
- analysing samples of body tissue and fluids to develop techniques to aid in the diagnosis and treatment of diseases
- advising Medical Practitioners on the interpretation of tests and methods for use in the diagnosis and treatment of disease
- setting up the steps and rules of laboratory medical testing
- operating and maintaining laboratory equipment
- maintaining laboratory quality assurance and safety standards
- preparing scientific papers and reports

Occupation:

234611 Medical Laboratory Scientist

234611 MEDICAL LABORATORY SCIENTIST

Alternative Titles:

Hospital Scientist
Medical Scientific Officer

Conducts medical laboratory tests to assist in the diagnosis, treatment and prevention of disease.

Skill Level: 1

UNIT GROUP 2347 VETERINARIANS

VETERINARIANS diagnose, treat and prevent animal diseases, ailments and injuries.

Indicative Skill Level:

In Australia and New Zealand:

Most occupations in this unit group have a level of skill commensurate with a bachelor degree or higher qualification (ANZSCO Skill Level 1).

Registration or licensing is required.

Tasks Include:

- treating animals medically and surgically, and administering and prescribing drugs, analgesics, and general and local anaesthetics
- determining the presence and nature of abnormal conditions by physical examination, laboratory testing and through diagnostic imaging techniques including radiography and ultrasound
- performing surgery, dressing wounds and setting broken bones
- rendering obstetric services to animals
- participating in programs designed to prevent the occurrence and spread of animal diseases
- inoculating animals against, and testing for, infectious diseases and notifying authorities of outbreaks of infectious animal diseases
- performing autopsies to determine cause of death
- advising clients on health, nutrition and feeding, hygiene, breeding and care of animals
- may provide professional services to commercial firms producing biological and pharmaceutical products
- may specialise in the treatment of a particular animal group or in a particular specialty area such as cardiology, chiropractic, dermatology or critical care

Occupation:

234711 Veterinarian

234711 VETERINARIAN

Alternative Title:

Veterinary Surgeon

Diagnoses, treats and prevents animal diseases, ailments and injuries. Registration or licensing is required.

Skill Level: 1

Specialisations:

Veterinary Parasitologist

Veterinary Pathologist

UNIT GROUP 2349 OTHER NATURAL AND PHYSICAL SCIENCE PROFESSIONALS

This unit group covers Natural and Physical Science Professionals not elsewhere classified.

It includes Conservators, Metallurgists, Meteorologists and Physicists.

Indicative Skill Level:
In Australia and New Zealand:

Most occupations in this unit group have a level of skill commensurate with a bachelor degree or higher qualification. In some instances relevant experience and/or on-the-job training may be required in addition to the formal qualification. In the case of Conservators, at least five years of relevant experience may substitute for the formal qualification (ANZSCO Skill Level 1).

Registration or licensing may be required.

Occupations:
- 234911 Conservator
- 234912 Metallurgist
- 234913 Meteorologist
- 234914 Physicist
- 234999 Natural and Physical Science Professionals nec

234911 CONSERVATOR

Plans and organises the conservation of materials and objects in libraries, archives, museums, art galleries and other institutions.

Skill Level: 1

Specialisation:
- Art Conservator

234912 METALLURGIST

Researches, develops, controls and provides advice on processes used in extracting metals from their ores, and processes used for casting, alloying, heat treating or welding refined metals, alloys and other materials to produce commercial metal products or develop new alloys and processes. Registration or licensing may be required.

Skill Level: 1

Specialisations:
- Hydrometallurgical Engineer
- Metallographer
- Pyrometallurgical Engineer
- Radiological Metallurgist

UNIT GROUP 2349 OTHER NATURAL AND PHYSICAL SCIENCE PROFESSIONALS *continued*

234913 METEOROLOGIST

Studies the physics and dynamics of the atmosphere to increase understanding of weather and climate, and to forecast changes in the weather and long-term climatic trends.

Skill Level: 1

Specialisations:

- Climatologist
- Weather Forecaster

234914 PHYSICIST

Studies matter, space, time, energy, forces and fields and the interrelationship between these physical phenomena to further understanding of the laws governing the behaviour of the universe, and seeks to apply these laws to solve practical problems and discover new information about the earth and the universe.

Skill Level: 1

Specialisations:

- Astronomer
- Medical Physicist

234999 NATURAL AND PHYSICAL SCIENCE PROFESSIONALS NEC

This occupation group covers Natural and Physical Science Professionals not elsewhere classified.

Skill Level: 1

Occupations in this group include:

- Ceramics Scientist
- Exercise Physiologist
- Mineral Processing Engineer
- Polymer Scientist
- Sports Scientist

SUB-MAJOR GROUP 24 EDUCATION PROFESSIONALS

EDUCATION PROFESSIONALS educate students in early childhood (pre-primary), primary, middle or intermediate, secondary, tertiary, private and special education institutions by teaching one or more subjects; research and develop curricula and teaching materials; and promote students' social, emotional, intellectual and physical development.

Indicative Skill Level:

In Australia and New Zealand:

Most occupations in this sub-major group have a level of skill commensurate with a bachelor degree or higher qualification. At least five years of relevant experience may substitute for the formal qualification. In some instances relevant experience and/or on-the-job training may be required in addition to the formal qualification (ANZSCO Skill Level 1).

Tasks Include:

- delivering prescribed courses of study
- monitoring and assessing the progress of individual students
- attending to the social, emotional, intellectual and physical wellbeing of students and maintaining acceptable standards of conduct
- discussing individual progress, courses, academic matters and problems with other professionals, parents and students
- providing tuition in specialised subjects such as art, music, dance and drama
- preparing and presenting theoretical and practical subject matter in lectures, tutorials and workshop sessions
- conducting research and advising on particular areas of the education process, curriculum coordination and design

Occupations in this sub-major group are classified into the following minor groups:

241 School Teachers
242 Tertiary Education Teachers
249 Miscellaneous Education Professionals

MINOR GROUP 241 SCHOOL TEACHERS

SCHOOL TEACHERS educate students in early childhood (pre-primary), primary, middle or intermediate, secondary and special institutions by teaching a range of subjects within a prescribed curriculum, and promote students' social, emotional, intellectual and physical development.

Indicative Skill Level:

In Australia and New Zealand:

Most occupations in this minor group have a level of skill commensurate with a bachelor degree or higher qualification. At least five years of relevant experience may substitute for the formal qualification. In some instances relevant experience and/or on-the-job training may be required in addition to the formal qualification (ANZSCO Skill Level 1).

Tasks Include:

- selecting, preparing and presenting courses, teaching materials and lessons suited to the abilities of the students
- correcting and assessing students' work
- attending to the social, emotional, intellectual and physical wellbeing of students and maintaining acceptable standards of behaviour
- assessing the abilities and level of achievement of students through direct observation and administering tests
- discussing individual progress and problems with other professionals, parents and students
- performing extra-curricular tasks such as assisting with sport, school concerts, excursions and special interest programs
- supervising student teachers on placement
- maintaining attendance and scholastic records, and school discipline

Occupations in this minor group are classified into the following unit groups:

2411 Early Childhood (Pre-primary School) Teachers
2412 Primary School Teachers
2413 Middle School Teachers (Aus) / Intermediate School Teachers (NZ)
2414 Secondary School Teachers
2415 Special Education Teachers

UNIT GROUP 2411 EARLY CHILDHOOD (PRE-PRIMARY SCHOOL) TEACHERS

EARLY CHILDHOOD (PRE-PRIMARY SCHOOL) TEACHERS teach the basics of numeracy, literacy, music, art and literature to early childhood (pre-primary) students and promote students' social, emotional, intellectual and physical development.

Indicative Skill Level:

In Australia and New Zealand:

Most occupations in this unit group have a level of skill commensurate with a bachelor degree or higher qualification. In some instances relevant experience and/or on-the-job training may be required in addition to the formal qualification (ANZSCO Skill Level 1).

Registration or licensing may be required.

Tasks Include:

- planning and structuring learning in both indoor and outdoor environments using a variety of materials and equipment to facilitate students' development
- providing a variety of experiences and activities to develop motor skills, cooperative social skills, confidence and understanding
- promoting language development through story telling, role play, songs, rhymes and informal discussions held individually and within groups
- observing students to evaluate progress and to detect signs of ill health, emotional disturbance and other disabilities
- observing nutritional health, welfare and safety needs of students and identifying factors which may impede students' progress
- discussing students' progress with parents
- attending parent interviews, and staff and committee meetings
- participating in community and family support programs as appropriate
- supervising student teachers on placement

Occupations:

241111 Early Childhood (Pre-primary School) Teacher
241112 Kaiako Kōhanga Reo (Māori Language Nest Teacher)

241111 EARLY CHILDHOOD (PRE-PRIMARY SCHOOL) TEACHER

Alternative Title:

Kindergarten Teacher

Plans, organises and conducts activities to help pre-primary school students to develop a wide variety of skills including speech, reading, writing, motor skills and social interaction. Registration or licensing is required.

Skill Level: 1

Specialisation:

Preschool Director

241112 KAIAKO KŌHANGA REO (MĀORI LANGUAGE NEST TEACHER)

Works with whanau (family) to care for young children in a Kōhanga Reo (Māori language nest) and helps them understand Māori language and customs.

Skill Level: 1

UNIT GROUP 2412 PRIMARY SCHOOL TEACHERS

PRIMARY SCHOOL TEACHERS teach a range of subjects within a prescribed curriculum to primary school students and promote students' social, emotional, intellectual and physical development.

Indicative Skill Level:

In Australia and New Zealand:

Most occupations in this unit group have a level of skill commensurate with a bachelor degree or higher qualification. In some instances relevant experience and/or on-the-job training may be required in addition to the formal qualification (ANZSCO Skill Level 1).

Registration or licensing may be required.

Tasks Include:

- teaching literacy, numeracy, social science, creative expression and physical education skills to primary school students
- presenting prescribed curriculum using a range of teaching techniques and materials
- developing students' interests, abilities and coordination by way of creative activities
- guiding discussions and supervising work in class
- preparing, administering and marking tests, projects and assignments to evaluate students' progress and recording the results
- discussing individual progress and problems with students and parents, and seeking advice from Student Counsellors and senior teachers
- maintaining discipline in classrooms and other school areas
- participating in staff meetings, educational conferences and workshops
- liaising with parent, community and business groups
- maintaining class and scholastic records
- performing extra-curricular tasks such as assisting with sport, school concerts, excursions and special interest programs
- supervising student teachers on placement

Occupations:

241211 Kaiako Kura Kaupapa Māori (Māori-medium Primary School Teacher)
241212 Pouako Kura Kaupapa Māori (Māori-medium Primary School Senior Teacher)
241213 Primary School Teacher

241211 KAIAKO KURA KAUPAPA MĀORI (MĀORI-MEDIUM PRIMARY SCHOOL TEACHER)

Teaches and coordinates curriculum activities in the Māori language for children at primary school level.

Skill Level: 1

241212 POUAKO KURA KAUPAPA MĀORI (MĀORI-MEDIUM PRIMARY SCHOOL SENIOR TEACHER)

Teaches and coordinates curriculum activities in the Māori language for students at primary school level. This is a senior level teaching position.

Skill Level: 1

UNIT GROUP 2412 PRIMARY SCHOOL TEACHERS *continued*

241213 PRIMARY SCHOOL TEACHER

Teaches and coordinates a range of subjects within a prescribed curriculum to primary school students. Registration or licensing is required.

Skill Level: 1

Specialisation:
Primary School Teacher-Librarian

UNIT GROUP 2413 MIDDLE SCHOOL TEACHERS (AUS) / INTERMEDIATE SCHOOL TEACHERS (NZ)

MIDDLE SCHOOL TEACHERS (AUS) / INTERMEDIATE SCHOOL TEACHERS (NZ) teach one or more subjects within a prescribed curriculum to middle school or intermediate school students and promote students' social, emotional, intellectual and physical development.

Indicative Skill Level:

In Australia and New Zealand:

Most occupations in this unit group have a level of skill commensurate with a bachelor degree or higher qualification. In some instances relevant experience and/or on-the-job training may be required in addition to the formal qualification (ANZSCO Skill Level 1).

Registration or licensing is required.

Tasks Include:

- presenting prescribed curriculum using a range of teaching techniques and materials
- developing students' interests, abilities and coordination by way of creative activities
- guiding discussions and supervising work in class
- preparing, administering and marking tests, projects and assignments to evaluate students' progress and recording the results
- discussing individual progress and problems with students and parents, and seeking advice from Student Counsellors and senior teachers
- maintaining discipline in classrooms and other school areas
- participating in staff meetings, educational conferences and workshops
- liaising with parent, community and business groups
- maintaining class and scholastic records
- performing extra-curricular tasks such as assisting with sport, school concerts, excursions and special interest programs
- supervising student teachers on placement

Occupation:

241311 Middle School Teacher (Aus) / Intermediate School Teacher (NZ)

241311 MIDDLE SCHOOL TEACHER (AUS) / INTERMEDIATE SCHOOL TEACHER (NZ)

Teaches one or more subjects within a prescribed curriculum to middle school or intermediate school students and promotes students' social, emotional, intellectual and physical development. Registration or licensing is required.

Skill Level: 1

UNIT GROUP 2414 SECONDARY SCHOOL TEACHERS

SECONDARY SCHOOL TEACHERS teach one or more subjects within a prescribed curriculum to secondary school students and promote students' social, emotional, intellectual and physical development.

Indicative Skill Level:

In Australia and New Zealand:

Most occupations in this unit group have a level of skill commensurate with a bachelor degree or higher qualification. In some instances relevant experience and/or on-the-job training may be required in addition to the formal qualification (ANZSCO Skill Level 1).

Registration or licensing is required.

Tasks Include:

- presenting prescribed curriculum using a range of teaching techniques and materials
- developing students' interests, abilities and coordination by way of creative activities
- guiding discussions and supervising work in class
- preparing, administering and marking tests, projects and assignments to evaluate students' progress and recording the results
- discussing individual progress and problems with students and parents, and seeking advice from Student Counsellors and senior teachers
- maintaining discipline in classrooms and other school areas
- participating in staff meetings, educational conferences and workshops
- liaising with parent, community and business groups
- maintaining class and scholastic records
- performing extra-curricular tasks such as assisting with sport, school concerts, excursions and special interest programs
- supervising student teachers on placement

Occupation:

241411 Secondary School Teacher

241411 SECONDARY SCHOOL TEACHER

Teaches one or more subjects within a prescribed curriculum to secondary school students and promotes students' social, emotional, intellectual and physical development. Registration or licensing is required.

Skill Level: 1

Specialisation:

Secondary School Teacher-Librarian

UNIT GROUP 2415 SPECIAL EDUCATION TEACHERS

SPECIAL EDUCATION TEACHERS teach primary, middle or intermediate, and secondary school students with learning difficulties, hearing impairment and sight impairment, and promote students' social, emotional, intellectual and physical development.

Indicative Skill Level:

In Australia and New Zealand:

Most occupations in this unit group have a level of skill commensurate with a bachelor degree or higher qualification. In some instances relevant experience and/or on-the-job training may be required in addition to the formal qualification (ANZSCO Skill Level 1).

Registration or licensing is required.

Tasks Include:

- assessing students' abilities and limitations with regard to intellectual, physical, social and emotional disabilities, exceptional intellectual gifts, or specific problems of language and culture
- planning, organising and implementing special programs to provide remedial or advanced tuition
- administering various forms of assessment and interpreting the results
- teaching basic academic subjects, and practical and self-help skills to hearing and sight impaired students
- devising instructional materials, methods and aids to assist in training and rehabilitation
- advising, instructing and counselling parents and teachers on the availability and use of special techniques
- stimulating and developing interests, abilities, manual skills and coordination
- conferring with other staff members to plan and schedule lessons for special needs students
- preparing and maintaining student data and other records and submitting reports

Occupations:

241511 Special Needs Teacher
241512 Teacher of the Hearing Impaired
241513 Teacher of the Sight Impaired
241599 Special Education Teachers nec

241511 SPECIAL NEEDS TEACHER

Teaches academic and living skills to primary, middle or intermediate, and secondary school students with particular learning difficulties using various techniques, and promotes students' social, emotional, intellectual and physical development. Registration or licensing is required.

Skill Level: 1

Specialisations:

Behaviour Support Teacher
Remedial Teacher
Teacher of Gifted Students

241512 TEACHER OF THE HEARING IMPAIRED

Teaches academic and living skills to hearing impaired students, and promotes students' social, emotional, intellectual and physical development. Registration or licensing is required.

Skill Level: 1

UNIT GROUP 2415 SPECIAL EDUCATION TEACHERS *continued*

241513 TEACHER OF THE SIGHT IMPAIRED

Teaches academic and living skills to sight impaired students, and promotes students' social, emotional, intellectual and physical development. Registration or licensing is required.

Skill Level: 1

241599 SPECIAL EDUCATION TEACHERS NEC

This occupation group covers Special Education Teachers not elsewhere classified. Registration or licensing is required.

Skill Level: 1

Occupations in this group include:

- Aboriginal Education Teacher (Aus)
- Distance Education Teacher (Aus) / Correspondence School Teacher (NZ)
- Migrant Teacher

MINOR GROUP 242 TERTIARY EDUCATION TEACHERS

TERITARY EDUCATION TEACHERS educate tertiary students in one or more subjects within a prescribed course of study at universities, polytechnics, technical and further education (TAFE) institutes and other vocational training providers, and teach adult and community education courses.

Indicative Skill Level:

In Australia and New Zealand:

Most occupations in this minor group have a level of skill commensurate with a bachelor degree or higher qualification. At least five years of relevant experience may substitute for the formal qualification. In some instances relevant experience and/or on-the-job training may be required in addition to the formal qualification (ANZSCO Skill Level 1).

Tasks Include:

- preparing and delivering lectures to tertiary students in one or more subjects within a prescribed course of study
- conducting tutorials, seminars, and laboratory sessions
- preparing and marking essays, assignments and examinations
- participating in course development and in meetings on departmental, budgetary, curriculum and policy matters
- attending conferences and seminars, and conducting research into a particular field of knowledge
- providing feedback to students on progress and advising students on courses and related matters
- consulting with Education Managers, Librarians, Student Counsellors and other support staff

Occupations in this minor group are classified into the following unit groups:

2421 University Lecturers and Tutors

2422 Vocational Education Teachers (Aus) / Polytechnic Teachers (NZ)

UNIT GROUP 2421 UNIVERSITY LECTURERS AND TUTORS

UNIVERSITY LECTURERS AND TUTORS prepare and deliver lectures and conduct tutorials in one or more subjects within a prescribed course of study at a university and conduct research in a particular field of knowledge.

Indicative Skill Level:

In Australia and New Zealand:

Most occupations in this unit group have a level of skill commensurate with a bachelor degree or higher qualification. In some instances relevant experience and/or on-the-job training may be required in addition to the formal qualification (ANZSCO Skill Level 1).

Registration or licensing may be required.

Tasks Include:

- preparing and delivering lectures, and conducting tutorials, seminars and laboratory sessions
- preparing and marking essays, assignments and examinations
- advising students on academic and related matters
- attending departmental and faculty meetings, conferences and seminars
- supervising work programs of postgraduate and honours students and tutorial staff
- participating in setting course and degree requirements, curriculum revision and academic planning
- serving on council, senate, faculty and other committees and professorial boards
- conducting research and undertaking consultancies in a particular field of knowledge
- stimulating and guiding class discussions
- compiling bibliographies of specialised materials for reading assignments

Occupations:

242111 University Lecturer
242112 University Tutor

242111 UNIVERSITY LECTURER

Lectures students and conducts tutorials in one or more subjects within a prescribed course of study at a university and conducts research in a particular field of knowledge. Registration or licensing may be required.

Skill Level: 1

242112 UNIVERSITY TUTOR

Conducts tutorials in one or more subjects within a prescribed course of study at a university. Registration or licensing may be required.

Skill Level: 1

Specialisation:

University Demonstrator

UNIT GROUP 2422 VOCATIONAL EDUCATION TEACHERS (AUS) / POLYTECHNIC TEACHERS (NZ)

VOCATIONAL EDUCATION TEACHERS (AUS) / POLYTECHNIC TEACHERS (NZ) teach one or more subjects within a prescribed course of study at technical and further education (TAFE) institutes, polytechnics and other training institutes to tertiary students for vocational education and training purposes.

Indicative Skill Level:

In Australia and New Zealand:

Most occupations in this unit group have a level of skill commensurate with a bachelor degree or higher qualification or a postgraduate diploma in adult education. In some instances relevant industry and vocational education experience and/or on-the-job training may be required in addition to the formal qualification (ANZSCO Skill Level 1).

Registration or licensing is required.

Tasks Include:

- identifying the various needs of students and creating effective learning options to meet these needs
- liaising with individuals, industry and education sectors to ensure provision of relevant programs and services
- planning, designing and developing course curriculum and method of instruction
- advising students on courses and related matters
- teaching students using teaching aids including presentation of lesson materials, discussions, workshops, laboratory sessions, multimedia aids and computer tutorials
- marking and grading students' assignments, papers and exams and providing feedback to students about their progress
- maintaining records of students' progress, attendance and training activities
- consulting with Education Managers, Librarians, Student Counsellors and other support staff

Occupation:

242211 Vocational Education Teacher (Aus) / Polytechnic Teacher (NZ)

242211 VOCATIONAL EDUCATION TEACHER (AUS) / POLYTECHNIC TEACHER (NZ)

Teaches one or more subjects within a prescribed course of study at a technical and further education (TAFE) institute, polytechnic or other training institute to tertiary students for vocational education and training purposes. Registration or licensing is required.

Skill Level: 1

Specialisations:

Adult Education Teacher
TAFE Lecturer
TAFE Teacher
Workplace Trainer and Assessor

MINOR GROUP 249 MISCELLANEOUS EDUCATION PROFESSIONALS

This minor group covers Education Professionals not elsewhere classified.

It includes Education Advisers and Reviewers, Private Tutors and Teachers, and Teachers of English to Speakers of Other Languages.

Indicative Skill Level:
In Australia and New Zealand:

Most occupations in this minor group have a level of skill commensurate with a bachelor degree or higher qualification. At least five years of relevant experience may substitute for the formal qualification. In some instances relevant experience and/or on-the-job training may be required in addition to the formal qualification (ANZSCO Skill Level 1).

Occupations in this minor group are classified into the following unit groups:

2491 Education Advisers and Reviewers
2492 Private Tutors and Teachers
2493 Teachers of English to Speakers of Other Languages

UNIT GROUP 2491 EDUCATION ADVISERS AND REVIEWERS

EDUCATION ADVISERS AND REVIEWERS conduct educational research, develop course curricula and associated teaching materials for use by educational institutions, and review and examine the work of teachers and the results from curriculum programs in school settings.

Indicative Skill Level:

In Australia and New Zealand:

Most occupations in this unit group have a level of skill commensurate with a bachelor degree or higher qualification (ANZSCO Skill Level 1).

Tasks Include:

- consulting with teachers, principals and administrative officials of educational institutions to coordinate educational programs and provide advice
- identifying and evaluating developments in education by conducting research into educational systems
- serving on committees to identify present and future needs within the educational system, and planning, developing and modifying facilities and programs
- documenting subjects and courses developed, and evaluating new courses
- organising and conducting workshops and conferences to train teachers in new programs and methods
- applying knowledge of learning processes and school structures to develop operational and training programs, and submitting them for decision and funding
- visiting schools and observing teachers in the classroom, noting pupil response, motivation and teaching techniques
- discussing programs, records and teachers with School Principals to record academic performance of schools, welfare of pupils and performance of individual teachers
- making suggestions to government officials about improvements to educational facilities, equipment, buildings and staff to ensure continued standards of education

Occupations:

249111 Education Adviser

249112 Education Reviewer

249111 EDUCATION ADVISER

Conducts educational research and develops course curricula and associated teaching materials for use by educational institutions.

Skill Level: 1

Specialisations:

Curriculum Advisory Teacher

Education Officer

Home-School Liaison Officer

Preschool Adviser

249112 EDUCATION REVIEWER

Reviews and examines the work of teachers in classrooms and schools, and observes the results of the application of curriculum programs in primary, middle or intermediate school, or secondary educational institutions.

Skill Level: 1

Specialisation:

School Inspector

UNIT GROUP 2492 PRIVATE TUTORS AND TEACHERS

PRIVATE TUTORS AND TEACHERS teach students in the practice, theory and performance of subjects, such as art, dance, drama and music, in private training establishments.

Indicative Skill Level:
In Australia and New Zealand:

Most occupations in this unit group have a level of skill commensurate with a bachelor degree or higher qualification. At least five years of relevant experience may substitute for the formal qualification. In some instances relevant experience and/or on-the-job training may be required in addition to the formal qualification (ANZSCO Skill Level 1).

Tasks Include:

- planning programs of study for individual students and groups
- preparing and presenting material on the theory of the subject area
- instructing and demonstrating practical aspects of the subject area
- assigning problems and exercises relative to students' training needs and talents
- assessing students and offering advice, criticism and encouragement
- revising curricula, course content, course materials and methods of instruction
- preparing students for examinations, performance and assessments
- keeping abreast of developments in the subject area by attending professional conferences, seminars and courses, reading current literature, and talking with colleagues
- may arrange visits and tours to professional exhibitions and performances
- may organise for exhibitions or performances of students' work

Occupations:

249211 Art Teacher (Private Tuition)
249212 Dance Teacher (Private Tuition)
249213 Drama Teacher (Private Tuition)
249214 Music Teacher (Private Tuition)
249299 Private Tutors and Teachers nec

249211 ART TEACHER (PRIVATE TUITION)

Teaches students in the practice and theory of art in private training establishments.

Skill Level: 1

249212 DANCE TEACHER (PRIVATE TUITION)

Teaches students in the practice, theory and performance of dance in private training establishments.

Skill Level: 1

Specialisation:

Ballet Teacher (Private Tuition)

249213 DRAMA TEACHER (PRIVATE TUITION)

Teaches students in the practice, theory and performance of drama in private training establishments.

Skill Level: 1

Specialisation:

Elocution Teacher

UNIT GROUP 2492 PRIVATE TUTORS AND TEACHERS *continued*

249214 MUSIC TEACHER (PRIVATE TUITION)

Teaches students in the practice, theory and performance of music in private training establishments.

Skill Level: 1

Specialisation:
Singing Teacher (Private Tuition)

249299 PRIVATE TUTORS AND TEACHERS NEC

This occupation group covers Private Tutors and Teachers not elsewhere classified.

Skill Level: 1

Occupations in this group include:
Dressmaking Teacher (Private Tuition)
Handicrafts Teacher (Private Tuition)

UNIT GROUP 2493 TEACHERS OF ENGLISH TO SPEAKERS OF OTHER LANGUAGES

TEACHERS OF ENGLISH TO SPEAKERS OF OTHER LANGUAGES teach classes in English to students whose first language is a language other than English.

Indicative Skill Level:

In Australia and New Zealand:

Most occupations in this unit group have a level of skill commensurate with a bachelor degree or higher qualification (ANZSCO Skill Level 1).

Tasks Include:

- assessing the extent of language difficulties in students for whom English is a second language
- teaching students individually and in small groups out of the regular classroom, and assisting students within normal classroom settings
- teaching students English language skills using a variety of methods including lecture and visual demonstration
- providing assistance to other classroom teachers by designing special teaching programs for students with English language difficulties
- designing and producing teaching materials and adapting existing materials
- preparing course outlines and goals
- assigning lessons, correcting homework, and preparing and grading exams
- analysing, recording and reporting progress to regular classroom teachers, parents and students

Occupation:

249311 Teacher of English to Speakers of Other Languages

249311 TEACHER OF ENGLISH TO SPEAKERS OF OTHER LANGUAGES

Alternative Title:

English as a Second Language Teacher

Teaches classes in English to students whose first language is a language other than English.

Skill Level: 1

SUB-MAJOR GROUP 25 HEALTH PROFESSIONALS

HEALTH PROFESSIONALS develop health care programs and policies, conduct tests and diagnose and treat physical and physiological disorders, and provide nursing care, advice and counselling to patients to maintain, promote and restore good health and safe and healthy working environments.

Indicative Skill Level:

In Australia and New Zealand:

Most occupations in this sub-major group have a level of skill commensurate with a bachelor degree or higher qualification. In some instances relevant experience and/or on-the-job training may be required in addition to the formal qualification (ANZSCO Skill Level 1).

Tasks Include:

- examining patients to establish the nature of their complaint and performing or ordering diagnostic procedures
- selecting and administering appropriate treatment, medication and therapy
- prescribing prosthetic and corrective devices
- providing remedial therapy, rehabilitation and nursing care
- advising on individual, community and population health measures and health promotion, and safe working environments

Occupations in this sub-major group are classified into the following minor groups:

251 Health Diagnostic and Promotion Professionals
252 Health Therapy Professionals
253 Medical Practitioners
254 Midwifery and Nursing Professionals

MINOR GROUP 251 HEALTH DIAGNOSTIC AND PROMOTION PROFESSIONALS

HEALTH DIAGNOSTIC AND PROMOTION PROFESSIONALS conduct diagnostic tests and operate equipment to assess illnesses, incapacities and disabilities, provide health advice and develop programs and policies which promote good health, safe and healthy working environments, and administer pharmaceuticals.

Chiropractors and Osteopaths, Complementary Health Therapists, Dental Practitioners, Occupational Therapists, Physiotherapists, Podiatrists, and Speech Professionals and Audiologists are excluded from this minor group. They are included in Minor Group 252 Health Therapy Professionals.

Indicative Skill Level:

In Australia and New Zealand:

Most occupations in this minor group have a level of skill commensurate with a bachelor degree or higher qualification. In some instances relevant experience and/or on-the-job training may be required in addition to the formal qualification (ANZSCO Skill Level 1).

Tasks Include:

- developing, implementing, reviewing, examining, testing and raising awareness of diets, menus and nutrition intervention programs, the nature and extent of vision problems, and patients' medicine therapy
- planning, implementing and reviewing strategies and procedures for safe, economic and suitable disposal of various wastes, and for safe work practices
- conducting research and assessing data relating to health and nutrition status of individuals, groups and communities, and to develop and improve pharmaceuticals, cosmetics and related chemical products
- producing images to assist Medical Practitioners diagnose patients' illnesses and diseases, and administering radiation treatment
- consulting with other Health Professionals, Chemists, Engineering Professionals and other professionals

Occupations in this minor group are classified into the following unit groups:

2511 Dietitians
2512 Medical Imaging Professionals
2513 Occupational and Environmental Health Professionals
2514 Optometrists and Orthoptists
2515 Pharmacists
2519 Other Health Diagnostic and Promotion Professionals

UNIT GROUP 2511 DIETITIANS

DIETITIANS apply the science of human nutrition to help people understand the relationship between food and health and make appropriate dietary choices to attain and maintain health, and to prevent and treat illness and disease.

Indicative Skill Level:

In Australia and New Zealand:

Most occupations in this unit group have a level of skill commensurate with a bachelor degree or higher qualification (ANZSCO Skill Level 1).

Tasks Include:

- planning diets and menus, and instructing people on the requirements and importance of diet and on the planning and preparation of food
- supervising the preparation and serving of meals
- collecting, organising and assessing data relating to health and nutritional status of individuals, groups and communities
- monitoring food intake and quality to provide nutritional care
- calculating nutritional values of food served
- planning, conducting and evaluating nutrition intervention programs and compiling educational material
- providing nutrition assessments, nutrition management, and nutrition education, research and training
- consulting with other Health Professionals and related workers to manage the dietary and nutritional needs of patients

Occupation:

251111 Dietitian

251111 DIETITIAN

Alternative Title:

Nutritionist

Applies the science of human nutrition to help people understand the relationship between food and health and make appropriate dietary choices to attain and maintain health, and to prevent and treat illness and disease.

Skill Level: 1

UNIT GROUP 2512 MEDICAL IMAGING PROFESSIONALS

MEDICAL IMAGING PROFESSIONALS operate X-ray and other radiation producing and imaging equipment for diagnostic, monitoring and treatment purposes under the direction of Radiologists and other Medical Practitioners.

Indicative Skill Level:

In Australia and New Zealand:

Most occupations in this unit group have a level of skill commensurate with a bachelor degree or higher qualification (ANZSCO Skill Level 1).

Registration or licensing may be required.

Tasks Include:

- receiving referrals from Medical Practitioners to perform medical imaging and radiation treatment of patients
- determining the appropriate equipment to use, such as X-ray equipment, radiation scanners, fluoroscopes, ultrasound equipment, nuclear instrumentation, angiography equipment and computed tomography (CT) equipment, and selecting the appropriate equipment settings to provide the diagnostic information requested by Medical Practitioners
- calculating details of procedures such as length and intensity of exposure to radiation, size and strength of dosage of isotopes, and settings of recording equipment
- explaining procedures to patients and answering patients' inquiries about processes
- ensuring patients' welfare during procedures
- positioning patients, screens and equipment preparatory to procedures
- viewing the screen and deciding if images are satisfactory for diagnostic purposes, and selecting images to show Medical Practitioners
- conveying findings of procedures to Medical Practitioners

Occupations:

251211 Medical Diagnostic Radiographer
251212 Medical Radiation Therapist
251213 Nuclear Medicine Technologist
251214 Sonographer

251211 MEDICAL DIAGNOSTIC RADIOGRAPHER

Alternative Title:

Medical Imaging Technologist

Operates X-ray and other medical imaging equipment to produce images for medical diagnostic purposes in conjunction with Radiologists or other Medical Practitioners. Registration or licensing is required.

Skill Level: 1

Specialisation:

Magnetic Resonance Technologist

UNIT GROUP 2512 MEDICAL IMAGING PROFESSIONALS *continued*

251212 MEDICAL RADIATION THERAPIST

Operates high energy X-ray and other radiation and electron generating and monitoring equipment to administer radiation treatment for medical purposes in conjunction with Radiologists or other specialist Medical Practitioners. Registration or licensing is required.

Skill Level: 1

251213 NUCLEAR MEDICINE TECHNOLOGIST

Performs or assists in performing diagnostic examinations using radionuclides and radiopharmaceuticals, and administers radionuclides and radiopharmaceuticals for therapeutic purposes under the direction of Nuclear Medicine Specialists or other specialist Medical Practitioners. Registration or licensing is required.

Skill Level: 1

251214 SONOGRAPHER

Alternative Title:

Ultrasonographer

Operates ultrasound equipment to acquire, interpret and selectively record anatomical images, physical data and real-time physiological information for medical diagnostic purposes in conjunction with other Medical Practitioners.

Skill Level: 1

UNIT GROUP 2513 OCCUPATIONAL AND ENVIRONMENTAL HEALTH PROFESSIONALS

OCCUPATIONAL AND ENVIRONMENTAL HEALTH PROFESSIONALS develop, implement and evaluate policies and programs to monitor environmental health and occupational health and safety and related legislation to ensure safe and healthy working conditions, and assist injured staff through the workers' compensation and rehabilitation process.

Indicative Skill Level:

In Australia and New Zealand:

Most occupations in this unit group have a level of skill commensurate with a bachelor degree or higher qualification. In some instances relevant experience and/or on-the-job training may be required in addition to the formal qualification (ANZSCO Skill Level 1).

Registration or licensing may be required.

Tasks Include:

- developing, implementing and reviewing environmental health management plans and occupational health and safety plans
- preparing and implementing plans and strategies for the safe, economic and suitable disposal of commercial, industrial, medical and household wastes
- advising on and enforcing legislation, implementing prevention programs and strategies for communicable diseases, food safety, waste water treatment and disposal systems, recreation and domestic water quality, contaminated and hazardous substances, and minimising air, sea, water and noise pollution to improve health outcomes
- identifying hazards, and assessing and controlling risks in the workplace
- developing, implementing and monitoring programs minimising workplace and environmental pollution involving chemical and physical hazards
- promoting ergonomic principles within the workplace such as matching furniture, equipment and work activities to the needs of employees
- inspecting and auditing workplaces, processes, plant, and chemical and physical hazards for legislative compliance
- training employees in personal protective equipment and safe working procedures
- recording and investigating injuries and equipment damage, and reporting safety performance
- coordinating the return of injured workers into the workplace

Occupations:

251311 Environmental Health Officer
251312 Occupational Health and Safety Adviser

251311 ENVIRONMENTAL HEALTH OFFICER

Develops, enforces and evaluates environmental health policies, programs and strategies to improve health outcomes, and oversees the implementation and monitoring of environmental health legislation. Registration or licensing may be required.

Skill Level: 1

Specialisations:

Food Safety Auditor
Food Safety Officer

UNIT GROUP 2513 OCCUPATIONAL AND ENVIRONMENTAL HEALTH PROFESSIONALS *continued*

251312 OCCUPATIONAL HEALTH AND SAFETY ADVISER

Alternative Titles:

Occupational Health and Safety Coordinator
Occupational Health and Safety Officer

Develops, implements and evaluates risk management policies and programs, trains employees in occupational health and safety procedures, monitors and audits the workplace, and records and investigates incidents to ensure safe and healthy working conditions.

Skill Level: 1

Specialisations:

Occupational Hygienist
Workplace Rehabilitation Officer

UNIT GROUP 2514 OPTOMETRISTS AND ORTHOPTISTS

OPTOMETRISTS AND ORTHOPTISTS perform eye examinations and vision tests, prescribe lenses, other optical aids and therapy, and diagnose and manage eye movement disorders and associated sensory problems.

Indicative Skill Level:

In Australia and New Zealand:

Occupations in this unit group have a level of skill commensurate with a bachelor degree or higher qualification (ANZSCO Skill Level 1).

Registration or licensing is required.

Tasks Include:

- examining patients' eyes and setting tests to determine the nature and extent of vision problems and abnormalities
- assessing ocular health and visual function by measuring visual acuity and refractive error, and testing the function of visual pathways, visual fields, eye movements, freedom of vision and intraocular pressure, and performing other tests using special eye test equipment
- detecting, diagnosing and managing eye disease, referring patients to, and receiving referrals from other health providers, and prescribing medications for the treatment of eye disease
- diagnosing eye movement disorders and defects of binocular function
- prescribing lenses, contact lenses and low vision aids, and checking suitability and comfort
- prescribing exercises to coordinate movement and focusing of eyes
- managing programs for eye movement disorders, and instructing and counselling patients in the use of corrective techniques and eye exercises
- advising on visual health matters such as contact lens care, vision care for the elderly, optics, visual ergonomics, and occupational and industrial eye safety
- conducting preventative screening programs
- conducting rehabilitation programs for the visually impaired

Occupations:

251411 Optometrist
251412 Orthoptist

251411 OPTOMETRIST

Performs eye examinations and vision tests to determine the presence of visual, ocular and other abnormalities, ocular diseases and systemic diseases with ocular manifestations, and prescribes lenses, other optical aids, therapy and medication to correct and manage vision problems and eye diseases. Registration or licensing is required.

Skill Level: 1

251412 ORTHOPTIST

Diagnoses and manages eye movement disorders and associated sensory deficiencies. Registration or licensing is required.

Skill Level: 1

UNIT GROUP 2515 PHARMACISTS

PHARMACISTS ensure safe and quality use of medicines, and optimise health outcomes by contributing to selecting, prescribing, monitoring and evaluating medicine therapy, and researching, testing and developing pharmaceuticals and medical products.

Indicative Skill Level:

In Australia and New Zealand:

Occupations in this unit group have a level of skill commensurate with a bachelor degree or higher qualification and a one year traineeship (ANZSCO Skill Level 1).

Registration or licensing is required.

Tasks Include:

- receiving prescriptions, checking patients' medicine histories, and ensuring optimal dosage and methods of administration and drug compatibility before dispensing
- preparing or supervising the preparation and labelling of liquid medicines, ointments, powders, tablets and other medications to fill prescriptions
- advising prescribers on drug incompatibility and contra-indications
- reviewing and monitoring the medicine therapy of individual patients, and assessing the effectiveness of the total medicine therapy
- maintaining prescription files and recording issue of narcotics, poisons and habit-forming drugs
- storing and preserving vaccines, serums and other drugs subject to deterioration
- supplying non-prescription medicines, and diagnostic and therapeutic aids
- supervising and coordinating the work of Pharmacy Technicians, pharmacy interns and Pharmacy Sales Assistants
- conducting research to develop and improve pharmaceuticals, cosmetics and related chemical products
- conferring with Chemists, Engineering Professionals and other professionals about manufacturing techniques and ingredients
- testing and analysing drugs to determine their identity, purity and strength in relation to specified standards
- developing standards for drugs used in pharmaceuticals

Occupations:

251511 Hospital Pharmacist
251512 Industrial Pharmacist
251513 Retail Pharmacist

251511 HOSPITAL PHARMACIST

Prepares and dispenses pharmaceuticals, drugs and medicines in a hospital pharmacy. Registration or licensing is required.

Skill Level: 1

Specialisation:

Pharmaceutical Officer (Army)

251512 INDUSTRIAL PHARMACIST

Undertakes research, testing and analysis related to the development, production, storage, quality control and distribution of drugs and related supplies. Registration or licensing is required.

Skill Level: 1

UNIT GROUP 2515 PHARMACISTS *continued*

251513 RETAIL PHARMACIST

Alternative Title:

Community Pharmacist

Dispenses prescribed pharmaceuticals to the public, educates customers on health promotion, disease prevention and the proper use of medicines, and sells non-prescription medicines and related goods in a community pharmacy. Registration or licensing is required.

Skill Level: 1

UNIT GROUP 2519 OTHER HEALTH DIAGNOSTIC AND PROMOTION PROFESSIONALS

This unit group covers Health Diagnostic and Promotion Professionals not elsewhere classified.

It includes Health Promotion Officers and Orthotists or Prosthetists.

Indicative Skill Level:
In Australia and New Zealand:
Most occupations in this unit group have a level of skill commensurate with a bachelor degree or higher qualification. In some instances relevant experience and/or on-the-job training may be required in addition to the formal qualification (ANZSCO Skill Level 1).

Registration or licensing may be required.

Occupations:
251911 Health Promotion Officer
251912 Orthotist or Prosthetist
251999 Health Diagnostic and Promotion Professionals nec

251911 HEALTH PROMOTION OFFICER

Alternative Titles:
Community Health Worker
Health Educator

Assists health and community groups to improve the health of individuals and the community by raising awareness of healthy lifestyles, disease and disability, and other health-related issues.

Skill Level: 1

Specialisations:
Asthma Educator
Childbirth Educator
Diabetes Educator

251912 ORTHOTIST OR PROSTHETIST

Designs, builds, fits and repairs splints, braces, callipers, artificial limbs and related appliances to restore function or compensate for muscular and skeletal disabilities. Registration or licensing may be required.

Skill Level: 1

251999 HEALTH DIAGNOSTIC AND PROMOTION PROFESSIONALS NEC

This occupation group covers Health Diagnostic and Promotion Professionals not elsewhere classified.

Skill Level: 1

Occupations in this group include:
Genetic Counsellor

MINOR GROUP 252 HEALTH THERAPY PROFESSIONALS

HEALTH THERAPY PROFESSIONALS assess, diagnose and treat illnesses and disabilities, and provide therapeutic services such as chiropractic, osteopathy, complementary health, dentistry, occupational therapy, physical therapy, podiatry, speech pathology and audiology.

Dietitians, Medical Imaging Professionals, Occupational and Environmental Health Professionals, Optometrists and Orthoptists, Pharmacists, Health Promotion Officers, and Orthotists or Prosthetists are excluded from this minor group. They are included in Minor Group 251 Health Diagnostic and Promotion Professionals.

Indicative Skill Level:
In Australia and New Zealand:
Occupations in this minor group have a level of skill commensurate with a bachelor degree or higher qualification (ANZSCO Skill Level 1).

Tasks Include:
- questioning, examining, observing and testing patients to identify and determine nature of disease, disorder, illness or problem
- designing, developing and implementing treatment plans to address patients' problems
- providing exercise, dietary, lifestyle and hygiene guidelines and advice, adaptive equipment and correctional aids
- recording patients' medical histories such as previous injuries, surgeries, general health and lifestyle
- evaluating and documenting patients' treatment response and progress

Occupations in this minor group are classified into the following unit groups:
2521 Chiropractors and Osteopaths
2522 Complementary Health Therapists
2523 Dental Practitioners
2524 Occupational Therapists
2525 Physiotherapists
2526 Podiatrists
2527 Speech Professionals and Audiologists

UNIT GROUP 2521 CHIROPRACTORS AND OSTEOPATHS

CHIROPRACTORS AND OSTEOPATHS diagnose and treat physiological and mechanical disorders of the locomotor system and tissue strain, stress and dysfunction that impede normal neural, vascular and biochemical mechanisms, and provide advice on preventing these disorders.

Indicative Skill Level:

In Australia and New Zealand:

Occupations in this unit group have a level of skill commensurate with a bachelor degree or higher qualification (ANZSCO Skill Level 1).

Registration or licensing is required.

Tasks Include:

- administering a variety of neurological, musculoskeletal and functional tests to identify and assess physical problems and ailments of patients
- planning and discussing effective management of patients' dysfunction
- designing, reviewing, monitoring, assessing and evaluating treatment programs
- assisting and improving the function of all body systems such as musculoskeletal, neurological, cardiovascular, respiratory, gastrointestinal, endocrine and genitourinary systems
- recording detailed patient medical histories, treatments delivered and the patients' responses and progress to treatments
- referring patients to specialists and liaising with other Health Professionals in relation to patients' problems, needs and progress
- educating patients, their partners, family and friends in therapeutic procedures, such as home exercises and lifestyle changes, to enhance patients' health and wellbeing

Occupations:

252111 Chiropractor
252112 Osteopath

252111 CHIROPRACTOR

Diagnoses and treats physiological and mechanical disorders of the human locomotor system, particularly neuromuscular skeletal disorders, and provides advice on preventing these disorders. Registration or licensing is required.

Skill Level: 1

252112 OSTEOPATH

Diagnoses and treats tissue strains, stresses and dysfunctions which impede normal neural, vascular and biochemical mechanisms, and provides advice on preventing these disorders. Registration or licensing is required.

Skill Level: 1

UNIT GROUP 2522 COMPLEMENTARY HEALTH THERAPISTS

COMPLEMENTARY HEALTH THERAPISTS treat patients with physical, mental, spiritual and emotional needs by considering the whole person rather than focusing on specific symptoms and by using various therapies, techniques and practices.

Indicative Skill Level:

In Australia and New Zealand:

Occupations in this unit group have a level of skill commensurate with a bachelor degree or higher qualification (ANZSCO Skill Level 1).

Registration or licensing may be required.

Tasks Include:

- assessing patients to determine the nature of the disorder, illness, problem or need by questioning, examining and observing
- developing and implementing treatment plans using applications such as acupuncture, homoeopathic and herbal medicine, and dance, drama, hypnotic and music therapies
- evaluating and documenting patients' progress through treatment plans
- providing dietary and lifestyle advice and guidelines
- prescribing natural medicines, such as herbal, mineral and animal extracts, to stimulate the body's capacity for self-healing

Occupations:

252211 Acupuncturist
252212 Homoeopath
252213 Naturopath
252214 Traditional Chinese Medicine Practitioner
252215 Traditional Māori Health Practitioner
252299 Complementary Health Therapists nec

252211 ACUPUNCTURIST

Treats disorders and illnesses by stimulating the body's defence mechanisms through inserting fine needles into the skin. Registration or licensing may be required.

Skill Level: 1

252212 HOMOEOPATH

Treats the body's immune and defence systems by assessing the whole person and using minute amounts of natural remedies made from substances such as plants, minerals and animal sources. Registration or licensing may be required.

Skill Level: 1

252213 NATUROPATH

Treats internal health problems, metabolic disorders and imbalances through treatment of the whole person using natural therapies. Registration or licensing may be required.

Skill Level: 1

UNIT GROUP 2522 COMPLEMENTARY HEALTH THERAPISTS *continued*

252214 TRADITIONAL CHINESE MEDICINE PRACTITIONER

Alternative Titles:

- Chinese Medicine Practitioner
- Oriental Medicine Practitioner

Treats imbalances of energy flows through the body by assessing the whole person and using techniques and methods such as acupuncture, Chinese herbal medicine, massage, diet, exercise and breathing therapy. Registration or licensing may be required.

Skill Level: 1

Specialisation:

- Chinese Herbalist

252215 TRADITIONAL MĀORI HEALTH PRACTITIONER

Diagnoses, assesses and treats patients in accordance with Tikanga Māori (Māori culture and custom) and with the participation of whanau (family).

Skill Level: 1

252299 COMPLEMENTARY HEALTH THERAPISTS NEC

This occupation group covers Complementary Health Therapists not elsewhere classified.

Skill Level: 1

Occupations in this group include:

- Dance Therapist
- Drama Therapist
- Hypnotherapist
- Music Therapist

UNIT GROUP 2523 DENTAL PRACTITIONERS

DENTAL PRACTITIONERS diagnose and treat dental disease, restore normal oral function using a broad range of treatments, such as surgery and other specialist techniques, and advise on oral health.

Indicative Skill Level:
In Australia and New Zealand:
Occupations in this unit group have a level of skill commensurate with a bachelor degree or higher qualification (ANZSCO Skill Level 1).

Registration or licensing is required.

Tasks Include:

- diagnosing dental diseases using a range of methods such as radiographs, salivary tests and medical histories
- providing preventative oral health care such as periodontal treatments, fluoride applications and oral health promotion
- providing restorative oral care such as implants, complex crown and bridge restorations, and orthodontics, and repairing damaged and decayed teeth
- providing oral surgical treatments such as biopsy of tissue and prescription of medication
- performing routine orthodontic treatment
- restoring oral function with removable and fixed oral prostheses
- assisting in diagnosing general diseases having oral manifestations such as diabetes
- educating patients to take care of their mouth and teeth
- leading a dental team which may comprise Dental Hygienists, Dental Therapists, Dental Assistants and other Dental Specialists

Occupations:
252311 Dental Specialist
252312 Dentist

252311 DENTAL SPECIALIST

Diagnoses and treats diseases, injuries, irregularities and malformations of teeth and associated structures in the mouth and jaw using surgery and other specialist techniques. Registration or licensing is required.

Skill Level: 1

Specialisations:
Endodontist
Oral and Maxillofacial Surgeon
Oral Pathologist
Orthodontist
Paedodontist
Periodontist
Prosthodontist

UNIT GROUP 2523 DENTAL PRACTITIONERS *continued*

252312 DENTIST

Alternative Titles:

Dental Practitioner

Dental Surgeon

Diagnoses and treats dental disease, injuries, decay and malformations of the teeth, periodontal tissue (gums), hard and soft tissue found on the mouth and other dento-facial structures using surgery and other techniques. Registration or licensing is required.

Skill Level: 1

UNIT GROUP 2524 OCCUPATIONAL THERAPISTS

OCCUPATIONAL THERAPISTS assess functional limitations of people resulting from illnesses and disabilities, and provide therapy to enable people to perform their daily activities and occupations.

Indicative Skill Level:

In Australia and New Zealand:

Occupations in this unit group have a level of skill commensurate with a bachelor degree or higher qualification (ANZSCO Skill Level 1).

Registration or licensing may be required.

Tasks Include:

- assessing clients' emotional, psychological, developmental and physical capabilities using clinical observations and standardised tests
- assessing clients' functional potential in their home, leisure, work and school environments, and recommending environmental adaptations to maximise their performance
- planning and directing programs through the use of vocational, recreational, remedial, social and educational activities on an individual and group basis
- providing advice to family members, carers, employers and teachers about adapting clients' home, leisure, work and school environments
- providing adaptive equipment, such as wheel chairs and splints, to assist clients to overcome their functional limitations
- working with other Health Professionals in overall case management of clients
- working with other professionals in providing specialist advice to specific client groups such as those requiring driver rehabilitation, third-party compensation and medico-legal representation
- recording clients' progress and maintaining professional relationships in accordance with relevant legislative requirements and ethical guidelines

Occupation:

252411 Occupational Therapist

252411 OCCUPATIONAL THERAPIST

Assesses functional limitations of people resulting from illnesses and disabilities, and provides therapy to enable people to perform their daily activities and occupations. Registration or licensing may be required.

Skill Level: 1

UNIT GROUP 2525 PHYSIOTHERAPISTS

PHYSIOTHERAPISTS assess, treat and prevent disorders in human movement caused by injury or disease.

Indicative Skill Level:
In Australia and New Zealand:
Occupations in this unit group have a level of skill commensurate with a bachelor degree or higher qualification (ANZSCO Skill Level 1).

Registration or licensing is required.

Tasks Include:
- administering muscle, nerve, joint and functional ability tests to identify and assess physical problems of patients
- designing treatment programs to address patients' problems
- treating patients to reduce pain, improve circulation, strengthen muscles, improve cardiothoracic, cardiovascular and respiratory functions, restore joint mobility, and improve balance and coordination
- using the therapeutic properties of exercise, heat, cold, massage, manipulation, hydrotherapy, electrotherapy, ultraviolet and infra-red light and ultrasound in the treatment of patients
- reviewing, continually monitoring, assessing and evaluating programs and treatments
- consulting with other Health Professionals as required about patients' problems, needs and progress
- instructing patients and their families in procedures to be continued at home
- recording treatments given and patients' responses and progress
- developing and implementing screening and preventative health promotion programs

Occupation:
252511 Physiotherapist

252511 PHYSIOTHERAPIST

Alternative Title:
Physical Therapist

Assesses, treats and prevents disorders in human movement caused by injury or disease. Registration or licensing is required.

Skill Level: 1

Specialisations:
Aquatic Physiotherapist
Cardiothoracic Physiotherapist
Continence and Women's Health Physiotherapist
Gerentological Physiotherapist
Māori Physiotherapist (NZ)
Musculoskeletal Physiotherapist
Neurological Physiotherapist
Occupational Health Physiotherapist
Paediatric Physiotherapist
Sports Physiotherapist

UNIT GROUP 2526 PODIATRISTS

PODIATRISTS prevent, diagnose and treat disorders of the feet.

Indicative Skill Level:

In Australia and New Zealand:

Occupations in this unit group have a level of skill commensurate with a bachelor degree or higher qualification (ANZSCO Skill Level 1).

Registration or licensing is required.

Tasks Include:

- examining patients' feet to determine the nature and extent of conditions, deformities and injuries
- examining and treating foot disabilities caused by diseases such as diabetes, peripheral vascular disorders, rheumatoid arthritis and other neuropathies
- prescribing and arranging the fabrication of footwear to correct foot abnormalities
- performing minor surgery to remove and improve abnormal conditions
- prescribing and fitting replaceable pads, palliative and functional supports and other devices for the protection and correction of foot abnormalities
- advising patients about continued treatment and foot care
- may provide rehabilitation services to the physically handicapped
- may refer patients to or have patients referred from Medical Practitioners

Occupation:

252611 Podiatrist

252611 PODIATRIST

Prevents, diagnoses and treats disorder of the feet. Registration or licensing is required.

Skill Level: 1

Specialisation:

Podiatric Surgeon

UNIT GROUP 2527 SPEECH PROFESSIONALS AND AUDIOLOGISTS

SPEECH PROFESSIONALS AND AUDIOLOGISTS provide diagnostic assessment and rehabilitation services related to human hearing defects, and assess and treat people with communication disorders and physical problems associated with eating and swallowing.

Indicative Skill Level:

In Australia and New Zealand:

Occupations in this unit group have a level of skill commensurate with a bachelor degree or higher qualification. (ANZSCO Skill Level 1).

Registration or licensing is required.

Tasks Include:

- administering and interpreting a wide range of audiometric tests to determine hearing efficiency and locate sites of detected hearing problems
- interpreting audiometric test results alongside other medical, social and behavioural diagnostic data
- evaluating total response pattern and acoustic tests to distinguish between organic and non-organic hearing loss
- planning, directing and participating in counselling, speech reading and other rehabilitation programs
- prescribing appropriate hearing aids and instructing patients in use
- administering tests and observing patients to determine nature and extent of disorders
- planning and conducting programs of remedial exercise to correct disorders such as stuttering and abnormal articulation
- administering individual and group therapy for rehabilitation of patients with communication problems caused by defective hearing, cerebral palsy, surgery and injury
- advising on treatment for children with difficulties in learning to speak
- counselling and guiding language-handicapped individuals, their families, teachers and employers

Occupations:

252711 Audiologist

252712 Speech Pathologist (Aus) / Speech Language Therapist (NZ)

252711 AUDIOLOGIST

Provides diagnostic assessment and rehabilitative services related to human hearing defects. Registration or licensing is required.

Skill Level: 1

252712 SPEECH PATHOLOGIST (AUS) / SPEECH LANGUAGE THERAPIST (NZ)

Alternative Title:

Speech Therapist

Assesses and treats people with communication disorders, such as speech, language, voice, fluency and literacy difficulties, and people who have physical problems with eating or swallowing. Registration or licensing is required.

Skill Level: 1

MINOR GROUP 253 MEDICAL PRACTITIONERS

MEDICAL PRACTITIONERS diagnose physical and mental illnesses, disorders and injuries, provide medical care to patients, and prescribe and perform medical and surgical treatments to promote and restore good health.

Indicative Skill Level:

In Australia and New Zealand:

Occupations in this minor group have a level of skill commensurate with a bachelor degree or higher qualification and one to two years hospital-based training. In some instances at least five years specialist study and training is also required (ANZSCO Skill Level 1).

Tasks Include:

- examining patients to establish the nature of their complaints, and performing and ordering tests, X-rays and other diagnostic procedures
- determining diagnosis based on examination and results of tests
- selecting and administering appropriate treatments and therapies, and advising patients of further treatment options and preventative and therapeutic measures
- prescribing, administering, preparing and dispensing medication and prosthetic and corrective devices
- monitoring patients' progress and response to treatment
- recording patients' illnesses, treatment given and patients' responses and progress
- advising on diet, exercise and other measures to prevent and aid treatment of diseases and disorders

Occupations in this minor group are classified into the following unit groups:

2531 Generalist Medical Practitioners
2532 Anaesthetists
2533 Internal Medicine Specialists
2534 Psychiatrists
2535 Surgeons
2539 Other Medical Practitioners

UNIT GROUP 2531 GENERALIST MEDICAL PRACTITIONERS

GENERALIST MEDICAL PRACTITIONERS diagnose and treat physical and mental illnesses, disorders and injuries, recommend preventative action and refer patients to specialist Medical Practitioners, other health care workers, and social, welfare and support workers.

Indicative Skill Level:

In Australia and New Zealand:

Occupations in this unit group have a level of skill commensurate with a bachelor degree or higher qualification and at least one year of hospital-based training (ANZSCO Skill Level 1).

Registration or licensing is required.

Tasks Include:

- conducting examinations and questioning patients to determine the nature of disorders and illnesses, and recording patients' medical information
- ordering laboratory tests, X-rays and other diagnostic procedures, and interpreting findings to assist in diagnosis
- providing overall care for patients, and prescribing and administering treatments, medications and other remedial measures
- monitoring patients' progress and response to treatment
- advising on diet, exercise and other habits which aid prevention and treatment of disease and disorders
- referring patients to, and exchanging medical information with, specialist Medical Practitioners
- reporting births, deaths and notifiable diseases to government authorities
- arranging the admission of patients to hospitals

Occupations:

253111 General Medical Practitioner
253112 Resident Medical Officer

253111 GENERAL MEDICAL PRACTITIONER

Alternative Title:

General Practitioner

Diagnoses, treats and prevents human physical and mental disorders and injuries. Registration or licensing is required.

Skill Level: 1

Specialisation:

Medical Officer (Navy)

253112 RESIDENT MEDICAL OFFICER

Diagnoses, treats and prevents human physical and mental disorders and injuries under the supervision of medical specialists or senior general practitioners. Registration or licensing is required.

Skill Level: 1

Specialisation:

Medical Intern

UNIT GROUP 2532 ANAESTHETISTS

ANAESTHETISTS provide direct medical care to patients requiring general or local anaesthesia for surgical, diagnostic and other procedures such as prevention of pain and maintenance of body function. Anaesthetic Registrars training as Anaesthetists are included in this unit group.

Indicative Skill Level:

In Australia and New Zealand:

Occupations in this unit group have a level of skill commensurate with a bachelor degree or higher qualification, two years hospital-based training, and at least five years specialist study and training (ANZSCO Skill Level 1).

Registration or licensing is required.

Tasks Include:

- performing pre-operative examinations of patients to determine appropriate anaesthetic and sedation in concurrence with Internal Medicine Specialists and Surgeons
- discussing the anaesthetic process with patients and obtaining their informed consent prior to surgery
- administering local, regional and general anaesthetics using a variety of methods such as inhalational and intravenous administration
- supervising the transfer of patients to operating theatres, positioning on operating tables, keeping patients warm, and responding quickly and accurately if any problems arise
- monitoring patients throughout surgical procedures and in immediate post-operative procedures
- recording details of anaesthetic and sedation administered, and the condition of patients before, during and after anaesthesia
- liaising with other health care workers to provide diagnosis and treatment for patients with chronic pain, and to diagnose and treat patients requiring intensive care or resuscitation
- may instruct medical, nursing, student and ancillary staff on the signs, symptoms and diagnosis of allergic and anaphylactic reactions to anaesthetic agents, and supervision and treatment of life threatening emergencies

Occupation:

253211 Anaesthetist

253211 ANAESTHETIST

Provides direct medical care to patients requiring general or local anaesthesia for surgical, diagnostic and other procedures such as prevention of pain and maintenance of body function. Registration or licensing is required.

Skill Level: 1

Specialisations:

Intensive Care Anaesthetist
Obstetric Anaesthetist
Pain Management Specialist

UNIT GROUP 2533 INTERNAL MEDICINE SPECIALISTS

INTERNAL MEDICINE SPECIALISTS diagnose and treat internal human disorders and diseases using specialist testing, diagnostic and medical techniques. Medical Registrars training as Internal Medicine Specialists are included in this unit group.

Indicative Skill Level:

In Australia and New Zealand:

Occupations in this unit group have a level of skill commensurate with a bachelor degree or higher qualification, two years hospital-based training, and at least five years specialist study and training (ANZSCO Skill Level 1).

Registration or licensing is required.

Tasks Include:

- examining patients to determine the nature and extent of problems after referral from General Medical Practitioners and other medical specialists, and undertaking laboratory tests and diagnostic procedures
- analysing test results and other medical information to make diagnoses
- prescribing and administering drugs, and remedial and therapeutic treatment and procedures
- recording medical information and data
- reporting specified contagious and notifiable diseases to government health and immigration authorities
- may admit or refer patients to hospitals
- may consult other medical specialists

Occupations:

253311 Specialist Physician (General Medicine)
253312 Cardiologist
253313 Clinical Haematologist
253314 Clinical Oncologist
253315 Endocrinologist
253316 Gastroenterologist
253317 Intensive Care Specialist
253318 Neurologist
253321 Paediatrician
253322 Renal Medicine Specialist
253323 Rheumatologist
253324 Thoracic Medicine Specialist
253399 Internal Medicine Specialists nec

253311 SPECIALIST PHYSICIAN (GENERAL MEDICINE)

Investigates and diagnoses internal human disorders and diseases, and administers treatment. Registration or licensing is required.

Skill Level: 1

253312 CARDIOLOGIST

Investigates, diagnoses and treats diseases of the human heart. Registration or licensing is required.

Skill Level: 1

UNIT GROUP 2533 INTERNAL MEDICINE SPECIALISTS *continued*

253313 CLINICAL HAEMATOLOGIST

Investigates and diagnoses blood and other genetic disorders by studying cellular composition of blood and blood-producing tissues. Registration or licensing is required.

Skill Level: 1

253314 CLINICAL ONCOLOGIST

Manages and treats patients with cancer and tumours. Registration or licensing is required.

Skill Level: 1

253315 ENDOCRINOLOGIST

Investigates, diagnoses and treats disorders of the human glandular and hormonal systems. Registration or licensing is required.

Skill Level: 1

Specialisation:
- Diabetologist

253316 GASTROENTEROLOGIST

Investigates, diagnoses and treats diseases and disorders of the human liver, stomach and associated organs. Registration or licensing is required.

Skill Level: 1

253317 INTENSIVE CARE SPECIALIST

Alternative Titles:
- Intensive Care Medicine Specialist
- Intensivist

Investigates, diagnoses and treats patients in need of intensive and critical care. Registration or licensing is required.

Skill Level: 1

253318 NEUROLOGIST

Investigates, diagnoses and treats diseases and injuries of the human brain, spinal cord, nervous system and muscle tissue. Registration or licensing is required.

Skill Level: 1

253321 PAEDIATRICIAN

Investigates, diagnoses and treats internal diseases and disorders in children from birth up to, and including, adolescence. Registration or licensing is required.

Skill Level: 1

Specialisations:
- Neonatologist
- Paediatric Thoracic Physician

UNIT GROUP 2533 INTERNAL MEDICINE SPECIALISTS *continued*

253322 RENAL MEDICINE SPECIALIST

Alternative Titles:

- Nephrologist
- Renal Medicine Physician

Investigates, diagnoses and treats disorders of the human kidney. Registration or licensing is required.

Skill Level: 1

253323 RHEUMATOLOGIST

Investigates, diagnoses and treats diseases, injuries and deficiencies of human joints, muscles and soft tissue. Registration or licensing is required.

Skill Level: 1

253324 THORACIC MEDICINE SPECIALIST

Alternative Titles:

- Respiratory Medicine Physician
- Thoracic Medicine Physician

Investigates, diagnoses and treats diseases and disorders of the human respiratory system. Registration or licensing is required.

Skill Level: 1

Specialisations:

- Pulmonary Specialist
- Respiratory Physician

253399 INTERNAL MEDICINE SPECIALISTS NEC

This occupation group covers Internal Medicine Specialists not elsewhere classified. Registration or licensing is required.

Skill Level: 1

Occupations in this group include:

- Clinical Allergist
- Clinical Geneticist
- Clinical Immunologist
- Clinical Pharmacologist
- Geriatrician
- Industrial Medicine Specialist
- Infectious Diseases Specialist
- Palliative Medicine Specialist
- Rehabilitation Medicine Physician (Aus) / Musculoskeletal Specialist (NZ)
- Sexual Health Physician
- Sleep Medicine Specialist

UNIT GROUP 2534 PSYCHIATRISTS

PSYCHIATRISTS diagnose, assess, treat and prevent human mental, emotional and behavioural disorders. Psychiatric Registrars training as Psychiatrists are included in this unit group.

Indicative Skill Level:

In Australia and New Zealand:

Occupations in this unit group have a level of skill commensurate with a bachelor degree or higher qualification, two years hospital-based training, and at least five years specialist study and training (ANZSCO Skill Level 1).

Registration or licensing is required.

Tasks Include:

- assessing patients' mental and physical status to determine the nature and extent of mental, emotional and behavioural disorders
- assessing patients' medical, psychiatric and psychological histories
- examining patients to determine general physical condition
- ordering laboratory tests, imaging, neuropsychological tests and other diagnostic procedures
- examining the results of tests and examinations to determine the most appropriate forms of treatment
- prescribing and administering medication, psychotherapy, and other physical treatments and rehabilitation programs
- arranging admission to hospitals and providing in-patient treatment
- consulting, supervising and working with other Medical Practitioners and Health Professionals
- determining whether patients require involuntary treatment in accordance with relevant mental health acts
- assisting courts and other statutory bodies in managing patients in legal and forensic settings
- teaching medical students and registrars, and assessing their progress by administering tests

Occupation:

253411 Psychiatrist

253411 PSYCHIATRIST

Diagnoses, assesses, treats and prevents human mental, emotional and behavioural disorders. Registration or licensing is required.

Skill Level: 1

Specialisations:

Adolescent Psychiatrist
Child and Adolescent Psychiatrist
Child Psychiatrist
Forensic Psychiatrist
Geriatric Psychiatrist
Medical Psychotherapist

UNIT GROUP 2535 SURGEONS

SURGEONS perform surgery to correct deformities, repair injuries, prevent and treat diseases, and improve human functioning and appearance. Medical Registrars training as Surgeons are included in this unit group.

Indicative Skill Level:
In Australia and New Zealand:

Occupations in this unit group have a level of skill commensurate with a bachelor degree or higher qualification, two years hospital-based training, and at least five years specialist study and training (ANZSCO Skill Level 1).

Registration or licensing is required.

Tasks Include:
- examining patients to determine the necessity of operations, estimating and explaining risks to patients, and selecting the best operational procedures
- reviewing reports on patients' general physical condition, reactions to medications and medical histories
- consulting with Anaesthetists regarding the correct anaesthesia for patients
- performing surgical operations
- examining instruments, equipment, and surgical set-up to ensure that antiseptic and aseptic methods have been followed
- instructing other medical, nursing and associated staff regarding the preparation of patients and instrument and equipment requirements
- prescribing post-operative care, and observing and investigating patients' progress
- maintaining records of operations performed
- may specialise in particular types of operations

Occupations:

253511 Surgeon (General)
253512 Cardiothoracic Surgeon
253513 Neurosurgeon
253514 Orthopaedic Surgeon
253515 Otorhinolaryngologist
253516 Paediatric Surgeon
253517 Plastic and Reconstructive Surgeon
253518 Urologist
253521 Vascular Surgeon

253511 SURGEON (GENERAL)

Performs surgery to correct diseases and disorders covering a broad range of medical conditions. Registration or licensing is required.

Skill Level: 1

253512 CARDIOTHORACIC SURGEON

Performs heart and lung surgery. Registration or licensing is required.

Skill Level: 1

UNIT GROUP 2535 SURGEONS *continued*

253513 NEUROSURGEON

Performs surgery to correct disorders of the brain, spine and nervous system. Registration or licensing is required.

Skill Level: 1

253514 ORTHOPAEDIC SURGEON

Performs surgery to treat muscular and skeletal diseases and injuries. Registration or licensing is required.

Skill Level: 1

253515 OTORHINOLARYNGOLOGIST

Alternative Titles:
- Ear, Nose and Throat Specialist
- Head and Neck Surgeon

Performs surgery to correct diseases and disorders of the ear, nose and throat. Registration or licensing is required.

Skill Level: 1

Specialisations:
- Laryngologist
- Otologist
- Rhinologist

253516 PAEDIATRIC SURGEON

Provides surgical care and treatment to children from birth up to, and including, adolescence. Registration or licensing is required.

Skill Level: 1

253517 PLASTIC AND RECONSTRUCTIVE SURGEON

Performs surgery to repair and reconstruct muscle and tissue injuries and congenital deformities. Registration or licensing is required.

Skill Level: 1

253518 UROLOGIST

Provides medical and surgical treatment to patients with disorders of the kidney, urinary bladder and urethra, and treats disorders of the male sex organs. Registration or licensing is required.

Skill Level: 1

253521 VASCULAR SURGEON

Performs surgery to treat patients with conditions affecting their arteries and veins. Registration or licensing is required.

Skill Level: 1

UNIT GROUP 2539 OTHER MEDICAL PRACTITIONERS

This unit group covers Medical Practitioners not elsewhere classified.

It includes Dermatologists, Emergency Medicine Specialists, Obstetricians and Gynaecologists, Ophthalmologists, Pathologists and Radiologists. Medical Registrars training in these specialties are included in this unit group.

Indicative Skill Level:
In Australia and New Zealand:

Occupations in this unit group have a level of skill commensurate with a bachelor degree or higher qualification, two years hospital-based training, and at least five years specialist study and training (ANZSCO Skill Level 1).

Registration or licensing is required.

Occupations:

253911 Dermatologist
253912 Emergency Medicine Specialist
253913 Obstetrician and Gynaecologist
253914 Ophthalmologist
253915 Pathologist
253916 Radiologist
253999 Medical Practitioners nec

253911 DERMATOLOGIST

Provides diagnostic, treatment and preventative medical services related to disorders of the human skin. Registration or licensing is required.

Skill Level: 1

253912 EMERGENCY MEDICINE SPECIALIST

Alternative Title:

Emergency Physician

Provides diagnostic medical services, and manages patients with acute and urgent illness and injury. Registration or licensing is required.

Skill Level: 1

253913 OBSTETRICIAN AND GYNAECOLOGIST

Provides diagnostic, treatment and preventative medical and surgical services related to the care of women, foetuses and children during pregnancy and childbirth, and to disorders of the female genital, urinary, rectal and reproductive organs. Registration or licensing is required.

Skill Level: 1

Specialisations:

Gynaecological Oncologist
Reproductive Endocrinologist
Urogynaecologist

UNIT GROUP 2539 OTHER MEDICAL PRACTITIONERS *continued*

253914 OPHTHALMOLOGIST

Alternative Titles:

Eye Specialist
Eye Surgeon

Provides diagnostic, treatment and preventative medical services related to diseases, injuries and deficiencies of the human eye and associated structures. Registration or licensing is required.

Skill Level: 1

253915 PATHOLOGIST

Identifies the cause and processes of disease and illness by examining changes in body tissue and in blood and other body fluids, and conducts tests on samples of tissues, blood and body secretions. Registration or licensing is required.

Skill Level: 1

Specialisations:

Clinical Cytopathologist
Forensic Pathologist
Immunologist

253916 RADIOLOGIST

Provides diagnostic medical services, and medical care and management of patients utilising radiant energy techniques such as general radiography, angiography, fluoroscopy, mammography, ultrasound, computed tomography, magnetic resonance imaging, nuclear medicine and bone densitometry. Registration or licensing is required.

Skill Level: 1

Specialisations:

Diagnostic Radiologist
Medical Imaging Specialist
Nuclear Medicine Specialist
Radiation Oncologist

253999 MEDICAL PRACTITIONERS NEC

This occupation group covers Medical Practitioners not elsewhere classified. Registration or licensing is required.

Skill Level: 1

Occupations in this group include:

Occupational Medicine Specialist
Public Health Physician
Sports Physician

MINOR GROUP 254 MIDWIFERY AND NURSING PROFESSIONALS

MIDWIFERY AND NURSING PROFESSIONALS provide care to mothers and their babies, the elderly, and physically and mentally ill patients in hospitals, nursing homes, medical centres and the community; provide clinical education to midwives and nurses; conduct research into clinical nursing practice; and manage health service units and sub-units.

Indicative Skill Level:

In Australia and New Zealand:

Occupations in this minor group have a level of skill commensurate with a bachelor degree or higher qualification. In some instances relevant experience and/or on-the-job training may be required in addition to the formal qualification (ANZSCO Skill Level 1).

Tasks Include:

- assisting in examining patients, administering prescribed treatment, monitoring patients' progress, and facilitating lifestyle options and treatment plans in conjunction with patients' families, other carers and the community
- evaluating nurses' ongoing educational needs and planning relevant syllabus structures
- directing and controlling the allocation of human and material resources for a health service unit such as recruiting staff, human resource management, preparing budgets and financial management
- providing care and management of pregnancy and birth
- undertaking and promoting nursing and interdisciplinary research projects, and promoting uptake of findings into clinical nursing practice and patient management

Occupations in this minor group are classified into the following unit groups:

2541 Midwives
2542 Nurse Educators and Researchers
2543 Nurse Managers
2544 Registered Nurses

UNIT GROUP 2541 MIDWIVES

MIDWIVES provide care and advice to women during pregnancy, labour and childbirth, and postnatal care for women and babies in a range of settings such as the home, community, hospitals, clinics and health units.

Indicative Skill Level:

In Australia and New Zealand:

Occupations in this unit group have a level of skill commensurate with a bachelor degree or higher qualification (ANZSCO Skill Level 1).

Registration or licensing is required.

Tasks Include:

- providing advice and support during pre-conception, intrapartum, antenatal and postnatal periods in partnership with women
- providing care and management of pregnancy and birth
- assessing progress and recognising warning signs of abnormal and potentially abnormal pregnancies requiring referral to an Obstetrician
- monitoring the condition of women and foetuses during pregnancy and throughout labour
- conducting health education classes and seminars to promote the health of mothers and babies such as reproductive health, antenatal education, preparation for parenthood and breastfeeding
- providing advice on nutrition, childcare and family planning

Occupation:

254111 Midwife

254111 MIDWIFE

Alternative Titles:

Certified Midwife
Registered Midwife

Provides care and advice to women during pregnancy, labour and childbirth, and postnatal care for women and babies in a range of settings such as the home, community, hospitals, clinics and health units. Registration or licensing is required.

Skill Level: 1

Specialisations:

Community Midwife
Midwife Practitioner

UNIT GROUP 2542 NURSE EDUCATORS AND RESEARCHERS

NURSE EDUCATORS AND RESEARCHERS provide clinical and theoretical education to and promote professional development of nurses and midwives, and conduct research into nursing practice.

Indicative Skill Level:

In Australia and New Zealand:

Occupations in this unit group have a level of skill commensurate with a bachelor degree or higher qualification and at least five years relevant experience (ANZSCO Skill Level 1).

Tasks Include:

- researching, planning, developing and implementing nursing curricula
- facilitating practical experience for general and specialist nurses
- evaluating ongoing and changing educational needs and monitoring course outcomes
- participating in developing and implementing policies affecting nursing, nurse education and health
- undertaking and promoting nursing and interdisciplinary research projects, and disseminating research information
- promoting utilisation of current research findings into clinical nursing practice and patient management
- promoting the implementation of research and research findings into organisation-wide functions such as safety, quality and risk management
- providing support and education for other nurses undertaking research

Occupations:

254211 Nurse Educator
254212 Nurse Researcher

254211 NURSE EDUCATOR

Alternative Titles:

Clinical Nurse Educator
Staff Development Nurse

Designs, plans, implements and evaluates the delivery of nursing education and staff development programs, and manages educational resources.

Skill Level: 1

254212 NURSE RESEARCHER

Designs, conducts and evaluates nursing and interdisciplinary research projects, and promotes the implementation of research findings into clinical nursing practice.

Skill Level: 1

UNIT GROUP 2543 NURSE MANAGERS

NURSE MANAGERS manage health service units and sub-units of hospitals, aged care and community health care facilities, supervise nursing staff and financial resources to enable the provision of safe, cost effective nursing care within specified fields or for particular units, and monitor quality, clinical standards and professional development of nurses.

Directors of Nursing are excluded from this unit group. Directors of Nursing are included in Unit Group 1342 Health and Welfare Services Managers.

Indicative Skill Level:
In Australia and New Zealand:
Occupations in this unit group have a level of skill commensurate with a bachelor degree or higher qualification and at least five years relevant experience (ANZSCO Skill Level 1).

Registration or licensing is required.

Tasks Include:
- developing, implementing and monitoring policies and objectives of nursing care as they apply to units, staff and community groups
- coordinating the allocation of human and material resources for a health service unit such as recruitment of staff, human resource management, preparation of budgets and fiscal management
- monitoring and controlling the performance of nursing and support staff within the unit, and providing leadership
- initiating studies to evaluate the effectiveness of nursing services in the unit in relation to objectives, costs and nursing care
- promoting working relationships with community agencies and health and education providers
- contributing to organisational objectives in relation to quality, safety and risk management

Occupation:
254311 Nurse Manager

254311 NURSE MANAGER

Alternative Titles:
Charge Nurse
Nurse Supervisor
Nurse Unit Manager

Manages a health service unit or sub-unit of a hospital, aged care or community health care facility, supervises nursing staff and financial resources to enable the provision of safe, cost effective nursing care within a specified field or for a particular unit, and monitors quality, clinical standards and professional development of nurses. Registration or licensing is required.

Skill Level: 1

UNIT GROUP 2544 REGISTERED NURSES

REGISTERED NURSES provide nursing care to patients in hospitals, aged care and other health care facilities, and in the community.

Indicative Skill Level:

In Australia and New Zealand:

Occupations in this unit group have a level of skill commensurate with a bachelor degree or higher qualification. In some instances relevant experience and/or on-the-job training may be required in addition to the formal qualification (ANZSCO Skill Level 1).

Registration or licensing is required.

Tasks Include:

- assessing, planning, implementing and evaluating nursing care for patients according to accepted nursing practice and standards
- working in consultation with other Health Professionals and members of health teams, and coordinating the care of patients
- providing interventions, treatments and therapies such as medications, and monitoring responses to treatment and care plan
- promoting health and assisting in preventing ill health by participating in health education and other health promotion activities
- answering questions and providing information to patients and families about treatment and care
- supervising and coordinating the work of Enrolled Nurses and other health care workers

Occupations:

254411 Nurse Practitioner
254412 Registered Nurse (Aged Care)
254413 Registered Nurse (Child and Family Health)
254414 Registered Nurse (Community Health)
254415 Registered Nurse (Critical Care and Emergency)
254416 Registered Nurse (Developmental Disability)
254417 Registered Nurse (Disability and Rehabilitation)
254418 Registered Nurse (Medical)
254421 Registered Nurse (Medical Practice)
254422 Registered Nurse (Mental Health)
254423 Registered Nurse (Perioperative)
254424 Registered Nurse (Surgical)
254499 Registered Nurses nec

254411 NURSE PRACTITIONER

Provides advanced and extended nursing care to patients, such as ordering diagnostic tests, undertaking diagnosis and health assessments, prescribing patient care management, medicines and therapies, as authorised in relevant nursing legislation, and referring to specialist Medical Practitioners and other Health Professionals in a range of health, welfare and community settings. Registration or licensing is required.

Skill Level: 1

UNIT GROUP 2544 REGISTERED NURSES *continued*

254412 REGISTERED NURSE (AGED CARE)

Provides nursing care to the elderly in community settings, residential aged care facilities, retirement villages and health care facilities. Registration or licensing is required.

Skill Level: 1

Specialisation:

Registered Nurse (Gerontology)

254413 REGISTERED NURSE (CHILD AND FAMILY HEALTH)

Provides nursing care to children from birth to school age and their families with an emphasis on the prevention, early detection of, and early intervention in, physical, emotional and social problems affecting children and their families such as assistance with parentcraft, immunisation and developmental milestones. Registration or licensing is required.

Skill Level: 1

254414 REGISTERED NURSE (COMMUNITY HEALTH)

Provides nursing care, health counselling, screening and education to individuals, families and groups in the wider community with a focus on patient independence and health promotion. Registration or licensing is required.

Skill Level: 1

Specialisations:

Registered Nurse (Health Education and Promotion)
Registered Nurse (Public Health)
Registered Nurse (School Nurse)

254415 REGISTERED NURSE (CRITICAL CARE AND EMERGENCY)

Provides nursing care to critically ill patients and patients with unstable health following injury, surgery or during the acute phase of diseases, integrating new technological equipment into care in settings such as high dependency units, intensive care units, emergency departments or retrieval services. Registration or licensing is required.

Skill Level: 1

Specialisations:

Registered Nurse (Acute Care)
Registered Nurse (Emergency/Trauma)
Registered Nurse (High Dependency)
Registered Nurse (Neonatal Intensive Care)
Registered Nurse (Paediatric Intensive Care)

254416 REGISTERED NURSE (DEVELOPMENTAL DISABILITY)

Alternative Title:

Mental Retardation Nurse

Provides nursing care to people with intellectual and development disabilities in a range of health, welfare and community settings. Registration or licensing is required.

Skill Level: 1

UNIT GROUP 2544 REGISTERED NURSES *continued*

254417 REGISTERED NURSE (DISABILITY AND REHABILITATION)

Provides nursing care to patients recovering from injury and illness, and assists and facilitates patients with disabilities to live more independently. Registration or licensing is required.

Skill Level: 1

Specialisation:
- Registered Nurse (Rehabilitation)

254418 REGISTERED NURSE (MEDICAL)

Provides nursing care to patients with conditions, such as infections, metabolic disorders and degenerative conditions, which require medical intervention in a range of health, aged care and community settings. Registration or licensing is required.

Skill Level: 1

254421 REGISTERED NURSE (MEDICAL PRACTICE)

Alternative Titles:
- Practice Nurse
- Primary Health Care Nurse (NZ)

Provides clinical care to patients, undertakes clinical organisation and practice administration, and facilitates communication within a general practice environment and between the practice and outside organisations and individuals. Registration or licensing is required.

Skill Level: 1

254422 REGISTERED NURSE (MENTAL HEALTH)

Alternative Title:
- Psychiatric Nurse

Provides nursing care to patients with mental health illness, disorder and dysfunction, and those experiencing emotional difficulties, distress and crisis in health, welfare and aged care facilities, correctional services and the community. Registration or licensing is required.

Skill Level: 1

Specialisations:
- Psychopaedic Nurse (NZ)
- Registered Nurse (Child and Adolescence Mental Health)
- Registered Nurse (Drug and Alcohol)
- Registered Nurse (Psychiatric Rehabilitation)
- Registered Nurse (Psychogeriatric Care)

UNIT GROUP 2544 REGISTERED NURSES *continued*

254423 REGISTERED NURSE (PERIOPERATIVE)

Alternative Title:

Operating Room Nurse

Provides nursing care to patients before, during and immediately after surgery, assesses patients' condition, plans nursing care for surgical intervention, maintains a safe and comfortable environment, assists Surgeons and Anaesthetists during surgery, and monitors patients' recovery from anaesthetic, prior to return to, or discharge from, ward. Registration or licensing is required.

Skill Level: 1

Specialisations:

Registered Nurse (Anaesthetic)
Registered Nurse (Operating Room)
Registered Nurse (Recovery)

254424 REGISTERED NURSE (SURGICAL)

Provides nursing care to patients with injuries and illness that require surgical intervention. Registration or licensing is required.

Skill Level: 1

254499 REGISTERED NURSES NEC

This occupation group covers Registered Nurses not elsewhere classified. Registration or licensing is required.

Skill Level: 1

Occupations in this group include:

Nursing Officer (Defence Forces)
Registered Nurse (Infection Control)
Registered Nurse (Paediatrics)
Registered Nurse (Remote or Rural Area)

SUB-MAJOR GROUP 26 ICT PROFESSIONALS

ICT PROFESSIONALS perform analytical, conceptual and practical tasks which support the efficient and secure provision of information and communication technology (ICT) services to government, commercial and industrial organisations, and individuals.

Indicative Skill Level:

In Australia and New Zealand:

Most occupations in this sub-major group have a level of skill commensurate with a bachelor degree or higher qualification. At least five years of relevant experience and/or relevant vendor certification may substitute for the formal qualification. In some instances relevant experience and/or on-the-job training may be required in addition to the formal qualification (ANZSCO Skill Level 1).

Tasks Include:

- developing and documenting strategies, policies and procedures relating to the use of ICT technologies and services
- planning, analysing, designing, developing, implementing, testing, operating, maintaining and assisting with the use of technologies and services that enable information, such as voice, image and data, to be accessed, networked, stored, processed, transformed, manipulated and transmitted over a variety of media
- assessing the performance of ICT technologies and services, identifying limitations and inefficiencies, and recommending and implementing solutions
- providing troubleshooting and service support in diagnosing, resolving and correcting problems associated with the use of ICT technologies and service

Occupations in this sub-major group are classified into the following minor groups:

261 Business and Systems Analysts, and Programmers

262 Database and Systems Administrators, and ICT Security Specialists

263 ICT Network and Support Professionals

MINOR GROUP 261 BUSINESS AND SYSTEMS ANALYSTS, AND PROGRAMMERS

BUSINESS AND SYSTEMS ANALYSTS, AND PROGRAMMERS work with users to formulate system requirements, develop system plans and documentation, review and evaluate existing systems, and design and modify systems to meet users' business needs, create audiovisual applications, and develop, test and maintain code for computer applications and web sites.

Indicative Skill Level:

In Australia and New Zealand:

Most occupations in this minor group have a level of skill commensurate with a bachelor degree or higher qualification. At least five years of relevant experience and/or relevant vendor certification may substitute for the formal qualification. In some instances relevant experience and/or on-the-job training may be required in addition to the formal qualification (ANZSCO Skill Level 1).

Tasks Include:

- identifying, formulating and documenting user ICT requirements
- providing advice, guidance, expertise and assistance in the system project decision making process and in the development of system proposals and strategies
- identifying and evaluating inefficiencies, deficiencies and limitations in existing systems and associated processes, procedures and methods, and recommending optimal business practices, and system functionality and behaviour
- testing, debugging, diagnosing and correcting problems to ensure acceptable quality and integrity of the system, and that programs and applications perform to specification
- designing and developing digital animations, imaging, presentations, games, video clips, and Internet applications using multimedia software, tools and utilities, interactive graphics and programming language

Occupations in this minor group are classified into the following unit groups:

2611 ICT Business and Systems Analysts

2612 Multimedia Specialists and Web Developers

2613 Software and Applications Programmers

UNIT GROUP 2611 ICT BUSINESS AND SYSTEMS ANALYSTS

ICT BUSINESS AND SYSTEMS ANALYSTS work with users to formulate system requirements, develop system plans and documentation, review and evaluate existing systems, and design and modify systems to meet users' business needs.

Non-ICT Business Analysts (for example, Management Consultants) are excluded from this unit group. Non-ICT Business Analysts are included in Unit Group 2247 Management and Organisation Analysts.

Indicative Skill Level:

In Australia and New Zealand:

Most occupations in this unit group have a level of skill commensurate with a bachelor degree or higher qualification. At least five years of relevant experience and/or relevant vendor certification may substitute for the formal qualification. In some instances relevant experience and/or on-the-job training may be required in addition to the formal qualification (ANZSCO Skill Level 1).

Tasks Include:

- working with users to formulate and document business requirements
- identifying, investigating, and analysing business processes, procedures and work practices
- identifying and evaluating inefficiencies and recommending optimal business practices, and system functionality and behaviour
- using project management methodologies, principles and techniques to develop project plans and to cost, resource and manage projects
- taking responsibility for deploying functional solutions, such as creating, adopting and implementing system test plans, which ensure acceptable quality and integrity of the system
- creating user and training documentation, and conducting formal training classes
- developing functional specifications for use by system developers
- using data and process modelling techniques to create clear system specifications for the design and development of system software
- acting as a central reference and information source, providing guidance and assistance in the system project decision making process

Occupations:

261111 ICT Business Analyst
261112 Systems Analyst

261111 ICT BUSINESS ANALYST

Alternative Titles:

BA (ICT)
Business Consultant (ICT)

Identifies and communicates with users to formulate and produce a requirements specification to create system and software solutions.

Skill Level: 1

Specialisation:

Business Systems Analyst

261112 SYSTEMS ANALYST

Evaluates processes and methods used in existing ICT systems, proposes modifications, additional system components or new systems to meet user needs as expressed in specifications and other documentation.

Skill Level: 1

UNIT GROUP 2612 MULTIMEDIA SPECIALISTS AND WEB DEVELOPERS

MULTIMEDIA SPECIALISTS AND WEB DEVELOPERS create computer animation, audio, video and graphic image files for multimedia presentations, games, motion pictures, CD-ROMs, information kiosks and the web, and plan, produce and maintain web sites and web applications using web programming, scripting, authoring, content management and file transfer software.

Indicative Skill Level:

In Australia and New Zealand:

Most occupations in this unit group have a level of skill commensurate with a bachelor degree or higher qualification. At least five years of relevant experience and/or relevant vendor certification may substitute for the formal qualification. In some instances relevant experience and/or on-the-job training may be required in addition to the formal qualification (ANZSCO Skill Level 1).

Tasks Include:

- analysing, designing and developing Internet sites applying a mixture of artistry and creativity with software programming and scripting languages and interfacing with operating environments
- designing and developing digital animations, imaging, presentations, games, audio and video clips, and Internet applications using multimedia software, tools and utilities, interactive graphics and programming languages
- communicating with network specialists regarding web-related issues, such as security and hosting web sites, to control and enforce Internet and web server security, space allocation, user access, business continuity, web site backup and disaster recovery planning
- designing, developing and integrating computer code with other specialised inputs, such as image files, audio files and scripting languages, to produce, maintain and support web sites
- assisting in analysing, specifying and developing Internet strategies, web-based methodologies and development plans

Occupations:

261211 Multimedia Specialist
261212 Web Developer

261211 MULTIMEDIA SPECIALIST

Alternative Titles:

Multimedia Developer
Multimedia Programmer

Creates and manipulates computer animation, audio, video and graphic image files into multimedia programs to produce data and content for CD-ROMS, information kiosks, multimedia presentations, web sites, mobile telephone resources, electronic gaming environments, e-commerce and e-security solutions, and entertainment and education products.

Skill Level: 1

261212 WEB DEVELOPER

Alternative Title:

Web Programmer

Plans, produces and maintains web sites using web programming languages, software applications, technologies and databases together with specifications of user needs, often in conjunction with other ICT Professionals such as Business Analysts, Web Designers and network and usability specialists.

Skill Level: 1

UNIT GROUP 2613 SOFTWARE AND APPLICATIONS PROGRAMMERS

SOFTWARE AND APPLICATIONS PROGRAMMERS design, develop, test, maintain and document program code in accordance with user requirements, and system and technical specifications.

Indicative Skill Level:

In Australia and New Zealand:

Most occupations in this unit group have a level of skill commensurate with a bachelor degree or higher qualification. At least five years of relevant experience and/or relevant vendor certification may substitute for the formal qualification. In some instances relevant experience and/or on-the-job training may be required in addition to the formal qualification (ANZSCO Skill Level 1).

Tasks Include:

- researching, consulting, analysing and evaluating system program needs
- identifying technology limitations and deficiencies in existing systems and associated processes, procedures and methods
- testing, debugging, diagnosing and correcting errors and faults in an applications programming language within established testing protocols, guidelines and quality standards to ensure programs and applications perform to specification
- writing and maintaining program code to meet system requirements, system designs and technical specifications in accordance with quality accredited standards
- writing, updating and maintaining technical program, end user documentation and operational procedures
- providing advice, guidance and expertise in developing proposals and strategies for software design activities such as financial evaluation and costings for recommending software purchases and upgrades

Occupations:

261311 Analyst Programmer
261312 Developer Programmer
261313 Software Engineer
261399 Software and Applications Programmers nec

261311 ANALYST PROGRAMMER

Alternative Title:

Programmer Analyst

Analyses user needs, produces requirements documentation and system plans, and encodes, tests, debugs, maintains and documents programs and applications.

Skill Level: 1

UNIT GROUP 2613 SOFTWARE AND APPLICATIONS PROGRAMMERS *continued*

261312 DEVELOPER PROGRAMMER

Alternative Titles:

- Applications Developer
- ICT Developer
- ICT Programmer

Interprets specifications, technical designs and flow charts, builds, maintains and modifies the code for software applications, constructs technical specifications from a business functional model, and tests and writes technical documentation.

Skill Level: 1

Specialisations:

- Communications Programmer (Systems)
- Database Developer
- Database Programmer (Systems)
- Network Programmer
- Software Developer
- Software Programmer

261313 SOFTWARE ENGINEER

Alternative Titles:

- Software Architect
- Software Designer

Designs, develops, modifies, documents, tests, implements, installs and supports software applications and systems.

Skill Level: 1

Specialisations:

- Database Designer
- Systems Architect

261399 SOFTWARE AND APPLICATIONS PROGRAMMERS NEC

This occupation group covers Software and Applications Programmers not elsewhere classified.

Skill Level: 1

Occupations in this group include:

- Software Tester

MINOR GROUP 262 DATABASE AND SYSTEMS ADMINISTRATORS, AND ICT SECURITY SPECIALISTS

DATABASE AND SYSTEMS ADMINISTRATORS, AND ICT SECURITY SPECIALISTS plan, develop, maintain, manage and administer organisations' database management systems, operating systems and security policies and procedures to ensure optimal database and system integrity, security, backup, reliability and performance.

Indicative Skill Level:
In Australia and New Zealand:

Most occupations in this minor group have a level of skill commensurate with a bachelor degree or higher qualification. At least five years of relevant experience and/or relevant vendor certification may substitute for the formal qualification. In some instances relevant experience and/or on-the-job training may be required in addition to the formal qualification (ANZSCO Skill Level 1).

Tasks Include:

- designing and maintaining database architecture, data structures, tables, dictionaries and naming conventions to ensure the accuracy and completeness of all data master files
- performing the operational establishment and preventive maintenance of backups, recovery procedures, and enforcing security and integrity controls
- implementing and administering database documentation, guidelines, policies and procedures
- testing database systems and upgrades, such as debugging, tracking, reproduction, logging and resolving all identified problems, according to approved quality testing scripts, procedures and processes
- accepting responsibility for the processes, procedures and operational management associated with system security and disaster recovery planning
- liaising with security vendors, suppliers, service providers and external resources; analysing, recommending, installing and maintaining software security applications; and monitoring contractual obligations, performance delivery and service level agreements
- troubleshooting and providing service support in diagnosing, resolving and repairing server-related hardware and software malfunctions, encompassing workstations and communication infrastructure
- preparing and maintaining documentation, policies and instructions, and recording and detailing operational procedures and system logs
- ensuring that the design of computer sites allows all components to fit together and work properly, and monitoring and adjusting the performance of networks
- continually surveying the current computer site to determine future network needs and making recommendations for enhancements in the implementation of future servers and networks

Occupations in this minor group are classified into the following unit group:

2621 Database and Systems Administrators, and ICT Security Specialists

UNIT GROUP 2621 DATABASE AND SYSTEMS ADMINISTRATORS, AND ICT SECURITY SPECIALISTS

DATABASE AND SYSTEMS ADMINISTRATORS, AND ICT SECURITY SPECIALISTS plan, develop, maintain, manage and administer organisations' database management systems, operating systems and security policies and procedures to ensure optimal database and system integrity, security, backup, reliability and performance.

Indicative Skill Level:

In Australia and New Zealand:

Most occupations in this unit group have a level of skill commensurate with a bachelor degree or higher qualification. At least five years of relevant experience and/or relevant vendor certification may substitute for the formal qualification. In some instances relevant experience and/or on-the-job training may be required in addition to the formal qualification (ANZSCO Skill Level 1).

Tasks Include:

- designing and maintaining database architecture, data structures, tables, dictionaries and naming conventions to ensure the accuracy and completeness of all data master files
- performing the operational establishment and preventive maintenance of backups, recovery procedures, and enforcing security and integrity controls
- implementing and administering database documentation, guidelines, policies and procedures
- testing database systems and upgrades, such as debugging, tracking, reproduction, logging and resolving all identified problems, according to approved quality testing scripts, procedures and processes
- accepting responsibility for the processes, procedures and operational management associated with system security and disaster recovery planning
- liaising with security vendors, suppliers, service providers and external resources; analysing, recommending, installing and maintaining software security applications; and monitoring contractual obligations, performance delivery and service level agreements
- troubleshooting and providing service support in diagnosing, resolving and repairing server-related hardware and software malfunctions, encompassing workstations and communication infrastructure
- preparing and maintaining documentation, policies and instructions, and recording and detailing operational procedures and system logs
- ensuring that the design of computer sites allows all components to fit together and work properly, and monitoring and adjusting the performance of networks
- continually surveying the current computer site to determine future network needs and making recommendations for enhancements in the implementation of future servers and networks

Occupations:

262111 Database Administrator
262112 ICT Security Specialist
262113 Systems Administrator

UNIT GROUP 2621 DATABASE AND SYSTEMS ADMINISTRATORS, AND ICT SECURITY SPECIALISTS *continued*

262111 DATABASE ADMINISTRATOR

Alternative Titles:

Database Operator
Database Specialist
Database Support
DBA

Plans, develops, configures, maintains and supports an organisation's database management system in accordance with user requirements ensuring optimal database integrity, security, backup, reliability and performance.

Skill Level: 1

Specialisation:

Database Analyst

262112 ICT SECURITY SPECIALIST

Alternative Title:

Security Administrator

Establishes, manages and administers an organisation's ICT security policy and procedures to ensure preventive and recovery strategies are in place, and minimise the risk of internal and external security threats.

Skill Level: 1

Specialisation:

Information Technology Security Manager

262113 SYSTEMS ADMINISTRATOR

Alternative Title:

Systems Manager

Plans, develops, installs, troubleshoots, maintains and supports an operating system and associated server hardware, software and databases ensuring optimum system integrity, security, backup and performance.

Skill Level: 1

MINOR GROUP 263 ICT NETWORK AND SUPPORT PROFESSIONALS

ICT NETWORK AND SUPPORT PROFESSIONALS research, analyse, plan, design, install, monitor and maintain ICT systems to support the business needs of organisations and individuals.

Indicative Skill Level:
In Australia and New Zealand:

Most occupations in this minor group have a level of skill commensurate with a bachelor degree or higher qualification. At least five years of relevant experience and/or relevant vendor certification may substitute for the formal qualification. In some instances relevant experience and/or on-the-job training may be required in addition to the formal qualification (ANZSCO Skill Level 1).

Tasks Include:

- planning, designing, developing, configuring and commissioning networks and systems
- analysing, evaluating and monitoring network infrastructure to ensure networks are configured to operate at optimal performance
- troubleshooting and diagnosing network and system problems, and determining the most appropriate means of resolving problems and issues to improve system performance
- monitoring the overall performance of systems to assess the need for updates, upgrades, enhancements, preventive maintenance and new systems, and recommending options for upgrading and improving the performance of systems
- scheduling and conducting quality audit inspections

Occupations in this minor group are classified into the following unit groups:

2631 Computer Network Professionals
2632 ICT Support and Test Engineers
2633 Telecommunications Engineering Professionals

UNIT GROUP 2631 COMPUTER NETWORK PROFESSIONALS

COMPUTER NETWORK PROFESSIONALS research, analyse and recommend strategies for network architecture and development, implement, manage, maintain and configure network hardware and software, and monitor and optimise performance, and troubleshoot and provide user support.

Indicative Skill Level:

In Australia and New Zealand:

Most occupations in this unit group have a level of skill commensurate with a bachelor degree or higher qualification. At least five years of relevant experience and/or relevant vendor certification may substitute for the formal qualification. In some instances relevant experience and/or on-the-job training may be required in addition to the formal qualification (ANZSCO Skill Level 1).

Tasks Include:

- analysing, developing, interpreting and evaluating complex system design and architecture specifications, data models and diagrams in the development, configuration and integration of computer systems
- researching, analysing, evaluating and monitoring network infrastructure to ensure networks are configured to operate at optimal performance
- assessing and recommending improvements to network operations and integrated hardware, software, communications and operating systems
- providing specialist skills in supporting and troubleshooting network problems and emergencies
- installing, configuring, testing, maintaining and administering new and upgraded networks, software database applications, servers and workstations
- providing network programming in support of specific business needs and requirements
- preparing and maintaining procedures and documentation for network inventory, and recording diagnosis and resolution of network faults, enhancements and modifications to networks, and maintenance instructions
- monitoring network traffic, and activity, capacity and usage to ensure continued integrity and optimal network performance

Occupations:

263111 Computer Network and Systems Engineer
263112 Network Administrator
263113 Network Analyst

263111 COMPUTER NETWORK AND SYSTEMS ENGINEER

Plans, develops, deploys, tests and optimises network and system services, taking responsibility for configuration management and overall operational readiness of network systems, especially environments with multiple operating systems and configurations, and provides troubleshooting and fault-finding services for network problems.

Skill Level: 1

Specialisations:

Computer Network Engineer
Computer Systems Integrator

UNIT GROUP 2631 COMPUTER NETWORK PROFESSIONALS *continued*

263112 NETWORK ADMINISTRATOR

Alternative Titles:

Network Specialist
Network Support

Installs and maintains hardware and software, documents diagnosis and resolution of faults, manages user passwords, security and inventory documentation, ensures the efficient performance of servers, printers and personal computers, and attends to other operational tasks. May also perform tasks such as help desk support and user training.

Skill Level: 1

Specialisation:

LAN Administrator

263113 NETWORK ANALYST

Alternative Titles:

Network Consultant
Network Designer
Network Strategist

Researches and analyses network architecture, and recommends policies and strategies for designing, planning and coordinating an organisation's network such as the total system environment and architecture. May also perform operational tasks such as monitoring system performance, software and hardware upgrades, backups, support and network maintenance.

Skill Level: 1

Specialisation:

Network Architect

UNIT GROUP 2632 ICT SUPPORT AND TEST ENGINEERS

ICT SUPPORT AND TEST ENGINEERS develop procedures and strategies to support, create, maintain and manage technical quality assurance processes and guidelines and systems infrastructure, investigate, analyse and resolve system problems and performance issues, and test the behaviour, functionality and integrity of systems.

Indicative Skill Level:

In Australia and New Zealand:

Most occupations in this unit group have a level of skill commensurate with a bachelor degree or higher qualification. At least five years of relevant experience and/or relevant vendor certification may substitute for the formal qualification. In some instances relevant experience and/or on-the-job training may be required in addition to the formal qualification (ANZSCO Skill Level 1).

Tasks Include:

- scheduling and conducting quality audit inspections, and analysing and reviewing systems, data and documentation
- identifying variations and potential high risk areas in securing adherence to standards and procedures
- recommending corrective action plans and improvements in the resolution of non-compliance with standards detected through monitoring and auditing of processes and procedures
- communicating, educating and liaising with users and management to ensure awareness and adherence to standards, procedures and quality control issues and activities
- assisting in troubleshooting, diagnosing, testing and resolving system problems and issues
- developing, conducting and providing technical guidance and training in application software and operational procedures
- analysing, evaluating and diagnosing technical problems and issues such as installation, maintenance, repair, upgrade and configuration and troubleshooting of desktops, software, hardware, printers, Internet, email, databases, operating systems and security systems
- testing, identifying and diagnosing functionality errors and faults in systems, and programming code within established testing protocols, guidelines and quality standards to ensure systems perform to specification
- performing organisational systems architecture reviews and assessments, and recommending current and future hardware and software strategies and directions
- creating and reviewing technical documentation such as procedural, instructional and operational guides and manuals, technical reports and specifications and maintenance inventory systems

Occupations:

263211 ICT Quality Assurance Engineer
263212 ICT Support Engineer
263213 ICT Systems Test Engineer
263299 ICT Support and Test Engineers nec

UNIT GROUP 2632 ICT SUPPORT AND TEST ENGINEERS *continued*

263211 ICT QUALITY ASSURANCE ENGINEER

Alternative Titles:

- Quality Analyst (ICT)
- Quality Manager (ICT)
- Quality Specialist (ICT)

Creates, maintains and manages technical quality assurance processes and procedures to assess efficiency, validity, value and functional performance of computer systems and environments, and audits systems to ensure compliance with, and adherence to, accredited internal and external industry quality standards and regulations. May supervise the work of ICT quality assurance teams.

Skill Level: 1

Specialisations:

- Computer Systems Auditor
- Systems Auditor (ICT)

263212 ICT SUPPORT ENGINEER

Alternative Titles:

- Support Analyst
- Support Architect

Develops support procedures and strategies for systems, networks, operating systems and applications development, solves problems and provides technical expertise and direction in support of system infrastructure and process improvements, and diagnoses and resolves complex system problems.

Skill Level: 1

263213 ICT SYSTEMS TEST ENGINEER

Alternative Titles:

- Systems Tester
- Test Analyst (ICT)

Specifies, develops and writes test plans and test scripts, produces test cases, carries out regression testing, and uses automated test software applications to test the behaviour, functionality and integrity of systems, and documents the results of tests in defect reports and related documentation.

Skill Level: 1

263299 ICT SUPPORT AND TEST ENGINEERS NEC

This occupation group covers ICT Support and Test Engineers not elsewhere classified.

Skill Level: 1

Occupations in this group include:

- Usability Specialist

UNIT GROUP 2633 TELECOMMUNICATIONS ENGINEERING PROFESSIONALS

TELECOMMUNICATIONS ENGINEERING PROFESSIONALS design, construct, install, service and support telecommunications equipment, systems and facilities.

Indicative Skill Level:

In Australia and New Zealand:

Most occupations in this unit group have a level of skill commensurate with a bachelor degree or higher qualification. At least five years of relevant experience and/or relevant vendor certification may substitute for the formal qualification. In some instances relevant experience and/or on-the-job training may be required in addition to the formal qualification (ANZSCO Skill Level 1).

Tasks Include:

- planning, designing, building, configuring and commissioning telecommunications devices, networks and systems, such as voice, radio, two-way, data, microwave, satellite and digital data systems, and ensuring telecommunications systems interconnect with equipment from different manufacturers, service providers and users
- compiling engineering project proposals to define goals, identify scope, background and need, and ascertain cost of equipment, parts and services
- evaluating and procuring new products and services from vendors
- ensuring compliance with laws, regulations, policies and procedures in the provision of telecommunications systems
- selecting and developing new telecommunications sites by locating sites, filing documents, drawing up documents for approval, drafting construction drawings and following through to approval
- determining appropriate configurations of telecommunications hardware and software, ensuring desired performance of telecommunications equipment
- preparing and interpreting specifications, drawings and regulations for the use of telecommunications equipment
- determining the type and arrangement of circuits, transformers, circuit-breakers, transmission lines and equipment
- identifying and analysing problems and needs of existing telecommunications systems, such as interference, intelligibility and clarity, to determine the most appropriate means of reducing, eliminating and avoiding current and future problems and improve communications
- monitoring telecommunications systems to assess need for updates, upgrades, enhancements, preventive maintenance and new systems
- assessing performance levels of system hardware and software to project future needs, and developing short- and long-terms plans for updating equipment, adding capabilities, enhancing existing systems and providing improved telecommunications

Occupations:

263311 Telecommunications Engineer
263312 Telecommunications Network Engineer

263311 TELECOMMUNICATIONS ENGINEER

Designs and develops telecommunications systems, devices and products.

Skill Level: 1

Specialisation:

Signals Corps Officer (Army) (Aus) / Signals Officer (Army) (NZ)

UNIT GROUP 2633 TELECOMMUNICATIONS ENGINEERING PROFESSIONALS *continued*

263312 TELECOMMUNICATIONS NETWORK ENGINEER

Alternative Titles:

- Communications Consultant
- Communications Specialist (ICT)
- Telecommunications Consultant
- Telecommunications Specialist

Plans, designs, and monitors complex telecommunications networks and associated broadcasting equipment.

Skill Level: 1

SUB-MAJOR GROUP 27 LEGAL, SOCIAL AND WELFARE PROFESSIONALS

LEGAL, SOCIAL AND WELFARE PROFESSIONALS provide legal, social, vocational and spiritual advice to clients and the community, administer justice, and study human behaviour, society and institutions from current and historical perspectives.

Indicative Skill Level:

In Australia and New Zealand:

Most occupations in this sub-major group have a level of skill commensurate with a bachelor degree or higher qualification. At least five years of relevant experience may substitute for the formal qualification. In some instances relevant experience and/or on-the-job training may be required in addition to the formal qualification (ANZSCO Skill Level 1).

Tasks Include:

- assessing, discussing and assisting with the legal, social, vocational and spiritual needs of individuals and the community
- providing legal advice and written opinions on points of law
- presiding over judicial proceedings and pronouncing judgments
- assessing resources for health, welfare, recreation, housing, employment and other community services
- developing, administering and evaluating individual and group treatment programs
- assisting clients to understand and resolve problems
- preparing and conducting services of public worship and acknowledgements of faith in accordance with accepted ceremonial requirements
- undertaking historical and cultural research into human activity, and preparing reports of research findings

Occupations in this sub-major group are classified into the following minor groups:

271 Legal Professionals

272 Social and Welfare Professionals

MINOR GROUP 271 LEGAL PROFESSIONALS

LEGAL PROFESSIONALS provide legal advice, prepare and draft legal documents, conduct negotiations on behalf of clients, plead cases in courts and tribunals, hear legal and other matters in courts and tribunals, and interpret, analyse, administer and review the law.

Indicative Skill Level:
In Australia and New Zealand:
Most occupations in this minor group have a level of skill commensurate with a bachelor degree or higher qualification (ANZSCO Skill Level 1).

Tasks Include:
- receiving written information in the form of briefs and verbal instructions concerning legal cases
- researching statutes and previous court decisions relevant to cases
- providing advice and written opinions on points of law
- interviewing clients, and recommending and undertaking appropriate legal action
- preparing cases for court by conducting investigations, undertaking research, arranging witnesses, and giving notice of court actions
- representing clients in court, outlining the facts to the court, calling and questioning witnesses, and making addresses to the court to argue a client's case
- presiding over judicial proceedings and pronouncing judgments in courts of law
- acting individually and as members of administrative and industrial tribunals to resolve disputes

Occupations in this minor group are classified into the following unit groups:

2711 Barristers
2712 Judicial and Other Legal Professionals
2713 Solicitors

UNIT GROUP 2711 BARRISTERS

BARRISTERS plead cases before civil, criminal and industrial courts and other tribunals.

Indicative Skill Level:
In Australia and New Zealand:
Most occupations in this unit group have a level of skill commensurate with a bachelor degree or higher qualification (ANZSCO Skill Level 1).

Registration or licensing is required.

Tasks Include:
- receiving written information in the form of briefs and verbal instructions concerning cases from Solicitors, other specialist Legal Professionals and clients
- providing advice and written opinions on points of law
- conferring with clients and witnesses in preparation for court proceedings
- drawing up pleadings, affidavits and other court documents
- researching statutes and previous court decisions relevant to cases
- outlining the facts to the court, calling and questioning witnesses, and making addresses to the court to argue a client's case
- providing opinion on complex legal issues
- may draw up or settle documents

Occupation:
271111 Barrister

271111 BARRISTER

Pleads cases before civil, criminal and industrial courts and other tribunals. Registration or licensing is required.

Skill Level: 1

Specialisations:
Queen's Counsel
Senior Counsel (Aus)

UNIT GROUP 2712 JUDICIAL AND OTHER LEGAL PROFESSIONALS

JUDICIAL AND OTHER LEGAL PROFESSIONALS hear legal and other matters in courts and tribunals; interpret, analyse, administer and provide advice on the law; and draft legislation.

Indicative Skill Level:

In Australia and New Zealand:

Most occupations in this unit group have a level of skill commensurate with a bachelor degree or higher qualification and at least five years of relevant experience. Judges require appointment by the government or crown and must have been a Magistrate or an experienced legal practitioner of at least seven years standing. Magistrates must have been a legal practitioner of at least five years standing (ANZSCO Skill Level 1).

Registration or licensing may be required.

Tasks Include:

- researching statutes and previous court decisions relevant to cases
- conducting trials and hearings
- calling and questioning witnesses
- hearing and evaluating arguments and evidence in civil and criminal summary matters
- deciding penalties and sentences within statutory limits, such as fines, bonds and detention, awarding damages in civil matters, and issuing court orders
- exercising arbitral powers if resolution is not achieved or seems improbable through conciliation
- preparing settlement memoranda and obtaining signatures of parties
- advising government of legal, constitutional and parliamentary matters and drafting bills and attending committee meetings during consideration of bills
- advising clients and agents on legal and technical matters

Occupations:

271211 Judge
271212 Magistrate
271213 Tribunal Member
271299 Judicial and Other Legal Professionals nec

271211 JUDGE

Presides over civil and criminal proceedings in a court of law.

Skill Level: 1

Specialisations:

District Court Judge
Family Court Justice
High Court Justice
Supreme Court Judge
Youth Court Judge (NZ)

UNIT GROUP 2712 JUDICIAL AND OTHER LEGAL PROFESSIONALS *continued*

271212 MAGISTRATE

Alternative Title:
- Chamber Magistrate

Hears criminal matters to determine whether defendants will be committed for trial and adjudicates minor criminal offences without a jury.

Skill Level: 1

Specialisations:
- Children's Court Magistrate
- Industrial Court Magistrate
- Licensing Court Magistrate

271213 TRIBUNAL MEMBER

Hears industrial, administrative or other disputes to assist in resolving differences and to arbitrate on issues.

Skill Level: 1

Specialisations:
- Administrative Appeals Tribunal Member
- Industrial Relations Commissioner
- Waitangi Tribunal Member (NZ)

271299 JUDICIAL AND OTHER LEGAL PROFESSIONALS NEC

This occupation group covers Judicial and Other Legal Professionals not elsewhere classified. Registration or licensing may be required.

Skill Level: 1

Occupations in this group include:
- Administrative Lawyer
- Family Court Registrar (Aus)
- Judicial Registrar (Aus)
- Law Researcher
- Legal Officer
- Parliamentary Counsel
- Patent Attorney
- Trade Mark Attorney

UNIT GROUP 2713 SOLICITORS

SOLICITORS provide legal advice, prepare and draft legal documents, and conduct negotiations on behalf of clients on matters associated with the law.

Indicative Skill Level:

In Australia and New Zealand:

Most occupations in this unit group have a level of skill commensurate with a bachelor degree or higher qualification (ANZSCO Skill Level 1).

Registration or licensing is required.

Tasks Include:

- interviewing clients to determine the nature of problems, and recommending and undertaking appropriate legal action
- preparing cases for court by conducting investigations, undertaking research, arranging witness preparation and attendance, and giving notice of court actions
- representing clients in court
- managing conveyancing and other property matters by preparing contracts of sale, mortgage documents, lease documents and other documents relating to the transfer of land and buildings
- preparing and critically reviewing contracts between parties
- preparing wills
- providing advice on family law, company law, partnerships, commercial law and trusts
- may act as trustee or guardian
- may act as executor of clients' wills

Occupation:

271311 Solicitor

271311 SOLICITOR

Provides legal advice, prepares and drafts legal documents, and conducts negotiations on behalf of clients on matters associated with the law. Registration or licensing is required.

Skill Level: 1

MINOR GROUP 272 SOCIAL AND WELFARE PROFESSIONALS

SOCIAL AND WELFARE PROFESSIONALS provide guidance to clients and the community in social, educational, vocational, relationship and spiritual matters to enable them to overcome difficulties and to find and use resources to achieve particular goals; and study human behaviour, society and institutions from current and historical perspectives.

Indicative Skill Level:

In Australia and New Zealand:

Most occupations in this minor group have a level of skill commensurate with a bachelor degree or higher qualification. At least five years of relevant experience may substitute for the formal qualification. In some instances relevant experience and/or on-the-job training may be required in addition to the formal qualification (ANZSCO Skill Level 1).

Tasks Include:

- assessing resources for health, welfare, recreation, housing, employment and other community services
- providing support while exploring alternatives with clients who experience difficulties such as marital problems, unemployment, illness and drug abuse
- consulting with clients to develop rehabilitation plans taking account of vocational, social, medical and psychological needs
- assisting clients with employment placement and matters relating to education and training
- developing, administering and evaluating individual and group treatment programs
- preparing and conducting services of public worship and acknowledgments of faith, and performing marriages, funerals and special memorial services according to tradition and ecclesiastical and civil law
- undertaking historical and cultural research into human activity, and preparing reports of research findings

Occupations in this minor group are classified into the following unit groups:

2721 Counsellors
2722 Ministers of Religion
2723 Psychologists
2724 Social Professionals
2725 Social Workers
2726 Welfare, Recreation and Community Arts Workers

UNIT GROUP 2721 COUNSELLORS

COUNSELLORS provide information on vocational, relationship, social and educational difficulties and issues, and work with people to help them to identify and define their emotional issues through therapies such as cognitive behaviour therapy, interpersonal therapy and other talking therapies.

Indicative Skill Level:

In Australia and New Zealand:

Most occupations in this unit group have a level of skill commensurate with a bachelor degree or higher qualification. In some instances relevant experience and/or on-the-job training may be required in addition to the formal qualification (ANZSCO Skill Level 1).

Tasks Include:

- working with clients on career, study and employment options by obtaining and examining information relevant to their abilities and needs
- providing information and resources to assist clients with job-seeking skills
- assessing client needs in relation to treatment for drug and alcohol abuse
- conducting counselling interviews with individuals, couples and family groups
- assisting the understanding and adjustment of attitudes, expectations and behaviour to develop more effective interpersonal and marital relationships
- presenting alternative approaches and discussing potential for attitude and behaviour change
- consulting with clients to develop rehabilitation plans taking account of vocational and social needs
- contributing information, understanding and advice on the learning and behaviour of students, especially those with special needs, and assisting parents and teachers in dealing with these needs
- may work in a call centre

Occupations:

272111 Careers Counsellor
272112 Drug and Alcohol Counsellor
272113 Family and Marriage Counsellor
272114 Rehabilitation Counsellor
272115 Student Counsellor
272199 Counsellors nec

272111 CAREERS COUNSELLOR

Alternative Title:

Careers Adviser

Provides individuals and groups with information about career choices and assists individuals with self-development.

Skill Level: 1

272112 DRUG AND ALCOHOL COUNSELLOR

Provides support and treatment for people with drug and alcohol dependency problems, develops strategies which assist them to set goals and affect and maintain change, and provides community education. May work in a call centre.

Skill Level: 1

UNIT GROUP 2721 COUNSELLORS *continued*

272113 FAMILY AND MARRIAGE COUNSELLOR

Assists individuals, couples and families with marriage and relationship difficulties. May work in a call centre.

Skill Level: 1

Specialisations:
- Family Court Counsellor
- Family Mediator

272114 REHABILITATION COUNSELLOR

Assists physically, mentally and socially disadvantaged people to reintegrate into work and the community.

Skill Level: 1

272115 STUDENT COUNSELLOR

Alternative Title:
- School Counsellor

Provides information and assistance to students, parents and teachers about a wide range of matters such as students' personal problems, learning difficulties and special requirements.

Skill Level: 1

272199 COUNSELLORS NEC

This occupation group covers Counsellors not elsewhere classified. Occupations in this group may work in a call centre.

Skill Level: 1

Occupations in this group include:
- Gambling Counsellor
- Grief Counsellor
- Life Coach
- Rape Crisis Counsellor
- Trauma Counsellor

UNIT GROUP 2722 MINISTERS OF RELIGION

MINISTERS OF RELIGION perform spiritual functions associated with beliefs and practices of religious faiths, and provide motivation, guidance and training in religious life for the people of congregations and parishes, and the wider community.

Indicative Skill Level:

In Australia and New Zealand:

Most occupations in this unit group have a level of skill commensurate with a bachelor degree or higher qualification. The occupation in this unit group requires high levels of personal commitment and interest as well as, or in place of, formal qualifications or experience (ANZSCO Skill Level 1).

Registration or licensing may be required.

Tasks Include:

- preparing and conducting services of public worship and acknowledgments of faith
- preparing and delivering sermons, homilies and special talks, and planning music for services
- participating in the social and welfare activities of communities, encouraging people to be aware of their responsibilities, and organising participation in community projects
- conducting classes of religious instruction, and supervising prayer and discussion groups, retreats and seminars
- conducting premarital and family counselling and referring people to professional service agencies where necessary
- performing marriages, funerals and special memorial services according to tradition and ecclesiastical and civil law
- visiting members of the community in their homes, hospitals and other institutions to provide advice and religious comfort
- keeping records as required by the church and civil law

Occupation:

272211 Minister of Religion

272211 MINISTER OF RELIGION

Performs spiritual functions associated with beliefs and practices of a religious faith, and provides motivation, guidance and training in religious life for the people of a congregation or parish, and the wider community. This occupation requires high levels of personal commitment and interest as well as, or in place of, formal qualifications or experience. Registration or licensing may be required.

Skill Level: 1

Specialisations:

Aboriginal Ceremonial Celebrant (Aus)
Chaplain
Imam
Monk
Priest
Rabbi
Salvation Army Officer

UNIT GROUP 2723 PSYCHOLOGISTS

PSYCHOLOGISTS investigate, assess and provide treatment and counselling to foster optimal personal, social, educational and occupational adjustment and development.

Indicative Skill Level:

In Australia and New Zealand:

Most occupations in this unit group have a level of skill commensurate with a bachelor degree or higher qualification. In some instances relevant experience and/or on-the-job training may be required in addition to the formal qualification (ANZSCO Skill Level 1).

Registration or licensing may be required.

Tasks Include:

- collecting data about clients and assessing their cognitive, behavioural and emotional disorders
- administering and interpreting diagnostic tests and formulating plans for treatment
- developing, administering and evaluating individual and group treatment programs
- consulting with other professionals on details of cases and treatment plans
- conducting research studies of motivation in learning, group performance and individual differences in mental abilities and educational performance
- collecting data and analysing characteristics of students and recommending educational programs
- formulating achievement, diagnostic and predictive tests for use by teachers in planning methods and content of instruction
- developing interview techniques, psychological tests and other aids in workplace selection, placement, appraisal and promotion
- conducting surveys and research studies on job design, work groups, morale, motivation, supervision and management
- performing job analyses and establishing job requirements by observing and interviewing employees and managers

Occupations:

272311 Clinical Psychologist
272312 Educational Psychologist
272313 Organisational Psychologist
272314 Psychotherapist
272399 Psychologists nec

272311 CLINICAL PSYCHOLOGIST

Consults with individuals and groups, assesses psychological disorders and administers programs of treatment. Registration or licensing is required.

Skill Level: 1

Specialisations:

Forensic Psychologist
Health Psychologist
Neuropsychologist

UNIT GROUP 2723 PSYCHOLOGISTS *continued*

272312 EDUCATIONAL PSYCHOLOGIST

Investigates learning and teaching, and develops psychological techniques to foster the development and skills of individuals and groups in educational settings. Registration or licensing is required.

Skill Level: 1

272313 ORGANISATIONAL PSYCHOLOGIST

Alternative Titles:
- Industrial Psychologist
- Occupational Psychologist

Applies psychological principles and techniques to study occupational behaviour, working conditions and organisational structure, and solve problems of work performance and organisational design. Registration or licensing is required.

Skill Level: 1

272314 PSYCHOTHERAPIST

Provides diagnosis and treatment of mental and emotional disorders using psychotherapeutic methods such as behavioural therapy, biofeedback, relaxation therapy and other techniques.

Skill Level: 1

Specialisation:
- Art Psychotherapist or Therapist

272399 PSYCHOLOGISTS NEC

This occupation group covers Psychologists not elsewhere classified. Registration or licensing is required.

Skill Level: 1

Occupations in this group include:
- Community Psychologist
- Counselling Psychologist
- Sport Psychologist

UNIT GROUP 2724 SOCIAL PROFESSIONALS

SOCIAL PROFESSIONALS research and study human behaviour, society and institutions from current and historical perspectives, and verbally render spoken statements, and transcribe text and recorded spoken material from one language into another.

Indicative Skill Level:

In Australia and New Zealand:

Most occupations in this unit group have a level of skill commensurate with a bachelor degree or higher qualification (ANZSCO Skill Level 1).

Tasks Include:

- assembling historical data by consulting sources of information such as historical indexes and catalogues, archives, court records, diaries, newspaper files and other materials
- organising, authenticating, evaluating and interpreting historical, political, sociological, anthropological and linguistic data
- undertaking historical and cultural research into human activity, and preparing and presenting research findings
- providing simultaneous and consecutive verbal or signed renditions of speeches into another language
- rendering the meaning and feeling of what is said and signed into another language in the appropriate register and style in a range of settings such as courts, hospitals, schools, workplaces and conferences
- studying original texts and transcripts of recorded spoken material to comprehend subject matter and translating them into another language
- rendering the meaning and feeling of written material, such as literary, legal, technical and scientific texts, into another language in the appropriate register and style, so that it will read as an original piece rather than as a translation

Occupations:

272411 Historian
272412 Interpreter
272413 Translator
272499 Social Professionals nec

272411 HISTORIAN

Researches the history of human activity and prepares accounts of findings.

Skill Level: 1

Specialisations:

Art Historian
Cultural Historian
Economic Historian
Geographical Historian

272412 INTERPRETER

Transfers a spoken or signed language into another spoken or signed language, usually within a limited time frame in the presence of the participants requiring the translation.

Skill Level: 1

UNIT GROUP 2724 SOCIAL PROFESSIONALS *continued*

272413 TRANSLATOR

Transfers a source text from one language into another, usually within an extended time frame to allow for corrections and modifications and without the presence of the participants requiring the translation.

Skill Level: 1

272499 SOCIAL PROFESSIONALS NEC

This occupation group covers Social Professionals not elsewhere classified.

Skill Level: 1

Occupations in this group include:

- Anthropologist
- Archaeologist
- Criminologist
- Ethnographer
- Geographer
- Heritage Consultant
- Linguist
- Parole Board Member
- Political Scientist
- Prehistorian
- Sociologist
- Transport Analyst

UNIT GROUP 2725 SOCIAL WORKERS

SOCIAL WORKERS assess the social needs of individuals, families and groups, assist and empower people to develop and use the skills and resources needed to resolve social and other problems, and further human wellbeing and human rights, social justice and social development.

Indicative Skill Level:

In Australia and New Zealand:

Most occupations in this unit group have a level of skill commensurate with a bachelor degree or higher qualification. In some instances relevant experience and/or on-the-job training may be required in addition to the formal qualification (ANZSCO Skill Level 1).

Registration or licensing may be required.

Tasks Include:

- acting as a facilitator between clients in need and community services
- assessing resources for health, welfare, recreation, housing, employment and other community services
- providing leadership and assistance for the implementation of pilot projects in community development and self-help, and planning and implementing research projects to address client needs, organisation goals and social policy
- cooperating with community organisations, social agencies and voluntary groups to improve services and develop new services
- conducting individual and family case interviews to identify the nature and extent of clients' problems
- assisting clients to understand and resolve problems by providing information, acting as a mediator and referring them to community and self-help agencies
- analysing, developing, promoting and implementing social policies through the use of practice experience, research, analytic frameworks, and negotiation skills to respond to social need through a fair, equitable and effective allocation of social resources
- monitoring the progress of clients by maintaining contact
- compiling case records and reports

Occupation:

272511 Social Worker

272511 SOCIAL WORKER

Assesses the social needs of individuals, families and groups, assists and empowers people to develop and use the skills and resources needed to resolve social and other problems, and furthers human wellbeing and human rights, social justice and social development. Registration or licensing may be required.

Skill Level: 1

UNIT GROUP 2726 WELFARE, RECREATION AND COMMUNITY ARTS WORKERS

WELFARE, RECREATION AND COMMUNITY ARTS WORKERS design and implement strategies and programs to meet community and individual needs and assist individuals, families and groups with social, emotional and financial difficulties to improve quality of life by educating and supporting them and working towards change in their social environment.

Indicative Skill Level:

In Australia and New Zealand:

Most occupations in this unit group have a level of skill commensurate with a bachelor degree or higher qualification. At least five years of relevant experience may substitute for the formal qualification. In some instances relevant experience and/or on-the-job training may be required in addition to the formal qualification (ANZSCO Skill Level 1).

Tasks Include:

- researching and analysing community issues, needs and problems
- developing, evaluating and maintaining community resources and programs
- evaluating data and writing reports such as submissions requesting funding for continuing programs and new projects
- identifying issues of local need, concerns and aspirations through community consultation
- organising local sporting, cultural and recreational events and activities such as community functions, hobby classes, community arts projects and sporting competitions
- providing support while exploring alternatives with clients who experience difficulties such as marital problems, unemployment, illness and drug abuse
- assessing risks and providing intensive short-term crisis counselling for victims of domestic violence, child abuse, disasters and other crises
- assisting to establish and administer neighbourhood houses, community groups, employment training programs and other services

Occupations:

272611 Community Arts Worker
272612 Recreation Officer (Aus) / Recreation Coordinator (NZ)
272613 Welfare Worker

272611 COMMUNITY ARTS WORKER

Alternative Titles:

Community Artist
Community Arts Officer

Identifies issues of local need, concerns and aspirations through community consultation, and designs and implements strategies to facilitate and encourage community arts projects and happenings, and promote the value of community cultural development.

Skill Level: 1

Specialisation:

Community Cultural Development Officer

UNIT GROUP 2726 WELFARE, RECREATION AND COMMUNITY ARTS WORKERS *continued*

272612 RECREATION OFFICER (AUS) / RECREATION COORDINATOR (NZ)

Alternative Title:

Recreation Adviser

Plans, organises and coordinates recreation facilities and programs through organisations such as local governments, schools, church bodies and youth organisations.

Skill Level: 1

272613 WELFARE WORKER

Alternative Title:

Welfare Case Worker

Assists individuals, families and groups with social, emotional or financial difficulties to improve quality of life, by educating and supporting them and working towards change in their social environment.

Skill Level: 1

MAJOR GROUP 3 TECHNICIANS AND TRADES WORKERS

TECHNICIANS AND TRADES WORKERS perform a variety of skilled tasks, applying broad or in-depth technical, trade or industry specific knowledge, often in support of scientific, engineering, building and manufacturing activities.

Indicative Skill Level:
Most occupations in this major group have a level of skill commensurate with the qualifications and experience outlined below.

In Australia:

AQF Associate Degree, Advanced Diploma or Diploma, or at least three years of experience (ANZSCO Skill Level 2); or

AQF Certificate III including at least two years of on-the-job training, or AQF Certificate IV or at least three years of relevant experience (ANZSCO Skill Level 3)

In New Zealand:

NZ Register Diploma, or at least three years of relevant experience (ANZSCO Skill Level 2); or

NZ Register Level 4 qualification, or at least three years of relevant experience (ANZSCO Skill Level 3)

In some instances relevant experience and/or on-the-job training may be required in addition to the formal qualification.

Tasks Include:

- carrying out tests and experiments, and providing technical support to Health Professionals, Natural and Physical Science Professionals and Engineering Professionals
- providing technical support to users of computer hardware and software
- fabricating, repairing and maintaining metal, wood, glass and textile products
- repairing and maintaining motor vehicles, aircraft, marine craft and electrical and electronic machines and equipment
- constructing, repairing, fitting-out and finishing buildings and other structures
- operating printing and binding equipment
- preparing and cooking food
- shearing, caring for, training and grooming animals, and assisting Veterinarians
- propagating and cultivating plants, and establishing and maintaining turf surfaces for sporting events
- cutting and styling hair
- operating chemical, gas, petroleum and power generation equipment
- providing technical assistance for the production, recording and broadcasting of artistic performances

Occupations in this major group are classified into the following sub-major groups:

31 Engineering, ICT and Science Technicians
32 Automotive and Engineering Trades Workers
33 Construction Trades Workers
34 Electrotechnology and Telecommunications Trades Workers
35 Food Trades Workers
36 Skilled Animal and Horticultural Workers
39 Other Technicians and Trades Workers

SUB-MAJOR GROUP 31 ENGINEERING, ICT AND SCIENCE TECHNICIANS

ENGINEERING, ICT AND SCIENCE TECHNICIANS perform tests and experiments, and provide technical support to Professionals engaged in research, design and development in the areas of agriculture, medicine, science, building, engineering, ICT and telecommunications.

Indicative Skill Level:
Most occupations in this sub-major group have a level of skill commensurate with the qualifications and experience outlined below.

In Australia:
AQF Associate Degree, Advanced Diploma or Diploma (ANZSCO Skill Level 2)

In New Zealand:
NZ Register Diploma (ANZSCO Skill Level 2)

At least three years of relevant experience may substitute for the formal qualifications listed above. In some instances relevant experience and/or on-the-job training may be required in addition to the formal qualification.

Tasks Include:
- performing scientific tests, experiments and computations and collating and analysing the results
- inspecting animals, plants and agricultural produce to ensure compliance with government and industry standards
- developing, operating and maintaining instruments and equipment used in clinical diagnosis and experimental research
- preparing, interpreting, inspecting and revising drawings, plans, diagrams, designs, maps and charts
- installing, testing, repairing and modifying electrical, electronic and mechanical equipment
- estimating quantities and costs of materials
- providing technical support for telecommunications networks, and to users of computer hardware and software

Occupations in this sub-major group are classified into the following minor groups:
311 Agricultural, Medical and Science Technicians
312 Building and Engineering Technicians
313 ICT and Telecommunications Technicians

MINOR GROUP 311 AGRICULTURAL, MEDICAL AND SCIENCE TECHNICIANS

AGRICULTURAL, MEDICAL AND SCIENCE TECHNICIANS perform tests and experiments, and provide support to Professionals engaged in agriculture, medicine and science including inspecting animals, plants and agricultural produce.

Indicative Skill Level:
Most occupations in this minor group have a level of skill commensurate with the qualifications and experience outlined below.

In Australia:
AQF Associate Degree, Advanced Diploma or Diploma (ANZSCO Skill Level 2)

In New Zealand:
NZ Register Diploma (ANZSCO Skill Level 2)

At least three years of relevant experience may substitute for the formal qualifications listed above. In some instances relevant experience and/or on-the-job training may be required in addition to the formal qualification.

Tasks Include:
- collecting information and samples and conducting field and laboratory experiments, tests and analyses
- presenting results in graphic and written form by preparing maps, charts, sketches, diagrams and reports
- inspecting animals, plants, agricultural produce and facilities to ensure compliance with government and industry standards with respect to quality, health and licensing
- testing samples of produce for quality, size and purity
- undertaking and assisting in medical analytical procedures, and assisting Anaesthetists, surgical teams and Pharmacists
- checking, calibrating and maintaining test equipment

Occupations in this minor group are classified into the following unit groups:
3111 Agricultural Technicians
3112 Medical Technicians
3113 Primary Products Inspectors
3114 Science Technicians

UNIT GROUP 3111 AGRICULTURAL TECHNICIANS

AGRICULTURAL TECHNICIANS perform tests and experiments, and provide technical support to assist Agricultural Scientists in areas such as research, production, servicing and marketing.

Indicative Skill Level:
Most occupations in this unit group have a level of skill commensurate with the qualifications and experience outlined below.

In Australia:
AQF Associate Degree, Advanced Diploma or Diploma (ANZSCO Skill Level 2)

In New Zealand:
NZ Register Diploma (ANZSCO Skill Level 2)

At least three years of relevant experience may substitute for the formal qualifications listed above. In some instances relevant experience and/or on-the-job training may be required in addition to the formal qualification.

Tasks Include:
- examining topographical, physical and soil characteristics of farmland to determine its most effective use and identify nutrient deficiencies
- assisting in developing new methods of planting, fertilising, harvesting and processing crops to achieve optimum land usage
- identifying pathogenic micro-organisms and insects, parasites, fungi and weeds harmful to crops and livestock, and assisting in devising methods of control
- analysing produce to set and maintain standards of quality
- inspecting livestock to gauge the effectiveness of feed formulae
- assisting in controlled breeding experiments to develop improved crop and livestock strains
- arranging the supply of drugs, vaccines and other chemicals to Farmers and Farm Managers, and giving advice on their use
- collecting and collating data for research
- planning slaughtering, harvesting and other aspects of production processes
- may advise producers on farming techniques and management

Occupation:
311111 Agricultural Technician

311111 AGRICULTURAL TECHNICIAN

Alternative Title:
Agricultural Technical Officer

Performs tests and experiments, and provides technical support to assist Agricultural Scientists in areas such as research, production, servicing and marketing.

Skill Level: 2

Specialisations:
Agriculture Laboratory Technician
Artificial Insemination Technical Officer
Dairy Technician
Field Crop Technical Officer
Herd Tester
Horticultural Technical Officer
Poultry Technical Officer

UNIT GROUP 3112 MEDICAL TECHNICIANS

MEDICAL TECHNICIANS operate anaesthetic, cardiac, operating theatre and medical testing equipment, perform and assist with laboratory tests, and fill prescriptions in support of Health Professionals.

Indicative Skill Level:
Most occupations in this unit group have a level of skill commensurate with the qualifications and experience outlined below.

In Australia:
AQF Associate Degree, Advanced Diploma or Diploma (ANZSCO Skill Level 2)

In New Zealand:
NZ Register Diploma (ANZSCO Skill Level 2)

At least three years of relevant experience may substitute for the formal qualifications listed above. In some instances relevant experience and/or on-the-job training may be required in addition to the formal qualification.

Registration or licensing may be required.

Tasks Include:

- operating equipment used in diagnosing and monitoring disorders of the heart, kidneys, nervous system and hearing, and in anaesthesia
- undertaking and assisting with medical analytical procedures and assisting Anaesthetists and surgical teams
- recording the electrical activity of the heart, from which the heart rate is measured and pattern and rhythm interpreted
- preparing and staining slides and tissue sections to study the cells of blood and for histological examination
- performing diagnostic tests on tissues and body fluids and analysing the chemical constituents of blood, urine, faeces and tissues
- testing for diseases by looking for the presence of antibodies and the products of immune response in samples
- setting up, checking and maintaining operating theatres, anaesthetic workstations, life support machines and associated equipment
- referring prescriptions to Pharmacists and assisting in preparing medications

Occupations:
311211 Anaesthetic Technician
311212 Cardiac Technician
311213 Medical Laboratory Technician
311214 Operating Theatre Technician
311215 Pharmacy Technician
311299 Medical Technicians nec

311211 ANAESTHETIC TECHNICIAN

Prepares and maintains anaesthetic equipment for operating theatres or clinics, and assists Anaesthetists during anaesthetic procedures.

Skill Level: 2

UNIT GROUP 3112 MEDICAL TECHNICIANS *continued*

311212 CARDIAC TECHNICIAN

Conducts tests on patients to record heart activity using specialised equipment, recording devices and laboratory instruments in support of Cardiologists and other Medical Practitioners engaged in diagnosing, monitoring and treating heart disease.

Skill Level: 2

Specialisations:
- Cardiac Technologist
- Electrocardiographic Technician

311213 MEDICAL LABORATORY TECHNICIAN

Alternative Title:
- Medical Laboratory Technical Officer

Performs routine medical laboratory tests and operates diagnostic laboratory equipment under the supervision of Medical Laboratory Scientists and Pathologists. Registration or licensing may be required.

Skill Level: 2

311214 OPERATING THEATRE TECHNICIAN

Prepares and maintains an operating theatre and its equipment, assists the surgical team during operations and provides support to patients in the recovery room.

Skill Level: 2

311215 PHARMACY TECHNICIAN

Alternative Title:
- Dispensary Technician

Fills and labels patients' prescriptions under the supervision of a Pharmacist. May record details of, place orders for, take stock of, and store medications and medical supplies and deliver them to patients.

Skill Level: 2

311299 MEDICAL TECHNICIANS NEC

This occupation group covers Medical Technicians not elsewhere classified.

Skill Level: 2

Occupations in this group include:
- Audiometrist
- Dialysis Technician
- Electroencephalographic Technician
- Mortuary Technician
- Neurophysiological Technician
- Orthotic and Prosthetic Technician
- Perfusionist
- Renal Technician
- Sleep Technician

UNIT GROUP 3113 PRIMARY PRODUCTS INSPECTORS

PRIMARY PRODUCTS INSPECTORS inspect animals, plants and agricultural produce and facilities to ensure compliance with government and industry standards with respect to quality, health and licensing.

Indicative Skill Level:
Most occupations in this unit group have a level of skill commensurate with the qualifications and experience outlined below.

In Australia:
AQF Associate Degree, Advanced Diploma or Diploma (ANZSCO Skill Level 2)

In New Zealand:
NZ Register Diploma (ANZSCO Skill Level 2)

At least three years of relevant experience may substitute for the formal qualifications listed above. In some instances relevant experience and/or on-the-job training may be required in addition to the formal qualification.

Tasks Include:

- inspecting animals, plants and agricultural produce to identify product quality issues, and providing advice to producers
- auditing and monitoring quality procedures at farms and food handling and processing facilities to ensure compliance with required standards
- testing samples of produce for quality, size and purity
- ensuring that required standards of hygiene are observed at storage, processing and packing facilities and in transport vehicles
- advising primary producers on economic aspects of disease eradication and informing producers and the general public of the health implications of diseases and impurities
- advising on the identification of pests and diseases and on regulations pertaining to grading, packing and loading of products
- examining imported plants and animals, and products, such as timber, seeds and dried fruits, and making quarantine arrangements
- patrolling and investigating waterways for unlawful fishing activities and the removal of protected marine life
- educating, advising and providing information on a wide range of topics relating to fish and their protection
- may initiate or assist in legal action to enforce regulations

Occupations:
311311 Fisheries Officer
311312 Meat Inspector
311313 Quarantine Officer
311399 Primary Products Inspectors nec

311311 FISHERIES OFFICER

Alternative Title:
Fisheries Inspector

Inspects fishing vessels, gear, licences and catches to ensure that fisheries laws and regulations are obeyed.

Skill Level: 2

UNIT GROUP 3113 PRIMARY PRODUCTS INSPECTORS *continued*

311312 MEAT INSPECTOR

Inspects animal carcasses, internal organs and meat processing facilities for disease to ensure compliance with government and industry standards with respect to quality and health.

Skill Level: 2

311313 QUARANTINE OFFICER

Alternative Titles:

- Biosecurity Officer (Ministry of Agriculture and Forestry) (NZ)
- Quarantine Inspector

Inspects incoming animals, plants, and animal and plant products to ensure compliance with laws and regulations to prevent the spread of exotic pests and diseases.

Skill Level: 2

311399 PRIMARY PRODUCTS INSPECTORS NEC

This occupation group covers Primary Products Inspectors not elsewhere classified.

Skill Level: 2

Occupations in this group include:

- Dairy Quality Assurance Officer
- Fruit and Vegetable Inspector

UNIT GROUP 3114 SCIENCE TECHNICIANS

SCIENCE TECHNICIANS perform tests and experiments, and provide technical support functions to assist with research, design, production and teaching in chemistry, earth sciences, life sciences, and physical sciences.

Indicative Skill Level:
Most occupations in this unit group have a level of skill commensurate with the qualifications and experience outlined below.

In Australia:
AQF Associate Degree, Advanced Diploma or Diploma (ANZSCO Skill Level 2)

In New Zealand:
NZ Register Diploma (ANZSCO Skill Level 2)

At least three years of relevant experience may substitute for the formal qualifications listed above. In some instances relevant experience and/or on-the-job training may be required in addition to the formal qualification.

Tasks Include:
- preparing materials for experimentation such as freezing and slicing specimens and mixing chemicals
- collecting information and samples
- conducting field and laboratory experiments, tests and analyses
- presenting results in graphic and written form by preparing maps, charts, sketches, diagrams and reports
- performing routine mathematical calculations, and computations of measurements
- controlling the quality and quantity of laboratory supplies by testing samples and monitoring usage
- checking, calibrating and maintaining test equipment
- participating in fabricating, installing and modifying equipment to ensure that critical standards are met
- preparing experiments and demonstrations for science classes

Occupations:
311411 Chemistry Technician
311412 Earth Science Technician
311413 Life Science Technician
311414 School Laboratory Technician
311499 Science Technicians nec

311411 CHEMISTRY TECHNICIAN

Alternative Title:
Chemistry Technical Officer

Performs laboratory tests on organic and inorganic chemicals, analyses test data and carries out technical functions in support of Chemists or Chemical Engineers in a wide variety of areas such as fuels, agricultural products, food, pharmaceuticals, paints, metals, plastics, textiles, detergents, paper, fertilisers and cosmetics.

Skill Level: 2

Specialisations:
Chemical Instrumentation Officer
Chemical Process Analyst
Chemistry Laboratory Technician
Dairy Laboratory Technician
Petroleum Laboratory Technician
Sugar Laboratory Assistant

UNIT GROUP 3114 SCIENCE TECHNICIANS *continued*

311412 EARTH SCIENCE TECHNICIAN

Alternative Title:

Earth Science Technical Officer

Collects and tests earth and water samples, records observations and analyses data in support of Geologists or Geophysicists.

Skill Level: 2

Specialisations:

Earth Science Laboratory Technician
Geochemical Laboratory Technician
Geological Technical Officer
Geoscience Laboratory Technician
Hydrographer
Hydrographical Technical Officer
Hydrological Technical Officer
Meteorological Observer
Seismology Technical Officer
Soil Science Technical Officer
Water Resources Technical Officer

311413 LIFE SCIENCE TECHNICIAN

Alternative Title:

Life Science Technical Officer

Identifies and collects living organisms and conducts field and laboratory studies in support of Life Scientists or Environmental Scientists.

Skill Level: 2

Specialisations:

Biological Technical Officer
Botanical Technical Officer
Ecological Technical Officer
Environmental Technical Officer
Fisheries Technical Officer
Forestry Technical Officer
Wood Technologist
Zoology Technical Officer

311414 SCHOOL LABORATORY TECHNICIAN

Prepares experiments and demonstrations, makes up solutions, prepares slides, orders books and equipment, and tidies up laboratories in support of teaching chemistry, earth sciences, life sciences and physical sciences.

Skill Level: 2

UNIT GROUP 3114 SCIENCE TECHNICIANS *continued*

311499 SCIENCE TECHNICIANS NEC

This occupation group covers Science Technicians not elsewhere classified.

Skill Level: 2

Occupations in this group include:

- Fibre Technologist
- Optics Technical Officer
- Physics Technical Officer
- Textile Technical Officer

MINOR GROUP 312 BUILDING AND ENGINEERING TECHNICIANS

BUILDING AND ENGINEERING TECHNICIANS perform tests and provide technical support to Construction Managers, Architects and Engineering Professionals in research, design, construction, operation and maintenance of equipment, distribution systems and installations, and resource estimation and site inspection.

Indicative Skill Level:
Most occupations in this minor group have a level of skill commensurate with the qualifications and experience outlined below.

In Australia:
AQF Associate Degree, Advanced Diploma or Diploma (ANZSCO Skill Level 2)

In New Zealand:
NZ Register Diploma (ANZSCO Skill Level 2)

At least three years of relevant experience may substitute for the formal qualifications listed above. In some instances relevant experience and/or on-the-job training may be required in addition to the formal qualification.

Tasks Include:
- preparing, interpreting, inspecting and revising drawings, plans, diagrams, designs, maps and charts
- performing complex computations and field and laboratory tests, and recording the results
- installing, testing, repairing and modifying electrical, electronic and mechanical equipment
- estimating quantities and costs of materials
- inspecting buildings, plumbing work, machines, equipment, working conditions and public places to ensure compliance with relevant laws, standards and regulations
- planning, scheduling, coordinating and monitoring maintenance of plant equipment
- testing materials
- overseeing the safety of mining operations and supervising Miners

Occupations in this minor group are classified into the following unit groups:
3121 Architectural, Building and Surveying Technicians
3122 Civil Engineering Draftspersons and Technicians
3123 Electrical Engineering Draftspersons and Technicians
3124 Electronic Engineering Draftspersons and Technicians
3125 Mechanical Engineering Draftspersons and Technicians
3126 Safety Inspectors
3129 Other Building and Engineering Technicians

UNIT GROUP 3121 ARCHITECTURAL, BUILDING AND SURVEYING TECHNICIANS

ARCHITECTURAL, BUILDING AND SURVEYING TECHNICIANS perform technical functions to assist Construction Managers, Architects and Surveyors by supervising and inspecting construction sites, estimating time, costs and resources, inspecting plumbing work, and collecting and evaluating survey data and preparing maps and plans.

Indicative Skill Level:
Most occupations in this unit group have a level of skill commensurate with the qualifications and experience outlined below.

In Australia:
AQF Associate Degree, Advanced Diploma or Diploma (ANZSCO Skill Level 2)

In New Zealand:
NZ Register Diploma (ANZSCO Skill Level 2)

At least three years of relevant experience may substitute for the formal qualifications listed above. In some instances relevant experience and/or on-the-job training may be required in addition to the formal qualification.

Registration or licensing may be required.

Tasks Include:
- assisting Construction Managers, Architects and Surveyors in planning and organisation
- interpreting plans, regulations and codes of practice
- preparing preliminary sketches, working drawings and specifications
- preparing, editing and revising plans, maps, charts and drawings
- coordinating works programs
- inspecting work and materials for compliance with specifications, regulations and standards
- calculating costs and estimating time scales
- collecting data using surveying instruments and photogrammetric equipment
- performing routine computations and plotting preliminary data

Occupations:
312111 Architectural Draftsperson
312112 Building Associate
312113 Building Inspector
312114 Construction Estimator
312115 Plumbing Inspector
312116 Surveying and Cartographic Technician

312111 ARCHITECTURAL DRAFTSPERSON

Alternative Title:
Architectural Associate

Completes Architects' concepts by preparing drawings and plans, and liaising with builders and contractors.

Skill Level: 2

Specialisation:
Building Drafting Officer

UNIT GROUP 3121 ARCHITECTURAL, BUILDING AND SURVEYING TECHNICIANS *continued*

312112 BUILDING ASSOCIATE

Supervises construction sites, and organises and coordinates the material and human resources required. Registration or licensing may be required.

Skill Level: 2

Specialisations:
- Building Construction Supervisor
- Clerk of Works

312113 BUILDING INSPECTOR

Alternative Titles:
- Building Certifier
- Building Surveyor

Inspects buildings to ensure compliance with laws and regulations and advises on building requirements. Registration or licensing may be required.

Skill Level: 2

Specialisation:
- Electrical Installation Inspector

312114 CONSTRUCTION ESTIMATOR

Alternative Title:
- Building Estimator

Prepares and delivers estimates and cost plans for construction projects up to the tender settlement stage.

Skill Level: 2

312115 PLUMBING INSPECTOR

Inspects plumbing work to ensure compliance with relevant standards and regulations. Registration or licensing is required.

Skill Level: 2

Specialisations:
- Drainage Inspector
- Gas Plumbing Inspector
- Sanitary Plumbing and Water Supply Inspector

UNIT GROUP 3121 ARCHITECTURAL, BUILDING AND SURVEYING TECHNICIANS *continued*

312116 SURVEYING OR CARTOGRAPHIC TECHNICIAN

Alternative Title:

Surveying Technologist

Collects, records and evaluates survey data and prepares maps, charts and plans in support of Surveyors or Cartographers. Registration or licensing may be required.

Skill Level: 2

Specialisations:

- Aerial Survey Technician
- Engineering Survey Drafting Technician
- Photogrammetrist
- Topographic Drafting Officer

UNIT GROUP 3122 CIVIL ENGINEERING DRAFTSPERSONS AND TECHNICIANS

CIVIL ENGINEERING DRAFTSPERSONS AND TECHNICIANS assist in civil engineering research, design, construction, operations and maintenance.

Indicative Skill Level:
Most occupations in this unit group have a level of skill commensurate with the qualifications and experience outlined below.

In Australia:
AQF Associate Degree, Advanced Diploma or Diploma (ANZSCO Skill Level 2)

In New Zealand:
NZ Register Diploma (ANZSCO Skill Level 2)

At least three years of relevant experience may substitute for the formal qualifications listed above. In some instances relevant experience and/or on-the-job training may be required in addition to the formal qualification.

Registration or licensing may be required.

Tasks Include:
- preparing sketches, charts, tabulations, plans and designs for civil engineering works such as drainage, water supply, sewerage reticulation systems, roads, airports, dams, bridges and other structures
- performing and directing fieldwork and laboratory testing
- interpreting work assignment instructions, applying appropriate procedures and selecting equipment
- collecting and analysing data, and carrying out computations
- estimating material costs and ensuring finished works are within specifications, regulations and contract provisions
- inspecting civil engineering works, and organising and supervising maintenance and repair work
- conducting field and laboratory tests of construction materials and soils, and collecting data for traffic surveys

Occupations:
312211 Civil Engineering Draftsperson
312212 Civil Engineering Technician

312211 CIVIL ENGINEERING DRAFTSPERSON

Prepares detailed drawings and plans for civil engineering work in support of Civil Engineering Professionals and Engineering Technologists. Registration or licensing may be required.

Skill Level: 2

Specialisations:
Civil Engineering Design Draftsperson
Plumbing Engineering Draftsperson
Road Design Draftsperson
Sewage Reticulation Drafting Officer
Structural Engineering Drafting Officer

UNIT GROUP 3122 CIVIL ENGINEERING DRAFTSPERSONS AND TECHNICIANS *continued*

312212 CIVIL ENGINEERING TECHNICIAN

Conducts tests of construction materials, prepares sketches and tabulations, and assists in estimating costs in support of Civil Engineering Professionals and Engineering Technologists. Registration or licensing may be required.

Skill Level: 2

Specialisations:
- Civil Engineering Assistant
- Civil Laboratory Technician
- Geotechnical Laboratory Technician

UNIT GROUP 3123 ELECTRICAL ENGINEERING DRAFTSPERSONS AND TECHNICIANS

ELECTRICAL ENGINEERING DRAFTSPERSONS AND TECHNICIANS assist in electrical engineering research, design, manufacture, assembly, construction, operation and maintenance of equipment, facilities and distribution systems.

Indicative Skill Level:
Most occupations in this unit group have a level of skill commensurate with the qualifications and experience outlined below.

In Australia:
AQF Associate Degree, Advanced Diploma or Diploma (ANZSCO Skill Level 2)

In New Zealand:
NZ Register Diploma (ANZSCO Skill Level 2)

At least three years of relevant experience may substitute for the formal qualifications listed above. In some instances relevant experience and/or on-the-job training may be required in addition to the formal qualification.

Registration or licensing may be required.

Tasks Include:
- preparing drawings, plans and diagrams of electrical installations and circuitry
- assisting Electrical Engineers and Engineering Technologists in design and layout of electrical installations and circuitry on substations, switchgear, cabling systems and motor control systems
- collecting data, performing tests and complex calculations, graphing results, and preparing charts and tabulations
- estimating materials costs and quantities
- inspecting designs and finished products for compliance with specifications and regulations
- assembling, installing, testing, calibrating, modifying and repairing electrical equipment and installations to conform with regulations and safety requirements
- undertaking electrical workshop functions such as installing assemblies for protection relays, metering and indicating devices
- assisting with research and experimentation programs

Occupations:
312311 Electrical Engineering Draftsperson
312312 Electrical Engineering Technician

312311 ELECTRICAL ENGINEERING DRAFTSPERSON

Prepares detailed drawings and plans of electrical installations and circuitry in support of Electrical Engineers and Engineering Technologists. Registration or licensing may be required.

Skill Level: 2

Specialisations:
Electrical Engineering Design Draftsperson
Electrical Engineering Drafting Officer
Relays Draftsperson
Substation Design Draftsperson

UNIT GROUP 3123 ELECTRICAL ENGINEERING DRAFTSPERSONS AND TECHNICIANS *continued*

312312 ELECTRICAL ENGINEERING TECHNICIAN

Alternative Title:

Electrical Engineering Technical Officer

Conducts tests of electrical systems, prepares charts and tabulations, and assists in estimating costs in support of Electrical Engineers and Engineering Technologists. Registration or licensing may be required.

Skill Level: 2

Specialisations:

Electrical Engineering Detail Draftsperson
Electrical Engineering Laboratory Technician
Electrical Instrument Technician

UNIT GROUP 3124 ELECTRONIC ENGINEERING DRAFTSPERSONS AND TECHNICIANS

ELECTRONIC ENGINEERING DRAFTSPERSONS AND TECHNICIANS assist in electronic engineering research, design, manufacture, assembly, construction, operation and maintenance of equipment, facilities and distribution systems.

Indicative Skill Level:
Most occupations in this unit group have a level of skill commensurate with the qualifications and experience outlined below.

In Australia:
AQF Associate Degree, Advanced Diploma or Diploma (ANZSCO Skill Level 2)

In New Zealand:
NZ Register Diploma (ANZSCO Skill Level 2)

At least three years of relevant experience may substitute for the formal qualifications listed above. In some instances relevant experience and/or on-the-job training may be required in addition to the formal qualification.

Registration or licensing may be required.

Tasks Include:
- preparing drawings, plans and diagrams for electronic engineering work
- developing, constructing and testing electronic equipment and associated circuitry in accordance with technical manuals and instructions of Electronics Engineers and Engineering Technologists
- performing tests, graphing results, preparing charts and tabulations
- estimating material costs and quantities
- evaluating performance of equipment
- inspecting designs and finished products for compliance with specifications, drawings, contracts and regulations
- installing, testing, repairing and modifying electronic equipment

Occupations:
312411 Electronic Engineering Draftsperson
312412 Electronic Engineering Technician

312411 ELECTRONIC ENGINEERING DRAFTSPERSON

Prepares detailed drawings and plans of electronic engineering work in support of Electronics Engineers and Engineering Technologists. Registration or licensing may be required.

Skill Level: 2

Specialisations:
Communications and Data Systems Drafting Officer
Control Systems Drafting Officer
Electronics Detail Draftsperson

UNIT GROUP 3124 ELECTRONIC ENGINEERING DRAFTSPERSONS AND TECHNICIANS *continued*

312412 ELECTRONIC ENGINEERING TECHNICIAN

Conducts tests of electronic systems, collects and analyses data, and assembles circuitry in support of Electronics Engineers and Engineering Technologists. Registration or licensing may be required.

Skill Level: 2

Specialisations:

- Aircraft Electronics Technical Officer
- Communications Engineering Technical Officer
- Communications Engineering Technician
- Computer Numeric Control Technical Officer
- Digital Controls Technical Officer
- Flight Surveyor
- Printed Circuit Board Designer
- Process Control Technician
- Telemetry Technician

UNIT GROUP 3125 MECHANICAL ENGINEERING DRAFTSPERSONS AND TECHNICIANS

MECHANICAL ENGINEERING DRAFTSPERSONS AND TECHNICIANS assist in mechanical engineering research, design, manufacture, construction, operation and maintenance of machines, manufacturing equipment, mechanical installations and facilities.

Indicative Skill Level:
Most occupations in this unit group have a level of skill commensurate with the qualifications and experience outlined below.

In Australia:
AQF Associate Degree, Advanced Diploma or Diploma (ANZSCO Skill Level 2)

In New Zealand:
NZ Register Diploma (ANZSCO Skill Level 2)

At least three years of relevant experience may substitute for the formal qualifications listed above. In some instances relevant experience and/or on-the-job training may be required in addition to the formal qualification.

Tasks Include:
- preparing drawings, plans and designs for mechanical engineering work under the direction of Mechanical Engineers and Engineering Technologists
- assisting Mechanical Engineers and Engineering Technologists in the design of mechanical equipment and plant
- selecting tools and equipment
- assembling and installing new and modified mechanical assemblies, components, machine tools and controls, and hydraulic power systems
- estimating material costs and quantities, and machine requirements
- performing and directing field and laboratory tests
- collecting and analysing data, carrying out complex computations and preparing diagrams
- organising and supervising inspection and maintenance of machines and plant
- ensuring that designs and finished work are within specifications, regulations and contract provisions

Occupations:
312511 Mechanical Engineering Draftsperson
312512 Mechanical Engineering Technician

312511 MECHANICAL ENGINEERING DRAFTSPERSON

Prepares detailed drawings and plans of mechanical engineering work in support of Mechanical Engineers and Engineering Technologists.

Skill Level: 2

Specialisations:
Airconditioning Drafting Officer
Heating and Ventilating Technical Officer
Tool Design Draftsperson
Tool Designer

UNIT GROUP 3125 MECHANICAL ENGINEERING DRAFTSPERSONS AND TECHNICIANS *continued*

312512 MECHANICAL ENGINEERING TECHNICIAN

Conducts tests of mechanical systems, collects and analyses data, and assembles and installs mechanical assemblies in support of Mechanical Engineers and Engineering Technologists.

Skill Level: 2

Specialisations:

- Boiler Testing Technician
- Hydraulic Controls Technician
- Mechanical Laboratory Technician
- Pipe Testing Technician

UNIT GROUP 3126 SAFETY INSPECTORS

SAFETY INSPECTORS inspect machines, equipment, working conditions and public places to ensure compliance with government and industry standards and regulations, in relation to occupational health and safety.

Indicative Skill Level:
Most occupations in this unit group have a level of skill commensurate with the qualifications and experience outlined below.

In Australia:
AQF Associate Degree, Advanced Diploma or Diploma (ANZSCO Skill Level 2)

In New Zealand:
NZ Register Diploma (ANZSCO Skill Level 2)

At least three years of relevant experience may substitute for the formal qualifications listed above. In some instances relevant experience and/or on-the-job training may be required in addition to the formal qualification.

Registration or licensing may be required.

Tasks Include:
- examining equipment specifications, and inspecting and testing machines, equipment and clothing to ensure compliance with safety standards and serviceability
- inspecting factories and other work sites to ensure compliance with government and industry standards and regulations
- observing workers to ensure protective devices are being utilised according to regulations and that combustible and other hazardous materials are used and stored in accordance with approved procedures
- conducting tests in work areas to detect toxic fumes, explosive gas-air mixtures and other work hazards
- ensuring fire prevention equipment and other safety supplies, such as first aid kits, stretchers and blankets, conform to standards
- assisting in conducting safety meetings and campaigns, and organising training in general safety principles in keeping with regulations
- advising organisations on ways to comply with occupational health and safety legislative requirements
- investigating incidents and fatalities, to determine causes and to collect evidence of non-compliance with occupational health and safety legislation

Occupation:
312611 Safety Inspector

312611 SAFETY INSPECTOR

Inspects machines, equipment, working conditions and public places to ensure compliance with government and industry standards and regulations, in relation to occupational health and safety. Registration or licensing may be required.

Skill Level: 2

Specialisations:
Boilers and Pressure Vessels Inspector
Gas Examiner
Lifts and Cranes Inspector
Mines Inspector
Occupational Health and Safety Inspector

UNIT GROUP 3129 OTHER BUILDING AND ENGINEERING TECHNICIANS

This unit group covers Building and Engineering Technicians not elsewhere classified.

It includes Maintenance Planners, Metallurgical or Materials Technicians, and Mine Deputies.

Indicative Skill Level:
Most occupations in this unit group have a level of skill commensurate with the qualifications and experience outlined below.

In Australia:
AQF Associate Degree, Advanced Diploma or Diploma (ANZSCO Skill Level 2)

In New Zealand:
NZ Register Diploma (ANZSCO Skill Level 2)

At least three years of relevant experience may substitute for the formal qualifications listed above. In some instances relevant experience and/or on-the-job training may be required in addition to the formal qualification.

Registration or licensing may be required.

Occupations:
312911 Maintenance Planner
312912 Metallurgical or Materials Technician
312913 Mine Deputy
312999 Building and Engineering Technicians nec

312911 MAINTENANCE PLANNER

Alternative Titles:
Maintenance Scheduler
Shutdown Coordinator
Shutdown Planner

Develops maintenance planning strategies, and schedules, coordinates and monitors the maintenance of all plant equipment.

Skill Level: 2

UNIT GROUP 3129 OTHER BUILDING AND ENGINEERING TECHNICIANS *continued*

312912 METALLURGICAL OR MATERIALS TECHNICIAN

Tests materials as part of mineral and metal processing and refining, or for research into metals, ceramics, polymers and other materials in support of Metallurgists and Materials Engineers. Registration or licensing may be required.

Skill Level: 2

Specialisations:
- Dye Penetrant Testing Technician
- Heat Treatment Technician
- Magnetic Testing Technician
- Metallurgy Laboratory Technician
- Non-destructive Testing Technician
- Petroleum Products Laboratory Technician
- Petroleum Refinery Laboratory Technician
- Pressure Testing Technician
- Ultrasound Technician

312913 MINE DEPUTY

Oversees the safety of mining operations and supervises Miners. Registration or licensing is required.

Skill Level: 2

Specialisations:
- Mining Technician
- Open Cut Examiner

312999 BUILDING AND ENGINEERING TECHNICIANS NEC

This occupation group covers Building and Engineering Technicians not elsewhere classified. Registration or licensing may be required.

Skill Level: 2

Occupations in this group include:
- Aircraft Detail Draftsperson
- Aircraft Systems Technician (Air Force)
- Airframe Technical Officer
- Avionics Systems Technician (Air Force)
- Biomedical Engineering Associate
- Mining Detail Draftsperson
- Shipbuilding Draftsperson

MINOR GROUP 313 ICT AND TELECOMMUNICATIONS TECHNICIANS

ICT AND TELECOMMUNICATIONS TECHNICIANS provide support to the development and maintenance of computer infrastructure, web technology and telecommunications networks, and the diagnosis and resolution of technical problems.

Indicative Skill Level:
Most occupations in this minor group have a level of skill commensurate with the qualifications and experience outlined below.

In Australia:
AQF Associate Degree, Advanced Diploma or Diploma (ANZSCO Skill Level 2)

In New Zealand:
NZ Register Diploma (ANZSCO Skill Level 2)

At least three years of relevant experience may substitute for the formal qualifications listed above. In some instances relevant experience and/or on-the-job training may be required in addition to the formal qualification.

Tasks Include:
- determining software and hardware requirements to provide solutions for problems
- responding to inquiries about software and hardware problems
- repairing and replacing peripheral equipment such as terminals, printers and modems
- installing and downloading appropriate software, and adapting existing programs to meet users' requirements
- implementing computer networks, and ensuring efficient use of applications and equipment
- designing and maintaining web sites
- installing and maintaining microwave, telemetry, multiplexing, satellite and other radio and electromagnetic wave communication systems
- configuring and integrating network and telecommunications technology with computer software, hardware, peripherals and operating systems
- planning the development of customer access telecommunications network infrastructure

Occupations in this minor group are classified into the following unit groups:
3131 ICT Support Technicians
3132 Telecommunications Technical Specialists

UNIT GROUP 3131 ICT SUPPORT TECHNICIANS

ICT SUPPORT TECHNICIANS provide support for the deployment and maintenance of computer infrastructure and web technology and the diagnosis and resolution of technical problems.

Indicative Skill Level:
Most occupations in this unit group have a level of skill commensurate with the qualifications and experience outlined below.

In Australia:
AQF Associate Degree, Advanced Diploma or Diploma (ANZSCO Skill Level 2)

In New Zealand:
NZ Register Diploma (ANZSCO Skill Level 2)

At least three years of relevant experience may substitute for the formal qualifications listed above. In some instances relevant experience and/or relevant vendor certification may be required in addition to the formal qualification.

Tasks Include:
- determining software and hardware requirements to provide solutions to problems
- responding to inquiries about software and hardware problems
- adapting existing programs to meet users' requirements
- installing and downloading appropriate software
- ensuring efficient use of applications and equipment
- implementing computer networks
- designing and maintaining web sites
- repairing and replacing peripheral equipment such as terminals, printers and modems
- may work in a call centre

Occupations:
313111 Hardware Technician
313112 ICT Customer Support Officer
313113 Web Administrator
313199 ICT Support Technicians nec

313111 HARDWARE TECHNICIAN

Supports and maintains computer systems and peripherals by installing, configuring, testing, troubleshooting, and repairing hardware.

Skill Level: 2

UNIT GROUP 3131 ICT SUPPORT TECHNICIANS *continued*

313112 ICT CUSTOMER SUPPORT OFFICER

Alternative Titles:
- ICT Help Desk Officer
- ICT Help Desk Technician
- Systems Support Officer

Provides support, education and guidance in the deployment and maintenance of computer infrastructure and the diagnosis and resolution of technical problems and issues. May work in a call centre.

Skill Level: 2

Specialisations:
- Network Support Technician
- Operator Command Support Systems (Army)

313113 WEB ADMINISTRATOR

Alternative Title:
- Web Master

Designs, builds and maintains web sites, and provides web technology solutions and services.

Skill Level: 2

313199 ICT SUPPORT TECHNICIANS NEC

This occupation group covers ICT Support Technicians not elsewhere classified.

Skill Level: 2

Occupations in this group include:
- Applications Packager
- Computer Systems Technician
- Telecommunications Computer Systems Technician

UNIT GROUP 3132 TELECOMMUNICATIONS TECHNICAL SPECIALISTS

TELECOMMUNICATIONS TECHNICAL SPECIALISTS develop, monitor and carry out technical support functions for telecommunications networks and install computer equipment, computer systems and microwave, telemetry, multiplexing, satellite and other radio and electromagnetic wave communication systems.

Indicative Skill Level:
Most occupations in this unit group have a level of skill commensurate with the qualifications and experience outlined below.

In Australia:
AQF Associate Degree, Advanced Diploma or Diploma (ANZSCO Skill Level 2)

In New Zealand:
NZ Register Diploma (ANZSCO Skill Level 2)

At least three years of relevant experience and/or relevant vendor certification may substitute for the formal qualifications listed above. In some instances relevant experience and/or on-the-job training may be required in addition to the formal qualification.

Tasks Include:
- installing, maintaining, repairing and diagnosing malfunctions of microwave, telemetry, multiplexing, satellite and other radio and electromagnetic wave communication systems
- configuring and integrating network and telecommunications technology with computer software, hardware, desktops, peripherals, databases and operating systems
- developing and recording logs of the details, locations and status of inventories, parts, equipment and instruments and maintaining the documentation of communication policies, procedures, guidelines and regulations, and quality standards
- providing technical advice and information, and monitoring the performance of complex telecommunications networks and equipment
- planning the development of customer access telecommunications network infrastructure
- liaising with vendors, suppliers, service providers and external resources and monitoring contractual obligations and performance delivery
- providing ongoing operational support in designing, optimising, troubleshooting, diagnosing, repairing and resolving of telecommunications network performance malfunctions, defects and faults

Occupations:
313211 Radiocommunications Technician
313212 Telecommunications Field Engineer
313213 Telecommunications Network Planner
313214 Telecommunications Technical Officer or Technologist

313211 RADIOCOMMUNICATIONS TECHNICIAN

Installs, maintains, repairs and diagnoses malfunctions of microwave, telemetry, multiplexing, satellite and other radio and electromagnetic wave communication systems.

Skill Level: 2

UNIT GROUP 3132 TELECOMMUNICATIONS TECHNICAL SPECIALISTS *continued*

313212 TELECOMMUNICATIONS FIELD ENGINEER

Plans, designs, commissions and monitors complex telecommunications networks and associated equipment, provides technical advice and information, and identifies complex problems and initiates action to resolve them.

Skill Level: 2

313213 TELECOMMUNICATIONS NETWORK PLANNER

Plans the development of customer access telecommunications network infrastructure.

Skill Level: 2

313214 TELECOMMUNICATIONS TECHNICAL OFFICER OR TECHNOLOGIST

Carries out specialised design and support functions in telecommunications engineering including optimisation and performance monitoring of telecommunications networks, diagnosis and repair of faults, and the selection and installation of equipment.

Skill Level: 2

SUB-MAJOR GROUP 32 AUTOMOTIVE AND ENGINEERING TRADES WORKERS

AUTOMOTIVE AND ENGINEERING TRADES WORKERS construct, repair and maintain motor vehicles and aircraft structures and systems, and cut, shape, cast, join and finish metal, metal parts, subassemblies and precision instruments.

Indicative Skill Level:
Most occupations in this sub-major group have a level of skill commensurate with the qualifications and experience outlined below.

In Australia:

AQF Certificate III including at least two years of on-the-job training, or AQF Certificate IV (ANZSCO Skill Level 3)

In New Zealand:

NZ Register Level 4 qualification (ANZSCO Skill Level 3)

At least three years of relevant experience may substitute for the formal qualifications listed above. In some instances relevant experience and/or on-the-job training may be required in addition to the formal qualification.

Tasks Include:
- diagnosing electrical and mechanical faults in motor vehicles and aircraft
- dismantling engines and electrical systems, and removing damaged panels and interior trim
- repairing and replacing worn and defective parts
- painting repaired vehicle surfaces
- constructing, modifying, painting and upholstering prototype and specialised vehicle bodies
- marking and cutting out metal stock
- shaping stock using hand and machine tools
- shaping metal in moulds
- joining stock and components by welding, soldering, brazing, riveting, bolting, screwing and gluing
- fitting and testing components, and applying protective and decorative finishes to metal products

Occupations in this sub-major group are classified into the following minor groups:

321 Automotive Electricians and Mechanics
322 Fabrication Engineering Trades Workers
323 Mechanical Engineering Trades Workers
324 Panelbeaters, and Vehicle Body Builders, Trimmers and Painters

MINOR GROUP 321 AUTOMOTIVE ELECTRICIANS AND MECHANICS

AUTOMOTIVE ELECTRICIANS AND MECHANICS repair and maintain automotive electrical systems and motor vehicle and other internal combustion engines.

Mechanical Engineering Trades Workers are excluded from this minor group. Mechanical Engineering Trades Workers are included in Minor Group 323 Mechanical Engineering Trades Workers.

Indicative Skill Level:
Most occupations in this minor group have a level of skill commensurate with the qualifications and experience outlined below.

In Australia:
AQF Certificate III including at least two years of on-the-job training, or AQF Certificate IV (ANZSCO Skill Level 3)

In New Zealand:
NZ Register Level 4 qualification (ANZSCO Skill Level 3)

At least three years of relevant experience may substitute for the formal qualifications listed above. In some instances relevant experience and/or on-the-job training may be required in addition to the formal qualification.

Tasks Include:
- diagnosing electrical and mechanical faults in motor vehicles and small engines
- dismantling engines and electrical systems
- repairing and replacing worn and defective parts
- installing electrical equipment and electronic components in motor vehicles
- testing and adjusting electrical and mechanical systems and parts after repair for proper performance
- performing scheduled maintenance on motor vehicles

Occupations in this minor group are classified into the following unit groups:
3211 Automotive Electricians
3212 Motor Mechanics

UNIT GROUP 3211 AUTOMOTIVE ELECTRICIANS

AUTOMOTIVE ELECTRICIANS install, maintain and repair electrical wiring and electronic components in motor vehicles.

Indicative Skill Level:
Most occupations in this unit group have a level of skill commensurate with the qualifications and experience outlined below.

In Australia:
AQF Certificate III including at least two years of on-the-job training, or AQF Certificate IV (ANZSCO Skill Level 3)

In New Zealand:
NZ Register Level 4 qualification (ANZSCO Skill Level 3)

At least three years of relevant experience may substitute for the formal qualifications listed above. In some instances relevant experience and/or on-the-job training may be required in addition to the formal qualification.

Registration or licensing may be required.

Tasks Include:
- using test equipment to locate electrical and electronic malfunctions
- dismantling and removing electrical and electronic assemblies and components
- installing electrical equipment and electronic components in motor vehicles
- connecting power-operated vehicle equipment and accessories to power supply
- adjusting engine control systems and timing
- testing and replacing defective alternators, generators, voltage regulators and starter motors
- repairing and replacing faulty ignition and electrical wiring
- replacing defective parts such as fuses, lamps and switches

Occupation:
321111 Automotive Electrician

321111 AUTOMOTIVE ELECTRICIAN

Alternative Title:
Automotive Electrical Fitter

Installs, maintains and repairs electrical wiring and electronic components in motor vehicles. Registration or licensing may be required.

Skill Level: 3

UNIT GROUP 3212 MOTOR MECHANICS

MOTOR MECHANICS repair, maintain and test motor vehicle and other internal combustion engines and related mechanical components.

Motor Vehicle Parts and Accessories Fitters are excluded from this unit group. Motor Vehicle Parts and Accessories Fitters are included in Unit Group 8994 Motor Vehicle Parts and Accessories Fitters.

Indicative Skill Level:
Most occupations in this unit group have a level of skill commensurate with the qualifications and experience outlined below.

In Australia:
AQF Certificate III including at least two years of on-the-job training, or AQF Certificate IV (ANZSCO Skill Level 3)

In New Zealand:
NZ Register Level 4 qualification (ANZSCO Skill Level 3)

At least three years of relevant experience may substitute for the formal qualifications listed above. In some instances relevant experience and/or on-the-job training may be required in addition to the formal qualification.

Registration or licensing may be required.

Tasks Include:
- detecting and diagnosing faults in engines and parts
- dismantling and removing engine assemblies, transmissions, steering mechanisms and other components, and checking parts
- repairing and replacing worn and defective parts and reassembling mechanical components, and referring to service manuals as needed
- performing scheduled maintenance services, such as oil changes, lubrications and engine tune-ups, to achieve smoother running of vehicles and ensure compliance with pollution regulations
- reassembling engines and parts after being repaired
- testing and adjusting mechanical parts after being repaired for proper performance
- diagnosing and testing parts with the assistance of computers
- may inspect vehicles and issue roadworthiness certificates or detail work required to achieve roadworthiness

Occupations:
321211 Motor Mechanic (General)
321212 Diesel Motor Mechanic
321213 Motorcycle Mechanic
321214 Small Engine Mechanic

UNIT GROUP 3212 MOTOR MECHANICS *continued*

321211 MOTOR MECHANIC (GENERAL)

Maintains, tests and repairs petrol engines and the mechanical parts of lightweight motor vehicles such as transmissions, suspension, steering and brakes. Registration or licensing may be required.

Skill Level: 3

Specialisations:
- Automatic Transmission Mechanic
- Automotive Airconditioning Mechanic
- Brake Mechanic
- Ground Support Equipment Fitter (Air Force)
- Marine Technician (Navy)
- Vehicle Mechanic (Army)

321212 DIESEL MOTOR MECHANIC

Maintains, tests and repairs diesel motors and the mechanical parts of trucks, buses and other heavy vehicles such as transmissions, suspension, steering and brakes. Registration or licensing may be required.

Skill Level: 3

321213 MOTORCYCLE MECHANIC

Maintains, tests and repairs the mechanical parts of motorcycles. Registration or licensing may be required.

Skill Level: 3

321214 SMALL ENGINE MECHANIC

Maintains, tests and repairs engines of chainsaws, lawn mowers, garden tractors and other equipment with small engines. Registration or licensing may be required.

Skill Level: 3

Specialisations:
- Chainsaw Mechanic
- Lawnmower Mechanic
- Outboard Motor Mechanic

MINOR GROUP 322 FABRICATION ENGINEERING TRADES WORKERS

FABRICATION ENGINEERING TRADES WORKERS cast, shape, cut, join and finish metal.

Panel Beaters and Vehicle Body Builders are excluded from this minor group. Panel Beaters and Vehicle Body Builders are included in Minor Group 324 Panelbeaters, and Vehicle Body Builders, Trimmers and Painters.

Indicative Skill Level:
Most occupations in this minor group have a level of skill commensurate with the qualifications and experience outlined below.

In Australia:
AQF Certificate III including at least two years of on-the-job training, or AQF Certificate IV (ANZSCO Skill Level 3)

In New Zealand:
NZ Register Level 4 qualification (ANZSCO Skill Level 3)

At least three years of relevant experience may substitute for the formal qualifications listed above. In some instances relevant experience and/or on-the-job training may be required in addition to the formal qualification.

Tasks Include:
- studying blueprints, drawings and specifications to determine requirements and material, and selecting metal stock
- moulding molten metal, and applying protective and decorative finishes to metal products
- drawing and marking out patterns
- heating metal stock and shaping it using hammers
- cutting stock and moulding components to shape using hand tools, cutting torches and machine tools
- joining components by welding, soldering, brazing, riveting, bolting, screwing and gluing
- finishing products by cleaning, filing, sanding and polishing, and applying protective finishes

Occupations in this minor group are classified into the following unit groups:
3221 Metal Casting, Forging and Finishing Trades Workers
3222 Sheetmetal Trades Workers
3223 Structural Steel and Welding Trades Workers

UNIT GROUP 3221 METAL CASTING, FORGING AND FINISHING TRADES WORKERS

METAL CASTING, FORGING AND FINISHING TRADES WORKERS fabricate mould patterns and form sand moulds and cores for the production of metal castings, heat and hammer metal into shape, and make, repair, coat and polish metal parts and articles.

Indicative Skill Level:
Most occupations in this unit group have a level of skill commensurate with the qualifications and experience outlined below.

In Australia:
AQF Certificate III including at least two years of on-the-job training, or AQF Certificate IV (ANZSCO Skill Level 3)

In New Zealand:
NZ Register Level 4 qualification (ANZSCO Skill Level 3)

At least three years of relevant experience may substitute for the formal qualifications listed above. In some instances relevant experience and/or on-the-job training may be required in addition to the formal qualification.

Tasks Include:
- selecting metal stock for job requirements
- heating metal in forges and furnaces and hammering, punching and cutting metal using hand tools and machine presses
- tempering and hardening finished articles by quenching in oil or water baths or by cooling gradually in air
- preparing electrolytic and silver solutions for electroforming, and applying solution to the objects to be coated
- setting and adjusting controls to regulate electric current and depositing of coating on objects
- preparing horses' hooves for shoeing, nailing horseshoes to hooves, and trimming hooves
- cutting, trimming, shaping and smoothing stock to form mould patterns
- filling boxes with sand and setting patterns in place, and pouring molten metal into moulds
- applying refractory paint and positioning cores in moulds
- finishing metal and articles by polishing and buffing and applying shellac, lacquer, paint and other finishes

Occupations:
322111 Blacksmith
322112 Electroplater
322113 Farrier
322114 Metal Casting Trades Worker
322115 Metal Polisher

322111 BLACKSMITH

Shapes bars, rods and blocks of metal by heating and hammering to produce or repair metal articles.

Skill Level: 3

Specialisations:
Hammer Smith
Spring Maker
Tool Smith

UNIT GROUP 3221 METAL CASTING, FORGING AND FINISHING TRADES WORKERS *continued*

322112 ELECTROPLATER

Controls plating processes and maintains solutions used to coat metal articles and other parts with non-ferrous metals.

Skill Level: 3

Specialisations:
- Anodiser
- Electroformer

322113 FARRIER

Inspects, trims and shapes horses' hooves, and forms, fits and nails horseshoes.

Skill Level: 3

322114 METAL CASTING TRADES WORKER

Forms sand moulds and cores for the production of metal castings.

Skill Level: 3

Specialisations:
- Coremaker
- Metal Moulder

322115 METAL POLISHER

Polishes metal to impart smooth, reflective and other finishes.

Skill Level: 3

UNIT GROUP 3222 SHEETMETAL TRADES WORKERS

SHEETMETAL TRADES WORKERS mark out, shape, form and join sheetmetal and other materials to make products and components.

Indicative Skill Level:
Most occupations in this unit group have a level of skill commensurate with the qualifications and experience outlined below.

In Australia:
AQF Certificate III including at least two years of on-the-job training, or AQF Certificate IV (ANZSCO Skill Level 3)

In New Zealand:
NZ Register Level 4 qualification (ANZSCO Skill Level 3)

At least three years of relevant experience may substitute for the formal qualifications listed above. In some instances relevant experience and/or on-the-job training may be required in addition to the formal qualification.

Tasks Include:
- studying blueprints, drawings and specifications to determine job, material and equipment requirements
- selecting metal stock, such as stainless steel, galvanised iron, mild steel, aluminium and copper, and checking sizes, gauges and other dimensions of metal stock against specifications
- marking out metal stock with reference points and lines, using templates, gauges and other measuring instruments
- cutting metal stock along guidelines using hand and power shears, guillotines and drills
- shaping and forming cut metal stock into products using folding and bending machines, rollers, presses and hammers
- fitting and assembling components into final products by welding, riveting, soldering, brazing and otherwise joining
- finishing products by polishing, filing, sanding and cleaning assembled products
- may repair damaged sheetmetal products and components
- may specialise in fabrication, or on-site assembly and installation, of sheetmetal products
- may produce aircraft sheet metal components requiring advanced drawing and calculating skills
- may specialise in decorative copperwork

Occupation:
322211 Sheetmetal Trades Worker

322211 SHEETMETAL TRADES WORKER

Marks out, shapes, forms and joins sheetmetal and other materials to make products and components.

Skill Level: 3

Specialisations:
Metal Spinner
Sheetmetal Patternmaker

UNIT GROUP 3223 STRUCTURAL STEEL AND WELDING TRADES WORKERS

STRUCTURAL STEEL AND WELDING TRADES WORKERS cut, shape, join and repair metal components of iron and steel structures, boilers, pressure vessels and pipes, ships and other vessels.

Indicative Skill Level:
Most occupations in this unit group have a level of skill commensurate with the qualifications and experience outlined below.

In Australia:
AQF Certificate III including at least two years of on-the-job training, or AQF Certificate IV (ANZSCO Skill Level 3)

In New Zealand:
NZ Register Level 4 qualification (ANZSCO Skill Level 3)

At least three years of relevant experience may substitute for the formal qualifications listed above. In some instances relevant experience and/or on-the-job training may be required in addition to the formal qualification.

Tasks Include:
- studying blueprints, drawings and specifications to determine job requirements
- selecting, cleaning and preparing metal stock
- cutting marked-out metal sections and shapes using hand tools, flame cutting torches and metal cutting machines
- shaping and bending metal sections and pipes using hand and machine tools, and by heating and hammering
- aligning parts to be joined using hand tools and measuring instruments
- joining metal sections using various welding techniques, bolting and riveting
- examining welds for width of bead, penetration and precision
- finishing products by cleaning, polishing, filing and bathing in acidic solutions
- cleaning and smoothing welds by filing, chiselling and grinding

Occupations:
322311 Metal Fabricator
322312 Pressure Welder
322313 Welder (First Class) (Aus) / Welder (NZ)

322311 METAL FABRICATOR

Marks off and fabricates structural steel and other metal stock to make or repair metal products and structures such as boilers and pressure vessels.

Skill Level: 3

Specialisations:
Boilermaker-Welder
Brass Finisher
Metal Fabricator-Welder
Metal Template Maker
Structural Steel Trades Worker

UNIT GROUP 3223 STRUCTURAL STEEL AND WELDING TRADES WORKERS *continued*

322312 PRESSURE WELDER

Assembles, welds and repairs pressure vessels and pipes to relevant standards.

Skill Level: 3

322313 WELDER (FIRST CLASS) (AUS) / WELDER (NZ)

Fabricates and repairs metal products using various welding techniques.

Skill Level: 3

Specialisation:
Special Class Welder

MINOR GROUP 323 MECHANICAL ENGINEERING TRADES WORKERS

MECHANICAL ENGINEERING TRADES WORKERS machine and prepare aircraft systems, metal parts, subassemblies and precision instruments.

Motor Mechanics are excluded from this minor group. Motor Mechanics are included in Minor Group 321 Automotive Electricians and Mechanics.

Indicative Skill Level:
Most occupations in this minor group have a level of skill commensurate with the qualifications and experience outlined below.

In Australia:

AQF Certificate III including at least two years of on-the-job training, or AQF Certificate IV (ANZSCO Skill Level 3)

In New Zealand:

NZ Register Level 4 qualification (ANZSCO Skill Level 3)

At least three years of relevant experience may substitute for the formal qualifications listed above. In some instances relevant experience and/or on-the-job training may be required in addition to the formal qualification.

Tasks Include:

- forming metal stock and castings to fine tolerances using machine tools to press, cut, grind, plane, bore and drill metal
- checking fabricated and assembled metal parts for fit
- fitting parts into machines using hand tools
- dismantling, repairing and replacing defective parts, and testing and reassembling aircraft components and systems and precision instruments
- assembling parts and subassemblies of precision implements, locks, timepieces and firearms
- testing circuits in electronic timepieces and manufactured articles
- making and repairing tools, equipment and engineering patterns

Occupations in this minor group are classified into the following unit groups:

3231 Aircraft Maintenance Engineers
3232 Metal Fitters and Machinists
3233 Precision Metal Trades Workers
3234 Toolmakers and Engineering Patternmakers

UNIT GROUP 3231 AIRCRAFT MAINTENANCE ENGINEERS

AIRCRAFT MAINTENANCE ENGINEERS maintain and repair aircraft structures, and avionic and mechanical systems.

Indicative Skill Level:
Most occupations in this unit group have a level of skill commensurate with the qualifications and experience outlined below.

In Australia:
AQF Certificate III including at least two years of on-the-job training, or AQF Certificate IV (ANZSCO Skill Level 3)

In New Zealand:
NZ Register Level 4 qualification (ANZSCO Skill Level 3)

At least three years of relevant experience may substitute for the formal qualifications listed above. In some instances relevant experience and/or on-the-job training may be required in addition to the formal qualification.

Registration or licensing may be required.

Tasks Include:
- dismantling, inspecting, testing, repairing and reassembling aircraft engines, ancillary motors and engine accessories, electrical systems, and subassemblies of aircraft frames
- installing electrical circuits and equipment
- testing aircraft communication equipment, aircraft instrumentation and electronic systems using electronic testing equipment and specialised test apparatus
- replacing and testing aircraft oxygen system components
- assembling parts and subassemblies of aircraft frames
- conducting routine pre-flight inspections of engines, aircraft frames and mechanical systems
- maintaining records of action taken
- may manufacture aircraft electrical, instrument and radio hardware components

Occupations:
323111 Aircraft Maintenance Engineer (Avionics)
323112 Aircraft Maintenance Engineer (Mechanical)
323113 Aircraft Maintenance Engineer (Structures)

323111 AIRCRAFT MAINTENANCE ENGINEER (AVIONICS)

Inspects, tests, aligns, repairs and installs aircraft electrical and avionic system components. Registration or licensing may be required.

Skill Level: 3

Specialisations:
Aircraft Maintenance Engineer (Electrical)
Aircraft Maintenance Engineer (Instruments)
Aircraft Maintenance Engineer (Radio)
Avionics Technician (Defence)
Licensed Aircraft Maintenance Engineer (Electrical)
Licensed Aircraft Maintenance Engineer (Instruments)
Licensed Aircraft Maintenance Engineer (Radio)

UNIT GROUP 3231 AIRCRAFT MAINTENANCE ENGINEERS *continued*

323112 AIRCRAFT MAINTENANCE ENGINEER (MECHANICAL)

Inspects, tests, repairs and installs aircraft hydromechanical and flight system components and aircraft engines, subassemblies and components. Registration or licensing may be required.

Skill Level: 3

Specialisations:
- Aircraft Maintenance Engineer (Airframes)
- Aircraft Maintenance Engineer (Engines)
- Aircraft Technician (Air Force, Army)
- Aviation Technician (Navy)
- Licensed Aircraft Maintenance Engineer (Airframes)
- Licensed Aircraft Maintenance Engineer (Engines)

323113 AIRCRAFT MAINTENANCE ENGINEER (STRUCTURES)

Inspects, dismantles and reassembles aircraft structures, and repairs and replaces components of aircraft frames. Works with both metal and carbon fibre composite materials. Registration or licensing may be required.

Skill Level: 3

Specialisation:
- Aircraft Structural Fitter (Air Force, Army)

UNIT GROUP 3232 METAL FITTERS AND MACHINISTS

METAL FITTERS AND MACHINISTS fit and assemble fabricated metal parts into products, set up machining tools, production machines and textile machines, and operate machining tools and machines to shape metal stock and castings.

Indicative Skill Level:
Most occupations in this unit group have a level of skill commensurate with the qualifications and experience outlined below.

In Australia:

AQF Certificate III including at least two years of on-the-job training, or AQF Certificate IV (ANZSCO Skill Level 3)

In New Zealand:

NZ Register Level 4 qualification (ANZSCO Skill Level 3)

At least three years of relevant experience may substitute for the formal qualifications listed above. In some instances relevant experience and/or on-the-job training may be required in addition to the formal qualification.

Tasks Include:

- studying drawings and specifications to determine suitable material, method and sequence of operations, and machine settings
- fitting fabricated metal parts into products and assembling metal parts and subassemblies to produce machines and equipment
- checking fabricated and assembled metal parts for accuracy, clearance and fit using precision measuring instruments
- setting guides, stops and other controls on machining tools, setting up prescribed cutting and shaping tools and dies in machines and presses, and setting controls for textile machines
- forming metal stock and castings to fine tolerances using machining tools to press, cut, grind, plane, bore and drill metal
- cutting, threading, bending and installing hydraulic and pneumatic pipes and lines
- preparing pattern mechanisms to control the operation of textile machines used to spin, weave, knit, sew and tuft fabric
- diagnosing faults and performing operational maintenance of machines, and overhauling and repairing mechanical parts and fluid power equipment
- may erect machines and equipment on-site

Occupations:

323211 Fitter (General)
323212 Fitter and Turner
323213 Fitter-Welder
323214 Metal Machinist (First Class)
323215 Textile, Clothing and Footwear Mechanic
323299 Metal Fitters and Machinists nec

UNIT GROUP 3232 METAL FITTERS AND MACHINISTS *continued*

323211 FITTER (GENERAL)

Fits and assembles metal parts and subassemblies to fabricate production machines and other equipment.

Skill Level: 3

Specialisations:

- Computer Numeric Control Setter
- Diesel Fitter-Mechanic
- Fitter-Machinist
- Fitter-Mechanic
- Maintenance Fitter
- Mechanic (Diesel and Heavy Earthmoving Equipment)
- Plant Mechanic

323212 FITTER AND TURNER

Fits, assembles, grinds and shapes metal parts and subassemblies to fabricate production machines and other equipment.

Skill Level: 3

Specialisation:

- Fitter Armament (Army)

323213 FITTER-WELDER

Fits, assembles and welds metal parts and subassemblies to fabricate production machines and other equipment.

Skill Level: 3

323214 METAL MACHINIST (FIRST CLASS)

Sets up and operates machine tools to shape and form metal stock and castings to fine tolerances, using detailed drawings and specifications.

Skill Level: 3

Specialisations:

- Aircraft Machinist
- Automotive Machinist
- Metal Machine Setter
- Metal Turner
- Milling Machinist
- Vertical Borer

UNIT GROUP 3232 METAL FITTERS AND MACHINISTS *continued*

323215 TEXTILE, CLOTHING AND FOOTWEAR MECHANIC

Sets up, adjusts and maintains industrial or domestic sewing machines, or machines used in the production of yarn, textiles or footwear.

Skill Level: 3

Specialisations:
- Loom Tuner
- Sewing Machine Mechanic
- Textile Machine Mechanic

323299 METAL FITTERS AND MACHINISTS NEC

This occupation group covers Metal Fitters and Machinists not elsewhere classified.

Skill Level: 3

Occupations in this group include:
- Printing Engineer

UNIT GROUP 3233 PRECISION METAL TRADES WORKERS

PRECISION METAL TRADES WORKERS fabricate, assemble, maintain and repair metal precision instruments.

Indicative Skill Level:
Most occupations in this unit group have a level of skill commensurate with the qualifications and experience outlined below.

In Australia:
AQF Certificate III including at least two years of on-the-job training, or AQF Certificate IV (ANZSCO Skill Level 3)

In New Zealand:
NZ Register Level 4 qualification (ANZSCO Skill Level 3)

At least three years of relevant experience may substitute for the formal qualifications listed above. In some instances relevant experience and/or on-the-job training may be required in addition to the formal qualification.

Registration or licensing may be required.

Tasks Include:
- assembling parts and subassemblies of precision instruments, locks, timepieces and firearms
- dismantling precision instruments, locks, timepieces and firearms, repairing and replacing defective parts, and reassembling articles using hand and power tools and specially designed machines
- inscribing letters, figures and designs on surfaces of jewellery, trophies and other ornamental items
- installing security systems, changing tumblers in locks, changing locks, cutting keys and opening locks by manipulation
- calibrating precision instruments using standard weights and measures, jigs and fixtures, and hand tools to adjust and align parts and small balancing weights
- making blades for circular, band and other power saws and repairing, setting and sharpening blades for hand and power saws
- testing circuits in electronic timepieces
- may estimate costs and prepare quotes for repairs

Occupations:
323311 Engraver
323312 Gunsmith
323313 Locksmith
323314 Precision Instrument Maker and Repairer
323315 Saw Maker and Repairer
323316 Watch and Clock Maker and Repairer

323311 ENGRAVER

Inscribes letters, figures and designs on metal, glass, wood, rubber, plastic and other surfaces.

Skill Level: 3

323312 GUNSMITH

Modifies, services and repairs rifles, revolvers and other firearms. Registration or licensing is required.

Skill Level: 3

UNIT GROUP 3233 PRECISION METAL TRADES WORKERS *continued*

323313 LOCKSMITH

Installs and maintains locks and related security devices and systems. Registration or licensing is required.

Skill Level: 3

Specialisation:
- Safemaker

323314 PRECISION INSTRUMENT MAKER AND REPAIRER

Assembles, calibrates, installs and overhauls mechanical precision instruments and equipment.

Skill Level: 3

Specialisations:
- Camera Repairer
- Scalemaker
- Scientific Instrument Maker and Repairer

323315 SAW MAKER AND REPAIRER

Makes, repairs, sets and sharpens blades for circular, band and other saws.

Skill Level: 3

Specialisation:
- Saw Sharpener

323316 WATCH AND CLOCK MAKER AND REPAIRER

Makes, repairs, cleans and adjusts watches and clocks.

Skill Level: 3

UNIT GROUP 3234 TOOLMAKERS AND ENGINEERING PATTERNMAKERS

TOOLMAKERS AND ENGINEERING PATTERNMAKERS make and repair tools, dies, jigs, fixtures and other precision parts and equipment to fine tolerances for machine tools and other production machinery, and construct full-size engineering, visual and experimental models and models for the manufacture of prototype developmental products.

Indicative Skill Level:
Most occupations in this unit group have a level of skill commensurate with the qualifications and experience outlined below.

In Australia:
AQF Certificate III including at least two years of on-the-job training, or AQF Certificate IV (ANZSCO Skill Level 3)

In New Zealand:
NZ Register Level 4 qualification (ANZSCO Skill Level 3)

At least three years of relevant experience may substitute for the formal qualifications listed above. In some instances relevant experience and/or on-the-job training may be required in addition to the formal qualification.

Tasks Include:
- studying drawings and specifications to determine dimensions and tolerances of articles to be manufactured and models to be constructed
- measuring and marking out metal stock and castings using various gauges
- shaping metal and wood stock using machine tools
- checking accuracy of manufactured articles and finished patterns to fine tolerances, using precision measuring instruments
- testing and modifying manufactured articles
- applying protective finishes to patterns and painting pattern sections to indicate method of assembly
- assembling pattern sections and shaping work pieces to specified finish
- pouring and spreading materials into moulds and over models of patterns, and building laminations of fibreglass cloth and plastic resin to fabricate patterns
- repairing broken and damaged patterns and correcting patterns to compensate for defects in casting
- constructing templates for layout and inspection

Occupations:
323411 Engineering Patternmaker
323412 Toolmaker

323411 ENGINEERING PATTERNMAKER

Constructs full-size engineering models usually made out of timber, which are used in manufacturing to produce metal castings, copy models, vacuum form tooling and tooling for the automotive, aircraft or fibreglass industries.

Skill Level: 3

UNIT GROUP 3234 TOOLMAKERS AND ENGINEERING PATTERNMAKERS *continued*

323412 TOOLMAKER

Makes and repairs tools, dies, jigs, fixtures and other precision parts and equipment to fine tolerances for machine tools and other production machinery.

Skill Level: 3

Specialisations:

- Die Caster
- Die Sinker
- Jigmaker (Metal)
- Plastic Mould Maker
- Press-tool Maker

MINOR GROUP 324 PANELBEATERS, AND VEHICLE BODY BUILDERS, TRIMMERS AND PAINTERS

PANELBEATERS, AND VEHICLE BODY BUILDERS, TRIMMERS AND PAINTERS repair damage to motor vehicle bodies; construct purpose-built vehicle bodies; fit, repair and replace interior trim and upholstery in vehicles; and paint vehicles.

Fabrication Engineering Trades Workers are excluded from this minor group. Fabrication Engineering Trades Workers are included in Minor Group 322 Fabrication Engineering Trades Workers.

Indicative Skill Level:
Most occupations in this minor group have a level of skill commensurate with the qualifications and experience outlined below.

In Australia:
AQF Certificate III including at least two years of on-the-job training, or AQF Certificate IV (ANZSCO Skill Level 3)

In New Zealand:
NZ Register Level 4 qualification (ANZSCO Skill Level 3)

At least three years of relevant experience may substitute for the formal qualifications listed above. In some instances relevant experience and/or on-the-job training may be required in addition to the formal qualification.

Tasks Include:
- removing, replacing and repairing damaged panels and parts using mechanical and hydraulic equipment
- filling depressions with plastic filler and chiselling and sanding surfaces
- constructing framework sections in metal, wood, fibreglass and other materials using stretching and shrinking machines and welding equipment
- bolting, screwing, riveting and welding sections together to form complete frameworks
- cutting and shaping panels of sheetmetal, aluminium and reinforced plastic and attaching to frameworks using hand and power tools
- preparing new vehicle trim work according to drawings and sketches, and removing old coverings and fittings from vehicles and taking new measurements
- selecting and cutting pieces of fabric, vinyl and leather and sewing pieces together using heavy-duty sewing machines
- installing and attaching interior lining, floor coverings, armrests, door trims, rubber seals, locks and handles
- masking areas not to be painted by covering with masking tape and paper
- selecting and mixing paint shades to match vehicle colour
- applying primer and finish coats with spray-guns, and sanding surfaces between coats

Occupations in this minor group are classified into the following unit groups:
3241 Panelbeaters
3242 Vehicle Body Builders and Trimmers
3243 Vehicle Painters

UNIT GROUP 3241 PANELBEATERS

PANELBEATERS repair damage to metal, fibreglass and plastic body work on vehicles, and form replacement vehicle panels.

Indicative Skill Level:
Most occupations in this unit group have a level of skill commensurate with the qualifications and experience outlined below.

In Australia:
AQF Certificate III including at least two years of on-the-job training, or AQF Certificate IV (ANZSCO Skill Level 3)

In New Zealand:
NZ Register Level 4 qualification (ANZSCO Skill Level 3)

At least three years of relevant experience may substitute for the formal qualifications listed above. In some instances relevant experience and/or on-the-job training may be required in addition to the formal qualification.

Registration or licensing may be required.

Tasks Include:
- removing damaged panels and parts, and removing upholstery and accessories to gain access
- removing dents by hammering panels
- straightening damaged vehicles and parts using mechanical and hydraulic equipment
- replacing badly damaged sections with new or second-hand panels
- filling depressions with plastic filler, and filing, grinding and sanding repaired surfaces
- cutting and joining replacement sections using welding equipment
- fitting repaired or replacement panels on vehicles and refitting body hardware such as door locks and trims
- may assist vehicle body builders in constructing and restoring custom-designed, vintage and other specialty vehicles
- may spray-paint vehicles

Occupation:
324111 Panelbeater

324111 PANELBEATER

Alternative Title:
Vehicle Refinisher

Repairs damage to metal, fibreglass and plastic body work on vehicles and forms replacement vehicle panels. Registration or licensing may be required.

Skill Level: 3

UNIT GROUP 3242 VEHICLE BODY BUILDERS AND TRIMMERS

VEHICLE BODY BUILDERS AND TRIMMERS manufacture and repair prototype production units and purpose-built vehicle bodies, and install, repair and replace the interior trim of vehicles.

Indicative Skill Level:
Most occupations in this unit group have a level of skill commensurate with the qualifications and experience outlined below.

In Australia:
AQF Certificate III including at least two years of on-the-job training, or AQF Certificate IV (ANZSCO Skill Level 3)

In New Zealand:
NZ Register Level 4 qualification (ANZSCO Skill Level 3)

At least three years of relevant experience may substitute for the formal qualifications listed above. In some instances relevant experience and/or on-the-job training may be required in addition to the formal qualification.

Registration or licensing may be required.

Tasks Include:
- constructing framework sections in metal, wood, fibreglass and other materials using shaping machines and cutting and welding equipment
- bolting, screwing, riveting and welding sections together to form complete frameworks
- cutting and shaping panels of sheetmetal, aluminium and reinforced plastic and attaching to frameworks using hand and power tools
- modifying assembly line vehicles to special requirements
- preparing new vehicle trim work according to drawings and sketches, and removing old coverings and fittings from vehicles and taking new measurements
- selecting and cutting pieces of fabric, vinyl and leather and sewing pieces together using heavy-duty sewing machines
- installing internal trim in vehicles such as lining, floor coverings and armrests
- attaching door trims, rubber seals, locks and handles

Occupations:
324211 Vehicle Body Builder
324212 Vehicle Trimmer

324211 VEHICLE BODY BUILDER

Alternative Title:
Vehicle Body Maker

Manufactures and repairs prototype production units and purpose-built vehicle bodies such as buses, trucks, fire engines and caravans. Registration or licensing may be required.

Skill Level: 3

Specialisation:
Coach Builder

UNIT GROUP 3242 VEHICLE BODY BUILDERS AND TRIMMERS *continued*

324212 VEHICLE TRIMMER

Installs, repairs and replaces the interior trim of vehicles such as seats, linings, floor coverings and door trims. Registration or licensing may be required.

Skill Level: 3

Specialisation:
Vehicle Upholsterer

UNIT GROUP 3243 VEHICLE PAINTERS

VEHICLE PAINTERS prepare surfaces of vehicles, match and mix colours and apply paint.

Indicative Skill Level:
Most occupations in this unit group have a level of skill commensurate with the qualifications and experience outlined below.

In Australia:
AQF Certificate III including at least two years of on-the-job training, or AQF Certificate IV (ANZSCO Skill Level 3)

In New Zealand:
NZ Register Level 4 qualification (ANZSCO Skill Level 3)

At least three years of relevant experience may substitute for the formal qualifications listed above. In some instances relevant experience and/or on-the-job training may be required in addition to the formal qualification.

Registration or licensing may be required.

Tasks Include:
- removing rough spots on vehicle panels
- sanding surfaces by hand and with power sanders
- masking areas not to be painted with tape and paper
- colour matching and mixing paints to match paint shades, and selecting pre-mixed paint
- applying primer and finishing coats using spray-guns, and sanding surfaces between coats
- touching up paintwork and applying polish to vehicles
- removing masking papers, and waxing and polishing finished paintwork
- painting signs and artwork on vehicles
- treating vehicles with rust-proofing chemicals

Occupation:
324311 Vehicle Painter

324311 VEHICLE PAINTER

Prepares surfaces of vehicles, matches and mixes colours and applies paint. Registration or licensing may be required.

Skill Level: 3

SUB-MAJOR GROUP 33 CONSTRUCTION TRADES WORKERS

CONSTRUCTION TRADES WORKERS construct and repair buildings and other structures, apply final finishes such as plaster, painting and flooring, make and install glass products, and provide plumbing, drainage and mechanical services.

Indicative Skill Level:
Most occupations in this sub-major group have a level of skill commensurate with the qualifications and experience outlined below.

In Australia:

AQF Certificate III including at least two years of on-the-job training, or AQF Certificate IV (ANZSCO Skill Level 3)

In New Zealand:

NZ Register Level 4 qualification (ANZSCO Skill Level 3)

At least three years of relevant experience may substitute for the formal qualifications listed above. In some instances relevant experience and/or on-the-job training may be required in addition to the formal qualification.

Tasks Include:

- studying drawings and plans, and estimating quantities of materials required
- laying bricks and blocks, spreading mortar between joints, and removing excess mortar
- erecting frameworks, roof frames, roofs and walls
- laying flooring, carpets and tiles
- painting and wallpapering surfaces
- applying plaster and cement coatings to walls
- building and installing fittings
- making and installing glass products
- installing plumbing and drainage systems, guttering and other rainwater systems
- installing gas appliances and airconditioning piping and ducting

Occupations in this sub-major group are classified into the following minor groups:

331 Bricklayers, and Carpenters and Joiners
332 Floor Finishers and Painting Trades Workers
333 Glaziers, Plasterers and Tilers
334 Plumbers

MINOR GROUP 331 BRICKLAYERS, AND CARPENTERS AND JOINERS

BRICKLAYERS, CARPENTERS AND JOINERS construct and erect buildings and other structures of brick, stone and timber.

Indicative Skill Level:
Most occupations in this minor group have a level of skill commensurate with the qualifications and experience outlined below.

In Australia:
AQF Certificate III including at least two years of on-the-job training, or AQF Certificate IV (ANZSCO Skill Level 3)

In New Zealand:
NZ Register Level 4 qualification (ANZSCO Skill Level 3)

At least three years of relevant experience may substitute for the formal qualifications listed above. In some instances relevant experience and/or on-the-job training may be required in addition to the formal qualification.

Tasks Include:
- studying plans and specifications to determine materials required, dimensions and installation procedures
- erecting frameworks, roof framing and scaffolding, and laying sub-floors and floorboards
- operating machines and hand tools to cut and shape stones, bricks and timber
- laying bricks and stone blocks in rows and securing with mortar to construct walls
- assembling prepared wood to form structures ready to install
- nailing fascia panels, sheathing roofs, and fitting wall cladding and door and window frames
- checking vertical and horizontal alignment

Occupations in this minor group are classified into the following unit groups:
3311 Bricklayers and Stonemasons
3312 Carpenters and Joiners

UNIT GROUP 3311 BRICKLAYERS AND STONEMASONS

BRICKLAYERS AND STONEMASONS lay bricks, pre-cut stones and other types of building blocks in mortar to construct and repair walls, partitions, arches and other structures, and cut and shape hard and soft stone blocks and masonry slabs for the construction and renovation of stone structures and monumental masonry.

Indicative Skill Level:
Most occupations in this unit group have a level of skill commensurate with the qualifications and experience outlined below.

In Australia:
AQF Certificate III including at least two years of on-the-job training, or AQF Certificate IV (ANZSCO Skill Level 3)

In New Zealand:
NZ Register Level 4 qualification (ANZSCO Skill Level 3)

At least three years of relevant experience may substitute for the formal qualifications listed above. In some instances relevant experience and/or on-the-job training may be required in addition to the formal qualification.

Registration or licensing may be required.

Tasks Include:
- studying plans and specifications to determine materials required, dimensions and installation procedures
- erecting and dismantling restricted height scaffolding
- sealing foundations with damp-resistant materials and spreading layers of mortar to serve as base and binder for blocks using trowels
- laying bricks in rows, designs and shapes, and spreading mortar between joints
- embedding blocks in mortar and removing excess mortar
- checking vertical and horizontal alignment
- cutting, shaping and polishing stones and bricks using machines and hand tools, and shaping bricks to fit irregular spaces
- repairing and maintaining bricks, cement blocks and related structures
- designing and cutting monumental masonry and lettering
- constructing walls using stone slabs and large masonry slab blocks

Occupations:
331111 Bricklayer
331112 Stonemason

UNIT GROUP 3311 BRICKLAYERS AND STONEMASONS *continued*

331111 BRICKLAYER

Alternative Title:

Blocklayer

Lays bricks, pre-cut stone and other types of building blocks in mortar to construct and repair walls, partitions, arches and other structures. Registration or licensing may be required.

Skill Level: 3

Specialisations:

Arch Builder
Chimney Builder
Refractory Bricklayer
Retort Setter (Bricklaying)
Tuckpointer

331112 STONEMASON

Cuts and shapes hard and soft stone blocks and masonry slabs to construct and renovate stone structures and monumental masonry. Registration or licensing may be required.

Skill Level: 3

Specialisations:

Construction Stonemason
Monumental Stonemason

UNIT GROUP 3312 CARPENTERS AND JOINERS

CARPENTERS AND JOINERS construct, erect, install, renovate and repair structures and fixtures made of wood, plywood, wallboard and other materials, and cut, shape and fit timber parts to form structures and fittings.

Indicative Skill Level:
Most occupations in this unit group have a level of skill commensurate with the qualifications and experience outlined below.

In Australia:
AQF Certificate III including at least two years of on-the-job training, or AQF Certificate IV (ANZSCO Skill Level 3)

In New Zealand:
NZ Register Level 4 qualification (ANZSCO Skill Level 3)

At least three years of relevant experience may substitute for the formal qualifications listed above. In some instances relevant experience and/or on-the-job training may be required in addition to the formal qualification.

Registration or licensing may be required.

Tasks Include:
- studying drawings and specifications to determine materials required, dimensions and installation procedures
- ordering and selecting timbers and materials, and preparing layouts
- cutting materials, and assembling and nailing cut and shaped parts
- erecting framework and roof framing, laying sub-flooring and floorboards and verifying trueness of structures
- nailing fascia panels, sheathing roofs, and fitting exterior wall cladding and door and window frames
- assembling prepared wood to form structures and fittings ready to install
- cutting wood joints
- may construct concrete formwork
- may repair existing fittings
- may work with plastic laminates, perspex and metals

Occupations:
331211 Carpenter and Joiner
331212 Carpenter
331213 Joiner

331211 CARPENTER AND JOINER

Constructs and installs structures and fixtures of wood, plywood, and wallboard, and cuts, shapes and fits timber parts to form structures and fittings. Registration or licensing may be required.

Skill Level: 3

Specialisation:
Shopfitter

UNIT GROUP 3312 CARPENTERS AND JOINERS *continued*

331212 CARPENTER

Constructs, erects, installs, renovates and repairs structures and fixtures of wood, plywood, wallboard and other materials. Registration or licensing may be required.

Skill Level: 3

Specialisations:
- Fixing Carpenter
- Formwork Carpenter
- Prop and Scenery Maker

331213 JOINER

Cuts, shapes and fits timber parts in workshops to form structures and fittings, ready for installation. Registration or licensing may be required.

Skill Level: 3

Specialisations:
- Joinery Machinist
- Joinery Patternmaker
- Joinery Setter-out

MINOR GROUP 332 FLOOR FINISHERS AND PAINTING TRADES WORKERS

FLOOR FINISHERS AND PAINTING TRADES WORKERS install and repair soft and resilient floor coverings, and apply paint, varnish, wallpaper and other finishes to protect, maintain and decorate the surfaces of buildings and structures.

Indicative Skill Level:
Most occupations in this minor group have a level of skill commensurate with the qualifications and experience outlined below.

In Australia:
AQF Certificate III including at least two years of on-the-job training, or AQF Certificate IV (ANZSCO Skill Level 3)

In New Zealand:
NZ Register Level 4 qualification (ANZSCO Skill Level 3)

At least three years of relevant experience may substitute for the formal qualifications listed above. In some instances relevant experience and/or on-the-job training may be required in addition to the formal qualification.

Tasks Include:

- measuring areas to be covered and consulting plans to estimate quantities of floor covering materials required
- preparing surfaces by removing old floor coverings, paint and wallpaper, fixing woodwork, filling holes and cracks, smoothing and sealing surfaces, and removing baseboard trims
- measuring, cutting and fixing underlay materials
- laying underlay and covering materials on floors, matching patterns, cutting shapes around fixtures and trimming edges
- selecting and preparing paints to required colours by mixing portions of pigment, oil, thinning and drying additives
- applying paints, varnishes and stains to surfaces using brushes, rollers and sprays, and hanging wallpaper

Occupations in this minor group are classified into the following unit groups:
3321 Floor Finishers
3322 Painting Trades Workers

UNIT GROUP 3321 FLOOR FINISHERS

FLOOR FINISHERS measure, cut, install and repair soft and resilient floor coverings.

Indicative Skill Level:
Most occupations in this unit group have a level of skill commensurate with the qualifications and experience outlined below.

In Australia:
AQF Certificate III including at least two years of on-the-job training, or AQF Certificate IV (ANZSCO Skill Level 3)

In New Zealand:
NZ Register Level 4 qualification (ANZSCO Skill Level 3)

At least three years of relevant experience may substitute for the formal qualifications listed above. In some instances relevant experience and/or on-the-job training may be required in addition to the formal qualification.

Registration or licensing may be required.

Tasks Include:

- measuring areas to be covered and consulting plans to estimate quantities of floor covering materials required
- preparing surfaces for covering and removing baseboard trims
- measuring, cutting and fixing underlay materials
- laying coverings, such as carpets, linoleum, parquetry blocks, cork tiles and other resilient flooring materials, over floors, matching patterns, cutting shapes around fixtures and trimming edges
- securing floor coverings and fitting edge trims in doorways
- sanding, staining and applying finishing coatings to timber floors
- may install wall, ceiling, counter and bench coverings

Occupation:
332111 Floor Finisher

332111 FLOOR FINISHER

Measures, cuts, installs and repairs soft and resilient floor coverings. Registration or licensing may be required.

Skill Level: 3

Specialisations:
Carpet Layer
Parquetry Layer

UNIT GROUP 3322 PAINTING TRADES WORKERS

PAINTING TRADES WORKERS apply paint, varnish, wallpaper and other finishes to protect, maintain and decorate surfaces of buildings and structures.

Indicative Skill Level:
Most occupations in this unit group have a level of skill commensurate with the qualifications and experience outlined below.

In Australia:
AQF Certificate III including at least two years of on-the-job training, or AQF Certificate IV (ANZSCO Skill Level 3)

In New Zealand:
NZ Register Level 4 qualification (ANZSCO Skill Level 3)

At least three years of relevant experience may substitute for the formal qualifications listed above. In some instances relevant experience and/or on-the-job training may be required in addition to the formal qualification.

Registration or licensing may be required.

Tasks Include:
- erecting scaffolding and ladders, and placing drop sheets to protect adjacent areas from paint splattering
- preparing surfaces by removing old paint and wallpaper, fixing woodwork, filling holes and cracks, and smoothing and sealing surfaces
- selecting and preparing paints to required colours by mixing portions of pigment, oil, and thinning and drying additives
- applying paints, varnishes and stains to surfaces using brushes, rollers and sprays
- hanging wallpaper, matching patterns and trimming edges
- cleaning equipment and work areas
- may repair windows and replace glass in wooden and metal frames
- may lay and repair wall and floor tiles

Occupation:
332211 Painting Trades Worker

332211 PAINTING TRADES WORKER

Applies paint, varnish, wallpaper and other finishes to protect, maintain and decorate surfaces of buildings and structures. Registration or licensing may be required.

Skill Level: 3

Specialisation:
Paperhanger

MINOR GROUP 333 GLAZIERS, PLASTERERS AND TILERS

GLAZIERS, PLASTERERS AND TILERS cut and install flat glass, apply plaster and secure plasterboard and suspended ceilings, and lay tiles on roofs, walls and floors.

Indicative Skill Level:
Most occupations in this minor group have a level of skill commensurate with the qualifications and experience outlined below.

In Australia:
AQF Certificate III including at least two years of on-the-job training, or AQF Certificate IV (ANZSCO Skill Level 3)

In New Zealand:
NZ Register Level 4 qualification (ANZSCO Skill Level 3)

At least three years of relevant experience may substitute for the formal qualifications listed above. In some instances relevant experience and/or on-the-job training may be required in addition to the formal qualification.

Tasks Include:
- studying drawings and taking measurements to determine materials required
- preparing surfaces and materials by removing old tiles, grout and adhesive, mixing plaster and cement, and marking and cutting glass
- installing glass and mirrors in windows, skylights, display cases, interior walls and ceilings
- measuring, marking and cutting plasterboard, lifting and positioning panels, and securing them to walls, ceilings and battens
- applying coats of plaster to structures using trowels, and levelling and smoothing coats to uniform thickness
- aligning starter rows of roofing material with edges of roofs, securing with wire, staples or nails, and overlapping successive layers of tiles
- spreading adhesive onto prepared surfaces and tiles, and setting tiles in position
- grouting tiles, and ensuring surfaces are waterproof

Occupations in this minor group are classified into the following unit groups:

3331 Glaziers
3332 Plasterers
3333 Roof Tilers
3334 Wall and Floor Tilers

UNIT GROUP 3331 GLAZIERS

GLAZIERS measure, cut, finish, fit and install flat glass and mirrors.

Indicative Skill Level:
Most occupations in this unit group have a level of skill commensurate with the qualifications and experience outlined below.

In Australia:
AQF Certificate III including at least two years of on-the-job training, or AQF Certificate IV (ANZSCO Skill Level 3)

In New Zealand:
NZ Register Level 4 qualification (ANZSCO Skill Level 3)

At least three years of relevant experience may substitute for the formal qualifications listed above. In some instances relevant experience and/or on-the-job training may be required in addition to the formal qualification.

Tasks Include:
- determining type and dimensions of glass required
- laying glass over patterns on padded tables and in jigs
- measuring and marking glass for cutting
- examining glass and marking defective areas
- cutting along patterns and templates
- breaking off sheets and excess glass with notched tools and glass pliers
- installing glass and mirrors in windows, skylights, display cases, interior walls and ceilings
- smoothing rough edges using belt sanders and smoothing wheels
- may coat, cut, etch, trim and treat glass to achieve special effects

Occupation:
333111 Glazier

333111 GLAZIER

Measures, cuts, finishes, fits and installs flat glass and mirrors.

Skill Level: 3

Specialisations:
Glass Beveller
Glass Embosser
Glass Etcher
Glass Silverer

UNIT GROUP 3332 PLASTERERS

PLASTERERS apply and fix plasterboard partitions, suspended ceilings, fire rating systems, acoustic tiles, and composite wall linings to buildings, and apply decorative and protective coverings of plaster, cement and similar materials to the interiors and exteriors of structures.

Indicative Skill Level:
Most occupations in this unit group have a level of skill commensurate with the qualifications and experience outlined below.

In Australia:
AQF Certificate III including at least two years of on-the-job training, or AQF Certificate IV (ANZSCO Skill Level 3)

In New Zealand:
NZ Register Level 4 qualification (ANZSCO Skill Level 3)

At least three years of relevant experience may substitute for the formal qualifications listed above. In some instances relevant experience and/or on-the-job training may be required in addition to the formal qualification.

Registration or licensing may be required.

Tasks Include:

- determining plasterboard layout, and installing insulation and vapour barriers
- measuring, marking and cutting plasterboard, lifting and positioning panels, and securing them to walls, ceilings and battens
- preparing corner beads and securing them in position
- fixing pre-cast cornices, panel mouldings, ceiling centres and other plaster fittings
- covering joins and nail holes with wet plaster and sealing compounds, and smoothing them using wet brushes and sand paper
- mixing and applying coats of plaster, cement and render to structures using trowels, and levelling and smoothing coats to uniform thickness
- plumbing and straightening corners, angles and wall and ceiling surfaces
- creating decorative textures in finishing coats
- applying and finishing acoustic, insulating and fireproofing materials bonded with plaster, plastic cement and similar materials

Occupations:
333211 Fibrous Plasterer
333212 Solid Plasterer

333211 FIBROUS PLASTERER

Applies and fixes plasterboard partitions, suspended ceilings, fire rating systems, acoustic tiles, and composite wall linings to buildings. Registration or licensing may be required.

Skill Level: 3

Specialisation:
Dry Wall Plasterer

333212 SOLID PLASTERER

Applies decorative and protective coverings of plaster, cement and similar materials to the interiors and exteriors of structures. Registration or licensing may be required.

Skill Level: 3

UNIT GROUP 3333 ROOF TILERS

ROOF TILERS cover roofs with tiles, sheets and shingles to form a waterproof surface.

Indicative Skill Level:
Most occupations in this unit group have a level of skill commensurate with the qualifications and experience outlined below.

In Australia:
AQF Certificate III including at least two years of on-the-job training, or AQF Certificate IV (ANZSCO Skill Level 3)

In New Zealand:
NZ Register Level 4 qualification (ANZSCO Skill Level 3)

At least three years of relevant experience may substitute for the formal qualifications listed above. In some instances relevant experience and/or on-the-job training may be required in addition to the formal qualification.

Registration or licensing may be required.

Tasks Include:
- studying drawings, specifications and work sites to determine materials required
- erecting ladders and scaffolds
- placing and securing waterproof sheets over eaves
- nailing and stapling roofing underlay to roofs
- aligning starter rows of roofing material with edges of roofs, securing with wire, staples and nails, and overlapping successive layers of tiles
- sizing and cutting roofing material to fit around vents, chimney edges, corners and ridges
- fixing edge and ridge tiles in cement mortar
- slipping roofing material under pre-fabricated flashing and nailing it down
- caulking and flashing exposed nail heads to prevent leaks

Occupation:
333311 Roof Tiler

333311 ROOF TILER

Cover roofs with tiles, sheets and shingles to form a waterproof surface. Registration or licensing may be required.

Skill Level: 3

Specialisations:
Roof Fixer
Roof Shingler
Roof Slater

UNIT GROUP 3334 WALL AND FLOOR TILERS

WALL AND FLOOR TILERS lay ceramic, clay, slate, marble and glass tiles on external and internal walls and floors to provide protective and decorative finishes.

Indicative Skill Level:
Most occupations in this unit group have a level of skill commensurate with the qualifications and experience outlined below.

In Australia:
AQF Certificate III including at least two years of on-the-job training, or AQF Certificate IV (ANZSCO Skill Level 3)

In New Zealand:
NZ Register Level 4 qualification (ANZSCO Skill Level 3)

At least three years of relevant experience may substitute for the formal qualifications listed above. In some instances relevant experience and/or on-the-job training may be required in addition to the formal qualification.

Registration or licensing may be required.

Tasks Include:
- examining plans, measuring and marking surfaces and laying out work
- preparing wall and floor surfaces by removing old tiles, grout and adhesive, filling holes and cracks, and cleaning surfaces
- spreading adhesive onto prepared surfaces and tiles, and setting tiles in position
- using tile-cutting tools to cut and shape tiles needed for edges and corners, and around objects such as fittings and pipes
- ensuring tiles are correctly aligned and spaced
- grouting tiles, and cleaning and removing excess grout
- applying waterproofing systems
- may lay floors of granolithic, terrazzo, cement or similar composition
- may lay coloured tiles in patterns to create mosaics

Occupation:
333411 Wall and Floor Tiler

333411 WALL AND FLOOR TILER

Lays ceramic, clay, slate, marble and glass tiles on external and internal walls and floors to provide protective and decorative finishes. Registration or licensing may be required.

Skill Level: 3

Specialisations:
Ceramic Tiler
Mosaic Tiler

MINOR GROUP 334 PLUMBERS

PLUMBERS install, maintain and repair pipes, drains, guttering and metal roofing, mechanical services and related equipment for water supply, gas, drainage, sewerage, heating, cooling and ventilation systems.

Indicative Skill Level:
Most occupations in this minor group have a level of skill commensurate with the qualifications and experience outlined below.

In Australia:
AQF Certificate III including at least two years of on-the-job training, or AQF Certificate IV (ANZSCO Skill Level 3)

In New Zealand:
NZ Register Level 4 qualification (ANZSCO Skill Level 3)

At least three years of relevant experience may substitute for the formal qualifications listed above. In some instances relevant experience and/or on-the-job training may be required in addition to the formal qualification.

Tasks Include:
- studying blueprints, drawings and specifications to determine the layout of plumbing systems and materials required
- setting out and installing hot and cold water systems and associated equipment
- installing water-based fire protections systems, including fire hydrants, hose reels and sprinkler systems
- designing and installing sanitary plumbing and water supply systems, discharge pipes and sanitary fixtures
- fabricating and installing soil and waste stacks
- assembling and installing mechanical services plant, air handling and conditioning equipment and small bore heating systems
- installing sewerage and effluent pumping equipment and disposal systems
- installing below-ground drainage systems and associated ground support systems
- installing gas appliances, flues and pressure regulating devices
- fabricating and installing metal roofing, rainwater goods and flashings

Occupations in this minor group are classified into the following unit group:
3341 Plumbers

UNIT GROUP 3341 PLUMBERS

PLUMBERS install, maintain and repair pipes, drains, guttering and metal roofing, mechanical services and related equipment for water supply, gas, drainage, sewerage, heating, cooling and ventilation systems.

Indicative Skill Level:
Most occupations in this unit group have a level of skill commensurate with the qualifications and experience outlined below.

In Australia:
AQF Certificate III including at least two years of on-the-job training, or AQF Certificate IV (ANZSCO Skill Level 3)

In New Zealand:
NZ Register Level 4 qualification (ANZSCO Skill Level 3)

At least three years of relevant experience may substitute for the formal qualifications listed above. In some instances relevant experience and/or on-the-job training may be required in addition to the formal qualification.

Registration or licensing is required.

Tasks Include:
- studying blueprints, drawings and specifications to determine the layout of plumbing systems and materials required
- setting out and installing hot and cold water systems and associated equipment
- installing water-based fire protections systems, including fire hydrants, hose reels and sprinkler systems
- designing and installing sanitary plumbing and water supply systems, discharge pipes and sanitary fixtures
- fabricating and installing soil and waste stacks
- assembling and installing mechanical services plant, air handling and conditioning equipment and small bore heating systems
- installing sewerage and effluent pumping equipment and disposal systems
- installing below-ground drainage systems and associated ground support systems
- installing gas appliances, flues and pressure regulating devices
- fabricating and installing metal roofing, rainwater goods and flashings

Occupations:
334111 Plumber (General)
334112 Airconditioning and Mechanical Services Plumber
334113 Drainer (Aus) / Drainlayer (NZ)
334114 Gasfitter
334115 Roof Plumber

334111 PLUMBER (GENERAL)

Installs and repairs water, drainage, gas and sewerage pipes and systems. Registration or licensing is required.

Skill Level: 3

Specialisations:
Fire Services Plumber
Sanitary Plumber
Water Plumber

UNIT GROUP 3341 PLUMBERS *continued*

334112 AIRCONDITIONING AND MECHANICAL SERVICES PLUMBER

Installs, maintains and repairs piping, ducting and equipment for heating, cooling and ventilation of buildings or vessels. Registration or licensing is required.

Skill Level: 3

Specialisation:
Ductfixing Plumber

334113 DRAINER (AUS) / DRAINLAYER (NZ)

Installs, maintains and designs below-ground drainage systems and associated sewerage or effluent disposal systems. Registration or licensing is required.

Skill Level: 3

Specialisation:
Septic Tank Installer

334114 GASFITTER

Installs, maintains and repairs gas mains, piping systems downstream of the billing meter, and appliances and ancillary equipment associated with the use of fuel gases, including liquefied petroleum gas systems. Registration or licensing is required.

Skill Level: 3

Specialisations:
Gas Main and Line Fitter
Liquefied Petroleum Gasfitter

334115 ROOF PLUMBER

Installs, maintains and repairs flashings, metallic roof and wall claddings and rainwater products such as gutters and downpipes. Registration or licensing is required.

Skill Level: 3

Specialisation:
Industrial Roof Plumber

SUB-MAJOR GROUP 34 ELECTROTECHNOLOGY AND TELECOMMUNICATIONS TRADES WORKERS

ELECTROTECHNOLOGY AND TELECOMMUNICATIONS TRADES WORKERS assemble, install, test and repair electrical appliances, networks and circuits, electronic systems and equipment, lifts, refrigeration and airconditioning equipment, electrical distribution networks, and telecommunications equipment.

Indicative Skill Level:
Most occupations in this sub-major group have a level of skill commensurate with the qualifications and experience outlined below.

In Australia:
AQF Certificate III including at least two years of on-the-job training, or AQF Certificate IV (ANZSCO Skill Level 3)

In New Zealand:
NZ Register Level 4 qualification (ANZSCO Skill Level 3)

At least three years of relevant experience may substitute for the formal qualifications listed above. In some instances relevant experience and/or on-the-job training may be required in addition to the formal qualification.

Tasks Include:
- examining blueprints, wiring diagrams and specifications to determine location and connections for installations
- testing for, locating and repairing electrical malfunctions
- cutting and connecting wiring and cables
- assembling, fabricating and installing electrical and electronic systems, networks, components and appliances
- installing, testing and adjusting electrical and mechanical parts of lifts
- installing and maintaining refrigeration and airconditioning equipment, and connecting electrical systems and appliances to power supplies
- installing, maintaining and repairing telecommunications and data transmission equipment
- transmitting and receiving radio messages

Occupations in this sub-major group are classified into the following minor groups:
341 Electricians
342 Electronics and Telecommunications Trades Workers

MINOR GROUP 341 ELECTRICIANS

ELECTRICIANS design, assemble, install, test, commission, diagnose, maintain and repair electrical networks, systems, circuits, equipment, components, appliances and facilities for industrial, commercial and domestic purposes, and service and repair lifts, escalators and related equipment.

Automotive Electricians are excluded from this minor group. Automotive Electricians are included in Minor Group 321 Automotive Electricians and Mechanics.

Indicative Skill Level:
Most occupations in this minor group have a level of skill commensurate with the qualifications and experience outlined below.

In Australia:
AQF Certificate III including at least two years of on-the-job training, or AQF Certificate IV (ANZSCO Skill Level 3)

In New Zealand:
NZ Register Level 4 qualification (ANZSCO Skill Level 3)

At least three years of relevant experience may substitute for the formal qualifications listed above. In some instances relevant experience and/or on-the-job training may be required in addition to the formal qualification.

Tasks Include:
- examining blueprints, wiring diagrams and specifications to determine sequences and methods of operation
- measuring and laying out installation reference points
- selecting, cutting and connecting wire and cable to terminals and connectors
- using electrical and electronic test instruments to trace and diagnose faults
- repairing or replacing faulty wiring and defective parts
- positioning and installing electrical switchboards
- connecting electrical systems to power supply
- testing continuity of circuit
- installing, testing and adjusting electric and mechanical parts of lifts

Occupations in this minor group are classified into the following unit group:
3411 Electricians

UNIT GROUP 3411 ELECTRICIANS

ELECTRICIANS design, assemble, install, test, commission, diagnose, maintain and repair electrical networks, systems, circuits, equipment, components, appliances and facilities for industrial, commercial and domestic purposes, and service and repair lifts, escalators and related equipment.

Automotive Electricians are excluded from this unit group. Automotive Electricians are included in Unit Group 3211 Automotive Electricians.

Indicative Skill Level:
Most occupations in this unit group have a level of skill commensurate with the qualifications and experience outlined below.

In Australia:
AQF Certificate III including at least two years of on-the-job training, or AQF Certificate IV (ANZSCO Skill Level 3)

In New Zealand:
NZ Register Level 4 qualification (ANZSCO Skill Level 3)

At least three years of relevant experience may substitute for the formal qualifications listed above. In some instances relevant experience and/or on-the-job training may be required in addition to the formal qualification.

Registration or licensing is required.

Tasks Include:
- examining blueprints, wiring diagrams and specifications to determine sequences and methods of operation
- measuring and laying out installation reference points
- selecting, cutting and connecting wire and cable to terminals and connectors
- using electrical and electronic test instruments to trace and diagnose faults
- repairing and replacing faulty wiring and defective parts
- positioning and installing electrical switchboards
- connecting electrical systems to power supply
- testing continuity of circuit
- installing, testing and adjusting electric and mechanical parts of lifts

Occupations:
341111 Electrician (General)
341112 Electrician (Special Class)
341113 Lift Mechanic

UNIT GROUP 3411 ELECTRICIANS *continued*

341111 ELECTRICIAN (GENERAL)

Alternative Title:
- Electrical Fitter

Installs, tests, connects, commissions, maintains and modifies electrical equipment, wiring and control systems. Registration or licensing is required.

Skill Level: 3

Specialisations:
- Armature Winder
- Electrical Contractor
- Heavy Coil Winder
- Railway Signal Electrician

341112 ELECTRICIAN (SPECIAL CLASS)

Services and repairs intricate and complex electrical and electronic circuitry. Registration or licensing is required.

Skill Level: 3

341113 LIFT MECHANIC

Alternative Title:
- Lift Electrician

Designs, installs, maintains, services and repairs electric and hydraulic passenger and freight lifts, escalators, moving walkways and other lift equipment. Registration or licensing is required.

Skill Level: 3

MINOR GROUP 342 ELECTRONICS AND TELECOMMUNICATIONS TRADES WORKERS

ELECTRONICS AND TELECOMMUNICATIONS TRADES WORKERS assemble, install, test and repair electronic systems and equipment, electrical distribution and telecommunications networks, airconditioning and refrigeration equipment, business machines and telecommunications equipment, and transmit and receive radio messages.

Indicative Skill Level:
Most occupations in this minor group have a level of skill commensurate with the qualifications and experience outlined below.

In Australia:
AQF Certificate III including at least two years of on-the-job training, or AQF Certificate IV (ANZSCO Skill Level 3)

In New Zealand:
NZ Register Level 4 qualification (ANZSCO Skill Level 3)

At least three years of relevant experience may substitute for the formal qualifications listed above. In some instances relevant experience and/or on-the-job training may be required in addition to the formal qualification.

Tasks Include:
- examining blueprints, wiring diagrams and specifications to determine job requirements
- testing for, locating, diagnosing and repairing electrical malfunctions
- cutting and connecting wiring and cables
- installing and maintaining airconditioning and refrigeration equipment, and connecting electrical systems and appliances to power supplies
- installing conductors, aerial and underground cables, and equipment associated with electrical supply
- installing, modifying, maintaining, adjusting and repairing business machines, radio and television receivers, audio and visual reproduction equipment, and electronic instruments and control systems
- transmitting and receiving radio messages
- installing, maintaining and repairing telecommunications and voice and data transmission equipment and appliances

Occupations in this minor group are classified into the following unit groups:
3421 Airconditioning and Refrigeration Mechanics
3422 Electrical Distribution Trades Workers
3423 Electronics Trades Workers
3424 Telecommunications Trades Workers

UNIT GROUP 3421 AIRCONDITIONING AND REFRIGERATION MECHANICS

AIRCONDITIONING AND REFRIGERATION MECHANICS assemble, install, maintain and repair industrial, commercial and domestic airconditioning and refrigeration systems and equipment.

Indicative Skill Level:
Most occupations in this unit group have a level of skill commensurate with the qualifications and experience outlined below.

In Australia:
AQF Certificate III including at least two years of on-the-job training, or AQF Certificate IV (ANZSCO Skill Level 3)

In New Zealand:
NZ Register Level 4 qualification (ANZSCO Skill Level 3)

At least three years of relevant experience may substitute for the formal qualifications listed above. In some instances relevant experience and/or on-the-job training may be required in addition to the formal qualification.

Registration or licensing may be required.

Tasks Include:
- establishing job requirements from drawings and specifications, and laying out installation reference points
- drilling holes, installing mounting brackets and cutting, bending and threading piping
- installing and repairing components such as compressors, motors, condensers, evaporators, switches and gauges, and copper lines for steam, gas, refrigerant, compressed air, oil and chilled water
- bolting, soldering, riveting, welding and brazing pipes to connect equipment, and checking alignment and accuracy of fit
- filling systems with gas or fluid to check for leaks
- test-operating refrigeration systems, checking mechanisms and making adjustments
- removing test gas and fluid using vacuum pumps, and filling with refrigerant
- checking and overhauling refrigeration systems, diagnosing faults and repairing and replacing defective components
- adjusting system controls and mechanisms and reassembling systems
- recording causes of malfunctioning and action taken

Occupation:
342111 Airconditioning and Refrigeration Mechanic

342111 AIRCONDITIONING AND REFRIGERATION MECHANIC

Assembles, installs, maintains and repairs industrial, commercial and domestic airconditioning and refrigeration systems and equipment. Registration or licensing may be required.

Skill Level: 3

UNIT GROUP 3422 ELECTRICAL DISTRIBUTION TRADES WORKERS

ELECTRICAL DISTRIBUTION TRADES WORKERS prepare, install, repair, maintain and patrol electric power distribution networks.

Indicative Skill Level:
Most occupations in this unit group have a level of skill commensurate with the qualifications and experience outlined below.

In Australia:
AQF Certificate III including at least two years of on-the-job training, or AQF Certificate IV (ANZSCO Skill Level 3)

In New Zealand:
NZ Register Level 4 qualification (ANZSCO Skill Level 3)

At least three years of relevant experience may substitute for the formal qualifications listed above. In some instances relevant experience and/or on-the-job training may be required in addition to the formal qualification.

Registration or licensing may be required.

Tasks Include:
- installing conductors and aerial equipment, and underground cables and equipment
- installing and maintaining equipment associated with electrical supply such as transformers
- attending to electrical breakdown and emergencies
- maintaining poles and associated hardware, and continuity of electrical supply and street lighting
- conducting routine maintenance on the aerial and underground electricity supply network
- conducting low-voltage switching operations
- fitting pole hardware and crossarms
- preparing low- and high-voltage cable joints and cable terminations while connecting and installing electrical equipment and overhead lines
- using heavy plant equipment such as elevated work platforms and portable equipment such as hydraulic drills
- may undertake substation installation and maintenance, and specialised testing and revenue meter installation

Occupations:
342211 Electrical Linesworker (Aus) / Electrical Line Mechanic (NZ)
342212 Technical Cable Jointer

342211 ELECTRICAL LINESWORKER (AUS) / ELECTRICAL LINE MECHANIC (NZ)

Installs, maintains, repairs and patrols electrical sub-transmission and distribution systems. Registration or licensing may be required.

Skill Level: 3

Specialisations:
Electrical Linesworker (Distribution) (Aus) / Electrical Line Mechanic (Distribution) (NZ)
Electrical Linesworker (Transmission) (Aus) / Electrical Line Mechanic (Transmission) (NZ)
Railway Traction Line Worker

UNIT GROUP 3422 ELECTRICAL DISTRIBUTION TRADES WORKERS *continued*

342212 TECHNICAL CABLE JOINTER

Joins insulated electric power cables installed in underground conduits and trenches, and prepares cable terminations for connection to electrical equipment and overhead lines. Registration or licensing may be required.

Skill Level: 3

UNIT GROUP 3423 ELECTRONICS TRADES WORKERS

ELECTRONICS TRADES WORKERS maintain, adjust and repair electronic equipment such as commercial and office machines, video and audio equipment, and electronic instruments and control systems, and transmit and receive radio messages.

Indicative Skill Level:
Most occupations in this unit group have a level of skill commensurate with the qualifications and experience outlined below.

In Australia:
AQF Certificate III including at least two years of on-the-job training, or AQF Certificate IV (ANZSCO Skill Level 3)

In New Zealand:
NZ Register Level 4 qualification (ANZSCO Skill Level 3)

At least three years of relevant experience may substitute for the formal qualifications listed above. In some instances relevant experience and/or on-the-job training may be required in addition to the formal qualification.

Registration or licensing may be required.

Tasks Include:
- examining and testing machines, equipment, instruments and control systems to diagnose faults
- adjusting, repairing, and replacing worn and defective parts and wiring, and maintaining machines, equipment and instruments
- reassembling, test operating and adjusting equipment
- advising users of correct operating procedures to prevent malfunctions
- receiving messages by interpreting code and converting to plain language, and writing and typing messages for transmission
- monitoring radio traffic, and transmitting and receiving voice messages
- installing electronic instruments and control systems
- applying knowledge of electrical, electronic, mechanical, hydraulic and pneumatic principles in commissioning and maintaining control systems

Occupations:
342311 Business Machine Mechanic
342312 Communications Operator
342313 Electronic Equipment Trades Worker
342314 Electronic Instrument Trades Worker (General)
342315 Electronic Instrument Trades Worker (Special Class)

342311 BUSINESS MACHINE MECHANIC

Maintains, adjusts and repairs computers, photocopiers, fax machines, cash registers and other electronic commercial and office machines.

Skill Level: 3

UNIT GROUP 3423 ELECTRONICS TRADES WORKERS *continued*

342312 COMMUNICATIONS OPERATOR

Transmits and receives radio messages by use of morse code, voice and radio teletype.

Skill Level: 3

Specialisations:

- Communication Information Systems Sailor (Navy)
- Communications and Information Systems Controller (Air Force)
- Operator Specialist Communications (Army)

342313 ELECTRONIC EQUIPMENT TRADES WORKER

Maintains, adjusts and repairs radio and television receivers, and related audio and visual reproduction equipment such as video cameras, digital versatile disc players, compact disc players and video cassette recorders.

Skill Level: 3

342314 ELECTRONIC INSTRUMENT TRADES WORKER (GENERAL)

Alternative Title:

- Instrument and Control Service Person

Installs, modifies, maintains and repairs electronic instruments and control systems. Registration or licensing may be required.

Skill Level: 3

Specialisations:

- Communication Electronic Technician (Air Force)
- Electronic Technician (Navy)

342315 ELECTRONIC INSTRUMENT TRADES WORKER (SPECIAL CLASS)

Alternative Title:

- Industrial Measurement and Control Technician

Installs, modifies, maintains and repairs complex electronic instruments and control systems which involve a combination of electrical, electronic, mechanical, hydraulic and pneumatic principles. Registration or licensing may be required.

Skill Level: 3

UNIT GROUP 3424 TELECOMMUNICATIONS TRADES WORKERS

TELECOMMUNICATIONS TRADES WORKERS install, maintain and repair data transmission equipment, aerial lines, conduits, cables, radio antennae and telecommunications equipment and appliances.

Indicative Skill Level:
Most occupations in this unit group have a level of skill commensurate with the qualifications and experience outlined below.

In Australia:
AQF Certificate III including at least two years of on-the-job training, or AQF Certificate IV (ANZSCO Skill Level 3)

In New Zealand:
NZ Register Level 4 qualification (ANZSCO Skill Level 3)

At least three years of relevant experience may substitute for the formal qualifications listed above. In some instances relevant experience and/or relevant vendor certification may be required in addition to the formal qualification.

Tasks Include:
- examining drawings, specifications and work areas to determine positioning and connections for equipment to be installed
- locating faults in telecommunications equipment using instruments such as ohmmeters, voltmeters, ammeters and transmission measuring equipment
- attaching wires and cables to appliances
- adjusting, replacing and repairing faulty items, and testing equipment using electronic instruments
- installing cabling for telephone, radio, pay TV and computer transmission
- joining cables and sealing sheaths with lead and thermoplastic
- erecting, testing and maintaining aerial and underground wires and cables, and radio and mobile phone antennae
- installing telecommunications equipment and appliances such as telephones, switchboards and data transmission equipment

Occupations:
342411 Cabler (Data and Telecommunications)
342412 Telecommunications Cable Jointer
342413 Telecommunications Linesworker (Aus) / Telecommunications Line Mechanic (NZ)
342414 Telecommunications Technician

342411 CABLER (DATA AND TELECOMMUNICATIONS)

Installs internal telecommunications and data cabling, equipment and peripherals for computer networks, telephony, cable television and monitored security and fire alarms.

Skill Level: 3

UNIT GROUP 3424 TELECOMMUNICATIONS TRADES WORKERS *continued*

342412 TELECOMMUNICATIONS CABLE JOINTER

Joints, terminates and repairs copper and fibre optic telecommunications cables installed in underground pipes, trenches and overhead systems.

Skill Level: 3

Specialisations:
- Fibre Optic Cable Splicer
- Fibre Optics Jointer

342413 TELECOMMUNICATIONS LINESWORKER (AUS) / TELECOMMUNICATIONS LINE MECHANIC (NZ)

Installs, maintains and repairs external telecommunication equipment such as aerial lines, conduits and underground cables, radio and mobile phone antennae, and limited items of terminal equipment.

Skill Level: 3

Specialisation:
- Operator Bearer Systems (Army)

342414 TELECOMMUNICATIONS TECHNICIAN

Alternative Title:
- Communications Technician

Installs, maintains and repairs telecommunications equipment and appliances, such as telephones, mobile telephones, switchboards and data transmission equipment, in homes, businesses, telephone exchanges and other network sites.

Skill Level: 3

Specialisation:
- Technician Telecommunication Systems (Army)

SUB-MAJOR GROUP 35 FOOD TRADES WORKERS

FOOD TRADES WORKERS bake bread and pastry goods, prepare meat for sale, and plan, organise, prepare and cook food for dining and catering establishments.

Indicative Skill Level:
Most occupations in this sub-major group have a level of skill commensurate with the qualifications and experience outlined below.

In Australia:

AQF Associate Degree, Advanced Diploma or Diploma, or at least three years of relevant experience (ANZSCO Skill Level 2); or

AQF Certificate III including at least two years of on-the-job training, or AQF Certificate IV, or at least three years of relevant experience (ANZSCO Skill Level 3)

In New Zealand:

NZ Register Diploma, or at least three years of relevant experience (ANZSCO Skill Level 2); or

NZ Register Level 4 qualification, or at least three years of relevant experience (ANZSCO Skill Level 3)

In some instances relevant experience and/or on-the-job training may be required in addition to the formal qualification.

Tasks Include:

- checking the cleanliness and operation of equipment and premises before production runs to ensure compliance with occupational health and safety regulations
- planning menus, estimating food and labour costs, and ordering food supplies
- monitoring quality of food at all stages of preparation and presentation
- preparing meat for sale and baking bread, cakes and pastries
- preparing food and cooking using ovens, hotplates, grills and similar equipment
- portioning food, placing it in dishes, and adding gravies, sauces and garnishes

Occupations in this sub-major group are classified into the following minor group:

351 Food Trades Workers

MINOR GROUP 351 FOOD TRADES WORKERS

FOOD TRADES WORKERS bake bread and pastry goods, prepare meat for sale, and plan, organise, prepare and cook food for dining and catering establishments.

Indicative Skill Level:
Most occupations in this minor group have a level of skill commensurate with the qualifications and experience outlined below.

In Australia:
AQF Associate Degree, Advanced Diploma or Diploma, or at least three years of relevant experience (ANZSCO Skill Level 2); or
AQF Certificate III including at least two years of on-the-job training, or AQF Certificate IV, or at least three years of relevant experience (ANZSCO Skill Level 3)

In New Zealand:
NZ Register Diploma, or at least three years of relevant experience (ANZSCO Skill Level 2); or
NZ Register Level 4 qualification, or at least three years of relevant experience (ANZSCO Skill Level 3)

In some instances relevant experience and/or on-the-job training may be required in addition to the formal qualification.

Tasks Include:
- checking the cleanliness and operation of equipment and premises before production runs to ensure compliance with occupational health and safety regulations
- planning menus, estimating food and labour costs, and ordering food supplies
- monitoring quality of food at all stages of preparation and presentation
- preparing meat for sale and baking bread, cakes and pastries
- preparing food and cooking using ovens, hotplates, grills and similar equipment
- portioning food, placing it in dishes, and adding gravies, sauces and garnishes

Occupations in this minor group are classified into the following unit groups:
3511 Bakers and Pastrycooks
3512 Butchers and Smallgoods Makers
3513 Chefs
3514 Cooks

UNIT GROUP 3511 BAKERS AND PASTRYCOOKS

BAKERS AND PASTRYCOOKS prepare and bake bread loaves and rolls, buns, cakes, biscuits and pastry goods.

Indicative Skill Level:
Most occupations in this unit group have a level of skill commensurate with the qualifications and experience outlined below.

In Australia:
AQF Certificate III including at least two years of on-the-job training, or AQF Certificate IV (ANZSCO Skill Level 3)

In New Zealand:
NZ Register Level 4 qualification (ANZSCO Skill Level 3)

At least three years of relevant experience may substitute for the formal qualifications listed above. In some instances relevant experience and/or on-the-job training may be required in addition to the formal qualification.

Tasks Include:
- checking the cleanliness of equipment and operation of premises before production runs to ensure compliance with occupational health and safety regulations
- checking the quality of raw materials and weighing ingredients
- kneading, maturing, cutting, moulding, mixing and shaping dough and pastry goods
- preparing pastry fillings
- monitoring oven temperatures and product appearance to determine baking times
- coordinating the forming, loading, baking, unloading, de-panning and cooling of batches of bread, rolls and pastry products
- glazing buns and pastries, and decorating cakes with cream and icing
- operating machines which roll and mould dough and cut biscuits
- emptying, cleaning and greasing baking trays, tins and other cooking equipment

Occupations:
351111 Baker
351112 Pastrycook

351111 BAKER

Prepares and bakes bread loaves and rolls.

Skill Level: 3

Specialisation:
Doughmaker

351112 PASTRYCOOK

Prepares and bakes buns, cakes, biscuits and pastry goods.

Skill Level: 3

Specialisation:
Cake Decorator

UNIT GROUP 3512 BUTCHERS AND SMALLGOODS MAKERS

BUTCHERS AND SMALLGOODS MAKERS select, cut trim, prepare and arrange meat for sale and supply, operate meat and smallgoods processing machines, and manage the processes in the production of smallgoods.

Indicative Skill Level:
Most occupations in this unit group have a level of skill commensurate with the qualifications and experience outlined below.

In Australia:

AQF Certificate III including at least two years of on-the-job training, or AQF Certificate IV (ANZSCO Skill Level 3)

In New Zealand:

NZ Register Level 4 qualification (ANZSCO Skill Level 3)

At least three years of relevant experience may substitute for the formal qualifications listed above. In some instances relevant experience and/or on-the-job training may be required in addition to the formal qualification.

Tasks Include:

- preparing meat for sale by removing bones, trimming fat and cutting, mincing and grinding meat to shape and size for display or as ordered
- preparing crumbed cuts of meat, and marinating, seasoning and curing special cuts
- selecting and preparing meat to produce smallgoods
- operating machines to grind, mix, mince and tenderise meat
- making seasonings and pickles by mixing spices, salt and other ingredients
- operating sausage filling machines, smoking chambers, and cooking kettles and vats
- advising customers on the suitability and uses of cuts of meat
- may assist in menu planning and scheduling, and in estimating food production costs

Occupation:

351211 Butcher or Smallgoods Maker

351211 BUTCHER OR SMALLGOODS MAKER

Selects, cuts, trims, prepares and arranges meat for sale or supply, operates meat or smallgoods processing machines, or manages the processes in the production of smallgoods.

Skill Level: 3

UNIT GROUP 3513 CHEFS

CHEFS plan and organise the preparation and cooking of food in dining and catering establishments.

Cooks, Fast Food Cooks and Kitchenhands are excluded from this unit group. Cooks are included in Unit Group 3514 Cooks. Fast Food Cooks and Kitchenhands are included in Minor Group 851 Food Preparation Assistants.

Indicative Skill Level:
Most occupations in this unit group have a level of skill commensurate with the qualifications and experience outlined below.

In Australia:
AQF Associate Degree, Advanced Diploma or Diploma (ANZSCO Skill Level 2)

In New Zealand:
NZ Register Diploma (ANZSCO Skill Level 2)

At least three years of relevant experience may substitute for the formal qualifications listed above. In some instances relevant experience and/or on-the-job training may be required in addition to the formal qualification.

Tasks Include:
- planning menus, estimating food and labour costs, and ordering food supplies
- monitoring quality of dishes at all stages of preparation and presentation
- discussing food preparation issues with Managers, Dietitians and kitchen and waiting staff
- demonstrating techniques and advising on cooking procedures
- preparing and cooking food
- explaining and enforcing hygiene regulations
- may select and train staff
- may freeze and preserve foods

Occupation:
351311 Chef

351311 CHEF

Plans and organises the preparation and cooking of food in a dining or catering establishment.

Skill Level: 2

Specialisations:
Chef de Partie
Commis Chef
Demi Chef
Second Chef
Sous Chef

UNIT GROUP 3514 COOKS

COOKS prepare, season and cook food in dining and catering establishments.

Chefs, Fast Food Cooks and Kitchenhands are excluded from this unit group. Chefs are included in Unit Group 3513 Chefs. Fast Food Cooks and Kitchenhands are included in Minor Group 851 Food Preparation Assistants.

Indicative Skill Level:
Most occupations in this unit group have a level of skill commensurate with the qualifications and experience outlined below.

In Australia:
AQF Certificate III including at least two years of on-the-job training, or AQF Certificate IV (ANZSCO Skill Level 3)

In New Zealand:
NZ Register Level 4 qualification (ANZSCO Skill Level 3)

At least three years of relevant experience may substitute for the formal qualifications listed above. In some instances relevant experience and/or on-the-job training may be required in addition to the formal qualification.

Tasks Include:
- examining foodstuffs to ensure quality
- regulating temperatures of ovens, grills and other cooking equipment
- preparing and cooking food
- seasoning food during cooking
- portioning food, placing it on plates, and adding gravies, sauces and garnishes
- storing food in temperature controlled facilities
- preparing food to meet special dietary requirements
- may plan menus and estimate food requirements
- may train other kitchen staff and apprentices

Occupation:
351411 Cook

351411 COOK

Prepares, seasons and cooks food in a dining or catering establishment.

Skill Level: 3

SUB-MAJOR GROUP 36 SKILLED ANIMAL AND HORTICULTURAL WORKERS

SKILLED ANIMAL AND HORTICULTURAL WORKERS care for, groom, train and shear animals, assist Veterinarians, establish and maintain gardens, parks and surfaces used for sport, and prepare and sell floral arrangements and flowers.

Indicative Skill Level:
Most occupations in this sub-major group have a level of skill commensurate with the qualifications and experience outlined below.

In Australia:
AQF Certificate III including at least two years of on-the-job training, or AQF Certificate IV (ANZSCO Skill Level 3)

In New Zealand:
NZ Register Level 4 qualification (ANZSCO Skill Level 3)

At least three years of relevant experience may substitute for the formal qualifications listed above. In some instances relevant experience and/or on-the-job training may be required in addition to the formal qualification.

Tasks Include:
- caring for and grooming animals
- training animals to obey commands and perform in competitions
- shearing wool and hair from animals
- assisting Veterinarians to perform procedures and operations
- arranging supply and storage of flowers and selecting, trimming and arranging flowers and decorations
- planning, constructing and maintaining gardens, parks and surfaces used for sports
- selecting seeds, bulbs and cuttings, and planting them in beds, lawn areas and tubs

Occupations in this sub-major group are classified into the following minor groups:
361 Animal Attendants and Trainers, and Shearers
362 Horticultural Trades Workers

MINOR GROUP 361 ANIMAL ATTENDANTS AND TRAINERS, AND SHEARERS

ANIMAL ATTENDANTS AND TRAINERS, AND SHEARERS care for, groom, train and shear animals, and assist Veterinarians.

Indicative Skill Level:
Most occupations in this minor group have a level of skill commensurate with the qualifications and experience outlined below.

In Australia:
AQF Certificate III including at least two years of on-the-job training, or AQF Certificate IV (ANZSCO Skill Level 3)

In New Zealand:
NZ Register Level 4 qualification (ANZSCO Skill Level 3)

At least three years of relevant experience may substitute for the formal qualifications listed above. In some instances relevant experience and/or on-the-job training may be required in addition to the formal qualification.

Tasks Include:
- training animals to obey commands and perform in competitions
- bathing, cutting, combing, blow-drying and styling pets' coats, clipping their nails and cleaning their ears
- inspecting, preparing, cleaning, disinfecting and maintaining comfortable animal cages and enclosures
- filling water troughs and feeding animals according to their individual needs
- shearing wool and hair from animals
- assisting Veterinarians to perform procedures and operations
- maintaining animal health records and monitoring animal health conditions and recovery after operations

Occupations in this minor group are classified into the following unit groups:
3611 Animal Attendants and Trainers
3612 Shearers
3613 Veterinary Nurses

UNIT GROUP 3611 ANIMAL ATTENDANTS AND TRAINERS

ANIMAL ATTENDANTS AND TRAINERS train, feed, groom and care for animals.

Indicative Skill Level:
Most occupations in this unit group have a level of skill commensurate with the qualifications and experience outlined below.

In Australia:
AQF Certificate III including at least two years of on-the-job training, or AQF Certificate IV (ANZSCO Skill Level 3)

In New Zealand:
NZ Register Level 4 qualification (ANZSCO Skill Level 3)

At least three years of relevant experience may substitute for the formal qualifications listed above. In some instances relevant experience and/or on-the-job training may be required in addition to the formal qualification.

Registration or licensing may be required.

Tasks Include:
- teaching animals to obey verbal and non-verbal commands and addressing behavioural problems
- training animals to accept riders and pull vehicles
- training animals to perform in competitions
- bathing, cutting, combing, blow-drying and styling pets' coats, clipping their nails and cleaning their ears
- inspecting, preparing, cleaning, disinfecting and maintaining comfortable animal cages and enclosures
- transporting food, filling water troughs and feeding animals according to their individual needs
- maintaining animal health records, treating minor injuries and reporting serious conditions to Veterinarians
- exercising and playing with animals, answering visitor questions, and transferring animals between enclosures by leading or carrying them
- dusting and spraying insecticides on animals and immersing them in insecticide baths, to control insect pests

Occupations:
361111 Dog Trainer or Handler
361112 Horse Trainer
361113 Pet Groomer
361114 Zookeeper
361199 Animal Attendants and Trainers nec

361111 DOG HANDLER OR TRAINER

Teaches dogs to obey commands and undertake specific tasks.

Skill Level: 3

361112 HORSE TRAINER

Prepares horses for riding, breeding, racing, work, show or competitions. Registration or licensing may be required.

Skill Level: 3

Specialisation:
Horse Breaker

UNIT GROUP 3611 ANIMAL ATTENDANTS AND TRAINERS *continued*

361113 PET GROOMER

Washes, dries, brushes, combs, cuts and styles pets' coats, clips their nails and cleans their ears.

Skill Level: 3

361114 ZOOKEEPER

Feeds, provides water for and monitors the health of animals in zoos, aquaria and wildlife parks, cleans, fixes and maintains animal cages, and informs visitors about animals.

Skill Level: 3

Specialisation:
- Aquarist

361199 ANIMAL ATTENDANTS AND TRAINERS NEC

This occupation group covers Animal Attendants and Trainers not elsewhere classified.

Skill Level: 3

Occupations in this group include:
- Crutching Contractor
- Kennel Hand
- Muleser

UNIT GROUP 3612 SHEARERS

SHEARERS remove wool and hair from sheep, goats, alpacas and other animals.

Indicative Skill Level:
Most occupations in this unit group have a level of skill commensurate with the qualifications and experience outlined below.

In Australia:
AQF Certificate III including at least two years of on-the-job training, or AQF Certificate IV (ANZSCO Skill Level 3)

In New Zealand:
NZ Register Level 4 qualification (ANZSCO Skill Level 3)

At least three years of relevant experience may substitute for the formal qualifications listed above. In some instances relevant experience and/or on-the-job training may be required in addition to the formal qualification.

Tasks Include:
- selecting and preparing shearing equipment
- catching and positioning animals for shearing
- shearing and removing wool and hair from animals
- identifying contaminated fibre and injured, infected and diseased animals
- treating skin cuts
- returning shorn animals to let-out pens for counting and checking
- may service, maintain and repair shearing equipment
- may shear stud animals with hand shears or special combs

Occupation:
361211 Shearer

361211 SHEARER

Removes wool and hair from sheep, goats, alpacas and other animals.

Skill Level: 3

UNIT GROUP 3613 VETERINARY NURSES

VETERINARY NURSES care for animals under treatment and in temporary residence at veterinary facilities and assist Veterinarians to perform procedures and operations.

Indicative Skill Level:
Most occupations in this unit group have a level of skill commensurate with the qualifications and experience outlined below.

In Australia:
AQF Certificate III including at least two years of on-the-job training, or AQF Certificate IV (ANZSCO Skill Level 3)

In New Zealand:
NZ Register Level 4 qualification (ANZSCO Skill Level 3)

At least three years of relevant experience may substitute for the formal qualifications listed above. In some instances relevant experience and/or on-the-job training may be required in addition to the formal qualification.

Tasks Include:
- holding animals to allow examination and treatment by Veterinarians
- cleaning and sterilising examination tables and equipment
- preparing instruments and handing them to the Veterinarian
- assisting Veterinarians to administer anaesthetics and oxygen during operations
- placing animals in cages for recovery from operations and monitoring their condition
- giving medications to animals
- maintaining stock control and records
- providing animal care advice, and preparing, delivering, and reviewing animal care education programs
- may perform diagnostic laboratory tests
- may act as receptionist, accept payments and undertake clerical work

Occupation:
361311 Veterinary Nurse

361311 VETERINARY NURSE

Alternative Titles:
Animal Nurse
Veterinary Assistant

Cares for animals under treatment or in temporary residence at veterinary facilities and assists Veterinarians to perform procedures and operations.

Skill Level: 3

MINOR GROUP 362 HORTICULTURAL TRADES WORKERS

HORTICULTURAL TRADES WORKERS prepare and sell floral arrangements and flowers, establish and maintain gardens, parks and surfaces used for sport, and plant, cultivate and maintain plants and trees.

Indicative Skill Level:
Most occupations in this minor group have a level of skill commensurate with the qualifications and experience outlined below.

In Australia:
AQF Certificate III including at least two years of on-the-job training, or AQF Certificate IV (ANZSCO Skill Level 3)

In New Zealand:
NZ Register Level 4 qualification (ANZSCO Skill Level 3)

At least three years of relevant experience may substitute for the formal qualifications listed above. In some instances relevant experience and/or on-the-job training may be required in addition to the formal qualification.

Tasks Include:
- arranging supply and storage of flowers and selecting, trimming and arranging flowers and decorations
- preparing plans and drawings, selecting materials and plants, and scheduling landscape construction
- preparing soil, potting media, growing sites and seedbeds before planting seeds, bulbs and new turf
- maintaining parks, gardens, surfaces used for sport, and plants in nurseries
- applying pesticides, fertilisers and other chemicals to control pests, diseases, weeds and nutritional and environmental plant disorders
- examining trees to assess their condition and determine treatment

Occupations in this minor group are classified into the following unit groups:
3621 Florists
3622 Gardeners
3623 Greenkeepers
3624 Nurserypersons

UNIT GROUP 3621 FLORISTS

FLORISTS prepare and sell floral arrangements.

Indicative Skill Level:
Most occupations in this unit group have a level of skill commensurate with the qualifications and experience outlined below.

In Australia:
AQF Certificate III including at least two years of on-the-job training, or AQF Certificate IV (ANZSCO Skill Level 3)

In New Zealand:
NZ Register Level 4 qualification (ANZSCO Skill Level 3)

At least three years of relevant experience may substitute for the formal qualifications listed above. In some instances relevant experience and/or on-the-job training may be required in addition to the formal qualification.

Tasks Include:
- planning and designing floral arrangements
- arranging supply and storage of flowers, greenery, decorations and other items
- treating flowers to extend their life
- selecting, trimming and arranging flowers and other materials
- packing, wrapping, and attaching message cards to, and organising delivery of, completed arrangements
- serving customers and accepting payments
- advising customers on the selection of flowers and floral arrangements
- may decorate hotels, churches, halls and other facilities for special events

Occupation:
362111 Florist

362111 FLORIST

Prepares and sells floral arrangements.

Skill Level: 3

UNIT GROUP 3622 GARDENERS

GARDENERS plant, cultivate, maintain, plan and construct parks, gardens and landscapes, and inspect, diagnose and treat trees and shrubs.

Indicative Skill Level:
Most occupations in this unit group have a level of skill commensurate with the qualifications and experience outlined below.

In Australia:
AQF Certificate III including at least two years of on-the-job training, or AQF Certificate IV (ANZSCO Skill Level 3)

In New Zealand:
NZ Register Level 4 qualification (ANZSCO Skill Level 3)

At least three years of relevant experience may substitute for the formal qualifications listed above. In some instances relevant experience and/or on-the-job training may be required in addition to the formal qualification.

Tasks Include:
- preparing and maintaining seedbeds and growing sites
- propagating and planting trees, bushes, hedges, flowers and bulbs
- preparing lawn areas by spreading top soil and planting grass, and by laying instant turf
- maintaining planted and grassed areas by weeding, trimming, fertilising, watering and mowing
- pruning trees and hedges, and installing plant support and protection devices
- preparing plans and drawings, selecting materials and plants, and scheduling landscape construction
- setting out and installing hardscape and softscape structures
- constructing gravel and paved areas, walls, fences, pergolas, ponds, barbecues and garden furniture
- examining trees to assess their condition and determine treatment
- lopping limbs off trees and shaping branches using chain and handsaws
- spraying and dusting plants and trees to control insects and disease, and felling diseased trees

Occupations:
362211 Gardener (General)
362212 Arborist
362213 Landscape Gardener

362211 GARDENER (GENERAL)

Plants, cultivates and maintains parks and gardens.

Skill Level: 3

362212 ARBORIST

Alternative Title:
Tree Surgeon

Maintains and cares for trees and shrubs by lopping limbs and shaping branches, treating trees with fertilisers and insecticides, removing dead or decaying trees, and advising on general tree care.

Skill Level: 3

362213 LANDSCAPE GARDENER

Plans and constructs garden landscapes.

Skill Level: 3

UNIT GROUP 3623 GREENKEEPERS

GREENKEEPERS establish and maintain fine turf, grassed areas and synthetic surfaces used for sporting events.

Indicative Skill Level:
Most occupations in this unit group have a level of skill commensurate with the qualifications and experience outlined below.

In Australia:
AQF Certificate III including at least two years of on-the-job training, or AQF Certificate IV (ANZSCO Skill Level 3)

In New Zealand:
NZ Register Level 4 qualification (ANZSCO Skill Level 3)

At least three years of relevant experience may substitute for the formal qualifications listed above. In some instances relevant experience and/or on-the-job training may be required in addition to the formal qualification.

Tasks Include:

- preparing seedbeds for new turf
- establishing and maintaining turf by watering, over sowing or over seeding, and repairing green damage
- mowing, rolling and levelling turf
- pegging and marking out lines and logos, installing nets, posts and stumps, and placing other sports equipment on playing areas
- operating and maintaining hand and power driven equipment such as mowers, aerators, cultivators, corers and line marking equipment
- constructing cricket wickets, tennis courts, and bowling, croquet and golf greens
- replanting, repairing, aerating, fertilising and top dressing lawns
- installing and maintaining synthetic surfaces
- may maintain buildings, fences and surrounding gardens

Occupation:
362311 Greenkeeper

362311 GREENKEEPER

Alternative Title:
Turf Keeper

Establishes and maintains fine turf, grassed areas and synthetic surfaces used for sporting events.

Skill Level: 3

UNIT GROUP 3624 NURSERYPERSONS

NURSERYPERSONS propagate and cultivate trees, shrubs, and ornamental and flowering plants in plant nurseries.

Indicative Skill Level:
Most occupations in this unit group have a level of skill commensurate with the qualifications and experience outlined below.

In Australia:
AQF Certificate III including at least two years of on-the-job training, or AQF Certificate IV (ANZSCO Skill Level 3)

In New Zealand:
NZ Register Level 4 qualification (ANZSCO Skill Level 3)

At least three years of relevant experience may substitute for the formal qualifications listed above. In some instances relevant experience and/or on-the-job training may be required in addition to the formal qualification.

Tasks Include:
- preparing potting media and containers before planting
- selecting seeds, bulbs and cuttings, and planting them in beds, lawn areas and tubs
- budding and grafting vegetative material onto root stock
- watering plants manually and controlling automatic watering operations
- applying pesticides to control pests, diseases, weeds and nutritional and environmental plant disorders
- keeping records of soil mixtures, plantings, treatments, losses and yields
- selecting plants and packaging them for presentation and delivery
- advising customers on plant care and appropriate plants for local conditions
- may plan sales area layouts and visual merchandise presentation

Occupation:
362411 Nurseryperson

362411 NURSERYPERSON

Propagates and cultivates trees, shrubs, and ornamental and flowering plants in a plant nursery.

Skill Level: 3

Specialisation:
Plant Propagator

SUB-MAJOR GROUP 39 OTHER TECHNICIANS AND TRADES WORKERS

This sub-major group covers Technicians and Trades Workers not elsewhere classified.

It includes Hairdressers, Printing Trades Workers, Textile, Clothing and Footwear Trades Workers, and Wood Trades Workers.

Indicative Skill Level:
Most occupations in this sub-major group have a level of skill commensurate with the qualifications and experience outlined below.

In Australia:

AQF Associate Degree, Advanced Diploma or Diploma, or at least three years of relevant experience (ANZSCO Skill Level 2); or

AQF Certificate III including at least two years of on-the-job training, or AQF Certificate IV, or at least three years of relevant experience (ANZSCO Skill Level 3)

In New Zealand:

NZ Register Diploma, or at least three years of relevant experience (ANZSCO Skill Level 2); or

NZ Register Level 4 qualification, or at least three years of relevant experience (ANZSCO Skill Level 3)

In some instances relevant experience and/or on-the-job training may be required in addition to the formal qualification.

Occupations in this sub-major group are classified into the following minor groups:

391 Hairdressers
392 Printing Trades Workers
393 Textile, Clothing and Footwear Trades Workers
394 Wood Trades Workers
399 Miscellaneous Technicians and Trades Workers

MINOR GROUP 391 HAIRDRESSERS

HAIRDRESSERS cut, style, colour, straighten and permanently wave hair, and treat hair and scalp conditions.

Indicative Skill Level:
Most occupations in this minor group have a level of skill commensurate with the qualifications and experience outlined below.

In Australia:
AQF Certificate III including at least two years of on-the-job training, or AQF Certificate IV (ANZSCO Skill Level 3)

In New Zealand:
NZ Register Level 4 qualification (ANZSCO Skill Level 3)

At least three years of relevant experience may substitute for the formal qualifications listed above. In some instances relevant experience and/or on-the-job training may be required in addition to the formal qualification.

Tasks Include:
- providing advice on hair care, beauty products and hairstyles
- shampooing hair and conditioning scalps
- colouring, straightening and permanently waving hair with chemical solutions
- cutting hair with scissors, clippers and razors
- styling hair into dreadlocks and braids and adding hair extensions
- shaving and trimming beards and moustaches
- cleaning work areas and sanitising instruments
- arranging appointments and collecting payments
- may clean, colour, cut and style wigs and hairpieces

Occupations in this minor group are classified into the following unit group:
3911 Hairdressers

UNIT GROUP 3911 HAIRDRESSERS

HAIRDRESSERS cut, style, colour, straighten and permanently wave hair, and treat hair and scalp conditions.

Indicative Skill Level:
Most occupations in this unit group have a level of skill commensurate with the qualifications and experience outlined below.

In Australia:
AQF Certificate III including at least two years of on-the-job training, or AQF Certificate IV (ANZSCO Skill Level 3)

In New Zealand:
NZ Register Level 4 qualification (ANZSCO Skill Level 3)

At least three years of relevant experience may substitute for the formal qualifications listed above. In some instances relevant experience and/or on-the-job training may be required in addition to the formal qualification.

Tasks Include:

- providing advice on hair care, beauty products and hairstyles
- shampooing hair and conditioning scalps
- colouring, straightening and permanently waving hair with chemical solutions
- cutting hair with scissors, clippers and razors
- styling hair into dreadlocks and braids and adding hair extensions
- shaving and trimming beards and moustaches
- cleaning work areas and sanitising instruments
- arranging appointments and collecting payments
- may clean, colour, cut and style wigs and hairpieces

Occupation:
391111 Hairdresser

391111 HAIRDRESSER

Cuts, styles, colours, straightens and permanently waves hair, and treats hair and scalp conditions.

Skill Level: 3

Specialisation:
Barber

MINOR GROUP 392 PRINTING TRADES WORKERS

PRINTING TRADES WORKERS compose and set type prior to printing, set up and operate printing presses, bind and finish printed products, and prepare stencils and operate screen printing equipment.

Indicative Skill Level:
Most occupations in this minor group have a level of skill commensurate with the qualifications and experience outlined below.

In Australia:
AQF Certificate III including at least two years of on-the-job training, or AQF Certificate IV (ANZSCO Skill Level 3)

In New Zealand:
NZ Register Level 4 qualification (ANZSCO Skill Level 3)

At least three years of relevant experience may substitute for the formal qualifications listed above. In some instances relevant experience and/or on-the-job training may be required in addition to the formal qualification.

Tasks Include:
- operating graphic cameras and other photographic equipment to reproduce camera-ready copy onto films, plates and digital output devices
- operating computer screen-based equipment for scanning, colour separation and correction, retouching and other processes used to transfer copy to film and produce film for plate, cylinder and digital output productions
- setting up, operating and monitoring machines used in typesetting, photographing copy, printing and cutting, folding, collating and binding printed material
- performing routine finishing operations and machine maintenance
- preparing stencils and operating screen printing equipment

Occupations in this minor group are classified into the following unit groups:
3921 Binders, Finishers and Screen Printers
3922 Graphic Pre-press Trades Workers
3923 Printers

UNIT GROUP 3921 BINDERS, FINISHERS AND SCREEN PRINTERS

BINDERS, FINISHERS AND SCREEN PRINTERS bind books and other publications, finish printed products by hand and machine, prepare stencils, and set up and operate power-driven and hand-operated screen print equipment.

Indicative Skill Level:
Most occupations in this unit group have a level of skill commensurate with the qualifications and experience outlined below.

In Australia:
AQF Certificate III including at least two years of on-the-job training, or AQF Certificate IV (ANZSCO Skill Level 3)

In New Zealand:
NZ Register Level 4 qualification (ANZSCO Skill Level 3)

At least three years of relevant experience may substitute for the formal qualifications listed above. In some instances relevant experience and/or on-the-job training may be required in addition to the formal qualification.

Tasks Include:
- setting up and supervising the operation of automatic binding and finishing equipment
- binding full, half and limp-bound books, and repairing bindings
- folding, collating and sewing signatures by machine and hand
- operating paper guillotines for pre-press and post-press paper cutting and trimming, and programming electronically operated units
- operating systems to insert printed material into newspapers, magazines and envelopes
- embellishing printed products automatically and manually
- operating photographic and electronic reproduction devices
- preparing stencils using computer and hand-cut methods
- selecting, mixing and matching coloured inks and loading into screen printing presses
- loading printed items into drying racks, and unloading and stacking dry items

Occupations:
392111 Binder and Finisher
392112 Screen Printer

392111 BINDER AND FINISHER

Alternative Title:
Print Finisher

Binds books and other publications, and finishes printed products by hand or machine.

Skill Level: 3

Specialisations:
Mailhouse Operator (Aus)
Paper Guillotine Operator (Bookbinding)

UNIT GROUP 3921 BINDERS, FINISHERS AND SCREEN PRINTERS *continued*

392112 SCREEN PRINTER

Prepares stencils, and sets up and operates power-driven or hand-operated screen print equipment.

Skill Level: 3

Specialisations:
- Screen Printing Stencil Preparer
- Textiles Printer

UNIT GROUP 3922 GRAPHIC PRE-PRESS TRADES WORKERS

GRAPHIC PRE-PRESS TRADES WORKERS manipulate, set and compose text and graphics into a format suitable for printing and other visual media.

Indicative Skill Level:
Most occupations in this unit group have a level of skill commensurate with the qualifications and experience outlined below.

In Australia:
AQF Certificate III including at least two years of on-the-job training, or AQF Certificate IV (ANZSCO Skill Level 3)

In New Zealand:
NZ Register Level 4 qualification (ANZSCO Skill Level 3)

At least three years of relevant experience may substitute for the formal qualifications listed above. In some instances relevant experience and/or on-the-job training may be required in addition to the formal qualification.

Tasks Include:
- operating graphic cameras and other photographic equipment to reproduce camera-ready copy onto films, plates and digital output devices
- using computer applications to generate images, text, layouts and impositions for print and other visual media displays
- operating plate making equipment to reproduce images from film to printing plates, digital output devices and presses
- operating computer screen-based equipment for scanning, colour separation, colour correction, masking, creative design, combining, imposing, retouching, and other processes used to transfer copy to film and produce film for plate, digital output and cylinder productions
- carrying out digital and chemical proofing from digital systems, and negative and positive films
- evaluating printed proofs, checking and correcting them for quality
- preparing and exposing carbon tissue for laying on cylinders by transfer method, and developing images

Occupation:
392211 Graphic Pre-press Trades Worker

392211 GRAPHIC PRE-PRESS TRADES WORKER

Manipulates, sets and composes text and graphics into a format suitable for printing and other visual media.

Skill Level: 3

Specialisation:
Desktop Publishing Operator

UNIT GROUP 3923 PRINTERS

PRINTERS set up and operate letterpress, lithographic, flexographic, gravure, newspaper, instant, digital and offset printing presses.

Indicative Skill Level:
Most occupations in this unit group have a level of skill commensurate with the qualifications and experience outlined below.

In Australia:

AQF Certificate III including at least two years of on-the-job training, or AQF Certificate IV (ANZSCO Skill Level 3)

In New Zealand:

NZ Register Level 4 qualification (ANZSCO Skill Level 3)

At least three years of relevant experience may substitute for the formal qualifications listed above. In some instances relevant experience and/or on-the-job training may be required in addition to the formal qualification.

Tasks Include:

- setting, adjusting and monitoring substrate-feed mechanisms, delivery mechanisms, inking systems and other printing machine functions
- mixing ink and solvents to standard, and regulating paper and ink supply during print runs
- monitoring, evaluating and determining press operations manually and by computer to check print quality standards against proofs and detect malfunctions
- producing a variety of printed products using relief, lithographic, flexographic and gravure printing presses, and in-line finishing systems
- preparing plates, blankets and impression cylinders on small offset lithographic printing presses
- loading paper into feeding mechanisms
- monitoring machine operations and quality of printing
- undertaking maintenance, adjustment, repair and cleaning of machines
- producing and managing digital print images, and transferring and outputting images
- may set up and operate paper and bookbinding guillotines

Occupations:

392311 Printing Machinist
392312 Small Offset Printer

392311 PRINTING MACHINIST

Produces books, magazines, newspapers, brochures, posters, leaflets, packaging materials and stationery using printing presses.

Skill Level: 3

Specialisations:

Flexographic Printing Machinist
Gravure Printing Machinist
Label Printing Machinist
Letterpress Printing Machinist
Lithographic Printing Machinist
Reel Fed Printer
Sheet Fed Printer

UNIT GROUP 3923 PRINTERS *continued*

392312 SMALL OFFSET PRINTER

Alternative Title:

Instant Printer Operator

Sets up and operates small offset printing presses used in instant print shops or for in-house printing.

Skill Level: 3

Specialisation:

Digital Printer

MINOR GROUP 393 TEXTILE, CLOTHING AND FOOTWEAR TRADES WORKERS

TEXTILE, CLOTHING AND FOOTWEAR TRADES WORKERS prepare patterns and materials for fabricating and repairing garments, shoes, covers for furniture and other fabric, leather and canvas goods.

Indicative Skill Level:
Most occupations in this minor group have a level of skill commensurate with the qualifications and experience outlined below.

In Australia:

AQF Certificate III including at least two years of on-the-job training, or AQF Certificate IV (ANZSCO Skill Level 3)

In New Zealand:

NZ Register Level 4 qualification (ANZSCO Skill Level 3)

At least three years of relevant experience may substitute for the formal qualifications listed above. In some instances relevant experience and/or on-the-job training may be required in addition to the formal qualification.

Tasks Include:

- conferring with clients to determine styles, designs and articles to be fabricated
- cutting out master patterns
- cutting and preparing leather, canvas and sailcloth
- sewing, gluing and riveting leather and canvas
- lasting shoes and providing modifications and repairs to footwear
- sewing, fitting and altering garments
- measuring, cutting and covering furniture with materials
- padding and covering spring units to upholster mattresses

Occupations in this minor group are classified into the following unit groups:

3931 Canvas and Leather Goods Makers
3932 Clothing Trades Workers
3933 Upholsterers

UNIT GROUP 3931 CANVAS AND LEATHER GOODS MAKERS

CANVAS AND LEATHER GOODS MAKERS make and repair boots, shoes, leather goods, canvas and sailcloth articles, and related products.

Indicative Skill Level:
Most occupations in this unit group have a level of skill commensurate with the qualifications and experience outlined below.

In Australia:
AQF Certificate III including at least two years of on-the-job training, or AQF Certificate IV (ANZSCO Skill Level 3)

In New Zealand:
NZ Register Level 4 qualification (ANZSCO Skill Level 3)

At least three years of relevant experience may substitute for the formal qualifications listed above. In some instances relevant experience and/or on-the-job training may be required in addition to the formal qualification.

Tasks Include:
- cutting and preparing canvas, leather and sailcloth to design specifications, patterns and drawings
- sewing, gluing and riveting sections of canvas together to make articles such as awnings, tents, tarpaulins and horse rugs
- attaching grommets, fastenings and other fittings to canvas goods
- joining parts of leather articles using rivets, hand sewing, sewing machines, tools and adhesive
- restoring and repairing leather articles
- fabricating sails
- designing patterns and prototypes of boots and shoes
- making and grading patterns using manual and computerised methods
- clicking synthetics, corrected grains, leather linings and leather outers by hand and machine
- altering and repairing footwear

Occupations:
393111 Canvas Goods Maker
393112 Leather Goods Maker
393113 Sail Maker
393114 Shoemaker

393111 CANVAS GOODS MAKER

Fabricates and repairs canvas and related products such as awnings, tents, tarpaulins, horse rugs and caravan annexes.

Skill Level: 3

393112 LEATHER GOODS MAKER

Fabricates and repairs leather articles such as wallets, cases, harnesses and saddlery.

Skill Level: 3

Specialisation:
Saddler

UNIT GROUP 3931 CANVAS AND LEATHER GOODS MAKERS *continued*

393113 SAIL MAKER

Fabricates and repairs sails and other articles from sailcloth.

Skill Level: 3

393114 SHOEMAKER

Makes and repairs boots or shoes.

Skill Level: 3

Specialisations:
- Medical Grade Shoemaker
- Shoe Repairer

UNIT GROUP 3932 CLOTHING TRADES WORKERS

CLOTHING TRADES WORKERS prepare and cut garment patterns and fabric, and make and repair garments.

Indicative Skill Level:
Most occupations in this unit group have a level of skill commensurate with the qualifications and experience outlined below.

In Australia:
AQF Certificate III including at least two years of on-the-job training, or AQF Certificate IV (ANZSCO Skill Level 3)

In New Zealand:
NZ Register Level 4 qualification (ANZSCO Skill Level 3)

At least three years of relevant experience may substitute for the formal qualifications listed above. In some instances relevant experience and/or on-the-job training may be required in addition to the formal qualification.

Tasks Include:

- conferring with customers to determine material, styles and designs of garments
- interpreting designs, sketches and samples to determine pattern specifications
- cutting out master patterns
- laying up and cutting fabric
- pinning, basting and draping garment parts
- sewing garments
- fitting basted garments on customers and marking areas requiring alteration
- sewing buttonholes, and sewing on buttons, hooks, eyes and press fasteners to finish garments
- pressing and finishing work

Occupations:
393211 Apparel Cutter
393212 Clothing Patternmaker
393213 Dressmaker or Tailor
393299 Clothing Trades Workers nec

393211 APPAREL CUTTER

Lays out, marks and cuts fabric to form parts of garments.

Skill Level: 3

393212 CLOTHING PATTERNMAKER

Draws sets of master patterns following sketches, sample articles and design specifications, and cuts out patterns for garments.

Skill Level: 3

Specialisations:
Pattern Grader (Clothing)
Patternmaker-Grader

UNIT GROUP 3932 CLOTHING TRADES WORKERS *continued*

393213 DRESSMAKER OR TAILOR

Makes, alters and repairs women's and men's tailored garments, formal wear, couturier clothing, and special occasion wear such as suits, dresses, coats, evening wear and bridal wear.

Skill Level: 3

Specialisations:
- Costume Maker
- Wardrobe Assistant
- Wardrobe Coordinator

393299 CLOTHING TRADES WORKERS NEC

This occupation group covers Clothing Trades Workers not elsewhere classified.

Skill Level: 3

Occupations in this group include:
- Fur Cutter
- Fur Matcher
- Furrier
- Milliner

UNIT GROUP 3933 UPHOLSTERERS

UPHOLSTERERS make, rebuild and repair upholstered articles such as chairs, sofas, beds and mattresses.

Indicative Skill Level:
Most occupations in this unit group have a level of skill commensurate with the qualifications and experience outlined below.

In Australia:
AQF Certificate III including at least two years of on-the-job training, or AQF Certificate IV (ANZSCO Skill Level 3)

In New Zealand:
NZ Register Level 4 qualification (ANZSCO Skill Level 3)

At least three years of relevant experience may substitute for the formal qualifications listed above. In some instances relevant experience and/or on-the-job training may be required in addition to the formal qualification.

Tasks Include:
- conferring with clients to determine materials and cost of furniture items to be made or repaired
- making and repairing wooden frames, and removing and replacing defective springs
- removing coverings, webbing and padding from old furniture
- securing material, padding, springs and webbing to articles to be upholstered
- measuring and cutting materials and covering furniture
- seaming cushions and joining sections of covering material
- attaching ornamental trims, braids and buttons
- padding and covering spring units to upholster mattresses
- may finish wooden surfaces on furniture
- may remove stains from fabric

Occupation:
393311 Upholsterer

393311 UPHOLSTERER

Makes, rebuilds and repairs upholstered articles such as chairs, sofas, beds and mattresses.

Skill Level: 3

Specialisations:
Furniture Upholsterer
Mattress Maker

MINOR GROUP 394 WOOD TRADES WORKERS

WOOD TRADES WORKERS fabricate, repair and finish wooden furniture and fit and assemble prepared wooden parts to make furniture, set up and operate woodworking machines and wood turning lathes to shape wood stock, and make picture frames and other wood products.

Indicative Skill Level:
Most occupations in this minor group have a level of skill commensurate with the qualifications and experience outlined below.

In Australia:
AQF Certificate III including at least two years of on-the-job training, or AQF Certificate IV (ANZSCO Skill Level 3)

In New Zealand:
NZ Register Level 4 qualification (ANZSCO Skill Level 3)

At least three years of relevant experience may substitute for the formal qualifications listed above. In some instances relevant experience and/or on-the-job training may be required in addition to the formal qualification.

Tasks Include:
- examining drawings, work orders and sample parts to determine specifications
- selecting and working with materials such as timber, veneers, particle board and synthetic wood
- marking out, cutting and shaping wood using tools ranging from hand tools to large timber cutting machines
- determining tooling and machine requirements
- assembling parts to form sections of furniture and completed articles
- repairing and finishing furniture
- fitting hinges, locks, catches, drawers and shelves
- making picture frames and other wood products.

Occupations in this minor group are classified into the following unit groups:
3941 Cabinetmakers
3942 Wood Machinists and Other Wood Trades Workers

UNIT GROUP 3941 CABINETMAKERS

CABINETMAKERS fabricate and repair wooden furniture, and fit and assemble prepared wooden parts to make furniture.

Indicative Skill Level:
Most occupations in this unit group have a level of skill commensurate with the qualifications and experience outlined below.

In Australia:
AQF Certificate III including at least two years of on-the-job training, or AQF Certificate IV (ANZSCO Skill Level 3)

In New Zealand:
NZ Register Level 4 qualification (ANZSCO Skill Level 3)

At least three years of relevant experience may substitute for the formal qualifications listed above. In some instances relevant experience and/or on-the-job training may be required in addition to the formal qualification.

Tasks Include:
- examining drawings, work orders and sample parts to determine specifications
- selecting and working with materials such as timber, veneers, particle board and synthetic wood
- marking out, cutting and shaping wood
- working from drawings and specifications to make furniture
- making fittings for boats, caravans and other items where fine detail is required
- assembling parts to form sections of furniture and completed articles
- fitting hinges, locks, catches, drawers and shelves
- making frames for chairs and couches
- may repair and refurbish furniture and antiques

Occupation:
394111 Cabinetmaker

394111 CABINETMAKER

Fabricates or repairs wooden furniture, and fits and assembles prepared wooden parts to make furniture.

Skill Level: 3

Specialisations:
Antique Furniture Reproducer
Antique Furniture Restorer
Chair and Couch Maker
Coffin Maker

UNIT GROUP 3942 WOOD MACHINISTS AND OTHER WOOD TRADES WORKERS

WOOD MACHINISTS AND OTHER WOOD TRADES WORKERS set up and operate woodworking machines and wood turning lathes to shape wood stock, finish and polish furniture, and make picture frames and frame paintings, photographs and other artwork.

Indicative Skill Level:
Most occupations in this unit group have a level of skill commensurate with the qualifications and experience outlined below.

In Australia:
AQF Certificate III including at least two years of on-the-job training, or AQF Certificate IV (ANZSCO Skill Level 3)

In New Zealand:
NZ Register Level 4 qualification (ANZSCO Skill Level 3)

At least three years of relevant experience may substitute for the formal qualifications listed above. In some instances relevant experience and/or on-the-job training may be required in addition to the formal qualification.

Tasks Include:
- studying drawings, work orders and sample parts to determine specifications
- determining tooling and machine requirements and sequence of operations
- setting up woodworking machines and wood stock for correct cutting, planning, turning, shaping and sanding
- operating machines to cut, plane, turn, shape and sand work pieces
- removing old finishes by stripping with steel wool and glasspaper, and by applying solvents and paint strippers, and removing softened finishes by scraping
- applying varnish, shellac, lacquer, stains and paint to surfaces and polishing and waxing finished surfaces
- fitting and fastening frame pieces
- mounting backing materials and subjects for framing

Occupations:
394211 Furniture Finisher
394212 Picture Framer
394213 Wood Machinist
394214 Wood Turner
394299 Wood Machinists and Other Wood Trades Workers nec

394211 FURNITURE FINISHER

Applies finishes, such as stain, lacquer, paint, oil and varnish, to furniture, and polishes and waxes finished furniture surfaces.

Skill Level: 3

Specialisation:
French Polisher

394212 PICTURE FRAMER

Cuts out and assembles mouldings to make picture frames, and frames paintings, photographs, needlework and other artwork.

Skill Level: 3

UNIT GROUP 3942 WOOD MACHINISTS AND OTHER WOOD TRADES WORKERS *continued*

394213 WOOD MACHINIST

Cuts, planes, turns, shapes and sands wood stock to specifications.

Skill Level: 3

Specialisations:

- Automatic Profile Sander Operator
- Copy Lathe Operator
- Edge Bander Operator
- Jigmaker (Wood)
- Panel Saw Operator
- Woodworking Machine Setter

394214 WOOD TURNER

Operates wood turning lathes to turn and shape wood stock.

Skill Level: 3

394299 WOOD MACHINISTS AND OTHER WOOD TRADES WORKERS NEC

This occupation group covers Wood Machinists and Wood Trades Workers not elsewhere classified.

Skill Level: 3

Occupations in this group include:

- Cane Furniture Maker
- Cooper
- Wood Model Maker

MINOR GROUP 399 MISCELLANEOUS TECHNICIANS AND TRADES WORKERS

This minor group covers Technicians and Trades Workers not elsewhere classified.

It includes Boat Builders and Shipwrights; Chemical, Gas Petroleum and Power Generation Plant Operators; Gallery, Library and Museum Technicians; Jewellers; Performing Arts Technicians; and Signwriters.

Indicative Skill Level:
Most occupations in this minor group have a level of skill commensurate with the qualifications and experience outlined below.

In Australia:
- AQF Associate Degree, Advanced Diploma or Diploma, or at least three years of relevant experience (ANZSCO Skill Level 2); or
- AQF Certificate III including at least two years of on-the-job training, or AQF Certificate IV, or at least three years of relevant experience (ANZSCO Skill Level 3)

In New Zealand:
- NZ Register Diploma, or at least three years of relevant experience (ANZSCO Skill Level 2); or
- NZ Register Level 4 qualification, or at least three years of relevant experience (ANZSCO Skill Level 3)

In some instances relevant experience and/or on-the-job training may be required in addition to the formal qualification.

Occupations in this minor group are classified into the following unit groups:
- 3991 Boat Builders and Shipwrights
- 3992 Chemical, Gas, Petroleum and Power Generation Plant Operators
- 3993 Gallery, Library and Museum Technicians
- 3994 Jewellers
- 3995 Performing Arts Technicians
- 3996 Signwriters
- 3999 Other Miscellaneous Technicians and Trades Workers

UNIT GROUP 3991 BOAT BUILDERS AND SHIPWRIGHTS

BOAT BUILDERS AND SHIPWRIGHTS construct, fit out and repair boats and ships.

Indicative Skill Level:
Most occupations in this unit group have a level of skill commensurate with the qualifications and experience outlined below.

In Australia:
AQF Certificate III including at least two years of on-the-job training, or AQF Certificate IV (ANZSCO Skill Level 3)

In New Zealand:
NZ Register Level 4 qualification (ANZSCO Skill Level 3)

At least three years of relevant experience may substitute for the formal qualifications listed above. In some instances relevant experience and/or on-the-job training may be required in addition to the formal qualification.

Registration or licensing may be required.

Tasks Include:
- studying plans and specifications, and preparing templates and scale plans for fabrication and cutting of hull sections
- marking reference points and lines on dry docks and slipways
- checking position and functioning of slipway apparatus
- assembling shells of boats and erecting hull sections of ship
- erecting and preparing launching platforms, conducting pre-launch tests and supervising launching procedures
- installing masts, frames, decking, fittings, machines, shafts and safety equipment
- building and installing structures such as cabins, machine mountings, propeller supports and rudders
- determining repair requirements and procedures
- may make hull moulds and fabricate and repair vessels using materials such as aluminium, wood, glass, reinforced plastics, carbon fibre, Kevlar, fibreglass and concrete

Occupations:
399111 Boat Builder and Repairer
399112 Shipwright

399111 BOAT BUILDER AND REPAIRER

Builds, repairs and modifies boats. Registration or licensing may be required.

Skill Level: 3

Specialisations:
Composite Boat Builder
Rigger (Boat)
Sparmaker
Wooden Boat Builder
Yacht Builder

UNIT GROUP 3991 BOAT BUILDERS AND SHIPWRIGHTS *continued*

399112 SHIPWRIGHT

Constructs, fits out and repairs ships. Registration or licensing is required.

Skill Level: 3

Specialisations:
- Loftsman/woman (Marine)
- Ship's Carpenter

UNIT GROUP 3992 CHEMICAL, GAS, PETROLEUM AND POWER GENERATION PLANT OPERATORS

CHEMICAL, GAS, PETROLEUM AND POWER PLANT OPERATORS control the operation of chemical production equipment, pump gas and oil from wellheads, refine and process petroleum products, and operate boilers, turbogenerators and associated plant to generate electrical power.

Indicative Skill Level:
Most occupations in this unit group have a level of skill commensurate with the qualifications and experience outlined below.

In Australia:
AQF Certificate III including at least two years of on-the-job training, or AQF Certificate IV (ANZSCO Skill Level 3)

In New Zealand:
NZ Register Level 4 qualification (ANZSCO Skill Level 3)

At least three years of relevant experience may substitute for the formal qualifications listed above. In some instances relevant experience and/or on-the-job training may be required in addition to the formal qualification.

Registration or licensing may be required.

Tasks Include:
- controlling equipment performing continuous and batch processes to process chemicals and natural gas, manufacture refined petroleum products, and blend petroleum base stocks to produce commercial fuels, lubricating oils and asphalt
- controlling the preparation, measuring and feeding of raw material and processing agents such as catalysts and filtering media into plant
- patrolling and inspecting equipment to ensure proper operation and setting operating controls on equipment
- analysing samples and readings and recording test data
- controlling records of production, quantities transferred and details of blending and pumping operations
- checking equipment for malfunctions and arranging maintenance
- operating power generation plant controls to produce required load
- monitoring operation of power generation plant and interpreting instrument readings
- authorising procedures to isolate high-voltage and low-voltage electrical apparatus and plant
- writing reports and maintaining records on equipment performance, instrument readings and switching operations
- carrying out routine operating tests

Occupations:
399211 Chemical Plant Operator
399212 Gas or Petroleum Operator
399213 Power Generation Plant Operator

UNIT GROUP 3992 CHEMICAL, GAS, PETROLEUM AND POWER GENERATION PLANT OPERATORS *continued*

399211 CHEMICAL PLANT OPERATOR

Controls the operation of chemical production plant.

Skill Level: 3

Specialisations:

- Chemicals Distiller
- Chemicals Fermentation Operator
- Industrial Gas Production Operator
- Paint Maker
- Pharmaceutical Plant Operator
- Pilot Plant Operator

399212 GAS OR PETROLEUM OPERATOR

Alternative Titles:

- Oil and Gas Well Treatment Operator
- Oil, Gas and Pipe Tester
- Petroleum and Gas Refining and Pumping Operator

Operates equipment to pump oil and gas from wellheads, and refine and process petroleum products.

Skill Level: 3

Specialisations:

- Gas Compressor Turbine Operator
- Petroleum Blending Plant Operator
- Petroleum Terminal Plant Operator
- Refinery Pipeline Operator

399213 POWER GENERATION PLANT OPERATOR

Operates boilers, turbogenerators and associated plant to generate electrical power. Registration or licensing is required.

Skill Level: 3

Specialisations:

- Hydro-electric Station Operator
- Power Generation Turbine Room Operator

UNIT GROUP 3993 GALLERY, LIBRARY AND MUSEUM TECHNICIANS

GALLERY, LIBRARY AND MUSEUM TECHNICIANS prepare artworks, specimens and artefacts for collections, arrange and construct gallery exhibits, and assist Librarians to organise and operate systems for handling recorded material and files.

Indicative Skill Level:
Most occupations in this unit group have a level of skill commensurate with the qualifications and experience outlined below.

In Australia:
AQF Associate Degree, Advanced Diploma or Diploma (ANZSCO Skill Level 2)

In New Zealand:
NZ Register Diploma (ANZSCO Skill Level 2)

At least three years of relevant experience may substitute for the formal qualifications listed above. In some instances relevant experience and/or on-the-job training may be required in addition to the formal qualification.

Tasks Include:

- mounting and preparing objects for display
- designing and arranging exhibit furnishings, display cases and display areas
- assisting in setting up lighting and display equipment
- receiving, shipping, packing and unpacking exhibits
- ordering new library materials and maintaining library records and circulation systems
- cataloguing printed and recorded material
- entering data into databases and editing computer records
- operating audiovisual and reprographic equipment
- searching and verifying bibliographic data

Occupations:
399311 Gallery or Museum Technician
399312 Library Technician

399311 GALLERY OR MUSEUM TECHNICIAN

Prepares artworks, specimens and artefacts for collections, and arranges and constructs gallery or museum exhibits.

Skill Level: 2

Specialisation:
Taxidermist

399312 LIBRARY TECHNICIAN

Assists Librarians and other information managers in organising and operating systems for handling recorded material and files.

Skill Level: 2

UNIT GROUP 3994 JEWELLERS

JEWELLERS make and repair jewellery such as rings, brooches, chains and bracelets, craft objects out of precious metals, and cut, shape and polish rough gemstones to produce fashion and industrial jewels.

Indicative Skill Level:
Most occupations in this unit group have a level of skill commensurate with the qualifications and experience outlined below.

In Australia:
AQF Certificate III including at least two years of on-the-job training, or AQF Certificate IV (ANZSCO Skill Level 3)

In New Zealand:
NZ Register Level 4 qualification (ANZSCO Skill Level 3)

At least three years of relevant experience may substitute for the formal qualifications listed above. In some instances relevant experience and/or on-the-job training may be required in addition to the formal qualification.

Tasks Include:

- examining designs and specifications for jewellery and precious metal objects
- shaping moulded metal by cutting, filing, beating, turning and bending, using specialised hand and power tools
- assembling articles by soldering, screwing, riveting and otherwise joining
- securing precious stones in retaining prongs and ridges, and smoothing and checking final settings
- engraving designs on ring settings, brooches, bracelets and other articles
- repairing jewellery by soldering, replacing and rebuilding worn and broken parts
- appraising the quality and value of jewellery
- cutting and dividing stones to approximate final shape, using precision hand and power tools and jigs
- securing stones and shapes, cutting angles, smoothing and polishing
- finishing articles using files, emery paper and buffing machines
- restyling old jewellery

Occupation:
399411 Jeweller

399411 JEWELLER

Makes and repairs jewellery such as rings, brooches, chains and bracelets, crafts objects out of precious metals, or cuts, shapes and polishes rough gemstones to produce fashion or industrial jewels.

Skill Level: 3

Specialisations:
Diamond Cutter
Faceter
Gem Setter
Goldsmith
Lapidary
Opal Polisher
Ring Maker
Silversmith

UNIT GROUP 3995 PERFORMING ARTS TECHNICIANS

PERFORMING ARTS TECHNICIANS provide technical and other assistance for the production, recording and broadcasting of artistic performances.

Indicative Skill Level:
Most occupations in this unit group have a level of skill commensurate with the qualifications and experience outlined below.

In Australia:
AQF Certificate III including at least two years of on-the-job training, or AQF Certificate IV (ANZSCO Skill Level 3)

In New Zealand:
NZ Register Level 4 qualification (ANZSCO Skill Level 3)

At least three years of relevant experience may substitute for the formal qualifications listed above. In some instances relevant experience and/or on-the-job training may be required in addition to the formal qualification.

Tasks Include:
- operating microwave equipment to transmit video information to transmitter sites and receiving video signals from remote locations
- maintaining and repairing radio and television transmitters and associated equipment
- selecting and attaching equipment to cameras, positioning cameras, and following the action of scenes being photographed while adjusting controls
- positioning equipment, such as spotlights, floodlights and cables, and operating lights during filming, broadcasting and stage performances
- applying and retouching make up during shooting and performance, including special effects make up such as scars and wounds
- designing and making musical instruments and instrument parts using specially selected materials and techniques similar to those used in cabinetmaking, metal pipe making, silversmithing and wood carving, and tuning and repairing musical instruments
- setting up and adjusting equipment such as microphones, and operating sound mixing consoles and associated equipment to regulate volume and sound quality
- selecting and setting up television recording, editing and mixing equipment, and adjusting and monitoring their operation

Occupations:
399511 Broadcast Transmitter Operator
399512 Camera Operator (Film, Television or Video)
399513 Light Technician
399514 Make Up Artist
399515 Musical Instrument Maker or Repairer
399516 Sound Technician
399517 Television Equipment Operator
399599 Performing Arts Technicians nec

399511 BROADCAST TRANSMITTER OPERATOR

Operates consoles to control radio or television broadcast transmitters.

Skill Level: 3

UNIT GROUP 3995 PERFORMING ARTS TECHNICIANS *continued*

399512 CAMERA OPERATOR (FILM, TELEVISION OR VIDEO)

Sets up and operates cameras to photograph scenes for film, television or video productions.

Skill Level: 3

Specialisation:

Focus Puller (Film)

399513 LIGHT TECHNICIAN

Positions and controls lighting equipment for film, television or video productions or stage performances.

Skill Level: 3

399514 MAKE UP ARTIST

Designs and applies make up to actors, presenters and other performing artists.

Skill Level: 3

399515 MUSICAL INSTRUMENT MAKER OR REPAIRER

Builds, repairs and restores musical instruments, and modifies and tunes them to owners' specifications.

Skill Level: 3

Specialisation:

Piano Tuner

399516 SOUND TECHNICIAN

Operates audio equipment to record, enhance, mix and amplify sound in support of television, radio, film or video productions, or stage performances.

Skill Level: 3

Specialisations:

Audio Operator
Dubbing Machine Operator
Foley Artist
Re-recording Mixer
Sound Editor
Sound Effects Person
Sound Mixer
Sound Recordist
Video and Sound Recorder

399517 TELEVISION EQUIPMENT OPERATOR

Operates television equipment to record, edit, mix and prepare material for broadcast.

Skill Level: 3

Specialisation:

Vision Mixer

UNIT GROUP 3995 PERFORMING ARTS TECHNICIANS *continued*

399599 PERFORMING ARTS TECHNICIANS NEC

This occupation group covers Performing Arts Technicians not elsewhere classified.

Skill Level: 3

Occupations in this group include:

- Continuity Person
- Microphone Boom Operator
- Performing Arts Road Manager
- Special Effects Person
- Theatrical Dresser

UNIT GROUP 3996 SIGNWRITERS

SIGNWRITERS design, fabricate and paint signs for displays, buildings, hoardings, boats and structures.

Indicative Skill Level:
Most occupations in this unit group have a level of skill commensurate with the qualifications and experience outlined below.

In Australia:
AQF Certificate III including at least two years of on-the-job training, or AQF Certificate IV (ANZSCO Skill Level 3)

In New Zealand:
NZ Register Level 4 qualification (ANZSCO Skill Level 3)

At least three years of relevant experience may substitute for the formal qualifications listed above. In some instances relevant experience and/or on-the-job training may be required in addition to the formal qualification.

Registration or licensing may be required.

Tasks Include:
- conferring with clients and responding to proposals, sketches and written instructions to determine composition of signs
- designing and creating signs and graphics using computer software and signmaking machines
- designing and creating signs by measuring and calculating letter size, preparing the surface, applying background paint using brushes, sprays and rollers, and creating the letters using brushes, stencils, enamel paint and decals
- designing and creating wall murals, screen prints, gold leaf work and custom vehicle art
- painting signs and lettering using lacquers, varnishes, paints and other materials
- painting signs on brick, metal, timber, glass, plastic and other surfaces
- making and erecting three dimensional signs
- preparing cost estimates for labour and materials
- may erect and work on scaffolding
- may install signs on-site

Occupation:
399611 Signwriter

399611 SIGNWRITER

Designs, fabricates and paints signs for displays, buildings, hoardings, boats and structures. Registration or licensing may be required.

Skill Level: 3

Specialisation:
Sign Manufacturer

UNIT GROUP 3999 OTHER MISCELLANEOUS TECHNICIANS AND TRADES WORKERS

This unit group covers Technicians and Trades Workers not elsewhere classified.

It includes Divers, Interior Decorators, Optical Dispensers / Dispensing Opticians, Optical Mechanics, Photographer's Assistants, Plastics Technicians and Wool Classers.

Indicative Skill Level:
Most occupations in this unit group have a level of skill commensurate with the qualifications and experience outlined below.

In Australia:
AQF Certificate III including at least two years of on-the-job training, or AQF Certificate IV (ANZSCO Skill Level 3)

In New Zealand:
NZ Register Level 4 qualification (ANZSCO Skill Level 3)

At least three years of relevant experience may substitute for the formal qualifications listed above. In some instances relevant experience and/or on-the-job training may be required in addition to the formal qualification.

The occupation Interior Decorator has a level of skill commensurate with the qualifications and experience outlined below.

In Australia:
AQF Associate Degree, Advanced Diploma or Diploma (ANZSCO Skill Level 2)

In New Zealand:
NZ Register Diploma (ANZSCO Skill Level 2)

At least three years of relevant experience may substitute for the formal qualifications listed above. In some instances relevant experience and/or on-the-job training may be required in addition to the formal qualification.

Registration or licensing may be required.

Occupations:
399911 Diver
399912 Interior Decorator
399913 Optical Dispenser (Aus) / Dispensing Optician (NZ)
399914 Optical Mechanic
399915 Photographer's Assistant
399916 Plastics Technician
399917 Wool Classer
399999 Technicians and Trades Workers nec

UNIT GROUP 3999 OTHER MISCELLANEOUS TECHNICIANS AND TRADES WORKERS *continued*

399911 DIVER

Swims underwater to undertake tasks such as seafood gathering, research, salvage and construction. Registration or licensing may be required.

Skill Level: 3

Specialisations:

- Abalone Diver
- Clearance Diver (Navy)
- Fisheries Diver
- Hyperbaric Welder Diver
- Offshore Diver
- Onshore Diver
- Pearl Diver
- Saturation Diver
- Scientific Diver

399912 INTERIOR DECORATOR

Plans the interior design of commercial or residential premises and arranges for decorating work to be done.

Skill Level: 2

399913 OPTICAL DISPENSER (AUS) / DISPENSING OPTICIAN (NZ)

Interprets optical prescriptions, and fits and services optical appliances such as spectacle frames and lenses. Registration or licensing may be required.

Skill Level: 3

399914 OPTICAL MECHANIC

Operates machines to grind, polish and surface optical lenses to meet prescription requirements, and fits lenses to spectacle frames.

Skill Level: 3

399915 PHOTOGRAPHER'S ASSISTANT

Assists Photographers in taking and developing photographs.

Skill Level: 3

399916 PLASTICS TECHNICIAN

Alternative Title:

- Plastics Fitter

Sets up, adjusts, repairs and troubleshoots machines which manufacture plastics products.

Skill Level: 3

399917 WOOL CLASSER

Classifies wool to industry standards or market requirements.

Skill Level: 3

UNIT GROUP 3999 OTHER MISCELLANEOUS TECHNICIANS AND TRADES WORKERS *continued*

399999 TECHNICIANS AND TRADES WORKERS NEC

This occupation group covers Technicians and Trades Workers not elsewhere classified.

Skill Level: 3

Occupations in this group include:

- Airborne Electronics Analyst (Air Force)
- Architectural Model Maker
- Canoe Maker
- Fire Alarm Technician
- Fire Extinguisher Technician
- Glass Blower
- Hide and Skin Classer
- Kayak Maker
- Micrographic Technician
- Milking Machine Technician
- Parachute Rigger
- Pearl Technician
- Pyrotechnician
- Surfboard Maker

MAJOR GROUP 4 COMMUNITY AND PERSONAL SERVICE WORKERS

COMMUNITY AND PERSONAL SERVICE WORKERS assist Health Professionals in the provision of patient care, provide information and support on a range of social welfare matters, and provide other services in the areas of aged care and childcare, education support, hospitality, defence, policing and emergency services, security, travel and tourism, fitness, sports and personal services.

Indicative Skill Level:
Most occupations in this major group have a level of skill commensurate with the qualifications and experience outlined below.

In Australia:
AQF Associate Degree, Advanced Diploma or Diploma, or at least three years of relevant experience (ANZSCO Skill Level 2); or
AQF Certificate III including at least two years of on-the-job training, or AQF Certificate IV, or at least three years of relevant experience (ANZSCO Skill Level 3); or
AQF Certificate II or III, or at least one year of relevant experience (ANZSCO Skill Level 4); or
AQF Certificate I, or compulsory secondary education (ANZSCO Skill Level 5)

In New Zealand:
NZ Register Diploma, or at least three years of relevant experience (ANZSCO Skill Level 2); or
NZ Register Level 4 qualification, or at least three years of relevant experience (ANZSCO Skill Level 3); or
NZ Register Level 2 or 3 qualification, or at least one year of relevant experience (ANZSCO Skill Level 4); or
NZ Register Level 1 qualification, or compulsory secondary education (ANZSCO Skill Level 5)

In some instances relevant experience and/or on-the-job training may be required in addition to the formal qualification. In the case of some Skill Level 5 occupations, a short period of on-the-job training may be required in addition to or instead of the formal qualification, or no formal qualification or on-the-job training may be required.

Tasks Include:
- attending accidents, planning and implementing leisure activities for individuals in health care and the community, and providing nursing care for patients
- advising clients on emotional, financial, recreational, health, housing and other social welfare matters
- planning, conducting and participating in educational and recreational activities to encourage the physical, social, emotional and intellectual development of children
- assisting Professionals in the provision of care and support to aged and disabled persons, patients in hospitals, clinics and nursing homes, and children in residential care establishments
- serving and selling food and beverages in bars, cafes and restaurants, supervising staff in hotels, carrying luggage and escorting guests
- maintaining public order and safety and providing specialised military services to the defence forces
- protecting, patrolling and guarding properties and advising clients on security requirements
- providing a range of personal services such as beauty therapy, teaching people to drive, arranging funerals, and organising and providing advice about travel and accommodation

- organising and supervising groups and individuals pursuing physical fitness goals and outdoor adventure, participating in and officiating at sporting competitions, and coaching and training sporting competitors

Occupations in this major group are classified into the following sub-major groups:

41 Health and Welfare Support Workers
42 Carers and Aides
43 Hospitality Workers
44 Protective Service Workers
45 Sports and Personal Service Workers

SUB-MAJOR GROUP 41 HEALTH AND WELFARE SUPPORT WORKERS

HEALTH AND WELFARE SUPPORT WORKERS assist Health Professionals in the provision of patient care in hospitals, nursing homes and other health and community-based care facilities, and provide support, information and advice to clients on a range of social welfare matters.

Indicative Skill Level:
Most occupations in this sub-major group have a level of skill commensurate with the qualifications and experience outlined below.

In Australia:

AQF Associate Degree, Advanced Diploma or Diploma, or at least three years of relevant experience (ANZSCO Skill Level 2); or

AQF Certificate III including at least two years of on-the-job training, or AQF Certificate IV, or at least three years of relevant experience (ANZSCO Skill Level 3)

In New Zealand:

NZ Register Diploma, or at least three years of relevant experience (ANZSCO Skill Level 2); or

NZ Register Level 4 qualification, or at least three years of relevant experience (ANZSCO Skill Level 3)

In some instances relevant experience and/or on-the-job training may be required in addition to the formal qualification.

Tasks Include:

- attending accidents and providing pre-hospital care and transport
- examining and treating ailments of the teeth and gums, and constructing and repairing dental devices
- planning and implementing leisure activity programs for individuals in health care and in the community to assist in their social development and promote a sense of wellbeing
- assessing, planning and implementing nursing care for patients according to accepted nursing practice and standards
- providing advice, training and support to parents of newborn infants
- acting as an advocate, interpreter and educator to assist in the provision and coordination of health care delivery to Indigenous communities
- utilising a range of techniques such as soft tissue massage to assist healing, prevent injury and promote relaxation
- advising clients on emotional, financial, recreational, health, housing and other social welfare matters

Occupations in this sub-major group are classified into the following minor group:

411 Health and Welfare Support Workers

MINOR GROUP 411 HEALTH AND WELFARE SUPPORT WORKERS

HEALTH AND WELFARE SUPPORT WORKERS assist Health Professionals in the provision of patient care in hospitals, nursing homes and other health and community-based care facilities, and provide support, information and advice to clients on a range of social welfare matters.

Indicative Skill Level:
Most occupations in this minor group have a level of skill commensurate with the qualifications and experience outlined below.

In Australia:

AQF Associate Degree, Advanced Diploma or Diploma, or at least three years of relevant experience (ANZSCO Skill Level 2); or

AQF Certificate III including at least two years of on-the-job training, or AQF Certificate IV, or at least three years of relevant experience (ANZSCO Skill Level 3)

In New Zealand:

NZ Register Diploma, or at least three years of relevant experience (ANZSCO Skill Level 2); or

NZ Register Level 4 qualification, or at least three years of relevant experience (ANZSCO Skill Level 3)

In some instances relevant experience and/or on-the-job training may be required in addition to the formal qualification.

Tasks Include:

- attending accidents and providing pre-hospital care and transport
- examining and treating ailments of the teeth and gums, and constructing and repairing dental devices
- planning and implementing leisure activity programs for individuals in health care and in the community to assist in their social development and promote a sense of wellbeing
- assessing, planning and implementing nursing care for patients according to accepted nursing practice and standards
- providing advice, training and support to parents of newborn infants
- acting as an advocate, interpreter and educator to assist in the provision and coordination of health care delivery to Indigenous communities
- utilising a range of techniques such as soft tissue massage to assist healing, prevent injury and promote relaxation
- advising clients on emotional, financial, recreational, health, housing and other social welfare matters

Occupations in this minor group are classified into the following unit groups:

4111 Ambulance Officers and Paramedics
4112 Dental Hygienists, Technicians and Therapists
4113 Diversional Therapists
4114 Enrolled and Mothercraft Nurses
4115 Indigenous Health Workers
4116 Massage Therapists
4117 Welfare Support Workers

UNIT GROUP 4111 AMBULANCE OFFICERS AND PARAMEDICS

AMBULANCE OFFICERS AND PARAMEDICS provide emergency health care and transport for injured, sick, infirm and aged persons to medical facilities.

Indicative Skill Level:
Most occupations in this unit group have a level of skill commensurate with the qualifications and experience outlined below.

In Australia:
AQF Associate Degree, Advanced Diploma or Diploma (ANZSCO Skill Level 2)

In New Zealand:
NZ Register Diploma (ANZSCO Skill Level 2)

At least three years of relevant experience may substitute for the formal qualifications listed above. In some instances relevant experience and/or on-the-job training may be required in addition to the formal qualification.

Registration or licensing is required.

Tasks Include:
- attending accidents, emergencies and requests for medical assistance
- assessing health of patients, determining need for assistance, and assessing specialised needs and factors affecting patients' conditions
- performing therapies and administering drugs according to protocol
- resuscitating and defibrillating patients and operating life-support equipment
- transporting accident victims to medical facilities
- transporting sick and disabled persons to and from medical facilities for specialised treatment and rehabilitation
- instructing community groups and essential service workers in first aid
- attending public gatherings and sporting events where accidents and other health emergencies may occur
- ensuring that ambulances are adequately maintained and stocked with medical supplies, and that equipment is in good working order
- preparing written reports on the state of patients' injuries and treatment provided

Occupations:
411111 Ambulance Officer
411112 Intensive Care Ambulance Paramedic (Aus) / Ambulance Paramedic (NZ)

411111 AMBULANCE OFFICER

Alternative Title:
Paramedic (Aus)

Provides specialised transport services and emergency health care for injured, sick, infirm and aged persons. Registration or licensing is required.

Skill Level: 2

Specialisation:
Patient Transport Officer (Aus)

UNIT GROUP 4111 AMBULANCE OFFICERS AND PARAMEDICS *continued*

411112 INTENSIVE CARE AMBULANCE PARAMEDIC (AUS) / AMBULANCE PARAMEDIC (NZ)

Provides intensive pre-hospital health care to injured, sick, infirm and aged persons and emergency transport to medical facilities. Registration or licensing is required.

Skill Level: 2

UNIT GROUP 4112 DENTAL HYGIENISTS, TECHNICIANS AND THERAPISTS

DENTAL HYGIENISTS, TECHNICIANS AND THERAPISTS provide supportive dental services in preventative and restorative dental procedures, and construct and repair dental appliances.

Indicative Skill Level:
Most occupations in this unit group have a level of skill commensurate with the qualifications and experience outlined below.

In Australia:
AQF Associate Degree, Advanced Diploma or Diploma (ANZSCO Skill Level 2)

In New Zealand:
NZ Register Diploma (ANZSCO Skill Level 2)

At least three years of relevant experience may substitute for the formal qualifications listed above. In some instances relevant experience and/or on-the-job training may be required in addition to the formal qualification.

Registration or licensing may be required.

Tasks Include:
- providing educational programs to motivate children, parents and the community in matters relating to oral health
- providing fluoride therapy by applying remineralising solutions and desensitising agents
- removing deposits from teeth
- applying non-invasive fissure sealants to teeth
- taking impressions of the mouth
- taking dental radiographs
- administering local anaesthesia by infiltration and mandibular nerve block
- fabricating full and partial dentures
- constructing mouth guards, crowns, metal clasps, inlays, bridgework and other aids
- repairing and relining denture bases

Occupations:
411211 Dental Hygienist
411212 Dental Prosthetist
411213 Dental Technician
411214 Dental Therapist

411211 DENTAL HYGIENIST

Carries out preventative dental procedures under the direction of a Dentist. Registration or licensing is required.

Skill Level: 2

411212 DENTAL PROSTHETIST

Alternative Title:
Clinical Dental Technician

Designs, constructs, repairs and fits dentures and mouthguards. Registration or licensing is required.

Skill Level: 2

UNIT GROUP 4112 DENTAL HYGIENISTS, TECHNICIANS AND THERAPISTS *continued*

411213 DENTAL TECHNICIAN

Constructs and repairs dentures and other dental appliances. Registration or licensing may be required.

Skill Level: 2

411214 DENTAL THERAPIST

Examines and treats diseases of the teeth in preschool, primary and secondary school children under the general supervision of a Dentist. Registration or licensing is required.

Skill Level: 2

Specialisation:
Oral Health Therapist

UNIT GROUP 4113 DIVERSIONAL THERAPISTS

DIVERSIONAL THERAPISTS plan, design, coordinate and implement recreation and leisure-based activity programs to support, challenge and enhance the psychological, spiritual, social, emotional and physical wellbeing of individuals.

Indicative Skill Level:
Most occupations in this unit group have a level of skill commensurate with the qualifications and experience outlined below.

In Australia:
AQF Certificate III including at least two years of on-the-job training, or AQF Certificate IV (ANZSCO Skill Level 3)

In New Zealand:
NZ Register Level 4 qualification (ANZSCO Skill Level 3)

At least three years of relevant experience may substitute for the formal qualifications listed above. In some instances relevant experience and/or on-the-job training may be required in addition to the formal qualification.

Registration or licensing may be required.

Tasks Include:
- planning and implementing leisure activity programs for individuals in health care and in the community to assist in their social development, and promote their sense of wellbeing
- identifying individual needs through task analysis
- evaluating and assessing clients' levels of abilities, interests, needs, strengths and weaknesses, and their ability to carry out a range of tasks and interact with others
- maintaining a knowledge of resources available within a facility and within the community
- organising leisure and recreational events
- assisting with training and supervising volunteers and staff
- providing information on available support resources within the local community
- encouraging and supporting clients to take part in activities suited to their particular needs and interests
- adapting programs to suit individual clients' needs, interests, skills and abilities

Occupation:
411311 Diversional Therapist

411311 DIVERSIONAL THERAPIST

Alternative Title:
Recreational Therapist

Plans, designs, coordinates and implements recreation and leisure-based activity programs to support, challenge and enhance the psychological, spiritual, social, emotional and physical wellbeing of individuals. Registration or licensing may be required.

Skill Level: 3

Specialisations:
Activities Coordinator
Activities Officer

UNIT GROUP 4114 ENROLLED AND MOTHERCRAFT NURSES

ENROLLED AND MOTHERCRAFT NURSES provide nursing care to patients in hospitals, aged care and other health care facilities and in the community, and assist parents in providing care to newborn infants under the supervision of a Registered Nurse or Midwife.

Indicative Skill Level:
Most occupations in this unit group have a level of skill commensurate with the qualifications and experience outlined below.

In Australia:
AQF Associate Degree, Advanced Diploma or Diploma (ANZSCO Skill Level 2)

In New Zealand:
NZ Register Diploma (ANZSCO Skill Level 2)

At least three years of relevant experience may substitute for the formal qualifications listed above. In some instances relevant experience and/or on-the-job training may be required in addition to the formal qualification.

Registration or licensing is required.

Tasks Include:
- assessing, planning and implementing nursing care for patients according to accepted nursing practice and standards
- providing interventions, treatments and therapies such as administering medications, and monitoring responses to treatments and care plans
- assisting Registered Nurses and other team members to coordinate and evaluate care provided
- promoting and assisting in health education activities for the prevention of ill health
- bathing, feeding, changing and settling newborn infants
- providing advice and training on infant care to parents of newborn infants
- providing emotional support to parents of newborn infants

Occupations:
411411 Enrolled Nurse
411412 Mothercraft Nurse

411411 ENROLLED NURSE

Alternative Title:
Nursing Assistant (NZ)

Provides nursing care to patients in a variety of health, aged care, welfare and community settings under the supervision of Registered Nurses. Registration or licensing is required.

Skill Level: 2

Specialisation:
Medical Assistant (Defence)

411412 MOTHERCRAFT NURSE

Provides care to newborn infants, and provides advice and training on infant care to parents of newborn infants. Registration or licensing is required.

Skill Level: 2

UNIT GROUP 4115 INDIGENOUS HEALTH WORKERS

INDIGENOUS HEALTH WORKERS assist with the coordination and provision of health care delivery to Indigenous communities.

Indicative Skill Level:
Most occupations in this unit group have a level of skill commensurate with the qualifications and experience outlined below.

In Australia:
AQF Associate Degree, Advanced Diploma or Diploma (ANZSCO Skill Level 2)

In New Zealand:
NZ Register Diploma (ANZSCO Skill Level 2)

At least three years of relevant experience may substitute for the formal qualifications listed above. In some instances relevant experience and/or on-the-job training may be required in addition to the formal qualification.

Registration or licensing may be required.

Tasks Include:
- maintaining health records and statistics
- acting as an advocate in the community they serve, and as a communicator and interpreter on behalf of clients and other health workers
- providing clinical functions, such as case management and follow-up, independently or in consultation with other health care providers
- providing health education to individual clients and staff in health facilities
- providing cultural education to persons outside the cultural community and life skills education to the community they serve
- providing counselling and referring clients to other health care providers where necessary

Occupations:
411511 Aboriginal and Torres Strait Islander Health Worker
411512 Kaiāwhina (Hauora) (Māori Health Assistant)

411511 ABORIGINAL AND TORRES STRAIT ISLANDER HEALTH WORKER

Liaises with patients, clients, visitors to hospitals and other medical facilities and staff at health clinics, and works as a team member to arrange, coordinate and provide health care delivery in Aboriginal and Torres Strait Islander community health clinics. Registration or licensing may be required.

Skill Level: 2

411512 KAIĀWHINA (HAUORA) (MĀORI HEALTH ASSISTANT)

Assists with health care delivery to patients and clients in accordance with Tikanga Māori (Māori culture and custom).

Skill Level: 2

UNIT GROUP 4116 MASSAGE THERAPISTS

MASSAGE THERAPISTS perform therapeutic massage and administer body treatments for health, fitness and remedial purposes.

Indicative Skill Level:
Most occupations in this unit group have a level of skill commensurate with the qualifications and experience outlined below.

In Australia:
AQF Associate Degree, Advanced Diploma or Diploma (ANZSCO Skill Level 2)

In New Zealand:
NZ Register Diploma (ANZSCO Skill Level 2)

At least three years of relevant experience may substitute for the formal qualifications listed above. In some instances relevant experience and/or on-the-job training may be required in addition to the formal qualification.

Tasks Include:
- massaging the soft tissues of the body, such as muscles, tendons and ligaments, to assist healing
- utilising a range of massage techniques to enhance sports performance and prevent injury
- administering treatments to promote relaxation, improve circulation and relieve muscle tension
- assessing and treating specific soft tissue dysfunction and providing rehabilitation advice
- employing other techniques, such as acupressure or Shiatsu, and complementary aids, such as infra-red lamps, wet compresses, ice, essential oils and herbal and mineral therapies, to assist recovery
- assessing client's physical condition and case history and advising on stretching exercises and relaxation techniques

Occupation:
411611 Massage Therapist

411611 MASSAGE THERAPIST

Performs therapeutic massage and administers body treatments for relaxation, health, fitness and remedial purposes.

Skill Level: 2

Specialisations:
Chinese (Tui-Na) Masseur
Remedial Masseur
Shiatsu Therapist
Sports Medicine Masseur

UNIT GROUP 4117 WELFARE SUPPORT WORKERS

WELFARE SUPPORT WORKERS provide support, information and advice to clients on emotional, financial, recreational, health, housing and other social welfare matters, and evaluate and coordinate the services of welfare and community service agencies.

Indicative Skill Level:
Most occupations in this unit group have a level of skill commensurate with the qualifications and experience outlined below.

In Australia:
AQF Associate Degree, Advanced Diploma or Diploma (ANZSCO Skill Level 2)

In New Zealand:
NZ Register Diploma (ANZSCO Skill Level 2)

At least three years of relevant experience may substitute for the formal qualifications listed above. In some instances relevant experience and/or on-the-job training may be required in addition to the formal qualification.

Tasks Include:
- assessing clients' needs and planning, developing and implementing educational, training and support programs
- interviewing clients and assessing the nature and extent of difficulties
- monitoring and reporting on the progress of clients
- referring clients to agencies that can provide additional help
- assessing community need and resources for health, welfare, housing, employment, training and other facilities and services
- liaising with community groups, welfare agencies, government bodies and private businesses about community issues and promoting awareness of community resources and services
- supporting families and providing education and care for children and disabled persons in adult service units, group housing and government institutions
- supervising offenders on probation and parole
- assisting young people to solve social, emotional and financial problems
- preparing submissions for funding and resources, and reports to government bodies and other agencies

Occupations:
411711 Community Worker
411712 Disabilities Services Officer
411713 Family Support Worker
411714 Parole or Probation Officer
411715 Residential Care Officer
411716 Youth Worker

UNIT GROUP 4117 WELFARE SUPPORT WORKERS *continued*

411711 COMMUNITY WORKER

Facilitates community development initiatives and collective solutions within a community to address issues, needs and problems associated with recreational, health, housing, employment and other welfare matters.

Skill Level: 2

Specialisations:
- Community Development Officer
- Community Support Worker
- Housing Officer

411712 DISABILITIES SERVICES OFFICER

Works in a range of service units which provide education and community access to people with intellectual, physical, social and emotional disabilities.

Skill Level: 2

411713 FAMILY SUPPORT WORKER

Assists the work of Social Workers and Welfare Workers by providing services and support to families.

Skill Level: 2

411714 PAROLE OR PROBATION OFFICER

Supervises offenders who have been placed on probation by court order or released conditionally from corrective service institutions.

Skill Level: 2

411715 RESIDENTIAL CARE OFFICER

Provides care and supervision for children or disabled persons in group housing or institutional care.

Skill Level: 2

411716 YOUTH WORKER

Alternative Titles:
- Youth Officer
- Youth Support Worker

Assists young people as individuals or groups to solve social, emotional and financial problems in an agency framework.

Skill Level: 2

Specialisations:
- Juvenile Justice Officer
- Youth Accommodation Support Worker
- Youth Liaison Officer

SUB-MAJOR GROUP 42 CARERS AND AIDES

CARERS AND AIDES provide basic care, supervision and other support services to individuals for the enhancement of their education, health, welfare and comfort.

Indicative Skill Level:
Most occupations in this sub-major group have a level of skill commensurate with the qualifications and experience outlined below.

In Australia:
AQF Certificate II or III (ANZSCO Skill Level 4)

In New Zealand:
NZ Register Level 2 or 3 qualification (ANZSCO Skill Level 4)

At least one year of relevant experience may substitute for the formal qualifications listed above. In some instances relevant experience and/or on-the-job training may be required in addition to the formal qualification.

Tasks Include:
- planning, conducting and participating in educational and recreational activities to encourage the physical, social, emotional and intellectual development of children
- supervising children in recreational activities
- preparing and distributing educational aids
- assisting children with intellectual, physical and behavioural difficulties with their academic studies
- assisting Professionals in the provision of care and support to aged and disabled persons, patients in hospitals, clinics and nursing homes, and children in residential care establishments
- assisting patients and clients with personal care needs, rehabilitative exercises and providing emotional support

Occupations in this sub-major group are classified into the following minor groups:
421 Child Carers
422 Education Aides
423 Personal Carers and Assistants

MINOR GROUP 421 CHILD CARERS

CHILD CARERS provide care and supervision for children in residential homes and non-residential childcare centres.

Indicative Skill Level:
Most occupations in this minor group have a level of skill commensurate with the qualifications and experience outlined below.

In Australia:
AQF Certificate II or III (ANZSCO Skill Level 4)

In New Zealand:
NZ Register Level 2 or 3 qualification (ANZSCO Skill Level 4)

At least one year of relevant experience may substitute for the formal qualifications listed above. In some instances relevant experience and/or on-the-job training may be required in addition to the formal qualification.

Tasks Include:
- assisting in the preparation of materials and equipment for children's education and recreational activities
- managing children's behaviour and guiding children's social development
- preparing and conducting activities for children
- entertaining children by reading and playing games
- supervising children in recreational activities
- supervising the daily routine of children
- supervising the hygiene of children

Occupations in this minor group are classified into the following unit group:
4211 Child Carers

UNIT GROUP 4211 CHILD CARERS

CHILD CARERS provide care and supervision for children in residential homes and non-residential childcare centres.

Indicative Skill Level:
Most occupations in this unit group have a level of skill commensurate with the qualifications and experience outlined below.

In Australia:
AQF Certificate II or III (ANZSCO Skill Level 4)

In New Zealand:
NZ Register Level 2 or 3 qualification (ANZSCO Skill Level 4)

At least one year of relevant experience may substitute for the formal qualifications listed above. In some instances relevant experience and/or on-the-job training may be required in addition to the formal qualification.

Registration or licensing may be required.

Tasks Include:
- assisting in the preparation of materials and equipment for children's education and recreational activities
- managing children's behaviour and guiding children's social development
- preparing and conducting activities for children
- entertaining children by reading and playing games
- supervising children in recreational activities
- supervising the daily routine of children
- supervising the hygiene of children

Occupations:
421111 Child Care Worker
421112 Family Day Care Worker
421113 Nanny
421114 Out of School Hours Care Worker

421111 CHILD CARE WORKER

Alternative Title:
Child Care Aide

Provides care and supervision for children in programs, such as long day care and occasional care, in childcare centres, hospitals and educational centres. Registration or licensing may be required.

Skill Level: 4

Specialisations:
Children's Nursery Assistant
Creche Attendant

UNIT GROUP 4211 CHILD CARERS *continued*

421112 FAMILY DAY CARE WORKER

Alternative Title:

Family Day Carer

Provides care and supervision for babies and children, usually in the carer's own home and under local government or community-based schemes. Registration or licensing may be required.

Skill Level: 4

421113 NANNY

Assists parents in the provision of ongoing care and supervision for babies and children, usually in the child's home.

Skill Level: 4

Specialisation:

Governess

421114 OUT OF SCHOOL HOURS CARE WORKER

Provides care for school age children in an out of school hours care program. Registration or licensing may be required.

Skill Level: 4

MINOR GROUP 422 EDUCATION AIDES

EDUCATION AIDES perform non-teaching duties to assist teaching staff in schools, provide care and supervision for children in preschools, and provide assistance to Aboriginal, Torres Strait Islander and Māori students and their teachers.

Indicative Skill Level:
Most occupations in this minor group have a level of skill commensurate with the qualifications and experience outlined below.

In Australia:
AQF Certificate II or III (ANZSCO Skill Level 4)

In New Zealand:
NZ Register Level 2 or 3 qualification (ANZSCO Skill Level 4)

At least one year of relevant experience may substitute for the formal qualifications listed above. In some instances relevant experience and/or on-the-job training may be required in addition to the formal qualification.

Tasks Include:
- demonstrating, supervising and participating in activities which enhance the physical, social, emotional and intellectual development of children in schools and preschool centres
- preparing indoor and outdoor areas for learning and recreational activities
- assisting children with intellectual, physical and behavioural difficulties with their academic studies
- assisting children individually to learn social skills
- assisting with preparing teaching aids, and copying and collating written and printed material
- distributing and collecting lesson material
- providing assistance to small groups of Aboriginal, Torres Strait Islander and Māori students
- providing home-school liaison and counselling for Aboriginal, Torres Strait Islander and Māori students and their families

Occupations in this minor group are classified into the following unit group:
4221 Education Aides

UNIT GROUP 4221 EDUCATION AIDES

EDUCATION AIDES perform non-teaching duties to assist teaching staff in schools, provide care and supervision for children in preschools, and provide assistance to Aboriginal, Torres Strait Islander and Māori students and their teachers.

Indicative Skill Level:
Most occupations in this unit group have a level of skill commensurate with the qualifications and experience outlined below.

In Australia:
AQF Certificate II or III (ANZSCO Skill Level 4)

In New Zealand:
NZ Register Level 2 or 3 qualification (ANZSCO Skill Level 4)

At least one year of relevant experience may substitute for the formal qualifications listed above. In some instances relevant experience and/or on-the-job training may be required in addition to the formal qualification.

Tasks Include:
- demonstrating, supervising and participating in activities which enhance the physical, social, emotional and intellectual development of children in schools and preschool centres
- preparing indoor and outdoor areas for learning and recreational activities
- assisting children with intellectual, physical and behavioural difficulties with their academic studies
- assisting children individually to learn social skills
- assisting with preparing teaching aids, and copying and collating written and printed material
- distributing and collecting lesson material
- providing assistance to small groups of Aboriginal, Torres Strait Islander and Māori students
- providing home-school liaison and counselling for Aboriginal, Torres Strait Islander and Māori students and their families

Occupations:
422111 Aboriginal and Torres Strait Islander Education Worker
422112 Integration Aide
422113 Kaiāwhina Kōhanga Reo (Māori Language Nest Assistant)
422114 Kaiāwhina Kura Kaupapa Māori (Māori-medium School Assistant)
422115 Preschool Aide
422116 Teachers' Aide

422111 ABORIGINAL AND TORRES STRAIT ISLANDER EDUCATION WORKER

Assists Aboriginal and Torres Strait Islander students in their education, provides feedback to parents or guardians and teachers about students' progress, and liaises with educational bodies, government agencies and committees.

Skill Level: 4

Specialisations:
Aboriginal Education Worker Coordinator
Aboriginal Home-School Liaison Officer

UNIT GROUP 4221 EDUCATION AIDES *continued*

422112 INTEGRATION AIDE

Assists children with developmental disabilities in mainstream schools

Skill Level: 4

422113 KAIĀWHINA KŌHANGA REO (MĀORI LANGUAGE NEST ASSISTANT)

Assists Kaiako Kōhanga Reo (Māori Language Nest Teachers) with teaching duties and activities in the Māori language for children at pre-primary or early childhood level with emphasis given to Tikanga Māori (Māori custom).

Skill Level: 4

422114 KAIĀWHINA KURA KAUPAPA MĀORI (MĀORI-MEDIUM SCHOOL ASSISTANT)

Assists Kaiako Kura Kaupapa Māori (Māori-medium Primary School Teachers) with teaching duties and activities in the Māori language for children at primary school level with emphasis given to Tikanga Māori (Māori custom).

Skill Level: 4

422115 PRESCHOOL AIDE

Provides care and supervision for children at preschool centres under the direction of Early Childhood (Pre-primary School) Teachers.

Skill Level: 4

Specialisation:
- Kindergarten Assistant

422116 TEACHERS' AIDE

Assists teaching staff in preparing teaching materials and with general classroom tasks.

Skill Level: 4

Specialisations:
- School Services Officer
- Student Liaison Officer
- Teachers' Assistant

MINOR GROUP 423 PERSONAL CARERS AND ASSISTANTS

PERSONAL CARERS AND ASSISTANTS provide basic care, supervision and other support services to individuals for the enhancement of their health, welfare and comfort.

Indicative Skill Level:
Most occupations in this minor group have a level of skill commensurate with the qualifications and experience outlined below.

In Australia:
AQF Certificate II or III (ANZSCO Skill Level 4)

In New Zealand:
NZ Register Level 2 or 3 qualification (ANZSCO Skill Level 4)

At least one year of relevant experience may substitute for the formal qualifications listed above. In some instances relevant experience and/or on-the-job training may be required in addition to the formal qualification.

Tasks Include:
- providing assistance, support, care and companionship to aged and disabled persons and others in need of care and in therapy programs
- assisting Dental Practitioners
- assisting in caring for patients in hospitals, clinics and nursing homes
- caring for and supervising children in residential childcare establishments and correctional institutions
- caring for people in refuges

Occupations in this minor group are classified into the following unit groups:
4231 Aged and Disabled Carers
4232 Dental Assistants
4233 Nursing Support and Personal Care Workers
4234 Special Care Workers

UNIT GROUP 4231 AGED AND DISABLED CARERS

AGED AND DISABLED CARERS provide general household assistance, emotional support, care and companionship for aged and disabled persons in their own homes.

Indicative Skill Level:
Most occupations in this unit group have a level of skill commensurate with the qualifications and experience outlined below.

In Australia:
AQF Certificate II or III (ANZSCO Skill Level 4)

In New Zealand:
NZ Register Level 2 or 3 qualification (ANZSCO Skill Level 4)

At least one year of relevant experience may substitute for the formal qualifications listed above. In some instances relevant experience and/or on-the-job training may be required in addition to the formal qualification.

Tasks Include:
- accompanying aged and disabled persons during daily activities
- assisting clients with their mobility
- preparing food for clients
- arranging social activities
- performing housekeeping tasks such as vacuuming and cleaning
- assisting in personal hygiene and dressing
- providing companionship, friendship and emotional support
- may do shopping and run errands
- may live in with the person

Occupation:
423111 Aged or Disabled Carer

423111 AGED OR DISABLED CARER

Alternative Titles:
Home Support Worker
Personal Carer
Personal Care Worker

Provides general household assistance, emotional support, care and companionship for aged or disabled people in their own homes.

Skill Level: 4

UNIT GROUP 4232 DENTAL ASSISTANTS

DENTAL ASSISTANTS prepare patients for dental examination and assist Dental Practitioners, Hygienists and Therapists in providing care and treatment.

Indicative Skill Level:
Most occupations in this unit group have a level of skill commensurate with the qualifications and experience outlined below.

In Australia:
AQF Certificate II or III (ANZSCO Skill Level 4)

In New Zealand:
NZ Register Level 2 or 3 qualification (ANZSCO Skill Level 4)

At least one year of relevant experience may substitute for the formal qualifications listed above. In some instances relevant experience and/or on-the-job training may be required in addition to the formal qualification.

Tasks Include:
- receiving and preparing patients
- arranging and handing instruments, medication, and other dental requisites to Dental Practitioners
- preparing dental materials and processing X-rays
- using suction devices and water sprays
- performing routine maintenance on equipment
- sterilising and preventing cross infection of equipment
- may advise patients on dental health education and post-operative care and procedures
- may act as receptionist for Dental Practitioners
- may perform billing and other clerical tasks

Occupation:
423211 Dental Assistant

423211 DENTAL ASSISTANT

Alternative Titles:
Dental Chairside Assistant
Dental Nurse

Prepares patients for dental examination and assists Dental Practitioners, Hygienists and Therapists in providing care and treatment.

Skill Level: 4

UNIT GROUP 4233 NURSING SUPPORT AND PERSONAL CARE WORKERS

NURSING SUPPORT AND PERSONAL CARE WORKERS provide assistance, support and direct care to patients in a variety of health, welfare and community settings.

Indicative Skill Level:
Most occupations in this unit group have a level of skill commensurate with the qualifications and experience outlined below.

In Australia:
AQF Certificate II or III (ANZSCO Skill Level 4)

In New Zealand:
NZ Register Level 2 or 3 qualification (ANZSCO Skill Level 4)

At least one year of relevant experience may substitute for the formal qualifications listed above. In some instances relevant experience and/or on-the-job training may be required in addition to the formal qualification.

Registration or licensing may be required.

Tasks Include:
- assisting patients with their personal care needs such as showering, dressing and eating
- assisting patients with their mobility and communication needs
- participating in planning the care of individuals
- following therapy plans such as interventions to assist those with dementia and behavioural problems
- observing and reporting changes in patients' condition, and reporting complaints about care
- assisting with rehabilitation exercises, basic treatment and delivering medications
- providing direct support and assistance to therapists

Occupations:
423311 Hospital Orderly
423312 Nursing Support Worker
423313 Personal Care Assistant
423314 Therapy Aide

423311 HOSPITAL ORDERLY

Alternative Titles:
Patient Services Assistant
Wardsperson

Assists with the provision of care to patients in a hospital by ensuring wards are neat and tidy, lifting and turning patients and transporting them in wheelchairs or on movable beds, and providing direct care and support.

Skill Level: 4

UNIT GROUP 4233 NURSING SUPPORT AND PERSONAL CARE WORKERS *continued*

423312 NURSING SUPPORT WORKER

Alternative Titles:

- Assistant in Nursing
- Nurses' Aide (NZ)

Provides limited patient care under the direction of nursing staff.

Skill Level: 4

Specialisation:

- Paramedical Aide

423313 PERSONAL CARE ASSISTANT

Provides routine personal care services to people in a range of health care facilities or in a person's home.

Skill Level: 4

423314 THERAPY AIDE

Alternative Title:

- Therapist's Assistant

Assists therapists in providing therapy programs and in the direct care of their patients in a variety of health, welfare and community settings. Registration or licensing may be required.

Skill Level: 4

Specialisations:

- Diversional Therapist's Assistant
- Occupational Therapist's Assistant
- Physiotherapist's Assistant

UNIT GROUP 4234 SPECIAL CARE WORKERS

SPECIAL CARE WORKERS provide care and supervision for children in residential childcare establishments and correctional institutions, and provide care and support to people in refuges.

Indicative Skill Level:
Most occupations in this unit group have a level of skill commensurate with the qualifications and experience outlined below.

In Australia:
AQF Certificate II or III (ANZSCO Skill Level 4)

In New Zealand:
NZ Register Level 2 or 3 qualification (ANZSCO Skill Level 4)

At least one year of relevant experience may substitute for the formal qualifications listed above. In some instances relevant experience and/or on-the-job training may be required in addition to the formal qualification.

Registration or licensing may be required.

Tasks Include:
- planning and implementing programs of supervision and care for children in residential care
- supervising and arranging activities to enhance the physical, social, emotional and intellectual development of children in residential care
- waking children and ensuring they are washed, dressed, fed and ready for educational and recreational activities
- supervising children during domestic activities such as eating meals and showering
- maintaining discipline, enforcing regulations and behaviour standards, compiling disciplinary reports and assisting in implementing remedial measures
- organising refuge accommodation
- providing emotional support to residents of refuges
- referring residents of refuges for health and welfare assistance
- ensuring security of refuge

Occupations:
423411 Child or Youth Residential Care Assistant
423412 Hostel Parent
423413 Refuge Worker

423411 CHILD OR YOUTH RESIDENTIAL CARE ASSISTANT

Alternative Title:
Residential Care Worker

Provides care and supervision for children in correctional services institutions. Registration or licensing may be required.

Skill Level: 4

UNIT GROUP 4234 SPECIAL CARE WORKERS *continued*

423412 HOSTEL PARENT

Alternative Title:

House Parent

Operates a residential childcare establishment which provides a home environment for children. Registration or licensing may be required.

Skill Level: 4

423413 REFUGE WORKER

Provides services and support to people seeking assistance in a refuge.

Skill Level: 4

SUB-MAJOR GROUP 43 HOSPITALITY WORKERS

HOSPITALITY WORKERS provide services to patrons of hotels, bars, cafes, restaurants, casinos and similar establishments.

Indicative Skill Level:
Most occupations in this sub-major group have a level of skill commensurate with the qualifications and experience outlined below.

In Australia:
AQF Certificate III including at least two years of on-the-job training, or AQF Certificate IV, or at least three years of relevant experience (ANZSCO Skill Level 3); or
AQF Certificate II or III, or at least one year of relevant experience (ANZSCO Skill Level 4); or
AQF Certificate I, or compulsory secondary education (ANZSCO Skill Level 5)

In New Zealand:
NZ Register Level 4 qualification, or at least three years of relevant experience (ANZSCO Skill Level 3); or
NZ Register Level 2 or 3 qualification, or at least one year of relevant experience (ANZSCO Skill Level 4); or
NZ Register Level 1 qualification, or compulsory secondary education (ANZSCO Skill Level 5)

In some instances relevant experience and/or on-the-job training may be required in addition to the formal qualification. In the case of some Skill Level 5 occupations, a short period of on-the-job training may be required in addition to or instead of the formal qualification, or no formal qualification or on-the-job training may be required.

Tasks Include:
- serving and selling beverages
- taking food orders and serving food
- clearing used dishes, cutlery and glassware from dining and drinking areas
- providing gaming services within casinos and other gaming establishments
- supervising and coordinating the activities of hotel porters and other hotel staff
- explaining and enforcing safety regulations in hotels
- carrying luggage and escorting guests
- may book tours, taxis and restaurants for guests

Occupations in this sub-major group are classified into the following minor group:
431 Hospitality Workers

MINOR GROUP 431 HOSPITALITY WORKERS

HOSPITALITY WORKERS provide services to patrons of hotels, bars, cafes, restaurants, casinos and similar establishments.

Indicative Skill Level:
Most occupations in this minor group have a level of skill commensurate with the qualifications and experience outlined below.

In Australia:

AQF Certificate III including at least two years of on-the-job training, or AQF Certificate IV, or at least three years of relevant experience (ANZSCO Skill Level 3); or
AQF Certificate II or III, or at least one year of relevant experience (ANZSCO Skill Level 4); or
AQF Certificate I, or compulsory secondary education (ANZSCO Skill Level 5)

In New Zealand:

NZ Register Level 4 qualification, or at least three years of relevant experience (ANZSCO Skill Level 3); or
NZ Register Level 2 or 3 qualification, or at least one year of relevant experience (ANZSCO Skill Level 4); or
NZ Register Level 1 qualification, or compulsory secondary education (ANZSCO Skill Level 5)

In some instances relevant experience and/or on-the-job training may be required in addition to the formal qualification. In the case of some Skill Level 5 occupations, a short period of on-the-job training may be required in addition to or instead of the formal qualification, or no formal qualification or on-the-job training may be required.

Tasks Include:

- serving and selling beverages
- taking food orders and serving food
- clearing used dishes, cutlery and glassware from dining and drinking areas
- providing gaming services within casinos and other gaming establishments
- supervising and coordinating the activities of hotel porters and other hotel staff
- explaining and enforcing safety regulations in hotels
- carrying luggage and escorting guests
- may book tours, taxis and restaurants for guests

Occupations in this minor group are classified into the following unit groups:

4311 Bar Attendants and Baristas
4312 Cafe Workers
4313 Gaming Workers
4314 Hotel Service Managers
4315 Waiters
4319 Other Hospitality Workers

UNIT GROUP 4311 BAR ATTENDANTS AND BARISTAS

BAR ATTENDANTS AND BARISTAS prepare, mix and serve alcoholic and non-alcoholic drinks to patrons in bars in licensed establishments, and prepare and serve espresso coffee and other hot beverages to patrons in cafes, coffee shops and dining establishments.

Indicative Skill Level:
Most occupations in this unit group have a level of skill commensurate with the qualifications and experience outlined below.

In Australia:
AQF Certificate II or III (ANZSCO Skill Level 4)

In New Zealand:
NZ Register Level 2 or 3 qualification (ANZSCO Skill Level 4)

At least one year of relevant experience may substitute for the formal qualifications listed above. In some instances relevant experience and/or on-the-job training may be required in addition to the formal qualification.

Tasks Include:

- preparing, serving and selling cocktails, mixed drinks, bottled, canned and other alcoholic and non-alcoholic beverages, and a variety of coffee beverages such as lattes, cappuccinos and other espresso-based beverages
- cleaning and maintaining bar service areas, coffee-making areas and espresso machines
- collecting payment for sales and operating cash registers
- promoting services and products
- washing glassware and arranging bottles and glasses
- tapping kegs and attaching supply lines
- replenishing drink dispensers, shelves and refrigerators
- selling light snacks
- selecting and grinding coffee

Occupations:
431111 Bar Attendant
431112 Barista

431111 BAR ATTENDANT

Alternative Title:
Bar Steward

Prepares, mixes and serves alcoholic and non-alcoholic drinks to patrons in a bar in a licensed establishment.

Skill Level: 4

431112 BARISTA

Prepares and serves espresso coffee and other hot beverages to patrons in a cafe, coffee shop, restaurant or dining establishment.

Skill Level: 4

UNIT GROUP 4312 CAFE WORKERS

CAFE WORKERS sell and serve food and beverages for consumption on premises in cafes and similar establishments.

Indicative Skill Level:
Most occupations in this unit group have a level of skill commensurate with the qualifications outlined below.

In Australia:
AQF Certificate I, or compulsory secondary education (ANZSCO Skill Level 5)

In New Zealand:
NZ Register Level 1 qualification, or compulsory secondary education (ANZSCO Skill Level 5)

For some occupations a short period of on-the-job training may be required in addition to or instead of the formal qualification. In some instances no formal qualification or on-the-job training may be required.

Tasks Include:
- preparing and serving food and beverages for consumption on the premises
- taking customers' food and beverage orders
- operating cash registers, accepting payments and preparing sales invoices
- clearing away used dishes and cutlery from tables when customers are finished
- cleaning and preparing tables for use
- washing dishes, cutlery and cooking utensils
- cleaning cafe equipment such as coffee grinders, espresso machines and ice makers
- participating in stocktakes and assisting in putting away new stock
- providing backup to other cafe employees

Occupation:
431211 Cafe Worker

431211 CAFE WORKER

Alternative Titles:
Cafe Assistant
Cafe Attendant

Sells and serves food and beverages for consumption on premises in a cafe or similar establishment.

Skill Level: 5

Specialisation:
Canteen Attendant

UNIT GROUP 4313 GAMING WORKERS

GAMING WORKERS provide gaming services within casinos and other gambling establishments.

Indicative Skill Level:
Most occupations in this unit group have a level of skill commensurate with the qualifications and experience outlined below.

In Australia:
AQF Certificate II or III (ANZSCO Skill Level 4)

In New Zealand:
NZ Register Level 2 or 3 qualification (ANZSCO Skill Level 4)

At least one year of relevant experience may substitute for the formal qualifications listed above. In some instances relevant experience and/or on-the-job training may be required in addition to the formal qualification.

Tasks Include:
- ensuring that games operating in the casino pit run smoothly
- monitoring cash drops to cashiers and chip transactions
- observing incidents and settling disputes arising at gaming tables
- dealing games in accordance with casino rules, policies and procedures and ensuring that bets are placed within the rules of the game
- checking that appropriate betting limit signs are in place
- checking playing cards
- verifying cash and colour chip change involving larger amounts with the casino gaming inspector
- advising patrons about the rules and etiquette of games
- counting the amount of cash chips in the float and entering a closer slip with the corresponding amount in the cash total
- calculating and paying winning bets

Occupation:
431311 Gaming Worker

431311 GAMING WORKER

Alternative Title:
Croupier

Provides gaming services within a casino or other gambling establishment.

Skill Level: 4

Specialisations:
Casino Gaming Inspector
Gaming Pit Boss

UNIT GROUP 4314 HOTEL SERVICE MANAGERS

HOTEL SERVICE MANAGERS supervise and coordinate the activities of hotel service workers.

Indicative Skill Level:
Most occupations in this unit group have a level of skill commensurate with the qualifications and experience outlined below.

In Australia:
AQF Certificate III including at least two years of on-the-job training, or AQF Certificate IV (ANZSCO Skill Level 3)

In New Zealand:
NZ Register Level 4 qualification (ANZSCO Skill Level 3)

At least three years of relevant experience may substitute for the formal qualifications listed above. In some instances relevant experience and/or on-the-job training may be required in addition to the formal qualification.

Tasks Include:
- determining work requirements and allocating duties to Commercial Housekeepers, Luggage Porters and Doorpersons
- conferring with managers to coordinate activities with other organisational units
- maintaining attendance records and rosters
- explaining and enforcing safety regulations
- overseeing the work of the unit and suggesting improvements and changes
- conferring with workers to resolve grievances
- may perform front office and hotel reception duties

Occupation:
431411 Hotel Service Manager

431411 HOTEL SERVICE MANAGER

Alternative Title:
Hotel Service Supervisor

Supervises and coordinates the activities of hotel service workers.

Skill Level: 3

Specialisations:
Front Office Manager (Hotel)
Head Housekeeper
Head Porter (Hotel)
Hotel Concierge
Hotel Office Manager

UNIT GROUP 4315 WAITERS

WAITERS serve food and beverages in hotels, restaurants, clubs and dining establishments.

Indicative Skill Level:
Most occupations in this unit group have a level of skill commensurate with the qualifications and experience outlined below.

In Australia:
AQF Certificate II or III (ANZSCO Skill Level 4)

In New Zealand:
NZ Register Level 2 or 3 qualification (ANZSCO Skill Level 4)

At least one year of relevant experience may substitute for the formal qualifications listed above. In some instances relevant experience and/or on-the-job training may be required in addition to the formal qualification.

Tasks Include:
- setting and arranging tables
- greeting customers and presenting them with menus and beverage lists
- taking orders and relaying them to kitchen and bar staff
- serving food and beverages
- opening bottles and pouring beverages
- clearing tables and returning dishes and cutlery to kitchen
- removing empty bottles and used glasses from tables, and refilling and replacing glasses
- collecting payments for sales and operating point of sales machines and cash registers
- may recommend wines to complement food

Occupation:
431511 Waiter

431511 WAITER

Alternative Title:
Food and Beverage Attendant

Serves food and beverages in a hotel, restaurant, club or dining establishment.

Skill Level: 4

Specialisations:
Drink Waiter
Formal Service Waiter
Silver Service Waiter
Sommelier
Wine Steward

UNIT GROUP 4319 OTHER HOSPITALITY WORKERS

This unit group covers Hospitality Workers not elsewhere classified.

It includes Bar Usefuls or Bussers, and Doorpersons or Luggage Porters.

Indicative Skill Level:
Most occupations in this unit group have a level of skill commensurate with the qualifications outlined below.

In Australia:
AQF Certificate I, or compulsory secondary education (ANZSCO Skill Level 5)

In New Zealand:
NZ Register Level 1 qualification, or compulsory secondary education (ANZSCO Skill Level 5)

For some occupations a short period of on-the-job training may be required in addition to or instead of the formal qualification. In some instances no formal qualification or on-the-job training may be required.

Occupations:
431911 Bar Useful or Busser
431912 Doorperson or Luggage Porter
431999 Hospitality Workers nec

431911 BAR USEFUL OR BUSSER

Alternative Titles:
Bar Back
Glassie

Cleans and maintains public areas in a bar, club or dining establishment by collecting and returning dishes, cutlery and glasses to the kitchen or bar, wiping tables, bars and spillages, and emptying bins and ashtrays.

Skill Level: 5

431912 DOORPERSON OR LUGGAGE PORTER

Assists guests in an accommodation establishment or passengers in a transport terminal by attending to and carrying luggage, welcoming and escorting guests, and attending to their general needs on arrival and departure.

Skill Level: 5

431999 HOSPITALITY WORKERS NEC

This occupation group covers Hospitality Workers not elsewhere classified.

Skill Level: 5

Occupations in this group include:
Cloakroom Attendant
Hospitality Trainee
Hotel Cellar Hand
Property Steward
Uniform Room Attendant

SUB-MAJOR GROUP 44 PROTECTIVE SERVICE WORKERS

PROTECTIVE SERVICE WORKERS protect and preserve property, public order and safety through the provision of defence, firefighting, police, custodial and security services.

Indicative Skill Level:
Most occupations in this sub-major group have a level of skill commensurate with the qualifications and experience outlined below.

In Australia:

AQF Associate Degree, Advanced Diploma or Diploma, or at least three years of relevant experience (ANZSCO Skill Level 2); or
AQF Certificate III including at least two years of on-the-job training, or AQF Certificate IV, or at least three years of relevant experience (ANZSCO Skill Level 3); or
AQF Certificate II or III, or at least one year of relevant experience (ANZSCO Skill Level 4); or
AQF Certificate I, or compulsory secondary education (ANZSCO Skill Level 5)

In New Zealand:

NZ Register Diploma, or at least three years of relevant experience (ANZSCO Skill Level 2); or
NZ Register Level 4 qualification, or at least three years of relevant experience (ANZSCO Skill Level 3); or
NZ Register Level 2 or 3 qualification, or at least one year of relevant experience (ANZSCO Skill Level 4); or
NZ Register Level 1 qualification, or compulsory secondary education (ANZSCO Skill Level 5)

In some instances relevant experience and/or on-the-job training may be required in addition to the formal qualification. In the case of some Skill Level 5 occupations, a short period of on-the-job training may be required in addition to or instead of the formal qualification, or no formal qualification or on-the-job training may be required.

Tasks Include:

- providing specialised military services to the defence forces
- controlling and extinguishing fires
- maintaining public order and safety through the enforcement of laws
- observing the conduct and behaviour of prisoners to prevent disturbances and escapes
- patrolling and guarding properties, and checking for unauthorised entry
- conducting investigations for clients
- advising clients on security requirements, and recommending and designing security specifications

Occupations in this sub-major group are classified into the following minor groups:

441 Defence Force Members, Fire Fighters and Police
442 Prison and Security Officers

MINOR GROUP 441 DEFENCE FORCE MEMBERS, FIRE FIGHTERS AND POLICE

DEFENCE FORCE MEMBERS, FIRE FIGHTERS AND POLICE protect and preserve property, public order and safety through the provision of specialised military services to the defence forces, the enforcement of laws, attendance at emergencies, and control and extinguishment of fires.

Indicative Skill Level:
Most occupations in this minor group have a level of skill commensurate with the qualifications and experience outlined below.

In Australia:
AQF Associate Degree, Advanced Diploma or Diploma, or at least three years of relevant experience (ANZSCO Skill Level 2); or
AQF Certificate III including at least two years of on-the-job training, or AQF Certificate IV, or at least three years of relevant experience (ANZSCO Skill Level 3)

In New Zealand:
NZ Register Diploma, or at least three years of relevant experience (ANZSCO Skill Level 2); or
NZ Register Level 4 qualification, or at least three years of relevant experience (ANZSCO Skill Level 3)

In some instances relevant experience and/or on-the-job training may be required in addition to the formal qualification.

Tasks Include:
- performing specialised military services for the defence forces
- controlling and extinguishing fires
- rescuing people stranded or trapped in dangerous situations
- maintaining public order and safety
- patrolling assigned areas
- investigating offences and complaints
- gathering evidence
- pursuing, arresting and interviewing suspects
- maintaining records and preparing reports

Occupations in this minor group are classified into the following unit groups:
4411 Defence Force Members - Other Ranks
4412 Fire and Emergency Workers
4413 Police

UNIT GROUP 4411 DEFENCE FORCE MEMBERS - OTHER RANKS

DEFENCE FORCE MEMBERS - OTHER RANKS provide specialised military services to the Australian and New Zealand Defence Forces.

Indicative Skill Level:
Most occupations in this unit group have a level of skill commensurate with the qualifications and experience outlined below.

In Australia:
AQF Certificate III including at least two years of on-the-job training, or AQF Certificate IV (ANZSCO Skill Level 3)

In New Zealand:
NZ Register Level 4 qualification (ANZSCO Skill Level 3)

At least three years of relevant experience may substitute for the formal qualifications listed above. In some instances relevant experience and/or on-the-job training may be required in addition to the formal qualification.

Tasks Include:

- guarding airfields and other defence force bases
- serving as an infantry soldier
- erecting bridges, building field defences, constructing temporary roads, clearing minefields, repairing airfields and demolishing targets using explosives
- crewing armoured fighting vehicles
- operating artillery, ground and surface-based defence systems, shipboard weapons and other advanced specialist military weapons and equipment
- providing firepower and ground-based air defence
- establishing and maintaining command, control and communications equipment and facilities
- conducting surveillance, reconnaissance and boarding operations
- maintaining personal arms and ammunition

Occupation:
441111 Defence Force Member - Other Ranks

UNIT GROUP 4411 DEFENCE FORCE MEMBERS - OTHER RANKS *continued*

441111 DEFENCE FORCE MEMBER - OTHER RANKS

Provides specialised military services to the Australian or New Zealand Defence Forces.

This occupation includes the following ranks:

Air Force: Aircraftman/Aircraftwoman, Corporal, Leading Aircraftman/Aircraftwoman

Army: Corporal, Lance Corporal, Private

Navy: Able Seaman, Leading Seaman, Seaman

This occupation excludes Defence Force Members - Other Ranks performing duties for which there is a civilian equivalent. These members are included with the closest civilian occupation. For example, Mechanic Recovery is included in Occupation 733115 Tow Truck Driver.

Skill Level: 3

Specialisations:

- Aircraft Life Support Fitter (Air Force)
- Airfield Defence Guard (Air Force)
- Air Surveillance Operator (Air Force)
- Geospatial Imagery Intelligence Analyst (Air Force)
- Security Police (Air Force)
- Signal Operator Linguist (Air Force)
- Signal Operator Technical (Air Force)
- Aircraft Life Support Fitter (Army)
- Air Dispatcher (Army)
- Combat Engineer (Army)
- Commando (Army)
- Crewman Armoured Personnel Carrier M113 (Army)
- Crewman Australian Light Armoured Vehicle (ASLAV) (Army)
- Crewman Main Battle Tank (Army)
- Ground Crewman Mission Support (Army)
- Gun Number (Army)
- Marine Specialist (Army)
- Operator Artillery Meteorology and Surveyor (Army)
- Operator Electronic Warfare (Army)
- Operator Ground Based Air Defence (Army)
- Operator Weapon Locating Radar (Army)
- Rifleman (Army)
- Acoustic Warfare Analyst (Navy)
- Aircrewman (Navy)
- Boatswains Mate (Navy)
- Combat Systems Operator (Navy)
- Combat Systems Operator Mine Warfare (Navy)
- Electronic Warfare - Linguist (Navy)
- Electronic Warfare - Technical (Navy)
- Naval Police Coxswain (Navy)

UNIT GROUP 4412 FIRE AND EMERGENCY WORKERS

FIRE AND EMERGENCY WORKERS attend emergencies to minimise risk to community safety and security and protect life and property.

Indicative Skill Level:
Most occupations in this unit group have a level of skill commensurate with the qualifications and experience outlined below.

In Australia:
AQF Certificate III including at least two years of on-the-job training, or AQF Certificate IV (ANZSCO Skill Level 3)

In New Zealand:
NZ Register Level 4 qualification (ANZSCO Skill Level 3)

At least three years of relevant experience may substitute for the formal qualifications listed above. In some instances relevant experience and/or on-the-job training may be required in addition to the formal qualification.

Registration or licensing may be required.

Tasks Include:
- attending the scene of fires and other emergencies reported to authorities
- rescuing and evacuating people stranded or trapped in dangerous situations
- operating pumps, spraying water, foam and chemicals from hoses, portable extinguishers and other appliances to extinguish fires and to disperse or neutralise dangerous substances
- cutting openings in buildings and crashed vehicles to free occupants
- maintaining site security systems
- administering first aid
- attending and participating in training activities, rescue classes, drills, demonstrations and courses in emergency and fire-fighting techniques
- training recruits in emergency procedures and practices
- visiting buildings and potential fire hazards to study access points and locations of hydrants
- maintaining tools and equipment

Occupations:
441211 Emergency Service Worker
441212 Fire Fighter

441211 EMERGENCY SERVICE WORKER

Alternative Title:
Emergency Response Officer

Attends the scene of emergencies to minimise risk to community safety and security.

Skill Level: 3

Specialisation:
Industrial Paramedic

UNIT GROUP 4412 FIRE AND EMERGENCY WORKERS *continued*

441212 FIRE FIGHTER

Responds to fire alarms and emergency calls, controls and extinguishes fires, and protects life and property. Registration or licensing is required.

Skill Level: 3

Specialisations:
- Fire Engineer (Army)
- Fire Prevention Officer

UNIT GROUP 4413 POLICE

POLICE protect and preserve property, public order and safety through the enforcement of laws.

Indicative Skill Level:
Most occupations in this unit group have a level of skill commensurate with the qualifications and experience outlined below.

In Australia:
AQF Associate Degree, Advanced Diploma or Diploma (ANZSCO Skill Level 2)

In New Zealand:
NZ Register Diploma (ANZSCO Skill Level 2)

At least three years of relevant experience may substitute for the formal qualifications listed above. In some instances relevant experience and/or on-the-job training may be required in addition to the formal qualification.

Tasks Include:
- investigating and prosecuting offences committed in areas such as organised, corporate and computer crime, environmental offences, drug trafficking, fraud, counterfeiting and terrorism
- securing and examining scenes of crimes and accidents to locate and obtain evidence for analysis
- protecting witnesses and investigating official corruption
- maintaining public order and safety
- patrolling assigned areas to minimise potential for public disturbance and crime
- investigating accidents, crimes, minor offences and citizens' complaints, gathering evidence, and pursuing, arresting and interviewing suspected offenders
- testing persons suspected of driving under the influence of alcohol and drugs and issuing infringement notices for traffic offences
- directing and re-routing traffic at congested areas
- attending community meetings and answering inquiries from the public where necessary
- providing advice and assistance to victims of crime and their families
- maintaining records and preparing reports

Occupations:
441311 Detective
441312 Police Officer

441311 DETECTIVE

Alternative Title:
Plain Clothes Police Officer

Investigates serious crimes, such as terrorism, homicide, armed robbery, vice and arson, and gathers evidence to arrest and prosecute suspected offenders.

Skill Level: 2

Specialisation:
Detective Sergeant

UNIT GROUP 4413 POLICE *continued*

441312 POLICE OFFICER

Maintains public order, and enforces laws by investigating crimes, patrolling public areas and arresting suspected offenders.

Skill Level: 2

Specialisations:
- Bomb Squad Officer
- Mounted Police Officer
- Search and Rescue Officer
- Tactical Response Group Officer

MINOR GROUP 442 PRISON AND SECURITY OFFICERS

PRISON AND SECURITY OFFICERS supervise and control the activities of inmates in correctional institutions, and provide security and investigative services to organisations and individuals.

Indicative Skill Level:
Most occupations in this minor group have a level of skill commensurate with the qualifications and experience outlined below.

In Australia:

AQF Certificate III including at least two years of on-the-job training, or AQF Certificate IV, or at least three years of relevant experience (ANZSCO Skill Level 3); or
AQF Certificate II or III, or at least one year of relevant experience (ANZSCO Skill Level 4); or
AQF Certificate I, or compulsory secondary education (ANZSCO Skill Level 5)

In New Zealand:

NZ Register Level 4 qualification, or at least three years of relevant experience (ANZSCO Skill Level 3); or
NZ Register Level 2 or 3 qualification, or at least one year of relevant experience (ANZSCO Skill Level 4); or
NZ Register Level 1 qualification, or compulsory secondary education (ANZSCO Skill Level 5)

In some instances relevant experience and/or on-the-job training may be required in addition to the formal qualification. In the case of some Skill Level 5 occupations, a short period of on-the-job training may be required in addition to or instead of the formal qualification, or no formal qualification or on-the-job training may be required.

Tasks Include:

- observing the conduct and behaviour of prisoners to prevent disturbances and escapes
- supervising prisoners during work assignments, recreational periods, sporting activities and meals
- patrolling and guarding properties, and checking for unauthorised entry
- providing armed escort for the transport of cash and other valuables
- maintaining order at venues where there are large gatherings of people
- conducting investigations for clients
- advising clients on security requirements and recommending and designing security specifications

Occupations in this minor group are classified into the following unit groups:

4421 Prison Officers
4422 Security Officers and Guards

UNIT GROUP 4421 PRISON OFFICERS

PRISON OFFICERS supervise and control the activities of inmates in prisons and other correctional institutions.

Indicative Skill Level:
Most occupations in this unit group have a level of skill commensurate with the qualifications and experience outlined below.

In Australia:
AQF Certificate II or III (ANZSCO Skill Level 4)

In New Zealand:
NZ Register Level 2 or 3 qualification (ANZSCO Skill Level 4)

At least one year of relevant experience may substitute for the formal qualifications listed above. In some instances relevant experience and/or on-the-job training may be required in addition to the formal qualification.

Tasks Include:
- observing the conduct and behaviour of prisoners to prevent disturbances and escapes
- inspecting and maintaining the security of locks, window bars, grilles, doors and gates
- supervising prisoners during work assignments, recreational periods, sporting activities and meals
- assisting with the implementation of education, rehabilitation and other programs organised for prisoners
- searching prisoners and cells for weapons, drugs and other contraband items
- patrolling assigned areas and reporting breaches of rules, unsatisfactory attitudes and prisoner adjustment problems
- requisitioning prisoners' clothing, toiletries, reading material and other allowable items
- supervising prisoners in transit between courts, prisons and other facilities

Occupation:
442111 Prison Officer

442111 PRISON OFFICER

Alternative Title:
Custodial Officer

Supervises and controls the activities of inmates in a prison or other correctional institution.

Skill Level: 4

UNIT GROUP 4422 SECURITY OFFICERS AND GUARDS

SECURITY OFFICERS AND GUARDS provide security and investigative services to organisations and individuals.

Indicative Skill Level:
Most occupations in this unit group have a level of skill commensurate with the qualifications and experience outlined below.

In Australia:
AQF Certificate I, or compulsory secondary education (ANZSCO Skill Level 5)

In New Zealand:
NZ Register Level 1 qualification, or compulsory secondary education (ANZSCO Skill Level 5)

For some occupations a short period of on-the-job training may be required in addition to or instead of the formal qualification. In some instances no formal qualification or on-the-job training may be required.

Registration or licensing may be required.

The occupation Security Consultant has a level of skill commensurate with the qualifications and experience outlined below.

In Australia:
AQF Certificate III including at least two years of on-the-job training, or AQF Certificate IV (ANZSCO Skill Level 3)

In New Zealand:
NZ Register Level 4 qualification (ANZSCO Skill Level 3)

At least three years of relevant experience may substitute for the formal qualifications listed above. In some instances relevant experience and/or on-the-job training may be required in addition to the formal qualification.

Registration or licensing may be required.

The occupations Crowd Controller and Private Investigator have a level of skill commensurate with the qualifications and experience outlined below.

In Australia:
AQF Certificate II or III (ANZSCO Skill Level 4)

In New Zealand:
NZ Register Level 2 or 3 qualification (ANZSCO Skill Level 4)

At least one year of relevant experience may substitute for the formal qualifications listed above. In some instances relevant experience and/or on-the-job training may be required in addition to the formal qualification.

Registration or licensing may be required.

UNIT GROUP 4422 SECURITY OFFICERS AND GUARDS *continued*

Tasks Include:

- patrolling property and checking doors, windows and gates for unauthorised entry
- watching for irregularities such as fire hazards, malfunctions of machines and equipment, lights left on, leaking water pipes and unlocked security doors
- issuing security passes to authorised visitors and giving directions
- monitoring alarms and contacting supervisors, police and fire brigades by radio or phone if security is breached or fire is detected
- picking up and ensuring the safe delivery of cash, payrolls and valuables
- operating coin and currency counting machines, and carrying out cash counting and packaging functions
- maintaining order at venues where there are large gatherings of people
- conducting investigations for clients and preparing evidence for court proceedings
- detecting and investigating theft and other unlawful acts carried out in retail establishments
- advising clients on security requirements and designing security specifications

Occupations:

442211 Alarm, Security or Surveillance Monitor
442212 Armoured Car Escort
442213 Crowd Controller
442214 Private Investigator
442215 Retail Loss Prevention Officer
442216 Security Consultant
442217 Security Officer
442299 Security Officers and Guards nec

442211 ALARM, SECURITY OR SURVEILLANCE MONITOR

Monitors security alarms and surveillance equipment, and contacts supervisors, police or fire brigades if security is breached or fire is detected. Registration or licensing may be required.

Skill Level: 5

442212 ARMOURED CAR ESCORT

Provides armed escort for transportation and delivery of cash and other valuables. Registration or licensing may be required.

Skill Level: 5

442213 CROWD CONTROLLER

Alternative Title:

Bouncer

Carries out crowd control duties at entertainment, sporting or recreational venues. Registration or licensing may be required.

Skill Level: 4

UNIT GROUP 4422 SECURITY OFFICERS AND GUARDS *continued*

442214 PRIVATE INVESTIGATOR

Alternative Title:

Private Inquiry Agent

Conducts investigations for clients and prepares evidence for court proceedings. Registration or licensing is required.

Skill Level: 4

442215 RETAIL LOSS PREVENTION OFFICER

Detects and investigates shoplifting, fraud and other unlawful acts of employees or customers of a retail establishment. Registration or licensing may be required.

Skill Level: 5

442216 SECURITY CONSULTANT

Advises clients on security requirements, and recommends and designs security specifications. Registration or licensing may be required.

Skill Level: 3

442217 SECURITY OFFICER

Alternative Title:

Security Guard

Patrols and guards industrial and commercial property, railway yards, stations and other facilities. Registration or licensing may be required.

Skill Level: 5

Specialisations:

Mobile Patrol Officer
Railway Patrol Officer

442299 SECURITY OFFICERS AND GUARDS NEC

This occupation group covers Security Officers and Guards not elsewhere classified. Registration or licensing may be required.

Skill Level: 5

Occupations in this group include:

Bodyguard

SUB-MAJOR GROUP 45 SPORTS AND PERSONAL SERVICE WORKERS

SPORTS AND PERSONAL SERVICE WORKERS participate in, and instruct people in, sports and fitness, and provide travel, tourism and other personal services to individuals.

Indicative Skill Level:
Most occupations in this sub-major group have a level of skill commensurate with the qualifications and experience outlined below.

In Australia:
AQF Associate Degree, Advanced Diploma or Diploma, or at least three years of relevant experience (ANZSCO Skill Level 2); or
AQF Certificate III including at least two years of on-the-job training, or AQF Certificate IV, or at least three years of relevant experience (ANZSCO Skill Level 3); or
AQF Certificate II or III, or at least one year of relevant experience (ANZSCO Skill Level 4); or
AQF Certificate I, or compulsory secondary education (ANZSCO Skill Level 5)

In New Zealand:
NZ Register Diploma, or at least three years of relevant experience (ANZSCO Skill Level 2); or
NZ Register Level 4 qualification, or at least three years of relevant experience (ANZSCO Skill Level 3); or
NZ Register Level 2 or 3 qualification, or at least one year of relevant experience (ANZSCO Skill Level 4); or
NZ Register Level 1 qualification, or compulsory secondary education (ANZSCO Skill Level 5)

In some instances relevant experience and/or on-the-job training may be required in addition to the formal qualification. In the case of some Skill Level 5 occupations, a short period of on-the-job training may be required in addition to or instead of the formal qualification, or no formal qualification or on-the-job training may be required.

Tasks Include:
- providing beauty therapy and personal care services
- instructing individuals and groups in the theory and application of driving
- preparing bodies for burial and arranging and conducting funerals
- escorting people on tours
- planning, organising and providing advice about travel and accommodation for clients
- providing services for the safety and comfort of passengers in aircraft, ships and railway sleeping cars
- delivering group exercise classes and one-to-one tuition in a variety of fitness activities
- organising and supervising groups involved in outdoor adventures such as bungy jumping, fishing and hunting, mountaineering, trekking and whitewater rafting
- participating in and officiating at sporting competitions, and coaching and training sporting competitors
- promoting sports and sports skill development

Occupations in this sub-major group are classified into the following minor groups:
451 Personal Service and Travel Workers
452 Sports and Fitness Workers

MINOR GROUP 451 PERSONAL SERVICE AND TRAVEL WORKERS

PERSONAL SERVICE AND TRAVEL WORKERS provide beauty and personal care services, travel and tourism services, driving instruction, and funereal and other personal services to clients.

Indicative Skill Level:
Most occupations in this minor group have a level of skill commensurate with the qualifications and experience outlined below.

In Australia:
AQF Associate Degree, Advanced Diploma or Diploma, or at least three years of relevant experience (ANZSCO Skill Level 2); or
AQF Certificate III including at least two years of on-the-job training, or AQF Certificate IV, or at least three years of relevant experience (ANZSCO Skill Level 3); or
AQF Certificate II or III, or at least one year of relevant experience (ANZSCO Skill Level 4); or
AQF Certificate I, or compulsory secondary education (ANZSCO Skill Level 5)

In New Zealand:
NZ Register Diploma, or at least three years of relevant experience (ANZSCO Skill Level 2); or
NZ Register Level 4 qualification, or at least three years of relevant experience (ANZSCO Skill Level 3); or
NZ Register Level 2 or 3 qualification, or at least one year of relevant experience (ANZSCO Skill Level 4); or
NZ Register Level 1 qualification, or compulsory secondary education (ANZSCO Skill Level 5)

In some instances relevant experience and/or on-the-job training may be required in addition to the formal qualification. In the case of some Skill Level 5 occupations, a short period of on-the-job training may be required in addition to or instead of the formal qualification, or no formal qualification or on-the-job training may be required.

Tasks Include:
- providing beauty therapy and personal care services
- instructing individuals and groups in the theory and application of driving
- preparing bodies for burial and arranging and conducting funerals
- conducting civil marriage and other ceremonies
- escorting people on tours
- planning and organising travel and accommodation for clients
- providing travel and accommodation advice
- providing services for the safety and comfort of passengers in aircraft, ships and railway sleeping cars
- providing sexual services and social companionship to clients

Occupations in this minor group are classified into the following unit groups:
4511 Beauty Therapists
4512 Driving Instructors
4513 Funeral Workers
4514 Gallery, Museum and Tour Guides
4515 Personal Care Consultants
4516 Tourism and Travel Advisers
4517 Travel Attendants
4518 Other Personal Service Workers

UNIT GROUP 4511 BEAUTY THERAPISTS

BEAUTY THERAPISTS provide skin analyses, facial therapies, skin-care treatments and body treatments such as massage to clients.

Indicative Skill Level:
Most occupations in this unit group have a level of skill commensurate with the qualifications and experience outlined below.

In Australia:
AQF Certificate II or III (ANZSCO Skill Level 4)

In New Zealand:
NZ Register Level 2 or 3 qualification (ANZSCO Skill Level 4)

At least one year of relevant experience may substitute for the formal qualifications listed above. In some instances relevant experience and/or on-the-job training may be required in addition to the formal qualification.

Tasks Include:
- discussing client needs, analysing skin characteristics and advising on suitable skin care, treatments and application of make-up
- applying general cosmetic and corrective make-up
- performing manicures and pedicures including decorative nail art, application of artificial nails, nail repair, and other specialised hand and foot treatments
- performing facial and body treatments such as massages
- treating unwanted hair through waxing, bleaching, tinting, depilation and electrolysis
- evaluating beauty therapy processes and products
- receiving bookings, arranging appointments and maintaining client records
- providing advice on and selling cosmetic products

Occupation:
451111 Beauty Therapist

451111 BEAUTY THERAPIST

Provides skin analyses, facial therapies, skin-care treatments and body treatments such as massage to clients.

Skill Level: 4

Specialisations:
Electrologist (Hair Remover)
Manicurist
Nail Technician

UNIT GROUP 4512 DRIVING INSTRUCTORS

DRIVING INSTRUCTORS instruct individuals and groups in the theory and application of driving motor vehicles.

Indicative Skill Level:
Most occupations in this unit group have a level of skill commensurate with the qualifications and experience outlined below.

In Australia:
AQF Certificate III including at least two years of on-the-job training, or AQF Certificate IV (ANZSCO Skill Level 3)

In New Zealand:
NZ Register Level 4 qualification (ANZSCO Skill Level 3)

At least three years of relevant experience may substitute for the formal qualifications listed above. In some instances relevant experience and/or on-the-job training may be required in addition to the formal qualification.

Registration or licensing is required.

Tasks Include:

- instructing students under actual driving conditions, and explaining and demonstrating the operation of brakes, clutch, gear selection, automatic transmission, signals and lights
- teaching road traffic regulations
- teaching road craft and road safety
- advising students when they are ready to undergo driving examination
- may advise on and teach advanced driving techniques required for emergency situations
- may illustrate and explain handling and mechanical operation of motor vehicles and driving techniques using blackboard diagrams and audiovisual aids

Occupation:
451211 Driving Instructor

451211 DRIVING INSTRUCTOR

Instructs individuals and groups in the theory and application of driving motor vehicles. Registration or licensing is required.

Skill Level: 3

Specialisation:
Motorcycle Riding Instructor

UNIT GROUP 4513 FUNERAL WORKERS

FUNERAL WORKERS prepare bodies for viewing and burial, arrange and conduct funerals, and perform other specialist funereal services.

Indicative Skill Level:
The occupation Funeral Director has a level of skill commensurate with the qualifications and experience outlined below.

In Australia:
AQF Associate Degree, Advanced Diploma or Diploma (ANZSCO Skill Level 2)

In New Zealand:
NZ Register Diploma (ANZSCO Skill Level 2)

At least three years of relevant experience may substitute for the formal qualifications listed above. In some instances relevant experience and/or on-the-job training may be required in addition to the formal qualification.

Registration or licensing may be required.

The occupation Funeral Workers nec has a level of skill commensurate with the qualifications and experience outlined below.

In Australia:
AQF Certificate III including at least two years of on-the-job training, or AQF Certificate IV (ANZSCO Skill Level 3)

In New Zealand:
NZ Register Level 4 qualification (ANZSCO Skill Level 3)

At least three years of relevant experience may substitute for the formal qualifications listed above. In some instances relevant experience and/or on-the-job training may be required in addition to the formal qualification.

Registration or licensing may be required.

Tasks Include:
- interviewing families and associates of the deceased to assist with funeral arrangements such as the selection of coffin, type of service and publication of death notices
- advising on funeral costs and welfare provisions
- collecting bodies from mortuaries
- ensuring death certificates have been issued, burial and cremation certificates processed and that other legal requirements are met
- preparing bodies for viewing and burial by washing, draining body fluids, applying padding and cosmetics, dressing bodies and placing them in coffins
- liaising with clergy and cemetery and crematorium staff
- coordinating the movement of coffins and funeral cars, arranging floral displays and collecting attendance and tribute cards
- arranging the placement of coffins at funeral sites, and placing and adjusting floral displays and lighting
- keeping records and accounts of transactions and services performed
- may arrange the construction of memorials and the disposal of ashes

Occupations:
451311 Funeral Director
451399 Funeral Workers nec

UNIT GROUP 4513 FUNERAL WORKERS *continued*

451311 FUNERAL DIRECTOR

Alternative Titles:
- Mortician
- Undertaker

Plans and coordinates arrangements for funerals according to the wishes of the deceased or their relatives. Registration or licensing may be required.

Skill Level: 2

451399 FUNERAL WORKERS NEC

This occupation group covers Funeral Workers not elsewhere classified. Registration or licensing may be required.

Skill Level: 3

Occupations in this group include:
- Chapel or Memorial Attendant
- Embalmer
- Funeral Director's Assistant

UNIT GROUP 4514 GALLERY, MUSEUM AND TOUR GUIDES

GALLERY, MUSEUM AND TOUR GUIDES direct and guide visitors in galleries and museums, and escort visitors on sightseeing, educational and other tours.

Indicative Skill Level:
Most occupations in this unit group have a level of skill commensurate with the qualifications and experience outlined below.

In Australia:
AQF Certificate II or III (ANZSCO Skill Level 4)

In New Zealand:
NZ Register Level 2 or 3 qualification (ANZSCO Skill Level 4)

At least one year of relevant experience may substitute for the formal qualifications listed above. In some instances relevant experience and/or on-the-job training may be required in addition to the formal qualification.

Tasks Include:
- meeting and greeting visitors
- controlling visitors' access to exhibits
- ensuring safety of collections
- maintaining attendance records
- planning and rearranging schedules and itineraries
- planning, organising and conducting tours
- arranging transportation and accommodation for visitors following planned itineraries
- arranging entry to places of interest
- answering questions, providing commentaries, issuing brochures and tour literature, showing audiovisual presentations, and explaining features and procedures at tour sites
- may assist with installing and dismantling exhibits

Occupations:
451411 Gallery or Museum Guide
451412 Tour Guide

451411 GALLERY OR MUSEUM GUIDE

Alternative Title:
Gallery or Museum Attendant

Answers inquires and directs and guides visitors in a gallery or museum.

Skill Level: 4

451412 TOUR GUIDE

Alternative Titles:
Tour Escort
Tour Leader

Escorts visitors on sightseeing, educational and other tours, and describes and explains points of interest.

Skill Level: 4

Specialisation:
Regional Guide

UNIT GROUP 4515 PERSONAL CARE CONSULTANTS

PERSONAL CARE CONSULTANTS provide personal care services, such as natural relaxation and health treatments, and weight loss advice.

Indicative Skill Level:
Most occupations in this unit group have a level of skill commensurate with the qualifications and experience outlined below.

In Australia:
AQF Certificate II or III (ANZSCO Skill Level 4)

In New Zealand:
NZ Register Level 2 or 3 qualification (ANZSCO Skill Level 4)

At least one year of relevant experience may substitute for the formal qualifications listed above. In some instances relevant experience and/or on-the-job training may be required in addition to the formal qualification.

Tasks Include:
- interviewing clients to work out their needs
- treating emotional, psychological and physical imbalances of the body using natural techniques and diagnostic methods
- monitoring and correcting imbalances in the body using muscle testing techniques
- advising clients on dietary requirements and exercise programs
- recording clients' weight and measurements
- instructing clients on the use of exercise equipment
- providing support and counselling

Occupations:
451511 Natural Remedy Consultant
451512 Weight Loss Consultant

451511 NATURAL REMEDY CONSULTANT

Uses natural techniques and diagnostic methods for treatment, relaxation and health purposes.

Skill Level: 4

Specialisations:
Aromatherapist
Herbalist (Western)
Holistic Pulser
Iridologist
Kinesiologist
Reflexologist

451512 WEIGHT LOSS CONSULTANT

Assists clients with advice and practical solutions for losing weight or body fat.

Skill Level: 4

UNIT GROUP 4516 TOURISM AND TRAVEL ADVISERS

TOURISM AND TRAVEL ADVISERS plan and organise travel and accommodation for clients, and provide travel and accommodation information to tourists.

Indicative Skill Level:
Most occupations in this unit group have a level of skill commensurate with the qualifications and experience outlined below.

In Australia:
AQF Certificate II or III (ANZSCO Skill Level 4)

In New Zealand:
NZ Register Level 2 or 3 qualification (ANZSCO Skill Level 4)

At least one year of relevant experience may substitute for the formal qualifications listed above. In some instances relevant experience and/or on-the-job training may be required in addition to the formal qualification.

Tasks Include:
- determining clients' requirements for travel, accommodation and special interests
- suggesting itineraries based on available travel routes and cost, availability and convenience of transport
- making and confirming travel and accommodation reservations and informing clients of bus, plane, ship and train connections
- notifying clients of travel dates, baggage limits, and medical and visa requirements
- providing information on tourist attractions and tour availability, and procedures for dealing with lost and stolen documents
- assisting with travel clearances
- collecting payments and issuing clients' itineraries, relevant documentation, tickets for travel and vouchers for accommodation
- providing information on travel insurance, relevant government regulations such as customs regulations, and use of credit cards and traveller's cheques
- answering inquiries from tourists and offering suggestions about tours, travel routes, accommodation and local customs
- providing literature and information on local and interstate tours and places of interest
- discussing transport availability and cost
- may work in a call centre

Occupations:
451611 Tourist Information Officer
451612 Travel Consultant

451611 TOURIST INFORMATION OFFICER

Alternative Title:
Tourist Adviser

Provides travel and accommodation information to tourists. May work in a call centre.

Skill Level: 4

UNIT GROUP 4516 TOURISM AND TRAVEL ADVISERS *continued*

451612 TRAVEL CONSULTANT

Alternative Title:

Travel Agent

Plans travel, accommodation and associated arrangements for clients and makes travel bookings. May work in a call centre.

Skill Level: 4

Specialisations:

Business Travel Consultant
Domestic Travel Consultant
International Travel Consultant

UNIT GROUP 4517 TRAVEL ATTENDANTS

TRAVEL ATTENDANTS provide services for the safety and comfort of passengers in aircraft, ships and railway sleeping cars.

Indicative Skill Level:
Most occupations in this unit group have a level of skill commensurate with the qualifications and experience outlined below.

In Australia:
AQF Certificate III including at least two years of on-the-job training, or AQF Certificate IV (ANZSCO Skill Level 3)

In New Zealand:
NZ Register Level 4 qualification (ANZSCO Skill Level 3)

At least three years of relevant experience may substitute for the formal qualifications listed above. In some instances relevant experience and/or on-the-job training may be required in addition to the formal qualification.

Tasks Include:
- managing safety and emergency procedures and making public announcements
- coordinating the sale of goods to passengers and completion of any customs and immigration documentation which may be required
- conducting safety checks and demonstrations of safety equipment and procedures
- assisting passengers in emergency drills, carrying out emergency procedures, assisting and directing passengers in emergencies
- checking passengers' tickets and directing them to seats and cabins
- tidying aircraft, ship and railway cabins, and receiving and stowing food, equipment and cabin baggage
- operating galleys, preparing and heating food for passengers, and serving refreshments and meals
- distributing reading material, pillows, blankets and other amenities for the comfort of passengers

Occupations:
451711 Flight Attendant
451799 Travel Attendants nec

451711 FLIGHT ATTENDANT

Provides services for the safety and comfort of aircraft passengers.

Skill Level: 3

Specialisations:
Cabin Supervisor (Aircraft)
Crew Attendant (Air Force)

451799 TRAVEL ATTENDANTS NEC

This occupation group covers Travel Attendants not elsewhere classified.

Skill Level: 3

Occupations in this group include:
Cabin Steward
Marine Steward
Railway Sleeping Car Conductor
Railway Steward

UNIT GROUP 4518 OTHER PERSONAL SERVICE WORKERS

This unit group covers Personal Service Workers not elsewhere classified.

It includes Civil Celebrants, Hair or Beauty Salon Assistants and Sex Workers or Escorts.

Indicative Skill Level:
The occupations Civil Celebrant and Personal Service Workers nec have a level of skill commensurate with the qualifications and experience outlined below.

In Australia:
AQF Certificate II or III (ANZSCO Skill Level 4)

In New Zealand:
NZ Register Level 2 or 3 qualification (ANZSCO Skill Level 4)

At least one year of relevant experience may substitute for the formal qualifications listed above. In some instances relevant experience and/or on-the-job training may be required in addition to the formal qualification.

Registration or licensing may be required.

The occupations Hair or Beauty Salon Assistant and Sex Worker or Escort have a level of skill commensurate with the qualifications outlined below.

In Australia:
AQF Certificate I, or compulsory secondary education (ANZSCO Skill Level 5)

In New Zealand:
NZ Register Level 1 qualification, or compulsory secondary education (ANZSCO Skill Level 5)

For some occupations a short period of on-the-job training may be required in addition to or instead of the formal qualification. In some instances no formal qualification or on-the-job training may be required.

Occupations:
451811 Civil Celebrant
451812 Hair or Beauty Salon Assistant
451813 Sex Worker or Escort
451899 Personal Service Workers nec

451811 CIVIL CELEBRANT

Conducts civil marriage ceremonies, funerals, commitment ceremonies, namings and other ceremonies, and maintains appropriate records. Registration or licensing is required.

Skill Level: 4

451812 HAIR OR BEAUTY SALON ASSISTANT

Assists Hairdressers or Beauty Therapists by performing routine tasks in a hairdressing or beauty salon.

Skill Level: 5

UNIT GROUP 4518 OTHER PERSONAL SERVICE WORKERS *continued*

451813 SEX WORKER OR ESCORT

Alternative Title:

Prostitute

Provides clients with sexual services or social companionship.

Skill Level: 5

Specialisations:

Dominatrix

Telephone Sex Worker

451899 PERSONAL SERVICE WORKERS NEC

This occupation group covers Personal Service Workers not elsewhere classified.

Skill Level: 4

Occupations in this group include:

Astrologer

Blood Bank Aide

Bus Escort

Butler

Dog Walker

First Aid Attendant

Gymnasium Attendant

Horse Racing Analyst

Red Cross Aide

School Bus Warden

Tattoo Artist

MINOR GROUP 452 SPORTS AND FITNESS WORKERS

SPORTS AND FITNESS WORKERS direct, instruct and guide individuals and groups in physical fitness and outdoor adventure activities, coach, train and instruct sporting competitors, and participate in and officiate at sporting events.

Indicative Skill Level:
Most occupations in this minor group have a level of skill commensurate with the qualifications and experience outlined below.

In Australia:
- AQF Associate Degree, Advanced Diploma or Diploma, or at least three years of relevant experience (ANZSCO Skill Level 2); or
- AQF Certificate III including at least two years of on-the-job training, or AQF Certificate IV, or at least three years of relevant experience (ANZSCO Skill Level 3); or
- AQF Certificate II or III, or at least one year of relevant experience (ANZSCO Skill Level 4)

In New Zealand:
- NZ Register Diploma, or at least three years of relevant experience (ANZSCO Skill Level 2); or
- NZ Register Level 4 qualification, or at least three years of relevant experience (ANZSCO Skill Level 3); or
- NZ Register Level 2 or 3 qualification, or at least one year of relevant experience (ANZSCO Skill Level 4)

In some instances relevant experience and/or on-the-job training may be required in addition to the formal qualification.

Tasks Include:
- developing and designing fitness programs
- delivering group exercise classes and personal tuition in a variety of fitness activities
- organising and supervising groups involved in outdoor adventures such as bungy jumping, fishing and hunting, mountaineering, trekking and whitewater rafting
- demonstrating and providing instruction in the use of outdoor adventure equipment and techniques required for participation
- coaching, training and instructing sportspersons by analysing performances and developing abilities
- planning and directing game strategies, developing play patterns, analysing game progress and motivating players
- promoting sports and sports skills development, and overseeing the participation of young people in sport
- officiating at sporting events to enforce rules
- coordinating and directing sporting activities, and liaising with other officials to interpret and enforce rules and regulations relating to sport
- competing in and training for sporting events

Occupations in this minor group are classified into the following unit groups:

4521 Fitness Instructors
4522 Outdoor Adventure Guides
4523 Sports Coaches, Instructors and Officials
4524 Sportspersons

UNIT GROUP 4521 FITNESS INSTRUCTORS

FITNESS INSTRUCTORS direct, instruct and guide individuals and groups in the pursuit of physical fitness and wellbeing.

Indicative Skill Level:
Most occupations in this unit group have a level of skill commensurate with the qualifications and experience outlined below.

In Australia:
AQF Certificate II or III (ANZSCO Skill Level 4)

In New Zealand:
NZ Register Level 2 or 3 qualification (ANZSCO Skill Level 4)

At least one year of relevant experience may substitute for the formal qualifications listed above. In some instances relevant experience and/or on-the-job training may be required in addition to the formal qualification.

Tasks Include:
- consulting with various Health Professionals to develop and design fitness programs
- designing individual fitness programs based on assessment of the client's age, level of fitness, goals and abilities
- delivering group exercise classes and personal tuition in a variety of fitness activities in a safe and creative manner
- demonstrating and teaching body movements and skills used in fitness routines
- setting up and monitoring fitness equipment and ensuring that equipment is safe, clean and in working condition
- teaching and advising on the use of fitness equipment
- ensuring clients are aware of and adhere to safety and injury prevention procedures
- reporting accidents and preparing accident reports
- maintaining a working knowledge of current health and safety standards and ensuring working practices and procedures conform to current legislation
- maintaining current first aid certificates

Occupation:
452111 Fitness Instructor

452111 FITNESS INSTRUCTOR

Directs, instructs and guides individuals or groups in the pursuit of physical fitness and wellbeing.

Skill Level: 4

Specialisations:
Aerobics Instructor
Gym Instructor
Physical Fitness Trainer

UNIT GROUP 4522 OUTDOOR ADVENTURE GUIDES

OUTDOOR ADVENTURE GUIDES direct, instruct and guide individuals and groups in outdoor adventure activities such as bungy jumping, fishing and hunting, mountaineering, trekking and whitewater rafting.

Indicative Skill Level:
Most occupations in this unit group have a level of skill commensurate with the qualifications and experience outlined below.

In Australia:
AQF Certificate II or III (ANZSCO Skill Level 4)

In New Zealand:
NZ Register Level 2 or 3 qualification (ANZSCO Skill Level 4)

At least one year of relevant experience may substitute for the formal qualifications listed above. In some instances relevant experience and/or on-the-job training may be required in addition to the formal qualification.

Tasks Include:

- meeting members of a tour on arrival and making introductions
- organising and supervising groups involved in outdoor adventures such as bungy jumping, fishing and hunting, mountaineering, trekking and whitewater rafting
- setting up and maintaining equipment, and ensuring that equipment is safe and in working condition
- demonstrating and providing instruction in the use of equipment and techniques required for participation
- providing advice on safety measures, and ensuring that activities are conducted in a manner to minimise risk to participants
- responding to emergencies by providing first aid assistance and taking appropriate further action if required
- answering questions and advising on local interest points within a specific region
- may maintain written reports of daily activities and carry out other administrative work

Occupations:
452211 Bungy Jump Master
452212 Fishing Guide
452213 Hunting Guide
452214 Mountain or Glacier Guide
452215 Outdoor Adventure Instructor
452216 Trekking Guide
452217 Whitewater Rafting Guide
452299 Outdoor Adventure Guides nec

452211 BUNGY JUMP MASTER

Directs, supervises and controls bungy jumping activities for individuals.

Skill Level: 4

UNIT GROUP 4522 OUTDOOR ADVENTURE GUIDES *continued*

452212 FISHING GUIDE

Plans, organises and provides guided fishing trips for individuals or groups.

Skill Level: 4

Specialisations:
- Fly Fishing Guide
- Ocean Fishing Guide

452213 HUNTING GUIDE

Plans, organises and provides guided hunting trips for individuals or groups.

Skill Level: 4

452214 MOUNTAIN OR GLACIER GUIDE

Plans, organises and provides guided trips for individuals or groups on mountains or glaciers.

Skill Level: 4

Specialisations:
- Climbing Guide
- Ski Guide

452215 OUTDOOR ADVENTURE INSTRUCTOR

Alternative Title:
- Outdoor Adventure Leader

Provides adventure-based experiential education in outdoor adventure and bushcraft.

Skill Level: 4

Specialisations:
- Abseiling Instructor
- Adventure Challenge Instructor
- Hang-gliding Instructor
- Outdoor Education Teacher
- Outdoor Pursuits Instructor
- Paragliding Instructor
- Rock Climbing Instructor

452216 TREKKING GUIDE

Alternative Title:
- Bushwalking Guide

Plans, organises and provides guided bushwalking and trekking trips for individuals or groups.

Skill Level: 4

452217 WHITEWATER RAFTING GUIDE

Plans, organises and provides guided rafting and kayaking trips for individuals or groups on whitewater rivers.

Skill Level: 4

UNIT GROUP 4522 OUTDOOR ADVENTURE GUIDES *continued*

452299 OUTDOOR ADVENTURE GUIDES NEC

This occupation group covers Outdoor Adventure Guides not elsewhere classified.

Skill Level: 4

Occupations in this group include:

- Caving Guide
- Cycle Touring Guide
- Horse Trekking Guide
- Sea Kayaking Guide

UNIT GROUP 4523 SPORTS COACHES, INSTRUCTORS AND OFFICIALS

SPORTS COACHES, INSTRUCTORS AND OFFICIALS coach, train and instruct participants in sports, and officiate at sporting events.

Indicative Skill Level:
Most occupations in this unit group have a level of skill commensurate with the qualifications and experience outlined below.

In Australia:
AQF Certificate III including at least two years of on-the-job training, or AQF certificate IV (ANZSCO Skill Level 3)

In New Zealand:
NZ Register Level 4 qualification (ANZSCO Skill Level 3)

At least three years of relevant experience may substitute for the formal qualifications listed above. In some instances relevant experience and/or on-the-job training may be required in addition to the formal qualification.

Registration or licensing may be required.

The occupation Sports Development Officer has a level of skill commensurate with the qualifications and experience outlined below.

In Australia:
AQF Associate Degree, Advanced Diploma or Diploma (ANZSCO Skill Level 2)

In New Zealand:
NZ Register Diploma (ANZSCO Skill Level 2)

At least three years of relevant experience may substitute for the formal qualifications listed above. In some instances relevant experience and/or on-the-job training may be required in addition to the formal qualification.

Registration or licensing is required.

Tasks Include:
- coaching, training and instructing sportspersons by analysing performances and developing abilities
- planning and directing game strategies, developing play patterns and analysing game progress
- motivating Sportspersons and supervising practice sessions
- recruiting players and other coaching staff
- arranging entries into sporting competitions
- promoting sports and skills development, and overseeing the participation of young people in sport
- officiating at sporting events to enforce rules
- coordinating and directing sporting activities, and liaising with other officials to interpret and enforce rules and regulations relating to sport

UNIT GROUP 4523 SPORTS COACHES, INSTRUCTORS AND OFFICIALS *continued*

Occupations:

452311 Diving Instructor (Open Water)
452312 Gymnastics Coach or Instructor
452313 Horse Riding Coach or Instructor
452314 Snowsport Instructor
452315 Swimming Coach or Instructor
452316 Tennis Coach
452317 Other Sports Coach or Instructor
452318 Dog and Horse Racing Official
452321 Sports Development Officer
452322 Sports Umpire
452323 Other Sports Official

452311 DIVING INSTRUCTOR (OPEN WATER)

Trains and instructs recreational or commercial open water divers in diving techniques, safety and the correct use of diving equipment. Registration or licensing is required.

Skill Level: 3

Specialisations:

Dive Master
Scuba Instructor
Snorkelling Instructor
Surface Supply Breathing Apparatus (SSBA) Instructor

452312 GYMNASTICS COACH OR INSTRUCTOR

Coaches, trains and instructs participants in gymnastics by analysing their performances and developing their abilities. Registration or licensing is required.

Skill Level: 3

Specialisations:

Callisthenics Instructor
Rhythmic Gymnastics Coach

452313 HORSE RIDING COACH OR INSTRUCTOR

Coaches, trains and instructs participants in horse riding by analysing their performances and developing their abilities. Registration or licensing is required.

Skill Level: 3

Specialisations:

Dressage Instructor
Polo Coach
Show Jumping Instructor

UNIT GROUP 4523 SPORTS COACHES, INSTRUCTORS AND OFFICIALS *continued*

452314 SNOWSPORT INSTRUCTOR

Coaches, trains and instructs participants in snow skiing, snowboarding or other snowsports by analysing their performances and developing their abilities. Registration or licensing is required.

Skill Level: 3

Specialisations:
- Skiing Instructor
- Snowboarding Instructor

452315 SWIMMING COACH OR INSTRUCTOR

Coaches, trains and instructs participants in swimming by analysing their performances and developing their abilities. Registration or licensing is required.

Skill Level: 3

Specialisations:
- Diving Coach
- Learn to Swim Instructor

452316 TENNIS COACH

Coaches, trains and instructs participants in tennis by analysing their performances and developing their abilities. Registration or licensing is required.

Skill Level: 3

452317 OTHER SPORTS COACH OR INSTRUCTOR

Coaches, trains and instructs participants in other sports by analysing their performances and developing their abilities. Registration or licensing may be required.

Skill Level: 3

Specialisations:
- Basketball Coach
- Cricket Coach
- Football Coach
- Sports Trainer
- Windsurfing Instructor

452318 DOG OR HORSE RACING OFFICIAL

Alternative Title:
- Race Steward

Coordinates and directs horse or dog racing activities, and liaises with other officials to interpret and enforce racing rules and regulations. Registration or licensing is required.

Skill Level: 3

Specialisation:
- Handicapper (Racing)

UNIT GROUP 4523 SPORTS COACHES, INSTRUCTORS AND OFFICIALS *continued*

452321 SPORTS DEVELOPMENT OFFICER

Promotes sports and skills development, and oversees the participation of young people and other special groups in sport. Registration or licensing is required.

Skill Level: 2

452322 SPORTS UMPIRE

Alternative Title:
Referee

Officiates at sporting events, such as netball, hockey, football, basketball, cricket, boxing and wrestling matches, by interpreting and enforcing match rules. Registration or licensing is required.

Skill Level: 3

Specialisation:
Linesperson (Sport)

452323 OTHER SPORTS OFFICIAL

Coordinates and directs sporting activities, and liaises with other officials to interpret and enforce sporting rules and regulations. Registration or licensing may be required.

Skill Level: 3

Specialisation:
Timekeeper (Sports)

UNIT GROUP 4524 SPORTSPERSONS

SPORTSPERSONS participate in sporting events for monetary gain either as individuals or as members of a team.

Indicative Skill Level:
Most occupations in this unit group have a level of skill commensurate with the qualifications and experience outlined below.

In Australia:
AQF Certificate III including at least two years of on-the-job training, or AQF Certificate IV (ANZSCO Skill Level 3)

In New Zealand:
NZ Register Level 4 qualification (ANZSCO Skill Level 3)

At least three years of relevant experience may substitute for the formal qualifications listed above. In some instances relevant experience and/or on-the-job training may be required in addition to the formal qualification.

Some occupations may require high levels of physical fitness, sporting ability and personal commitment as well as, or in place of, formal qualifications or experience.

Registration or licensing may be required.

Tasks Include:
- maintaining a high degree of expertise in a particular sport
- attending regular practice sessions and undertaking private training to maintain the required standard of fitness
- deciding on strategies in consultation with coaches
- assessing other competitors and conditions at venues
- competing in sporting events
- adhering to the rules and regulations associated with a specific sport
- promoting water safety awareness and undertaking rescue of persons in difficulty in the water
- undertaking sports promotional activities and television appearances

Occupations:
452411 Footballer
452412 Golfer
452413 Jockey
452414 Lifeguard
452499 Sportspersons nec

UNIT GROUP 4524 SPORTSPERSONS *continued*

452411 FOOTBALLER

Plays football professionally in competitions. This occupation requires high levels of physical fitness, sporting ability and personal commitment as well as, or in place of, formal qualifications or experience. Registration or licensing is required.

Skill Level: 3

Specialisations:

- Australian Rules Footballer
- Rugby League Footballer
- Rugby Union Footballer
- Soccer Player

452412 GOLFER

Plays golf professionally in tournaments or as a resident professional, and organises golf-related activities. This occupation requires high levels of physical fitness, sporting ability and personal commitment as well as, or in place of, formal qualifications or experience. Registration or licensing is required.

Skill Level: 3

452413 JOCKEY

Rides horses in competitive races, race trials, and in exercise. This occupation requires high levels of physical fitness, sporting ability and personal commitment as well as, or in place of, formal qualifications or experience. Registration or licensing is required.

Skill Level: 3

Specialisations:

- Apprentice Jockey
- Steeplechase Jockey

452414 LIFEGUARD

Looks after the safety of people at beaches or swimming pools through accident prevention and rescue, and educating the public on water safety. This occupation requires high levels of physical fitness, sporting ability and personal commitment as well as, or in place of, formal qualifications or experience. Registration or licensing is required.

Skill Level: 3

452499 SPORTSPERSONS NEC

This occupation groups covers Sportspersons not elsewhere classified. This occupation group requires high levels of physical fitness, sporting ability and personal commitment as well as, or in place of, formal qualifications or experience. Registration or licensing may be required.

Skill Level: 3

Occupations in this group include:

- Athlete
- Cricketer
- Cyclist
- Racing Driver
- Surfer
- Tennis Player

CLERICAL AND ADMINISTRATIVE WORKERS provide support to Managers, Professionals and organisations by organising, storing, manipulating and retrieving information.

Indicative Skill Level:
Most occupations in this major group have a level of skill commensurate with the qualifications and experience outlined below.

In Australia:
AQF Associate Degree, Advanced Diploma or Diploma, or at least three years of relevant experience (ANZSCO Skill Level 2); or
AQF Certificate III including at least two years of on-the-job training, or AQF Certificate IV, or at least three years of relevant experience (ANZSCO Skill Level 3); or
AQF Certificate II or III, or at least one year of relevant experience (ANZSCO Skill Level 4); or
AQF Certificate I, or compulsory secondary education (ANZSCO Skill Level 5)

In New Zealand:
NZ Register Diploma, or at least three years of relevant experience (ANZSCO Skill Level 2); or
NZ Register Level 4 qualification, or at least three years of relevant experience (ANZSCO Skill Level 3); or
NZ Register Level 2 or 3 qualification, or at least one year of relevant experience (ANZSCO Skill Level 4); or
NZ Register Level 1 qualification, or compulsory secondary education (ANZSCO Skill Level 5)

In some instances relevant experience and/or on-the-job training may be required in addition to the formal qualification. In the case of some Skill Level 5 occupations, a short period of on-the-job training may be required in addition to or instead of the formal qualification, or no formal qualification or on-the-job training may be required.

Tasks Include:
- administering contracts, programs and projects
- setting, reviewing and controlling office functions
- performing clerical, secretarial, organisational and other administrative functions
- entering, processing and editing text and data
- greeting clients and visitors, and responding to inquiries and requests for information
- producing, recording and evaluating financial, production, stock and statistical information
- receiving, processing and sending mail, documents and information

Occupations in this major group are classified into the following sub-major groups:
51 Office Managers and Program Administrators
52 Personal Assistants and Secretaries
53 General Clerical Workers
54 Inquiry Clerks and Receptionists
55 Numerical Clerks
56 Clerical and Office Support Workers
59 Other Clerical and Administrative Workers

SUB-MAJOR GROUP 51 OFFICE MANAGERS AND PROGRAM ADMINISTRATORS

OFFICE MANAGERS AND PROGRAM ADMINISTRATORS plan and undertake administration of organisational programs and projects, and organise and manage the activities of offices and practices.

Indicative Skill Level:
Most occupations in this sub-major group have a level of skill commensurate with the qualifications and experience outlined below.

In Australia:
AQF Associate Degree, Advanced Diploma or Diploma (ANZSCO Skill Level 2)

In New Zealand:
NZ Register Diploma (ANZSCO Skill Level 2)

At least three years of relevant experience may substitute for the formal qualifications listed above. In some instances relevant experience and/or on-the-job training may be required in addition to the formal qualification.

Tasks Include:
- coordinating activities to ensure that objectives of the organisation and office are met
- liaising with professionals, owners, other departments and personnel to ensure that goals are met
- advising senior management on matters requiring attention and implementing their decisions
- managing paperwork, records and information associated with undertaking projects and running offices and practices
- responding to inquiries concerning programs and services
- setting, reviewing and controlling office functions

Occupations in this sub-major group are classified into the following minor groups:
511 Contract, Program and Project Administrators
512 Office and Practice Managers

MINOR GROUP 511 CONTRACT, PROGRAM AND PROJECT ADMINISTRATORS

CONTRACT, PROGRAM AND PROJECT ADMINISTRATORS plan and undertake administration of contracts, organisational programs, special projects and support services.

Indicative Skill Level:
Most occupations in this minor group have a level of skill commensurate with the qualifications and experience outlined below.

In Australia:
AQF Associate Degree, Advanced Diploma or Diploma (ANZSCO Skill Level 2)

In New Zealand:
NZ Register Diploma (ANZSCO Skill Level 2)

At least three years of relevant experience may substitute for the formal qualifications listed above. In some instances relevant experience and/or on-the-job training may be required in addition to the formal qualification.

Tasks Include:

- negotiating, developing and reviewing contracts, programs, projects and services
- responding to inquiries and resolving problems concerning contracts, programs, projects, services provided, and persons affected
- managing paperwork associated with contracts, programs, projects and services provided
- working with Project Managers, Architects, Engineering Professionals, owners and others to ensure that goals are met
- advising senior management on matters requiring attention and implementing their decisions
- overseeing work by contractors and reporting on variations to work orders
- preparing and reviewing submissions and reports concerning the organisation's activities
- collecting and analysing data associated with projects undertaken, and reporting on project outcomes
- reviewing and arranging new office accommodation

Occupations in this minor group are classified into the following unit group:
5111 Contract, Program and Project Administrators

UNIT GROUP 5111 CONTRACT, PROGRAM AND PROJECT ADMINISTRATORS

CONTRACT, PROGRAM AND PROJECT ADMINISTRATORS plan and undertake administration of contracts, organisational programs, special projects and support services.

Indicative Skill Level:
Most occupations in this unit group have a level of skill commensurate with the qualifications and experience outlined below.

In Australia:
AQF Associate Degree, Advanced Diploma or Diploma (ANZSCO Skill Level 2)

In New Zealand:
NZ Register Diploma (ANZSCO Skill Level 2)

At least three years of relevant experience may substitute for the formal qualifications listed above. In some instances relevant experience and/or on-the-job training may be required in addition to the formal qualification.

Tasks Include:
- developing, reviewing and negotiating variations to contracts, programs, projects and services
- responding to inquiries and resolving problems concerning contracts, programs, projects, services provided, and persons affected
- managing paperwork associated with contracts, programs, projects and services provided
- working with Project Managers, Architects, Engineering Professionals, owners and others to ensure that goals are met
- advising senior management on matters requiring attention and implementing their decisions
- overseeing work by contractors and reporting on variations to work orders
- preparing and reviewing submissions and reports concerning the organisation's activities
- collecting and analysing data associated with projects undertaken, and reporting on project outcomes
- reviewing and arranging new office accommodation

Occupations:
511111 Contract Administrator
511112 Program or Project Administrator

511111 CONTRACT ADMINISTRATOR

Alternative Title:
Contract Officer

Prepares, interprets, maintains, reviews and negotiates variations to contracts on behalf of an organisation.

Skill Level: 2

511112 PROGRAM OR PROJECT ADMINISTRATOR

Alternative Title:
Project Coordinator

Plans and undertakes administration of organisational programs, special projects and support services.

Skill Level: 2

MINOR GROUP 512 OFFICE AND PRACTICE MANAGERS

OFFICE AND PRACTICE MANAGERS organise and manage the functions and resources of offices and professional practices such as administrative systems and office personnel.

Indicative Skill Level:
Most occupations in this minor group have a level of skill commensurate with the qualifications and experience outlined below.

In Australia:
AQF Associate Degree, Advanced Diploma or Diploma (ANZSCO Skill Level 2)

In New Zealand:
NZ Register Diploma (ANZSCO Skill Level 2)

At least three years of relevant experience may substitute for the formal qualifications listed above. In some instances relevant experience and/or on-the-job training may be required in addition to the formal qualification.

Tasks Include:

- contributing to the planning and review of office services, and setting priorities and office service standards
- allocating human resources, space and equipment
- assigning work to and monitoring work performance of staff
- managing records and accounts of the office
- liaising with Professionals to coordinate office business and to facilitate resolution of problems
- managing physical facilities and ensuring buildings and equipment are maintained
- ensuring compliance with occupational health and safety regulations
- ensuring work complies with relevant government legislation, policies and procedures
- coordinating personnel activities such as hiring, promotions, performance appraisals, payroll, training and supervision

Occupations in this minor group are classified into the following unit groups:

5121 Office Managers
5122 Practice Managers

UNIT GROUP 5121 OFFICE MANAGERS

OFFICE MANAGERS organise and control the functions and resources of offices such as administrative systems and office personnel.

Indicative Skill Level:
Most occupations in this unit group have a level of skill commensurate with the qualifications and experience outlined below.

In Australia:
AQF Associate Degree, Advanced Diploma or Diploma (ANZSCO Skill Level 2)

In New Zealand:
NZ Register Diploma (ANZSCO Skill Level 2)

At least three years of relevant experience may substitute for the formal qualifications listed above. In some instances relevant experience and/or on-the-job training may be required in addition to the formal qualification.

Tasks Include:
- contributing to the planning and review of office services, and setting priorities and office service standards
- allocating human resources, space and equipment
- assigning work to and monitoring work performance of staff
- managing records and accounts of the office
- liaising with Professionals to coordinate office business and to facilitate resolution of problems
- managing physical facilities and ensuring buildings and equipment are maintained
- ensuring compliance with occupational health and safety regulations
- ensuring work complies with relevant government legislation, policies and procedures
- coordinating personnel activities such as hiring, promotions, performance management, payroll, training and supervision

Occupation:
512111 Office Manager

512111 OFFICE MANAGER

Organises and controls the functions and resources of an office such as administrative systems and office personnel.

Skill Level: 2

UNIT GROUP 5122 PRACTICE MANAGERS

PRACTICE MANAGERS organise and control the functions and resources of professional practices such as administrative systems and practice personnel.

Indicative Skill Level:
Most occupations in this unit group have a level of skill commensurate with the qualifications and experience outlined below.

In Australia:
AQF Associate Degree, Advance Diploma or Diploma (ANZSCO Skill Level 2)

In New Zealand:
NZ Register Diploma (ANZSCO Skill Level 2)

At least three years of relevant experience may substitute for the formal qualifications listed above. In some instances relevant experience and/or on-the-job training may be required in addition to the formal qualification.

Tasks Include:
- contributing to the planning and review of office services, and setting priorities and office service standards
- allocating human resources, space and equipment
- assigning work to and monitoring work performance of staff
- managing records and accounts of the practice
- liaising with Professionals to coordinate practice business and to facilitate resolution of problems
- managing physical facilities and ensuring buildings and equipment are maintained
- ensuring compliance with occupational health and safety regulations
- ensuring work complies with relevant government legislation, policies and procedures
- coordinating personnel activities such as hiring, promotions, performance management, payroll, training and supervision

Occupations:
512211 Health Practice Manager
512299 Practice Managers nec

512211 HEALTH PRACTICE MANAGER

Organises and controls the functions and resources of a health practice such as administrative systems and practice personnel.

Skill Level: 2

Specialisations:
Chiropractic Practice Manager
Dental Practice Manager
Medical Practice Manager
Physiotherapy Practice Manager

UNIT GROUP 5122 PRACTICE MANAGERS *continued*

512299 PRACTICE MANAGERS NEC

This occupation group covers Practice Managers not elsewhere classified.

Skill Level: 2

Occupations in this group include:

- Accounting Practice Manager
- Architectural Practice Manager
- Legal Practice Manager
- Veterinary Practice Manager

SUB-MAJOR GROUP 52 PERSONAL ASSISTANTS AND SECRETARIES

PERSONAL ASSISTANTS AND SECRETARIES perform organisational, clerical, secretarial and other administrative tasks in support of Managers and Professionals.

Indicative Skill Level:
Most occupations in this sub-major group have a level of skill commensurate with the qualifications and experience outlined below.

In Australia:
AQF Certificate III including at least two years of on-the-job training, or AQF Certificate IV (ANZSCO Skill Level 3)

In New Zealand:
NZ Register Level 4 qualification (ANZSCO Skill Level 3)

At least three years of relevant experience may substitute for the formal qualifications listed above. In some instances relevant experience and/or on-the-job training may be required in addition to the formal qualification.

Tasks Include:
- liaising with other staff about a range of matters relating to the organisation's operations
- drafting and preparing documents such as briefing notes, memoranda and correspondence
- maintaining appointment diaries and making travel arrangements
- processing mail, filing correspondence and maintaining records
- answering telephone calls and inquiries
- taking and transcribing dictation of letters and other documents

Occupations in this sub-major group are classified into the following minor group:
521 Personal Assistants and Secretaries

MINOR GROUP 521 PERSONAL ASSISTANTS AND SECRETARIES

PERSONAL ASSISTANTS AND SECRETARIES perform organisational, clerical, secretarial and other administrative tasks in support of Managers and Professionals.

Indicative Skill Level:
Most occupations in this minor group have a level of skill commensurate with the qualifications and experience outlined below.

In Australia:
AQF Certificate III including at least two years of on-the-job training, or AQF Certificate IV (ANZSCO Skill Level 3)

In New Zealand:
NZ Register Level 4 qualification (ANZSCO Skill Level 3)

At least three years of relevant experience may substitute for the formal qualifications listed above. In some instances relevant experience and/or on-the-job training may be required in addition to the formal qualification.

Tasks Include:
- liaising with other staff about a range of matters relating to the organisation's operations
- drafting and preparing documents such as briefing notes, memoranda and correspondence
- maintaining appointment diaries and making travel arrangements
- processing mail, filing correspondence and maintaining records
- answering telephone calls and inquiries
- taking and transcribing dictation of letters and other documents

Occupations in this minor group are classified into the following unit groups:
5211 Personal Assistants
5212 Secretaries

UNIT GROUP 5211 PERSONAL ASSISTANTS

PERSONAL ASSISTANTS perform liaison, coordination and organisational tasks in support of Managers and Professionals.

Indicative Skill Level:
Most occupations in this unit group have a level of skill commensurate with the qualifications and experience outlined below.

In Australia:
AQF Certificate III including at least two years of on-the-job training, or AQF Certificate IV (ANZSCO Skill Level 3)

In New Zealand:
NZ Register Level 4 qualification (ANZSCO Skill Level 3)

At least three years of relevant experience may substitute for the formal qualifications listed above. In some instances relevant experience and/or on-the-job training may be required in addition to the formal qualification.

Tasks Include:
- liaising with other staff on matters relating to the organisation's operations
- researching and preparing reports, briefing notes, memoranda, correspondence and other routine documents
- maintaining confidential files and documents
- attending meetings and acting as secretary as required
- maintaining appointment diaries and making travel arrangements
- processing incoming and outgoing mail, filing correspondence and maintaining records
- screening telephone calls and answering inquiries
- taking and transcribing dictation of letters and other documents
- may supervise other secretarial and clerical staff

Occupation:
521111 Personal Assistant

521111 PERSONAL ASSISTANT

Performs liaison, coordination and organisational tasks in support of Managers and Professionals.

Skill Level: 3

UNIT GROUP 5212 SECRETARIES

SECRETARIES perform secretarial, clerical and other administrative tasks in support of Managers, Legal Professionals and other professionals.

Medical Secretaries are excluded from this unit group. Medical Secretaries are included in Unit Group 5421 Receptionists, in Occupation 542114 Medical Receptionist.

Indicative Skill Level:
Most occupations in this unit group have a level of skill commensurate with the qualifications and experience outlined below.

In Australia:
AQF Certificate III including at least two years of on-the-job training, or AQF Certificate IV (ANZSCO Skill Level 3)

In New Zealand:
NZ Register Level 4 qualification (ANZSCO Skill Level 3)

At least three years of relevant experience may substitute for the formal qualifications listed above. In some instances relevant experience and/or on-the-job training may be required in addition to the formal qualification.

Tasks Include:
- liaising with other staff to arrange meetings, and to gain and provide information
- preparing reports, briefing notes and correspondence, and proofreading work for typographical and grammatical errors
- maintaining appointment diaries and making travel arrangements
- processing incoming and outgoing mail, filing correspondence and maintaining records
- answering telephone calls, responding to inquiries and redirecting callers
- taking and transcribing dictation of letters and other documents
- greeting visitors, ascertaining nature of business and directing visitors to appropriate persons
- may implement management decisions and maintain records of meetings
- may handle bookkeeping and petty cash functions

Occupation:
521211 Secretary (General)
521212 Legal Secretary

521211 SECRETARY (GENERAL)

Performs secretarial, clerical and other administrative tasks in support of Managers and Professionals.

Skill Level: 3

521212 LEGAL SECRETARY

Performs secretarial, clerical and other administrative tasks in support of Legal Professionals applying knowledge of legal terminology, procedures and documents.

Skill Level: 3

SUB-MAJOR GROUP 53 GENERAL CLERICAL WORKERS

GENERAL CLERICAL WORKERS perform general administrative, data entry and word processing tasks.

Indicative Skill Level:
Most occupations in this sub-major group have a level of skill commensurate with the qualifications and experience outlined below.

In Australia:
AQF Certificate II or III (ANZSCO Skill Level 4)

In New Zealand:
NZ Register Level 2 or 3 qualification (ANZSCO Skill Level 4)

At least one year of relevant experience may substitute for the formal qualifications listed above. In some instances relevant experience and/or on-the-job training may be required in addition to the formal qualification.

Tasks Include:
- receiving, sorting, opening, classifying, photocopying and filing information
- entering text and data via keyboards for further processing
- retrieving and updating data in storage and keeping records
- preparing reports, letters and similar matter
- transcribing information, and proofreading and correcting copy

Occupations in this sub-major group are classified into the following minor groups:
531 General Clerks
532 Keyboard Operators

MINOR GROUP 531 GENERAL CLERKS

GENERAL CLERKS perform a range of clerical and administrative tasks.

Indicative Skill Level:
Most occupations in this minor group have a level of skill commensurate with the qualifications and experience outlined below.

In Australia:
AQF Certificate II or III (ANZSCO Skill Level 4)

In New Zealand:
NZ Register Level 2 or 3 qualification (ANZSCO Skill Level 4)

At least one year of relevant experience may substitute for the formal qualifications listed above. In some instances relevant experience and/or on-the-job training may be required in addition to the formal qualification.

Tasks Include:
- recording, preparing, sorting, classifying and filing information
- sorting, opening and sending mail
- photocopying and faxing documents
- preparing reports of a routine nature
- recording issue of equipment to staff
- receiving letters and telephone messages
- transcribing information onto computers, and proofreading and correcting copy
- may provide customers with information about services
- may perform receptionist duties

Occupations in this minor group are classified into the following unit group:
5311 General Clerks

UNIT GROUP 5311 GENERAL CLERKS

GENERAL CLERKS perform a range of clerical and administrative tasks.

Indicative Skill Level:
Most occupations in this unit group have a level of skill commensurate with the qualifications and experience outlined below.

In Australia:
AQF Certificate II or III (ANZSCO Skill Level 4)

In New Zealand:
NZ Register Level 2 or 3 qualification (ANZSCO Skill Level 4)

At least one year of relevant experience may substitute for the formal qualifications listed above. In some instances relevant experience and/or on-the-job training may be required in addition to the formal qualification.

Tasks Include:
- recording, preparing, sorting, classifying and filing information
- sorting, opening and sending mail
- photocopying and faxing documents
- preparing reports of a routine nature
- recording issue of equipment to staff
- receiving letters and telephone messages
- transcribing information onto computers, and proofreading and correcting copy
- may provide customers with information about services
- may perform receptionist duties

Occupation:
531111 General Clerk

531111 GENERAL CLERK

Performs a range of clerical and administrative tasks.

Skill Level: 4

MINOR GROUP 532 KEYBOARD OPERATORS

KEYBOARD OPERATORS input and process text and data, and prepare, edit and generate documents for storage, processing, publication and transmission.

Indicative Skill Level:
Most occupations in this minor group have a level of skill commensurate with the qualifications and experience outlined below.

In Australia:
AQF Certificate II or III (ANZSCO Skill Level 4)

In New Zealand:
NZ Register Level 2 or 3 qualification (ANZSCO Skill Level 4)

At least one year of relevant experience may substitute for the formal qualifications listed above. In some instances relevant experience and/or on-the-job training may be required in addition to the formal qualification.

Tasks Include:

- entering data and codes required to process information
- retrieving, confirming and updating data in storage and keeping records of data input
- taking verbatim records of proceedings in rapid shorthand using computerised equipment and shorthand-writing machines
- transcribing information recorded in shorthand and on sound recording equipment, and proofreading and correcting copy
- reading portions of transcripts during trials and other proceedings on request of Judges and other officials
- reproducing the spoken word, environmental sounds and song lyrics as captions for television programming, and the deaf and hearing impaired
- preparing reports, letters and similar material for publication and electronic transmission
- sorting outgoing material and preparing documents for transmission

Occupations in this minor group are classified into the following unit group:
5321 Keyboard Operators

UNIT GROUP 5321 KEYBOARD OPERATORS

KEYBOARD OPERATORS input and process text and data, and prepare, edit and generate documents for storage, processing, publication and transmission.

Indicative Skill Level:
Most occupations in this unit group have a level of skill commensurate with the qualifications and experience outlined below.

In Australia:
AQF Certificate II or III (ANZSCO Skill Level 4)

In New Zealand:
NZ Register Level 2 or 3 qualification (ANZSCO Skill Level 4)

At least one year of relevant experience may substitute for the formal qualifications listed above. In some instances relevant experience and/or on-the-job training may be required in addition to the formal qualification.

Tasks Include:
- entering data and codes required to process information
- retrieving, confirming and updating data in storage and keeping records of data input
- taking verbatim records of proceedings in rapid shorthand using computerised equipment and shorthand-writing machines
- transcribing information recorded in shorthand and on sound recording equipment, and proofreading and correcting copy
- reading portions of transcripts during trials and other proceedings on request of Judges and other officials
- reproducing the spoken word, environmental sounds and song lyrics as captions for television programming, and the deaf and hearing impaired
- preparing reports, letters and similar material for publication and electronic transmission
- sorting outgoing material and preparing documents for transmission

Occupations:
532111 Data Entry Operator
532112 Machine Shorthand Reporter
532113 Word Processing Operator

532111 DATA ENTRY OPERATOR

Alternative Title:
Data Processing Operator

Operates a keyboard to input and transfer data into a computer for storage, processing and transmission.

Skill Level: 4

UNIT GROUP 5321 KEYBOARD OPERATORS *continued*

532112 MACHINE SHORTHAND REPORTER

Records and reproduces the spoken word in court and parliamentary proceedings, television programming and for the deaf and hearing impaired using handwritten shorthand, stenotype shorthand machines, computer-assisted transcription software and sound recording equipment.

Skill Level: 4

Specialisations:

- Braille Transcriber
- Court Reporter
- Hansard Reporter
- Realtime Reporter
- Stenocaptioner

532113 WORD PROCESSING OPERATOR

Alternative Title:

- Typist

Operates a computer to type, edit and generate a variety of documents and reports.

Skill Level: 4

SUB-MAJOR GROUP 54 INQUIRY CLERKS AND RECEPTIONISTS

INQUIRY CLERKS AND RECEPTIONISTS respond to requests for information, and receive and greet people.

Indicative Skill Level:
Most occupations in this sub-major group have a level of skill commensurate with the qualifications and experience outlined below.

In Australia:
AQF Certificate III including at least two years of on-the-job training, or AQF Certificate IV, or at least three years of relevant experience (ANZSCO Skill Level 3); or
AQF Certificate II or III, or at least one year of relevant experience (ANZSCO Skill Level 4)

In New Zealand:
NZ Register Level 4 qualification, or at least three years of relevant experience (ANZSCO Skill Level 3); or
NZ Register Level 2 or 3 qualification, or at least one year of relevant experience (ANZSCO Skill Level 4)

In some instances relevant experience and/or on-the-job training may be required in addition to the formal qualification.

Tasks Include:
- answering customer inquiries for information about the organisation and the goods and services it offers
- resolving customer complaints and problems with goods and services provided
- recording information about inquiries and complaints
- greeting and welcoming visitors, and directing them to the appropriate person
- arranging and recording details of appointments
- answering, connecting and transferring telephone calls

Occupations in this sub-major group are classified into the following minor groups:
541 Call or Contact Centre Information Clerks
542 Receptionists

MINOR GROUP 541 CALL OR CONTACT CENTRE INFORMATION CLERKS

CALL OR CONTACT CENTRE INFORMATION CLERKS provide information to customers about goods and services.

Indicative Skill Level:
Most occupations in this minor group have a level of skill commensurate with the qualifications and experience outlined below.

In Australia:

AQF Certificate III including at least two years of on-the-job training, or AQF Certificate IV, or at least three years of relevant experience (ANZSCO Skill Level 3); or

AQF Certificate II or III, or at least one year of relevant experience (ANZSCO Skill Level 4)

In New Zealand:

NZ Register Level 4 qualification, or at least three years of relevant experience (ANZSCO Skill Level 3); or

NZ Register Level 2 or 3 qualification, or at least one year of relevant experience (ANZSCO Skill Level 4)

In some instances relevant experience and/or on-the-job training may be required in addition to the formal qualification.

Tasks Include:

- answering customer inquiries for information about goods and services
- resolving customer complaints and problems with goods and services provided
- recording information about inquiries and complaints
- referring complex inquiries to supervisors
- arranging the despatch of information kits and brochures

Occupations in this minor group are classified into the following unit groups:

5411 Call or Contact Centre Workers

5412 Inquiry Clerks

UNIT GROUP 5411 CALL OR CONTACT CENTRE WORKERS

CALL OR CONTACT CENTRE WORKERS respond to telephone, Internet and email inquiries and complaints about an organisation's goods and services, and promote the goods and services.

Indicative Skill Level:
The occupation Call or Contact Centre Team Leader has a level of skill commensurate with the qualifications and experience outlined below.

In Australia:
AQF Certificate III including at least two years of on-the-job training, or AQF Certificate IV (ANZSCO Skill Level 3)

In New Zealand:
NZ Register Level 4 qualification (ANZSCO Skill Level 3)

At least three years of relevant experience may substitute for the formal qualifications listed above. In some instances relevant experience and/or on-the-job training may be required in addition to the formal qualification.

The occupation Call or Contact Centre Operator has a level of skill commensurate with the qualifications and experience outlined below.

In Australia:
AQF Certificate II or III (ANZSCO Skill Level 4)

In New Zealand:
NZ Register Level 2 or 3 qualification (ANZSCO Skill Level 4)

At least one year of relevant experience may substitute for the formal qualifications listed above. In some instances relevant experience and/or on-the-job training may be required in addition to the formal qualification.

Tasks Include:
- answering incoming calls, emails and messages, and assisting customers with their specific inquiries
- identifying requirements and recording information into computer systems
- coaching staff and assisting call centre operators to resolve problems and customer inquiries
- developing rosters and managing staff numbers to meet work flows
- listening to calls conducted by call centre operators and providing performance feedback
- monitoring and timing calls
- creating further interest in goods and services by offering customers more information about goods and inviting customers to use services on offer
- updating databases to reflect changes to the status of customers and prospective customers
- arranging the despatch of goods, information kits and brochures to customers and interested parties
- undertaking clerical duties, such as faxing, and filling out paperwork, and liaising with other departments associated with completing the customer contact
- issuing invoices and receiving electronic payments for goods and services provided

Occupations:
541111 Call or Contact Centre Team Leader
541112 Call or Contact Centre Operator

UNIT GROUP 5411 CALL OR CONTACT CENTRE WORKERS *continued*

541111 CALL OR CONTACT CENTRE TEAM LEADER

Alternative Titles:
- Call Centre Supervisor
- Contact Centre Supervisor

Oversees and determines work requirements, monitors telephone calls, coaches and allocates duties to Call or Contact Centre Operators.

Skill Level: 3

Specialisations:
- Call or Contact Centre Coach
- Call or Contact Centre Workforce Planner

541112 CALL OR CONTACT CENTRE OPERATOR

Answers customer telephone, Internet and email inquiries about goods and services, and promotes the goods and services.

Skill Level: 4

UNIT GROUP 5412 INQUIRY CLERKS

INQUIRY CLERKS respond to personal, written and telephone inquiries and complaints about the organisation's goods and services, provide information and refer people to other sources.

Indicative Skill Level:
Most occupations in this unit group have a level of skill commensurate with the qualifications and experience outlined below.

In Australia:
AQF Certificate II or III (ANZSCO Skill Level 4)

In New Zealand:
NZ Register Level 2 or 3 qualification (ANZSCO Skill Level 4)

At least one year of relevant experience may substitute for the formal qualifications listed above. In some instances relevant experience and/or on-the-job training may be required in addition to the formal qualification.

Tasks Include:
- answering inquiries about goods and services, and providing information about their availability, location, price and related issues
- responding to inquiries about problems and providing advice, information and assistance
- recording information about inquiries and complaints
- referring complex inquiries to team leaders or expert advisers
- issuing relevant forms, information kits and brochures to interested parties
- accessing and operating computer network systems and communication systems such as public address and paging systems
- may refer inquiries to other sources

Occupation:
541211 Inquiry Clerk

541211 INQUIRY CLERK

Alternative Title:
Inquiry Officer (Aus)

Responds to personal, written and telephone inquiries and complaints about the organisation's goods and services, provides information and refers people to other sources.

Skill Level: 4

Specialisation:
Information Clerk

MINOR GROUP 542 RECEPTIONISTS

RECEPTIONISTS receive and welcome visitors, patients, guests and clients, and respond to inquiries and requests.

Medical Secretaries are included in this minor group, in Occupation 542114 Medical Receptionist.

Indicative Skill Level:
Most occupations in this minor group have a level of skill commensurate with the qualifications and experience outlined below.

In Australia:
AQF Certificate II or III (ANZSCO Skill Level 4)

In New Zealand:
NZ Register Level 2 or 3 qualification (ANZSCO Skill Level 4)

At least one year of relevant experience may substitute for the formal qualifications listed above. In some instances relevant experience and/or on-the-job training may be required in addition to the formal qualification.

Tasks Include:

- greeting and welcoming visitors, and directing them to the appropriate person
- arranging and recording details of appointments
- answering inquiries and providing information on the goods, services and activities of the organisation
- answering, connecting and transferring telephone calls
- receiving and resolving complaints from clients and the public
- receiving and distributing correspondence, facsimile messages and deliveries
- maintaining the reception area
- advising on and arranging reservations and accommodation
- may perform other clerical tasks such as word processing, data entry, filing, mail despatch and photocopying

Occupations in this minor group are classified into the following unit group:
5421 Receptionists

UNIT GROUP 5421 RECEPTIONISTS

RECEPTIONISTS receive and welcome visitors, patients, guests and clients, and respond to inquiries and requests.

Medical Secretaries are included in this unit group, in Occupation 542114 Medical Receptionist.

Indicative Skill Level:
Most occupations in this unit group have a level of skill commensurate with the qualifications and experience outlined below.

In Australia:
AQF Certificate II or III (ANZSCO Skill Level 4)

In New Zealand:
NZ Register Level 2 or 3 qualification (ANZSCO Skill Level 4)

At least one year of relevant experience may substitute for the formal qualifications listed above. In some instances relevant experience and/or on-the-job training may be required in addition to the formal qualification.

Tasks Include:
- greeting and welcoming visitors, and directing them to the appropriate person
- arranging and recording details of appointments
- answering inquiries and providing information on the goods, services and activities of the organisation
- answering, connecting and transferring telephone calls
- receiving and resolving complaints from clients and the public
- receiving and distributing correspondence, facsimile messages and deliveries
- maintaining the reception area
- advising on and arranging reservations and accommodation
- may perform other clerical tasks such as word processing, data entry, filing, mail despatch and photocopying

Occupations:
542111 Receptionist (General)
542112 Admissions Clerk
542113 Hotel or Motel Receptionist
542114 Medical Receptionist

542111 RECEPTIONIST (GENERAL)

Greets clients and visitors, and responds to personal, telephone, email and written inquiries and requests.

Skill Level: 4

542112 ADMISSIONS CLERK

Alternative Title:
Hospital Ward Clerk

Records and processes information required for the admission and discharge of hospital patients and responds to telephone inquiries.

Skill Level: 4

UNIT GROUP 5421 RECEPTIONISTS *continued*

542113 HOTEL OR MOTEL RECEPTIONIST

Greets and checks in guests, and looks after their needs on arrival and during their stay in a hotel or motel.

Skill Level: 4

542114 MEDICAL RECEPTIONIST

Greets patients and other clients in a health facility, such as a clinic, practice, centre or surgery, and responds to personal, telephone and written inquiries and requests.

Skill Level: 4

SUB-MAJOR GROUP 55 NUMERICAL CLERKS

NUMERICAL CLERKS compile, record and process documents relating to creditors and debtors, operating costs, financial transactions and payrolls, provide financial services to bank customers, and undertake routine statistical and actuarial computations.

Indicative Skill Level:
Most occupations in this sub-major group have a level of skill commensurate with the qualifications and experience outlined below.

In Australia:
AQF Certificate II or III (ANZSCO Skill Level 4)

In New Zealand:
NZ Register Level 2 or 3 qualification (ANZSCO Skill Level 4)

At least one year of relevant experience may substitute for the formal qualifications listed above. In some instances relevant experience and/or on-the-job training may be required in addition to the formal qualification.

Tasks Include:
- processing accounts payable and receivable, and payment of wages and salaries
- keeping financial records, and maintaining, reconciling and balancing accounts
- processing and authorising credit and loan applications, and processing insurance applications and claims
- accepting money deposited by customers and crediting customers' accounts
- maintaining records of securities registrations and transactions
- compiling financial and statistical data, tables, graphs and charts

Occupations in this sub-major group are classified into the following minor groups:
551 Accounting Clerks and Bookkeepers
552 Financial and Insurance Clerks

MINOR GROUP 551 ACCOUNTING CLERKS AND BOOKKEEPERS

ACCOUNTING CLERKS AND BOOKKEEPERS compile, record and process documents relating to creditors and debtors, operating costs, financial transactions and payrolls.

Indicative Skill Level:
Most occupations in this minor group have a level of skill commensurate with the qualifications and experience outlined below.

In Australia:
AQF Certificate II or III (ANZSCO Skill Level 4)

In New Zealand:
NZ Register Level 2 or 3 qualification (ANZSCO Skill Level 4)

At least one year of relevant experience may substitute for the formal qualifications listed above. In some instances relevant experience and/or on-the-job training may be required in addition to the formal qualification.

Tasks Include:
- preparing and processing documentation related to accounts payable and receivable, and wages and salaries
- reconciling invoices and despatching payments
- investigating, compiling and preparing reports of operating cost data
- keeping financial records
- maintaining, reconciling and balancing accounts
- processing payments of accounts, and wages and salaries

Occupations in this minor group are classified into the following unit groups:
5511 Accounting Clerks
5512 Bookkeepers
5513 Payroll Clerks

UNIT GROUP 5511 ACCOUNTING CLERKS

ACCOUNTING CLERKS monitor creditor and debtor accounts, undertake related routine documentation, and calculate and investigate the cost of wages, materials, overheads and other operating costs.

Indicative Skill Level:
Most occupations in this unit group have a level of skill commensurate with the qualifications and experience outlined below.

In Australia:
AQF Certificate II or III (ANZSCO Skill Level 4)

In New Zealand:
NZ Register Level 2 or 3 qualification (ANZSCO Skill Level 4)

At least one year of relevant experience may substitute for the formal qualifications listed above. In some instances relevant experience and/or on-the-job training may be required in addition to the formal qualification.

Tasks Include:

- preparing and processing documentation related to accounts payable and receivable
- reconciling invoices and despatching payments
- calculating, analysing and investigating the costs of proposed expenditure, wages and standard costs
- preparing bank reconciliations
- allocating expenditure to specified budget accounts
- summarising expenditure and receipts
- preparing records of standard costs and values for items such as raw materials and packaging supplies
- recording cost variations and contract price movements
- compiling cost data for preparation of operating budgets, and profit and loss calculations
- investigating the costs of proposed expenditures, quotations and estimates
- preparing reports of total costs, inventory adjustments, selling prices and profits
- may work in a call centre

Occupations:
551111 Accounts Clerk
551112 Cost Clerk

551111 ACCOUNTS CLERK

Alternative Title:
Accounts Payable or Receivable Clerk

Monitors creditor and debtor accounts, and undertakes related routine documentation. May work in a call centre.

Skill Level: 4

Specialisations:
Audit Clerk
Investment Accounting Clerk

551112 COST CLERK

Calculates and investigates the cost of wages, materials, overheads and other operating expenses.

Skill Level: 4

UNIT GROUP 5512 BOOKKEEPERS

BOOKKEEPERS maintain and evaluate records of financial transactions in account books and computerised accounting systems.

Indicative Skill Level:
Most occupations in this unit group have a level of skill commensurate with the qualifications and experience outlined below.

In Australia:
AQF Certificate II or III (ANZSCO Skill Level 4)

In New Zealand:
NZ Register Level 2 or 3 qualification (ANZSCO Skill Level 4)

At least one year of relevant experience may substitute for the formal qualifications listed above. In some instances relevant experience and/or on-the-job training may be required in addition to the formal qualification.

Tasks Include:
- keeping financial records, and maintaining and balancing accounts using manual and computerised systems
- monitoring cash flow and lines of credit
- preparing and producing financial statements, budget and expenditure reports and analyses using account books, ledgers and accounting software packages
- preparing invoices, purchase orders and bank deposits
- reconciling accounts against monthly bank statements
- verifying recorded transactions and reporting irregularities to management
- may be required to prepare forms reporting business tax entitlements and obligations such as the amount of goods and services tax paid and collected

Occupation:
551211 Bookkeeper

551211 BOOKKEEPER

Maintains and evaluates records of financial transactions in account books and computerised accounting systems.

Skill Level: 4

Specialisation:
Financial Administration Officer

UNIT GROUP 5513 PAYROLL CLERKS

PAYROLL CLERKS prepare payrolls and related records for employee salaries and statutory record-keeping purposes.

Indicative Skill Level:
Most occupations in this unit group have a level of skill commensurate with the qualifications and experience outlined below.

In Australia:
AQF Certificate II or III (ANZSCO Skill Level 4)

In New Zealand:
NZ Register Level 2 or 3 qualification (ANZSCO Skill Level 4)

At least one year of relevant experience may substitute for the formal qualifications listed above. In some instances relevant experience and/or on-the-job training may be required in addition to the formal qualification.

Tasks Include:
- creating files for new employees to record payroll data
- maintaining and updating files for existing employees to record information such as employee contact details, leave taken, overtime, promotions, transfers, tax deductions, health insurance payments and superannuation
- preparing payroll data from time sheets and other payroll and personnel records
- processing payment of wages and salaries
- issuing and recording adjustments to employees' pay
- interpreting industrial awards
- providing information to employees and managers about payroll matters such as tax issues, benefits and deductions
- finalising files and arrangements when employees retire, resign or transfer
- may be in involved in maintaining superannuation and other deduction and contribution records

Occupation:
551311 Payroll Clerk

551311 PAYROLL CLERK

Alternative Titles:
Pay Clerk
Payroll Officer

Prepares payroll and related records for employee salaries and statutory record-keeping purposes.

Skill Level: 4

MINOR GROUP 552 FINANCIAL AND INSURANCE CLERKS

FINANCIAL AND INSURANCE CLERKS receive deposits and pay out money in financial institutions, process credit, loan and insurance applications, maintain records of securities transactions and registrations, offer odds and accept bets, and compile data and undertake statistical and actuarial computations.

Indicative Skill Level:
Most occupations in this minor group have a level of skill commensurate with the qualifications and experience outlined below.

In Australia:
AQF Certificate II or III (ANZSCO Skill Level 4)

In New Zealand:
NZ Register Level 2 or 3 qualification (ANZSCO Skill Level 4)

At least one year of relevant experience may substitute for the formal qualifications listed above. In some instances relevant experience and/or on-the-job training may be required in addition to the formal qualification.

Tasks Include:
- answering customer inquiries about bank accounts, credit standing and loans
- receiving deposits of money from and paying withdrawals of money to customers, and crediting and debiting their accounts
- processing and authorising the approval of credit and loan applications
- offering and varying odds on sporting events, and accepting and paying out bets
- processing insurance applications, adjustments to cover and claims against policies
- maintaining records of securities registrations and transactions
- compiling tables, graphs and charts

Occupations in this minor group are classified into the following unit groups:
5521 Bank Workers
5522 Credit and Loans Officers
5523 Insurance, Money Market and Statistical Clerks

UNIT GROUP 5521 BANK WORKERS

BANK WORKERS receive deposits and pay out money in financial and commercial institutions, keep records of transactions, issue receipts and cash cheques.

Indicative Skill Level:
Most occupations in this unit group have a level of skill commensurate with the qualifications and experience outlined below.

In Australia:
AQF Certificate II or III (ANZSCO Skill Level 4)

In New Zealand:
NZ Register Level 2 or 3 qualification (ANZSCO Skill Level 4)

At least one year of relevant experience may substitute for the formal qualifications listed above. In some instances relevant experience and/or on-the-job training may be required in addition to the formal qualification.

Tasks Include:
- greeting customers, identifying their needs and answering customer inquiries
- ensuring customers' forms are filled in correctly and checking customers' identification
- accepting cash and cheques deposited by customers, verifying records and receipts, and crediting customers' accounts
- paying money to customers according to advice slips, cheques and negotiable documents, and debiting customers' accounts
- providing change, cashing cheques and recording transactions
- opening and closing accounts for customers
- balancing cash and advising supervisors of cash position and discrepancies
- explaining and promoting bank services to customers and referring them to appropriate financial services

Occupation:
552111 Bank Worker

552111 BANK WORKER

Receives deposits and pays out money in a financial or commercial institution, keeps records of transactions, issues receipts and cashes cheques.

Skill Level: 4

UNIT GROUP 5522 CREDIT AND LOANS OFFICERS

CREDIT AND LOANS OFFICERS analyse, evaluate and process credit and loan applications.

Indicative Skill Level:
Most occupations in this unit group have a level of skill commensurate with the qualifications and experience outlined below.

In Australia:
AQF Certificate II or III (ANZSCO Skill Level 4)

In New Zealand:
NZ Register Level 2 or 3 qualification (ANZSCO Skill Level 4)

At least one year of relevant experience may substitute for the formal qualifications listed above. In some instances relevant experience and/or on-the-job training may be required in addition to the formal qualification.

Tasks Include:
- analysing information about customers and examining references, credit ratings, investment risks, pay slips and other information against predetermined policy standards
- contacting financial and credit institutions to obtain information about customers
- preparing papers setting out conditions of credit and loans, rates of repayment and loan periods, and providing information about customers' standing to financial and credit institutions
- authorising the approval of credit and loan applications and recommending credit and loan conditions and limits
- keeping records of payments, and preparing routine letters requesting payment for overdue accounts and forwarding these for legal action
- answering inquiries concerning credit standing of customers, loan balances and penalties
- may recommend, approve and arrange mortgages
- may work in a call centre

Occupation:
552211 Credit or Loans Officer

552211 CREDIT OR LOANS OFFICER

Alternative Titles:
Credit Clerk
Finance Clerk
Lending Consultant
Loans Consultant
Loans Officer

Analyses, evaluates and processes credit and loan applications. May work in a call centre.

Skill Level: 4

UNIT GROUP 5523 INSURANCE, MONEY MARKET AND STATISTICAL CLERKS

INSURANCE, MONEY MARKET AND STATISTICAL CLERKS prepare and check documentation associated with insurance, maintain records of securities transactions and registrations, offer odds and accept bets, and compile data and undertake statistical and actuarial computations.

Indicative Skill Level:

Most occupations in this unit group have a level of skill commensurate with the qualifications and experience outlined below.

In Australia:

AQF Certificate II or III (ANZSCO Skill Level 4)

In New Zealand:

NZ Register level 2 or 3 qualification (ANZSCO Skill Level 4)

At least one year of relevant experience may substitute for the formal qualifications listed above. In some instances relevant experience and/or on-the-job training may be required in addition to the formal qualification.

Registration or licensing may be required.

Tasks Include:

- obtaining information on the form of competitors by research, attending race trials and liaising with contacts
- offering and varying odds on competitors after considering the type of event, handicaps, weather conditions and odds offered by other Bookmakers
- processing insurance applications, adjustments to insurance cover, standard endorsements and insurance claims
- monitoring balances of accounts and summarising reinsurance to determine outstanding risk
- surveying potential risk exposure
- despatching notices of premiums due and forms concerning conservation and transfer of insurance
- reviewing, checking, verifying and issuing transaction documentation for securities
- claiming accruing dividends and processing dividend payments
- compiling statistics from financial records, survey returns and other data sources, and verifying the authenticity of the material
- operating computers to input, manipulate and output information
- compiling results of calculations into tables, graphs and charts to be used in analysis
- may work in a call centre

Occupations:

552311 Bookmaker
552312 Insurance Consultant
552313 Money Market Clerk
552314 Statistical Clerk

552311 BOOKMAKER

Determines risk, offers odds and accepts bets on the outcome of racing and other events. Registration or licensing is required.

This occupation is illegal in New Zealand.

Skill Level: 4

UNIT GROUP 5523 INSURANCE, MONEY MARKET AND STATISTICAL CLERKS *continued*

552312 INSURANCE CONSULTANT

Alternative Title:

Insurance Clerk

Prepares and checks documentation associated with insurance. May work in a call centre.

Skill Level: 4

Specialisations:

Health Insurance Assessor

Superannuation Clerk

552313 MONEY MARKET CLERK

Alternative Titles:

Scrip Clerk (Stockbroking)

Securities Clerk

Processes documentation and maintains records of securities transactions and registrations.

Skill Level: 4

552314 STATISTICAL CLERK

Compiles data and undertakes statistical and actuarial computations.

Skill Level: 4

Specialisation:

Actuarial Clerk

SUB-MAJOR GROUP 56 CLERICAL AND OFFICE SUPPORT WORKERS

CLERICAL AND OFFICE SUPPORT WORKERS perform a range of routine clerical and administrative tasks necessary to support the operation of organisations.

Indicative Skill Level:
Most occupations in this sub-major group have a level of skill commensurate with the qualifications outlined below.

In Australia:
AQF Certificate I, or compulsory secondary education (ANZSCO Skill Level 5)

In New Zealand:
NZ Register Level 1 qualification, or compulsory secondary education (ANZSCO Skill Level 5)

For some occupations a short period of on-the-job training may be required in addition to or instead of the formal qualification. In some instances no formal qualification or on-the-job training may be required.

Tasks Include:
- recording and entering bets, debiting credit and bank accounts electronically, and receiving cash
- sorting documents, mail and parcels, and delivering items to customers
- recording and updating information in record management systems
- interviewing people in surveys and market research to obtain information and their attitudes
- connecting, holding and transferring telephone calls, and providing telephone service information
- receiving advertising copy and entering text and other details
- reading meters

Occupations in this sub-major group are classified into the following minor group:
561 Clerical and Office Support Workers

MINOR GROUP 561 CLERICAL AND OFFICE SUPPORT WORKERS

CLERICAL AND OFFICE SUPPORT WORKERS perform a range of routine clerical and administrative tasks necessary to support the operation of organisations.

Indicative Skill Level:
Most occupations in this minor group have a level of skill commensurate with the qualifications outlined below.

In Australia:
AQF Certificate I, or compulsory secondary education (ANZSCO Skill Level 5)

In New Zealand:
NZ Register Level 1 qualification, or compulsory secondary education (ANZSCO Skill Level 5)

For some occupations a short period of on-the-job training may be required in addition to or instead of the formal qualification. In some instances no formal qualification or on-the-job training may be required.

Tasks Include:
- recording and entering bets, debiting credit and bank accounts electronically, and receiving cash
- sorting documents, mail and parcels, and delivering items to customers
- recording and updating information in record management systems
- interviewing people in surveys and market research to obtain information and their attitudes
- connecting, holding and transferring telephone calls, and providing telephone service information
- receiving advertising copy and entering text and other details
- reading meters

Occupations in this minor group are classified into the following unit groups:
5611 Betting Clerks
5612 Courier and Postal Deliverers
5613 Filing and Registry Clerks
5614 Mail Sorters
5615 Survey Interviewers
5616 Switchboard Operators
5619 Other Clerical and Office Support Workers

UNIT GROUP 5611 BETTING CLERKS

BETTING CLERKS take bets from customers at betting agencies, over the telephone and on course.

Indicative Skill Level:
Most occupations in this unit group have a level of skill commensurate with the qualifications outlined below.

In Australia:
AQF Certificate I, or compulsory secondary education (ANZSCO Skill Level 5)

In New Zealand:
NZ Register Level 1 qualification, or compulsory secondary education (ANZSCO Skill Level 5)

For some occupations a short period of on-the-job training may be required in addition to or instead of the formal qualification. In some instances no formal qualification or on-the-job training may be required.

Registration or licensing may be required.

Tasks Include:
- taking bets and debiting credit accounts and bank accounts electronically, and receiving cash
- recording and entering bets electronically and in transaction ledgers
- issuing tickets and preparing summaries of transactions
- monitoring amounts of money placed on race entrants
- checking details and numbers on winning betting tickets against those in betting ledgers and electronic records, and paying out money on winning tickets
- verifying the identity and account balances of betting agency customers
- answering betting inquiries over the telephone, via email and in person
- may work in a call centre

Occupations:
561111 Betting Agency Counter Clerk
561112 Bookmaker's Clerk
561113 Telephone Betting Clerk
561199 Betting Clerks nec

561111 BETTING AGENCY COUNTER CLERK

Records and processes customer bets, payments and payouts over the counter at a betting agency for horse and dog racing, and other sports and events.

Skill Level: 5

561112 BOOKMAKER'S CLERK

Alternative Title:
Penciller

Assists Bookmakers to provide oncourse betting services at race meetings. Registration or licensing is required.

This occupation is illegal in New Zealand.

Skill Level: 5

Specialisation:
Bagman/woman (Aus)

UNIT GROUP 5611 BETTING CLERKS *continued*

561113 TELEPHONE BETTING CLERK

Records and processes customer bets and account details over the telephone for horse and dog racing, and other sports events. May work in a call centre.

Skill Level: 5

561199 BETTING CLERKS NEC

This occupation group covers Betting Clerks not elsewhere classified.

Skill Level: 5

Occupations in this group include:
- Bingo Caller
- Keno Terminal Operator

UNIT GROUP 5612 COURIERS AND POSTAL DELIVERERS

COURIERS AND POSTAL DELIVERERS deliver small items such as documents, messages, mail and parcels.

Indicative Skill Level:
Most occupations in this unit group have a level of skill commensurate with the qualifications outlined below.

In Australia:
AQF Certificate I, or compulsory secondary education (ANZSCO Skill Level 5)

In New Zealand:
NZ Register Level 1 qualification, or compulsory secondary education (ANZSCO Skill Level 5)

For some occupations a short period of on-the-job training may be required in addition to or instead of the formal qualification. In some instances no formal qualification or on-the-job training may be required.

Tasks Include:
- sorting and sequencing items for delivery
- delivering mail, parcels, documents and other items to customers' premises and mailboxes
- receiving orders for deliveries from customers
- collecting signatures and charges for cash-on-delivery orders
- issuing and collecting receipts for pick-up and delivery items
- keeping records of items received and delivered
- maintaining walk books, directories, mail counts, equipment maintenance logs and other delivery records
- loading and unloading mail conveyances and internal mail handling equipment
- assisting with receipting inward mail, checking wrongly addressed, missorted, undelivered and redirected mail, and processing freepost and underpaid mail

Occupations:
561211 Courier
561212 Postal Delivery Officer

561211 COURIER

Delivers goods, documents, messages, samples, x-rays and test results.

Skill Level: 5

Specialisations:
Bicycle Courier
Motorbike Courier
Parcel Contractor
Rural Mail Contractor

561212 POSTAL DELIVERY OFFICER

Alternative Title:
Postie

Delivers mail on foot, by bicycle or by motorised transport over allocated delivery rounds.

Skill Level: 5

UNIT GROUP 5613 FILING AND REGISTRY CLERKS

FILING AND REGISTRY CLERKS process and handle information and documents to maintain access to and security of database and record management systems.

Indicative Skill Level:
Most occupations in this unit group have a level of skill commensurate with the qualifications outlined below.

In Australia:
AQF Certificate I, or compulsory secondary education (ANZSCO Skill Level 5)

In New Zealand:
NZ Register Level 1 qualification, or compulsory secondary education (ANZSCO Skill Level 5)

For some occupations a short period of on-the-job training may be required in addition to or instead of the formal qualification. In some instances no formal qualification or on-the-job training may be required.

Tasks Include:
- sorting information and documents for filing according to database and record management system protocols
- classifying and coding information and documents for inclusion in database and record management systems
- updating and modifying records
- filing information and documents in database and record management systems
- identifying and retrieving information and documents for users
- recording file and document movements
- labelling storage locations, and assembling and labelling new files
- removing inactive and dead files

Occupation:
561311 Filing or Registry Clerk

561311 FILING OR REGISTRY CLERK

Alternative Title:
Records Clerk

Processes and handles information and documents to maintain access to and security of database and record management systems.

Skill Level: 5

UNIT GROUP 5614 MAIL SORTERS

MAIL SORTERS receive, sort and despatch mail in organisations and postal sorting centres.

Indicative Skill Level:
Most occupations in this unit group have a level of skill commensurate with the qualifications outlined below.

In Australia:
AQF Certificate I, or compulsory secondary education (ANZSCO Skill Level 5)

In New Zealand:
NZ Register Level 1 qualification, or compulsory secondary education (ANZSCO Skill Level 5)

For some occupations a short period of on-the-job training may be required in addition to or instead of the formal qualification. In some instances no formal qualification or on-the-job training may be required.

Tasks Include:
- receiving and checking incoming mail and mail bags
- assisting with the verification of registered and special articles
- operating mail processing equipment such as letter preparation lines, letter indexing and sorting equipment
- performing manual sorting duties and preparing documentation for despatching mail
- processing underpaid mail, bulk mail lodgements, express mail and other mail services
- operating letter indexing and sorting machines, multi-line optical character machines and bar-coding equipment
- investigating complaints regarding lost items

Occupations:
561411 Mail Clerk
561412 Postal Sorting Officer

561411 MAIL CLERK

Alternative Title:
Mail Officer

Collects, sorts and despatches mail within an organisation.

Skill Level: 5

561412 POSTAL SORTING OFFICER

Receives, sorts and despatches mail in a post office or postal sorting centre.

Skill Level: 5

Specialisation:
Parcel Post Officer

UNIT GROUP 5615 SURVEY INTERVIEWERS

SURVEY INTERVIEWERS interview people and record their responses to survey and market research questions on a range of topics.

Indicative Skill Level:
Most occupations in this unit group have a level of skill commensurate with the qualifications outlined below.

In Australia:
AQF Certificate I, or compulsory secondary education (ANZSCO Skill Level 5)

In New Zealand:
NZ Register Level 1 qualification, or compulsory secondary education (ANZSCO Skill Level 5)

For some occupations a short period of on-the-job training may be required in addition to or instead of the formal qualification. In some instances no formal qualification or on-the-job training may be required.

Tasks Include:
- contacting people face-to-face and via the telephone to conduct surveys
- recording answers to survey questions manually and electronically
- recording the distribution of questionnaires
- collecting questionnaires and returning them to supervisors
- scanning questionnaires to ensure that important questions have been answered
- may interview people at random in crowds and on the street
- may provide self-completion questionnaires
- may encode responses and check their consistency
- may work in a call centre

Occupation:
561511 Survey Interviewer

561511 SURVEY INTERVIEWER

Alternative Title:
Interviewer

Interviews people and records their responses to survey and market research questions on a range of topics. May work in a call centre.

Skill Level: 5

Specialisation:
Market Research Interviewer

UNIT GROUP 5616 SWITCHBOARD OPERATORS

SWITCHBOARD OPERATORS operate telecommunication switchboards and consoles to assist callers establish telephone connections, and receive caller inquiries and fault reports.

Indicative Skill Level:
Most occupations in this unit group have a level of skill commensurate with the qualifications outlined below.

In Australia:
AQF Certificate I, or compulsory secondary education (ANZSCO Skill Level 5)

In New Zealand:
NZ Register Level 1 qualification, or compulsory secondary education (ANZSCO Skill Level 5)

For some occupations a short period of on-the-job training may be required in addition to or instead of the formal qualification. In some instances no formal qualification or on-the-job training may be required.

Tasks Include:

- operating switchboards and consoles to connect, hold, transfer and disconnect telephone calls
- responding to callers' inquiries by providing information such as telephone numbers, dialling codes, call costs, time delays and service difficulties
- investigating operating system problems and informing maintenance services
- alerting emergency services when required
- recording details and determining charges for designated types of calls
- may monitor the efficiency of systems and maintain service sampling records

Occupation:
561611 Switchboard Operator

561611 SWITCHBOARD OPERATOR

Alternative Title:
Telephone Operator

Operates telecommunication switchboards and consoles to assist callers establish telephone connections, and receive caller inquiries and fault reports.

Skill Level: 5

UNIT GROUP 5619 OTHER CLERICAL AND OFFICE SUPPORT WORKERS

This unit group covers Clerical and Office Support Workers not elsewhere classified.

It includes Classified Advertising Clerks, Meter Readers and Parking Inspectors.

Indicative Skill Level:
Most occupations in this unit group have a level of skill commensurate with the qualifications outlined below.

In Australia:
AQF Certificate I, or compulsory secondary education (ANZSCO Skill Level 5)

In New Zealand:
NZ Register Level 1 qualification, or compulsory secondary education (ANZSCO Skill Level 5)

For some occupations a short period of on-the-job training may be required in addition to or instead of the formal qualification. In some instances no formal qualification or on-the-job training may be required.

Occupations:
561911 Classified Advertising Clerk
561912 Meter Reader
561913 Parking Inspector
561999 Clerical and Office Support Workers nec

561911 CLASSIFIED ADVERTISING CLERK

Receives and records advertising copy for publication and broadcasting.

Skill Level: 5

561912 METER READER

Reads electric, gas or water meters, records usage, inspects meters and connections for defects and damage, and reports irregularities.

Skill Level: 5

561913 PARKING INSPECTOR

Patrols assigned areas and issues parking infringement notices to owners of vehicles that are illegally parked.

Skill Level: 5

561999 CLERICAL AND OFFICE SUPPORT WORKERS NEC

This occupation group covers Clerical and Office Support Workers not elsewhere classified.

Skill Level: 5

Occupations in this group include:
Media Monitor (Aus)

SUB-MAJOR GROUP 59 OTHER CLERICAL AND ADMINISTRATIVE WORKERS

This sub-major group covers Clerical and Administrative Workers not elsewhere classified.

It includes Logistics Clerks.

Indicative Skill Level:
Most occupations in this sub-major group have a level of skill commensurate with the qualifications and experience outlined below.

In Australia:
- AQF Associate Degree, Advanced Diploma or Diploma, or at least three years of relevant experience (ANZSCO Skill Level 2); or
- AQF Certificate III including at least two years of on-the-job training, or AQF Certificate IV, or at least three years of relevant experience (ANZSCO Skill Level 3); or
- AQF Certificate II or III, or at least one year of relevant experience (ANZSCO Skill Level 4)

In New Zealand:
- NZ Register Diploma, or at least three years of relevant experience (ANZSCO Skill Level 2); or
- NZ Register Level 4 qualification, or at least three years of relevant experience (ANZSCO Skill Level 3); or
- NZ Register Level 2 or 3 qualification, or at least one year of relevant experience (ANZSCO Skill Level 4)

In some instances relevant experience and/or on-the-job training may be required in addition to the formal qualifications.

Occupations in this sub-major group are classified into the following minor groups:
- 591 Logistics Clerks
- 599 Miscellaneous Clerical and Administrative Workers

MINOR GROUP 591 LOGISTICS CLERKS

LOGISTICS CLERKS coordinate the purchasing, receipt, recording, monitoring, and distribution of goods and services, and the clearance and collection of imported cargo and shipment of cargo for export.

Indicative Skill Level:
Most occupations in this minor group have a level of skill commensurate with the qualifications and experience outlined below.

In Australia:
AQF Certificate II or III (ANZSCO Skill Level 4)

In New Zealand:
NZ Register Level 2 or 3 qualification (ANZSCO Skill Level 4)

At least one year of relevant experience may substitute for the formal qualifications listed above. In some instances relevant experience and/or on-the-job training may be required in addition to the formal qualification.

Tasks Include:
- receiving, checking and processing purchase requests
- verifying incoming and outgoing goods against records
- providing information about price, and calculating storage and clearance charges
- maintaining records of goods received and despatched
- recording customs clearance requirements and authorising collection of cargo
- organising despatch and collection of goods

Occupations in this minor group are classified into the following unit groups:
5911 Purchasing and Supply Logistics Clerks
5912 Transport and Despatch Clerks

UNIT GROUP 5911 PURCHASING AND SUPPLY LOGISTICS CLERKS

PURCHASING AND SUPPLY LOGISTICS CLERKS prepare and process orders for goods and services, monitor stock levels and supply sources and maintain stock and inventory levels, record and coordinate the flow of materials between departments, prepare production schedules, and administer and coordinate storage and distribution operations within organisations.

Indicative Skill Level:
Most occupations in this unit group have a level of skill commensurate with the qualifications and experience outlined below.

In Australia:
AQF Certificate II or III (ANZSCO Skill Level 4)

In New Zealand:
NZ Register Level 2 or 3 qualification (ANZSCO Skill Level 4)

At least one year of relevant experience may substitute for the formal qualifications listed above. In some instances relevant experience and/or on-the-job training may be required in addition to the formal qualification.

Tasks Include:

- requisitioning supplies from stock and sending orders to production departments and other firms
- confirming completion of orders and compliance with details specified, signing tally sheets and attaching to checked items
- receiving and checking purchase requests against inventory records and stock on hand
- examining orders and compiling data for production schedules
- checking inventories and preparing delivery schedules
- examining containers to ensure that they are filled, and recording quantities
- investigating and identifying supply sources and preparing and processing purchase orders
- providing price and other information about goods to prospective customers
- counting incoming stock and reconciling it with requisitions, and updating inventory and stock location records
- establishing and coordinating the operating procedures for receiving, handling, storing and shipping goods

Occupations:
591111 Order Clerk
591112 Production Clerk
591113 Purchasing Officer
591114 Sales Clerk
591115 Stock Clerk
591116 Warehouse Administrator

591111 ORDER CLERK

Alternative Title:
Customer Orders Clerk

Receives purchase requests for good and services, checks requests against inventory records and stock, and processes orders.

Skill Level: 4

Specialisation:
Mail Order Clerk

UNIT GROUP 5911 PURCHASING AND SUPPLY LOGISTICS CLERKS *continued*

591112 PRODUCTION CLERK

Alternative Titles:
- Production Recorder
- Schedule Clerk

Records and coordinates the flow of work and materials between departments, examines orders for goods, and prepares production schedules.

Skill Level: 4

Specialisations:
- Delivery Clerk
- Logistics Clerk

591113 PURCHASING OFFICER

Alternative Title:
- Procurement Clerk

Prepares purchase orders, monitors supply sources and negotiates contracts with suppliers.

Skill Level: 4

591114 SALES CLERK

Alternative Title:
- Internal Salesperson (Aus)

Receives and processes purchase orders for goods and services, and provides information and advice about goods and services.

Skill Level: 4

Specialisations:
- Engineering Sales Clerk
- Lay-by Clerk (Aus)

591115 STOCK CLERK

Alternative Titles:
- Stock Control Clerk
- Stores Clerk

Monitors stock levels and maintains stock, order and inventory records.

Skill Level: 4

Specialisations:
- Inventory Clerk
- Supply Clerk

591116 WAREHOUSE ADMINISTRATOR

Administers and coordinates storage and distribution operations within an organisation.

Skill Level: 4

UNIT GROUP 5912 TRANSPORT AND DESPATCH CLERKS

TRANSPORT AND DESPATCH CLERKS verify and maintain records of incoming and outgoing goods, prepare goods for despatch, arrange clearance and collection of imported cargo from customs and bond stores, and arrange shipment of cargo for export.

Indicative Skill Level:
Most occupations in this unit group have a level of skill commensurate with the qualifications and experience outlined below.

In Australia:
AQF Certificate II or III (ANZSCO Skill Level 4)

In New Zealand:
NZ Register Level 2 or 3 qualification (ANZSCO Skill Level 4)

At least one year of relevant experience may substitute for the formal qualifications listed above. In some instances relevant experience and/or on-the-job training may be required in addition to the formal qualification.

Tasks Include:
- identifying items and containers of incoming and outgoing shipments and verifying them against consignment records
- ensuring outgoing shipments are in good condition and meet specifications
- arranging internal distribution of goods received
- organising the despatch of goods with completed documentation
- maintaining prescribed records of goods received and despatched
- examining shipping documents and verifying cargo to be released
- recording customs clearance requirements and authorising collection of cargo
- calculating storage and clearance charges and billing customers
- receiving details of outgoing cargo, and arranging bookings of freight space and collection of goods from customers
- providing information to customers on custom tariffs, tariff classifications and concessions, and methods of clearing goods

Occupations:
591211 Despatching and Receiving Clerk
591212 Import-Export Clerk

591211 DESPATCHING AND RECEIVING CLERK

Alternative Titles:
Despatch Clerk
Freight Clerk

Verifies and maintains records of incoming and outgoing goods in a warehouse or distribution centre and prepares goods for despatch.

Skill Level: 4

Specialisations:
Aircraft Load Controller
Shipping and Receiving Clerk
Truck Despatcher

UNIT GROUP 5912 TRANSPORT AND DESPATCH CLERKS *continued*

591212 IMPORT-EXPORT CLERK

Alternative Title:
Customs Broker

Arranges the clearance and collection of imported cargo from customs and bond stores, and the shipment of cargo for export.

Skill Level: 4

Specialisations:
Bond Clerk
Customs Agent
Wharf Tally Clerk

MINOR GROUP 599 MISCELLANEOUS CLERICAL AND ADMINISTRATIVE WORKERS

This minor group covers Clerical and Administrative Workers not elsewhere classified.

It includes Conveyancers and Legal Executives, Court and Legal Clerks, Debt Collectors, Human Resource Clerks, Inspectors and Regulatory Officers, Insurance Investigators, Loss Adjusters and Risk Surveyors, and Library Assistants.

Indicative Skill Level:
Most occupations in this minor group have a level of skill commensurate with the qualifications and experience outlined below.

In Australia:
- AQF Associate Degree, Advanced Diploma or Diploma, or at least three years of relevant experience (ANZSCO Skill Level 2); or
- AQF Certificate III including at least two years of on-the-job training, or AQF Certificate IV, or at least three years of relevant experience (ANZSCO Skill Level 3); or
- AQF Certificate II or III, or at least one year of relevant experience (ANZSCO Skill Level 4)

In New Zealand:
- NZ Register Diploma, or at least three years of relevant experience (ANZSCO Skill Level 2); or
- NZ Register Level 4 qualification, or at least three years of relevant experience (ANZSCO Skill Level 3); or
- NZ Register Level 2 or 3 qualification, or at least one year of relevant experience (ANZSCO Skill Level 4)

In some instances relevant experience and/or on-the-job training may be required in addition to the formal qualifications.

Occupations in this minor group are classified into the following unit groups:
- 5991 Conveyancers and Legal Executives
- 5992 Court and Legal Clerks
- 5993 Debt Collectors
- 5994 Human Resource Clerks
- 5995 Inspectors and Regulatory Officers
- 5996 Insurance Investigators, Loss Adjusters and Risk Surveyors
- 5997 Library Assistants
- 5999 Other Miscellaneous Clerical and Administrative Workers

UNIT GROUP 5991 CONVEYANCERS AND LEGAL EXECUTIVES

CONVEYANCERS AND LEGAL EXECUTIVES act for and on behalf of clients in the areas of property transfer, company and business law, trusts, wills, probate and litigation.

Indicative Skill Level:
Most occupations in this unit group have a level of skill commensurate with the qualifications and experience outlined below.

In Australia:
AQF Associate Degree, Advanced Diploma or Diploma (ANZSCO Skill Level 2)

In New Zealand:
NZ Register Diploma (ANZSCO Skill Level 2)

At least three years of relevant experience may substitute for the formal qualifications listed above. In some instances relevant experience and/or on-the-job training may be required in addition to the formal qualification.

Registration or licensing may be required.

Tasks Include:
- preparing, examining and advising on contracts of sale for properties and businesses
- carrying out title searches and contacting government authorities to find out if any planned development, illegal building work and disputes could affect properties and businesses
- preparing, examining and advising on mortgage documentation
- negotiating the terms and conditions of, and exchanging, contracts of sale, paying deposits, arranging payment of stamp duty, checking for outstanding arrears and land tax obligations, and calculating adjustments for council and water rates
- interviewing clients to determine the nature of issues, and receiving written information concerning cases from Legal Professionals and clients
- conferring with clients and potential witnesses and drawing up statements and proposed affidavits in preparation for court proceedings
- maintaining legal files
- preparing, analysing and interpreting a variety of legal documents
- assisting Legal Professionals to prepare cases for court by conducting investigations, undertaking research, arranging witness preparation and attendance, and preparing and filing court documents
- may supervise Law Clerks and Legal Secretaries and take responsibility for their work

Occupations:
599111 Conveyancer
599112 Legal Executive

599111 CONVEYANCER

Alternative Title:
Settlement Agent

Acts for and on behalf of clients in the area of property and business transfers. Registration or licensing may be required.

Skill Level: 2

UNIT GROUP 5991 CONVEYANCERS AND LEGAL EXECUTIVES *continued*

599112 LEGAL EXECUTIVE

Acts for and on behalf of clients in the areas of property transfer, company and business law, trusts, wills, probate and litigation under the general supervision of a Barrister or Solicitor. Registration or licensing may be required.

Skill Level: 2

UNIT GROUP 5992 COURT AND LEGAL CLERKS

COURT AND LEGAL CLERKS provide administrative and operational support to Legal Professionals by performing clerical work associated with the functions of courts, legal practices and the administration of trusts and estates.

Legal Secretaries, Court Reporters and Legal Executives are excluded from this unit group. Legal Secretaries are included in Unit Group 5212 Secretaries. Court Reporters are included in Unit Group 5321, in Occupation 532112 Machine Shorthand Reporter. Legal Executives are included in Unit Group 5991 Conveyancers and Legal Executives.

Indicative Skill Level:
Most occupations in this unit group have a level of skill commensurate with the qualifications and experience outlined below.

In Australia:
AQF Certificate III including at least two years of on-the-job training, or AQF Certificate IV (ANZSCO Skill Level 3)

In New Zealand:
NZ Register Level 4 qualification (ANZSCO Skill Level 3)

At least three years of relevant experience may substitute for the formal qualifications listed above. In some instances relevant experience and/or on-the-job training may be required in addition to the formal qualification.

Tasks Include:
- listing actions for hearing and processing documentation for court actions
- documenting details of court proceedings, actions and decisions
- enforcing the law as an officer of the court by executing court orders such as eviction notices
- serving legal orders and documents such as summonses and subpoenas
- organising jury and witness lists, and summonsing and swearing in juries and witnesses
- maintaining order in court and hearing rooms and adjacent areas
- assisting Solicitors in areas of conveyancing, contracts, common law, probate and other legal practice matters
- satisfying statutory requirements, establishing beneficial entitlements and distributing assets
- maintaining probate and trust files, investing trust funds and administering accounts

Occupations:
599211 Clerk of Court
599212 Court Bailiff or Sheriff (Aus) / Court Collections Officer (NZ)
599213 Court Orderly (Aus) / Court Registry Officer (NZ)
599214 Law Clerk
599215 Trust Officer

599211 CLERK OF COURT

Administers court registry services and performs administrative functions in support of Judges and Magistrates.

Skill Level: 3

UNIT GROUP 5992 COURT AND LEGAL CLERKS *continued*

599212 COURT BAILIFF OR SHERIFF (AUS) / COURT COLLECTIONS OFFICER (NZ)

Implements court orders and serves legal orders and summonses as an officer of the court.

Skill Level: 3

Specialisation:
Sheriff's Officer (Aus)

599213 COURT ORDERLY (AUS) / COURT REGISTRY OFFICER (NZ)

Alternative Titles:
Court Attendant
Court Officer

Provides operational support to a court or registry.

Skill Level: 3

Specialisation:
Court Usher

599214 LAW CLERK

Alternative Title:
Legal Clerk

Performs specialised clerical work associated with legal practice and law courts.

Skill Level: 3

599215 TRUST OFFICER

Alternative Title:
Trust Clerk

Administers trusts, estates and settlements on behalf of beneficiaries.

Skill Level: 3

UNIT GROUP 5993 DEBT COLLECTORS

DEBT COLLECTORS collect consumer, commercial, insurance and other forms of debt for clients, make arrangements to settle overdue accounts, formalise payment arrangements and follow up until accounts are fully paid.

Indicative Skill Level:
Most occupations in this unit group have a level of skill commensurate with the qualifications and experience outlined below.

In Australia:
AQF Certificate II or III (ANZSCO Skill Level 4)

In New Zealand:
NZ Register Level 2 or 3 qualification (ANZSCO Skill Level 4)

At least one year of relevant experience may substitute for the formal qualifications listed above. In some instances relevant experience and/or on-the-job training may be required in addition to the formal qualification.

Registration or licensing may be required.

Tasks Include:
- liaising with clients, credit staff, accounts receivable departments, process servers, Private Investigators, Barristers and Solicitors to find solutions to payment problems
- identifying, locating and notifying debtors of overdue accounts in writing, by telephoning and in person, and arranging for payments to be made
- tracing addresses of debtors who have moved
- arranging new repayment plans for debtors having difficulties making existing repayments
- referring debtors' disputes to creditors
- issuing instructions for the commencement of legal action and enforcement to recover money
- arranging for money and goods collected to be transferred to creditors' possession, and preparing statements of account for creditors
- recording amounts collected and noting any further action required
- complying with debt collection guidelines and relevant legislation

Occupation:
599311 Debt Collector

599311 DEBT COLLECTOR

Alternative Titles:
Debt Recovery Officer
Mercantile Agent (Aus)

Collects consumer, commercial, insurance and other forms of debt for clients, makes arrangements to settle overdue accounts, formalises payment arrangements and follows up until accounts are fully paid. Registration or licensing may be required.

Skill Level: 4

Specialisations:
Collection Agent
Collection Officer
Repossession Agent

UNIT GROUP 5994 HUMAN RESOURCE CLERKS

HUMAN RESOURCE CLERKS maintain and update personnel records such as information on transfers and promotions, employee leave taken and accumulated, salaries, superannuation and taxation, qualifications and training.

Indicative Skill Level:
Most occupations in this unit group have a level of skill commensurate with the qualifications and experience outlined below.

In Australia:
AQF Certificate II or III (ANZSCO Skill Level 4)

In New Zealand:
NZ Register Level 2 or 3 qualification (ANZSCO Skill Level 4)

At least one year of relevant experience may substitute for the formal qualifications listed above. In some instances relevant experience and/or on-the-job training may be required in addition to the formal qualification.

Tasks Include:
- updating information on leave taken and accumulated, employment history, salaries, superannuation and taxation, qualifications and training
- raising records for newly appointed workers and checking records for completeness
- processing applications for employment and promotions and advising applicants of results
- receiving and answering inquiries about employment entitlements and conditions
- sending out announcements of job openings and job examinations
- issuing job application forms
- compiling data from personnel records and preparing reports
- storing and retrieving personnel records and files on request

Occupation:
599411 Human Resource Clerk

599411 HUMAN RESOURCE CLERK

Alternative Titles:
Employment Office Clerk
Human Resources Records Clerk
Personnel Records Clerk

Maintains and updates personnel records such as information on transfers and promotions, employee leave taken and accumulated, salaries, superannuation and taxation, qualifications and training.

Skill Level: 4

Specialisations:
Psychological Examiner (Army)
Roster Clerk

UNIT GROUP 5995 INSPECTORS AND REGULATORY OFFICERS

INSPECTORS AND REGULATORY OFFICERS administer and enforce government and corporate regulations and standards.

Indicative Skill Level:
Most occupations in this unit group have a level of skill commensurate with the qualifications and experience outlined below.

In Australia:
AQF Certificate II or III (ANZSCO Skill Level 4)

In New Zealand:
NZ Register Level 2 or 3 qualification (ANZSCO Skill Level 4)

At least one year of relevant experience may substitute for the formal qualifications listed above. In some instances relevant experience and/or on-the-job training may be required in addition to the formal qualification.

Registration or licensing may be required.

Tasks Include:

- searching aircraft, vehicles, premises and people, and checking documents and goods to detect illegal activities such as undocumented cargo, prohibited goods and illegal aliens
- examining and assessing visas and residency applications
- testing applicants' ability to operate a motor vehicle, assessing applicants' suitability to hold learner's permits and probationary licences, and issuing learner's permits and probationary licences
- identifying pest and weed problems and determining treatments and management
- assessing claims for government benefits
- carrying out random checks of taxation documents to detect non-compliance with taxation legislation
- conducting visual checks of the mechanical, structural, electrical, pneumatic and hydraulic systems of railway wagons, carriages and locomotives for condition and correct classification
- ensuring that train, tram and bus services are provided according to schedule, monitoring the cleanliness, presentation and condition of vehicles, and recommending improvements and changes to services
- receiving and assessing applications for licences to use water, investigating the ability of water resources to meet new requirements, and conducting site inspections

Occupations:
599511 Customs Officer
599512 Immigration Officer
599513 Motor Vehicle Licence Examiner
599514 Noxious Weeds and Pest Inspector
599515 Social Security Assessor
599516 Taxation Inspector
599517 Train Examiner
599518 Transport Operations Inspector
599521 Water Inspector
599599 Inspectors and Regulatory Officers nec

UNIT GROUP 5995 INSPECTORS AND REGULATORY OFFICERS *continued*

599511 CUSTOMS OFFICER

Alternative Title:
Customs Inspector

Administers and enforces customs and related legislation, and assists with customs control of overseas passengers, crew, aircraft, ships, cargo, mail and bond stores.

Skill Level: 4

Specialisation:
Customs Investigator

599512 IMMIGRATION OFFICER

Examines and assesses the entry of people from other countries, administers visas and residency applications according to immigration legislation, rules and policies, and, where necessary, uses legal powers to detain and remove illegal entrants.

Skill Level: 4

599513 MOTOR VEHICLE LICENCE EXAMINER

Tests motor vehicle driving licence applicants and issues learner's permits and probationary licences. Registration or licensing is required.

Skill Level: 4

599514 NOXIOUS WEEDS AND PEST INSPECTOR

Alternative Title:
Biosecurity Officer (Weeds and Pests)

Inspects and monitors plants, land and water for noxious plants and animal species, and organises for their control or eradication.

Skill Level: 4

599515 SOCIAL SECURITY ASSESSOR

Assesses social welfare claims and entitlements under government legislation and investigates fraud and suspected breaches of legislation.

Skill Level: 4

599516 TAXATION INSPECTOR

Inspects and assesses taxation returns to ensure compliance with government legislation, and investigates suspected breaches of taxation legislation.

Skill Level: 4

599517 TRAIN EXAMINER

Inspects rolling stock in railway yards, terminals and stations to ensure adherence to safety standards and operational rules and regulations.

Skill Level: 4

Specialisation:
Locomotive Inspector

UNIT GROUP 5995 INSPECTORS AND REGULATORY OFFICERS *continued*

599518 TRANSPORT OPERATIONS INSPECTOR

Monitors scheduled train, tram and bus services and investigates accidents, complaints and service disruptions.

Skill Level: 4

Specialisations:
- Bus Inspector
- Tram Inspector

599521 WATER INSPECTOR

Monitors the allocation and use of water from water resources such as streams, rivers and underground sources.

Skill Level: 4

Specialisations:
- Boring Inspector
- Stream Control Officer

599599 INSPECTORS AND REGULATORY OFFICERS NEC

This occupation group covers Inspectors and Regulatory Officers not elsewhere classified.

Skill Level: 4

Occupations in this group include:
- Dog Catcher
- Technician Preventative Medicine (Army)
- Trade Mark Examiner (Aus)
- Travel Accommodation Inspector
- Weights and Measures Inspector

UNIT GROUP 5996 INSURANCE INVESTIGATORS, LOSS ADJUSTERS AND RISK SURVEYORS

INSURANCE INVESTIGATORS, LOSS ADJUSTERS AND RISK SURVEYORS conduct investigations into insurance claims to ensure their validity, inspect and assess the damage and loss to insured properties and businesses, estimate insurance costs, and inspect insured properties to evaluate conditions affecting underwriting standards.

Indicative Skill Level:
Most occupations in this unit group have a level of skill commensurate with the qualifications and experience outlined below.

In Australia:

AQF Certificate III including at least two years of on-the-job training, or AQF Certificate IV (ANZSCO Skill Level 3)

In New Zealand:

NZ Register Level 4 qualification (ANZSCO Skill Level 3)

At least three years of relevant experience may substitute for the formal qualifications listed above. In some instances relevant experience and/or on-the-job training may be required in addition to the formal qualification.

Registration or licensing may be required.

Tasks Include:
- examining scenes of incidents resulting in insurance claims to determine causes and effects
- interviewing witnesses and claimants to obtain details required to assess the validity of claims and identify the parties responsible for accidents, damage and loss, and preparing statements and reports
- inspecting damaged buildings, equipment and motor vehicles and estimating the cost of repairs
- estimating business losses resulting from fire, theft and other business disruptions
- reporting the extent of damage and estimated costs to the insurer
- inspecting property, buildings and operations of commercial and industrial establishments to assess physical conditions and work practices
- evaluating the adequacy of security, fire and related systems
- preparing reports and recommending action to reduce risks
- compiling data which influence the determination of premium rates

Occupations:

599611 Insurance Investigator
599612 Insurance Loss Adjuster
599613 Insurance Risk Surveyor

599611 INSURANCE INVESTIGATOR

Conducts investigations into insurance claims to ensure their validity. Registration or licensing is required.

Skill Level: 3

UNIT GROUP 5996 INSURANCE INVESTIGATORS, LOSS ADJUSTERS AND RISK SURVEYORS *continued*

599612 INSURANCE LOSS ADJUSTER

Alternative Title:

Insurance Loss Assessor

Inspects and assesses the damage and loss to insured property and business, estimates insurance costs, and acts to minimise the cost of claims to an insurance company.

Skill Level: 3

599613 INSURANCE RISK SURVEYOR

Inspects items and properties to evaluate conditions affecting underwriting standards, and develops and promotes safety programs.

Skill Level: 3

UNIT GROUP 5997 LIBRARY ASSISTANTS

LIBRARY ASSISTANTS issue, receive and shelve library items and maintain associated records.

Indicative Skill Level:
Most occupations in this unit group have a level of skill commensurate with the qualifications and experience outlined below.

In Australia:
AQF Certificate II or III (ANZSCO Skill Level 4)

In New Zealand:
NZ Register Level 2 or 3 qualification (ANZSCO Skill Level 4)

At least one year of relevant experience may substitute for the formal qualifications listed above. In some instances relevant experience and/or on-the-job training may be required in addition to the formal qualification.

Tasks Include:
- issuing library items to borrowers and recording identification data and due dates
- sorting and shelving returned items
- locating and retrieving items on request
- maintaining records and index systems
- receiving overdue items, issuing overdue notices, and receiving fines
- inspecting returned items for damage and making minor repairs
- assisting with the preparation of displays and promotional activities
- may prepare catalogued items for shelving

Occupation:
599711 Library Assistant

599711 LIBRARY ASSISTANT

Alternative Titles:
Library Attendant
Library Clerk

Issues, receives and shelves library items and maintains associated records.

Skill Level: 4

UNIT GROUP 5999 OTHER MISCELLANEOUS CLERICAL AND ADMINISTRATIVE WORKERS

This unit group covers Clerical and Administrative Workers not elsewhere classified.

It includes Coding Clerks, Production Assistants (Film, Television, Radio or Stage), Proof Readers and Radio Despatchers.

Indicative Skill Level:
Most occupations in this unit group have a level of skill commensurate with the qualifications and experience outlined below.

In Australia:
AQF Certificate II or III (ANZSCO Skill Level 4)

In New Zealand:
NZ Register Level 2 or 3 qualification (ANZSCO Skill Level 4)

At least one year of relevant experience may substitute for the formal qualifications listed above. In some instances relevant experience and/or on-the-job training may be required in addition to the formal qualification.

Registration or licensing may be required.

Occupations:
599911 Coding Clerk
599912 Production Assistant (Film, Television, Radio or Stage)
599913 Proof Reader
599914 Radio Despatcher
599999 Clerical and Administrative Workers nec

599911 CODING CLERK

Translates narrative descriptions and numeric information into classification or record systems.

Skill Level: 4

Specialisations:
Clinical Coder
Medical Record Clerk

599912 PRODUCTION ASSISTANT (FILM, TELEVISION, RADIO OR STAGE)

Provides technical, administrative and organisational support to producers or directors for film, television, radio or stage productions.

Skill Level: 4

599913 PROOF READER

Reads draft copies and proofs, detects errors and marks corrections to grammar, typing and composition.

Skill Level: 4

UNIT GROUP 5999 OTHER MISCELLANEOUS CLERICAL AND ADMINISTRATIVE WORKERS *continued*

599914 RADIO DESPATCHER

Alternative Titles:

- Communications Controller
- Control Room Operator

Provides radio and communications services for the coordination of operational units in transport, courier, military, emergency, security, rescue and road service organisations. Registration or licensing may be required.

Skill Level: 4

599999 CLERICAL AND ADMINISTRATIVE WORKERS NEC

This occupation group covers Clerical and Administrative Workers not elsewhere classified.

Skill Level: 4

Occupations in this group include:

- Examination Supervisor
- Train Planner
- Travel Clerk

MAJOR GROUP 6 SALES WORKERS

SALES WORKERS sell goods, services and property, and provide sales support in areas such as operating cash registers and displaying and demonstrating goods.

ICT and Technical Sales Representatives are excluded from this major group. ICT Sales Representatives are included in Unit Group 2252 ICT Sales Professionals. Technical Sales Representatives are included in Unit Group 2254 Technical Sales Representatives.

Indicative Skill Level:
Most occupations in this major group have a level of skill commensurate with the qualifications and experience outlined below.

In Australia:
AQF Associate Degree, Advanced Diploma or Diploma, or at least three years of relevant experience (ANZSCO Skill Level 2); or
AQF Certificate III including at least two years of on-the-job training, or AQF Certificate IV, or at least three years of relevant experience (ANZSCO Skill Level 3); or
AQF Certificate II or III, or at least one year of relevant experience (ANZSCO Skill Level 4); or
AQF Certificate I, or compulsory secondary education (ANZSCO Skill Level 5)

In New Zealand:
NZ Register Diploma, or at least three years of relevant experience (ANZSCO Skill Level 2); or
NZ Register Level 4 qualification, or at least three years of relevant experience (ANZSCO Skill Level 3); or
NZ Register Level 2 or 3 qualification, or at least one year of relevant experience (ANZSCO Skill Level 4); or
NZ Register Level 1 qualification, or compulsory secondary education (ANZSCO Skill Level 5)

In some instances relevant experience and/or on-the-job training may be required in addition to the formal qualification. In the case of some Skill Level 5 occupations, a short period of on-the-job training may be required in addition to or instead of the formal qualification, or no formal qualification or on-the-job training may be required.

Tasks Include:
- promoting goods and services, properties and businesses to potential buyers
- selling goods and services, properties and businesses to buyers
- engaging prospective buyers
- determining buyers' requirements
- receiving and processing payments for goods and services, properties and businesses purchased by a variety of payment methods

Occupations in this major group are classified into the following sub-major groups:
61 Sales Representatives and Agents
62 Sales Assistants and Salespersons
63 Sales Support Workers

SUB-MAJOR GROUP 61 SALES REPRESENTATIVES AND AGENTS

SALES REPRESENTATIVES AND AGENTS represent companies in selling their goods and services, and sell real estate and other property on behalf of clients.

ICT and Technical Sales Representatives are excluded from this sub-major group. ICT Sales Representatives are included in Unit Group 2252 ICT Sales Professionals. Technical Sales Representatives are included in Unit Group 2254 Technical Sales Representatives.

Indicative Skill Level:
Most occupations in this sub-major group have a level of skill commensurate with the qualifications and experience outlined below.

In Australia:
AQF Associate Degree, Advanced Diploma or Diploma, or at least three years of relevant experience (ANZSCO Skill Level 2); or
AQF Certificate III including at least two years of on-the-job training, or AQF Certificate IV, or at least three years of relevant experience (ANZSCO Skill Level 3); or
AQF Certificate II or III, or at least one year of relevant experience (ANZSCO Skill Level 4)

In New Zealand:
NZ Register Diploma, or at least three years of relevant experience (ANZSCO Skill Level 2); or
NZ Register Level 4 qualification, or at least three years of relevant experience (ANZSCO Skill Level 3); or
NZ Register Level 2 or 3 qualification, or at least one year of relevant experience (ANZSCO Skill Level 4)

In some instances relevant experience and/or on-the-job training may be required in addition to the formal qualification.

Tasks Include:
- promoting and selling goods and services, properties and businesses
- engaging prospective clients
- determining the needs of prospective clients and explaining which goods, services and properties meet their needs
- visiting clients to establish selling opportunities
- following up clients and gauging satisfaction with goods and services purchased
- monitoring clients' changing needs and competitor activity

Occupations in this sub-major group are classified into the following minor groups:
611 Insurance Agents and Sales Representatives
612 Real Estate Sales Agents

MINOR GROUP 611 INSURANCE AGENTS AND SALES REPRESENTATIVES

INSURANCE AGENTS AND SALES REPRESENTATIVES represent companies in selling their goods and services, and sell property on behalf of clients.

ICT and Technical Sales Representatives are excluded from this minor group. ICT Sales Representatives are included in Unit Group 2252 ICT Sales Professionals. Technical Sales Representatives are included in Unit Group 2254 Technical Sales Representatives.

Indicative Skill Level:
Most occupations in this minor group have a level of skill commensurate with the qualifications and experience outlined below.

In Australia:

AQF Certificate III including at least two years of on-the-job training, or AQF Certificate IV, or at least three years of relevant experience (ANZSCO Skill Level 3); or
AQF Certificate II or III, or at least one year of relevant experience (ANZSCO Skill Level 4)

In New Zealand:

NZ Register Level 4 qualification, or at least three years of relevant experience (ANZSCO Skill Level 3); or
NZ Register Level 2 or 3 qualification, or at least one year of relevant experience (ANZSCO Skill Level 4)

In some instances relevant experience and/or on-the-job training may be required in addition to the formal qualification.

Tasks Include:

- assisting clients to sell property by auction, and buy and sell livestock, rural equipment, and goods and services
- compiling lists of prospective clients and making contact to seek interviews and gauge interest
- determining the needs of prospective clients and explaining which goods and services would meet their needs
- informing and supplying details to clients about goods and services for sale
- selling a range of goods and services to clients
- keeping up-to-date with clients' changing needs and competitor activity

Occupations in this minor group are classified into the following unit groups:

6111 Auctioneers, and Stock and Station Agents
6112 Insurance Agents
6113 Sales Representatives

UNIT GROUP 6111 AUCTIONEERS, AND STOCK AND STATION AGENTS

AUCTIONEERS, AND STOCK AND STATION AGENTS sell property at auction, and advise and represent farmers in business transactions such as buying and selling livestock, rural property, and goods and services.

Indicative Skill Level:
Most occupations in this unit group have a level of skill commensurate with the qualifications and experience outlined below.

In Australia:
AQF Certificate III including at least two years of on-the-job training, or AQF Certificate IV (ANZSCO Skill Level 3)

In New Zealand:
NZ Register Level 4 qualification (ANZSCO Skill Level 3)

At least three years of relevant experience may substitute for the formal qualifications listed above. In some instances relevant experience and/or on-the-job training may be required in addition to the formal qualification.

Registration or licensing may be required.

Tasks Include:
- appraising and listing property for auction
- organising advertising, catalogues and other publicity for auctions
- consulting vendors and setting reserve prices
- describing property presented and the conditions of sale
- asking for or setting opening bids and determining reserve prices
- accepting bids from potential buyers and closing sales to the highest bidders
- purchasing and selling livestock and rural property on behalf of clients
- selling agricultural supplies, such as seed, grains, feed, sprays, dips, drenches and veterinary products, in accordance with statutory requirements
- acting as an insurance agent for rural clients

Occupations:
611111 Auctioneer
611112 Stock and Station Agent

611111 AUCTIONEER

Conducts sales of real estate, goods and livestock by taking offers from buyers and accepting the highest purchase price. Registration or licensing is required.

Skill Level: 3

611112 STOCK AND STATION AGENT

Provides advice to clients and acts on their behalf in relation to the sale and purchase of rural property, livestock, crops and agricultural products and services. Registration or licensing may be required.

Skill Level: 3

UNIT GROUP 6112 INSURANCE AGENTS

INSURANCE AGENTS represent insurance companies in selling insurance to clients.

Indicative Skill Level:
Most occupations in this unit group have a level of skill commensurate with the qualifications and experience outlined below.

In Australia:
AQF Certificate III including at least two years of on-the-job training, or AQF Certificate IV (ANZSCO Skill Level 3)

In New Zealand:
NZ Register Level 4 qualification (ANZSCO Skill Level 3)

At least three years of relevant experience may substitute for the formal qualifications listed above. In some instances relevant experience and/or on-the-job training may be required in addition to the formal qualification.

Registration or licensing is required.

Tasks Include:
- interviewing clients to identify their insurance needs
- explaining to clients details of insurance and conditions, risk coverage, premiums and benefits
- assisting clients to determine the type and level of coverage required
- calculating premiums and establishing method of payment
- reviewing clients' circumstances to ensure that the level and coverage of insurance is still appropriate
- settling and monitoring insurance claims to ensure that both client and insurer are satisfied with the outcome
- recording information about clients and their policies
- identifying and drawing up lists of potential clients from a variety of sources and contacting them to arrange interviews
- keeping up-to-date with changes in the insurance industry and informing clients of new developments

Occupation:
611211 Insurance Agent

611211 INSURANCE AGENT

Represents insurance companies in selling insurance to clients. Registration or licensing is required.

Skill Level: 3

Specialisations:
Insurance Underwriter
Life Assurance Representative

UNIT GROUP 6113 SALES REPRESENTATIVES

SALES REPRESENTATIVES represent companies to sell their goods and business services to wholesale and retail establishments.

ICT and Technical Sales Representatives are excluded from this unit group. ICT Sales Representatives are included in Unit Group 2252 ICT Sales Professionals. Technical Sales Representatives are included in Unit Group 2254 Technical Sales Representatives.

Indicative Skill Level:
Most occupations in this unit group have a level of skill commensurate with the qualifications and experience outlined below.

In Australia:
AQF Certificate II or III (ANZSCO Skill Level 4)

In New Zealand:
NZ Register Level 2 or 3 qualification (ANZSCO Skill Level 4)

At least one year of relevant experience may substitute for the formal qualifications listed above. In some instances relevant experience and/or on-the-job training may be required in addition to the formal qualification.

Tasks Include:
- promoting and selling their company's goods and services such as building and plumbing supplies, business services, motor vehicle parts and accessories, and personal and household goods
- acquiring and updating knowledge of employer's and competitors' goods and services, and market conditions
- using directories and other sources to compile lists of prospective business clients
- visiting clients and retail outlets to establish selling opportunities
- quoting prices and credit terms, recording orders and arranging deliveries
- following up clients and ensuring satisfaction with goods and services and resolving any problems
- monitoring clients' changing needs and competitor activity and reporting on these developments to sales and marketing management
- preparing sales reports
- maintaining and submitting records of business expenses incurred

Occupations:
611311 Sales Representative (Building and Plumbing Supplies)
611312 Sales Representative (Business Services)
611313 Sales Representative (Motor Vehicle Parts and Accessories)
611314 Sales Representative (Personal and Household Goods)
611399 Sales Representatives nec

611311 SALES REPRESENTATIVE (BUILDING AND PLUMBING SUPPLIES)

Represents their company in selling builders' timber, and building and plumbing hardware and supplies to wholesale and retail establishments.

Skill Level: 4

UNIT GROUP 6113 SALES REPRESENTATIVES *continued*

611312 SALES REPRESENTATIVE (BUSINESS SERVICES)

Represents their company in selling financial, advertising and other business services.

Skill Level: 4

Specialisations:
- Sales Representative (Advertising)
- Sales Representative (Printing)

611313 SALES REPRESENTATIVE (MOTOR VEHICLE PARTS AND ACCESSORIES)

Represents their company in selling motor vehicle parts and accessories to wholesale and retail establishments.

Skill Level: 4

611314 SALES REPRESENTATIVE (PERSONAL AND HOUSEHOLD GOODS)

Represents their company in selling consumer goods, such as toys, sporting goods, books, stationery, hardware, floor coverings, furniture, textiles, clothing, footwear, toiletries and groceries, to wholesale and retail establishments.

Skill Level: 4

611399 SALES REPRESENTATIVES NEC

This occupation group covers Sales Representatives not elsewhere classified.

Skill Level: 4

Occupations in this group include:
- Sales Representative (Jewellery and Watches)
- Sales Representative (Musical Goods)
- Sales Representative (Photographic Equipment and Supplies)

MINOR GROUP 612 REAL ESTATE SALES AGENTS

REAL ESTATE SALES AGENTS sell, lease and manage commercial and private properties, and broker the buying and selling of businesses.

Indicative Skill Level:
Most occupations in this minor group have a level of skill commensurate with the qualifications and experience outlined below.

In Australia:

AQF Associate Degree, Advanced Diploma or Diploma, or at least three years of relevant experience (ANZSCO Skill Level 2); or

AQF Certificate III including at least two years of on-the-job training, or AQF Certificate IV, or at least three years of relevant experience (ANZSCO Skill Level 3)

In New Zealand:

NZ Register Diploma, or at least three years of relevant experience (ANZSCO Skill Level 2); or

NZ Register Level 4 qualification, or at least three years of relevant experience (ANZSCO Skill Level 3)

In some instances relevant experience and/or on-the-job training may be required in addition to the formal qualification.

Tasks Include:

- accepting and listing properties and businesses for sale and lease, conducting inspections, and advising buyers on the merits of properties and businesses and the terms of sale or lease
- advising vendors of sales and marketing options such as sale by auction and open house inspections
- cataloguing and detailing land, buildings and businesses for sale or lease, and arranging advertising
- assessing buyers' needs and locating properties and businesses for their consideration
- offering valuations and advice for buying and selling properties and businesses, and structuring the terms of settlement
- collecting and holding rent monies from tenants, and remitting to owner on agreed basis
- monitoring and addressing non-compliance with terms and conditions of tenancy and pursuing rental arrears
- developing and implementing business plans, budgets, policies and procedures for the agency
- may arrange finance, land brokerage, conveyancing and maintenance of premises

Occupations in this minor group are classified into the following unit group:

6121 Real Estate Sales Agents

UNIT GROUP 6121 REAL ESTATE SALES AGENTS

REAL ESTATE SALES AGENTS sell, lease and manage commercial and private properties, and broker the buying and selling of businesses.

Indicative Skill Level:
Most occupations in this unit group have a level of skill commensurate with the qualifications and experience outlined below.

In Australia:
AQF Certificate III including at least two years of on-the-job training, or AQF Certificate IV (ANZSCO Skill Level 3)

In New Zealand:
NZ Register Level 4 qualification (ANZSCO Skill Level 3)

At least three years of relevant experience may substitute for the formal qualifications listed above. In some instances relevant experience and/or on-the-job training may be required in addition to the formal qualification.

Registration or licensing may be required.

The occupation Real Estate Agency Principal (Aus) / Real Estate Agency Licensee (NZ) has a level of skill commensurate with the qualifications and experience outlined below.

In Australia:
AQF Associate Degree, Advanced Diploma or Diploma (ANZSCO Skill Level 2)

In New Zealand:
NZ Register Diploma (ANZSCO Skill Level 2)

At least three years of relevant experience may substitute for the formal qualifications listed above. In some instances relevant experience and/or on-the-job training may be required in addition to the formal qualification.

Tasks Include:
- accepting and listing properties and businesses for sale and lease, conducting inspections, and advising buyers on the merits of properties and businesses and the terms of sale or lease
- advising vendors of sales and marketing options such as sale by auction and open house inspections
- cataloguing and detailing land, buildings and businesses for sale or lease and arranging advertising
- assessing buyers' needs and locating properties and businesses for their consideration
- offering valuations and advice for buying and selling properties and businesses, and structuring the terms of settlement
- collecting and holding rent monies from tenants, and remitting to owner on agreed basis
- monitoring and addressing non-compliance with terms and conditions of tenancy and pursuing rental arrears
- developing and implementing business plans, budgets, policies and procedures for the agency
- may arrange finance, land brokerage, conveyancing and maintenance of premises

Occupations:
612111 Business Broker
612112 Property Manager
612113 Real Estate Agency Principal (Aus) / Real Estate Agency Licensee (NZ)
612114 Real Estate Agent
612115 Real Estate Representative

UNIT GROUP 6121 REAL ESTATE SALES AGENTS *continued*

612111 BUSINESS BROKER

Alternative Title:

Business Agent

Operates as an independent agent in the buying and selling of businesses. Registration or licensing may be required.

Skill Level: 3

Specialisation:

Franchise Broker

612112 PROPERTY MANAGER

Supervises the leasing of rental properties on behalf of owners. Registration or licensing may be required.

Skill Level: 3

Specialisation:

Body Corporate Manager

612113 REAL ESTATE AGENCY PRINCIPAL (AUS) / REAL ESTATE AGENCY LICENSEE (NZ)

Manages the overall activities of a real estate agency. Registration or licensing is required.

Skill Level: 2

612114 REAL ESTATE AGENT

Coordinates the activities of real estate representatives in selling and leasing real estate, ensuring compliance with legislative requirements. Registration or licensing is required.

Skill Level: 3

612115 REAL ESTATE REPRESENTATIVE

Alternative Titles:

Real Estate Salesperson

Real Estate Subagent

Arranges the conduct of real estate transactions such as sales and leasing, and assists buyers to find suitable properties, on behalf of an agency. Registration or licensing is required.

Skill Level: 3

Specialisation:

Property Portfolio Officer

SUB-MAJOR GROUP 62 SALES ASSISTANTS AND SALESPERSONS

SALES ASSISTANTS AND SALESPERSONS sell a range of goods and services directly to the public on behalf of retail and wholesale establishments.

Indicative Skill Level:
Most occupations in this sub-major group have a level of skill commensurate with the qualifications and experience outlined below.

In Australia:
AQF Certificate II or III, or at least one year of relevant experience (ANZSCO Skill Level 4); or
AQF Certificate I, or compulsory secondary education (ANZSCO Skill Level 5)

In New Zealand:
NZ Register Level 2 or 3 qualification, or at least one year of relevant experience (ANZSCO Skill Level 4); or
NZ Register Level 1 qualification, or compulsory secondary education (ANZSCO Skill Level 5)

In some instances relevant experience and/or on-the-job training may be required in addition to the formal qualification. In the case of some Skill Level 5 occupations, a short period of on-the-job training may be required in addition to or instead of the formal qualification, or no formal qualification or on-the-job training may be required.

Tasks Include:

- determining customer requirements and advising on product range, price, delivery, warranties and product use and care
- demonstrating and explaining to customers the establishment's goods and services
- selling goods and services
- accepting payment for goods and services by a variety of payment methods and preparing sales invoices
- assisting with the ongoing management of stock such as product inventories and participating in stocktakes
- stacking and displaying goods for sale, and wrapping and packing goods sold

Occupations in this sub-major group are classified into the following minor group:
621 Sales Assistants and Salespersons

MINOR GROUP 621 SALES ASSISTANTS AND SALESPERSONS

SALES ASSISTANTS AND SALESPERSONS sell a range of goods and services directly to the public on behalf of retail and wholesale establishments.

Indicative Skill Level:
Most occupations in this minor group have a level of skill commensurate with the qualifications and experience outlined below.

In Australia:
AQF Certificate II or III, or at least one year of relevant experience (ANZSCO Skill Level 4); or
AQF Certificate I, or compulsory secondary education (ANZSCO Skill Level 5)

In New Zealand:
NZ Register Level 2 or 3 qualification, or at least one year of relevant experience (ANZSCO Skill Level 4); or
NZ Register Level 1 qualification, or compulsory secondary education (ANZSCO Skill Level 5)

In some instances relevant experience and/or on-the-job training may be required in addition to the formal qualification. In the case of some Skill Level 5 occupations, a short period of on-the-job training may be required in addition to or instead of the formal qualification, or no formal qualification or on-the-job training may be required.

Tasks Include:
- determining customer requirements and advising on product range, price, delivery, warranties and product use and care
- demonstrating and explaining to customers the establishment's goods and services
- selling goods and services
- accepting payment for goods and services by a variety of payment methods and preparing sales invoices
- assisting with the ongoing management of stock such as product inventories and participating in stocktakes
- stacking and displaying goods for sale, and wrapping and packing goods sold

Occupations in this minor group are classified into the following unit groups:
6211 Sales Assistants (General)
6212 ICT Sales Assistants
6213 Motor Vehicle and Vehicle Parts Salespersons
6214 Pharmacy Sales Assistants
6215 Retail Supervisors
6216 Service Station Attendants
6217 Street Vendors and Related Salespersons
6219 Other Sales Assistants and Salespersons

UNIT GROUP 6211 SALES ASSISTANTS (GENERAL)

SALES ASSISTANTS (GENERAL) sell goods and services, such as food, clothing, hardware, household appliances, office supplies and cosmetics, in retail and wholesale establishments.

Indicative Skill Level:
Most occupations in this unit group have a level of skill commensurate with the qualifications outlined below.

In Australia:
AQF Certificate I, or compulsory secondary education (ANZSCO Skill Level 5)

In New Zealand:
NZ Register Level 1 qualification, or compulsory secondary education (ANZSCO Skill Level 5)

For some occupations a short period of on-the-job training may be required in addition to or instead of the formal qualification. In some instances no formal qualification or on-the-job training may be required.

Tasks Include:
- determining customer requirements and advising on product range, price, delivery, warranties and product use and care
- demonstrating and explaining to customers the establishment's goods and services
- selling food, beverages, clothing, footwear and other personal and household goods and services
- accepting payment for goods and services by a variety of payment methods and preparing sales invoices
- assisting with the ongoing management of stock such as product inventories and participating in stocktakes
- stacking and displaying goods for sale, and wrapping and packing goods sold

Occupation:
621111 Sales Assistant (General)

621111 SALES ASSISTANT (GENERAL)

Alternative Title:
Retail Sales Assistant

Sells goods and services, such as food, clothing, hardware, household appliances, office supplies and cosmetics, in a retail or wholesale establishment.

Skill Level: 5

Specialisations:
Clothing Sales Assistant
Cosmetic Sales Assistant
Fast Food Sales Assistant
Hardware Sales Assistant

UNIT GROUP 6212 ICT SALES ASSISTANTS

ICT SALES ASSISTANTS sell computing and telecommunications related goods and services in retail and wholesale establishments.

Indicative Skill Level:
Most occupations in this unit group have a level of skill commensurate with the qualifications outlined below.

In Australia:
AQF Certificate I, or compulsory secondary education (ANZSCO Skill Level 5)

In New Zealand:
NZ Register Level 1 qualification, or compulsory secondary education (ANZSCO Skill Level 5)

For some occupations a short period of on-the-job training may be required in addition to or instead of the formal qualification. In some instances no formal qualification or on-the-job training may be required.

Tasks Include:
- determining customer requirements and advising on product range, price, delivery, warranties and product use and care
- demonstrating and explaining to customers the establishment's goods and services
- selling computers, computer peripherals, software, mobile telephones and telephone accessories and services such as Internet access and mobile telephone plans
- accepting payment for goods and services by a variety of payment methods and preparing sales invoices
- assisting with the ongoing management of stock such as product inventories and participating in stocktakes
- stacking and displaying goods for sale, and wrapping and packing goods sold

Occupation:
621211 ICT Sales Assistant

621211 ICT SALES ASSISTANT

Sells computing and telecommunications related goods and services in a retail or wholesale establishment.

Skill Level: 5

Specialisation:
Mobile Phone Salesperson

UNIT GROUP 6213 MOTOR VEHICLE AND VEHICLE PARTS SALESPERSONS

MOTOR VEHICLE AND VEHICLE PARTS SALESPERSONS sell motor vehicles, boats, caravans, earthmoving equipment, vehicle accessories and parts in retail and wholesale establishments.

Indicative Skill Level:
Most occupations in this unit group have a level of skill commensurate with the qualifications and experience outlined below.

In Australia:
AQF Certificate II or III (ANZSCO Skill Level 4)

In New Zealand:
NZ Register Level 2 or 3 qualification (ANZSCO Skill Level 4)

At least one year of relevant experience may substitute for the formal qualifications listed above. In some instances relevant experience and/or on-the-job training may be required in addition to the formal qualification.

Tasks Include:

- determining customer requirements and advising on product range, price, delivery, warranties and product use and care
- showing vehicles to customers and test driving vehicles with customers
- selling motor vehicles and vehicle products such as parts, tyres, lubricating oils, batteries, car stereos and alarms
- taking sales orders and preparing contracts of sale
- receiving orders for parts
- determining part sizes and details such as vehicle make, model, manufacturer and year
- searching lists of parts to identify part numbers, price and availability

Occupations:
621311 Motor Vehicle or Caravan Salesperson
621312 Motor Vehicle Parts Interpreter (Aus) / Automotive Parts Salesperson (NZ)

621311 MOTOR VEHICLE OR CARAVAN SALESPERSON

Alternative Title:
Motor Vehicle Salesperson

Sells new and used motor cars, motor cycles, trucks, boats, caravans and earthmoving equipment in a retail or wholesale establishment.

Skill Level: 4

Specialisation:
Fleet Salesperson

621312 MOTOR VEHICLE PARTS INTERPRETER (AUS) / AUTOMOTIVE PARTS SALESPERSON (NZ)

Alternative Title:
Automotive Parts Interpreter

Sells motor vehicle accessories and parts in a retail or wholesale establishment.

Skill Level: 4

UNIT GROUP 6214 PHARMACY SALES ASSISTANTS

PHARMACY SALES ASSISTANTS sell pharmaceutical goods, toiletries and related goods in retail pharmacies.

Pharmacy Technicians are excluded from this unit group. Pharmacy Technicians are included in Unit Group 3112 Medical Technicians.

Indicative Skill Level:
Most occupations in this unit group have a level of skill commensurate with the qualifications outlined below.

In Australia:
AQF Certificate I, or compulsory secondary education (ANZSCO Skill Level 5)

In New Zealand:
NZ Register Level 1 qualification, or compulsory secondary education (ANZSCO Skill Level 5)

For some occupations a short period of on-the-job training may be required in addition to or instead of the formal qualification. In some instances no formal qualification or on-the-job training may be required.

Tasks Include:
- accepting prescriptions for filling by Retail Pharmacists
- determining customer requirements and advising customers on the selection, price and usage of non-prescription medicines
- advising customers on the correct application and storage of medicines
- selling goods such as non-prescription drugs, first aid supplies, toiletries and cosmetics
- accepting payment for goods and services by a variety of payment methods and preparing sales invoices
- promoting goods and services that are for sale
- assisting with the ongoing management of stock such as product inventories and participating in stocktakes
- stacking and displaying goods for sale, and wrapping and packing goods sold

Occupation:
621411 Pharmacy Sales Assistant

621411 PHARMACY SALES ASSISTANT

Sells pharmaceutical goods, toiletries and related goods in a retail pharmacy.

Skill Level: 5

UNIT GROUP 6215 RETAIL SUPERVISORS

RETAIL SUPERVISORS supervise and coordinate the activities of retail sales workers.

Indicative Skill Level:
Most occupations in this unit group have a level of skill commensurate with the qualifications and experience outlined below.

In Australia:
AQF Certificate II or III (ANZSCO Skill Level 4)

In New Zealand:
NZ Register Level 2 or 3 qualification (ANZSCO Skill Level 4)

At least one year of relevant experience may substitute for the formal qualifications listed above. In some instances relevant experience and/or on-the-job training may be required in addition to the formal qualification.

Tasks Include:
- ensuring that customers receive prompt service and quality goods and services
- responding to customers' inquiries and complaints about goods and services
- planning and preparing work schedules and assigning staff to specific duties
- interviewing, hiring, training, evaluating, dismissing and promoting staff, and resolving staff grievances
- instructing staff on how to handle difficult and complicated sales procedures
- examining returned goods and deciding on appropriate action
- taking inventory of goods for sale and ordering new stock
- ensuring that goods and services are correctly priced and displayed
- ensuring safety and security procedures are enforced

Occupation:
621511 Retail Supervisor

621511 RETAIL SUPERVISOR

Alternative Titles:
Checkout Supervisor
Sales Department Supervisor

Supervises and coordinates the activities of retail sales workers.

Skill Level: 4

UNIT GROUP 6216 SERVICE STATION ATTENDANTS

SERVICE STATION ATTENDANTS sell fuel, lubricants and other automotive accessories, and perform minor maintenance on motor vehicles at service stations.

Indicative Skill Level:
Most occupations in this unit group have a level of skill commensurate with the qualifications outlined below.

In Australia:
AQF Certificate I, or compulsory secondary education (ANZSCO Skill Level 5)

In New Zealand:
NZ Register Level 1 qualification, or compulsory secondary education (ANZSCO Skill Level 5)

For some occupations a short period of on-the-job training may be required in addition to or instead of the formal qualification. In some instances no formal qualification or on-the-job training may be required.

Tasks Include:

- filling fuel tanks and containers to level specified by customer
- checking and replenishing air pressure in vehicle tyres, and oil and other vehicle fluid levels
- washing vehicle windscreens and windows
- performing minor repair work to vehicles such as replacing tyres, light bulbs and windscreen wiper blades
- maintaining and operating automatic car wash facilities
- collecting payments from customers for purchases
- cleaning petrol pumps and surrounding driveway, shop and facilities
- undertaking stock control and preparing reports on fuel, oil, accessories and other items sold
- replenishing stock of fast foods, newspapers, magazines and grocery items

Occupation:
621611 Service Station Attendant

621611 SERVICE STATION ATTENDANT

Alternative Title:
Driveway Attendant

Sells fuel, lubricants and other automotive accessories, and performs minor maintenance on motor vehicles at a service station.

Skill Level: 5

UNIT GROUP 6217 STREET VENDORS AND RELATED SALESPERSONS

STREET VENDORS AND RELATED SALESPERSONS sell goods and services on established routes, door-to-door, and at street and market locations.

Indicative Skill Level:
Most occupations in this unit group have a level of skill commensurate with the qualifications outlined below.

In Australia:
AQF Certificate I, or compulsory secondary education (ANZSCO Skill Level 5)

In New Zealand:
NZ Register Level 1 qualification, or compulsory secondary education (ANZSCO Skill Level 5)

For some occupations a short period of on-the-job training may be required in addition to or instead of the formal qualification. In some instances no formal qualification or on-the-job training may be required.

Tasks Include:

- collecting goods and transporting them along established routes, to door-to-door areas, and to street and market locations
- displaying and demonstrating goods, and explaining the qualities of goods to customers
- informing customers of new goods and services
- receiving payments from customers and giving change
- recording transactions on customer receipts and sales records
- wrapping and packaging goods sold
- developing lists of prospective customers and calling on them to obtain new business
- ordering and purchasing goods for sale, and monitoring and maintaining stock levels
- may attract attention by playing music, singing and calling out goods and services for sale

Occupations:
621711 Cash Van Salesperson
621712 Door-to-door Salesperson
621713 Street Vendor

621711 CASH VAN SALESPERSON

Drives a van or light truck on established routes to sell goods and services.

Skill Level: 5

Specialisations:
Ice-cream Van Vendor
Milk Vendor

621712 DOOR-TO-DOOR SALESPERSON

Sells goods or services from door-to-door.

Skill Level: 5

Specialisations:
Door-to-door Fundraising Collector
Party Plan Salesperson

UNIT GROUP 6217 STREET VENDORS AND RELATED SALESPERSONS *continued*

621713 STREET VENDOR

Sells goods or services to customers at a street or market location.

Skill Level: 5

Specialisation:
Market Stall Vendor

UNIT GROUP 6219 OTHER SALES ASSISTANTS AND SALESPERSONS

This unit group covers Sales Assistants and Salespersons not elsewhere classified.

It includes Materials Recyclers and Rental Salespersons.

Indicative Skill Level:
Most occupations in this unit group have a level of skill commensurate with the qualifications outlined below.

In Australia:
AQF Certificate I, or compulsory secondary education (ANZSCO Skill Level 5)

In New Zealand:
NZ Register Level 1 qualification, or compulsory secondary education (ANZSCO Skill Level 5)

For some occupations a short period of on-the-job training may be required in addition to or instead of the formal qualification. In some instances no formal qualification or on-the-job training may be required.

Occupations:
621911 Materials Recycler
621912 Rental Salesperson
621999 Sales Assistants and Salespersons nec

621911 MATERIALS RECYCLER

Alternative Title:
Scrap Materials Buyer

Salvages materials from industrial, commercial and private establishments for resale.

Skill Level: 5

Specialisations:
Automotive Dismantler
Bottle Dealer
Waste Recycler

621912 RENTAL SALESPERSON

Alternative Title:
Rental Clerk

Rents goods and equipment to individuals and businesses.

Skill Level: 5

Specialisations:
Car Rental Sales Assistant
Industrial Hire Sales Assistant
Video Library Assistant

621999 SALES ASSISTANTS AND SALESPERSONS NEC

This occupation group covers Sales Assistants and Salespersons not elsewhere classified.

Skill Level: 5

UNIT GROUP 6219 OTHER SALES ASSISTANTS AND SALESPERSONS *continued*

Occupations in this group include:

- Carpet Measurer
- Lotteries Agent
- Stockyard Salesperson
- Swimming Pool Salesperson

SUB-MAJOR GROUP 63 SALES SUPPORT WORKERS

SALES SUPPORT WORKERS provide assistance to retailers, wholesalers and sales staff by undertaking support activities such as operating cash registers, and modelling, demonstrating, selecting, buying, promoting and displaying goods.

Indicative Skill Level:
Most occupations in this sub-major group have a level of skill commensurate with the qualifications and experience outlined below.

In Australia:

AQF Certificate III including at least two years of on-the-job training, or AQF Certificate IV, or at least three years of relevant experience (ANZSCO Skill Level 3); or
AQF Certificate II or III, or at least one year of relevant experience (ANZSCO Skill Level 4); or
AQF Certificate I, or compulsory secondary education (ANZSCO Skill Level 5)

In New Zealand:

NZ Register Level 4 qualification, or at least three years of relevant experience (ANZSCO Skill Level 3); or
NZ Register Level 2 or 3 qualification, or at least one year of relevant experience (ANZSCO Skill Level 4); or
NZ Register Level 1 qualification, or compulsory secondary education (ANZSCO Skill Level 5)

In some instances relevant experience and/or on-the-job training may be required in addition to the formal qualification. In the case of some Skill Level 5 occupations, a short period of on-the-job training may be required in addition to or instead of the formal qualification, or no formal qualification or on-the-job training may be required.

Tasks Include:

- receiving payments from customers for goods and services purchased by a variety of payment methods
- counting and recording money received and balancing against register sales records, and preparing money for deposit in financial institutions
- promoting the organisation's goods and services by telephoning customers, and demonstrating goods to customers
- modelling merchandise and posing for art
- buying goods for resale and negotiating purchase, promotion and supply arrangements with suppliers
- setting up displays of products within stores and shopping centres

Occupations in this sub-major group are classified into the following minor groups:

631 Checkout Operators and Office Cashiers
639 Miscellaneous Sales Support Workers

MINOR GROUP 631 CHECKOUT OPERATORS AND OFFICE CASHIERS

CHECKOUT OPERATORS AND OFFICE CASHIERS operate cash registers and receive payments from customers, and issue receipts and return change due.

Indicative Skill Level:
Most occupations in this minor group have a level of skill commensurate with the qualifications outlined below.

In Australia:
AQF Certificate I, or compulsory secondary education (ANZSCO Skill Level 5)

In New Zealand:
NZ Register Level 1 qualification, or compulsory secondary education (ANZSCO Skill Level 5)

For some occupations a short period of on-the-job training may be required in addition to or instead of the formal qualification. In some instances no formal qualification or on-the-job training may be required.

Tasks Include:
- scanning, weighing and recording prices of goods
- receiving and processing payments for goods and services by cash, cheques, gift vouchers, credit and debit cards and other payment types
- issuing sales dockets and giving change
- maintaining supplies of change, wrapping and other materials used at checkout
- counting and recording money received and balancing against register sales records, and preparing money for deposit in financial institutions
- recording and balancing petty cash disbursements
- operating a computer terminal to administer the store financial transaction system
- cashing authorised cheques

The occupations in this minor group are classified into the following unit group:
6311 Checkout Operators and Office Cashiers

UNIT GROUP 6311 CHECKOUT OPERATORS AND OFFICE CASHIERS

CHECKOUT OPERATORS AND OFFICE CASHIERS operate cash registers and receive payments from customers, and issue receipts and return change due.

Indicative Skill Level:
Most occupations in this unit group have a level of skill commensurate with the qualifications outlined below.

In Australia:
AQF Certificate I, or compulsory secondary education (ANZSCO Skill Level 5)

In New Zealand:
NZ Register Level 1 qualification, or compulsory secondary education (ANZSCO Skill Level 5)

For some occupations a short period of on-the-job training may be required in addition to or instead of the formal qualification. In some instances no formal qualification or on-the-job training may be required.

Tasks Include:
- scanning, weighing and recording prices of goods
- receiving and processing payments for goods and services by cash, cheques, gift vouchers, credit and debit cards and other payment types
- issuing sales dockets and giving change
- maintaining supplies of change, wrapping and other materials used at checkout
- counting and recording money received and balancing against register sales records, and preparing money for deposit in financial institutions
- recording and balancing petty cash disbursements
- operating a computer terminal to administer the store's financial transaction system
- cashing authorised cheques

Occupations:
631111 Checkout Operator
631112 Office Cashier

631111 CHECKOUT OPERATOR

Operates cash registers and receives payments for goods purchased by customers.

Skill Level: 5

Specialisation:
Service Station Console Operator

631112 OFFICE CASHIER

Alternative Title:
Cashier

Receives payments from customers, issues receipts, returns change due, and meets the public and explains charging and billing policy.

Skill Level: 5

MINOR GROUP 639 MISCELLANEOUS SALES SUPPORT WORKERS

This minor group covers Sales Support Workers not elsewhere classified.

It includes Models and Sales Demonstrators, Retail and Wool Buyers, Telemarketers, Ticket Salespersons and Visual Merchandisers.

Indicative Skill Level:
Most occupations in this minor group have a level of skill commensurate with the qualifications and experience outlined below.

In Australia:

AQF Certificate III including at least two years of on-the-job training, or AQF Certificate IV, or at least three years of relevant experience (ANZSCO Skill Level 3); or
AQF Certificate II or III, or at least one year of relevant experience (ANZSCO Skill Level 4); or
AQF Certificate I, or compulsory secondary education (ANZSCO Skill Level 5)

In New Zealand:

NZ Register Level 4 qualification, or at least three years of relevant experience (ANZSCO Skill Level 3); or
NZ Register Level 2 or 3 qualification, or at least one year of relevant experience (ANZSCO Skill Level 4); or
NZ Register Level 1 qualification, or compulsory secondary education (ANZSCO Skill Level 5)

In some instances relevant experience and/or on-the-job training may be required in addition to the formal qualification. In the case of some Skill Level 5 occupations, a short period of on-the-job training may be required in addition to or instead of the formal qualification, or no formal qualification or on-the-job training may be required.

Occupations in this minor group are classified into the following unit groups:

6391 Models and Sales Demonstrators
6392 Retail and Wool Buyers
6393 Telemarketers
6394 Ticket Salespersons
6395 Visual Merchandisers
6399 Other Sales Support Workers

UNIT GROUP 6391 MODELS AND SALES DEMONSTRATORS

MODELS AND SALES DEMONSTRATORS wear and display clothing and accessories and pose for art and photography, and demonstrate goods at commercial premises, exhibitions and private homes.

Indicative Skill Level:
Most occupations in this unit group have a level of skill commensurate with the qualifications outlined below.

In Australia:
AQF Certificate I, or compulsory secondary education (ANZSCO Skill Level 5)

In New Zealand:
NZ Register Level 1 qualification, or compulsory secondary education (ANZSCO Skill Level 5)

For some occupations a short period of on-the-job training may be required in addition to or instead of the formal qualification. In some instances no formal qualification or on-the-job training may be required.

Tasks Include:
- modelling garments, footwear and fashion accessories for customers, sales personnel and fashion designers
- posing for television, video and cinema commercials and for still photographs which appear in magazines, newspapers, catalogues and on billboards
- posing as subjects for paintings, sculptures and other types of art
- setting up displays and demonstrating goods to commercial customers and guests in private homes
- answering questions and offering advice on the use of goods
- selling goods or directing purchasers to sales counters
- undertaking merchandising of goods in retail outlets and ensuring there is adequate stock attractively presented for sale
- taking orders and making arrangements for payment, delivery and collection
- offering sample goods and distributing catalogues and other literature advertising goods for sale

Occupations:
639111 Model
639112 Sales Demonstrator

639111 MODEL

Wears and displays clothing and accessories, and poses for photographs, paintings, sculptures and other types of art.

Skill Level: 5

639112 SALES DEMONSTRATOR

Alternative Title:
Merchandiser

Displays and demonstrates goods at commercial premises, exhibitions and private homes.

Skill Level: 5

UNIT GROUP 6392 RETAIL AND WOOL BUYERS

RETAIL AND WOOL BUYERS select and buy goods for resale in retail establishments, and value and buy wool sold by wool growers.

Indicative Skill Level:
Most occupations in this unit group have a level of skill commensurate with the qualifications and experience outlined below.

In Australia:
AQF Certificate III including at least two years of on-the-job training, or AQF Certificate IV (ANZSCO Skill Level 3)

In New Zealand:
NZ Register Level 4 qualification (ANZSCO Skill Level 3)

At least three years of relevant experience may substitute for the formal qualifications listed above. In some instances relevant experience and/or on-the-job training may be required in addition to the formal qualification.

Tasks Include:
- monitoring sales data and stock levels, and studying trade, manufacturers' and market information to keep informed of changing market conditions
- negotiating purchase, promotion and supply arrangements with suppliers
- designing and implementing pricing, marketing, promotional and display strategies
- liaising with management on long-term planning and sales promotions
- establishing working plans according to seasonal and budgetary requirements
- anticipating consumer trends and determining quantity, style and quality of goods to be purchased
- inspecting, comparing, selecting and valuing wool by determining colour, yield, micron and length
- inspecting and buying wool at auction, in wool brokers' stores and in farm sheds
- receiving samples from scoured wool exchanges
- may visit freezing works to buy slipe wool

Occupations:
639211 Retail Buyer
639212 Wool Buyer

639211 RETAIL BUYER

Selects and buys goods for resale in a retail establishment.

Skill Level: 3

Specialisation:
Merchandise Planner

639212 WOOL BUYER

Values and buys wool sold by wool growers.

Skill Level: 3

UNIT GROUP 6393 TELEMARKETERS

TELEMARKETERS telephone existing and prospective customers to promote goods and services, and obtain sales and arrange sales visits.

Indicative Skill Level:
Most occupations in this unit group have a level of skill commensurate with the qualifications outlined below.

In Australia:
AQF Certificate I, or compulsory secondary education (ANZSCO Skill Level 5)

In New Zealand:
NZ Register Level 1 qualification, or compulsory secondary education (ANZSCO Skill Level 5)

For some occupations a short period of on-the-job training may be required in addition to or instead of the formal qualification. In some instances no formal qualification or on-the-job training may be required.

Tasks Include:
- working from scripts and lists of contacts to promote goods and services by telephone
- creating interest in goods and services, and seeking a sale or agreement to see sales representatives
- arranging processing and despatch of goods and services, information kits and brochures to customers
- arranging appointments for sales representatives
- recording notes for follow-up action and updating marketing databases to reflect changes to the status of each customer
- reporting competitor activities and issues raised by contacts for attention by managers
- maintaining statistics of calls made and successes achieved
- submitting periodic reports on telemarketing activities and results
- may work in a call centre

Occupation:
639311 Telemarketer

639311 TELEMARKETER

Telephones existing and prospective customers to promote goods and services, and obtain sales or arrange sales visits. May work in a call centre.

Skill Level: 5

UNIT GROUP 6394 TICKET SALESPERSONS

TICKET SALESPERSONS sell tickets and make reservations for services such as travel and admission to sporting and entertainment venues, and collect fares on transport vehicles.

Indicative Skill Level:
Most occupations in this unit group have a level of skill commensurate with the qualifications outlined below.

In Australia:
AQF Certificate I, or compulsory secondary education (ANZSCO Skill Level 5)

In New Zealand:
NZ Register Level 1 qualification, or compulsory secondary education (ANZSCO Skill Level 5)

For some occupations a short period of on-the-job training may be required in addition to or instead of the formal qualification. In some instances no formal qualification or on-the-job training may be required.

Tasks Include:
- receiving customers' requests, accepting payments, collecting fares from passengers, and issuing tickets, receipts and change
- answering inquiries about charges, routes, schedules, reservations, coming attractions and fares
- checking service availability and times, and making reservations
- contacting customers to cancel or confirm reservations
- organising displays of service availability, times and other information
- collecting tickets and change from depot clerks
- signalling drivers to stop and proceed
- overseeing passengers' safety in emergency circumstances, and opening and closing vehicle doors
- assisting passengers to board and alight from vehicles and assisting passengers with baggage

Occupations:
639411 Ticket Seller
639412 Transport Conductor

639411 TICKET SELLER

Sells tickets and makes reservations for services such as travel and admission to sporting and entertainment venues. May work in a call centre.

Skill Level: 5

Specialisations:
Booking Clerk
Reservations Clerk

639412 TRANSPORT CONDUCTOR

Collects fares and issues tickets on a transport vehicle.

Skill Level: 5

UNIT GROUP 6395 VISUAL MERCHANDISERS

VISUAL MERCHANDISERS plan and install internal, window and fixed displays to show goods to their best advantage.

Indicative Skill Level:
Most occupations in this unit group have a level of skill commensurate with the qualifications and experience outlined below.

In Australia:
AQF Certificate II or III (ANZSCO Skill Level 4)

In New Zealand:
NZ Register Level 2 or 3 qualification (ANZSCO Skill Level 4)

At least one year of relevant experience may substitute for the formal qualifications listed above. In some instances relevant experience and/or on-the-job training may be required in addition to the formal qualification.

Tasks Include:
- determining goods for display in accordance with prospective seasonal and promotional events
- developing overall promotional and display plans for approval
- preparing sketches and models showing layout, colour and other features for approval
- obtaining props and other accessories, and building displays
- setting up fabricated displays in store windows and other areas
- organising the setting out of goods to be shown as part of permanent displays
- arranging ticketing and signage
- arranging lighting to highlight fixtures, displays and goods

Occupation:
639511 Visual Merchandiser

639511 VISUAL MERCHANDISER

Alternative Title:
Window Dresser

Plans and installs internal, window and fixed displays to show goods to their best advantage.

Skill Level: 4

UNIT GROUP 6399 OTHER SALES SUPPORT WORKERS

This unit group covers Sales Support Workers not elsewhere classified.

It includes Other Sales Support Workers.

Indicative Skill Level:
Most occupations in this unit group have a level of skill commensurate with the qualifications outlined below.

In Australia:
AQF Certificate I, or compulsory secondary education (ANZSCO Skill Level 5)

In New Zealand:
NZ Register Level 1 qualification, or compulsory secondary education (ANZSCO Skill Level 5)

For some occupations a short period of on-the-job training may be required in addition to or instead of the formal qualification. In some instances no formal qualification or on-the-job training may be required.

Occupation:
639911 Other Sales Support Worker

639911 OTHER SALES SUPPORT WORKER

This occupation group covers Sales Support Workers not elsewhere classified.

Skill Level: 5

Specialisations:
Mystery Shopper
Personal Shopper

MAJOR GROUP 7 MACHINERY OPERATORS AND DRIVERS

MACHINERY OPERATORS AND DRIVERS operate machines, plant, vehicles and other equipment to perform a range of agricultural, manufacturing and construction functions, move materials, and transport passengers and freight.

Indicative Skill Level:
Most occupations in this major group have a level of skill commensurate with the qualifications and experience outlined below.

In Australia:
AQF Certificate II or III (ANZSCO Skill Level 4)

In New Zealand:
NZ Register Level 2 or 3 qualification (ANZSCO Skill Level 4)

At least one year of relevant experience may substitute for the formal qualifications listed above. In some instances relevant experience and/or on-the-job training may be required in addition to the formal qualification.

Tasks Include:
- setting up, controlling and monitoring the operation of machines, plant and equipment
- cleaning machines, plant and equipment and performing minor repairs
- transporting passengers and freight to set destinations
- receiving, loading, unloading and despatching goods

Occupations in this major group are classified into the following sub-major groups:
71 Machine and Stationary Plant Operators
72 Mobile Plant Operators
73 Road and Rail Drivers
74 Storepersons

SUB-MAJOR GROUP 71 MACHINE AND STATIONARY PLANT OPERATORS

MACHINE AND STATIONARY PLANT OPERATORS operate stationary machines to process, manufacture, treat and finish a range of products, and perform activities such as extracting, loading, unloading, moving, placing, controlling, storing and measuring materials and equipment.

Indicative Skill Level:
Most occupations in this sub-major group have a level of skill commensurate with the qualifications and experience outlined below.

In Australia:
AQF Certificate II or III (ANZSCO Skill Level 4)

In New Zealand:
NZ Register Level 2 or 3 qualification (ANZSCO Skill Level 4)

At least one year of relevant experience may substitute for the formal qualifications listed above. In some instances relevant experience and/or on-the-job training may be required in addition to the formal qualification.

Tasks Include:

- setting up, starting, controlling and stopping machines and plant
- monitoring machines and plant and adjusting controls to regulate operation and quality of output
- cleaning and performing minor repairs to machines and plant
- checking machines and plant to ensure safe operations

Occupations in this sub-major group are classified into the following minor groups:

711 Machine Operators
712 Stationary Plant Operators

MINOR GROUP 711 MACHINE OPERATORS

MACHINE OPERATORS operate stationary machines to process, manufacture, treat and finish a range of products.

Indicative Skill Level:
Most occupations in this minor group have a level of skill commensurate with the qualifications and experience outlined below.

In Australia:
AQF Certificate II or III (ANZSCO Skill Level 4)

In New Zealand:
NZ Register Level 2 or 3 qualification (ANZSCO Skill Level 4)

At least one year of relevant experience may substitute for the formal qualifications listed above. In some instances relevant experience and/or on-the-job training may be required in addition to the formal qualification.

Tasks Include:
- fixing attachments to machines, setting controls and loading material to be processed
- starting machines, observing operation and adjusting controls to regulate temperature, pressure, intake of raw materials and speed
- sampling output for defects and variations, and adjusting machine settings accordingly
- unloading and storing output
- cleaning and lubricating machines and performing minor repairs

Occupations in this minor group are classified into the following unit groups:
7111 Clay, Concrete, Glass and Stone Processing Machine Operators
7112 Industrial Spraypainters
7113 Paper and Wood Processing Machine Operators
7114 Photographic Developers and Printers
7115 Plastics and Rubber Production Machine Operators
7116 Sewing Machinists
7117 Textile and Footwear Production Machine Operators
7119 Other Machine Operators

UNIT GROUP 7111 CLAY, CONCRETE, GLASS AND STONE PROCESSING MACHINE OPERATORS

CLAY, CONCRETE, GLASS AND STONE PROCESSING MACHINE OPERATORS operate machines to manufacture and finish a variety of clay, concrete, glassware and stone products by extruding, shaping, mixing, grinding, cutting and other processes.

Indicative Skill Level:
Most occupations in this unit group have a level of skill commensurate with the qualifications and experience outlined below.

In Australia:
AQF Certificate II or III (ANZSCO Skill Level 4)

In New Zealand:
NZ Register Level 2 or 3 qualification (ANZSCO Skill Level 4)

At least one year of relevant experience may substitute for the formal qualifications listed above. In some instances relevant experience and/or on-the-job training may be required in addition to the formal qualification.

Tasks Include:
- monitoring the flow of clay and other raw materials and products into machines, and adjusting valves and controls to specifications
- positioning clay and stone on machines to be cut and worked
- operating concrete mixing, stacking and splitting machines
- setting up and installing moulds and other machine fixtures
- setting up and operating glass-making machines to produce molten glass, and regulating temperature of molten glass
- pressing and blowing glass into moulds to form glassware products
- collecting and examining samples for conformity to specifications and adjusting machine settings accordingly
- setting grinding and cutting edges
- using hand tools to cut, inscribe and polish roughly hewn stone to finished condition

Occupations:
711111 Clay Products Machine Operator
711112 Concrete Products Machine Operator
711113 Glass Production Machine Operator
711114 Stone Processing Machine Operator
711199 Clay, Concrete, Glass and Stone Processing Machine Operators nec

711111 CLAY PRODUCTS MACHINE OPERATOR

Operates machines to manufacture clay products, such as bricks, tiles, insulators, porcelain and pottery, by shaping and firing clay.

Skill Level: 4

Specialisations:
Brick Extruder Operator
Porcelain Turner
Slip Caster

UNIT GROUP 7111 CLAY, CONCRETE, GLASS AND STONE PROCESSING MACHINE OPERATORS *continued*

711112 CONCRETE PRODUCTS MACHINE OPERATOR

Operates machines to manufacture moulded concrete products such as cement pipes and fittings, concrete railway sleepers, concrete bricks, tiles and paving blocks, structural beams, building panels and cast products.

Skill Level: 4

Specialisations:

- Concrete Pipe Machine Operator
- Concrete Precast Moulder
- Concrete Tile Machine Operator

711113 GLASS PRODUCTION MACHINE OPERATOR

Operates machines to manufacture molten glass and shape glassware products such as containers, sheet glass, structural and stained glass, glass lenses and prisms.

Skill Level: 4

Specialisations:

- Glass Furnace Operator
- Glass Laminating Operator
- Glass Maker
- Glass Melt Operator
- Glass Toughening Operator
- Glassware Maker

711114 STONE PROCESSING MACHINE OPERATOR

Operates machines to cut and finish stones for tiles, building blocks and facings.

Skill Level: 4

Specialisations:

- Marble Cutter
- Stone Polisher
- Stone Sawyer

711199 CLAY, CONCRETE, GLASS AND STONE PROCESSING MACHINE OPERATORS NEC

This occupation group covers Clay, Concrete, Glass and Stone Processing Machine Operators not elsewhere classified.

Skill Level: 4

Occupations in this group include:

- Abrasive Wheel Maker
- Brake Lining Maker
- Fibre Cement Moulder
- Plaster Caster
- Plaster Machine Operator

UNIT GROUP 7112 INDUSTRIAL SPRAYPAINTERS

INDUSTRIAL SPRAYPAINTERS operate spray painting equipment to paint and apply other industrial coatings to manufactured items.

Vehicle Painters are excluded from this unit group. Vehicle Painters are included in Unit Group 3243 Vehicle Painters.

Indicative Skill Level:
Most occupations in this unit group have a level of skill commensurate with the qualifications and experience outlined below.

In Australia:
AQF Certificate II or III (ANZSCO Skill Level 4)

In New Zealand:
NZ Register Level 2 or 3 qualification (ANZSCO Skill Level 4)

At least one year of relevant experience may substitute for the formal qualifications listed above. In some instances relevant experience and/or on-the-job training may be required in addition to the formal qualification.

Tasks Include:
- grinding, sanding and cleaning surfaces of items to be painted
- loading paint, oil, lacquer, varnish and rustproofing agents into spray equipment
- connecting hoses to spray equipment and adjusting spray nozzles to required pressure
- securing items to be sprayed within spray booths or placing them onto conveyors
- directing spray guns to apply even coatings
- moving items to drying areas and stacking them for further painting and packaging
- starting and monitoring extractor and drying fans, and heaters
- cleaning nozzles, containers and hoses of equipment
- may operate paint dipping baths
- may mix coating solutions and regulate their temperature

Occupation:
711211 Industrial Spraypainter

711211 INDUSTRIAL SPRAYPAINTER

Operates spray painting equipment to paint and apply other industrial coatings to manufactured items.

Skill Level: 4

Specialisations:
Powder Coater
Rust Proofer

UNIT GROUP 7113 PAPER AND WOOD PROCESSING MACHINE OPERATORS

PAPER AND WOOD PROCESSING MACHINE OPERATORS operate machines to manufacture paper packaging and other paper products, fibreboard stock, logs, plywood, particle board, solid laminate and similar timber products.

Indicative Skill Level:
Most occupations in this unit group have a level of skill commensurate with the qualifications and experience outlined below.

In Australia:
AQF Certificate II or III (ANZSCO Skill Level 4)

In New Zealand:
NZ Register Level 2 or 3 qualification (ANZSCO Skill Level 4)

At least one year of relevant experience may substitute for the formal qualifications listed above. In some instances relevant experience and/or on-the-job training may be required in addition to the formal qualification.

Tasks Include:
- setting up printing plates, ink circulation systems, knives, creases, cutting dies, and folding and gluing machines
- loading machines with paper and fibreboard
- operating machines to form cardboard containers, paper plates, egg cartons, tissue paper and other paper products
- adjusting and cleaning machines and performing minor repairs
- securing timber into place and setting saws to produce specified sizes of plank and board to be cut
- starting machines and feeding stock onto cutting saw, and operating automatic feed mechanisms
- raising and lowering saws to trim boards and remove defects such as rot and splits
- controlling lathes and slicing machines to produce veneers, and laminating veneer using glue
- verifying dimensions of cut stock and accuracy of cuts
- checking saws and other machines for safety, sharpness and correct functioning

Occupations:
711311 Paper Products Machine Operator
711312 Wood Processing Machine Operator

711311 PAPER PRODUCTS MACHINE OPERATOR

Operates machines to manufacture paper packaging and other products from paper and fibreboard stock.

Skill Level: 4

Specialisations:
Carton Making Machinist
Embosser
Paper Bag Making Machinist

UNIT GROUP 7113 PAPER AND WOOD PROCESSING MACHINE OPERATORS *continued*

711312 WOOD PROCESSING MACHINE OPERATOR

Operates sawing, rolling, pressing and other machines to manufacture logs, timber poles and pieces, plywood, particle board, solid laminate and similar products.

Skill Level: 4

Specialisations:

- Band Saw Operator
- Beam Saw Operator
- Cant Gang Sawyer
- Debarker Operator
- Docking Saw Operator
- Log Preparer
- Plywood and Veneer Repairer
- Resawyer
- Ripsaw Operator
- Sawmill Moulder Operator
- Veneer Production Machine Operator

UNIT GROUP 7114 PHOTOGRAPHIC DEVELOPERS AND PRINTERS

PHOTOGRAPHIC DEVELOPERS AND PRINTERS edit and adjust digital images, develop photographic film, and print photographic images from digital media, negatives and positives using computer software, fully automatic equipment and by separate processes.

Indicative Skill Level:
Most occupations in this unit group have a level of skill commensurate with the qualifications and experience outlined below.

In Australia:
AQF Certificate II or III (ANZSCO Skill Level 4)

In New Zealand:
NZ Register Level 2 or 3 qualification (ANZSCO Skill Level 4)

At least one year of relevant experience may substitute for the formal qualifications listed above. In some instances relevant experience and/or on-the-job training may be required in addition to the formal qualification.

Tasks Include:
- cropping images and adjusting colour, brightness and contrast
- preparing exposed film for different processing batches in dark rooms and dark chambers
- adjusting settings and running automatic developing equipment
- inspecting prints and adjusting settings on print-making equipment to produce required number, size and type of prints
- scanning images onto electronic media
- checking and replenishing chemicals and water supply for chemical and water baths required to produce negative and positive prints
- monitoring and testing photographic processing and printing equipment, and maintaining operational standards
- may prepare chemical solutions for different techniques and effects
- may develop black and white images by separate processes and operate enlargers
- may develop motion picture film

Occupation:
711411 Photographic Developer and Printer

711411 PHOTOGRAPHIC DEVELOPER AND PRINTER

Edits and adjusts digital images, develops photographic film, and prints photographic images from digital media, negatives and positives using computer software, fully automatic equipment and by separate processes.

Skill Level: 4

Specialisations:
Copy Camera Operator
Dark Room Attendant
Digital Photographic Printer
Film Process Operator
Minilab Operator
Photographic Enlarger Operator
Silver Recovery Operator (Aus)
Slide Developer

UNIT GROUP 7115 PLASTICS AND RUBBER PRODUCTION MACHINE OPERATORS

PLASTICS AND RUBBER PRODUCTION MACHINE OPERATORS operate machines to manufacture and finish plastic and rubber products.

Indicative Skill Level:
Most occupations in this unit group have a level of skill commensurate with the qualifications and experience outlined below.

In Australia:
AQF Certificate II or III (ANZSCO Skill Level 4)

In New Zealand:
NZ Register Level 2 or 3 qualification (ANZSCO Skill Level 4)

At least one year of relevant experience may substitute for the formal qualifications listed above. In some instances relevant experience and/or on-the-job training may be required in addition to the formal qualification.

Tasks Include:
- operating controls to regulate temperature, pressure, speed and flow of operation
- measuring and loading materials, items and ingredients for mixing into machines and feeding mechanisms
- monitoring operation, regulating material supply and adding chemicals and colorants to mixture
- threading uncoated wire and cable through plastic coating machines, around take-up reels and through dies and cooling chambers
- laying casings, beads, ply and rubber sheets on moulds
- operating rollers to remove air
- operating vulcaniser presses and controlling curing
- examining output for defects and conformity to specifications
- performing minor repairs and maintaining production records

Occupations:
711511 Plastic Cablemaking Machine Operator
711512 Plastic Compounding and Reclamation Machine Operator
711513 Plastics Fabricator or Welder
711514 Plastics Production Machine Operator (General)
711515 Reinforced Plastic and Composite Production Worker
711516 Rubber Production Machine Operator
711599 Plastics and Rubber Production Machine Operators nec

711511 PLASTIC CABLEMAKING MACHINE OPERATOR

Operates extruding machines to encase wire, cord, cable and optic fibre in plastic or rubber.

Skill Level: 4

Specialisations:
Insulation Extruder Operator
Optic Fibre Drawer
Wire Drawer (Plastics)

UNIT GROUP 7115 PLASTICS AND RUBBER PRODUCTION MACHINE OPERATORS *continued*

711512 PLASTIC COMPOUNDING AND RECLAMATION MACHINE OPERATOR

Operates mixing and grinding machines to prepare plastic powders and liquid blends, and recycle waste plastic materials from factory operations.

Skill Level: 4

Specialisations:
- Pelletising Extruder Operator
- Powder Hand (Plastics)
- Shredder/Granulator Operator

711513 PLASTICS FABRICATOR OR WELDER

Operates machines to measure, cut, shape, fit and assemble plastics materials to produce plastic products.

Skill Level: 4

Specialisations:
- Acrylic Fabricator
- Vinyl Welder and Fabricator

711514 PLASTICS PRODUCTION MACHINE OPERATOR (GENERAL)

Operates extruding, injection moulding and blow moulding machines to produce finished plastic products.

Skill Level: 4

Specialisations:
- Blow Moulding Machine Operator
- Extruding Machine Operator (Plastics)
- Injection Moulding Machine Operator (Plastics)
- Lamination Machine Operator
- Plastic Production Machine Setter

711515 REINFORCED PLASTIC AND COMPOSITE PRODUCTION WORKER

Operates machines to apply gelcoat, colouring and fibre reinforced plastic to moulds to produce fibreglass and laminated products.

Skill Level: 4

Specialisations:
- Fibreglass Gun Hand
- Fibreglass Hand Laminator
- Resin Transfer Moulding Machine Operator

UNIT GROUP 7115 PLASTICS AND RUBBER PRODUCTION MACHINE OPERATORS *continued*

711516 RUBBER PRODUCTION MACHINE OPERATOR

Operates machines to manufacture rubber products such as tyres.

Skill Level: 4

Specialisations:

- Rubber Belt Splicer
- Rubber Compounder
- Rubber Extrusion Machine Operator
- Rubber Knitting and Reinforcing Machine Operator
- Rubber Moulding Machine Operator
- Rubber Roller Grinder Operator
- Tyre Builder
- Tyre Retreader

711599 PLASTICS AND RUBBER PRODUCTION MACHINE OPERATORS NEC

This occupation group covers Plastics and Rubber Production Machine Operators not elsewhere classified.

Skill Level: 4

Occupations in this group include:

- Rotational Moulding Operator (Plastics)
- Thermoforming Machine Operator

UNIT GROUP 7116 SEWING MACHINISTS

SEWING MACHINISTS operate industrial sewing machines to sew and finish garments and soft furnishings such as curtains.

Indicative Skill Level:
Most occupations in this unit group have a level of skill commensurate with the qualifications and experience outlined below.

In Australia:
AQF Certificate II or III (ANZSCO Skill Level 4)

In New Zealand:
NZ Register Level 2 or 3 qualification (ANZSCO Skill Level 4)

At least one year of relevant experience may substitute for the formal qualifications listed above. In some instances relevant experience and/or on-the-job training may be required in addition to the formal qualification.

Tasks Include:
- threading machines, inserting bobbins and positioning parts to be sewn
- starting, stopping and controlling speed of machines with pedals and knee levers to coordinate actions of presser feet, clothes guides, blades and other attachments, and guiding parts under needles, following edges, seams and markings
- changing needles and adjusting, securing and modifying attachments to machines
- finishing items by cutting excess material and threads
- operating thread trimming and other non-sewing machines
- inspecting stitching for defects and notifying repair mechanics of machine malfunctions
- performing basic maintenance such as lubrication of machines
- may do laying up and bundling tasks
- may ticket, label and finish work

Occupation:
711611 Sewing Machinist

711611 SEWING MACHINIST

Operates industrial sewing machines to sew and finish garments and soft furnishings such as curtains.

Skill Level: 4

Specialisation:
Embroiderer

UNIT GROUP 7117 TEXTILE AND FOOTWEAR PRODUCTION MACHINE OPERATORS

TEXTILE AND FOOTWEAR PRODUCTION MACHINE OPERATORS operate machines to process raw hides and skins, raw textile fibres, and dye, weave and knit fibres for use in textile and footwear production.

Indicative Skill Level:
Most occupations in this unit group have a level of skill commensurate with the qualifications and experience outlined below.

In Australia:
AQF Certificate II or III (ANZSCO Skill Level 4)

In New Zealand:
NZ Register Level 2 or 3 qualification (ANZSCO Skill Level 4)

At least one year of relevant experience may substitute for the formal qualifications listed above. In some instances relevant experience and/or on-the-job training may be required in addition to the formal qualification.

Tasks Include:
- preparing machines for operation by selecting and installing attachments and components for specialised functions
- setting and operating controls used to regulate processing operations
- starting machines and monitoring operation to detect faults and ensure effectiveness of operation
- loading drums with hides and skins, textiles, and dyeing and tanning solutions
- cutting and machining leather and synthetic shoe uppers, and making shoes using moulded and cement construction techniques
- threading loom shuttles with cross-yarn arms
- positioning and feeding machines with fibre packages
- repairing broken yarns by tying and splicing ends
- examining finished products for defects and variations, reporting faults in machines, and carrying out quality control procedures

Occupations:
711711 Footwear Production Machine Operator
711712 Hide and Skin Processing Machine Operator
711713 Knitting Machine Operator
711714 Textile Dyeing and Finishing Machine Operator
711715 Weaving Machine Operator
711716 Yarn Carding and Spinning Machine Operator
711799 Textile and Footwear Production Machine Operators nec

711711 FOOTWEAR PRODUCTION MACHINE OPERATOR

Operates machines to manufacture ready-to-wear footwear.

Skill Level: 4

Specialisations:
Shoemaking Cutter
Shoemaking Finisher

UNIT GROUP 7117 TEXTILE AND FOOTWEAR PRODUCTION MACHINE OPERATORS *continued*

711712 HIDE AND SKIN PROCESSING MACHINE OPERATOR

Alternative Title:

Leather Production Machine Operator

Operates machines to convert raw hides and skins into finished leather for use in clothing, footwear and upholstery.

Skill Level: 4

Specialisations:

Fellmongering Machine Operator
Hide and Skin Fleshing Machine Operator
Sammying Machine Operator
Tanner

711713 KNITTING MACHINE OPERATOR

Alternative Title:

Textile Knitter

Operates machines to knit fabrics, garment parts and other articles from yarns such as cotton, wool, nylon and rayon.

Skill Level: 4

Specialisations:

Flat Bed Knitter
Warp Knitter

711714 TEXTILE DYEING AND FINISHING MACHINE OPERATOR

Operates machines to bleach, dye and finish knitted garments such as hosiery and woollen garments.

Skill Level: 4

Specialisations:

Textile Dyer
Textile Finisher

711715 WEAVING MACHINE OPERATOR

Alternative Title:

Loom Operator

Operates looms to weave yarn into cloth, carpet and other fabrics.

Skill Level: 4

Specialisations:

Beamer
Carpet Weaver
Warper

UNIT GROUP 7117 TEXTILE AND FOOTWEAR PRODUCTION MACHINE OPERATORS *continued*

711716 YARN CARDING AND SPINNING MACHINE OPERATOR

Operates machines to convert raw textile fibres into continuous untwisted and twisted strands of yarn for use in clothing, carpets, curtains and other fabrics.

Skill Level: 4

Specialisations:
- Cotton Ginner
- Gill Box Operator
- Yarn Comber
- Yarn Texture Machine Operator

711799 TEXTILE AND FOOTWEAR PRODUCTION MACHINE OPERATORS NEC

This occupation group covers Textile and Footwear Production Machine Operators not elsewhere classified.

Skill Level: 4

Occupations in this group include:
- Cord Making Machine Operator
- Feltmaker
- Net Maker
- Rope Making Machine Operator
- Tufting Machine Operator

UNIT GROUP 7119 OTHER MACHINE OPERATORS

This unit group covers Machine Operators not elsewhere classified.

It includes Chemical Production Machine Operators, Motion Picture Projectionists, Sand Blasters and Sterilisation Technicians.

Indicative Skill Level:
Most occupations in this unit group have a level of skill commensurate with the qualifications and experience outlined below.

In Australia:
AQF Certificate II or III (ANZSCO Skill Level 4)

In New Zealand:
NZ Register Level 2 or 3 qualification (ANZSCO Skill Level 4)

At least one year of relevant experience may substitute for the formal qualifications listed above. In some instances relevant experience and/or on-the-job training may be required in addition to the formal qualification.

Occupations:
711911 Chemical Production Machine Operator
711912 Motion Picture Projectionist
711913 Sand Blaster
711914 Sterilisation Technician
711999 Machine Operators nec

711911 CHEMICAL PRODUCTION MACHINE OPERATOR

Operates machines to produce chemical goods such as soaps, detergents, pharmaceuticals, toiletries and explosives.

Skill Level: 4

Specialisations:
Bullet Maker
Candle Maker
Cosmetics Machine Operator
Explosives Mixer Operator
Nitrocellulose Maker
Paint Tinter
Tablet Making Machine Operator

711912 MOTION PICTURE PROJECTIONIST

Operates film projection and related sound reproduction equipment.

Skill Level: 4

711913 SAND BLASTER

Operates sandblasting machines to clean and grind metal products and other hard surfaces.

Skill Level: 4

UNIT GROUP 7119 OTHER MACHINE OPERATORS *continued*

711914 STERILISATION TECHNICIAN

Cleans, sterilises and packages surgical instruments and other hospital equipment, soft goods and linen in a sterilisation service facility.

Skill Level: 4

711999 MACHINE OPERATORS NEC

This occupation group covers Machine Operators not elsewhere classified.

Skill Level: 4

Occupations in this group include:

- Asbestos Remover
- Brush Maker
- Film Cutter
- Film Spooler
- Linoleum Maker
- Pressurised Container Filler
- Remotely Operated Vehicle (ROV) Pilot
- Sideshow Ride Operator
- Venetian Blind Machine Operator

MINOR GROUP 712 STATIONARY PLANT OPERATORS

STATIONARY PLANT OPERATORS operate stationary plant to perform a range of activities such as extracting minerals from the earth; refining and treating metals and mineral ore, and producing basic metal products; lifting, moving and placing materials and equipment; and loading and stacking bulk materials.

Indicative Skill Level:
Most occupations in this minor group have a level of skill commensurate with the qualifications and experience outlined below.

In Australia:
AQF Certificate II or III (ANZSCO Skill Level 4)

In New Zealand:
NZ Register Level 2 or 3 qualification (ANZSCO Skill Level 4)

At least one year of relevant experience may substitute for the formal qualifications listed above. In some instances relevant experience and/or on-the-job training may be required in addition to the formal qualification.

Tasks Include:
- fixing attachments to plant and setting controls prior to operation
- manipulating controls to move materials between process phases
- observing operation and adjusting controls to regulate temperature, pressure, mix of materials to be processed and handled, and speed of operation
- monitoring operation of plant directly or by referring to instruments ensuring safe operation
- cleaning and lubricating plant, repairing minor defects and reporting major problems
- unloading and storing output

Occupations in this minor group are classified into the following unit groups:
7121 Crane, Hoist and Lift Operators
7122 Drillers, Miners and Shot Firers
7123 Engineering Production Systems Workers
7129 Other Stationary Plant Operators

UNIT GROUP 7121 CRANE, HOIST AND LIFT OPERATORS

CRANE, HOIST AND LIFT OPERATORS operate stationary and mobile cranes, hoists, lifts and winches to lift, move and place materials, equipment and people in areas such as building sites, factories, mines, sawmills, wharves and shipyards.

Indicative Skill Level:
Most occupations in this unit group have a level of skill commensurate with the qualifications and experience outlined below.

In Australia:
AQF Certificate II or III (ANZSCO Skill Level 4)

In New Zealand:
NZ Register Level 2 or 3 qualification (ANZSCO Skill Level 4)

At least one year of relevant experience may substitute for the formal qualifications listed above. In some instances relevant experience and/or on-the-job training may be required in addition to the formal qualification.

Registration or licensing is required.

Tasks Include:
- testing the operation of plant before use to ensure safety
- operating controls to rotate cranes, move cranes on fixed rails, raise and lower jibs and booms, and raise, lower and move hooks and objects
- working in conjunction with Construction Riggers and Crane Chasers to position hooks and raise, move and place loads
- controlling the movement of loads, and monitoring speed, acceleration and braking distances directly and by signalling to other operators
- monitoring plant operation, instruments and gauges to detect malfunctions and problems
- lubricating ropes and winches on cranes and replacing worn cables
- may operate cranes fitted with attachments for purposes such as demolition and pile driving
- may operate overhead cranes using hand controls suspended by cables from cranes

Occupation:
712111 Crane, Hoist or Lift Operator

712111 CRANE, HOIST OR LIFT OPERATOR

Operates stationary and mobile cranes, hoists, lifts and winches to lift, move and place materials, equipment and people in areas such as building sites, factories, mines, sawmills, wharves and shipyards. Registration or licensing is required.

Skill Level: 4

Specialisations:
Braceperson
Chairlift Operator
Cherry Picker Operator
Elevated Work Platform Operator
Pile Driver
Portainer Operator
Tower Crane Operator
Winch Operator
Winding Engine Driver

UNIT GROUP 7122 DRILLERS, MINERS AND SHOT FIRERS

DRILLERS, MINERS AND SHOT FIRERS assemble, position and operate drilling rigs and mining plant, and detonate explosives to extract materials from the earth and demolish structures.

Indicative Skill Level:
Most occupations in this unit group have a level of skill commensurate with the qualifications and experience outlined below.

In Australia:
AQF Certificate II or III (ANZSCO Skill Level 4)

In New Zealand:
NZ Register Level 2 or 3 qualification (ANZSCO Skill Level 4)

At least one year of relevant experience may substitute for the formal qualifications listed above. In some instances relevant experience and/or on-the-job training may be required in addition to the formal qualification.

Registration or licensing may be required.

Tasks Include:
- dismantling, moving and reassembling drilling rigs and accessory plant
- taking samples of ore, liquids and gases and packaging them
- performing minor maintenance and repairs, and lubricating and cleaning plant
- recording performance details and information obtained from wells, and keeping logs detailing operations
- operating surface and underground mining plant
- undertaking development work such as opening up new shafts, drives, air vents, rises and crib rooms
- positioning explosives in bore holes and priming explosives using detonators and explosive cartridges
- connecting wires, fuses and detonating cords to explosive cartridges and detonators, and detonating explosives
- monitoring operation of plant and ensuring safety of other workers on mining sites and during drilling operations
- operating auxiliary plant such as pumps to expel air, water and mud

Occupations:
712211 Driller
712212 Miner
712213 Shot Firer

UNIT GROUP 7122 DRILLERS, MINERS AND SHOT FIRERS *continued*

712211 DRILLER

Alternative Title:

Drilling Plant Operator

Assembles, positions and operates a drilling rig and related equipment to extract ore, liquids or gases from the earth. Registration or licensing may be required.

Skill Level: 4

Specialisations:

Development Driller
Directional Driller
Exploration Driller
Jumbo Operator
Power Tong Operator
Raise Drill Operator
Rig Manager
Rock Drill Operator
Stope Miner
Tool Pusher

712212 MINER

Alternative Title:

Mining Plant Operator

Operates plant to excavate, load and transport coal, ore and rock in an underground or open-cut mine. Registration or licensing may be required.

Skill Level: 4

Specialisations:

Bogger Operator
Coal Cutter
Dragline Operator
Opal Miner
Underground Truck Operator

712213 SHOT FIRER

Alternative Title:

Powder Monkey (Aus)

Assembles, positions and detonates explosives at a mining or demolition site. Registration or licensing may be required.

Skill Level: 4

Specialisation:

Seismograph Shooter

UNIT GROUP 7123 ENGINEERING PRODUCTION SYSTEMS WORKERS

ENGINEERING PRODUCTION SYSTEMS WORKERS perform a range of production process tasks to refine and treat metals and mineral ore, fire ceramics, and operate plant to produce and finish metal products such as rods, tubing and structural shapes, and moulds for casting.

Indicative Skill Level:
Most occupations in this unit group have a level of skill commensurate with the qualifications and experience outlined below.

In Australia:
AQF Certificate II or III (ANZSCO Skill Level 4)

In New Zealand:
NZ Register Level 2 or 3 qualification (ANZSCO Skill Level 4)

At least one year of relevant experience may substitute for the formal qualifications listed above. In some instances relevant experience and/or on-the-job training may be required in addition to the formal qualification.

Registration or licensing is required.

Tasks Include:

- interpreting engineering production drawings
- setting up, operating and adjusting production plant to shape metal stock and castings and cut sheet metal
- operating welding and electroplating plant
- operating furnaces and quenching plant to smelt and change the structure of metals
- using kilns and ovens to fire ceramics
- processing mineral ore and operating metal rolling plant
- casting molten metal and operating plant to draw metal wire through dies
- operating computer-controlled production plant

Occupation:
712311 Engineering Production Systems Worker

712311 ENGINEERING PRODUCTION SYSTEMS WORKER

Performs a range of production process tasks to refine and treat metals and mineral ore, fire ceramics, and operate plant to produce and finish metal products such as rods, tubing and structural shapes, and moulds for casting. Registration or licensing is required.

Skill Level: 4

Specialisations:
Alumina Refinery Operator
Arc Welder
Brake Press Operator
Computer Numeric Control Machine Operator
Foundry Operator
Furnace Operator (Metals)
Kiln Operator (Metals)
Metal Rolling Mill Operator
Mineral Ore Treatment Plant Operator
Sheetmetal Worker (Second Class)
Tool Setter
Turret Punch Operator

UNIT GROUP 7129 OTHER STATIONARY PLANT OPERATORS

This unit group covers Stationary Plant Operators not elsewhere classified.

It includes Boiler or Engine Operators, Bulk Materials Handling Plant Operators, Cement Production Plant Operators, Concrete Batching Plant Operators, Concrete Pump Operators, Paper and Pulp Mill Operators, Railway Signal Operators, Train Controllers, Waste Water or Water Plant Operators and Weighbridge Operators.

Indicative Skill Level:
Most occupations in this unit group have a level of skill commensurate with the qualifications and experience outlined below.

In Australia:
AQF Certificate II or III (ANZSCO Skill Level 4)

In New Zealand:
NZ Register Level 2 or 3 qualification (ANZSCO Skill Level 4)

At least one year of relevant experience may substitute for the formal qualifications listed above. In some instances relevant experience and/or on-the-job training may be required in addition to the formal qualification.

Registration or licensing may be required.

Occupations:
712911 Boiler or Engine Operator
712912 Bulk Materials Handling Plant Operator
712913 Cement Production Plant Operator
712914 Concrete Batching Plant Operator
712915 Concrete Pump Operator
712916 Paper and Pulp Mill Operator
712917 Railway Signal Operator
712918 Train Controller
712921 Waste Water or Water Plant Operator
712922 Weighbridge Operator
712999 Stationary Plant Operators nec

712911 BOILER OR ENGINE OPERATOR

Operates and maintains stationary engines, boilers, refrigeration and airconditioning systems, and associated mechanical plant. Registration or licensing is required.

Skill Level: 4

Specialisations:
Airconditioning Plant Operator
Marine Engine Driver
Motorman/woman (Fluids Drilling)
Refrigeration Plant Operator

UNIT GROUP 7129 OTHER STATIONARY PLANT OPERATORS *continued*

712912 BULK MATERIALS HANDLING PLANT OPERATOR

Operates plant to load, unload, move, store and stack bulk materials such as grain, sugar and mineral ore.

Skill Level: 4

Specialisations:

Bulk Fluids Handler
Conveyor Belt Operator
Grain Handler
Palletiser Operator
Tank Farm Operator (Petroleum)

712913 CEMENT PRODUCTION PLANT OPERATOR

Operates plant to produce cement, lime and clinker.

Skill Level: 4

Specialisations:

Cement Crusher Operator
Cement Despatch Operator
Cement Grinding Mill Operator
Cement Kiln Operator

712914 CONCRETE BATCHING PLANT OPERATOR

Operates mixing plant to produce batches of concrete from cement, sand, aggregate, water and other ingredients.

Skill Level: 4

Specialisation:

Premix Concrete Batcher

712915 CONCRETE PUMP OPERATOR

Operates plant to pump, cast and mould concrete. Registration or licensing is required.

Skill Level: 4

Specialisation:

Concrete Boom Operator

UNIT GROUP 7129 OTHER STATIONARY PLANT OPERATORS *continued*

712916 PAPER AND PULP MILL OPERATOR

Alternative Titles:
- Paper Machine Operator
- Paper Maker

Operates plant to produce paper pulp from woodchips and to make paper sheets.

Skill Level: 4

Specialisations:
- Bleach Plant Operator
- Dryerman/woman (Paper Mill)
- Fourdrinier Machine Operator
- Inverform Machine Operator
- Paperboard Machine Operator
- Paper Rewinder Operator
- Supercalender Operator (Papermaking)

712917 RAILWAY SIGNAL OPERATOR

Alternative Title:
- Railway Switching and Signalling Operator

Controls the movement of trains, and assembles and disassembles trains within a marshalling yard.

Skill Level: 4

Specialisations:
- Railway Shunter
- Railway Yard Assistant

712918 TRAIN CONTROLLER

Oversees the safe movement of trains using a computerised train control signalling system.

Skill Level: 4

712921 WASTE WATER OR WATER PLANT OPERATOR

Operates plant to store, distribute and treat water including purifying water for human consumption and removing wastes from sewage.

Skill Level: 4

712922 WEIGHBRIDGE OPERATOR

Operates weighing plant and issues measurement tickets which provide readings of vehicle and livestock weight.

Skill Level: 4

Specialisation:
- Licensed Weigher

UNIT GROUP 7129 OTHER STATIONARY PLANT OPERATORS *continued*

712999 STATIONARY PLANT OPERATORS NEC

This occupation group covers Stationary Plant Operators not elsewhere classified.

Skill Level: 4

Occupations in this group include:

- Air Compressor Operator
- Beverage Distiller
- Bitumen Plant Operator
- Car Compactor Operator
- Crematorium Operator
- Lock Master (Water Transport)
- Oilseed Processing Operator
- Snow Maker
- Timber Treatment Plant Operator
- Wash Plant Operator

SUB-MAJOR GROUP 72 MOBILE PLANT OPERATORS

MOBILE PLANT OPERATORS operate mobile plant to clear and cultivate land, sow and harvest crops, fell trees, move and excavate rock and soil, move pallets and containers, and lay roads and railway tracks.

Indicative Skill Level:
Most occupations in this sub-major group have a level of skill commensurate with the qualifications and experience outlined below.

In Australia:
AQF Certificate II or III (ANZSCO Skill Level 4)

In New Zealand:
NZ Register Level 2 or 3 qualification (ANZSCO Skill Level 4)

At least one year of relevant experience may substitute for the formal qualifications listed above. In some instances relevant experience and/or on-the-job training may be required in addition to the formal qualification.

Tasks Include:
- driving plant to work site
- driving and manoeuvring plant on site
- selecting and fitting attachments to plant
- manipulating controls to operate attachments mechanically, hydraulically and electrically, and to move materials
- monitoring operation of plant directly and by referring to instruments
- monitoring condition of plant, performing minor repairs and reporting mechanical faults

Occupations in this sub-major group are classified into the following minor group:
721 Mobile Plant Operators

MINOR GROUP 721 MOBILE PLANT OPERATORS

MOBILE PLANT OPERATORS operate mobile plant to clear and cultivate land, sow and harvest crops, fell trees, move and excavate rock and soil, move pallets and containers, and lay roads and railway tracks.

Indicative Skill Level:
Most occupations in this minor group have a level of skill commensurate with the qualifications and experience outlined below.

In Australia:
AQF Certificate II or III (ANZSCO Skill Level 4)

In New Zealand:
NZ Register Level 2 or 3 qualification (ANZSCO Skill Level 4)

At least one year of relevant experience may substitute for the formal qualifications listed above. In some instances relevant experience and/or on-the-job training may be required in addition to the formal qualification.

Tasks Include:

- driving plant to worksite
- driving and manoeuvring plant on site
- selecting and fitting attachments to plant
- manipulating controls to operate attachments mechanically, hydraulically and electrically, and to move materials
- monitoring operation of plant directly and by referring to instruments
- monitoring condition of plant, performing minor repairs and reporting mechanical faults

Occupations in this minor group are classified into the following unit groups:

7211 Agricultural, Forestry and Horticultural Plant Operators
7212 Earthmoving Plant Operators
7213 Forklift Drivers
7219 Other Mobile Plant Operators

UNIT GROUP 7211 AGRICULTURAL, FORESTRY AND HORTICULTURAL PLANT OPERATORS

AGRICULTURAL, FORESTRY AND HORTICULTURAL PLANT OPERATORS operate agricultural, forestry and horticultural plant to clear and cultivate land, sow and harvest crops, and fell trees and move logs.

Indicative Skill Level:
Most occupations in this unit group have a level of skill commensurate with the qualifications and experience outlined below.

In Australia:
AQF Certificate II or III (ANZSCO Skill Level 4)

In New Zealand:
NZ Register Level 2 or 3 qualification (ANZSCO Skill Level 4)

At least one year of relevant experience may substitute for the formal qualifications listed above. In some instances relevant experience and/or on-the-job training may be required in addition to the formal qualification.

Registration or licensing may be required.

Tasks Include:
- preparing and positioning plant for operation
- operating tractor-drawn and self-propelled plant to plough land and sow, fertilise, cultivate and harvest crops, and avoid damaging crops
- adjusting speed, height and depth of implements
- operating plant to hold, lift and cut trees
- operating attachments to lift, swing, release and sort trees and logs, and operating auxiliary plant such as chipping machines and log splitting machines
- feeding felled trees into processors to strip limbs and cut into logs and loading logs onto stockpiles and into trucks
- keeping log tallies and writing work reports
- servicing plant and performing minor repairs

Occupations:
721111 Agricultural and Horticultural Mobile Plant Operator
721112 Logging Plant Operator

721111 AGRICULTURAL AND HORTICULTURAL MOBILE PLANT OPERATOR

Operates agricultural and horticultural plant to clear and cultivate land, and sow and harvest crops. Registration or licensing may be required.

Skill Level: 4

Specialisations:
Cotton Picking Machine Operator (Aus)
Harvester Operator
Rotary Hoe Operator

UNIT GROUP 7211 AGRICULTURAL, FORESTRY AND HORTICULTURAL PLANT OPERATORS *continued*

721112 LOGGING PLANT OPERATOR

Operates plant to fell trees and drag, transport and load logs onto trucks. Registration or licensing may be required.

Skill Level: 4

Specialisations:
- Forwarder Operator
- Skidder Operator
- Tree Feller Operator

UNIT GROUP 7212 EARTHMOVING PLANT OPERATORS

EARTHMOVING PLANT OPERATORS operate plant to excavate earth, ore and rock, break up pavement, road, rock and obstructions, move and load earth, rock and debris, and level, smooth and compact surfaces in construction and other projects.

Indicative Skill Level:
Most occupations in this unit group have a level of skill commensurate with the qualifications and experience outlined below.

In Australia:
AQF Certificate II or III (ANZSCO Skill Level 4)

In New Zealand:
NZ Register Level 2 or 3 qualification (ANZSCO Skill Level 4)

At least one year of relevant experience may substitute for the formal qualifications listed above. In some instances relevant experience and/or on-the-job training may be required in addition to the formal qualification.

Registration or licensing is required.

Tasks Include:
- preparing and positioning plant for operation
- selecting, fitting and removing attachments such as buckets, winches, loading scoops, shovel blades and rock breaking hammers
- operating controls to excavate, break, drill, level, compact, gouge out, move, load and spread earth, rock, rubble, soil and other materials
- monitoring operation of plant and adjusting controls to regulate pressure, speed and flow of operation, and ensuring safety of other workers
- raising, lowering and manipulating attachments using manual and hydraulic controls
- working from drawings, markers and verbal instructions
- servicing, lubricating, cleaning and refuelling plant and performing minor adjustments and repairs

Occupations:
721211 Earthmoving Plant Operator (General)
721212 Backhoe Operator
721213 Bulldozer Operator
721214 Excavator Operator
721215 Grader Operator
721216 Loader Operator

721211 EARTHMOVING PLANT OPERATOR (GENERAL)

Alternative Title:
Construction Plant Operator (General)

Operates a range of earthmoving plant to assist with building roads, rail, water supply, dams, treatment plants and agricultural earthworks. Registration or licensing is required.

Skill Level: 4

UNIT GROUP 7212 EARTHMOVING PLANT OPERATORS *continued*

721212 BACKHOE OPERATOR

Operates a backhoe and attachments to excavate, break, drill, level and compact earth, rock and other material. Registration or licensing is required.

Skill Level: 4

721213 BULLDOZER OPERATOR

Operates a bulldozer using blades and other attachments to gouge out, level and move materials in construction, forestry, mining and other projects. Registration or licensing is required.

Skill Level: 4

Specialisation:
- Scraper Operator (Earthmoving)

721214 EXCAVATOR OPERATOR

Operates heavy excavation plant to excavate, move and load earth, rock and rubble. Registration or licensing is required.

Skill Level: 4

Specialisations:
- Hydraulic Rockbreaker Operator
- Trench Digging Machine Operator

721215 GRADER OPERATOR

Operates a grader to spread and level materials in construction projects. Registration or licensing is required.

Skill Level: 4

721216 LOADER OPERATOR

Operates a motorised loader to move and load soil, rock and other material. Registration or licensing is required.

Skill Level: 4

Specialisations:
- Bobcat Operator
- Front-end Loader Operator

UNIT GROUP 7213 FORKLIFT DRIVERS

FORKLIFT DRIVERS operate forklifts to move bulk materials, containers, crates, palletised goods, cartons and bales.

Indicative Skill Level:
Most occupations in this unit group have a level of skill commensurate with the qualifications and experience outlined below.

In Australia:
AQF Certificate II or III (ANZSCO Skill Level 4)

In New Zealand:
NZ Register Level 2 or 3 qualification (ANZSCO Skill Level 4)

At least one year of relevant experience may substitute for the formal qualifications listed above. In some instances relevant experience and/or on-the-job training may be required in addition to the formal qualification.

Registration or licensing may be required.

Tasks Include:

- operating controls to align forklifts and raise and lower forks to stack and unstack items in warehouses, factories, timber yards and shipping terminals
- operating forklifts which run on rails or use electronic guidance systems to control movements in narrow aisles
- transporting goods to designated areas in warehouses, factories, timber yards and shipping terminals
- ensuring goods are stored in correct areas so that they can be easily located when orders are made up
- monitoring equipment operation visually through gauges and instruments and through computerised monitoring equipment
- inspecting and controlling equipment to identify wear and damage
- servicing and performing minor repairs and adjustments to forklifts
- may operate specialised trucks to carry items beneath elevated frames

Occupation:
721311 Forklift Driver

721311 FORKLIFT DRIVER

Alternative Titles:
Forklift Operator
Fork Truck Operator

Operates a forklift to move bulk materials, containers, crates, palletised goods, cartons and bales. Registration or licensing may be required.

Skill Level: 4

Specialisation:
Reach Truck Operator

UNIT GROUP 7219 OTHER MOBILE PLANT OPERATORS

This unit group covers Mobile Plant Operators not elsewhere classified.

It includes Aircraft Baggage Handlers and Airline Ground Crew, Linemarkers, Paving Plant Operators, Railway Track Plant Operators, Road Roller Operators and Streetsweeper Operators.

Indicative Skill Level:
Most occupations in this unit group have a level of skill commensurate with the qualifications and experience outlined below.

In Australia:
AQF Certificate II or III (ANZSCO Skill Level 4)

In New Zealand:
NZ Register Level 2 or 3 qualification (ANZSCO Skill Level 4)

At least one year of relevant experience may substitute for the formal qualifications listed above. In some instances relevant experience and/or on-the-job training may be required in addition to the formal qualification.

Registration or licensing may be required.

Occupations:
721911 Aircraft Baggage Handler and Airline Ground Crew
721912 Linemarker
721913 Paving Plant Operator
721914 Railway Track Plant Operator
721915 Road Roller Operator
721916 Streetsweeper Operator
721999 Mobile Plant Operators nec

721911 AIRCRAFT BAGGAGE HANDLER AND AIRLINE GROUND CREW

Loads and unloads baggage, directs planes, positions staircases, fills aircraft fuel tanks and performs other aircraft ground services to ensure aircraft operations run efficiently. Registration or licensing is required.

Skill Level: 4

Specialisation:
Ramp Agent

721912 LINEMARKER

Operates plant to apply markings to roads and other surfaces such as car parks, airports and sportsgrounds. Registration or licensing is required.

Skill Level: 4

Specialisation:
Road Marker

721913 PAVING PLANT OPERATOR

Operates plant to spread and level hot bituminous paving materials and lay concrete on areas such as highways, roads and car parks. Registration or licensing is required.

Skill Level: 4

UNIT GROUP 7219 OTHER MOBILE PLANT OPERATORS *continued*

721914 RAILWAY TRACK PLANT OPERATOR

Operates plant to lay, align, repair and maintain railway tracks. Registration or licensing may be required.

Skill Level: 4

721915 ROAD ROLLER OPERATOR

Operates a power-driven roller to prepare surfaces for roads, runways and car parks. Registration or licensing is required.

Skill Level: 4

721916 STREETSWEEPER OPERATOR

Operates plant to clean streets and gutters of litter and debris. Registration or licensing is required.

Skill Level: 4

721999 MOBILE PLANT OPERATORS NEC

This occupation group covers Mobile Plant Operators not elsewhere classified. Registration or licensing may be required.

Skill Level: 4

Occupations in this group include:

- Cable Ferry Operator
- Dredge Operator
- Mulcher Operator
- Snow Groomer
- Straddle Carrier Operator
- Tunnelling Plant Operator

SUB-MAJOR GROUP 73 ROAD AND RAIL DRIVERS

ROAD AND RAIL DRIVERS drive cars, buses, coaches, trains, trams, vans and trucks to transport passengers and freight.

Indicative Skill Level:
Most occupations in this sub-major group have a level of skill commensurate with the qualifications and experience outlined below.

In Australia:
AQF Certificate II or III (ANZSCO Skill Level 4)

In New Zealand:
NZ Register Level 2 or 3 qualification (ANZSCO Skill Level 4)

At least one year of relevant experience may substitute for the formal qualifications listed above. In some instances relevant experience and/or on-the-job training may be required in addition to the formal qualification.

Tasks Include:
- stopping at designated locations to pick up and set down passengers and freight
- establishing destinations and determining most appropriate routes
- ensuring passengers and freight arrive at destinations on time
- manoeuvring vehicles into position for loading and unloading
- assisting with loading and unloading operations using lifting and tipping devices
- observing safety requirements when loading and unloading vehicles

Occupations in this sub-major group are classified into the following minor groups:
731 Automobile, Bus and Rail Drivers
732 Delivery Drivers
733 Truck Drivers

MINOR GROUP 731 AUTOMOBILE, BUS AND RAIL DRIVERS

AUTOMOBILE, BUS AND RAIL DRIVERS drive cars, buses, coaches, trains and trams to transport passengers and freight.

Indicative Skill Level:
Most occupations in this minor group have a level of skill commensurate with the qualifications and experience outlined below.

In Australia:
AQF Certificate II or III (ANZSCO Skill Level 4)

In New Zealand:
NZ Register Level 2 or 3 qualification (ANZSCO Skill Level 4)

At least one year of relevant experience may substitute for the formal qualifications listed above. In some instances relevant experience and/or on-the-job training may be required in addition to the formal qualification.

Tasks Include:
- stopping at designated locations to pick up and set down passengers and freight
- collecting fares and giving change
- controlling lighting, heating and ventilation to ensure passenger comfort
- observing prescribed speeds, nearby traffic, travelling conditions and signals to ensure safe arrival of passengers and freight
- ensuring passengers and freight arrive at destinations on time

Occupations in this minor group are classified into the following unit groups:
7311 Automobile Drivers
7312 Bus and Coach Drivers
7313 Train and Tram Drivers

UNIT GROUP 7311 AUTOMOBILE DRIVERS

AUTOMOBILE DRIVERS drive motor cars to transport passengers to destinations.

Indicative Skill Level:
Most occupations in this unit group have a level of skill commensurate with the qualifications and experience outlined below.

In Australia:
AQF Certificate II or III (ANZSCO Skill Level 4)

In New Zealand:
NZ Register Level 2 or 3 qualification (ANZSCO Skill Level 4)

At least one year of relevant experience may substitute for the formal qualifications listed above. In some instances relevant experience and/or on-the-job training may be required in addition to the formal qualification.

Registration or licensing is required.

Tasks Include:
- using mobile computer systems and radio networks to log into waiting passenger information
- picking up passengers at designated locations or when hailed
- checking passenger destinations and determining most appropriate route
- transporting passengers to desired destinations
- assisting passengers with luggage
- collecting fares and processing fare payments
- may collect and deliver parcels

Occupations:
731111 Chauffeur
731112 Taxi Driver
731199 Automobile Drivers nec

731111 CHAUFFEUR

Drives a limousine, van or private car to transport passengers to destinations on a fee-for-service basis, usually on a long-term hiring arrangement. Registration or licensing is required.

Skill Level: 4

Specialisations:
Hire Car Driver
Limousine Driver

731112 TAXI DRIVER

Drives a taxi to transport passengers to destinations on a fee-for-service basis, usually on a short-term, metered fare hiring arrangement. Registration or licensing is required.

Skill Level: 4

UNIT GROUP 7311 AUTOMOBILE DRIVERS *continued*

731199 AUTOMOBILE DRIVERS NEC

This occupation group covers Automobile Drivers not elsewhere classified. Registration or licensing is required.

Skill Level: 4

Occupations in this group include:

- Oversize Load Pilot Escort
- Rental Car Ferry Driver

UNIT GROUP 7312 BUS AND COACH DRIVERS

BUS AND COACH DRIVERS drive buses and coaches to transport passengers over established and special routes.

Indicative Skill Level:
Most occupations in this unit group have a level of skill commensurate with the qualifications and experience outlined below.

In Australia:
AQF Certificate II or III (ANZSCO Skill Level 4)

In New Zealand:
NZ Register Level 2 or 3 qualification (ANZSCO Skill Level 4)

At least one year of relevant experience may substitute for the formal qualifications listed above. In some instances relevant experience and/or on-the-job training may be required in addition to the formal qualification.

Registration or licensing is required.

Tasks Include:
- stopping at set locations to pick up and set down passengers
- opening and closing doors before and after passengers board or alight
- controlling lighting, heating and ventilation on buses
- collecting fares and giving change and tickets, and monitoring electronic entry
- advising passengers on destinations
- maintaining conduct of passengers
- may use public address systems to provide information and tour commentaries for passengers
- may assist coach passengers with baggage and accommodation bookings
- may maintain, service and clean coaches

Occupations:
731211 Bus Driver
731212 Charter and Tour Bus Driver
731213 Passenger Coach Driver

731211 BUS DRIVER

Drives a bus to transport passengers short distances on scheduled intra-city services over established routes. Registration or licensing is required.

Skill Level: 4

Specialisations:
Minibus Driver
School Bus Driver

731212 CHARTER AND TOUR BUS DRIVER

Drives a coach to transport passengers on sightseeing, educational and other tours. Registration or licensing is required.

Skill Level: 4

Specialisation:
Coach Tour Driver

UNIT GROUP 7312 BUS AND COACH DRIVERS *continued*

731213 PASSENGER COACH DRIVER

Drives a coach to transport passengers long distances on scheduled intercity services over established routes. Registration or licensing is required.

Skill Level: 4

UNIT GROUP 7313 TRAIN AND TRAM DRIVERS

TRAIN AND TRAM DRIVERS drive trains and trams to transport passengers and freight on rail networks.

Indicative Skill Level:
Most occupations in this unit group have a level of skill commensurate with the qualifications and experience outlined below.

In Australia:
AQF Certificate II or III (ANZSCO Skill Level 4)

In New Zealand:
NZ Register Level 2 or 3 qualification (ANZSCO Skill Level 4)

At least one year of relevant experience may substitute for the formal qualifications listed above. In some instances relevant experience and/or on-the-job training may be required in addition to the formal qualification.

Registration or licensing is required.

Tasks Include:
- stopping at stations and set locations to pick up and set down passengers and freight
- opening and closing doors before and after passengers board or alight
- observing signals, track conditions, nearby traffic and prescribed speeds to ensure safety
- monitoring indicator gauges, changing controls and power supply poles and reporting operating irregularities
- checking time and adherence to timetables
- may advise passengers on destinations

Occupations:
731311 Train Driver
731312 Tram Driver

731311 TRAIN DRIVER

Alternative Title:
Locomotive Driver

Drives a train to transport passengers and freight on railways. Registration or licensing is required.

Skill Level: 4

Specialisations:
Electric Train Driver
Fireperson (Railway)
Locomotive Observer
Rail Car Driver
Steam Train Driver

731312 TRAM DRIVER

Drives a tram to transport passengers on urban light rail networks. Registration or licensing is required.

Skill Level: 4

MINOR GROUP 732 DELIVERY DRIVERS

DELIVERY DRIVERS drive vans and cars to deliver goods.

Indicative Skill Level:
Most occupations in this minor group have a level of skill commensurate with the qualifications and experience outlined below.

In Australia:
AQF Certificate II or III (ANZSCO Skill Level 4)

In New Zealand:
NZ Register Level 2 or 3 qualification (ANZSCO Skill Level 4)

At least one year of relevant experience may substitute for the formal qualifications listed above. In some instances relevant experience and/or on-the-job training may be required in addition to the formal qualification.

Tasks Include:
- determining the destinations of goods and most appropriate delivery routes
- manoeuvring vehicles into position for loading and unloading
- assisting with loading to ensure goods are arranged for ease of delivery and safely secured to avoid damage
- verifying loading documents
- arranging and performing unloading operations and obtaining certification of deliveries
- reporting vehicle maintenance needs
- may receive payments for deliveries and arrange accounts

Occupations in this minor group are classified into the following unit group:
7321 Delivery Drivers

UNIT GROUP 7321 DELIVERY DRIVERS

DELIVERY DRIVERS drive vans and cars to deliver goods.

Indicative Skill Level:
Most occupations in this unit group have a level of skill commensurate with the qualifications and experience outlined below.

In Australia:
AQF Certificate II or III (ANZSCO Skill Level 4)

In New Zealand:
NZ Register Level 2 or 3 qualification (ANZSCO Skill Level 4)

At least one year of relevant experience may substitute for the formal qualifications listed above. In some instances relevant experience and/or on-the-job training may be required in addition to the formal qualification.

Registration or licensing is required.

Tasks Include:
- determining the destinations of goods and most appropriate delivery routes
- manoeuvring vehicles into position for loading and unloading
- assisting with loading to ensure goods are arranged for ease of delivery and safely secured to avoid damage
- verifying loading documents
- arranging and performing unloading operations and obtaining certification of deliveries
- reporting vehicle maintenance needs
- may receive payments for deliveries and arrange accounts

Occupation:
732111 Delivery Driver

732111 DELIVERY DRIVER

Alternative Title:
Van Driver

Drives a van or car to deliver goods. Registration or licensing is required.

Skill Level: 4

Specialisations:
Fast Food Delivery Driver
Grocery Deliverer
Meals on Wheels Driver
Taxi Truck Driver

MINOR GROUP 733 TRUCK DRIVERS

TRUCK DRIVERS drive heavy trucks, removal vans, tankers and tow trucks to transport bulky goods and liquids.

Indicative Skill Level:
Most occupations in this minor group have a level of skill commensurate with the qualifications and experience outlined below.

In Australia:
AQF Certificate II or III (ANZSCO Skill Level 4)

In New Zealand:
NZ Register Level 2 or 3 qualification (ANZSCO Skill Level 4)

At least one year of relevant experience may substitute for the formal qualifications listed above. In some instances relevant experience and/or on-the-job training may be required in addition to the formal qualification.

Tasks Include:
- manoeuvring vehicles into position for loading and unloading
- loading and unloading vehicles using lifting and tipping devices
- observing safety requirements when loading and unloading vehicles
- making regular quality checks of vehicles to ensure they can be driven safely
- estimating weights to comply with load limitations and ensuring safe distribution of weight
- ensuring goods are stowed and securely covered to prevent loss and damage
- verifying loading documents, checking condition of goods and obtaining certification of deliveries

Occupations in this minor group are classified into the following unit group:
7331 Truck Drivers

UNIT GROUP 7331 TRUCK DRIVERS

TRUCK DRIVERS drive heavy trucks, removal vans, tankers and tow trucks to transport bulky goods and liquids.

Indicative Skill Level:
Most occupations in this unit group have a level of skill commensurate with the qualifications and experience outlined below.

In Australia:
AQF Certificate II or III (ANZSCO Skill Level 4)

In New Zealand:
NZ Register Level 2 or 3 qualification (ANZSCO Skill Level 4)

At least one year of relevant experience may substitute for the formal qualifications listed above. In some instances relevant experience and/or on-the-job training may be required in addition to the formal qualification.

Registration or licensing is required.

Tasks Include:

- manoeuvring vehicles into position for loading and unloading
- loading and unloading vehicles using lifting and tipping devices
- observing safety requirements when loading and unloading vehicles
- making regular quality checks of vehicles to ensure they can be driven safely
- estimating weights to comply with load limitations and ensuring safe distribution of weight
- ensuring goods are stowed and securely covered to prevent loss and damage
- verifying loading documents, checking condition of goods and obtaining certification of deliveries

Occupations:
733111 Truck Driver (General)
733112 Aircraft Refueller
733113 Furniture Removalist
733114 Tanker Driver
733115 Tow Truck Driver

733111 TRUCK DRIVER (GENERAL)

Drives a heavy truck, requiring a specially endorsed class of licence, to transport bulky goods. Registration or licensing is required.

Skill Level: 4

Specialisations:
Cement Mixer Driver
Compactor Driver (Rubbish Collection)
Haulpak Driver
Livestock Haulier
Logging Truck Driver
Road Train Driver
Tilt Tray Driver

UNIT GROUP 7331 TRUCK DRIVERS *continued*

733112 AIRCRAFT REFUELLER

Drives a tanker truck filled with aviation fuel to waiting aircraft, attaches a fuel hose to aircraft fuel tank and fills it with fuel. Registration or licensing is required.

Skill Level: 4

Specialisation:
- Ground Crewman Aircraft Support (Army)

733113 FURNITURE REMOVALIST

Drives a removal van or truck to move household and office furniture and equipment between locations. Registration or licensing is required.

Skill Level: 4

Specialisations:
- Office Mover
- Piano Removalist

733114 TANKER DRIVER

Drives a tanker truck, requiring a specially endorsed class of licence, to transport bulk liquids. Registration or licensing is required.

Skill Level: 4

Specialisations:
- Milk Tanker Driver
- Petrol Tanker Driver
- Water Tanker Driver

733115 TOW TRUCK DRIVER

Drives a tow truck, requiring a specially endorsed class of licence, to transport broken-down motor vehicles. Registration or licensing is required.

Skill Level: 4

Specialisation:
- Mechanic Recovery (Army)

SUB-MAJOR GROUP 74 STOREPERSONS

STOREPERSONS receive, handle and despatch goods in stores and warehouses.

Indicative Skill Level:
Most occupations in this sub-major group have a level of skill commensurate with the qualifications and experience outlined below.

In Australia:
AQF Certificate II or III (ANZSCO Skill Level 4)

In New Zealand:
NZ Register Level 2 or 3 qualification (ANZSCO Skill Level 4)

At least one year of relevant experience may substitute for the formal qualifications listed above. In some instances relevant experience and/or on-the-job training may be required in addition to the formal qualification.

Tasks Include:
- receiving incoming goods, checking for damage and for discrepancies between goods and invoices
- unloading vehicles, opening packages and removing contents
- operating computers to obtain details of location and quantity of items in stock
- labelling goods with details of storage location
- packing and weighing goods and sealing boxes
- operating machines to lift, place and remove goods on high levels
- operating specialised equipment, such as manually and electronically guided order pickers, and checking goods off picking list
- assisting with regular stocktakes
- may use materials handling equipment, such as hydraulic pallet lifters and hand trucks, to move goods

Occupations in this sub-major group are classified into the following minor group:
741 Storepersons

MINOR GROUP 741 STOREPERSONS

STOREPERSONS receive, handle and despatch goods in stores and warehouses.

Indicative Skill Level:
Most occupations in this minor group have a level of skill commensurate with the qualifications and experience outlined below.

In Australia:
AQF Certificate II or III (ANZSCO Skill Level 4)

In New Zealand:
NZ Register Level 2 or 3 qualification (ANZSCO Skill Level 4)

At least one year of relevant experience may substitute for the formal qualifications listed above. In some instances relevant experience and/or on-the-job training may be required in addition to the formal qualification.

Tasks Include:

- receiving incoming goods, checking for damage and for discrepancies between goods and invoices
- unloading vehicles, opening packages and removing contents
- operating computers to obtain details of location and quantity of items in stock
- labelling goods with details of storage location
- packing and weighing goods and sealing boxes
- operating machines to lift, place and remove goods on high levels
- operating specialised equipment, such as manually and electronically guided order pickers, and checking goods off picking list
- assisting with regular stocktakes
- may use materials handling equipment, such as hydraulic pallet lifters and hand trucks, to move goods

Occupations in this minor group are classified into the following unit group:
7411 Storepersons

UNIT GROUP 7411 STOREPERSONS

STOREPERSONS receive, handle and despatch goods in stores and warehouses.

Indicative Skill Level:
Most occupations in this unit group have a level of skill commensurate with the qualifications and experience outlined below.

In Australia:
AQF Certificate II or III (ANZSCO Skill Level 4)

In New Zealand:
NZ Register Level 2 or 3 qualification (ANZSCO Skill Level 4)

At least one year of relevant experience may substitute for the formal qualifications listed above. In some instances relevant experience and/or on-the-job training may be required in addition to the formal qualification.

Tasks Include:
- receiving incoming goods, checking for damage and for discrepancies between goods and invoices
- unloading vehicles, opening packages and removing contents
- operating computers to obtain details of location and quantity of items in stock
- labelling goods with details of storage location
- packing and weighing goods and sealing boxes
- operating machines to lift, place and remove goods on high levels
- operating specialised equipment, such as manually and electronically guided order pickers, and checking goods off picking list
- assisting with regular stocktakes
- may use materials handling equipment, such as hydraulic pallet lifters and hand trucks, to move goods

Occupation:
741111 Storeperson

741111 STOREPERSON

Alternative Titles:
Stores Assistant
Warehouse Assistant

Receives, handles and despatches goods in a store or warehouse.

Skill Level: 4

Specialisations:
Chiller Hand
Manufacturing Storeperson
Operator Supply (Army)
Order Picker/Assembler
Stores Despatch Hand
Stores Naval (Navy)

MAJOR GROUP 8 LABOURERS

LABOURERS perform a variety of routine and repetitive physical tasks using hand and power tools, and machines either as an individual or as part of a team assisting more skilled workers such as Trades Workers, and Machinery Operators and Drivers.

Indicative Skill Level:
Most occupations in this major group have a level of skill commensurate with the qualifications and experience outlined below.

In Australia:
AQF Certificate II or III, or at least one year of relevant experience (ANZSCO Skill Level 4); or
AQF Certificate I, or compulsory secondary education (ANZSCO Skill Level 5)

In New Zealand:
NZ Register Level 2 or 3 qualification, or at least one year of relevant experience (ANZSCO Skill Level 4); or
NZ Register Level 1 qualification, or compulsory secondary education (ANZSCO Skill Level 5)

In some instances relevant experience and/or on-the-job training may be required in addition to the formal qualification. In the case of some Skill Level 5 occupations, a short period of on-the-job training may be required in addition to or instead of the formal qualification, or no formal qualification or on-the-job training may be required.

Tasks Include:
- cleaning commercial, industrial and domestic premises, vehicles and machines
- spreading, levelling and finishing concrete and bituminous paving materials, and assembling and erecting scaffolding and rigging
- loading and unloading machines, assembling components, and grading, inspecting and packing products
- assisting with cultivating and harvesting crops, plants and forests, and with livestock production
- processing meat and seafood, and assisting with producing and preparing food
- loading and unloading freight from trucks, trains and ships, and stocking shelves in stores and supermarkets

Occupations in this major group are classified into the following sub-major groups:
81 Cleaners and Laundry Workers
82 Construction and Mining Labourers
83 Factory Process Workers
84 Farm, Forestry and Garden Workers
85 Food Preparation Assistants
89 Other Labourers

SUB-MAJOR GROUP 81 CLEANERS AND LAUNDRY WORKERS

CLEANERS AND LAUNDRY WORKERS clean vehicles, commercial, industrial and domestic premises, construction sites and industrial machines, and clothing and other items in laundries and drycleaning establishments.

Indicative Skill Level:
Most occupations in this sub-major group have a level of skill commensurate with the qualifications outlined below.

In Australia:
AQF Certificate I, or compulsory secondary education (ANZSCO Skill Level 5)

In New Zealand:
NZ Register Level 1 qualification, or compulsory secondary education (ANZSCO Skill Level 5)

For some occupations a short period of on-the-job training may be required in addition to or instead of the formal qualification. In some instances no formal qualification or on-the-job training may be required.

Tasks Include:
- removing rubbish and emptying containers, bins and trays
- dusting and polishing furniture, fixtures and fittings
- vacuuming and steam cleaning carpets, upholstery, curtains and floors
- applying cleaning agents to remove stains and dirt
- washing articles to remove stains and dirt
- picking up, sorting, washing, drying and ironing linen and clothes
- cleaning windows and other glass surfaces

Occupations in this sub-major group are classified into the following minor group:
811 Cleaners and Laundry Workers

MINOR GROUP 811 CLEANERS AND LAUNDRY WORKERS

CLEANERS AND LAUNDRY WORKERS clean vehicles, commercial, industrial and domestic premises, construction sites and industrial machines, and clothing and other items in laundries and drycleaning establishments.

Indicative Skill Level:
Most occupations in this minor group have a level of skill commensurate with the qualifications outlined below.

In Australia:
AQF Certificate I, or compulsory secondary education (ANZSCO Skill Level 5)

In New Zealand:
NZ Register Level 1 qualification, or compulsory secondary education (ANZSCO Skill Level 5)

For some occupations a short period of on-the-job training may be required in addition to or instead of the formal qualification. In some instances no formal qualification or on-the-job training may be required.

Tasks Include:
- removing rubbish and emptying containers, bins and trays
- dusting and polishing furniture, fixtures and fittings
- vacuuming and steam cleaning carpets, upholstery, curtains and floors
- applying cleaning agents to remove stains and dirt
- washing articles to remove stains and dirt
- picking up, sorting, washing, drying and ironing linen and clothes
- cleaning windows and other glass surfaces

Occupations in this minor group are classified into the following unit groups:
8111 Car Detailers
8112 Commercial Cleaners
8113 Domestic Cleaners
8114 Housekeepers
8115 Laundry Workers
8116 Other Cleaners

UNIT GROUP 8111 CAR DETAILERS

CAR DETAILERS wash and clean exteriors and interiors of motor vehicles, and touch up paint work, glass and upholstery to prepare them for sale or rent.

Indicative Skill Level:
Most occupations in this unit group have a level of skill commensurate with the qualifications outlined below.

In Australia:
AQF Certificate I, or compulsory secondary education (ANZSCO Skill Level 5)

In New Zealand:
NZ Register Level 1 qualification, or compulsory secondary education (ANZSCO Skill Level 5)

For some occupations a short period of on-the-job training may be required in addition to or instead of the formal qualification. In some instances no formal qualification or on-the-job training may be required.

Tasks Include:
- washing, drying, polishing and waxing vehicle exteriors
- vacuuming vehicle interiors, and drycleaning carpets and upholstery
- applying cleaning agents to remove stains from vehicle interiors
- washing tyres and wheel arches, and blackening tyres
- washing and polishing vehicle windows
- emptying and cleaning compartments in vehicles
- may make minor repairs and touch up finishes to remove scratches

Occupation:
811111 Car Detailer

811111 CAR DETAILER

Alternative Title:
Vehicle Detailer

Washes and cleans exteriors and interiors of motor vehicles, and touches up paint work, glass and upholstery to prepare them for sale or rent.

Skill Level: 5

UNIT GROUP 8112 COMMERCIAL CLEANERS

COMMERCIAL CLEANERS clean offices, residential complexes, hospitals, schools, industrial work areas, industrial machines, construction sites and other commercial premises using heavy duty cleaning equipment.

Indicative Skill Level:
Most occupations in this unit group have a level of skill commensurate with the qualifications outlined below.

In Australia:
AQF Certificate I, or compulsory secondary education (ANZSCO Skill Level 5)

In New Zealand:
NZ Register Level 1 qualification, or compulsory secondary education (ANZSCO Skill Level 5)

For some occupations a short period of on-the-job training may be required in addition to or instead of the formal qualification. In some instances no formal qualification or on-the-job training may be required.

Tasks Include:
- vacuuming carpets, curtains and upholstered furniture
- cleaning, dusting and polishing furniture, fixtures and fittings
- removing rubbish and recyclable material, and emptying containers, bins and trays
- stripping wax from floors, re-waxing and polishing floors
- cleaning and disinfecting laundry and bathroom fixtures, replenishing supplies and reporting defective plumbing fixtures
- operating industrial vacuum cleaners to clean floors, work areas and machines
- removing dust and dirt from ceilings, walls, overhead pipes and fixtures
- applying acids and solvents to surfaces to remove stains and dirt
- removing lint, dust, soot, oil, grease, sludge and other residues from machines, hulls and holds of ships, and interiors and exteriors of furnaces, boilers and tanks
- may clean exteriors of buildings by sand-blasting and applying solvents

Occupation:
811211 Commercial Cleaner

811211 COMMERCIAL CLEANER

Cleans offices, residential complexes, hospitals, schools, industrial work areas, industrial machines, construction sites and other commercial premises using heavy duty cleaning equipment.

Skill Level: 5

Specialisations:
Aircraft Cabin Cleaner
School Cleaner

UNIT GROUP 8113 DOMESTIC CLEANERS

DOMESTIC CLEANERS clean and tidy private dwellings such as houses, units, flats, apartments and townhouses.

Indicative Skill Level:
Most occupations in this unit group have a level of skill commensurate with the qualifications outlined below.

In Australia:
AQF Certificate I, or compulsory secondary education (ANZSCO Skill Level 5)

In New Zealand:
NZ Register Level 1 qualification, or compulsory secondary education (ANZSCO Skill Level 5)

For some occupations a short period of on-the-job training may be required in addition to or instead of the formal qualification. In some instances no formal qualification or on-the-job training may be required.

Tasks Include:
- vacuuming carpets, curtains and upholstered furniture
- sweeping, mopping, waxing and polishing tiled, vinyl, timber and concrete floors
- tidying rooms, emptying wastepaper bins and removing refuse and recyclable material
- cleaning, disinfecting and deodorising kitchens, bathrooms and toilets
- dusting, cleaning and polishing furniture and other homewares
- cleaning windows and other glass surfaces

Occupation:
811311 Domestic Cleaner

811311 DOMESTIC CLEANER

Cleans and tidies private dwellings such as houses, units, flats, apartments and townhouses.

Skill Level: 5

UNIT GROUP 8114 HOUSEKEEPERS

HOUSEKEEPERS perform cleaning and housekeeping duties in hotels, motels and other commercial premises, and in private residences.

Indicative Skill Level:
Most occupations in this unit group have a level of skill commensurate with the qualifications outlined below.

In Australia:
AQF Certificate I, or compulsory secondary education (ANZSCO Skill Level 5)

In New Zealand:
NZ Register Level 1 qualification, or compulsory secondary education (ANZSCO Skill Level 5)

For some occupations a short period of on-the-job training may be required in addition to or instead of the formal qualification. In some instances no formal qualification or on-the-job training may be required.

Tasks Include:
- cleaning the interior of buildings and the immediate outside areas
- sweeping, mopping and polishing floors, vacuuming and shampooing carpets, and cleaning curtains and upholstered furniture
- dusting and polishing furniture, fixtures and fittings
- picking up rubbish, emptying garbage containers, and taking contents to waste areas for removal
- restocking minibars and replenishing items such as drinking glasses, writing equipment, linen and groceries
- stripping and making beds, and changing bed linen
- maintaining kitchens, washing dishes and cooking utensils, and cleaning appliances, cupboards, counters, pantries and floors
- picking up, sorting, washing, drying, ironing and mending linen and clothes
- preparing and cooking meals, setting and clearing tables, and serving food and beverages
- taking care of household pets and plants, receiving visitors, answering telephones, delivering messages, and shopping for groceries

Occupations:
811411 Commercial Housekeeper
811412 Domestic Housekeeper

811411 COMMERCIAL HOUSEKEEPER

Cleans, vacuums and mops floors, makes beds, and re-stocks mini bars and bathroom supplies in hotel and motel rooms and other commercial premises.

Skill Level: 5

811412 DOMESTIC HOUSEKEEPER

Cleans, cooks and performs other housekeeping tasks in private residences.

Skill Level: 5

UNIT GROUP 8115 LAUNDRY WORKERS

LAUNDRY WORKERS sort, clean, fold, iron and package linen, clothing and other items in laundries and drycleaning establishments, and private residences.

Indicative Skill Level:
Most occupations in this unit group have a level of skill commensurate with the qualifications outlined below.

In Australia:
AQF Certificate I, or compulsory secondary education (ANZSCO Skill Level 5)

In New Zealand:
NZ Register Level 1 qualification, or compulsory secondary education (ANZSCO Skill Level 5)

For some occupations a short period of on-the-job training may be required in addition to or instead of the formal qualification. In some instances no formal qualification or on-the-job training may be required.

Tasks Include:
- sorting articles for cleaning according to the type, colour, fabric and cleaning treatment required
- placing sorted articles into receptacles and onto conveyor belts for moving to repair and cleaning areas
- checking and removing stains from garments, and replacing buttons and making minor repairs
- loading and unloading washing machines, driers and extractors
- adding cleaning agents and starches to articles
- smoothing articles and guiding them through cleaning and pressing machines
- stopping and starting machines to untangle, straighten and remove articles
- ironing and pressing clean articles
- placing articles on shelves and hanging articles for delivery and collection
- packaging articles and preparing orders for despatch

Occupations:
811511 Laundry Worker (General)
811512 Drycleaner
811513 Ironer or Presser

811511 LAUNDRY WORKER (GENERAL)

Sorts, cleans, irons, folds and packages linen, clothing and other garments in a commercial laundry.

Skill Level: 5

Specialisations:
Folding Machine Operator
Linen Sorter

811512 DRYCLEANER

Cleans clothing, garments, upholstery and other fabrics using drycleaning agents and machines.

Skill Level: 5

811513 IRONER OR PRESSER

Irons or presses garments and other fabrics, such as delicate and formal wear, in a commercial laundry or private residence.

Skill Level: 5

UNIT GROUP 8116 OTHER CLEANERS

OTHER CLEANERS clean surfaces, materials and objects, such as carpets, windows, walls, swimming pools and cooling towers, using specialised cleaning equipment and chemicals.

It includes Carpet Cleaners and Window Cleaners.

Indicative Skill Level:
Most occupations in this unit group have a level of skill commensurate with the qualifications outlined below.

In Australia:
AQF Certificate I, or compulsory secondary education (ANZSCO Skill Level 5)

In New Zealand:
NZ Register Level 1 qualification, or compulsory secondary education (ANZSCO Skill Level 5)

For some occupations a short period of on-the-job training may be required in addition to or instead of the formal qualification. In some instances no formal qualification or on-the-job training may be required.

Tasks Include:
- cleaning carpets and upholstered furniture using cleaning machines and their attachments
- selecting and applying cleaning agents to remove stains from carpets, windows and surfaces
- filling carpet cleaning machines with water and other cleaning agents
- pushing pile-lifting machines over carpets and brushing pile to raise and fluff nap
- treating carpets with soil-repellent chemicals and deodorants, and treating for pests
- using ladders, swinging scaffolds, bosun's chairs, hydraulic bucket trucks and other equipment to reach and clean windows in multi-storey buildings
- cleaning stone walls, metal surfaces, fascias and window frames using high pressure water cleaners and solvents
- applying chemicals and high pressure cleaning methods to remove micro-organisms from water and filtration systems, and using wet vacuums and other suction equipment to remove scale, accumulated dirt and other deposits from swimming pools, cooling tower components and drains

Occupations:
811611 Carpet Cleaner
811612 Window Cleaner
811699 Cleaners nec

811611 CARPET CLEANER

Cleans carpets, rugs and furniture upholstery using powder, liquid and steam cleaning methods, and applies soil-repellent chemicals and deodorants.

Skill Level: 5

Specialisation:
Upholstery Cleaner

811612 WINDOW CLEANER

Cleans interior and exterior window surfaces.

Skill Level: 5

UNIT GROUP 8116 OTHER CLEANERS *continued*

811699 CLEANERS NEC

This occupation group covers Cleaners not elsewhere classified.

Skill Level: 5

Occupations in this group include:

- Chimney Sweep
- Graffiti Cleaner
- Swimming Pool Cleaner

SUB-MAJOR GROUP 82 CONSTRUCTION AND MINING LABOURERS

CONSTRUCTION AND MINING LABOURERS perform a variety of routine tasks in house building, and road, rail and general construction, and in drilling, mining and mineral ore treatment, usually under close supervision.

Indicative Skill Level:
Most occupations in this sub-major group have a level of skill commensurate with the qualifications and experience outlined below.

In Australia:
AQF Certificate II or III, or at least one year of relevant experience (ANZSCO Skill Level 4); or
AQF Certificate I, or compulsory secondary education (ANZSCO Skill Level 5)

In New Zealand:
NZ Register Level 2 or 3 qualification, or at least one year of relevant experience (ANZSCO Skill Level 4); or
NZ Register Level 1 qualification, or compulsory secondary education (ANZSCO Skill Level 5)

In some instances relevant experience and/or on-the-job training may be required in addition to the formal qualification. In the case of some Skill Level 5 occupations, a short period of on-the-job training may be required in addition to or instead of the formal qualification, or no formal qualification or on-the-job training may be required.

Tasks Include:
- loading and unloading construction materials and equipment, and transporting them around building sites
- mixing, spreading and levelling concrete, plaster and mortar
- laying out fence lines and lifting and positioning posts
- insulating buildings, pipes and ducting
- fitting awnings, security screens and other home improvements
- assembling and erecting scaffolding and rigging
- digging holes and trenches, and spreading, levelling and compacting soil, gravel, sand and ballast
- erecting and dismantling barricades
- directing cranes

Occupations in this sub-major group are classified into the following minor group:
821 Construction and Mining Labourers

MINOR GROUP 821 CONSTRUCTION AND MINING LABOURERS

CONSTRUCTION AND MINING LABOURERS perform a variety of routine tasks in house building, and road, rail and general construction, and in drilling, mining and mineral ore treatment, usually under close supervision.

Indicative Skill Level:
Most occupations in this minor group have a level of skill commensurate with the qualifications and experience outlined below.

In Australia:
AQF Certificate II or III, or at least one year of relevant experience (ANZSCO Skill Level 4); or
AQF Certificate I, or compulsory secondary education (ANZSCO Skill Level 5)

In New Zealand:
NZ Register Level 2 or 3 qualification, or at least one year of relevant experience (ANZSCO Skill Level 4); or
NZ Register Level 1 qualification, or compulsory secondary education (ANZSCO Skill Level 5)

In some instances relevant experience and/or on-the-job training may be required in addition to the formal qualification. In the case of some Skill Level 5 occupations, a short period of on-the-job training may be required in addition to or instead of the formal qualification, or no formal qualification or on-the-job training may be required.

Tasks Include:
- loading and unloading construction materials and equipment, and transporting them around building sites
- mixing, spreading and levelling concrete, plaster and mortar
- laying out fence lines and lifting and positioning posts
- insulating buildings, pipes and ducting
- fitting awnings, security screens and other home improvements
- assembling and erecting scaffolding and rigging
- digging holes and trenches, and spreading, levelling and compacting soil, gravel, sand and ballast
- erecting and dismantling barricades
- directing cranes

Occupations in this minor group are classified into the following unit groups:
8211 Building and Plumbing Labourers
8212 Concreters
8213 Fencers
8214 Insulation and Home Improvement Installers
8215 Paving and Surfacing Labourers
8216 Railway Track Workers
8217 Structural Steel Construction Workers
8219 Other Construction and Mining Labourers

UNIT GROUP 8211 BUILDING AND PLUMBING LABOURERS

BUILDING AND PLUMBING LABOURERS perform a variety of routine tasks associated with erecting and repairing structures and facilities, maintaining stormwater, drainage and sewerage systems, excavating earth and clearing and levelling sites, and installing and maintaining piping systems, fixtures and water regulators.

Indicative Skill Level:
Most occupations in this unit group have a level of skill commensurate with the qualifications outlined below.

In Australia:
AQF Certificate I, or compulsory secondary education (ANZSCO Skill Level 5)

In New Zealand:
NZ Register Level 1 qualification, or compulsory secondary education (ANZSCO Skill Level 5)

For some occupations a short period of on-the-job training may be required in addition to or instead of the formal qualification. In some instances no formal qualification or on-the-job training may be required.

Tasks Include:
- loading and unloading building and construction materials, tools and equipment and transporting them around building sites
- erecting and dismantling temporary structures such as barricades and scaffolding
- mixing, pouring and spreading materials such as concrete, plaster and mortar
- cleaning and carrying out minor repairs on stormwater drains and canals, and checking for cracks and leaks in sewerage systems
- digging holes and shovelling excavated material onto conveyors, wheelbarrows and trucks for removal
- spreading and levelling soil, gravel and sand on roads and driveways, trench bottoms and similar locations
- assisting with assembling and installing piping, valves and fittings
- assisting with installing fixtures such as toilets, wash basins and sprinkler systems

Occupations:
821111 Builder's Labourer
821112 Drainage, Sewerage and Stormwater Labourer
821113 Earthmoving Labourer
821114 Plumber's Assistant

821111 BUILDER'S LABOURER

Alternative Title:
Construction Worker

Performs routine tasks in erecting and repairing structures and facilities on building and construction sites and in factories producing prefabricated building components.

Skill Level: 5

Specialisations:
Bricklayer's Assistant
Carpenter's Assistant
Tiler's Assistant

UNIT GROUP 8211 BUILDING AND PLUMBING LABOURERS *continued*

821112 DRAINAGE, SEWERAGE AND STORMWATER LABOURER

Performs routine tasks in maintaining drainage, sewerage and stormwater systems.

Skill Level: 5

821113 EARTHMOVING LABOURER

Performs routine tasks in excavating earth, clearing and levelling sites, and digging irrigation channels.

Skill Level: 5

Specialisation:
Grave Digger

821114 PLUMBER'S ASSISTANT

Performs routine tasks in fabricating, laying, installing and maintaining pipes, fixtures, water meters and regulators.

Skill Level: 5

UNIT GROUP 8212 CONCRETERS

CONCRETERS pour, spread, smooth and finish concrete for structures such as floors, stairs, ramps, footpaths and bridges.

Indicative Skill Level:
Most occupations in this unit group have a level of skill commensurate with the qualifications outlined below.

In Australia:
AQF Certificate I, or compulsory secondary education (ANZSCO Skill Level 5)

In New Zealand:
NZ Register Level 1 qualification, or compulsory secondary education (ANZSCO Skill Level 5)

For some occupations a short period of on-the-job training may be required in addition to or instead of the formal qualification. In some instances no formal qualification or on-the-job training may be required.

Tasks Include:
- erecting concrete form work and laying steel reinforcing
- pouring, spreading and levelling concrete using screeds and templates
- tamping, smoothing, shaping and sealing concrete
- operating trowelling machines to float, trowel and polish concrete surfaces
- forming expansion joints and edges using edging tools, jointers and straight edges
- installing fixtures in concrete such as anchor bolts, steel plates and door sills
- wetting concrete and rubbing with abrasives to finish vertical surfaces
- covering concrete with plastic sheeting and sand to cure it
- cutting lines in concrete using power cutters
- may cover freshly poured concrete with colouring powders and other materials

Occupation:
821211 Concreter

821211 CONCRETER

Alternative Title:
Concrete Worker

Pours, spreads, smoothes and finishes concrete for structures such as floors, stairs, ramps, footpaths and bridges.

Skill Level: 5

UNIT GROUP 8213 FENCERS

FENCERS erect and repair fences and gates.

Indicative Skill Level:
Most occupations in this unit group have a level of skill commensurate with the qualifications and experience outlined below.

In Australia:
AQF Certificate II or III (ANZSCO Skill Level 4)

In New Zealand:
NZ Register Level 2 or 3 qualification (ANZSCO Skill Level 4)

At least one year of relevant experience may substitute for the formal qualifications listed above. In some instances relevant experience and/or on-the-job training may be required in addition to the formal qualification.

Tasks Include:
- laying out fence lines and marking positions for post holes
- lifting and positioning fence posts in holes and securing posts with concrete, stone fill and soil
- forming fence frames
- constructing and attaching gates to fences
- stretching materials between fence posts
- constructing wooden paling, fibre-cement and metal fences
- repairing and demolishing existing fences

Occupation:
821311 Fencer

821311 FENCER

Alternative Title:
Fence Erector

Erects and repairs fences and gates.

Skill Level: 4

UNIT GROUP 8214 INSULATION AND HOME IMPROVEMENT INSTALLERS

INSULATION AND HOME IMPROVEMENT INSTALLERS install a variety of insulation materials to improve resistance to heat, cold, air, sound and moisture, and install functional and decorative home improvements.

Indicative Skill Level:
Most occupations in this unit group have a level of skill commensurate with the qualifications and experience outlined below.

In Australia:
AQF Certificate II or III (ANZSCO Skill Level 4)

In New Zealand:
NZ Register Level 2 or 3 qualification (ANZSCO Skill Level 4)

At least one year of relevant experience may substitute for the formal qualifications listed above. In some instances relevant experience and/or on-the-job training may be required in addition to the formal qualification.

Registration or licensing may be required.

Tasks Include:

- examining plans, specifications and work sites to determine the type and quality of installations required and their location
- preparing site for insulation and installation of fittings by nailing up furring, drilling holes for screws and bolts, and erecting scaffolding and ladders
- gluing blocks and slabs of foamed plastic and cork to walls
- operating equipment to blow and spray mineral wool, fibre fill and foam insulation material into cavities
- cutting insulation material to size and shape, and nailing and stapling batt-type insulation to joists, studs and furring
- measuring, cutting and applying solar control film to windows
- fitting awnings, security screens, shower screens, prefabricated windows and doors, exterior cladding and other home improvements using hand tools
- drilling holes in wood, brick, stone and fibrous structures, and bolting, screwing and nailing fittings into place
- attaching and adjusting mechanical fittings such as cranks, locks and pull-cords
- installing flashing and waterproofing to fittings such as shower screens and prefabricated windows and doors

Occupations:
821411 Building Insulation Installer
821412 Home Improvement Installer

821411 BUILDING INSULATION INSTALLER

Installs and applies insulating material, such as foam, granules, foil, solar film, batts and blankets, to walls, floors, windows and ceilings of buildings to insulate against heat, cold, air, sound and moisture. Registration or licensing may be required.

Skill Level: 4

Specialisation:
Window Tinter (Building)

UNIT GROUP 8214 INSULATION AND HOME IMPROVEMENT INSTALLERS *continued*

821412 HOME IMPROVEMENT INSTALLER

Installs functional and decorative home improvements such as awnings, curtains, blinds, security screens, garage doors, exterior cladding, shower screens and prefabricated windows and doors. Registration or licensing may be required.

Skill Level: 4

Specialisations:

- Awning Installer
- Carport Erector
- Curtain Fitter
- Security Door Installer
- Shower Screen Installer

UNIT GROUP 8215 PAVING AND SURFACING LABOURERS

PAVING AND SURFACING LABOURERS perform routine tasks associated in laying bituminous and other paving materials on roads, runways, parking areas and other surfaces to be paved.

Indicative Skill Level:
Most occupations in this unit group have a level of skill commensurate with the qualifications outlined below.

In Australia:
AQF Certificate I, or compulsory secondary education (ANZSCO Skill Level 5)

In New Zealand:
NZ Register Level 1 qualification, or compulsory secondary education (ANZSCO Skill Level 5)

For some occupations a short period of on-the-job training may be required in addition to or instead of the formal qualification. In some instances no formal qualification or on-the-job training may be required.

Tasks Include:

- sweeping paving bases prior to laying asphalt and other surfaces
- sprinkling and brushing hot and cold-mix asphalt over surfaces to be paved and repaired to bond asphalt toppings to bases
- operating tank-truck distributors and hoses to spray tar and road oils and emulsions on graded surfaces prior to paving
- tripping tail-gate levers to discharge hot-mix asphalt into paving machines, and spreading stone chips, gravel and cold-mix asphalt onto road surfaces
- shovelling asphalt mix into areas inaccessible to paving machines, and compacting mix using rakes and hand tampers
- cutting and trimming damaged surfaces using jack-hammers and softening edges of areas to be repaired with blowtorches
- erecting and dismantling barricades
- loading and unloading equipment, and cleaning work sites
- may direct traffic

Occupation:
821511 Paving and Surfacing Labourer

821511 PAVING AND SURFACING LABOURER

Performs routine tasks associated in laying bituminous and other paving materials on roads, runways, parking areas and other surfaces to be paved.

Skill Level: 5

UNIT GROUP 8216 RAILWAY TRACK WORKERS

RAILWAY TRACK WORKERS lay and repair tracks for railways, tramways, quarries and mines, and install and repair signals and other equipment.

Indicative Skill Level:
Most occupations in this unit group have a level of skill commensurate with the qualifications and experience outlined below.

In Australia:
AQF Certificate II or III (ANZSCO Skill Level 4)

In New Zealand:
NZ Register Level 2 or 3 qualification (ANZSCO Skill Level 4)

At least one year of relevant experience may substitute for the formal qualifications listed above. In some instances relevant experience and/or on-the-job training may be required in addition to the formal qualification.

Tasks Include:
- spreading and tamping ballast to provide firm foundation for sleepers
- cutting rails to length and grinding worn and rough rail ends
- placing sleepers across roadbeds, and positioning and fastening rails on sleepers
- drilling bolt holes, and bolting and welding rail sections
- removing and replacing worn and damaged rails, sleepers and switches
- cleaning and lubricating switches
- examining track, lubricating wheel bearings on rolling stock and maintaining switch signal lamps
- installing and repairing signals and other equipment
- may assist with the righting of derailed rolling stock

Occupation:
821611 Railway Track Worker

821611 RAILWAY TRACK WORKER

Alternative Title:
Railway Fettler

Lays and repairs tracks for railways, tramways, quarries and mines, and installs and repairs signals and other equipment.

Skill Level: 4

Specialisation:
Track Inspector

UNIT GROUP 8217 STRUCTURAL STEEL CONSTRUCTION WORKERS

STRUCTURAL STEEL CONSTRUCTION WORKERS assemble rigging gear to move and position equipment and structural components, erect scaffolding, position and secure steel reinforcing in concrete forms, and erect and dismantle structural steel frames.

Indicative Skill Level:
Most occupations in this unit group have a level of skill commensurate with the qualifications and experience outlined below.

In Australia:
AQF Certificate II or III (ANZSCO Skill Level 4)

In New Zealand:
NZ Register Level 2 or 3 qualification (ANZSCO Skill Level 4)

At least one year of relevant experience may substitute for the formal qualifications listed above. In some instances relevant experience and/or on-the-job training may be required in addition to the formal qualification.

Registration or licensing may be required.

Tasks Include:
- erecting lifting tackles by attaching pulleys and blocks to fixed overhead structures, and installing cables and attaching counterweights
- attaching slinging gear to hoisting equipment and objects to be moved using clamps, hooks, bolts and knots
- fitting and bolting tubes, support braces and components to form bases and build up scaffolding
- lifting and positioning sections of scaffolding
- measuring, cutting, bending and fitting welded wire mesh into concrete areas to be mesh-reinforced
- fixing mesh and reinforcing steel into position in formwork for concrete pours
- setting up winches and rigging equipment to raise and position girders, plates, columns and other steel units
- erecting guard rails, guy wires, ropes and clears, laying planks and hanging safety nets

Occupations:
821711 Construction Rigger
821712 Scaffolder
821713 Steel Fixer
821714 Structural Steel Erector

821711 CONSTRUCTION RIGGER

Assembles and installs rigging gear, such as cables, ropes, pulleys and winches, to lift, lower, move and position equipment, structural steel and other heavy objects. Registration or licensing may be required.

Skill Level: 4

821712 SCAFFOLDER

Erects and dismantles scaffolding to provide work platforms on building and industrial sites, and for temporary structures such as staging and seating. Registration or licensing may be required.

Skill Level: 4

UNIT GROUP 8217 STRUCTURAL STEEL CONSTRUCTION WORKERS *continued*

821713 STEEL FIXER

Positions and secures steel bars and steel mesh in concrete forms to reinforce concrete structures. Registration or licensing may be required.

Skill Level: 4

821714 STRUCTURAL STEEL ERECTOR

Erects and dismantles structural steel frames of buildings and other structures. Registration or licensing may be required.

Skill Level: 4

UNIT GROUP 8219 OTHER CONSTRUCTION AND MINING LABOURERS

This unit group covers Construction and Mining Labourers not elsewhere classified.

It includes Crane Chasers, Driller's Assistants, Laggers, Mining Support Workers and Surveyor's Assistants.

Indicative Skill Level:
Most occupations in this unit group have a level of skill commensurate with the qualifications outlined below.

In Australia:
AQF Certificate I, or compulsory secondary education (ANZSCO Skill Level 5)

In New Zealand:
NZ Register Level 1 qualification, or compulsory secondary education (ANZSCO Skill Level 5)

For some occupations a short period of on-the-job training may be required in addition to or instead of the formal qualification. In some instances no formal qualification or on-the-job training may be required.

Occupations:
- 821911 Crane Chaser
- 821912 Driller's Assistant
- 821913 Lagger
- 821914 Mining Support Worker
- 821915 Surveyor's Assistant

821911 CRANE CHASER

Slings cranes and winches, and directs the movement of loads ensuring loads do not exceed lifting capacities.

Skill Level: 5

Specialisations:
- Dogman/woman
- Slinger

821912 DRILLER'S ASSISTANT

Alternative Titles:
- Driller's Offsider
- Roustabout (Oil and Gas)

Performs routine tasks in setting up, operating and dismantling drilling sites for extracting oil, gas, mineral ore or water.

Skill Level: 5

Specialisations:
- Derrick Hand
- Roughneck
- Well Treatment Offsider

821913 LAGGER

Applies insulating materials, such as felt, fibreglass, polyurethane and cork, to pipes, steam generators, process vats and ducting, and secures insulation with wire, wire netting, staples, metal strapping and using welding torches.

Skill Level: 5

UNIT GROUP 8219 OTHER CONSTRUCTION AND MINING LABOURERS *continued*

821914 MINING SUPPORT WORKER

Alternative Title:

Mineral Ore Processing Labourer

Performs routine tasks in mining and mineral ore treating operations such as assembling, operating and dismantling mining equipment, taking ore, rock and dust samples, and mixing ore treating chemicals and catalysts.

Skill Level: 5

Specialisations:

Pit Crew Support Worker
Wash Plant Attendant

821915 SURVEYOR'S ASSISTANT

Performs routine tasks to assist Surveyors and Geologists by transporting, assembling, maintaining and laying out prospecting and surveying equipment, and collecting and labelling samples.

Skill Level: 5

Specialisations:

Geological Survey Field Assistant
Seismic Survey Assistant

SUB-MAJOR GROUP 83 FACTORY PROCESS WORKERS

FACTORY PROCESS WORKERS perform routine tasks in processing, manufacturing and packaging food, beverages and other products.

Indicative Skill Level:
Most occupations in this sub-major group have a level of skill commensurate with the qualifications and experience outlined below.

In Australia:
AQF Certificate II or III, or at least one year of relevant experience (ANZSCO Skill Level 4); or
AQF Certificate I, or compulsory secondary education (ANZSCO Skill Level 5)

In New Zealand:
NZ Register Level 2 or 3 qualification, or at least one year of relevant experience (ANZSCO Skill Level 4); or
NZ Register Level 1 qualification, or compulsory secondary education (ANZSCO Skill Level 5)

In some instances relevant experience and/or on-the-job training may be required in addition to the formal qualification. In the case of some Skill Level 5 occupations, a short period of on-the-job training may be required in addition to or instead of the formal qualification, or no formal qualification or on-the-job training may be required.

Tasks Include:
- sourcing, weighing and mixing raw materials, and loading into machines
- wrapping products, and filling, labelling and sealing containers by hand and machine
- storing and stacking finished products, and cleaning machines and work areas
- inspecting and grading products
- assembling components and subassemblies for further processing and to make finished products

Occupations in this sub-major group are classified into the following minor groups:
831 Food Process Workers
832 Packers and Product Assemblers
839 Miscellaneous Factory Process Workers

MINOR GROUP 831 FOOD PROCESS WORKERS

FOOD PROCESS WORKERS perform routine tasks in processing and manufacturing food and beverages.

Indicative Skill Level:
Most occupations in this minor group have a level of skill commensurate with the qualifications and experience outlined below.

In Australia:
AQF Certificate II or III, or at least one year of relevant experience (ANZSCO Skill Level 4); or
AQF Certificate I, or compulsory secondary education (ANZSCO Skill Level 5)

In New Zealand:
NZ Register Level 2 or 3 qualification, or at least one year of relevant experience (ANZSCO Skill Level 4); or
NZ Register Level 1 qualification, or compulsory secondary education (ANZSCO Skill Level 5)

In some instances relevant experience and/or on-the-job training may be required in addition to the formal qualification. In the case of some Skill Level 5 occupations, a short period of on-the-job training may be required in addition to or instead of the formal qualification, or no formal qualification or on-the-job training may be required.

Tasks Include:

- measuring, weighing and mixing ingredients and loading into food and beverage machines
- operating machines to process and produce food and beverage products
- cutting, trimming and dressing meat, poultry and seafood
- checking product quality before packaging
- packing finished product
- cleaning machines, storage tanks and floors, and maintaining infestation control programs

Occupations in this minor group are classified into the following unit groups:
8311 Food and Drink Factory Workers
8312 Meat Boners and Slicers, and Slaughterers
8313 Meat, Poultry and Seafood Process Workers

UNIT GROUP 8311 FOOD AND DRINK FACTORY WORKERS

FOOD AND DRINK FACTORY WORKERS perform routine tasks in manufacturing food and beverages.

Indicative Skill Level:
Most occupations in this unit group have a level of skill commensurate with the qualifications outlined below.

In Australia:
AQF Certificate I, or compulsory secondary education (ANZSCO Skill Level 5)

In New Zealand:
NZ Register Level 1 qualification, or compulsory secondary education (ANZSCO Skill Level 5)

For some occupations a short period of on-the-job training may be required in addition to or instead of the formal qualification. In some instances no formal qualification or on-the-job training may be required.

Tasks Include:
- weighing, measuring, mixing, dissolving and boiling ingredients
- adding materials, such as spices and preservatives, to food and beverages
- operating heating, chilling, freezing, pasteurising, carbonating, sulphuring and desulphuring plant
- monitoring product quality before packaging by inspecting, taking samples and adjusting treatment conditions when necessary
- operating machines to peel, core, slice, dice, pit and juice fruit and vegetables
- cleaning equipment, pumps, hoses, storage tanks, vessels and floors, and maintaining infestation control programs
- regulating speed of conveyors and crusher rollers, and adjusting tension of rollers to ensure total extraction of juice from sugar cane
- moving products from production lines into storage and shipping areas
- packaging and bottling products

Occupations:
831111 Baking Factory Worker
831112 Brewery Worker
831113 Confectionery Maker
831114 Dairy Products Maker
831115 Fruit and Vegetable Factory Worker
831116 Grain Mill Worker
831117 Sugar Mill Worker
831118 Winery Cellar Hand
831199 Food and Drink Factory Workers nec

831111 BAKING FACTORY WORKER

Operates machines and performs routine tasks to make bread, cakes and other baked products, and slices and wraps products.

Skill Level: 5

Specialisations:
Biscuit Factory Worker
Bread Room Hand

UNIT GROUP 8311 FOOD AND DRINK FACTORY WORKERS *continued*

831112 BREWERY WORKER

Operates machines and performs routine tasks to make beer, and package, store and despatch beer in bottles, cans and kegs.

Skill Level: 5

831113 CONFECTIONERY MAKER

Operates machines and performs routine tasks to make and wrap confectionery.

Skill Level: 5

Specialisation:
- Chocolate Maker

831114 DAIRY PRODUCTS MAKER

Operates machines and performs routine tasks to make and package milk, milk powder, yoghurt, butter, cheese and other dairy products.

Skill Level: 5

Specialisations:
- Butter Maker
- Cheese Factory Worker
- Cheese Maker
- Milk Processing Worker
- Milk Treater
- Pasteuriser Operator
- Yoghurt Maker

831115 FRUIT AND VEGETABLE FACTORY WORKER

Operates machines and performs routine tasks to prepare canned and frozen fruit and vegetables, and make and package sauces, jams and juices.

Skill Level: 5

831116 GRAIN MILL WORKER

Operates machines and performs routine tasks to mix, mill and treat grains and by-products to make flour, meal and stockfeed.

Skill Level: 5

Specialisation:
- Stockfeed Miller

831117 SUGAR MILL WORKER

Operates machines and performs routine tasks to extract juice from sugar cane to make granular sugar and molasses.

Skill Level: 5

UNIT GROUP 8311 FOOD AND DRINK FACTORY WORKERS *continued*

831118 WINERY CELLAR HAND

Operates machines and performs routine tasks to make and bottle wine.

Skill Level: 5

831199 FOOD AND DRINK FACTORY WORKERS NEC

This occupation group covers Food and Drink Factory Workers not elsewhere classified.

Skill Level: 5

Occupations in this group include:

- Egg Factory Worker
- Ice-cream Maker
- Margarine Maker

UNIT GROUP 8312 MEAT BONERS AND SLICERS, AND SLAUGHTERERS

MEAT BONERS AND SLICERS, AND SLAUGHTERERS trim and cut meat from bones, sides and carcasses, and slaughter livestock in abattoirs.

Indicative Skill Level:
Most occupations in this unit group have a level of skill commensurate with the qualifications and experience outlined below.

In Australia:
AQF Certificate II or III (ANZSCO Skill Level 4)

In New Zealand:
NZ Register Level 2 or 3 qualification (ANZSCO Skill Level 4)

At least one year of relevant experience may substitute for the formal qualifications listed above. In some instances relevant experience and/or on-the-job training may be required in addition to the formal qualification.

Tasks Include:
- operating switching controls to direct and drop carcasses and meat cuts from supply rails to boning tables
- cutting meat to separate meat, fat and tissue from around bones
- washing, scraping and trimming foreign material and blood from meat
- cutting sides and quarters of meat into standard meat cuts, such as rumps, flanks and shoulders, and removing internal fat, blood clots, bruises and other matter to prepare them for packing and marketing
- operating restrainer and stunning equipment
- severing jugular veins of stunned animals to drain blood and facilitate dressing
- trimming and removing head meat and severing animal heads
- slitting open, eviscerating and trimming animal carcasses
- may slaughter livestock according to procedures required by religious customs

Occupations:
831211 Meat Boner and Slicer
831212 Slaughterer

831211 MEAT BONER AND SLICER

Trims and cuts meat from bones, sides and carcasses.

Skill Level: 4

Specialisation:
Meat Trimmer

831212 SLAUGHTERER

Stuns and kills livestock, and prepares carcasses for further processing by removing internal organs and hides.

Skill Level: 4

Specialisation:
Stunner and Shackler (Abattoir)

UNIT GROUP 8313 MEAT, POULTRY AND SEAFOOD PROCESS WORKERS

MEAT, POULTRY AND SEAFOOD PROCESS WORKERS slaughter and eviscerate poultry, and process, grade and package meat, poultry, fish and shellfish.

Indicative Skill Level:
Most occupations in this unit group have a level of skill commensurate with the qualifications outlined below.

In Australia:
AQF Certificate I, or compulsory secondary education (ANZSCO Skill Level 5)

In New Zealand:
NZ Register Level 1 qualification, or compulsory secondary education (ANZSCO Skill Level 5)

For some occupations a short period of on-the-job training may be required in addition to or instead of the formal qualification. In some instances no formal qualification or on-the-job training may be required.

Tasks Include:
- processing offal and tripe
- moving carcasses to chillers and freezers
- loading meat products into trucks
- packing boned and sliced meat into cartons
- stunning and shackling poultry for killing and processing
- severing jugular veins of poultry, and removing viscera and residual material from poultry carcasses
- separating organs and glands, such as sweetbreads, livers, hearts and spleens, from poultry carcasses
- inspecting and grading poultry, fish and shellfish for size and quality
- packing fish and counting packs before freezing, and packing frozen fish blocks into cartons
- operating machines which slice, peel, skin and crumb fish
- cleaning and sanitising equipment and work areas

Occupations:
831311 Meat Process Worker
831312 Poultry Process Worker
831313 Seafood Process Worker

831311 MEAT PROCESS WORKER

Processes carcasses of slaughtered livestock and prepares meat and meat products.

Skill Level: 5

Specialisation:
Offal Separator

831312 POULTRY PROCESS WORKER

Stuns and kills, dresses, trims, cuts into portions, bones, fillets, weighs, grades and packages poultry.

Skill Level: 5

Specialisations:
Poultry Boner
Poultry Slaughterer

UNIT GROUP 8313 MEAT, POULTRY AND SEAFOOD PROCESS WORKERS *continued*

831313 SEAFOOD PROCESS WORKER

Scales, cleans, fillets, cuts, shells, grades and packages fish and shellfish.

Skill Level: 5

Specialisations:
- Abalone Sheller
- Oyster Opener

MINOR GROUP 832 PACKERS AND PRODUCT ASSEMBLERS

PACKERS AND PRODUCT ASSEMBLERS wrap and place items into containers and seal containers in preparation for despatch to customers, and assemble components and subassemblies of products.

Indicative Skill Level:
Most occupations in this minor group have a level of skill commensurate with the qualifications outlined below.

In Australia:
AQF Certificate I, or compulsory secondary education (ANZSCO Skill Level 5)

In New Zealand:
NZ Register Level 1 qualification, or compulsory secondary education (ANZSCO Skill Level 5)

For some occupations a short period of on-the-job training may be required in addition to or instead of the formal qualification. In some instances no formal qualification or on-the-job training may be required.

Tasks Include:
- filling and packing products into containers
- labelling and wrapping finished products
- monitoring weight and quantity of products
- locating, positioning and securing components on workbenches
- assembling and securing components in sequence
- assembling parts, deburring and finishing items and fitting hardware

Occupations in this minor group are classified into the following unit groups:
8321 Packers
8322 Product Assemblers

UNIT GROUP 8321 PACKERS

PACKERS weigh, wrap, seal and label chocolate, fruit, vegetables, meat, seafood and other products.

Indicative Skill Level:
Most occupations in this unit group have a level of skill commensurate with the qualifications outlined below.

In Australia:
AQF Certificate I, or compulsory secondary education (ANZSCO Skill Level 5)

In New Zealand:
NZ Register Level 1 qualification, or compulsory secondary education (ANZSCO Skill Level 5)

For some occupations a short period of on-the-job training may be required in addition to or instead of the formal qualification. In some instances no formal qualification or on-the-job training may be required.

Tasks Include:
- obtaining supplies of products and assembling bags, package folders and cartons
- packing containers and bags with products, and counting, weighing and measuring amounts and adjusting quantities
- wrapping protective material around products, sealing bags and containers, and attaching pre-printed labels
- counting and placing bags and packages onto trays and racks, and into shipping cartons
- recording information such as numbers, weight, times and dates
- monitoring the filling of containers and adjusting machines to maintain volume and seal quality
- monitoring the supply and quality of containers and contents of holding tanks
- checking the cleanliness and operation of machines, equipment and containers

Occupations:
832111 Chocolate Packer
832112 Container Filler
832113 Fruit and Vegetable Packer
832114 Meat Packer
832115 Seafood Packer
832199 Packers nec

832111 CHOCOLATE PACKER

Weighs, wraps, seals and labels chocolate products.

Skill Level: 5

832112 CONTAINER FILLER

Fills and seals containers with products, such as food, beverages, paints, oils and lotions, and packages the filled containers.

Skill Level: 5

Specialisations:
Bottling Attendant
Cannery Worker
Labelling Machine Operator

UNIT GROUP 8321 PACKERS *continued*

832113 FRUIT AND VEGETABLE PACKER

Weighs, wraps, seals and labels fruit and vegetables.

Skill Level: 5

832114 MEAT PACKER

Weighs, wraps, seals and labels meat and meat products.

Skill Level: 5

832115 SEAFOOD PACKER

Weighs, wraps, seals and labels seafood products.

Skill Level: 5

832199 PACKERS NEC

This occupation group covers Packers not elsewhere classified.

Skill Level: 5

Occupations in this group include:

- Cheese Packer
- Egg Packer

UNIT GROUP 8322 PRODUCT ASSEMBLERS

PRODUCT ASSEMBLERS put together components and subassemblies that go into the production of metal products, electrical and electronic equipment, jewellery and precious metal articles, and joinery products.

Indicative Skill Level:
Most occupations in this unit group have a level of skill commensurate with the qualifications outlined below.

In Australia:
AQF Certificate I, or compulsory secondary education (ANZSCO Skill Level 5)

In New Zealand:
NZ Register Level 1 qualification, or compulsory secondary education (ANZSCO Skill Level 5)

For some occupations a short period of on-the-job training may be required in addition to or instead of the formal qualification. In some instances no formal qualification or on-the-job training may be required.

Tasks Include:
- locating, positioning and securing components on workbenches
- punching and drilling mounting holes in parts and assembled products
- assembling and securing components in sequence
- assembling parts by nailing, screwing, gluing and dowelling, riveting, crimping, soldering and spot welding components
- fitting hardware items, such as hinges, catches and knobs, to parts
- attaching and fastening jewellery and jewellery parts to fabricate bracelets, necklaces, brooches and earrings
- deburring and finishing items using files, grinding wheels and emery paper
- may manually wind light electrical field coils

Occupation:
832211 Product Assembler

832211 PRODUCT ASSEMBLER

Puts together components and subassemblies that go into the production of metal products, electrical and electronic equipment, jewellery and precious metal articles, and joinery products.

Skill Level: 5

Specialisations:
Electrical and Electronic Assembler
Light Coil Winder
Vehicle Assembler

MINOR GROUP 839 MISCELLANEOUS FACTORY PROCESS WORKERS

This minor group covers Factory Process Workers not elsewhere classified.

It includes Metal Engineering Process Workers, Plastics and Rubber Factory Workers, Product Quality Controllers, and Timber and Wood Process Workers.

Indicative Skill Level:
Most occupations in this minor group have a level of skill commensurate with the qualifications and experience outlined below.

In Australia:
AQF Certificate II or III, or at least one year of relevant experience (ANZSCO Skill Level 4); or
AQF Certificate I, or compulsory secondary education (ANZSCO Skill Level 5)

In New Zealand:
NZ Register Level 2 or 3 qualification, or at least one year of relevant experience (ANZSCO Skill Level 4); or
NZ Register Level 1 qualification, or compulsory secondary education (ANZSCO Skill Level 5)

In some instances relevant experience and/or on-the-job training may be required in addition to the formal qualification. In the case of some Skill Level 5 occupations, a short period of on-the-job training may be required in addition to or instead of the formal qualification, or no formal qualification or on-the-job training may be required.

Occupations in this minor group are classified into the following unit groups:
8391 Metal Engineering Process Workers
8392 Plastics and Rubber Factory Workers
8393 Product Quality Controllers
8394 Timber and Wood Process Workers
8399 Other Factory Process Workers

UNIT GROUP 8391 METAL ENGINEERING PROCESS WORKERS

METAL ENGINEERING PROCESS WORKERS perform routine tasks in manufacturing metal products.

Indicative Skill Level:
Most occupations in this unit group have a level of skill commensurate with the qualifications outlined below.

In Australia:
AQF Certificate I, or compulsory secondary education (ANZSCO Skill Level 5)

In New Zealand:
NZ Register Level 1 qualification, or compulsory secondary education (ANZSCO Skill Level 5)

For some occupations a short period of on-the-job training may be required in addition to or instead of the formal qualification. In some instances no formal qualification or on-the-job training may be required.

Tasks Include:
- positioning and holding tools and metal products
- performing assembly and dismantling operations such as screwing and bolting
- operating power hammers, presses and other metal cutting and shaping tools and machines
- soldering and spot welding components using electrical spot and butt welding machines
- transporting tools, materials and work pieces to and from sites and workbenches
- cleaning and preparing working surfaces

Occupation:
839111 Metal Engineering Process Worker

839111 METAL ENGINEERING PROCESS WORKER

Performs routine tasks in manufacturing metal products.

Skill Level: 5

Specialisations:
Boilermaker's Assistant
Fitter's Assistant
Metal Forger's Assistant
Metal Moulder's Assistant

UNIT GROUP 8392 PLASTICS AND RUBBER FACTORY WORKERS

PLASTICS AND RUBBER FACTORY WORKERS perform routine tasks in manufacturing plastic and rubber products.

Indicative Skill Level:
Most occupations in this unit group have a level of skill commensurate with the qualifications outlined below.

In Australia:
AQF Certificate I, or compulsory secondary education (ANZSCO Skill Level 5)

In New Zealand:
NZ Register Level 1 qualification, or compulsory secondary education (ANZSCO Skill Level 5)

For some occupations a short period of on-the-job training may be required in addition to or instead of the formal qualification. In some instances no formal qualification or on-the-job training may be required.

Tasks Include:
- dumping material into hoppers of machines
- stopping moulding machines and discharging contents
- cutting foam products from foam blocks
- cleaning, smoothing and waxing moulds for making products
- brushing and spraying release agents onto moulds to assist with the removal of moulded products
- building up layers of fibreglass and resin on moulds
- cleaning work areas, tools and equipment
- may smooth rough edges of moulds using files, grinders and sanders

Occupations:
839211 Plastics Factory Worker
839212 Rubber Factory Worker

839211 PLASTICS FACTORY WORKER

Alternative Title:
Plastics Process Hand

Performs routine tasks in manufacturing plastic goods.

Skill Level: 5

Specialisations:
Cellular Plastics Cutter
Fibreglass Lay Up Worker

839212 RUBBER FACTORY WORKER

Alternative Title:
Rubber Process Hand

Performs routine tasks in manufacturing tyres and other rubber products.

Skill Level: 5

Specialisation:
Latex Foam Worker

UNIT GROUP 8393 PRODUCT QUALITY CONTROLLERS

PRODUCT QUALITY CONTROLLERS examine manufactured products and primary produce to ensure conformity to specifications and standards of presentation and quality.

Indicative Skill Level:
Most occupations in this unit group have a level of skill commensurate with the qualifications and experience outlined below.

In Australia:
AQF Certificate II or III (ANZSCO Skill Level 4)

In New Zealand:
NZ Register Level 2 or 3 qualification (ANZSCO Skill Level 4)

At least one year of relevant experience may substitute for the formal qualifications listed above. In some instances relevant experience and/or on-the-job training may be required in addition to the formal qualification.

Tasks Include:
- studying product specifications and taking measurements to determine conformity to specifications
- examining and marking output for visible defects such as cracks, holes and breakages
- making minor repairs and adjustments to products
- compiling quality assurance reports, maintaining documentation and reporting findings
- examining products for defects and grading produce
- designating grading of produce and recording details of assessments according to classification system
- collecting and labelling samples for inspection
- recording details of sampling procedures and sources of samples
- preparing samples and carrying out prescribed tests

Occupations:
839311 Product Examiner
839312 Product Grader
839313 Product Tester

839311 PRODUCT EXAMINER

Alternative Titles:
Quality Assurance Assessor
Quality Control Assessor

Examines products to ensure conformity to specifications and standards of presentation and quality.

Skill Level: 4

Specialisations:
Film Examiner
Metal Products Viewer
Textile Examiner
Tyre Finisher and Examiner
Vehicle Assembly Inspector

UNIT GROUP 8393 PRODUCT QUALITY CONTROLLERS *continued*

839312 PRODUCT GRADER

Grades primary produce by evaluating individual items or batches against established standards and records results.

Skill Level: 4

Specialisations:
- Fruit and Vegetable Classer
- Meat Grader
- Milk and Cream Grader
- Timber Grader

839313 PRODUCT TESTER

Collects product samples, conducts tests to determine quality of produce and maintains records of results.

Skill Level: 4

Specialisations:
- Coal Sample Tester
- Glassware Verifier
- Iron Pellet Tester

UNIT GROUP 8394 TIMBER AND WOOD PROCESS WORKERS

TIMBER AND WOOD PROCESS WORKERS perform routine tasks in paper and pulp mills, sawmills, timber yards, and wood processing and timber products factories.

Indicative Skill Level:
Most occupations in this unit group have a level of skill commensurate with the qualifications outlined below.

In Australia:
AQF Certificate I, or compulsory secondary education (ANZSCO Skill Level 5)

In New Zealand:
NZ Register Level 1 qualification, or compulsory secondary education (ANZSCO Skill Level 5)

For some occupations a short period of on-the-job training may be required in addition to or instead of the formal qualification. In some instances no formal qualification or on-the-job training may be required.

Tasks Include:
- rolling logs from trucks and conveyors to log decks, saw carriages and stacking bays
- placing logs and wood billets onto conveyors and lathes for processing into chips, veneers and pulp
- sorting and stacking timber during milling
- placing timber for processing by machines and unloading cut timber from tail end of machines
- assisting with setting up and operating plant and ancillary equipment used in the manufacture of sheets and boards
- transporting processed wood products, such as plywood, chipboard sheets and panels, to work areas
- clearing blockages in machines
- assisting with measuring and cutting materials
- packing and loading finished products for transportation
- cleaning work areas, tools and equipment

Occupations:
839411 Paper and Pulp Mill Worker
839412 Sawmill or Timber Yard Worker
839413 Wood and Wood Products Factory Worker

839411 PAPER AND PULP MILL WORKER

Alternative Title:
Pulp, Paper Making and Paper Products Labourer

Performs routine tasks in a paper and pulp mill such as placing logs onto conveyors for chipping, and loading woodchip and pulp for processing.

Skill Level: 5

UNIT GROUP 8394 TIMBER AND WOOD PROCESS WORKERS *continued*

839412 SAWMILL OR TIMBER YARD WORKER

Alternative Titles:

Timber Mill Worker
Wood Processing Worker

Performs routine tasks in a sawmill or timber yard such as sorting and stacking timber, assisting timber machinists, assembling orders and racking offcuts.

Skill Level: 5

Specialisation:

Tailer-out

839413 WOOD AND WOOD PRODUCTS FACTORY WORKER

Alternative Title:

Wood and Wood Products Labourer

Performs routine tasks in a wood processing and timber product factory such as placing logs on equipment and conveyors, assisting with measuring and cutting of materials, and setting up and operating plant equipment.

Skill Level: 5

Specialisations:

Hardboard Factory Worker
Joinery Factory Worker
Particleboard Factory Worker
Plywood Factory Worker

UNIT GROUP 8399 OTHER FACTORY PROCESS WORKERS

This unit group covers Factory Process Workers not elsewhere classified.

It includes Cement and Concrete Plant Workers, Chemical Plant Workers, Clay Processing Factory Workers, Fabric and Textile Factory Workers, Footwear Factory Workers, Glass Processing Workers, and Hide and Skin Processing Workers.

Indicative Skill Level:
Most occupations in this unit group have a level of skill commensurate with the qualifications outlined below.

In Australia:
AQF Certificate I, or compulsory secondary education (ANZSCO Skill Level 5)

In New Zealand:
NZ Register Level 1 qualification, or compulsory secondary education (ANZSCO Skill Level 5)

For some occupations a short period of on-the-job training may be required in addition to or instead of the formal qualification. In some instances no formal qualification or on-the-job training may be required.

Occupations:
839911 Cement and Concrete Plant Worker
839912 Chemical Plant Worker
839913 Clay Processing Factory Worker
839914 Fabric and Textile Factory Worker
839915 Footwear Factory Worker
839916 Glass Processing Worker
839917 Hide and Skin Processing Worker
839999 Factory Process Workers nec

839911 CEMENT AND CONCRETE PLANT WORKER

Performs routine tasks in manufacturing cement and concrete products such as greasing and assembling concrete moulds, holding reinforcing steel in position during concrete pours, stripping moulds from dried concrete products, and finishing products.

Skill Level: 5

839912 CHEMICAL PLANT WORKER

Performs routine tasks in a chemical processing plant such as delivering materials to processing areas, dumping ingredients into hoppers, operating machines to heat, cool and agitate chemical solutions, filling and fastening covers on containers, and attaching labels and information on products.

Skill Level: 5

Specialisations:
Gas Plant Worker
Munitions Factory Worker
Paint Factory Worker

UNIT GROUP 8399 OTHER FACTORY PROCESS WORKERS *continued*

839913 CLAY PROCESSING FACTORY WORKER

Alternative Title:

Clay Processing Labourer

Performs routine tasks in manufacturing clay and ceramic products such as loading clay into machines, stacking products on kiln cars, pallets and trolleys, and moving kiln cars and trolleys to and from kilns, dryers, sorting, storage and shipping areas.

Skill Level: 5

Specialisations:

Brick Handler
Carousel Minder
Kiln Labourer

839914 FABRIC AND TEXTILE FACTORY WORKER

Performs routine tasks in a fabric and textile factory such as cutting canvas, upholstery and curtain fabrics, delivering materials to machines, operating automatic machines using computerised patterns, pressing partially completed and finished garments, and inspecting and finishing completed garments.

Skill Level: 5

839915 FOOTWEAR FACTORY WORKER

Performs routine tasks in manufacturing footwear such as basic hand cutting of shoe components, delivering materials to machines, and inspecting and finishing completed footwear.

Skill Level: 5

839916 GLASS PROCESSING WORKER

Performs routine tasks in manufacturing glassware such as setting up, adjusting and repairing automatic machines and equipment, and checking weight of glassware.

Skill Level: 5

Specialisation:

Glass Mould Cleaner

839917 HIDE AND SKIN PROCESSING WORKER

Performs routine tasks in tanning and finishing leather, hides and skins such as fleshing hides by cutting out pieces of flesh and fat, laying out hides and skins for classing and drying and arranging heaters to dry them, spraying dried hides with preservatives, and treating, pressing and securing hides and skins.

Skill Level: 5

Specialisations:

Fellmongery Worker
Hand Flesher
Tannery Worker

UNIT GROUP 8399 OTHER FACTORY PROCESS WORKERS *continued*

839999 FACTORY PROCESS WORKERS NEC

This occupation group covers Factory Process Workers not elsewhere classified.

Skill Level: 5

Occupations in this group include:

Sheltered Workshop Worker

SUB-MAJOR GROUP 84 FARM, FORESTRY AND GARDEN WORKERS

FARM, FORESTRY AND GARDEN WORKERS perform a variety of routine tasks in cultivating and harvesting crops, plants and forests, breeding and raising of livestock and aquatic stock, and the management of pests and weeds.

Indicative Skill Level:
Most occupations in this sub-major group have a level of skill commensurate with the qualifications and experience outlined below.

In Australia:
AQF Certificate II or III, or at least one year of relevant experience (ANZSCO Skill Level 4); or
AQF Certificate I, or compulsory secondary education (ANZSCO Skill Level 5)

In New Zealand:
NZ Register Level 2 or 3 qualification, or at least one year of relevant experience (ANZSCO Skill Level 4); or
NZ Register Level 1 qualification, or compulsory secondary education (ANZSCO Skill Level 5)

In some instances relevant experience and/or on-the-job training may be required in addition to the formal qualification. In the case of some Skill Level 5 occupations, a short period of on-the-job training may be required in addition to or instead of the formal qualification, or no formal qualification or on-the-job training may be required.

Tasks Include:
- planting and propagating trees, seeds, seedlings, bulbs and vines
- pruning and thinning crops, vines and trees
- monitoring the condition of crops and livestock and ensuring that they have adequate water and feed
- harvesting, sorting and packing produce into containers
- mustering and droving livestock
- cultivating and maintaining natural and plantation forests and felling trees
- managing and controlling pests and weeds species by applying chemicals and trapping and shooting animals

Occupations in this sub-major group are classified into the following minor group:
841 Farm, Forestry and Garden Workers

MINOR GROUP 841 FARM, FORESTRY AND GARDEN WORKERS

FARM, FORESTRY AND GARDEN WORKERS perform a variety of routine tasks in cultivating and harvesting crops, plants and forests, breeding and raising of livestock and aquatic stock, and the management of pests and weeds.

Indicative Skill Level:
Most occupations in this minor group have a level of skill commensurate with the qualifications and experience outlined below.

In Australia:
AQF Certificate II or III, or at least one year of relevant experience (ANZSCO Skill Level 4); or
AQF Certificate I, or compulsory secondary education (ANZSCO Skill Level 5)

In New Zealand:
NZ Register Level 2 or 3 qualification, or at least one year of relevant experience (ANZSCO Skill Level 4); or
NZ Register Level 1 qualification, or compulsory secondary education (ANZSCO Skill Level 5)

In some instances relevant experience and/or on-the-job training may be required in addition to the formal qualification. In the case of some Skill Level 5 occupations, a short period of on-the-job training may be required in addition to or instead of the formal qualification, or no formal qualification or on-the-job training may be required.

Tasks Include:
- planting and propagating trees, seeds, seedlings, bulbs and vines
- pruning and thinning crops, vines and trees
- monitoring the condition of crops and livestock and ensuring that they have adequate water and feed
- harvesting, sorting and packing produce into containers
- mustering and droving livestock
- cultivating and maintaining natural and plantation forests and felling trees
- managing and controlling pests and weeds species by applying chemicals and trapping and shooting animals

Occupations in this minor group are classified into the following unit groups:
8411 Aquaculture Workers
8412 Crop Farm Workers
8413 Forestry and Logging Workers
8414 Garden and Nursery Labourers
8415 Livestock Farm Workers
8416 Mixed Crop and Livestock Farm Workers
8419 Other Farm, Forestry and Garden Workers nec

UNIT GROUP 8411 AQUACULTURE WORKERS

AQUACULTURE WORKERS perform routine tasks in breeding and raising fish and other aquatic stock.

Indicative Skill Level:
Most occupations in this unit group have a level of skill commensurate with the qualifications outlined below.

In Australia:
AQF Certificate I, or compulsory secondary education (ANZSCO Skill Level 5)

In New Zealand:
NZ Register Level 1 qualification, or compulsory secondary education (ANZSCO Skill Level 5)

For some occupations a short period of on-the-job training may be required in addition to or instead of the formal qualification. In some instances no formal qualification or on-the-job training may be required.

Tasks Include:
- feeding and grading fish, and monitoring their growth
- assisting with farm layout and constructing nets, long-lines and cages
- checking and looking after equipment and fish housing
- operating pumps and other equipment
- testing and checking on water quality
- removing dead and dying fish
- operating lifting equipment such as forklifts and small cranes
- harvesting fish, and sorting and packing for transportation
- restocking pens, pools, tanks, ponds, rivers and dams with juvenile fish
- collecting and recording growth, production and water quality data

Occupation:
841111 Aquaculture Worker

841111 AQUACULTURE WORKER

Performs routine tasks in breeding and raising fish and other aquatic stock.

Skill Level: 5

UNIT GROUP 8412 CROP FARM WORKERS

CROP FARM WORKERS perform routine tasks in producing crops such as fruit, nuts, grains and vegetables.

Indicative Skill Level:
Most occupations in this unit group have a level of skill commensurate with the qualifications outlined below.

In Australia:
AQF Certificate I, or compulsory secondary education (ANZSCO Skill Level 5)

In New Zealand:
NZ Register Level 1 qualification, or compulsory secondary education (ANZSCO Skill Level 5)

For some occupations a short period of on-the-job training may be required in addition to or instead of the formal qualification. In some instances no formal qualification or on-the-job training may be required.

Tasks Include:
- planting trees, seeds, seedlings, roots, bulbs, vines and other plants using hand tools and farm machines
- building trellises for climbing vegetables and vines
- operating farm machines to cultivate, fertilise, spray and harvest fruit, nuts, grains and vegetables
- spraying trees, vines and other plants with chemicals to control weed growth, insects, fungus growth and diseases
- thinning, weeding and hoeing row crops, and pruning trees and vines
- irrigating land for crop growth
- selecting and picking fruit, nuts, grains and vegetables according to size and ripeness, and discarding rotting and over-ripened produce
- grading, sorting, bunching and packing produce into containers
- loading filled fruit, nut, grain and vegetable containers onto trucks

Occupations:
841211 Fruit or Nut Farm Worker
841212 Fruit or Nut Picker
841213 Grain, Oilseed or Pasture Farm Worker (Aus) / Field Crop Farm Worker (NZ)
841214 Vegetable Farm Worker (Aus) / Market Garden Worker (NZ)
841215 Vegetable Picker
841216 Vineyard Worker
841299 Crop Farm Workers nec

841211 FRUIT OR NUT FARM WORKER

Performs routine tasks on a fruit or nut farm such as cultivating and fertilising soil, and planting, irrigating and pruning crops. May spray chemicals on crops to treat disease and pests.

Fruit or Nut Pickers are excluded from this occupation. Fruit or Nut Pickers are included in Occupation 841212 Fruit or Nut Picker.

Skill Level: 5

Specialisation:
Orchard Worker

UNIT GROUP 8412 CROP FARM WORKERS *continued*

841212 FRUIT OR NUT PICKER

Harvests fruit and nuts and prepares produce for distribution.

Fruit or Nut Farm Workers, and Grape Pickers are excluded from this occupation. Fruit or Nut Farm Workers are included in Occupation 841211 Fruit or Nut Farm Worker. Grape Pickers are included in Occupation 841216 Vineyard Worker.

Skill Level: 5

841213 GRAIN, OILSEED OR PASTURE FARM WORKER (AUS) / FIELD CROP FARM WORKER (NZ)

Performs routine tasks on a grain, oilseed protein, or pasture farm such as cultivating and fertilising soil, and planting and irrigating crops. May spray chemicals on crops to treat disease and pests.

Skill Level: 5

841214 VEGETABLE FARM WORKER (AUS) / MARKET GARDEN WORKER (NZ)

Performs routine tasks on a vegetable farm or market garden such as cultivating and fertilising soil, and planting and irrigating crops. May spray chemicals on crops to treat disease and pests.

Vegetable Pickers are excluded from this occupation. Vegetable Pickers are included in Occupation 841215 Vegetable Picker.

Skill Level: 5

841215 VEGETABLE PICKER

Harvests vegetables and prepares produce for distribution.

Vegetable Farm Workers (Aus) / Market Garden Workers (NZ) are excluded from this occupation. Vegetable Farm Workers (Aus) / Market Garden Workers (NZ) are included in Occupation 841214 Vegetable Farm Worker (Aus) / Market Garden Worker (NZ).

Skill Level: 5

841216 VINEYARD WORKER

Performs routine tasks on a vineyard such as cultivating and fertilising soil, planting, training and pruning vines, and picking grapes.

Skill Level: 5

UNIT GROUP 8412 CROP FARM WORKERS *continued*

841299 CROP FARM WORKERS NEC

This occupation group covers Crop Farm Workers not elsewhere classified.

Skill Level: 5

Occupations in this group include:

Coffee Plantation Worker (Aus)
Duboisia Farm Worker (Aus)
Flower Buncher or Picker
Hop Farm Worker
Lavender Farm Worker
Sugar Cane Planter (Aus)
Tea Plantation Worker (Aus)
Tea Tree Farm Worker
Tobacco Farm Worker
Turf Farm Worker

UNIT GROUP 8413 FORESTRY AND LOGGING WORKERS

FORESTRY AND LOGGING WORKERS perform routine tasks associated in cultivating and maintaining natural and plantation forests, and logging, felling and sawing trees.

Tree Surgeons are excluded from this unit group. Tree Surgeons are included in Unit Group 362212 Arborist.

Indicative Skill Level:
Most occupations in this unit group have a level of skill commensurate with the qualifications and experience outlined below.

In Australia:
AQF Certificate II or III (ANZSCO Skill Level 4)

In New Zealand:
NZ Register Level 2 or 3 qualification (ANZSCO Skill Level 4)

At least one year of relevant experience may substitute for the formal qualifications listed above. In some instances relevant experience and/or on-the-job training may be required in addition to the formal qualification.

Registration or licensing may be required.

Tasks Include:
- maintaining forest roads, buildings, facilities, signs and equipment
- killing weeds, felling and de-barking non-productive trees and thinning young plantations
- collecting seeds, and cultivating and planting seedlings for reafforestation purposes
- applying fertilisers, insecticides and herbicides to individual trees and general forest areas
- maintaining look-out for fires in forests
- removing major branches and tree tops, trimming branches and sawing trunks into logs
- assisting with loading and transporting logs
- planning the felling of trees and determining the natural and intended fall of each tree
- clearing surrounding area of saplings and debris prior to tree-felling
- operating and maintaining manual and machine saws to fell trees and to cut felled trees into logs

Occupations:
841311 Forestry Worker
841312 Logging Assistant
841313 Tree Faller

841311 FORESTRY WORKER

Assists with cultivating, maintaining and protecting forests. Registration or licensing may be required.

Skill Level: 4

Specialisations:
Fire Lookout
Tree Planter

841312 LOGGING ASSISTANT

Assists with logging, felling and sawing of trees in forests.

Skill Level: 4

Specialisation:
Sleeper Cutter

UNIT GROUP 8413 FORESTRY AND LOGGING WORKERS *continued*

841313 TREE FALLER

Alternative Title:

Tree Feller

Fells trees in forests, and trims and saws them into logs. Registration or licensing may be required.

Skill Level: 4

Specialisations:

Hardwood Faller

Softwood Faller

UNIT GROUP 8414 GARDEN AND NURSERY LABOURERS

GARDEN AND NURSERY LABOURERS perform a variety of routine tasks in propagating, cultivating and maintaining plants in gardens and horticultural nurseries.

Indicative Skill Level:
Most occupations in this unit group have a level of skill commensurate with the qualifications outlined below.

In Australia:
AQF Certificate I, or compulsory secondary education (ANZSCO Skill Level 5)

In New Zealand:
NZ Register Level 1 qualification, or compulsory secondary education (ANZSCO Skill Level 5)

For some occupations a short period of on-the-job training may be required in addition to or instead of the formal qualification. In some instances no formal qualification or on-the-job training may be required.

Tasks Include:
- loading, unloading and moving garden supplies and equipment
- preparing garden sites and plots using hand tools and machines
- assisting with planting and transplanting flowers, shrubs, trees and lawns
- maintaining gardens by watering, weeding and mowing lawns
- cleaning gardens and removing rubbish
- assisting with propagating, planting and potting seeds, bulbs and cuttings
- tending plants by hand watering and weeding
- adjusting mist irrigation systems, shade and ventilation
- harvesting and packaging plants for sale and transport

Occupations:
841411 Garden Labourer
841412 Horticultural Nursery Assistant

841411 GARDEN LABOURER

Assists in cultivating and maintaining gardens.

Skill Level: 5

Specialisation:
Lawn Mower

841412 HORTICULTURAL NURSERY ASSISTANT

Alternative Title:
Nursery Hand (Horticulture)

Assists in propagating, cultivating and harvesting plants in a horticultural nursery.

Skill Level: 5

UNIT GROUP 8415 LIVESTOCK FARM WORKERS

LIVESTOCK FARM WORKERS perform routine tasks in livestock, egg and wool production.

Indicative Skill Level:
Most occupations in this unit group have a level of skill commensurate with the qualifications outlined below.

In Australia:
AQF Certificate I, or compulsory secondary education (ANZSCO Skill Level 5)

In New Zealand:
NZ Register Level 1 qualification, or compulsory secondary education (ANZSCO Skill Level 5)

For some occupations a short period of on-the-job training may be required in addition to or instead of the formal qualification. In some instances no formal qualification or on-the-job training may be required.

Tasks Include:
- patrolling, inspecting and reporting on the condition of livestock
- providing livestock with feed and water
- assisting with maintaining the health and welfare of livestock
- mustering and droving livestock to milking and shearing sheds and between paddocks to ensure sufficient feed is available
- washing and cleaning udders, and attaching milking machines to udders and milking cows
- collecting eggs and placing in incubators
- herding sheep for shearing and keeping mobs separate during shearing
- spreading fleeces on skirting tables for classing, pressing wool and branding bales
- exercising horses by walking, riding, leading and swimming, and attending to horses at track work, barrier trials and races
- cleaning stables and hatcheries, storing bedding and performing minor repairs on fixtures, buildings and fences
- assembling, preparing and storing horse gear

Occupations:
841511 Beef Cattle Farm Worker
841512 Dairy Cattle Farm Worker
841513 Mixed Livestock Farm Worker
841514 Poultry Farm Worker
841515 Sheep Farm Worker
841516 Stablehand
841517 Wool Handler
841599 Livestock Farm Workers nec

841511 BEEF CATTLE FARM WORKER

Performs routine tasks on a beef cattle farm such as feeding, mustering and moving cattle, and assisting with animal husbandry.

Skill Level: 5

841512 DAIRY CATTLE FARM WORKER

Performs routine tasks on a dairy farm such as herding and milking cattle.

Skill Level: 5

UNIT GROUP 8415 LIVESTOCK FARM WORKERS *continued*

841513 MIXED LIVESTOCK FARM WORKER

Performs routine tasks on a mixed livestock farm such as moving, feeding and counting livestock, and assisting with animal husbandry.

Skill Level: 5

841514 POULTRY FARM WORKER

Performs routine tasks on a poultry farm such as collecting eggs and placing them in incubators, providing poultry with feed and water, and disinfecting hatcheries to prevent disease.

Skill Level: 5

841515 SHEEP FARM WORKER

Performs routine tasks on a sheep farm such as herding sheep into pens in preparation for branding, shearing, crutching, dipping and yarding for sale.

Skill Level: 5

841516 STABLEHAND

Assists with handling of horses and maintaining and cleaning stables.

Skill Level: 5

Specialisations:

- Horse Stud Worker
- Track Rider

841517 WOOL HANDLER

Alternative Titles:

- Shearing Shed Hand
- Shearing Shed Worker

Regulates the flow of sheep to be shorn, keeps the shearing shed clean and tidy, and assists with wool clip preparation.

Skill Level: 5

841599 LIVESTOCK FARM WORKERS NEC

This occupation group covers Livestock Farm Workers not elsewhere classified.

Skill Level: 5

Occupations in this group include:

- Deer Farm Worker
- Emu Farm Worker (Aus)
- Goat Herder
- Ostrich Farm Worker
- Piggery Worker

UNIT GROUP 8416 MIXED CROP AND LIVESTOCK FARM WORKERS

MIXED CROP AND LIVESTOCK FARM WORKERS perform routine tasks in crop cultivation and animal production.

Indicative Skill Level:
Most occupations in this unit group have a level of skill commensurate with the qualifications outlined below.

In Australia:
AQF Certificate I, or compulsory secondary education (ANZSCO Skill Level 5)

In New Zealand:
NZ Register Level 1 qualification, or compulsory secondary education (ANZSCO Skill Level 5)

For some occupations a short period of on-the-job training may be required in addition to or instead of the formal qualification. In some instances no formal qualification or on-the-job training may be required.

Tasks Include:

- patrolling, inspecting and reporting on the condition of crops and livestock
- operating farm machines to cultivate, plant, fertilise, spray and harvest field crops
- maintaining and repairing buildings, machines, fences, plant and water systems
- loading and unloading seed grain, fertilisers and livestock feed, and loading field crops into transporters for marketing
- irrigating land for crop growth
- providing livestock with feed and water
- assisting with maintaining the health and welfare of livestock
- mustering and droving livestock to milking and shearing sheds and between paddocks to ensure sufficient feed is available

Occupation:
841611 Mixed Crop and Livestock Farm Worker

841611 MIXED CROP AND LIVESTOCK FARM WORKER

Alternative Title:
General Farm Hand

Performs routine tasks in crop cultivation and animal production.

Skill Level: 5

UNIT GROUP 8419 OTHER FARM, FORESTRY AND GARDEN WORKERS

This unit group covers Farm, Forestry and Garden Workers not elsewhere classified.

It includes Hunter-Trappers and Pest or Weed Controllers.

Indicative Skill Level:
Most occupations in this unit group have a level of skill commensurate with the qualifications outlined below.

In Australia:
AQF Certificate I, or compulsory secondary education (ANZSCO Skill Level 5)

In New Zealand:
NZ Register Level 1 qualification, or compulsory secondary education (ANZSCO Skill Level 5)

For some occupations a short period of on-the-job training may be required in addition to or instead of the formal qualification. In some instances no formal qualification or on-the-job training may be required.

The occupation Pest or Weed Controller has a level of skill commensurate with the qualifications and experience outlined below.

In Australia:
AQF Certificate II or III (ANZSCO Skill Level 4)

In New Zealand:
NZ Register Level 2 or 3 qualification (ANZSCO Skill Level 4)

At least one year of relevant experience may substitute for the formal qualifications listed above. In some instances relevant experience and/or on-the-job training may be required in addition to the formal qualification.

Registration or licensing may be required.

Occupations:
- 841911 Hunter-Trapper
- 841912 Pest or Weed Controller
- 841999 Farm, Forestry and Garden Workers nec

841911 HUNTER-TRAPPER

Alternative Titles:
- Hunter
- Shooter

Hunts, traps and shoots animals for food, pelts, research and for pest control. Registration or licensing may be required.

Skill Level: 5

841912 PEST OR WEED CONTROLLER

Applies pest or weed management techniques to kill and control pests or weeds in domestic, commercial and industrial areas, roadsides, and private and public lands. Registration or licensing may be required.

Skill Level: 4

Specialisation:
- Fumigator

UNIT GROUP 8419 OTHER FARM, FORESTRY AND GARDEN WORKERS *continued*

841999 FARM, FORESTRY AND GARDEN WORKERS NEC

This occupation group covers Farm, Forestry and Garden Workers not elsewhere classified.

Skill Level: 5

Occupations in this group include:

- Coral Collector
- Indoor Plant Technician
- Irrigationist
- Kelp or Seagrass Gatherer
- Seed Collector

SUB-MAJOR GROUP 85 FOOD PREPARATION ASSISTANTS

FOOD PREPARATION ASSISTANTS prepare food in fast food establishments, assist Food Trades Workers and service staff to prepare and serve food, and clean food preparation and service areas.

Indicative Skill Level:
Most occupations in this sub-major group have a level of skill commensurate with the qualifications outlined below.

In Australia:
AQF Certificate I, or compulsory secondary education (ANZSCO Skill Level 5)

In New Zealand:
NZ Register Level 1 qualification, or compulsory secondary education (ANZSCO Skill Level 5)

For some occupations a short period of on-the-job training may be required in addition to or instead of the formal qualification. In some instances no formal qualification or on-the-job training may be required.

Tasks Include:
- assisting Cooks and Chefs by assembling and preparing ingredients
- preparing salads, sandwiches, hamburgers, fish and chips and other fast foods
- packing food and beverages in containers and on trays for serving
- cleaning kitchens and food preparation areas
- cleaning cooking and general utensils

Occupations in this sub-major group are classified into the following minor group:
851 Food Preparation Assistants

MINOR GROUP 851 FOOD PREPARATION ASSISTANTS

FOOD PREPARATION ASSISTANTS prepare food in fast food establishments, assist Food Trades Workers and service staff to prepare and serve food, and clean food preparation and service areas.

Indicative Skill Level:
Most occupations in this minor group have a level of skill commensurate with the qualifications outlined below.

In Australia:
AQF Certificate I, or compulsory secondary education (ANZSCO Skill Level 5)

In New Zealand:
NZ Register Level 1 qualification, or compulsory secondary education (ANZSCO Skill Level 5)

For some occupations a short period of on-the-job training may be required in addition to or instead of the formal qualification. In some instances no formal qualification or on-the-job training may be required.

Tasks Include:
- assisting Cooks and Chefs by assembling and preparing ingredients
- preparing salads, sandwiches, hamburgers, fish and chips and other fast foods
- packing food and beverages in containers and on trays for serving
- cleaning kitchens and food preparation areas
- cleaning cooking and general utensils

Occupations in this minor group are classified into the following unit groups:

8511 Fast Food Cooks
8512 Food Trades Assistants
8513 Kitchenhands

UNIT GROUP 8511 FAST FOOD COOKS

FAST FOOD COOKS prepare a restricted range of foods in fast food establishments.

Indicative Skill Level:
Most occupations in this unit group have a level of skill commensurate with the qualifications outlined below.

In Australia:
AQF Certificate I, or compulsory secondary education (ANZSCO Skill Level 5)

In New Zealand:
NZ Register Level 1 qualification, or compulsory secondary education (ANZSCO Skill Level 5)

For some occupations a short period of on-the-job training may be required in addition to or instead of the formal qualification. In some instances no formal qualification or on-the-job training may be required.

Tasks Include:
- taking and serving food and beverage orders, and receiving payment from customers
- preparing food such as hamburgers, pizzas, fish and chips
- washing, cutting, measuring and mixing foods for cooking
- operating cooking equipment such as grills, microwaves and deep-fat fryers
- cleaning food preparation areas, cooking surfaces and utensils
- ordering and taking delivery of fast food ingredients
- may arrange delivery of prepared food and beverages

Occupation:
851111 Fast Food Cook

851111 FAST FOOD COOK

Prepares a restricted range of foods in a fast food establishment.

Skill Level: 5

Specialisation:
Short Order Cook

UNIT GROUP 8512 FOOD TRADES ASSISTANTS

FOOD TRADES ASSISTANTS assist Food Trades Workers by performing routine food preparation, cleaning and storage tasks.

Indicative Skill Level:
Most occupations in this unit group have a level of skill commensurate with the qualifications outlined below.

In Australia:
AQF Certificate I, or compulsory secondary education (ANZSCO Skill Level 5)

In New Zealand:
NZ Register Level 1 qualification, or compulsory secondary education (ANZSCO Skill Level 5)

For some occupations a short period of on-the-job training may be required in addition to or instead of the formal qualification. In some instances no formal qualification or on-the-job training may be required.

Tasks Include:
- gathering food ingredients in preparation for cooking and serving
- gathering pots and pans in preparation for cooking food ingredients
- weighing and measuring ingredients
- washing, peeling, cutting, slicing and dicing ingredients for cooking and serving
- cutting and grinding meat, poultry and seafood in preparation for cooking
- removing cooked food from ovens and food warmers
- washing work areas, equipment, utensils, dishes and silverware
- storing food supplies, equipment, utensils, dishes and silverware in refrigerators, cupboards, pantries and other storage areas
- informing Foods Trade Workers when food supplies are low

Occupations:
851211 Pastrycook's Assistant
851299 Food Trades Assistants nec

851211 PASTRYCOOK'S ASSISTANT

Assists a Pastrycook by performing routine tasks in the kitchen such as preparing ingredients, and cleaning and storing equipment.

Skill Level: 5

Specialisation:
Bakery Assistant

851299 FOOD TRADES ASSISTANTS NEC

This occupation group covers Food Trades Assistants not elsewhere classified.

Skill Level: 5

Occupations in this group include:
Butcher's Assistant
Chef's Assistant
Cook's Assistant

UNIT GROUP 8513 KITCHENHANDS

KITCHENHANDS assist kitchen and service staff in preparing and serving food, and clean food preparation and service areas.

Indicative Skill Level:
Most occupations in this unit group have a level of skill commensurate with the qualifications outlined below.

In Australia:
AQF Certificate I, or compulsory secondary education (ANZSCO Skill Level 5)

In New Zealand:
NZ Register Level 1 qualification, or compulsory secondary education (ANZSCO Skill Level 5)

For some occupations a short period of on-the-job training may be required in addition to or instead of the formal qualification. In some instances no formal qualification or on-the-job training may be required.

Tasks Include:
- cleaning kitchens, food preparation areas and sculleries
- cleaning cooking and general utensils used in kitchens and restaurants
- transferring, weighing and checking supplies and equipment
- assembling and preparing ingredients for cooking, and preparing salads, savouries and sandwiches
- packing food and beverage trays for serving
- cooking, toasting and heating simple food items

Occupation:
851311 Kitchenhand

851311 KITCHENHAND

Alternative Title:
Kitchen Steward

Assists kitchen and service staff in preparing and serving food, and cleans food preparation and service areas.

Skill Level: 5

Specialisations:
Dishwasher
Pantry Attendant
Sandwich Hand

SUB-MAJOR GROUP 89 OTHER LABOURERS

This sub-major group covers Labourers not elsewhere classified.

It includes Freight Handlers and Shelf Fillers.

Indicative Skill Level:
Most occupations in this sub-major group have a level of skill commensurate with the qualifications and experience outlined below.

In Australia:
AQF Certificate II or III, or at least one year of relevant experience (ANZSCO Skill Level 4); or
AQF Certificate I, or compulsory secondary education (ANZSCO Skill Level 5)

In New Zealand:
NZ Register Level 2 or 3 qualification, or at least one year of relevant experience (ANZSCO Skill Level 4); or
NZ Register Level 1 qualification, or compulsory secondary education (ANZSCO Skill Level 5)

In some instances relevant experience and/or on-the-job training may be required in addition to the formal qualification. In the case of some Skill Level 5 occupations, a short period of on-the-job training may be required in addition to or instead of the formal qualification, or no formal qualification or on-the-job training may be required.

Occupations in this sub-major group are classified into the following minor groups:
891 Freight Handlers and Shelf Fillers
899 Miscellaneous Labourers

MINOR GROUP 891 FREIGHT HANDLERS AND SHELF FILLERS

FREIGHT HANDLERS AND SHELF FILLERS load and unload trucks, containers and rail cars, transfer cargo between ships and other forms of transport and storage facilities, and fill shelves in stores and supermarkets.

Indicative Skill Level:
Most occupations in this minor group have a level of skill commensurate with the qualifications outlined below.

In Australia:
AQF Certificate I, or compulsory secondary education (ANZSCO Skill Level 5)

In New Zealand:
NZ Register Level 1 qualification, or compulsory secondary education (ANZSCO Skill Level 5)

For some occupations a short period of on-the-job training may be required in addition to or instead of the formal qualification. In some instances no formal qualification or on-the-job training may be required.

Tasks Include:
- receiving goods and checking against documents
- operating equipment to load, unload and move goods
- securing loads to prevent shifting during transport
- replenishing empty shelves in stores and supermarkets
- collecting goods from stockrooms to place on shelves

Occupations in this minor group are classified into the following unit groups:
8911 Freight and Furniture Handlers
8912 Shelf Fillers

UNIT GROUP 8911 FREIGHT AND FURNITURE HANDLERS

FREIGHT AND FURNITURE HANDLERS load and unload trucks, containers and rail cars, and transfer cargo between ships and other forms of transport and storage facilities.

Indicative Skill Level:
Most occupations in this unit group have a level of skill commensurate with the qualifications outlined below.

In Australia:
AQF Certificate I, or compulsory secondary education (ANZSCO Skill Level 5)

In New Zealand:
NZ Register Level 1 qualification, or compulsory secondary education (ANZSCO Skill Level 5)

For some occupations a short period of on-the-job training may be required in addition to or instead of the formal qualification. In some instances no formal qualification or on-the-job training may be required.

Tasks Include:
- labelling goods with customers' details and destinations
- loading goods into trucks, containers and rail wagons, and securing loads
- assisting to tie down loads and cover them with tarpaulins
- guiding truck drivers into loading bays and through confined spaces
- performing clerical functions to record and check cargo on arrival, storage and despatch
- opening and closing hatches and securing cargo to prevent shifting during voyages
- transferring loads using moving equipment and directing equipment operations using communication systems
- stacking cargo on pallets, trays, flats and slings to facilitate transfer to and from ships

Occupations:
891111 Freight Handler (Rail or Road)
891112 Truck Driver's Offsider
891113 Waterside Worker

891111 FREIGHT HANDLER (RAIL OR ROAD)

Alternative Title:
Freight Loader

Loads and unloads freight trucks, containers and rail cars.

Skill Level: 5

891112 TRUCK DRIVER'S OFFSIDER

Loads and unloads trucks and containers.

Skill Level: 5

Specialisation:
Furniture Removalist's Assistant

UNIT GROUP 8911 FREIGHT AND FURNITURE HANDLERS *continued*

891113 WATERSIDE WORKER

Alternative Titles:

Stevedore

Wharf Labourer

Transfers cargo between ships and other forms of transport or storage facilities.

Skill Level: 5

UNIT GROUP 8912 SHELF FILLERS

SHELF FILLERS fill up shelves and display areas in stores and supermarkets.

Indicative Skill Level:
Most occupations in this unit group have a level of skill commensurate with the qualifications outlined below.

In Australia:
AQF Certificate I, or compulsory secondary education (ANZSCO Skill Level 5)

In New Zealand:
NZ Register Level 1 qualification, or compulsory secondary education (ANZSCO Skill Level 5)

For some occupations a short period of on-the-job training may be required in addition to or instead of the formal qualification. In some instances no formal qualification or on-the-job training may be required.

Tasks Include:
- placing goods neatly in bins and on racks, and stacking bulky goods on floors
- filling shelves with goods ensuring goods with the earliest use-by dates are at the front of shelves
- noting what has been sold and collecting goods needed from the stockroom using a trolley
- maintaining shelf order by removing stock belonging to a different location
- may help customers find goods they need
- may price goods

Occupation:
891211 Shelf Filler

891211 SHELF FILLER

Alternative Title:
Night Filler

Fills up shelves and display areas in a store or supermarket.

Skill Level: 5

MINOR GROUP 899 MISCELLANEOUS LABOURERS

This minor group includes Labourers not elsewhere classified.

It includes Caretakers, Deck and Fishing Hands, Handypersons, Motor Vehicle Parts and Accessories Fitters, Printing Assistants and Table Workers, Recycling and Rubbish Collectors, and Vending Machine Attendants.

Indicative Skill Level:
Most occupations in this minor group have a level of skill commensurate with the qualifications and experience outlined below.

In Australia:
AQF Certificate II or III, or at least one year of relevant experience (ANZSCO Skill Level 4); or
AQF Certificate I, or compulsory secondary education (ANZSCO Skill Level 5)

In New Zealand:
NZ Register Level 2 or 3 qualification, or at least one year of relevant experience (ANZSCO Skill Level 4); or
NZ Register Level 1 qualification, or compulsory secondary education (ANZSCO Skill Level 5)

In some instances relevant experience and/or on-the-job training may be required in addition to the formal qualification. In the case of some Skill Level 5 occupations, a short period of on-the-job training may be required in addition to or instead of the formal qualification, or no formal qualification or on-the-job training may be required.

Occupations in this minor group are classified into the following unit groups:
8991 Caretakers
8992 Deck and Fishing Hands
8993 Handypersons
8994 Motor Vehicle Parts and Accessories Fitters
8995 Printing Assistants and Table Workers
8996 Recycling and Rubbish Collectors
8997 Vending Machine Attendants
8999 Other Miscellaneous Labourers

UNIT GROUP 8991 CARETAKERS

CARETAKERS maintain and clean residential buildings, schools, offices, holiday camps, caravan parks and associated grounds.

Indicative Skill Level:
Most occupations in this unit group have a level of skill commensurate with the qualifications outlined below.

In Australia:
AQF Certificate I, or compulsory secondary education (ANZSCO Skill Level 5)

In New Zealand:
NZ Register Level 1 qualification, or compulsory secondary education (ANZSCO Skill Level 5)

For some occupations a short period of on-the-job training may be required in addition to or instead of the formal qualification. In some instances no formal qualification or on-the-job training may be required.

Tasks Include:
- filling out registration forms and providing tenants with copies of rules
- collecting rent, and filling out and issuing receipts
- cleaning common facilities, grounds and gardens, replacing light bulbs, checking fire hoses and extinguishers, and performing other cleaning and maintenance tasks
- notifying management and owners of buildings of the need for major repairs
- cautioning tenants regarding excessive noise, disorderly conduct and abuse of property
- patrolling buildings to ensure security is maintained
- purchasing cleaning supplies

Occupation:
899111 Caretaker

899111 CARETAKER

Maintains and cleans a residential building, school, office, holiday camp or caravan park and associated grounds.

Skill Level: 5

Specialisation:
Janitor

UNIT GROUP 8992 DECK AND FISHING HANDS

DECK AND FISHING HANDS maintain ships' equipment and structures, and catch fish, crustacea and molluscs.

Indicative Skill Level:
Most occupations in this unit group have a level of skill commensurate with the qualifications and experience outlined below.

In Australia:
AQF Certificate II or III (ANZSCO Skill Level 4)

In New Zealand:
NZ Register Level 2 or 3 qualification (ANZSCO Skill Level 4)

At least one year of relevant experience may substitute for the formal qualifications listed above. In some instances relevant experience and/or on-the-job training may be required in addition to the formal qualification.

Tasks Include:
- handling ropes and wires, and operating mooring equipment when berthing and unberthing
- standing lookout watches at sea and adjusting the ship's course as directed
- assisting with cargo operations using on-board equipment and stowing and securing cargo
- patrolling ships to ensure safety of the vessel, cargo and passengers
- performing routine maintenance and checks on deck equipment, cargo gear, rigging, and lifesaving and firefighting appliances
- attaching gear and fastening towing cables to nets
- casting and lowering nets, pots, lines and traps into water
- preparing lines, attaching running gear and bait, and setting lines into position
- hauling in fishing gear and removing fish and other marine life
- sorting, cleaning, preserving, stowing and refrigerating catch

Occupations:
899211 Deck Hand
899212 Fishing Hand

899211 DECK HAND

Alternative Title:
Seafarer

Performs maintenance and lookout tasks aboard a ship.

Skill Level: 4

Specialisations:
Barge Hand
Ferry Hand
Tug Hand

UNIT GROUP 8992 DECK AND FISHING HANDS *continued*

899212 FISHING HAND

Alternative Title:

Fishing Boat Mate

Catches fish, crustacea and molluscs using nets, pots, lines and traps in ocean and inland waters.

Skill Level: 4

Specialisations:

Cray Fishing Hand
Prawn Trawler Hand
Purse Seining Hand

UNIT GROUP 8993 HANDYPERSONS

HANDYPERSONS clean, paint, repair and maintain buildings, grounds and facilities.

Indicative Skill Level:
Most occupations in this unit group have a level of skill commensurate with the qualifications outlined below.

In Australia:
AQF Certificate I, or compulsory secondary education (ANZSCO Skill Level 5)

In New Zealand:
NZ Register Level 1 qualification, or compulsory secondary education (ANZSCO Skill Level 5)

For some occupations a short period of on-the-job training may be required in addition to or instead of the formal qualification. In some instances no formal qualification or on-the-job training may be required.

Tasks Include:
- repairing broken windows, screens, doors, fences, barbecues, picnic tables, shelves, cupboards and other items
- replacing defective items such as light bulbs
- repairing and painting interior and exterior surfaces such as walls, ceilings and fences
- clearing rubbish and leaves from driveways and grounds
- mowing lawns and cultivating gardens
- adjusting doors and windows
- replacing tap washers
- putting up handrails and grab rails

Occupation:
899311 Handyperson

899311 HANDYPERSON

Cleans, paints, repairs and maintains buildings, grounds and facilities.

Skill Level: 5

Specialisations:
Hotel Useful
Hotel Yardperson

UNIT GROUP 8994 MOTOR VEHICLE PARTS AND ACCESSORIES FITTERS

MOTOR VEHICLE PARTS AND ACCESSORIES FITTERS fit and replace parts and accessories on motor vehicles.

Indicative Skill Level:
Most occupations in this unit group have a level of skill commensurate with the qualifications and experience outlined below.

In Australia:
AQF Certificate II or III (ANZSCO Skill Level 4)

In New Zealand:
NZ Register Level 2 or 3 qualification (ANZSCO Skill Level 4)

At least one year of relevant experience may substitute for the formal qualifications listed above. In some instances relevant experience and/or on-the-job training may be required in addition to the formal qualification.

Tasks Include:
- removing old and damaged parts and cleaning surrounding areas on vehicles
- fitting batteries and installing accessories such as sun roofs, stereos and alarms
- removing damaged glass, trimming strips and rubber seals from window frames and mountings on motor vehicles, positioning new windscreens and glass windows on frames and attaching and sealing them
- inspecting, removing and repairing muffler mountings, and fitting new mufflers, extractors and exhaust pipes
- removing radiators from vehicles and cleaning and repairing them
- installing new or repaired radiators into vehicles and repairing and replacing other units in the cooling system such as thermostats, head gaskets and water pumps
- inspecting tyres to determine which repair action to implement and repairing punctures in tubes and tubeless tyres
- operating air driven equipment to remove and refit tyres and tubes on vehicles
- balancing wheels and tyres using static and electronic equipment

Occupations:
899411 Motor Vehicle Parts and Accessories Fitter (General)
899412 Autoglazier
899413 Exhaust and Muffler Fitter
899414 Radiator Fitter
899415 Tyre Fitter

899411 MOTOR VEHICLE PARTS AND ACCESSORIES FITTER (GENERAL)

Fits and replaces parts and accessories on motor vehicles.

Skill Level: 4

899412 AUTOGLAZIER

Alternative Title:
Windscreen Fitter

Repairs and replaces windscreens and side and rear glass in motor vehicles.

Skill Level: 4

Specialisation:
Vehicle Window Tinter

UNIT GROUP 8994 MOTOR VEHICLE PARTS AND ACCESSORIES FITTERS *continued*

899413 EXHAUST AND MUFFLER FITTER

Repairs and replaces defective exhaust and muffler systems in motor vehicles.

Skill Level: 4

899414 RADIATOR FITTER

Repairs and replaces radiators and cooling systems in motor vehicles.

Skill Level: 4

899415 TYRE FITTER

Fits, repairs and replaces tyres on motor vehicles.

Skill Level: 4

UNIT GROUP 8995 PRINTING ASSISTANTS AND TABLE WORKERS

PRINTING ASSISTANTS AND TABLE WORKERS perform routine printing tasks, operate bindery machines and perform manual binding and finishing of books and printed products.

Indicative Skill Level:
Most occupations in this unit group have a level of skill commensurate with the qualifications and experience outlined below.

In Australia:
AQF Certificate II or III (ANZSCO Skill Level 4)

In New Zealand:
NZ Register Level 2 or 3 qualification (ANZSCO Skill Level 4)

At least one year of relevant experience may substitute for the formal qualifications listed above. In some instances relevant experience and/or on-the-job training may be required in addition to the formal qualification.

Tasks Include:
- assisting with setting up, operating and adjusting machines
- maintaining and lubricating printing and bindery machines
- clearing waste and cleaning work areas and machines
- folding, collating and fastening printed products by machine and hand
- performing hand binding and finishing operations
- operating specialised machines such as casing-in and mini binders, and automatic carton folding and gluing machines
- carrying out routine quality control

Occupations:
899511 Printer's Assistant
899512 Printing Table Worker

899511 PRINTER'S ASSISTANT

Performs routine tasks in the production of printed materials.

Skill Level: 4

899512 PRINTING TABLE WORKER

Alternative Titles:
Printing Bindery Assistant
Printing Table Hand

Operates bindery machines and performs manual binding and finishing of books and printed products.

Skill Level: 4

UNIT GROUP 8996 RECYCLING AND RUBBISH COLLECTORS

RECYCLING AND RUBBISH COLLECTORS collect household, commercial and industrial waste for recycling and disposal.

Indicative Skill Level:
Most occupations in this unit group have a level of skill commensurate with the qualifications outlined below.

In Australia:
AQF Certificate I, or compulsory secondary education (ANZSCO Skill Level 5)

In New Zealand:
NZ Register Level 1 qualification, or compulsory secondary education (ANZSCO Skill Level 5)

For some occupations a short period of on-the-job training may be required in addition to or instead of the formal qualification. In some instances no formal qualification or on-the-job training may be required.

Tasks Include:
- riding on and in garbage and recycling trucks
- collecting rubbish and items for recycling from domestic, commercial and industrial premises
- loading rubbish and recycling into bins and garbage and recycling trucks
- unloading garbage and recycling trucks
- may operate compacting equipment on garbage trucks
- may supervise other garbage collectors

Occupation:
899611 Recycling or Rubbish Collector

899611 RECYCLING OR RUBBISH COLLECTOR

Alternative Title:
Waste Removalist

Collects household, commercial and industrial waste for recycling or disposal.

Skill Level: 5

Specialisation:
Garbage Depot Worker

UNIT GROUP 8997 VENDING MACHINE ATTENDANTS

VENDING MACHINE ATTENDANTS stock and maintain vending and amusement machines and collect money from coin boxes.

Indicative Skill Level:
Most occupations in this unit group have a level of skill commensurate with the qualifications outlined below.

In Australia:
AQF Certificate I, or compulsory secondary education (ANZSCO Skill Level 5)

In New Zealand:
NZ Register Level 1 qualification, or compulsory secondary education (ANZSCO Skill Level 5)

For some occupations a short period of on-the-job training may be required in addition to or instead of the formal qualification. In some instances no formal qualification or on-the-job training may be required.

Tasks Include:
- loading, unloading and transporting stock and equipment
- replenishing vending machines with stock
- clearing money from machines, accounting for money collected, and checking monitoring systems
- keeping stock records, and machine maintenance and repair records
- may test vending machines' dispensing, coin-handling, electrical, refrigeration, carbonation and ice-making systems
- may adjust and repair vending machines and replace defective mechanical and electrical parts using hand tools and soldering-irons

Occupation:
899711 Vending Machine Attendant

899711 VENDING MACHINE ATTENDANT

Alternative Title:
Vending Machine Refiller

Stocks and maintains vending and amusement machines and collects money from coin boxes.

Skill Level: 5

Specialisation:
Poker Machine Attendant

UNIT GROUP 8999 OTHER MISCELLANEOUS LABOURERS

This unit group covers Labourers not elsewhere classified.

It includes Bicycle Mechanics, Car Park Attendants, Crossing Supervisors, Electrical or Telecommunications Trades Assistants, Leaflet or Newspaper Deliverers, Mechanic's Assistants, Railways Assistants, Sign Erectors, Ticket Collectors or Ushers, and Trolley Collectors.

Indicative Skill Level:
Most occupations in this unit group have a level of skill commensurate with the qualifications outlined below.

In Australia:
AQF Certificate I, or compulsory secondary education (ANZSCO Skill Level 5)

In New Zealand:
NZ Register Level 1 qualification, or compulsory secondary education (ANZSCO Skill Level 5)

For some occupations a short period of on-the-job training may be required in addition to or instead of the formal qualification. In some instances no formal qualification or on-the-job training may be required.

Occupations:
899911 Bicycle Mechanic
899912 Car Park Attendant
899913 Crossing Supervisor
899914 Electrical or Telecommunications Trades Assistant
899915 Leaflet or Newspaper Deliverer
899916 Mechanic's Assistant
899917 Railways Assistant
899918 Sign Erector
899921 Ticket Collector or Usher
899922 Trolley Collector
899999 Labourers nec

899911 BICYCLE MECHANIC

Alternative Titles:
Bicycle Repairer
Bicycle Technician

Repairs and adjusts bicycles, and assembles bicycle kits.

Skill Level: 5

899912 CAR PARK ATTENDANT

Operates and maintains a car parking facility by guarding cars in parking areas and collecting fees at car park entry or exit points. May drive and park cars, and operate boom gates.

Skill Level: 5

899913 CROSSING SUPERVISOR

Assists children, disabled and other pedestrians to cross roads by stopping traffic and ensuring all pedestrians have crossed safely before allowing traffic to flow through the crossing.

Skill Level: 5

UNIT GROUP 8999 OTHER MISCELLANEOUS LABOURERS *continued*

899914 ELECTRICAL OR TELECOMMUNICATIONS TRADES ASSISTANT

Assists Electrotechnology and Telecommunications Trades Workers to install and maintain electrical and telecommunications systems.

Skill Level: 5

899915 LEAFLET OR NEWSPAPER DELIVERER

Collects leaflets or newspapers from a collection point and delivers them to homes in a specified area.

Skill Level: 5

899916 MECHANIC'S ASSISTANT

Assists Motor Mechanics to replace and repair worn and defective parts, re-assemble mechanical components, change oil and filters, and perform other routine mechanical tasks.

Skill Level: 5

Specialisation:
Lube Attendant

899917 RAILWAYS ASSISTANT

Assists with operating and maintaining facilities at a railway station by updating platform indicators showing train times and destinations, collecting and checking passenger tickets, giving signals for train departures, and cleaning station facilities.

Skill Level: 5

899918 SIGN ERECTOR

Erects and installs signs, and cleans signs and their sites after installation.

Skill Level: 5

899921 TICKET COLLECTOR OR USHER

Alternative Title:
Venue Attendant

Collects tickets or admission passes and ushers patrons to their seats at an entertainment, sporting or recreational venue, prepares the venue before an event and locks up premises afterwards.

Skill Level: 5

Specialisations:
Entertainment Usher
Gatekeeper
Turnstile Attendant

899922 TROLLEY COLLECTOR

Collects supermarket trolleys from car parks and other areas, and returns them to the supermarket by hand or trailer. May drive a small tractor to tow the trolleys.

Skill Level: 5

UNIT GROUP 8999 OTHER MISCELLANEOUS LABOURERS *continued*

899999 LABOURERS NEC

This occupation group covers Labourers not elsewhere classified.

Skill Level: 5

Occupations in this group include:

- Bowling Alley Attendant
- Grip
- Milk Runner
- Racecourse Barrier Attendant
- Road Maintenance Traffic Controller
- Stagehand
- Studio Hand
- Swimming Pool Serviceperson

APPENDIX A SUMMARY CORRESPONDENCE BETWEEN ANZSCO AND ASCO SECOND EDITION AND NZSCO 1999

The summary correspondence table (concordance) presented here provides an overview of the conceptual relationship between occupations in the Australian and New Zealand Standard Classification of Occupations (ANZSCO) and both the Australian Standard Classification of Occupations (ASCO) Second Edition and the New Zealand Standard Classification of Occupations (NZSCO) 1999.

The letter 'p' indicates where there is a partial match from one classification to the other. For example:

ASCO Second Edition			ANZSCO		NZSCO 1999		
p = partial match					p = partial match		
6199-15	p	Mail Supervisor	561212	Postal Delivery Officer	41423		Postal Deliverer
8114-13		Postal Delivery Officer			41424	p	Mail Delivery Contractor
					91211	p	Courier and Deliverer

The relationship between ANZSCO and ASCO Second Edition illustrated in the correspondence table above, indicates that the ANZSCO occupation 561212 Postal Delivery Officer comprises some of the jobs which were classified in the ASCO Second Edition occupation 6199-15 Mail Supervisor and all of the jobs classified in the ASCO Second Edition occupation 8114-13 Postal Delivery Officer.

The relationship between ANZSCO and NZSCO 1999 illustrated above, indicates that the ANZSCO occupation 561212 Postal Delivery Officer comprises all of the jobs which were classified in the NZSCO 1999 occupation 41423 Postal Deliverer, some of the jobs classified in the NZSCO 1999 occupation 41424 Mail Delivery Contractor and some of the jobs classified in the NZSCO 1999 occupation 91211 Courier and Deliverer.

Detailed correspondence tables between ANZSCO and ASCO Second Edition, and ANZSCO and NZSCO 1999 will be available in electronic format from the Australian Bureau of Statistics (ABS) and Statistics New Zealand (Statistics NZ) web sites.

These correspondences present important summary information for understanding the changes that have taken place in the definition of occupations between ANZSCO and ASCO Second Edition, and ANZSCO and NZSCO 1999. They do not, however, identify the proportion of an ASCO Second Edition occupation or an NZSCO 1999 occupation that would fall into a relevant ANZSCO occupation and vice versa.

In Australia, this information will be compiled by coding 2006 Australian Census of Population and Housing data to both the ANZSCO and ASCO Second Edition. A number of other ABS collections, including the Australian Labour Force Survey will be dual-coded to both ANZSCO and ASCO Second Edition to assist in maintaining time series.

In New Zealand, this information will be compiled by coding 2006 New Zealand Census of Population and Dwellings data to both the ANZSCO and NZSCO 1999. A number of other Statistics NZ collections, including the Household Labour Force Survey will be dual-coded to both ANZSCO and NZSCO 1999 to assist in maintaining time series.

The ABS will produce a detailed link file following completion of data processing of the 2006 Australian Census of Population and Housing. The link file will show the one-to-one links and numerical/proportional relationships between the categories of ASCO Second Edition and ANZSCO.

ASCO Second Edition			ANZSCO		NZSCO 1999		
			**1**	**MANAGERS**			
1112-11	p	General Manager	111111	Chief Executive or Managing Director	11211		Chief Executive - Central Government
					11212		Chief Executive - Local Government
					11311		Chief Executive and/or Managing Director
					12111	p	General Manager
1112-11	p	General Manager	111211	Corporate General Manager	11411	p	Special-Interest Organisation Administrator
					12111	p	General Manager
					12212	p	Broadcasting and Theatrical Production Manager
					12213	p	Production Manager (Manufacturing)
					33613	p	Editor
1112-11	p	General Manager	111212	Defence Force Senior Officer	51551	p	Armed Forces
1111-11	p	Parliamentarian or Councillor	111311	Local Government Legislator	11112		Local Government Legislator
1111-11	p	Parliamentarian or Councillor	111312	Member of Parliament	11111		Member of Parliament
1111-11	p	Parliamentarian or Councillor	111399	Legislators nec	No equivalent category		
1111-79	p	Legislators and Government Appointed Officials nec					
1314-11		Aquaculture Farmer	121111	Aquaculture Farmer	61421	p	Fish Farmer, Worker
4611-11	p	Farm Overseer			61422	p	Mussel and Oyster Farmer, Worker
1313-79	p	Crop Farmers nec	121211	Cotton Grower	61111	p	Field Crop Grower and Related Worker
4611-11	p	Farm Overseer					
1313-21		Flower Grower	121212	Flower Grower	61131	p	Nursery Grower, Nursery Worker
4611-11	p	Farm Overseer					
1313-17	p	Fruit and Nut Grower	121213	Fruit or Nut Grower	61121	p	Fruit Grower, Worker
4611-11	p	Farm Overseer					
1313-11		Grain, Oilseed and Pasture Grower	121214	Grain, Oilseed or Pasture Grower (Aus) / Field Crop Grower (NZ)	61111	p	Field Crop Grower and Related Worker
4611-11	p	Farm Overseer					
1313-17	p	Fruit and Nut Grower	121215	Grape Grower	61122	p	Grape Grower and/or Wine Maker, Worker
4611-11	p	Farm Overseer					
1313-00		Crop Farmers, nfd	121216	Mixed Crop Farmer	61111	p	Field Crop Grower and Related Worker
4611-11	p	Farm Overseer					
1313-13		Sugar Cane Grower	121217	Sugar Cane Grower	61111	p	Field Crop Grower and Related Worker
4611-11	p	Farm Overseer					
1313-79	p	Crop Farmers nec	121218	Turf Grower	61111	p	Field Crop Grower and Related Worker
4611-11	p	Farm Overseer					
1313-19		Vegetable Grower	121221	Vegetable Grower (Aus) / Market Gardener (NZ)	61111	p	Field Crop Grower and Related Worker
4611-11	p	Farm Overseer			61112	p	Market Gardener and Related Worker
1313-15		Tobacco Grower	121299	Crop Farmers nec	61111	p	Field Crop Grower and Related Worker
1313-79	p	Crop Farmers nec			61131	p	Nursery Grower, Nursery Worker
4611-11	p	Farm Overseer					
1312-25		Apiarist	121311	Apiarist	61241	p	Apiarist and Apiary Worker
1312-13		Beef Cattle Farmer	121312	Beef Cattle Farmer	61213	p	Cattle Farmer, Cattle Farm Worker
4611-11	p	Farm Overseer					
1312-15		Dairy Farmer	121313	Dairy Cattle Farmer	61211	p	Dairy Farmer, Dairy Farm Worker
4611-11	p	Farm Overseer					
1312-79	p	Livestock Farmers nec	121314	Deer Farmer	61216	p	Deer Farmer, Deer Farm Worker
4611-11	p	Farm Overseer					
1312-79	p	Livestock Farmers nec	121315	Goat Farmer	61215	p	Goat Farmer, Goat Farm Worker
4611-11	p	Farm Overseer					
1312-23		Horse Breeder	121316	Horse Breeder	61217	p	Stud Racehorse Breeder, Stud Worker
4611-11	p	Farm Overseer					
1312-11		Mixed Livestock Farmer	121317	Mixed Livestock Farmer	61221	p	Mixed Livestock Farmer, Mixed Livestock Farm Worker
4611-11	p	Farm Overseer					
1312-19		Pig Farmer	121318	Pig Farmer	61214	p	Pig Farmer, Pig Farm Worker
4611-11	p	Farm Overseer					
1312-21		Poultry Farmer	121321	Poultry Farmer	61231	p	Poultry Farmer and Poultry Farm Worker
4611-11	p	Farm Overseer					
1312-17		Sheep Farmer	121322	Sheep Farmer	61212	p	Sheep Farmer, Sheep Farm Worker
4611-11	p	Farm Overseer					
1312-79	p	Livestock Farmers nec	121399	Livestock Farmers nec	61218	p	Other Livestock Farmer, Other Livestock Farm Worker
4611-11	p	Farm Overseer			61221	p	Mixed Livestock Farmer, Mixed Livestock Farm Worker
1311-11		Mixed Crop and Livestock Farmer	121411	Mixed Crop and Livestock Farmer	61213	p	Cattle Farmer, Cattle Farm Worker
4611-11	p	Farm Overseer			61251	p	Crop and Livestock Farmer, Worker

ASCO Second Edition			ANZSCO		NZSCO 1999		
1231-11	p	Sales and Marketing Manager	131111	Advertising and Public Relations Manager	12251	p	Advertising and Public Relations Manager
					12291	p	Office Manager
					33153	p	Sales Representative
1231-11	p	Sales and Marketing Manager	131112	Sales and Marketing Manager	12241	p	Sales and/or Marketing Manager
					12251	p	Advertising and Public Relations Manager
3292-11	p	Project or Program Administrator	132111	Corporate Services Manager	12222	p	Administration Manager
					12224	p	Finance Manager
1211-11		Finance Manager	132211	Finance Manager	12222	p	Administration Manager
					12224	p	Finance Manager
					24111	p	Accountant
1213-11		Human Resource Manager	132311	Human Resource Manager	12222	p	Administration Manager
					12231	p	Human Resources Manager
					24121	p	Human Resources Officer
					24122	p	Training and Development Officer
1291-11		Policy and Planning Manager	132411	Policy and Planning Manager	12221	p	Health Services Manager
					12222	p	Administration Manager
					12291	p	Office Manager
					24133	p	Financial Adviser
					24135	p	Management Consultant
1299-11		Research and Development Manager	132511	Research and Development Manager	12281	p	Research and Development Manager
					24421	p	Social Scientist
1191-11		Construction Project Manager	133111	Construction Project Manager	12218	p	Construction Manager
3121-11	p	Building Associate					
1191-13		Project Builder	133112	Project Builder	12218	p	Construction Manager
					71122	p	Builder (including Contractor)
1221-11		Engineering Manager	133211	Engineering Manager	12219		Engineering Technical Manager
1192-11		Importer or Exporter	133311	Importer or Exporter	12261	p	Supply and Distribution Manager
1192-13		Wholesaler	133312	Wholesaler	12262	p	Wholesale and Warehouse Manager
1193-11		Manufacturer	133411	Manufacturer	12213	p	Production Manager (Manufacturing)
2114-13	p	Forester	133511	Production Manager (Forestry)	12215	p	Forest Manager
1222-11		Production Manager (Manufacturing)	133512	Production Manager (Manufacturing)	12213	p	Production Manager (Manufacturing)
1222-13		Production Manager (Mining)	133513	Production Manager (Mining)	12216		Quarry Manager
1223-11		Supply and Distribution Manager	133611	Supply and Distribution Manager	12214	p	Transport Manager
					12261	p	Supply and Distribution Manager
1295-11		Child Care Co-ordinator	134111	Child Care Centre Manager	12222	p	Administration Manager
1292-13	p	Medical Administrator	134211	Medical Administrator (Aus) / Medical Superintendent (NZ)	12221	p	Health Services Manager
1292-11		Director of Nursing	134212	Nursing Clinical Director	12221	p	Health Services Manager
1292-13	p	Medical Administrator	134213	Primary Health Organisation Manager	12221	p	Health Services Manager
1299-15		Welfare Centre Manager	134214	Welfare Centre Manager	12222	p	Administration Manager
1112-11	p	General Manager	134299	Health and Welfare Services Managers nec	12221	p	Health Services Manager
1292-13	p	Medical Administrator			12291	p	Office Manager
3329-11	p	Other Hospitality and Accommodation Managers					
1293-11		School Principal	134311	School Principal	12211	p	Senior Education Manager
1293-13		Faculty Head	134411	Faculty Head	12211	p	Senior Education Manager
1293-15		Regional Education Manager	134412	Regional Education Manager	12211	p	Senior Education Manager
1293-79		Education Managers nec	134499	Education Managers nec	12211	p	Senior Education Manager
					12222	p	Administration Manager
1224-11	p	Information Technology Manager	135111	Chief Information Officer	12222	p	Administration Manager
					12271	p	Information Technology Manager
1224-11	p	Information Technology Manager	135112	ICT Project Manager	12271	p	Information Technology Manager
2231-13	p	Systems Designer			21313	p	Systems Manager
2231-15	p	Software Designer					
1224-11	p	Information Technology Manager	135199	ICT Managers nec	12271	p	Information Technology Manager
3399-79	p	Managing Supervisors (Sales and Service) nec					
1294-11		Commissioned Defence Force Officer	139111	Commissioned Defence Force Officer	51551	p	Armed Forces
1294-81		Trainee Commissioned Defence Force Officer					
1294-13		Commissioned Fire Officer	139112	Commissioned Fire Officer	51511	p	Fire Fighter
1294-15		Commissioned Police Officer	139113	Commissioned Police Officer	51521	p	Detective
					51522	p	Police Officer
3994-11		Senior Non-Commissioned Defence Force Officer	139211	Senior Non-commissioned Defence Force Member	51551	p	Armed Forces
1299-79	p	Specialist Managers nec	139911	Arts Administrator or Manager	12212	p	Broadcasting and Theatrical Production Manager
1299-17		Environment, Parks and Land Care Manager	139912	Environmental Manager	12222	p	Administration Manager
					12281	p	Research and Development Manager
1299-13		Laboratory Manager	139913	Laboratory Manager	12222	p	Administration Manager
2294-15	p	Quality Assurance Manager	139914	Quality Assurance Manager	12282		Quality Assurance Manager

ASCO Second Edition			ANZSCO		NZSCO 1999		
1299-19		Sports Administrator	139915	Sports Administrator	11411	p	Special-Interest Organisation Administrator
					12222	p	Administration Manager
1111-79	p	Legislators and Government Appointed Officials nec	139999	Specialist Managers nec	11213		Diplomatic Representative
1299-79	p	Specialist Managers nec			11411	p	Special-Interest Organisation Administrator
					12214	p	Transport Manager
					12222	p	Administration Manager
					12291	p	Office Manager
					24312	p	Art Gallery and/or Museum Curator
					24461	p	Diplomatic Official
					24511	p	Minister of Religion
					31423	p	Other Ships' Deck Officer and Pilot
3321-11		Restaurant and Catering Manager	141111	Cafe or Restaurant Manager	12265	p	Restaurant or Tavern Manager
					12267	p	Other Catering Services Manager
3325-11		Caravan Park and Camping Ground Manager	141211	Caravan Park and Camping Ground Manager	12266	p	Other Lodging Services Manager
3323-11		Hotel or Motel Manager	141311	Hotel or Motel Manager	12264		Hotel or Motel Manager
					12265	p	Restaurant or Tavern Manager
3324-11		Club Manager (Licensed Premises)	141411	Licensed Club Manager	12265	p	Restaurant or Tavern Manager
3329-11	p	Other Hospitality and Accommodation Managers	141911	Bed and Breakfast Operator	12266	p	Other Lodging Services Manager
3329-11	p	Other Hospitality and Accommodation Managers	141999	Accommodation and Hospitality Managers nec	12222	p	Administration Manager
3399-79	p	Managing Supervisors (Sales and Service) nec			12266	p	Other Lodging Services Manager
3311-11		Shop Manager	142111	Retail Manager (General)	12241	p	Sales and/or Marketing Manager
					12263	p	Retail Manager
					12267	p	Other Catering Services Manager
3399-79	p	Managing Supervisors (Sales and Service) nec	142112	Antique Dealer	12263	p	Retail Manager
3399-15		Betting Agency Branch Manager	142113	Betting Agency Manager	12263	p	Retail Manager
3399-17		Hair and Beauty Salon Manager	142114	Hair or Beauty Salon Manager	12263	p	Retail Manager
3399-11		Post Office Manager	142115	Post Office Manager	12263	p	Retail Manager
3399-25		Travel Agency Manager	142116	Travel Agency Manager	12263	p	Retail Manager
1112-11	p	General Manager	149111	Amusement Centre Manager	12263	p	Retail Manager
3391-15		Amusement Centre Manager					
3391-79	p	Sports and Recreation Managers nec					
3391-11		Fitness Centre Manager	149112	Fitness Centre Manager	12222	p	Administration Manager
3391-13		Other Sports Centre Manager	149113	Sports Centre Manager	12222	p	Administration Manager
3391-79	p	Sports and Recreation Managers nec					
3392-11	p	Customer Service Manager	149211	Call or Contact Centre Manager	12291	p	Office Manager
					33153	p	Sales Representative
3392-11	p	Customer Service Manager	149212	Customer Service Manager	12222	p	Administration Manager
					12241	p	Sales and/or Marketing Manager
					12251	p	Advertising and Public Relations Manager
					33151	p	Business Services Representative
					33152	p	Technical Representative
3292-11	p	Project or Program Administrator	149311	Conference and Event Organiser	33212	p	Conference/Function Organiser
3399-21		Fleet Manager	149411	Fleet Manager	12214	p	Transport Manager
3399-13		Railway Station Manager	149412	Railway Station Manager	12214	p	Transport Manager
3393-11		Transport Company Manager	149413	Transport Company Manager	12214	p	Transport Manager
3399-19		Car Rental Agency Manager					
3399-79	p	Managing Supervisors (Sales and Service) nec	149911	Boarding Kennel or Cattery Operator	61441	p	Animal Welfare Worker
3399-27		Theatre or Cinema Manager	149912	Cinema or Theatre Manager	12222	p	Administration Manager
3292-11	p	Project or Program Administrator	149913	Facilities Manager	12222	p	Administration Manager
					12223	p	Property Manager
					12291	p	Office Manager
					33211	p	Administration Officer
					33212	p	Conference/Function Organiser
3211-13		Financial Institution Branch Manager	149914	Financial Institution Branch Manager	12224	p	Finance Manager
3399-79	p	Managing Supervisors (Sales and Service) nec	149999	Hospitality, Retail and Service Managers nec	12222	p	Administration Manager
			**2**	**PROFESSIONALS**			
2538-11		Actor	211111	Actor	33661	p	Actor
2538-13		Dancer or Choreographer	211112	Dancer or Choreographer	33651		Dancer
					33652	p	Dancing Teacher and/or Choreographer
					33681	p	Clown, Magician, Acrobat and Related Worker

ASCO Second Edition			ANZSCO		NZSCO 1999		
2538-79	p	Actors, Dancers and Related Professionals nec	211113	Entertainer or Variety Artist	33643	p	Singer
					33681	p	Clown, Magician, Acrobat and Related Worker
2538-79	p	Actors, Dancers and Related Professionals nec	211199	Actors, Dancers and Other Entertainers nec	33661	p	Actor
					33681	p	Clown, Magician, Acrobat and Related Worker
2537-17		Composer	211211	Composer	33641	p	Composer, Arranger and/or Conductor
2537-11		Music Director	211212	Music Director	33641	p	Composer, Arranger and/or Conductor
					33642	p	Instrumentalist
2537-15		Instrumental Musician	211213	Musician (Instrumental)	33642	p	Instrumentalist
2537-13		Singer	211214	Singer	33643	p	Singer
2537-79		Musicians and Related Professionals nec	211299	Music Professionals nec	33642	p	Instrumentalist
2532-11		Photographer	211311	Photographer	31311	p	Photographer
2531-11		Painter (Visual Arts)	211411	Painter (Visual Arts)	33621	p	Sculptor, Painter and Related Artist
2531-15		Potter or Ceramic Artist	211412	Potter or Ceramic Artist	33621	p	Sculptor, Painter and Related Artist
2531-13		Sculptor	211413	Sculptor	33621	p	Sculptor, Painter and Related Artist
2531-79	p	Visual Arts and Crafts Professionals nec	211499	Visual Arts and Crafts Professionals nec	33621	p	Sculptor, Painter and Related Artist
1296-13		Artistic Director	212111	Artistic Director	33662	p	Artistic Director
1296-11		Media Producer	212112	Media Producer (excluding Video)	12212	p	Broadcasting and Theatrical Production Manager
2539-11		Radio Presenter	212113	Radio Presenter	33671	p	Radio and Television Presenter
2539-13		Television Presenter	212114	Television Presenter	33671	p	Radio and Television Presenter
2535-11		Author	212211	Author	33611	p	Author and Critic
2535-13		Book Editor	212212	Book or Script Editor	33613	p	Editor
2535-15		Script Editor			33614	p	Sub-Editor
2536-11		Art Director (Film, Television or Stage)	212311	Art Director (Film, Television or Stage)	33662	p	Artistic Director
2536-13		Director (Film, Television, Radio or Stage)	212312	Director (Film, Television, Radio or Stage)	33662	p	Artistic Director
2536-15		Director of Photography	212313	Director of Photography	33662	p	Artistic Director
2536-17		Film and Video Editor	212314	Film and Video Editor	31321	p	Broadcasting Transmitting and Studio Equipment Operator
2536-21		Program Director (Radio or Television)	212315	Program Director (Television or Radio)	33662	p	Artistic Director
2536-19		Stage Manager	212316	Stage Manager	33662	p	Artistic Director
2536-23		Technical Director	212317	Technical Director	33662	p	Artistic Director
2536-79	p	Film, Television, Radio and Stage Directors nec	212318	Video Producer	12212	p	Broadcasting and Theatrical Production Manager
2536-79	p	Film, Television, Radio and Stage Directors nec	212399	Film, Television, Radio and Stage Directors nec	12212	p	Broadcasting and Theatrical Production Manager
2534-19		Copywriter	212411	Copywriter	33615		Copywriter
2534-11		Editor	212412	Newspaper or Periodical Editor	33613	p	Editor
					33614	p	Sub-Editor
2534-13		Print Journalist	212413	Print Journalist	33612	p	Reporter
2534-17		Radio Journalist	212414	Radio Journalist	33612	p	Reporter
2534-21		Technical Writer	212415	Technical Writer	33611	p	Author and Critic
2534-15		Television Journalist	212416	Television Journalist	33612	p	Reporter
2534-79		Journalists and Related Professionals nec	212499	Journalists and Other Writers nec	33611	p	Author and Critic
					33612	p	Reporter
					33613	p	Editor
					33614	p	Sub-Editor
2211-11	p	Accountant	221111	Accountant (General)	12224	p	Finance Manager
3211-11		Branch Accountant (Financial Institution)			24111	p	Accountant
					24133	p	Financial Adviser
2211-11	p	Accountant	221112	Management Accountant	24111	p	Accountant
2211-11	p	Accountant	221113	Taxation Accountant	24111	p	Accountant
1212-11		Company Secretary	221211	Company Secretary	12222	p	Administration Manager
2213-11		Corporate Treasurer	221212	Corporate Treasurer	12224	p	Finance Manager
2212-11		External Auditor	221213	External Auditor	12222	p	Administration Manager
					12224	p	Finance Manager
					24113	p	Auditor
2212-13		Internal Auditor	221214	Internal Auditor	12222	p	Administration Manager
					24113	p	Auditor
3212-17	p	Commodities Trader	222111	Commodities Trader	33111	p	Financial Dealer and Broker
					33161	p	Wholesale and/or Retail Buyer
3212-79	p	Financial Dealers and Brokers nec	222112	Finance Broker	12224	p	Finance Manager
					24133	p	Financial Adviser
					33111	p	Financial Dealer and Broker
3212-19	p	Insurance Broker	222113	Insurance Broker	12224	p	Finance Manager
					33111	p	Financial Dealer and Broker
3212-79	p	Financial Dealers and Brokers nec	222199	Financial Brokers nec	33111	p	Financial Dealer and Broker
3212-15		Financial Market Dealer	222211	Financial Market Dealer	33111	p	Financial Dealer and Broker

ASCO Second Edition			ANZSCO		NZSCO 1999		
3212-13		Futures Trader	222212	Futures Trader	33111	p	Financial Dealer and Broker
3212-11		Stockbroking Dealer	222213	Stockbroking Dealer	33111	p	Financial Dealer and Broker
3212-79	p	Financial Dealers and Brokers nec	222299	Financial Dealers nec	12251	p	Advertising and Public Relations Manager
					33111	p	Financial Dealer and Broker
3213-11		Financial Investment Adviser	222311	Financial Investment Adviser	12224	p	Finance Manager
					24133	p	Financial Adviser
3212-79	p	Financial Dealers and Brokers nec	222312	Financial Investment Manager	33111	p	Financial Dealer and Broker
2291-11	p	Personnel Officer	223111	Human Resource Adviser	12231	p	Human Resources Manager
2291-13	p	Personnel Consultant			24121	p	Human Resources Officer
2291-11	p	Personnel Officer	223112	Recruitment Consultant	21311	p	Systems Analyst
2291-13	p	Personnel Consultant			24121	p	Human Resources Officer
3399-79	p	Managing Supervisors (Sales and Service) nec			33151	p	Business Services Representative
					33511	p	Careers, Transition, Employment Adviser
2291-15		Industrial Relations Officer	223113	Workplace Relations Adviser	11411	p	Special-Interest Organisation Administrator
					24121	p	Human Resources Officer
					24231	p	Other Legal Professional
2491-79	p	Extra-Systemic Teachers nec	223211	ICT Trainer	24122	p	Training and Development Officer
2291-17	p	Training Officer	223311	Training and Development Professional	21312	p	Computer Applications Engineer
					23111	p	University and Higher Education Lecturer and/or Tutor
					24122	p	Training and Development Officer
2293-15		Actuary	224111	Actuary	21211	p	Mathematician and/or Statistician
2293-11		Mathematician	224112	Mathematician	21211	p	Mathematician and/or Statistician
2293-13		Statistician	224113	Statistician	21211	p	Mathematician and/or Statistician
					24421	p	Social Scientist
2299-15		Archivist	224211	Archivist	24311	p	Archivist
2549-21		Museum or Gallery Curator	224212	Gallery or Museum Curator	24312	p	Art Gallery and/or Museum Curator
2299-11		Health Information Manager	224213	Health Information Manager	24322	p	Information Services Administrator
2299-13		Records Manager	224214	Records Manager	24322	p	Information Services Administrator
					41412	p	Record and Filing Clerk
2522-11		Economist	224311	Economist	24411		Economist
2299-19		Intelligence Officer	224411	Intelligence Officer	24421	p	Social Scientist
					24422	p	Policy Analyst
2299-17		Policy Analyst	224412	Policy Analyst	24421	p	Social Scientist
					24422	p	Policy Analyst
					42213	p	Information Clerk and Other Receptionist
2295-13		Land Economist	224511	Land Economist	12223	p	Property Manager
2295-11		Valuer	224512	Valuer	33171		Valuer
2292-11		Librarian	224611	Librarian	12222	p	Administration Manager
					12271	p	Information Technology Manager
					24321		Librarian
					41411	p	Library Assistant
2294-11		Management Consultant	224711	Management Consultant	12222	p	Administration Manager
					21311	p	Systems Analyst
					24113	p	Auditor
					24121	p	Human Resources Officer
					24133	p	Financial Adviser
					24135	p	Management Consultant
					41222	p	Statistical Clerk
					41443	p	General Clerk
2294-13		Organisation and Methods Analyst	224712	Organisation and Methods Analyst	12222	p	Administration Manager
2294-15	p	Quality Assurance Manager			21311	p	Systems Analyst
					24133	p	Financial Adviser
					33241	p	Organisation and Methods Analyst
2549-13		Electorate Officer	224911	Electorate Officer	12291	p	Office Manager
2299-79	p	Business and Information Professionals nec	224912	Liaison Officer	24121	p	Human Resources Officer
					24132	p	Public Relations Officer
					24461	p	Diplomatic Official
2299-79	p	Business and Information Professionals nec	224913	Migration Agent (Aus) / Immigration Consultant (NZ)	24135	p	Management Consultant
2549-15		Patents Examiner	224914	Patents Examiner	24231	p	Other Legal Professional
2299-79	p	Business and Information Professionals nec	224999	Information and Organisation Professionals nec	22135		Land Management Officer
2549-79	p	Professionals nec			33211	p	Administration Officer
3999-79	p	Associate Professionals nec			33241	p	Organisation and Methods Analyst
2221-17		Advertising Specialist	225111	Advertising Specialist	12251	p	Advertising and Public Relations Manager
					24132	p	Public Relations Officer
					33151	p	Business Services Representative
2221-15		Market Research Analyst	225112	Market Research Analyst	24131	p	Market Research Analyst

ASCO Second Edition			ANZSCO		NZSCO 1999		
2221-13		Marketing Specialist	225113	Marketing Specialist	12241	p	Sales and/or Marketing Manager
					24131	p	Market Research Analyst
					33153	p	Sales Representative
1231-11	p	Sales and Marketing Manager	225211	ICT Account Manager	12241	p	Sales and/or Marketing Manager
2222-13	p	Sales Representative (Information and Communication Products)					
3392-11	p	Customer Service Manager					
1231-11	p	Sales and Marketing Manager	225212	ICT Business Development Manager	12271	p	Information Technology Manager
					33151	p	Business Services Representative
2222-13	p	Sales Representative (Information and Communication Products)					
2222-13	p	Sales Representative (Information and Communication Products)	225213	ICT Sales Representative	33152	p	Technical Representative
					33153	p	Sales Representative
2221-11		Public Relations Officer	225311	Public Relations Professional	12251	p	Advertising and Public Relations Manager
					12291	p	Office Manager
					24132	p	Public Relations Officer
2222-11		Sales Representative (Industrial Products)	225411	Sales Representative (Industrial Products)	33153	p	Sales Representative
2222-15		Sales Representative (Medical and Pharmaceutical Products)	225412	Sales Representative (Medical and Pharmaceutical Products)	33153	p	Sales Representative
2222-79		Technical Sales Representatives nec	225499	Technical Sales Representatives nec	33151	p	Business Services Representative
					33152	p	Technical Representative
2541-11	p	Aircraft Pilot	231111	Aeroplane Pilot	31431	p	Aircraft Pilot and Flight Crew
					51551	p	Armed Forces
2541-13		Air Traffic Controller	231112	Air Traffic Controller	31441		Air Traffic Controller
2541-15		Flight Service Officer					
2541-19		Flying Instructor	231113	Flying Instructor	31433		Flying Instructor
2541-11	p	Aircraft Pilot	231114	Helicopter Pilot	31434		Helicopter Pilot
2541-11	p	Aircraft Pilot	231199	Air Transport Professionals nec	31431	p	Aircraft Pilot and Flight Crew
2541-17		Flight Engineer			51551	p	Armed Forces
2541-79		Air Transport Professionals nec					
3999-79	p	Associate Professionals nec					
4999-15		Aircraft Safety Equipment Worker					
2542-13		Master Fisher	231211	Master Fisher	61411	p	Fishing Skipper, Fisherperson
2542-15		Ship's Engineer	231212	Ship's Engineer	31411		Ships' Engineer
2542-11		Ship's Master	231213	Ship's Master	31421	p	Ships' Officer (Deck) including Master
					31422		Launch Master
					31423	p	Other Ships' Deck Officer and Pilot
					83412	p	Other Ship or Boat Hand
2542-19		Ship's Officer	231214	Ship's Officer	31421	p	Ships' Officer (Deck) including Master
					31423	p	Other Ships' Deck Officer and Pilot
2542-17		Ship's Surveyor	231215	Ship's Surveyor	21452	p	Naval Architect and/or Ships' Surveyor
2542-79		Sea Transport Professionals nec	231299	Marine Transport Professionals nec	31423	p	Other Ships' Deck Officer and Pilot
3999-79	p	Associate Professionals nec					
2121-11		Architect	232111	Architect	21411		Architect
2121-13		Landscape Architect	232112	Landscape Architect	21413		Landscape Architect
2123-11		Cartographer	232211	Cartographer	21483	p	Cartographer and Photogrammetrist
2123-13		Surveyor	232212	Surveyor	21481		Surveyor
					21483	p	Cartographer and Photogrammetrist
2533-11		Fashion Designer	232311	Fashion Designer	33632		Fashion Designer
					33634	p	Industrial Designer
2533-15		Industrial Designer	232312	Industrial Designer	33634	p	Industrial Designer
2531-79	p	Visual Arts and Crafts Professionals nec	232313	Jewellery Designer	33621	p	Sculptor, Painter and Related Artist
					73131	p	Jeweller and Jewellery Repairer
2533-13	p	Graphic Designer	232411	Graphic Designer	33631	p	Graphic Designer
2533-19		Illustrator	232412	Illustrator	33631	p	Graphic Designer
2533-13	p	Graphic Designer	232413	Multimedia Designer	33631	p	Graphic Designer
2533-13	p	Graphic Designer	232414	Web Designer	21312	p	Computer Applications Engineer
					33631	p	Graphic Designer
2533-17		Interior Designer	232511	Interior Designer	33636	p	Interior Designer
2523-11		Urban and Regional Planner	232611	Urban and Regional Planner	21412		Resource Management Planner
2129-17	p	Chemical Engineer	233111	Chemical Engineer	21461	p	Chemical Engineer
2127-15		Materials Engineer	233112	Materials Engineer	31191	p	Other Engineering Technician
2124-11	p	Civil Engineer	233211	Civil Engineer	21422		Water Resources Engineer
					21423	p	Public Health Engineer
					21426	p	Other Civil Engineer
2124-11	p	Civil Engineer	233212	Geotechnical Engineer	21426	p	Other Civil Engineer
2122-11		Quantity Surveyor	233213	Quantity Surveyor	31121	p	Quantity Surveyor
2124-11	p	Civil Engineer	233214	Structural Engineer	21425		Structural Engineer
2124-11	p	Civil Engineer	233215	Transport Engineer	21421		Roading Engineer
2125-11		Electrical Engineer	233311	Electrical Engineer	21431		Electrical Engineer
2125-13	p	Electronics Engineer	233411	Electronics Engineer	21441	p	Electronic and Telecommunications Engineer

ASCO Second Edition			ANZSCO		NZSCO 1999		
2129-19		Industrial Engineer	233511	Industrial Engineer	21455	p	Other Mechanical Engineer
2126-11		Mechanical Engineer	233512	Mechanical Engineer	21451		Heating, Ventilation and Refrigeration Engineer
					21455	p	Other Mechanical Engineer
2125-13	p	Electronics Engineer	233513	Production or Plant Engineer	21455	p	Other Mechanical Engineer
2126-13		Production or Plant Engineer					
2127-11		Mining Engineer (excluding Petroleum)	233611	Mining Engineer (excluding Petroleum)	21472	p	Mining Engineer
2127-13		Petroleum Engineer	233612	Petroleum Engineer	21461	p	Chemical Engineer
					21472	p	Mining Engineer
2129-11		Aeronautical Engineer	233911	Aeronautical Engineer	21453	p	Aeronautical Engineer and/or Aircraft Surveyor
2129-13		Agricultural Engineer	233912	Agricultural Engineer	21454		Agricultural Engineer
2129-15		Biomedical Engineer	233913	Biomedical Engineer	21423	p	Public Health Engineer
					22122	p	Microbiologist
2128-11		Civil Engineering Technologist	233914	Engineering Technologist	31151	p	Mechanical Engineering Technician
2128-13		Mechanical Engineering Technologist			31161	p	Chemical Engineering Technician
2128-15		Electrical or Electronics Engineering Technologist			31191	p	Other Engineering Technician
2128-79		Engineering Technologists nec			32111	p	Life Science Technician
2129-17	p	Chemical Engineer	233915	Environmental Engineer	22117	p	Environmental Scientist
2129-21		Naval Architect	233916	Naval Architect (Aus) / Marine Designer (NZ)	21452	p	Naval Architect and/or Ships' Surveyor
					31181	p	Draughting Technician
2125-13	p	Electronics Engineer	233999	Engineering Professionals nec	21455	p	Other Mechanical Engineer
2129-79		Building and Engineering Professionals nec			31191	p	Other Engineering Technician
2549-79	p	Professionals nec					
2114-21		Agricultural Adviser	234111	Agricultural Consultant	22132	p	Agricultural Consultant
					22134		Horticultural Consultant
2114-19		Agricultural Scientist	234112	Agricultural Scientist	22114	p	Agronomist
					22115	p	Horticultural Scientist
2114-13	p	Forester	234113	Forester (Aus) / Forest Scientist (NZ)	12215	p	Forest Manager
					22116		Forestry Scientist
					22132	p	Agricultural Consultant
2111-11	p	Chemist	234211	Chemist	21131	p	Chemist (other than Pharmacist)
2111-11	p	Chemist	234212	Food Technologist	21461	p	Chemical Engineer
2549-17		Oenologist	234213	Wine Maker	61122	p	Grape Grower and/or Wine Maker, Worker
2549-79	p	Professionals nec					
2114-79	p	Environmental and Agricultural Science Professionals nec	234311	Conservation Officer	22133		Conservation Officer
2114-79	p	Environmental and Agricultural Science Professionals nec	234312	Environmental Consultant	22117	p	Environmental Scientist
2114-11		Environmental Research Scientist	234313	Environmental Research Scientist	22117	p	Environmental Scientist
					22131	p	Soil Scientist
2114-15		Park Ranger	234314	Park Ranger	33811		Acclimatisation Field Officer
					33812		National Park Ranger
					51542	p	Security Officer
2114-17		Soil Scientist	234399	Environmental Scientists nec	22117	p	Environmental Scientist
2114-79	p	Environmental and Agricultural Science Professionals nec			22131	p	Soil Scientist
2112-11		Geologist	234411	Geologist	21141		Geologist
2112-13		Geophysicist	234412	Geophysicist	21142		Geophysicist
2113-00		Life Scientists, nfd	234511	Life Scientist (General)	22111	p	Biologist
2113-11		Anatomist or Physiologist	234512	Anatomist or Physiologist	22113	p	Zoologist
					22121	p	Biochemist
2113-17	p	Biochemist	234513	Biochemist	22121	p	Biochemist
2113-17	p	Biochemist	234514	Biotechnologist	21461	p	Chemical Engineer
2113-79	p	Life Scientists nec			22111	p	Biologist
					22121	p	Biochemist
					32112	p	Medical Laboratory Technician
2113-13		Botanist	234515	Botanist	22112		Botanist
2113-19		Marine Biologist	234516	Marine Biologist	22111	p	Biologist
2113-79	p	Life Scientists nec	234517	Microbiologist	22122	p	Microbiologist
2113-15		Zoologist	234518	Zoologist	22113	p	Zoologist
2113-79	p	Life Scientists nec	234599	Life Scientists nec	22113	p	Zoologist
					22114	p	Agronomist
					22115	p	Horticultural Scientist
					22121	p	Biochemist
					22122	p	Microbiologist
2115-11	p	Medical Scientist	234611	Medical Laboratory Scientist	22121	p	Biochemist
					22122	p	Microbiologist
2392-11		Veterinarian	234711	Veterinarian	22231		Veterinarian
2549-11		Conservator	234911	Conservator	24311	p	Archivist
					24312	p	Art Gallery and/or Museum Curator

ASCO Second Edition			ANZSCO		NZSCO 1999		
2119-15		Extractive Metallurgist	234912	Metallurgist	21471		Metallurgist
2119-17		Physical Metallurgist					
2119-13		Meteorologist	234913	Meteorologist	21121		Meteorologist
2115-11	p	Medical Scientist	234914	Physicist	21111	p	Physicist
2119-11		Physicist					
2119-19		Materials Scientist	234999	Natural and Physical Science Professionals nec	21111	p	Physicist
2119-79		Natural and Physical Science Professionals nec			21461	p	Chemical Engineer
					22111	p	Biologist
2411-11	p	Pre-Primary School Teacher	241111	Early Childhood (Pre-primary School) Teacher	23321	p	Early Childhood Teacher
2411-11	p	Pre-Primary School Teacher	241112	Kaiako Kōhanga Reo (Māori Language Nest Teacher)	23322		Kōhanga Reo Teacher
2412-11	p	Primary School Teacher	241211	Kaiako Kura Kaupapa Māori (Māori-medium Primary School Teacher)	23311	p	Primary School Teacher
2412-11	p	Primary School Teacher	241212	Pouako Kura Kaupapa Māori (Māori-medium Primary School Senior Teacher)	23311	p	Primary School Teacher
2412-11	p	Primary School Teacher	241213	Primary School Teacher	23311	p	Primary School Teacher
2412-11	p	Primary School Teacher	241311	Middle School Teacher (Aus) / Intermediate School Teacher (NZ)	23311	p	Primary School Teacher
2413-11		Secondary School Teacher	241411	Secondary School Teacher	23211		Secondary School Teacher
2414-11		Special Needs Teacher	241511	Special Needs Teacher	23411	p	Special Education Teacher
2414-13		Teacher of the Hearing Impaired	241512	Teacher of the Hearing Impaired	23411	p	Special Education Teacher
2414-15		Teacher of the Sight Impaired	241513	Teacher of the Sight Impaired	23411	p	Special Education Teacher
2414-79		Special Education Teachers nec	241599	Special Education Teachers nec	23411	p	Special Education Teacher
2421-11		University Lecturer	242111	University Lecturer	23111	p	University and Higher Education Lecturer and/or Tutor
2421-13		University Tutor	242112	University Tutor	23111	p	University and Higher Education Lecturer and/or Tutor
2422-11		Vocational Education Teacher	242211	Vocational Education Teacher (Aus) / Polytechnic Teacher (NZ)	23111	p	University and Higher Education Lecturer and/or Tutor
2493-11	p	Education Officer	249111	Education Adviser	23511		Education Adviser
					23521	p	Education Reviewer
					24132	p	Public Relations Officer
					24312	p	Art Gallery and/or Museum Curator
2493-11	p	Education Officer	249112	Education Reviewer	23521	p	Education Reviewer
2491-11		Art Teacher (Private)	249211	Art Teacher (Private Tuition)	33621	p	Sculptor, Painter and Related Artist
2491-15		Dance Teacher (Private)	249212	Dance Teacher (Private Tuition)	33652	p	Dancing Teacher and/or Choreographer
2491-17		Drama Teacher (Private)	249213	Drama Teacher (Private Tuition)	23111	p	University and Higher Education Lecturer and/or Tutor
					23412	p	Speech-Language Therapist
2491-13		Music Teacher (Private)	249214	Music Teacher (Private Tuition)	33644		Singing and Music Teacher
2491-79	p	Extra-Systemic Teachers nec	249299	Private Tutors and Teachers nec	23111	p	University and Higher Education Lecturer and/or Tutor
2492-11		English as a Second Language Teacher	249311	Teacher of English to Speakers of Other Languages	23413		Teacher of English to Speakers of Other Languages
2393-11		Dietitian	251111	Dietitian	22251		Dietitian and Public Health Nutritionist
2391-11		Medical Diagnostic Radiographer	251211	Medical Diagnostic Radiographer	31331	p	Medical Radiation Technologist
2391-13		Radiation Therapist	251212	Medical Radiation Therapist	22216	p	Radiologist, Radiation Oncologist
					31331	p	Medical Radiation Technologist
2391-15		Nuclear Medicine Technologist	251213	Nuclear Medicine Technologist	31331	p	Medical Radiation Technologist
2391-17		Sonographer	251214	Sonographer	31333		Sonographer
2543-13		Environmental Health Officer	251311	Environmental Health Officer	31514	p	Health Inspector
2543-11		Occupational Health and Safety Officer	251312	Occupational Health and Safety Adviser	31511	p	Safety Inspector
2384-11		Optometrist	251411	Optometrist	22261	p	Optometrist
2399-13		Orthoptist	251412	Orthoptist	22261	p	Optometrist
2382-11		Hospital Pharmacist	251511	Hospital Pharmacist	22241		Hospital Pharmacist
2382-13		Industrial Pharmacist	251512	Industrial Pharmacist	21131	p	Chemist (other than Pharmacist)
2382-15		Retail Pharmacist	251513	Retail Pharmacist	22242		Retail Pharmacist
2512-13	p	Community Worker	251911	Health Promotion Officer	31514	p	Health Inspector
					32312	p	Karitane Nurse
					33411	p	Social Worker
2399-15		Orthotist	251912	Orthotist or Prosthetist	32234	p	Orthotist and/or Prosthetist
2399-79	p	Health Professionals nec	251999	Health Diagnostic and Promotion Professionals nec	22214	p	Physician
					24451	p	Counsellor
2387-11		Chiropractor	252111	Chiropractor	32236		Chiropractor
2387-13		Osteopath	252112	Osteopath	32233		Osteopath
2394-13		Acupuncturist	252211	Acupuncturist	32261	p	Other Health Associate Professional
2394-79	p	Natural Therapy Professionals nec	252212	Homoeopath	32261	p	Other Health Associate Professional
2394-11		Naturopath	252213	Naturopath	32261	p	Other Health Associate Professional
2394-79	p	Natural Therapy Professionals nec	252214	Traditional Chinese Medicine Practitioner	32261	p	Other Health Associate Professional

ASCO Second Edition			ANZSCO		NZSCO 1999		
2394-79	p	Natural Therapy Professionals nec	252215	Traditional Māori Health Practitioner	22211	p	General Practitioner
2394-79	p	Natural Therapy Professionals nec	252299	Complementary Health Therapists nec	32261	p	Other Health Associate Professional
2399-79	p	Health Professionals nec					
2381-13		Dental Specialist	252311	Dental Specialist	22221	p	Dentist and Dental Surgeon
2381-11		Dentist	252312	Dentist	22221	p	Dentist and Dental Surgeon
2383-11		Occupational Therapist	252411	Occupational Therapist	32232	p	Occupational Therapist
2385-11		Physiotherapist	252511	Physiotherapist	32231	p	Physiotherapist
2388-11		Podiatrist	252611	Podiatrist	22213	p	Surgeon
					32235		Podiatrist
2399-11		Audiologist	252711	Audiologist	32261	p	Other Health Associate Professional
2386-11		Speech Pathologist	252712	Speech Pathologist (Aus) / Speech Language Therapist (NZ)	23412	p	Speech-Language Therapist
2311-11		General Medical Practitioner	253111	General Medical Practitioner	22211	p	General Practitioner
2311-81		Medical Practitioner in Training	253112	Resident Medical Officer	22212	p	Resident Medical Officer
2312-11		Anaesthetist	253211	Anaesthetist	22217		Anaesthetist
2312-25	p	Specialist Physician	253311	Specialist Physician (General Medicine)	22214	p	Physician
2312-25	p	Specialist Physician	253312	Cardiologist	22214	p	Physician
2312-25	p	Specialist Physician	253313	Clinical Haematologist	22123	p	Medical Pathologist
2312-25	p	Specialist Physician	253314	Clinical Oncologist	22214	p	Physician
2312-25	p	Specialist Physician	253315	Endocrinologist	22214	p	Physician
2312-25	p	Specialist Physician	253316	Gastroenterologist	22214	p	Physician
2312-25	p	Specialist Physician	253317	Intensive Care Specialist	22213	p	Surgeon
2312-25	p	Specialist Physician	253318	Neurologist	22214	p	Physician
2312-21		Paediatrician	253321	Paediatrician	22214	p	Physician
2312-25	p	Specialist Physician	253322	Renal Medicine Specialist	22214	p	Physician
2312-25	p	Specialist Physician	253323	Rheumatologist	22214	p	Physician
2312-25	p	Specialist Physician	253324	Thoracic Medicine Specialist	22214	p	Physician
2312-25	p	Specialist Physician	253399	Internal Medicine Specialists nec	22214	p	Physician
2312-27		Psychiatrist	253411	Psychiatrist	22214	p	Physician
2312-31	p	Surgeon	253511	Surgeon (General)	22213	p	Surgeon
2312-31	p	Surgeon	253512	Cardiothoracic Surgeon	22213	p	Surgeon
2312-31	p	Surgeon	253513	Neurosurgeon	22213	p	Surgeon
2312-31	p	Surgeon	253514	Orthopaedic Surgeon	22213	p	Surgeon
2312-31	p	Surgeon	253515	Otorhinolaryngologist	22214	p	Physician
2312-31	p	Surgeon	253516	Paediatric Surgeon	22213	p	Surgeon
2312-31	p	Surgeon	253517	Plastic and Reconstructive Surgeon	22213	p	Surgeon
2312-31	p	Surgeon	253518	Urologist	22213	p	Surgeon
2312-31	p	Surgeon	253521	Vascular Surgeon	22213	p	Surgeon
2312-13		Dermatologist	253911	Dermatologist	22214	p	Physician
2312-15		Emergency Medicine Specialist	253912	Emergency Medicine Specialist	22214	p	Physician
2312-17		Obstetrician and Gynaecologist	253913	Obstetrician and Gynaecologist	22215		Gynaecologist and Obstetrician
2312-19		Ophthalmologist	253914	Ophthalmologist	22214	p	Physician
2312-23		Pathologist	253915	Pathologist	22123	p	Medical Pathologist
					22214	p	Physician
2312-29		Radiologist	253916	Radiologist	22214	p	Physician
					22216	p	Radiologist, Radiation Oncologist
2312-79		Specialist Medical Practitioners nec	253999	Medical Practitioners nec	22214	p	Physician
2324-11		Registered Midwife	254111	Midwife	22317	p	Midwife
2322-11		Nurse Educator	254211	Nurse Educator	23111	p	University and Higher Education Lecturer and/or Tutor
2322-13		Nurse Researcher	254212	Nurse Researcher	22312	p	Registered Nurse
2321-11		Nurse Manager	254311	Nurse Manager	22311		Principal Nurse
					22312	p	Registered Nurse
2323-11	p	Registered Nurse	254411	Nurse Practitioner	22312	p	Registered Nurse
2323-11	p	Registered Nurse	254412	Registered Nurse (Aged Care)	22312	p	Registered Nurse
2323-11	p	Registered Nurse	254413	Registered Nurse (Child and Family Health)	22312	p	Registered Nurse
					22314		Plunket Nurse
					22317	p	Midwife
2323-11	p	Registered Nurse	254414	Registered Nurse (Community Health)	22315		Public Health and District Nurse
					22316		Occupational Health Nurse
2323-11	p	Registered Nurse	254415	Registered Nurse (Critical Care and Emergency)	22312	p	Registered Nurse
2326-11		Registered Developmental Disability Nurse	254416	Registered Nurse (Developmental Disability)	22312	p	Registered Nurse
2323-11	p	Registered Nurse	254417	Registered Nurse (Disability and Rehabilitation)	22312	p	Registered Nurse
2323-11	p	Registered Nurse	254418	Registered Nurse (Medical)	22312	p	Registered Nurse
2323-11	p	Registered Nurse	254421	Registered Nurse (Medical Practice)	22312	p	Registered Nurse
2325-11		Registered Mental Health Nurse	254422	Registered Nurse (Mental Health)	22313		Psychiatric Nurse
2323-11	p	Registered Nurse	254423	Registered Nurse (Perioperative)	22312	p	Registered Nurse
2323-11	p	Registered Nurse	254424	Registered Nurse (Surgical)	22312	p	Registered Nurse
2323-11	p	Registered Nurse	254499	Registered Nurses nec	22312	p	Registered Nurse

ASCO Second Edition			ANZSCO		NZSCO 1999		
2231-13	p	Systems Designer	261111	ICT Business Analyst	21311	p	Systems Analyst
2231-15	p	Software Designer					
2231-17	p	Applications and Analyst Programmer					
2231-19	p	Systems Programmer					
2231-79	p	Computing Professionals nec					
2231-13	p	Systems Designer	261112	Systems Analyst	21311	p	Systems Analyst
					21312	p	Computer Applications Engineer
2533-13	p	Graphic Designer	261211	Multimedia Specialist	21312	p	Computer Applications Engineer
2231-17	p	Applications and Analyst Programmer	261212	Web Developer	21312	p	Computer Applications Engineer
2533-13	p	Graphic Designer			21313	p	Systems Manager
2231-17	p	Applications and Analyst Programmer	261311	Analyst Programmer	21311	p	Systems Analyst
					31211	p	Computer Programmer
2231-17	p	Applications and Analyst Programmer	261312	Developer Programmer	21311	p	Systems Analyst
2231-19	p	Systems Programmer			21312	p	Computer Applications Engineer
					21313	p	Systems Manager
					31211	p	Computer Programmer
2231-15	p	Software Designer	261313	Software Engineer	21311	p	Systems Analyst
					21312	p	Computer Applications Engineer
					21313	p	Systems Manager
2231-15	p	Software Designer	261399	Software and Applications Programmers nec	21312	p	Computer Applications Engineer
2231-79	p	Computing Professionals nec					
2231-11	p	Systems Manager	262111	Database Administrator	21313	p	Systems Manager
2231-79	p	Computing Professionals nec					
2231-21		Computer Systems Auditor	262112	ICT Security Specialist	21311	p	Systems Analyst
2231-11	p	Systems Manager	262113	Systems Administrator	12271	p	Information Technology Manager
					21312	p	Computer Applications Engineer
					21313	p	Systems Manager
2125-13	p	Electronics Engineer	263111	Computer Network and Systems Engineer	21311	p	Systems Analyst
2231-13	p	Systems Designer			21312	p	Computer Applications Engineer
2231-79	p	Computing Professionals nec					
2231-11	p	Systems Manager	263112	Network Administrator	21312	p	Computer Applications Engineer
2231-79	p	Computing Professionals nec			21313	p	Systems Manager
2231-11	p	Systems Manager	263113	Network Analyst	21311	p	Systems Analyst
2231-13	p	Systems Designer			21312	p	Computer Applications Engineer
2294-15	p	Quality Assurance Manager	263211	ICT Quality Assurance Engineer	21312	p	Computer Applications Engineer
3294-11	p	Computing Support Technician	263212	ICT Support Engineer	21312	p	Computer Applications Engineer
2231-79	p	Computing Professionals nec	263213	ICT Systems Test Engineer	21312	p	Computer Applications Engineer
					21313	p	Systems Manager
2231-79	p	Computing Professionals nec	263299	ICT Support and Test Engineers nec	21312	p	Computer Applications Engineer
					21313	p	Systems Manager
2125-13	p	Electronics Engineer	263311	Telecommunications Engineer	21441	p	Electronic and Telecommunications Engineer
2125-13	p	Electronics Engineer	263312	Telecommunications Network Engineer	21441	p	Electronic and Telecommunications Engineer
2231-13	p	Systems Designer					
2521-11		Barrister	271111	Barrister	24211	p	Barrister and Solicitor
1111-13		Judge	271211	Judge	24221	p	Judge
1111-15		Magistrate	271212	Magistrate	24221	p	Judge
1111-17		Tribunal Member	271213	Tribunal Member	24231	p	Other Legal Professional
1111-79	p	Legislators and Government Appointed Officials nec	271299	Judicial and Other Legal Professionals nec	24231	p	Other Legal Professional
1299-79	p	Specialist Managers nec			33222	p	Legal Clerk
2521-79		Legal Professionals nec					
2521-13		Solicitor	271311	Solicitor	24211	p	Barrister and Solicitor
2513-17		Careers Counsellor	272111	Careers Counsellor	24451	p	Counsellor
					33511	p	Careers, Transition, Employment Adviser
2513-13		Drug and Alcohol Counsellor	272112	Drug and Alcohol Counsellor	24451	p	Counsellor
2513-15		Family Counsellor	272113	Family and Marriage Counsellor	24451	p	Counsellor
2513-11		Rehabilitation Counsellor	272114	Rehabilitation Counsellor	24451	p	Counsellor
2513-19		Student Counsellor	272115	Student Counsellor	24451	p	Counsellor
2513-79		Counsellors nec	272199	Counsellors nec	24451	p	Counsellor
2515-11		Minister of Religion	272211	Minister of Religion	24511	p	Minister of Religion
					33711		Non-ordained Religious Assistant
2514-11	p	Clinical Psychologist	272311	Clinical Psychologist	22212	p	Resident Medical Officer
					24441	p	Psychologist
2514-13		Educational Psychologist	272312	Educational Psychologist	24441	p	Psychologist
2514-15		Organisational Psychologist	272313	Organisational Psychologist	24441	p	Psychologist
2514-11	p	Clinical Psychologist	272314	Psychotherapist	22214	p	Physician
					24441	p	Psychologist
					24442		Psychotherapist
2514-79		Psychologists nec	272399	Psychologists nec	24441	p	Psychologist
2529-11		Historian	272411	Historian	24421	p	Social Scientist

ASCO Second Edition			ANZSCO		NZSCO 1999		
2529-13		Interpreter	272412	Interpreter	24431	p	Philologist, Translator or Interpreter
2529-15		Translator	272413	Translator	24431	p	Philologist, Translator or Interpreter
2529-79		Social Professionals nec	272499	Social Professionals nec	24421	p	Social Scientist
2549-79	p	Professionals nec			24431	p	Philologist, Translator or Interpreter
					41443	p	General Clerk
2511-11		Social Worker	272511	Social Worker	33411	p	Social Worker
2531-79	p	Visual Arts and Crafts Professionals nec	272611	Community Arts Worker	33411	p	Social Worker
2549-79	p	Professionals nec					
2549-19	p	Recreation Officer	272612	Recreation Officer (Aus) / Recreation Coordinator (NZ)	33411	p	Social Worker
2512-11		Welfare Worker	272613	Welfare Worker	33411	p	Social Worker
2549-79	p	Professionals nec			33413	p	Case Worker
			**3**	**TECHNICIANS AND TRADES WORKERS**			
3112-17		Agricultural Technical Officer	311111	Agricultural Technician	32111	p	Life Science Technician
					32121		Agricultural Technician
					61267		Sampling Officer
3111-79	p	Medical Technical Officers nec	311211	Anaesthetic Technician	31332	p	Other Medical Equipment Controller
3111-79	p	Medical Technical Officers nec	311212	Cardiac Technician	31332	p	Other Medical Equipment Controller
3111-11		Medical Laboratory Technical Officer	311213	Medical Laboratory Technician	32112	p	Medical Laboratory Technician
3111-79	p	Medical Technical Officers nec	311214	Operating Theatre Technician	31332	p	Other Medical Equipment Controller
3111-79	p	Medical Technical Officers nec	311215	Pharmacy Technician	31332	p	Other Medical Equipment Controller
					32251		Hospital Dispensary Assistant
					32252	p	Retail Dispensary Assistant
3111-79	p	Medical Technical Officers nec	311299	Medical Technicians nec	31332	p	Other Medical Equipment Controller
3129-11	p	Biomedical Engineering Associate			32234	p	Orthotist and/or Prosthetist
3999-79	p	Associate Professionals nec					
3991-11	p	Primary Products Inspector	311311	Fisheries Officer	31515	p	Agricultural Inspector
3991-11	p	Primary Products Inspector	311312	Meat Inspector	31512		Meat Inspector
3991-11	p	Primary Products Inspector	311313	Quarantine Officer	32111	p	Life Science Technician
					33312	p	Quarantine and Agriculture Ports Officer
3991-11	p	Primary Products Inspector	311399	Primary Products Inspectors nec	31515	p	Agricultural Inspector
3112-11	p	Chemistry Technical Officer	311411	Chemistry Technician	31111	p	Physical Science Technician
					31161	p	Chemical Engineering Technician
3112-13		Earth Science Technical Officer	311412	Earth Science Technician	31111	p	Physical Science Technician
3112-15		Life Science Technical Officer	311413	Life Science Technician	32111	p	Life Science Technician
					32122		Forest Technician
3112-11	p	Chemistry Technical Officer	311414	School Laboratory Technician	31111	p	Physical Science Technician
3112-79		Science Technical Officers nec	311499	Science Technicians nec	31111	p	Physical Science Technician
3121-13		Architectural Associate	312111	Architectural Draftsperson	31181	p	Draughting Technician
3121-11	p	Building Associate	312112	Building Associate	31123	p	Clerk of Works
					31181	p	Draughting Technician
					71121	p	Carpenter and/or Joiner
3121-17		Building Inspector	312113	Building Inspector	31511	p	Safety Inspector
3121-11	p	Building Associate	312114	Construction Estimator	31121	p	Quantity Surveyor
					31123	p	Clerk of Works
3121-19		Plumbing Inspector	312115	Plumbing Inspector	31511	p	Safety Inspector
3121-15		Surveying and Cartographic Associate	312116	Surveying or Cartographic Technician	21483	p	Cartographer and Photogrammetrist
					31122		Surveyor's Technician
3121-21	p	Plumbing Engineering Associate	312211	Civil Engineering Draftsperson	31181	p	Draughting Technician
3122-11	p	Civil Engineering Associate					
3122-13	p	Civil Engineering Technician					
3121-21	p	Plumbing Engineering Associate	312212	Civil Engineering Technician	22131	p	Soil Scientist
3122-11	p	Civil Engineering Associate			31124		Other Civil Engineering Technician
3122-13	p	Civil Engineering Technician					
3123-11	p	Electrical Engineering Associate	312311	Electrical Engineering Draftsperson	31181	p	Draughting Technician
3123-13	p	Electrical Engineering Technician					
3123-11	p	Electrical Engineering Associate	312312	Electrical Engineering Technician	31131	p	Electrical Engineering Technician
3123-13	p	Electrical Engineering Technician					
3124-11	p	Electronic Engineering Associate	312411	Electronic Engineering Draftsperson	31143	p	Other Electronics Engineering Technician
3124-13	p	Electronic Engineering Technician					
3124-11	p	Electronic Engineering Associate	312412	Electronic Engineering Technician	31143	p	Other Electronics Engineering Technician
3124-13	p	Electronic Engineering Technician			31144	p	Avionics Technician
3125-11	p	Mechanical Engineering Associate	312511	Mechanical Engineering Draftsperson	31181	p	Draughting Technician
3125-13	p	Mechanical Engineering Technician					
3125-11	p	Mechanical Engineering Associate	312512	Mechanical Engineering Technician	31151	p	Mechanical Engineering Technician
3125-13	p	Mechanical Engineering Technician					
3992-11	p	Safety Inspector	312611	Safety Inspector	31511	p	Safety Inspector
					33241	p	Organisation and Methods Analyst
3129-79	p	Building and Engineering Associate Professionals nec	312911	Maintenance Planner	31191	p	Other Engineering Technician

ASCO Second Edition			ANZSCO		NZSCO 1999		
3129-13		Metallurgical and Materials Technician	312912	Metallurgical or Materials Technician	31191	p	Other Engineering Technician
					31192		Non-Destructive Testing Technician
3129-15		Mine Deputy	312913	Mine Deputy	31191	p	Other Engineering Technician
3992-11	p	Safety Inspector			81111	p	Quarry and Mine Worker
3129-11	p	Biomedical Engineering Associate	312999	Building and Engineering Technicians nec	31181	p	Draughting Technician
3129-79	p	Building and Engineering Associate Professionals nec			31191	p	Other Engineering Technician
3294-11	p	Computing Support Technician	313111	Hardware Technician	31213	p	Computer Support Technician
4315-01	p	Supervisor, Electronic and Office Equipment Tradespersons					
4315-11	p	Electronic Equipment Tradesperson					
4315-81	p	Apprentice Electronic Equipment Tradesperson					
3294-11	p	Computing Support Technician	313112	ICT Customer Support Officer	21311	p	Systems Analyst
					21312	p	Computer Applications Engineer
					31142	p	Computer Systems Technician
					31213	p	Computer Support Technician
2231-11	p	Systems Manager	313113	Web Administrator	21312	p	Computer Applications Engineer
					21313	p	Systems Manager
3124-11	p	Electronic Engineering Associate	313199	ICT Support Technicians nec	31142	p	Computer Systems Technician
3124-13	p	Electronic Engineering Technician			31212		Computer Operator
3294-11	p	Computing Support Technician					
3124-13	p	Electronic Engineering Technician	313211	Radiocommunications Technician	31141	p	Telecommunications Technician
4316-01	p	Supervisor, Communications Tradespersons	313212	Telecommunications Field Engineer	31141	p	Telecommunications Technician
4316-11	p	General Communications Tradesperson					
4316-81	p	Apprentice General Communications Tradesperson					
3124-11	p	Electronic Engineering Associate	313213	Telecommunications Network Planner	31141	p	Telecommunications Technician
3124-13	p	Electronic Engineering Technician					
3124-11	p	Electronic Engineering Associate	313214	Telecommunications Technical Officer or Technologist	31141	p	Telecommunications Technician
3124-13	p	Electronic Engineering Technician					
4212-01		Supervisor, Automotive Electricians	321111	Automotive Electrician	71312		Transport Electrician
4212-11		Automotive Electrician					
4212-81		Apprentice Automotive Electrician					
4211-01	p	Supervisor, Motor Mechanics	321211	Motor Mechanic (General)	72311	p	Machinery Mechanic
4211-11	p	Motor Mechanic			72312	p	Motor Mechanic
4211-81	p	Apprentice Motor Mechanic					
4211-01	p	Supervisor, Motor Mechanics	321212	Diesel Motor Mechanic	72311	p	Machinery Mechanic
4211-11	p	Motor Mechanic					
4211-81	p	Apprentice Motor Mechanic					
4211-01	p	Supervisor, Motor Mechanics	321213	Motorcycle Mechanic	72315	p	Small Engine Mechanic
4211-11	p	Motor Mechanic					
4211-81	p	Apprentice Motor Mechanic					
4211-01	p	Supervisor, Motor Mechanics	321214	Small Engine Mechanic	72315	p	Small Engine Mechanic
4211-11	p	Motor Mechanic					
4211-81	p	Apprentice Motor Mechanic					
4999-79	p	Tradespersons and Related Workers nec					
4123-01	p	Supervisor, Forging Tradespersons	322111	Blacksmith	72211	p	Blacksmith
4123-11		Blacksmith			82113		Spring Maker and Wire Worker
4123-81		Apprentice Blacksmith					
4126-01	p	Supervisor, Metal Finishing Tradespersons	322112	Electroplater	82221		Electroplater
4126-13		Electroplater (First Class)					
4126-83		Apprentice Electroplater					
4123-01	p	Supervisor, Forging Tradespersons	322113	Farrier	72211	p	Blacksmith
4123-13		Farrier					
4123-83		Apprentice Farrier					
4125-01		Supervisor, Metal Casting Tradespersons	322114	Metal Casting Trades Worker	72111		Metal Mould Maker
4125-11		Metal Casting Tradesperson					
4125-81		Apprentice Metal Casting Tradesperson					
4126-01	p	Supervisor, Metal Finishing Tradespersons	322115	Metal Polisher	82223	p	Metal Polisher
4126-11		Metal Polisher					
4126-81		Apprentice Metal Polisher					

ASCO Second Edition			ANZSCO		NZSCO 1999		
4121-01	p	Supervisor, General Fabrication Engineering Tradespersons	322211	Sheetmetal Trades Worker	72122	p	Sheet-Metal Worker
4121-11	p	General Fabrication Engineering Tradesperson					
4121-81	p	Apprentice General Fabrication Engineering Tradesperson					
4124-01	p	Supervisor, Sheetmetal Tradespersons					
4124-11	p	Sheetmetal Worker (First Class)					
4124-81	p	Apprentice Sheetmetal Worker					
4121-01	p	Supervisor, General Fabrication Engineering Tradespersons	322311	Metal Fabricator	72123		Boiler Maker
4121-11	p	General Fabrication Engineering Tradesperson			72124	p	Fitter and Welder
4121-81	p	Apprentice General Fabrication Engineering Tradesperson			82223	p	Metal Polisher
4122-01	p	Supervisor, Structural Steel and Welding Tradespersons					
4122-11	p	Metal Fabricator					
4122-81	p	Apprentice Metal Fabricator					
4122-01	p	Supervisor, Structural Steel and Welding Tradespersons	322312	Pressure Welder	72124	p	Fitter and Welder
4122-13		Pressure Welder					
4122-83	p	Apprentice Welder					
4121-01	p	Supervisor, General Fabrication Engineering Tradespersons	322313	Welder (First Class) (Aus) / Welder (NZ)	72124	p	Fitter and Welder
4121-11	p	General Fabrication Engineering Tradesperson			81231	p	Welder and Flame-Cutter
4121-81	p	Apprentice General Fabrication Engineering Tradesperson					
4122-01	p	Supervisor, Structural Steel and Welding Tradespersons					
4122-15		Welder (First Class)					
4122-83	p	Apprentice Welder					
4114-01	p	Supervisor, Aircraft Maintenance Engineers	323111	Aircraft Maintenance Engineer (Avionics)	31144	p	Avionics Technician
4114-15		Aircraft Maintenance Engineer (Avionics)			72422		Avionics Mechanic
4114-85		Apprentice Aircraft Maintenance Engineer (Avionics)					
4114-01	p	Supervisor, Aircraft Maintenance Engineers	323112	Aircraft Maintenance Engineer (Mechanical)	21453	p	Aeronautical Engineer and/or Aircraft Surveyor
4114-11		Aircraft Maintenance Engineer (Mechanical)			72313	p	Aircraft Engine Mechanic
4114-81		Apprentice Aircraft Maintenance Engineer (Mechanical)					
4114-01	p	Supervisor, Aircraft Maintenance Engineers	323113	Aircraft Maintenance Engineer (Structures)	72313	p	Aircraft Engine Mechanic
4114-13		Aircraft Maintenance Engineer (Structures)					
4114-83		Apprentice Aircraft Maintenance Engineer (Structures)					
4112-01	p	Supervisor, Metal Fitters and Machinists	323211	Fitter (General)	72231	p	Fitter and Turner
4112-11	p	Fitter			72311	p	Machinery Mechanic
4112-81	p	Apprentice Fitter			82111	p	Machine Tool Operator
4112-01	p	Supervisor, Metal Fitters and Machinists	323212	Fitter and Turner	72231	p	Fitter and Turner
4112-11	p	Fitter					
4112-81	p	Apprentice Fitter					
4112-01	p	Supervisor, Metal Fitters and Machinists	323213	Fitter-Welder	72124	p	Fitter and Welder
4112-11	p	Fitter					
4112-81	p	Apprentice Fitter					
4112-01	p	Supervisor, Metal Fitters and Machinists	323214	Metal Machinist (First Class)	82111	p	Machine Tool Operator
4112-13	p	Metal Machinist (First Class)					
4112-83	p	Apprentice Metal Machinist					
4112-01	p	Supervisor, Metal Fitters and Machinists	323215	Textile, Clothing and Footwear Mechanic	72311	p	Machinery Mechanic
4112-15		Textile, Clothing or Footwear Mechanic					
4112-81	p	Apprentice Fitter					
4112-85		Apprentice Textile, Clothing or Footwear Mechanic					

ASCO Second Edition			ANZSCO		NZSCO 1999		
4111-01		Supervisor, General Mechanical Engineering Tradespersons	323299	Metal Fitters and Machinists nec	72311	p	Machinery Mechanic
4111-11		General Mechanical Engineering Tradesperson					
4111-81		Apprentice General Mechanical Engineering Tradesperson					
4112-01	p	Supervisor, Metal Fitters and Machinists					
4112-11	p	Fitter					
4112-13	p	Metal Machinist (First Class)					
4112-81	p	Apprentice Fitter					
4112-83	p	Apprentice Metal Machinist					
4999-79	p	Tradespersons and Related Workers nec					
4115-01	p	Supervisor, Precision Metal Tradespersons	323311	Engraver	33621	p	Sculptor, Painter and Related Artist
4115-21		Engraver			73131	p	Jeweller and Jewellery Repairer
4115-81	p	Apprentice Precision Metal Tradesperson			73211	p	Glass Cutter and Beveller
					73317	p	Printing Machinist
					73331	p	Photolithographer, Photo Engraver
4115-01	p	Supervisor, Precision Metal Tradespersons	323312	Gunsmith	51522	p	Police Officer
4115-19		Gunsmith			73111	p	Industrial Precision Instrument Maker and Repairer
4115-81	p	Apprentice Precision Metal Tradesperson					
4115-01	p	Supervisor, Precision Metal Tradespersons	323313	Locksmith	73112		Locksmith
4115-15		Locksmith					
4115-81	p	Apprentice Precision Metal Tradesperson					
4115-01	p	Supervisor, Precision Metal Tradespersons	323314	Precision Instrument Maker and Repairer	73111	p	Industrial Precision Instrument Maker and Repairer
4115-11		Precision Instrument Maker and Repairer			73113	p	Optical Instrument Maker, Repairer and Mechanic
4115-81	p	Apprentice Precision Metal Tradesperson			82114	p	Tool Grinder and Sharpener
4115-01	p	Supervisor, Precision Metal Tradespersons	323315	Saw Maker and Repairer	72241		Saw Doctor
4115-17		Saw Maker and Repairer					
4115-81	p	Apprentice Precision Metal Tradesperson					
4115-01	p	Supervisor, Precision Metal Tradespersons	323316	Watch and Clock Maker and Repairer	73115		Watchmaker and Repairer
4115-13		Watch and Clock Maker and Repairer					
4115-81	p	Apprentice Precision Metal Tradesperson					
4122-01	p	Supervisor, Structural Steel and Welding Tradespersons	323411	Engineering Patternmaker	72221		Pattern Maker
4122-11	p	Metal Fabricator					
4122-81	p	Apprentice Metal Fabricator					
4124-01	p	Supervisor, Sheetmetal Tradespersons					
4124-11	p	Sheetmetal Worker (First Class)					
4124-81	p	Apprentice Sheetmetal Worker					
4113-01		Supervisor, Toolmakers	323412	Toolmaker	72222		Tool and/or Die Maker
4113-11		Toolmaker					
4113-81		Apprentice Toolmaker					
4213-01		Supervisor, Panel Beaters	324111	Panelbeater	72125		Panel Beater
4213-11		Panel Beater					
4213-81		Apprentice Panel Beater					
4215-01		Supervisor, Vehicle Body Makers	324211	Vehicle Body Builder	72121		Coach Builder
4215-11		Vehicle Body Maker					
4215-81		Apprentice Vehicle Body Maker					
4216-01		Supervisor, Vehicle Trimmers	324212	Vehicle Trimmer	74322		Vehicle Upholsterer and Trimmer
4216-11		Vehicle Trimmer					
4216-81		Apprentice Vehicle Trimmer					
4214-01		Supervisor, Vehicle Painters	324311	Vehicle Painter	71242	p	Spray Painter
4214-11		Vehicle Painter					
4214-81		Apprentice Vehicle Painter					
4414-01		Supervisor, Bricklayers	331111	Bricklayer	71111	p	Bricklayer and/or Blocklayer
4414-11		Bricklayer					
4414-81		Apprentice Bricklayer					
4416-01	p	Supervisor, Wall and Floor Tilers and Stonemasons	331112	Stonemason	71112	p	Stonemason
4416-13		Stonemason					
4416-83		Apprentice Stonemason					

ASCO Second Edition			ANZSCO		NZSCO 1999		
4411-01	p	Supervisor, Carpentry and Joinery Tradespersons	331211	Carpenter and Joiner	71121	p	Carpenter and/or Joiner
4411-11		Carpenter and Joiner					
4411-81		Apprentice Carpenter and Joiner					
4411-01	p	Supervisor, Carpentry and Joinery Tradespersons	331212	Carpenter	71121	p	Carpenter and/or Joiner
4411-13		Carpenter					
4411-83		Apprentice Carpenter					
4411-01	p	Supervisor, Carpentry and Joinery Tradespersons	331213	Joiner	71121	p	Carpenter and/or Joiner
4411-15		Joiner					
4411-85		Apprentice Joiner					
4423-01		Supervisor, Floor Finishers	332111	Floor Finisher	74331		Carpet and Other Floor Covering Layer
4423-11		Floor Finisher					
4423-81		Apprentice Floor Finisher					
4421-01		Supervisor, Painters and Decorators	332211	Painting Trades Worker	71241		Painter, Decorator and/or Paperhanger
4421-11		Painter and Decorator					
4421-81		Apprentice Painter and Decorator					
4982-11		Flat Glass Tradesperson	333111	Glazier	71221	p	Glazier
4982-81		Apprentice Flat Glass Tradesperson			73211	p	Glass Cutter and Beveller
4412-01		Supervisor, Fibrous Plasterers	333211	Fibrous Plasterer	71211	p	Plasterer
4412-11		Fibrous Plasterer					
4412-81		Apprentice Fibrous Plasterer					
4415-01		Supervisor, Solid Plasterers	333212	Solid Plasterer	71211	p	Plasterer
4415-11		Solid Plasterer					
4415-81		Apprentice Solid Plasterer					
4413-01		Supervisor, Roof Slaters and Tilers	333311	Roof Tiler	84117		Roofer
4413-11		Roof Slater and Tiler					
4413-81		Apprentice Roof Slater and Tiler					
4416-01	p	Supervisor, Wall and Floor Tilers and Stonemasons	333411	Wall and Floor Tiler	71111	p	Bricklayer and/or Blocklayer
4416-11		Wall and Floor Tiler					
4416-81		Apprentice Wall and Floor Tiler					
4431-01	p	Supervisor, Plumbers	334111	Plumber (General)	71231	p	Plumber
4431-11		General Plumber			72412	p	Fire Alarm Technician
4431-81		Apprentice General Plumber			84112	p	Pipe Fitter
4431-01	p	Supervisor, Plumbers	334112	Airconditioning and Mechanical Services Plumber	71231	p	Plumber
4431-19		Mechanical Services and Airconditioning Plumber					
4431-89		Apprentice Mechanical Services and Airconditioning Plumber					
4431-01	p	Supervisor, Plumbers	334113	Drainer (Aus) / Drainlayer (NZ)	84111	p	Drainlayer
4431-15		Drainer					
4431-85		Apprentice Drainer					
4431-01	p	Supervisor, Plumbers	334114	Gasfitter	84112	p	Pipe Fitter
4431-13		Gasfitter					
4431-83		Apprentice Gasfitter					
4431-01	p	Supervisor, Plumbers	334115	Roof Plumber	71231	p	Plumber
4431-17		Roof Plumber					
4431-87		Apprentice Roof Plumber					
4311-01	p	Supervisor, Electricians	341111	Electrician (General)	71311	p	Electrician
4311-11		General Electrician			71313		Appliance Electrician
4311-81		Apprentice Electrician			72411	p	Electrical Fitter
					82921		Coil Winder
4311-01	p	Supervisor, Electricians	341112	Electrician (Special Class)	71311	p	Electrician
4311-13		Electrician (Special Class)					
4311-01	p	Supervisor, Electricians	341113	Lift Mechanic	72411	p	Electrical Fitter
4311-15		Lift Mechanic					
4311-83		Apprentice Lift Mechanic					
4312-01		Supervisor, Refrigeration and Airconditioning Mechanics	342111	Airconditioning and Refrigeration Mechanic	72314		Heating, Ventilation and Refrigeration Mechanic
4312-11		Refrigeration and Airconditioning Mechanic					
4312-81		Apprentice Refrigeration and Airconditioning Mechanic					
4313-01	p	Supervisor, Electrical Distribution Tradespersons	342211	Electrical Linesworker (Aus) / Electrical Line Mechanic (NZ)	82923	p	Linesperson
4313-11		Electrical Powerline Tradesperson					
4313-81		Apprentice Electrical Powerline Tradesperson					

ASCO Second Edition			ANZSCO		NZSCO 1999		
4313-01	p	Supervisor, Electrical Distribution Tradespersons	342212	Technical Cable Jointer	82924	p	Electric Cable Jointer
4313-13		Cable Jointer					
4313-83		Apprentice Cable Jointer					
4315-01	p	Supervisor, Electronic and Office Equipment Tradespersons	342311	Business Machine Mechanic	72421	p	Electronics Servicer
4315-13		Business Machine Mechanic					
4315-83		Apprentice Business Machine Mechanic					
3999-15		Radio Operator	342312	Communications Operator	31322	p	Radio Operator
3999-79	p	Associate Professionals nec					
4315-01	p	Supervisor, Electronic and Office Equipment Tradespersons	342313	Electronic Equipment Trades Worker	72311	p	Machinery Mechanic
4315-11	p	Electronic Equipment Tradesperson			72421	p	Electronics Servicer
4315-81	p	Apprentice Electronic Equipment Tradesperson			72431		Radio and Television Repairer
4314-01	p	Supervisor, Electronic Instrument Tradespersons	342314	Electronic Instrument Trades Worker (General)	72421	p	Electronics Servicer
4314-11		General Electronic Instrument Tradesperson					
4314-81		Apprentice Electronic Instrument Tradesperson					
4314-01	p	Supervisor, Electronic Instrument Tradespersons	342315	Electronic Instrument Trades Worker (Special Class)	31131	p	Electrical Engineering Technician
4314-13		Electronic Instrument Tradesperson (Special Class)			72421	p	Electronics Servicer
4316-01	p	Supervisor, Communications Tradespersons	342411	Cabler (Data and Telecommunications)	31143	p	Other Electronics Engineering Technician
4316-13	p	Communications Linesperson					
4316-83	p	Apprentice Communications Linesperson					
7914-13	p	Home Improvements Installer					
4316-01	p	Supervisor, Communications Tradespersons	342412	Telecommunications Cable Jointer	82924	p	Electric Cable Jointer
4316-13	p	Communications Linesperson					
4316-83	p	Apprentice Communications Linesperson					
4316-01	p	Supervisor, Communications Tradespersons	342413	Telecommunications Linesworker (Aus) / Telecommunications Line Mechanic (NZ)	82923	p	Linesperson
4316-13	p	Communications Linesperson					
4316-83	p	Apprentice Communications Linesperson					
4316-01	p	Supervisor, Communications Tradespersons	342414	Telecommunications Technician	31141	p	Telecommunications Technician
4316-11	p	General Communications Tradesperson					
4316-81	p	Apprentice General Communications Tradesperson					
4512-01	p	Supervisor, Bakers and Pastrycooks	351111	Baker	74121	p	Baker
4512-11		Baker					
4512-81		Apprentice Baker					
4512-01	p	Supervisor, Bakers and Pastrycooks	351112	Pastrycook	74121	p	Baker
4512-13		Pastrycook					
4512-83		Apprentice Pastrycook					
4511-01	p	Supervisor, Meat Tradespersons	351211	Butcher or Smallgoods Maker	74111	p	Butcher
4511-11		Butcher			82713	p	Smallgoods Maker
4511-13		Smallgoods Maker					
4511-81		Apprentice Butcher					
4511-83		Apprentice Smallgoods Maker					
3322-01		Head Chef	351311	Chef	51221		Chef
3322-11		Chef					
4513-11		Cook	351411	Cook	51222	p	Cook
4513-81		Apprentice Cook or Chef					
4614-79	p	Animal Trainers nec	361111	Dog Handler or Trainer	61441	p	Animal Welfare Worker
4611-11	p	Farm Overseer	361112	Horse Trainer	61265	p	Horse Trainer, Groom or Stable Hand
4614-11		Horse Trainer					
6399-15	p	Animal Attendant	361113	Pet Groomer	61441	p	Animal Welfare Worker
6399-15	p	Animal Attendant	361114	Zookeeper	61441	p	Animal Welfare Worker
4614-79	p	Animal Trainers nec	361199	Animal Attendants and Trainers nec	32241	p	Veterinary Assistant
4999-79	p	Tradespersons and Related Workers nec			33312	p	Quarantine and Agriculture Ports Officer
6399-15	p	Animal Attendant			61212	p	Sheep Farmer, Sheep Farm Worker
					61441	p	Animal Welfare Worker
4612-11		Shearer	361211	Shearer	61262		Shearing Contractor/Shearer
6392-11		Veterinary Nurse	361311	Veterinary Nurse	32241	p	Veterinary Assistant
4984-11		Florist	362111	Florist	52111	p	Sales Assistant
4623-01	p	Head Gardener	362211	Gardener (General)	61133	p	Grounds or Green Keeper
4623-11		General Gardener			61134	p	Gardener
4623-81		Apprentice General Gardener					

ASCO Second Edition			ANZSCO		NZSCO 1999		
4623-01	p	Head Gardener	362212	Arborist	61134	p	Gardener
4623-15		Tree Surgeon					
4623-85		Apprentice Tree Surgeon					
4623-01	p	Head Gardener	362213	Landscape Gardener	61132		Landscape Gardener
4623-13		Landscape Gardener					
4623-83		Apprentice Landscape Gardener					
4622-11		Greenkeeper	362311	Greenkeeper	61133	p	Grounds or Green Keeper
4622-81		Apprentice Greenkeeper					
4621-11		Nurseryperson	362411	Nurseryperson	61131	p	Nursery Grower, Nursery Worker
4621-81		Apprentice Nurseryperson					
4931-01		Supervisor, Hairdressers	391111	Hairdresser	51411	p	Hairdresser
4931-11		Hairdresser					
4931-81		Apprentice Hairdresser					
4913-11		Binder and Finisher	392111	Binder and Finisher	73321	p	Bookbinder
4913-81		Apprentice Binder and Finisher					
4914-11		Screen Printer	392112	Screen Printer	73316		Screen Printer
4914-81		Apprentice Screen Printer					
4911-11		Graphic Pre-Press Tradesperson	392211	Graphic Pre-press Trades Worker	33635		Paste Up Artist
4911-81		Apprentice Graphic Pre-Press Tradesperson			73311		Graphic Pre-press Tradesperson
5995-11		Desktop Publishing Operator			73318		Desktop Publisher
					73331	p	Photolithographer, Photo Engraver
4912-11		Printing Machinist	392311	Printing Machinist	73317	p	Printing Machinist
4912-81		Apprentice Printing Machinist					
4912-13		Small Offset Printer	392312	Small Offset Printer	73317	p	Printing Machinist
4912-83		Apprentice Small Offset Printer					
4944-13		Canvas Goods Maker	393111	Canvas Goods Maker	74323	p	Canvas Worker
4944-81	p	Apprentice Leather Goods, Canvas Goods or Sail Maker					
4944-11		Leather Goods Maker	393112	Leather Goods Maker	74411		Saddler and Harness Maker
4944-81	p	Apprentice Leather Goods, Canvas Goods or Sail Maker			82953	p	Leather Goods Assembler
4944-15		Sail Maker	393113	Sail Maker	74323	p	Canvas Worker
4944-81	p	Apprentice Leather Goods, Canvas Goods or Sail Maker					
4943-11		Shoemaker	393114	Shoemaker	32234	p	Orthotist and/or Prosthetist
4943-13		Medical Grade Shoemaker			74412		Shoe Repairer
4943-81		Apprentice Shoemaker			82954	p	Footwear Production Machine Operator
4941-17		Apparel Cutter	393211	Apparel Cutter	74313	p	Textile Products Marker and Cutter
4941-81	p	Apprentice Clothing Tradesperson					
4941-19		Patternmaker-Grader (Clothing)	393212	Clothing Patternmaker	74312		Textile Products Pattern Maker
4941-81	p	Apprentice Clothing Tradesperson			74313	p	Textile Products Marker and Cutter
4941-11		General Clothing Tradesperson	393213	Dressmaker or Tailor	74311	p	Tailor/Dressmaker
4941-13		Tailor					
4941-15		Dressmaker					
4941-81	p	Apprentice Clothing Tradesperson					
4941-79		Clothing Tradespersons nec	393299	Clothing Trades Workers nec	74311	p	Tailor/Dressmaker
4941-81	p	Apprentice Clothing Tradesperson			74313	p	Textile Products Marker and Cutter
					82635		Hat Maker
4942-11		Furniture Upholsterer	393311	Upholsterer	74321	p	Furniture Upholsterer
4942-79		Upholsterers and Bedding Tradespersons nec					
4942-81		Apprentice Upholsterer or Bedding Tradespersons					
4922-01		Supervisor, Cabinetmakers	394111	Cabinetmaker	74211	p	Cabinetmaker
4922-11		Cabinetmaker			74212	p	Furniture Finisher
4922-81		Apprentice Cabinetmaker					
4929-13		Furniture Finisher	394211	Furniture Finisher	74212	p	Furniture Finisher
4929-83		Apprentice Furniture Finisher					
4929-11		Picture Framer	394212	Picture Framer	74211	p	Cabinetmaker
4929-81		Apprentice Picture Framer					
4921-11		Wood Machinist (A-Grade)	394213	Wood Machinist	82411	p	Woodworking Machinist
4921-81		Apprentice Wood Machinist					
4921-13		Wood Turner	394214	Wood Turner	82411	p	Woodworking Machinist
4921-83		Apprentice Wood Turner					
4929-79		Wood Tradespersons nec	394299	Wood Machinists and Other Wood Trades Workers nec	74211	p	Cabinetmaker
4929-99		Apprentice Wood Tradespersons nec			82411	p	Woodworking Machinist
					82942	p	Basket and Wicker Worker
4981-13		Boat Builder and Repairer	399111	Boat Builder and Repairer	71123	p	Boatbuilder
4981-83		Apprentice Boat Builder and Repairer			72124	p	Fitter and Welder
4999-79	p	Tradespersons and Related Workers nec			82932	p	Plastic and Rubber Goods Assembler
					84115	p	Rigger and Cable Splicer

ASCO Second Edition			ANZSCO		NZSCO 1999		
4981-11		Shipwright	399112	Shipwright	71121	p	Carpenter and/or Joiner
4981-81		Apprentice Shipwright			71123	p	Boatbuilder
					72124	p	Fitter and Welder
4987-01	p	Supervisor, Chemical, Petroleum and Gas Plant Operators	399211	Chemical Plant Operator	81511	p	Chemical Crushing, Grinding and Mixing Operator
4987-11		Chemical Plant Operator			81531	p	Still and Reactor Operator
					81541	p	Other Chemical Processing Plant Operator
					82211	p	Pharmaceutical and Toiletry Products Machine Operator
4987-01	p	Supervisor, Chemical, Petroleum and Gas Plant Operators	399212	Gas or Petroleum Operator	81531	p	Still and Reactor Operator
4987-13	p	Petroleum and Gas Plant Operator					
4988-01		Supervisor, Power Generation Plant Operators	399213	Power Generation Plant Operator	81611		Power Generating Plant Operator
4988-11		Power Generation Plant Operator					
3999-13		Museum or Art Gallery Technician	399311	Gallery or Museum Technician	31111	p	Physical Science Technician
					32111	p	Life Science Technician
3997-11		Library Technician	399312	Library Technician	41411	p	Library Assistant
2531-79	p	Visual Arts and Crafts Professionals nec	399411	Jeweller	73131	p	Jeweller and Jewellery Repairer
4983-11		Jeweller			73132		Gem Cutter and Polisher
4983-13		Gem Cutter and Polisher					
4983-81		Apprentice Jeweller					
4983-83		Apprentice Gem Cutter and Polisher					
4992-17		Broadcast Transmitter Operator	399511	Broadcast Transmitter Operator	31321	p	Broadcasting Transmitting and Studio Equipment Operator
4992-13		Camera Operator (Film, Television or Video)	399512	Camera Operator (Film, Television or Video)	31312		Camera Operator
4992-21		Light Technician	399513	Light Technician	31321	p	Broadcasting Transmitting and Studio Equipment Operator
					33634	p	Industrial Designer
4992-27		Make Up Artist	399514	Make Up Artist	51412	p	Beauty Therapist
2531-79	p	Visual Arts and Crafts Professionals nec	399515	Musical Instrument Maker or Repairer	73121		Musical Instrument Maker, Repairer and Tuner
4999-17		Piano Tuner					
4999-79	p	Tradespersons and Related Workers nec					
4992-11		Sound Technician	399516	Sound Technician	31313		Sound Recording Equipment Controller
4992-15		Television Equipment Operator	399517	Television Equipment Operator	31321	p	Broadcasting Transmitting and Studio Equipment Operator
					33662	p	Artistic Director
4992-79		Performing Arts Support Workers nec	399599	Performing Arts Technicians nec	31321	p	Broadcasting Transmitting and Studio Equipment Operator
4422-01		Supervisor, Signwriters	399611	Signwriter	71243		Signwriter
4422-11		Signwriter					
4422-81		Apprentice Signwriter					
4999-13	p	Diver	399911	Diver	61411	p	Fishing Skipper, Fisherperson
					61413	p	Shell Fisher
					84121		Underwater Worker
3999-11		Interior Decorator	399912	Interior Decorator	33636	p	Interior Designer
4999-11	p	Optical Mechanic	399913	Optical Dispenser (Aus) / Dispensing Optician (NZ)	32211		Dispensing Optician
4999-11	p	Optical Mechanic	399914	Optical Mechanic	73113	p	Optical Instrument Maker, Repairer and Mechanic
5999-17		Photographer's Assistant	399915	Photographer's Assistant	31311	p	Photographer
7291-01	p	Supervisor, Plastics Production Machine Operators	399916	Plastics Technician	82322	p	Plastics Machine Operator
7291-11	p	General Plastics Production Machine Operator					
7291-13	p	Plastics Compounding and Reclamation Machine Operator					
7291-15	p	Reinforced Plastic and Composite Production Worker					
7291-17	p	Plastic Cablemaking Machine Operator					
7291-79	p	Plastics Production Machine Operators nec					
4613-11		Wool Classer	399917	Wool Classer	61263		Wool Classer
3999-79	p	Associate Professionals nec	399999	Technicians and Trades Workers nec	31191	p	Other Engineering Technician
4613-13		Hide and Skin Classer			72315	p	Small Engine Mechanic
4982-13		Glass Blower			72411	p	Electrical Fitter
4982-83		Apprentice Glass Blower			72412	p	Fire Alarm Technician
4999-79	p	Tradespersons and Related Workers nec			74211	p	Cabinetmaker
8319-79	p	Elementary Service Workers nec			81321	p	Glass Pressing and Drawing Machine Operator

ASCO Second Edition			ANZSCO		NZSCO 1999		
			**4**	**COMMUNITY AND PERSONAL SERVICE WORKERS**			
3491-11		Ambulance Officer	411111	Ambulance Officer	51313	p	Ambulance Officer
3491-13		Intensive Care Ambulance Paramedic	411112	Intensive Care Ambulance Paramedic (Aus) / Ambulance Paramedic (NZ)	51313	p	Ambulance Officer
3492-13		Dental Hygienist	411211	Dental Hygienist	32221	p	Dental Therapist
3492-15	p	Dental Technician	411212	Dental Prosthetist	32262	p	Dental Technician
3492-15	p	Dental Technician	411213	Dental Technician	32262	p	Dental Technician
3492-11		Dental Therapist	411214	Dental Therapist	32221	p	Dental Therapist
2399-79	p	Health Professionals nec	411311	Diversional Therapist	32232	p	Occupational Therapist
2549-19	p	Recreation Officer			32261	p	Other Health Associate Professional
3411-11	p	Enrolled Nurse	411411	Enrolled Nurse	32311		Enrolled Nurse
3411-11	p	Enrolled Nurse	411412	Mothercraft Nurse	32312	p	Karitane Nurse
3493-11		Aboriginal and Torres Strait Islander Health Worker	411511	Aboriginal and Torres Strait Islander Health Worker	51312	p	Health Assistant
3411-11	p	Enrolled Nurse	411512	Kaiāwhina (Hauora) (Māori Health Assistant)	51312	p	Health Assistant
3494-11		Massage Therapist	411611	Massage Therapist	51413		Massage Therapist
2512-13	p	Community Worker	411711	Community Worker	33411	p	Social Worker
3421-17		Disabilities Services Officer	411712	Disabilities Services Officer	33411	p	Social Worker
					33413	p	Case Worker
3421-19		Family Support Worker	411713	Family Support Worker	33411	p	Social Worker
3421-11		Parole or Probation Officer	411714	Parole or Probation Officer	33412		Probation Worker
3421-15		Residential Care Officer	411715	Residential Care Officer	33411	p	Social Worker
3421-13		Youth Worker	411716	Youth Worker	33411	p	Social Worker
6312-11	p	Child Care Worker	421111	Child Care Worker	23321	p	Early Childhood Teacher
					51316	p	Caregiver
					51421	p	Child Care Worker
6312-13		Family Day Care Worker	421112	Family Day Care Worker	51316	p	Caregiver
6312-15		Nanny	421113	Nanny	51421	p	Child Care Worker
6312-11	p	Child Care Worker	421114	Out of School Hours Care Worker	51316	p	Caregiver
6311-17		Aboriginal and Torres Strait Islander Education Worker	422111	Aboriginal and Torres Strait Islander Education Worker	33422	p	Teacher Aide
6311-13		Integration Aide	422112	Integration Aide	33422	p	Teacher Aide
6311-11	p	Pre-School Aide	422113	Kaiāwhina Kōhanga Reo (Māori Language Nest Assistant)	33422	p	Teacher Aide
					51421	p	Child Care Worker
6311-15	p	Teachers' Aide	422114	Kaiāwhina Kura Kaupapa Māori (Māori-medium School Assistant)	33422	p	Teacher Aide
					51421	p	Child Care Worker
6311-11	p	Pre-School Aide	422115	Preschool Aide	23321	p	Early Childhood Teacher
					51421	p	Child Care Worker
6311-15	p	Teachers' Aide	422116	Teachers' Aide	33421		Employment Programme Teaching Associate Professional
					33422	p	Teacher Aide
6313-17		Aged or Disabled Person Carer	423111	Aged or Disabled Carer	51312	p	Health Assistant
					51316	p	Caregiver
6391-11		Dental Assistant	423211	Dental Assistant	51314	p	Nurse Aide
6314-11	p	Personal Care Assistant	423311	Hospital Orderly	51311		Hospital Orderly
6314-13	p	Nursing Assistant			91111	p	Cleaner
3999-79	p	Associate Professionals nec	423312	Nursing Support Worker	51312	p	Health Assistant
6314-13	p	Nursing Assistant			51314	p	Nurse Aide
6314-11	p	Personal Care Assistant	423313	Personal Care Assistant	51312	p	Health Assistant
					51314	p	Nurse Aide
					51316	p	Caregiver
6313-19		Therapy Aide	423314	Therapy Aide	32231	p	Physiotherapist
					32232	p	Occupational Therapist
					32261	p	Other Health Associate Professional
					51314	p	Nurse Aide
6313-13		Child or Youth Residential Care Assistant	423411	Child or Youth Residential Care Assistant	51421	p	Child Care Worker
6313-11		Hostel Parent	423412	Hostel Parent	51421	p	Child Care Worker
6313-15		Refuge Worker	423413	Refuge Worker	51316	p	Caregiver
6322-01		Supervisor, Bar Attendants	431111	Bar Attendant	12267	p	Other Catering Services Manager
6322-11		Bar Attendant			51231	p	Bartender
6323-01	p	Supervisor, Waiters	431112	Barista	51234	p	Catering Counter Assistant
6213-11	p	Retail Supervisor	431211	Cafe Worker	51234	p	Catering Counter Assistant
8211-11	p	Sales Assistant (Food and Drink Products)					
6394-11		Gaming Pit Boss	431311	Gaming Worker	42131		Gaming Dealer
6394-13		Gaming Table Supervisor					
6394-15		Gaming Dealer					

ASCO Second Edition			ANZSCO		NZSCO 1999		
3291-11	p	Office Manager	431411	Hotel Service Manager	12267	p	Other Catering Services Manager
6321-11		Hotel Service Supervisor			51212	p	Housekeeper (Not Private)
					91212	p	Hotel Porter
6323-01	p	Supervisor, Waiters	431511	Waiter	51232		Wine Waiter
6323-11		General Waiter			51233		Waiter
6323-13		Drink Waiter					
6399-79	p	Intermediate Service Workers nec					
8319-79	p	Elementary Service Workers nec	431911	Bar Useful or Busser	51231	p	Bartender
8312-13	p	Luggage Porter or Doorperson	431912	Doorperson or Luggage Porter	51236	p	Usher and Cloakroom Attendant
					91212	p	Hotel Porter
					91412	p	Loader and/or Checker
6324-11		Hospitality Trainee	431999	Hospitality Workers nec	51231	p	Bartender
6399-79	p	Intermediate Service Workers nec			51236	p	Usher and Cloakroom Attendant
8312-13	p	Luggage Porter or Doorperson					
8319-79	p	Elementary Service Workers nec					
4991-11		Defence Force Member Not Elsewhere Included	441111	Defence Force Member - Other Ranks	51551	p	Armed Forces
4991-81		Trainee Defence Force Member Not Elsewhere Included					
3999-79	p	Associate Professionals nec	441211	Emergency Service Worker	51511	p	Fire Fighter
3995-11		Senior Fire Fighter	441212	Fire Fighter	51511	p	Fire Fighter
4985-11		Fire Fighter					
3911-01	p	Supervisor, Police Officers	441311	Detective	51521	p	Detective
3911-11	p	Police Officer					
3911-01	p	Supervisor, Police Officers	441312	Police Officer	51522	p	Police Officer
3911-11	p	Police Officer			51551	p	Armed Forces
6393-11		Prison Officer	442111	Prison Officer	41443	p	General Clerk
					51531		Prison Officer
8311-79	p	Guards and Security Officers nec	442211	Alarm, Security or Surveillance Monitor	42221	p	Telephone Switchboard Operator
					51542	p	Security Officer
7314-11	p	Delivery Driver	442212	Armoured Car Escort	51542	p	Security Officer
8311-13		Armoured Car Escort					
8311-11	p	Security Officer	442213	Crowd Controller	51542	p	Security Officer
8312-13	p	Luggage Porter or Doorperson					
3999-17		Private Investigator	442214	Private Investigator	51541		Private Investigator
8311-11	p	Security Officer	442215	Retail Loss Prevention Officer	51542	p	Security Officer
3999-19		Security Adviser	442216	Security Consultant	51542	p	Security Officer
8311-11	p	Security Officer	442217	Security Officer	51542	p	Security Officer
8311-79	p	Guards and Security Officers nec	442299	Security Officers and Guards nec	51542	p	Security Officer
6395-11		Beauty Therapist	451111	Beauty Therapist	51412	p	Beauty Therapist
6399-13		Driving Instructor	451211	Driving Instructor	83213		Driving Instructor
3399-29	p	Funeral Director	451311	Funeral Director	51431	p	Funeral Director
3399-29	p	Funeral Director	451399	Funeral Workers nec	51431	p	Funeral Director
6399-11		Museum or Gallery Attendant	451411	Gallery or Museum Guide	51121	p	Tour and Travel Guide
6397-15		Tour Guide	451412	Tour Guide	51121	p	Tour and Travel Guide
6395-13		Natural Remedy Consultant	451511	Natural Remedy Consultant	32261	p	Other Health Associate Professional
6395-15		Weight Loss Consultant	451512	Weight Loss Consultant	51414		Weight Loss Consultant
6397-13		Tourist Information Officer	451611	Tourist Information Officer	42213	p	Information Clerk and Other Receptionist
6397-11		Travel Agent	451612	Travel Consultant	12241	p	Sales and/or Marketing Manager
					33141	p	Travel Consultant
5996-11		Flight Service Director	451711	Flight Attendant	51111	p	Travel Attendant
5996-13		Flight Attendant					
5996-79		Travel Attendants nec	451799	Travel Attendants nec	51111	p	Travel Attendant
6399-79	p	Intermediate Service Workers nec	451811	Civil Celebrant	99000	p	Response Outside Scope
8319-13		Hair and Beauty Salon Assistant	451812	Hair or Beauty Salon Assistant	51411	p	Hairdresser
8319-27		Prostitute or Escort	451813	Sex Worker or Escort	99000	p	Response Outside Scope
6399-79	p	Intermediate Service Workers nec	451899	Personal Service Workers nec	33621	p	Sculptor, Painter and Related Artist
8319-79	p	Elementary Service Workers nec			33681	p	Clown, Magician, Acrobat and Related Worker
					51111	p	Travel Attendant
					51211	p	Housekeeper (Private Service)
					51236	p	Usher and Cloakroom Attendant
					51314	p	Nurse Aide
					51542	p	Security Officer
					61441	p	Animal Welfare Worker
					99000	p	Response Outside Scope
6396-11	p	Fitness Instructor	452111	Fitness Instructor	33692	p	Sports Coach or Trainer
6396-13	p	Outdoor Adventure Leader	452211	Bungy Jump Master	51122	p	Outdoor Recreation Guide
6396-13	p	Outdoor Adventure Leader	452212	Fishing Guide	51122	p	Outdoor Recreation Guide
6396-13	p	Outdoor Adventure Leader	452213	Hunting Guide	51122	p	Outdoor Recreation Guide
6396-13	p	Outdoor Adventure Leader	452214	Mountain or Glacier Guide	51122	p	Outdoor Recreation Guide
6396-13	p	Outdoor Adventure Leader	452215	Outdoor Adventure Instructor	33692	p	Sports Coach or Trainer
					51122	p	Outdoor Recreation Guide
6396-13	p	Outdoor Adventure Leader	452216	Trekking Guide	51122	p	Outdoor Recreation Guide

ASCO Second Edition			ANZSCO		NZSCO 1999		
6396-13	p	Outdoor Adventure Leader	452217	Whitewater Rafting Guide	51122	p	Outdoor Recreation Guide
3993-27	p	Other Sports Coach	452299	Outdoor Adventure Guides nec	51122	p	Outdoor Recreation Guide
6396-13	p	Outdoor Adventure Leader					
4999-13	p	Diver	452311	Diving Instructor (Open Water)	33692	p	Sports Coach or Trainer
3993-19		Gymnastics Coach	452312	Gymnastics Coach or Instructor	33692	p	Sports Coach or Trainer
3993-25		Horseriding Coach	452313	Horse Riding Coach or Instructor	33692	p	Sports Coach or Trainer
3993-27	p	Other Sports Coach	452314	Snowsport Instructor	33692	p	Sports Coach or Trainer
3993-23		Swimming Coach	452315	Swimming Coach or Instructor	33692	p	Sports Coach or Trainer
6396-11	p	Fitness Instructor					
3993-21		Tennis Coach	452316	Tennis Coach	33692	p	Sports Coach or Trainer
3993-27	p	Other Sports Coach	452317	Other Sports Coach or Instructor	33692	p	Sports Coach or Trainer
3993-33		Horse or Dog Racing Official	452318	Dog or Horse Racing Official	33693	p	Sports Official
3993-29		Sports Development Officer	452321	Sports Development Officer	33693	p	Sports Official
3993-31		Sports Umpire	452322	Sports Umpire	33693	p	Sports Official
3993-35		Other Sports Official	452323	Other Sports Official	33693	p	Sports Official
3993-15		Footballer	452411	Footballer	33691	p	Professional Sportsperson
3993-13		Golfer	452412	Golfer	33691	p	Professional Sportsperson
3993-11		Jockey	452413	Jockey	33691	p	Professional Sportsperson
3993-17	p	Other Sportsperson	452414	Lifeguard	33691	p	Professional Sportsperson
3993-17	p	Other Sportsperson	452499	Sportspersons nec	33691	p	Professional Sportsperson
			**5**	**CLERICAL AND ADMINISTRATIVE WORKERS**			
3292-11	p	Project or Program Administrator	511111	Contract Administrator	33241	p	Organisation and Methods Analyst
3292-11	p	Project or Program Administrator	511112	Program or Project Administrator	12222	p	Administration Manager
					12291	p	Office Manager
					33211	p	Administration Officer
					33212	p	Conference/Function Organiser
					33242		Building Control/Consents Officer
					41443	p	General Clerk
3291-11	p	Office Manager	512111	Office Manager	12291	p	Office Manager
					41443	p	General Clerk
3291-11	p	Office Manager	512211	Health Practice Manager	12291	p	Office Manager
3291-11	p	Office Manager	512299	Practice Managers nec	12222	p	Administration Manager
					12291	p	Office Manager
5111-13		Personal Assistant	521111	Personal Assistant	41141	p	Secretary
5111-11	p	Secretary	521211	Secretary (General)	24461	p	Diplomatic Official
					41141	p	Secretary
5111-11	p	Secretary	521212	Legal Secretary	41141	p	Secretary
6111-11		General Clerk	531111	General Clerk	41443	p	General Clerk
6121-13		Data Entry Operator	532111	Data Entry Operator	41121	p	Data Entry Operator
5992-11		Court or Hansard Reporter	532112	Machine Shorthand Reporter	24431	p	Philologist, Translator or Interpreter
6121-11	p	Typist and Word Processing Operator			41111	p	Typist and Word Processor Operator
6121-11	p	Typist and Word Processing Operator	532113	Word Processing Operator	41111	p	Typist and Word Processor Operator
2291-17	p	Training Officer	541111	Call or Contact Centre Team Leader	41443	p	General Clerk
6191-11	p	Inquiry Clerk			42221	p	Telephone Switchboard Operator
8113-11	p	Switchboard Operator					
6191-11	p	Inquiry Clerk	541112	Call or Contact Centre Operator	42221	p	Telephone Switchboard Operator
8113-11	p	Switchboard Operator					
6191-11	p	Inquiry Clerk	541211	Inquiry Clerk	24461	p	Diplomatic Official
					33152	p	Technical Representative
					41443	p	General Clerk
					42213	p	Information Clerk and Other Receptionist
6131-11	p	Receptionist	542111	Receptionist (General)	42213	p	Information Clerk and Other Receptionist
6191-13		Admissions Clerk	542112	Admissions Clerk	41412	p	Record and Filing Clerk
					42212	p	Patient Receptionist
6131-11	p	Receptionist	542113	Hotel or Motel Receptionist	42111	p	Cashier
					42211		Hotel and/or Motel Receptionist
6131-11	p	Receptionist	542114	Medical Receptionist	42212	p	Patient Receptionist
6141-01	p	Supervisor, Accounting Clerks	551111	Accounts Clerk	24121	p	Human Resources Officer
6141-11		Accounts Clerk			41211	p	Accounts Clerk
					41212		Audit Clerk
					41221	p	Finance Clerk
6141-01	p	Supervisor, Accounting Clerks	551112	Cost Clerk	31143	p	Other Electronics Engineering Technician
6141-15		Cost Clerk			31151	p	Mechanical Engineering Technician
					41213	p	Costing Clerk
5911-11		Bookkeeper	551211	Bookkeeper	33231		Bookkeeper
6142-11		Payroll Clerk	551311	Payroll Clerk	12231	p	Human Resources Manager
					24121	p	Human Resources Officer
					41445	p	Human Resources Clerk
6143-01		Supervisor, Bank Workers	552111	Bank Worker	42121		Bank Officer
6143-11		Bank Worker					

APPENDIX A SUMMARY CORRESPONDENCE BETWEEN ANZSCO AND ASCO SECOND EDITION AND NZSCO 1999 *continued*

ASCO Second Edition			ANZSCO		NZSCO 1999		
5912-11		Credit and Loans Officer	552211	Credit or Loans Officer	12224	p	Finance Manager
6141-01	p	Supervisor, Accounting Clerks			33153	p	Sales Representative
6141-13		Credit Clerk			41213	p	Costing Clerk
					41221	p	Finance Clerk
3212-21		Bookmaker	552311	Bookmaker	12263	p	Retail Manager
3212-19	p	Insurance Broker	552312	Insurance Consultant	33121	p	Insurance Representative
6144-01		Supervisor, Insurance Clerks			41211	p	Accounts Clerk
6144-11		Insurance Clerk					
6145-01	p	Supervisor, Money Market and Statistical Clerks	552313	Money Market Clerk	33111	p	Financial Dealer and Broker
6145-11		Money Market Clerk			41221	p	Finance Clerk
6145-01	p	Supervisor, Money Market and Statistical Clerks	552314	Statistical Clerk	41222	p	Statistical Clerk
6145-13		Statistical Clerk					
8115-11		Betting Agency Counter Clerk	561111	Betting Agency Counter Clerk	42113	p	Ticket-Seller
8115-15		Bookmaker's Clerk	561112	Bookmaker's Clerk	42113	p	Ticket-Seller
8115-13		Telephone Betting Clerk	561113	Telephone Betting Clerk	42113	p	Ticket-Seller
8115-79		Betting Clerks nec	561199	Betting Clerks nec	42113	p	Ticket-Seller
7314-11	p	Delivery Driver	561211	Courier	41424	p	Mail Delivery Contractor
8114-11		Courier			91211	p	Courier and Deliverer
6199-15	p	Mail Supervisor	561212	Postal Delivery Officer	41423		Postal Deliverer
8114-13		Postal Delivery Officer			41424	p	Mail Delivery Contractor
					91211	p	Courier and Deliverer
8111-11		Registry or Filing Clerk	561311	Filing or Registry Clerk	41412	p	Record and Filing Clerk
6199-15	p	Mail Supervisor	561411	Mail Clerk	41422		Mail Clerk
8112-11		Mail Clerk					
6199-15	p	Mail Supervisor	561412	Postal Sorting Officer	41421		Mail Sorting Clerk
8112-13		Postal Sorting Officer					
8119-15		Interviewer	561511	Survey Interviewer	41223	p	Survey Interviewer
8113-11	p	Switchboard Operator	561611	Switchboard Operator	42221	p	Telephone Switchboard Operator
8119-13		Classified Advertising Clerk	561911	Classified Advertising Clerk	41443	p	General Clerk
8119-11		Meter Reader	561912	Meter Reader	41213	p	Costing Clerk
8119-17		Parking Inspector	561913	Parking Inspector	51522	p	Police Officer
8116-11		Office Trainee	561999	Clerical and Office Support Workers nec	41443	p	General Clerk
8119-79		Elementary Clerks nec			41444		Office Machine Operator
6153-01	p	Supervisor, Stock and Purchasing Clerks	591111	Order Clerk	41311	p	Stock Clerk
6153-15		Order Clerk			52111	p	Sales Assistant
6151-11		Production Recording Clerk	591112	Production Clerk	41321		Material and Production Planning Clerk
					41331	p	Transport Clerk
					91412	p	Loader and/or Checker
6153-01	p	Supervisor, Stock and Purchasing Clerks	591113	Purchasing Officer	33163		Purchasing Agent
6153-13		Purchasing Officer			41311	p	Stock Clerk
6153-01	p	Supervisor, Stock and Purchasing Clerks	591114	Sales Clerk	41311	p	Stock Clerk
6153-17		Sales Clerk			52111	p	Sales Assistant
6153-01	p	Supervisor, Stock and Purchasing Clerks	591115	Stock Clerk	41311	p	Stock Clerk
6153-11		Stock Clerk			83411	p	Deck Rating
6153-01	p	Supervisor, Stock and Purchasing Clerks	591116	Warehouse Administrator	12261	p	Supply and Distribution Manager
7993-01	p	Supervisor, Storepersons			12262	p	Wholesale and Warehouse Manager
					41311	p	Stock Clerk
6152-01	p	Supervisor, Transport and Despatching Clerks	591211	Despatching and Receiving Clerk	41312	p	Dispatch and Receiving Clerk
6152-11		Receiving and Despatching Clerk			41331	p	Transport Clerk
6152-01	p	Supervisor, Transport and Despatching Clerks	591212	Import-Export Clerk	33153	p	Sales Representative
6152-13	p	Import-Export Clerk			33311	p	Customs Officer
					41312	p	Dispatch and Receiving Clerk
					41313	p	Weighing and Tally Clerk
5991-13	p	Law Clerk	599111	Conveyancer	33222	p	Legal Clerk
5991-13	p	Law Clerk	599112	Legal Executive	24231	p	Other Legal Professional
					33221	p	Legal Executive
5991-11		Clerk of Court	599211	Clerk of Court	24231	p	Other Legal Professional
					33222	p	Legal Clerk
6199-19		Bailiff or Sheriff	599212	Court Bailiff or Sheriff (Aus) / Court Collections Officer (NZ)	24231	p	Other Legal Professional
					42151	p	Bill and Debt Collector
6199-17		Court Orderly	599213	Court Orderly (Aus) / Court Registry Officer (NZ)	51236	p	Usher and Cloakroom Attendant
					51542	p	Security Officer
5991-13	p	Law Clerk	599214	Law Clerk	33221	p	Legal Executive
					33222	p	Legal Clerk
5991-15		Trust Officer	599215	Trust Officer	33222	p	Legal Clerk

ASCO Second Edition			ANZSCO		NZSCO 1999		
6199-13		Debt Collector	599311	Debt Collector	41211	p	Accounts Clerk
					42151	p	Bill and Debt Collector
6193-11		Personnel Records Clerk	599411	Human Resource Clerk	41445	p	Human Resources Clerk
6193-13		Employment Office Clerk					
6152-13	p	Import-Export Clerk	599511	Customs Officer	33311	p	Customs Officer
6194-11		Customs Inspector					
6194-79	p	Intermediate Inspectors and Examiners nec	599512	Immigration Officer	33313		Immigration Officer
6199-79	p	Intermediate Clerical Workers nec					
6194-17		Motor Vehicle Licence Examiner	599513	Motor Vehicle Licence Examiner	41443	p	General Clerk
3991-11	p	Primary Products Inspector	599514	Noxious Weeds and Pest Inspector	31513		Noxious Weeds/Pest Inspector
6194-79	p	Intermediate Inspectors and Examiners nec					
6194-15		Social Security Assessor	599515	Social Security Assessor	33121	p	Insurance Representative
6194-13		Taxation Inspector	599516	Taxation Inspector	12224	p	Finance Manager
					24111	p	Accountant
					24113	p	Auditor
6194-21		Train Examiner	599517	Train Examiner	41331	p	Transport Clerk
6194-19		Transport Operations Inspector	599518	Transport Operations Inspector	41331	p	Transport Clerk
6194-23		Water Inspector	599521	Water Inspector	31514	p	Health Inspector
6194-79	p	Intermediate Inspectors and Examiners nec	599599	Inspectors and Regulatory Officers nec	31191	p	Other Engineering Technician
					31511	p	Safety Inspector
					31516		Quality Inspector
					32111	p	Life Science Technician
					61441	p	Animal Welfare Worker
					72316	p	Mechanical Products Inspector and Tester
5994-13		Insurance Investigator	599611	Insurance Investigator	33121	p	Insurance Representative
5994-15		Insurance Loss Adjuster	599612	Insurance Loss Adjuster	33121	p	Insurance Representative
5994-11		Insurance Risk Surveyor	599613	Insurance Risk Surveyor	33121	p	Insurance Representative
6192-11		Library Assistant	599711	Library Assistant	41411	p	Library Assistant
6199-21		Coding Clerk	599911	Coding Clerk	41121	p	Data Entry Operator
4992-23		Production Assistant (Film, Television or Radio)	599912	Production Assistant (Film, Television, Radio or Stage)	33662	p	Artistic Director
4992-25		Production Assistant (Theatre)					
6199-11		Proof Reader	599913	Proof Reader	41432		Proof-Reader
5999-11		Radio Despatcher	599914	Radio Despatcher	31322	p	Radio Operator
3999-79	p	Associate Professionals nec	599999	Clerical and Administrative Workers nec	33141	p	Travel Consultant
6199-79	p	Intermediate Clerical Workers nec			41443	p	General Clerk
8319-25		Examination Supervisor					
			**6**	**SALES WORKERS**			
5999-13		Auctioneer	611111	Auctioneer	33181		Auctioneer
3399-23		Stock and Station Agent	611112	Stock and Station Agent	33162		Livestock Buyer
					33191		Stock and Station Agent
5993-11		Insurance Agent	611211	Insurance Agent	33121	p	Insurance Representative
6211-15	p	Sales Representative (Builder's and Plumber's Supplies)	611311	Sales Representative (Building and Plumbing Supplies)	33153	p	Sales Representative
6211-13		Sales Representative (Business Services)	611312	Sales Representative (Business Services)	33151	p	Business Services Representative
6211-17	p	Sales Representative (Motor Vehicle Parts and Accessories)	611313	Sales Representative (Motor Vehicle Parts and Accessories)	33153	p	Sales Representative
6211-11	p	Sales Representative (Personal and Household Goods)	611314	Sales Representative (Personal and Household Goods)	33153	p	Sales Representative
6211-79	p	Sales Representatives nec	611399	Sales Representatives nec	12262	p	Wholesale and Warehouse Manager
					33151	p	Business Services Representative
					33152	p	Technical Representative
					33153	p	Sales Representative
					33161	p	Wholesale and/or Retail Buyer
3212-79	p	Financial Dealers and Brokers nec	612111	Business Broker	24133	p	Financial Adviser
3293-13	p	Property Manager	612112	Property Manager	12223	p	Property Manager
					33132	p	Property Developer
3293-11	p	Real Estate Agency Manager	612113	Real Estate Agency Principal (Aus) / Real Estate Agency Licensee (NZ)	12263	p	Retail Manager
					33131	p	Real Estate Agent/Property Consultant
3293-11	p	Real Estate Agency Manager	612114	Real Estate Agent	33131	p	Real Estate Agent/Property Consultant
3293-13	p	Property Manager			33132	p	Property Developer
3293-15		Real Estate Salesperson	612115	Real Estate Representative	33131	p	Real Estate Agent/Property Consultant

ASCO Second Edition			ANZSCO		NZSCO 1999		
8211-11	p	Sales Assistant (Food and Drink Products)	621111	Sales Assistant (General)	12263	p	Retail Manager
8211-13		Sales Assistant (Fabric, Clothing and Footwear)			51234	p	Catering Counter Assistant
8211-15	p	Sales Assistant (Other Personal and Household Goods)			51412	p	Beauty Therapist
8211-17		Sales Assistant (Postal Services)			52111	p	Sales Assistant
8211-79	p	Sales Assistants nec			74311	p	Tailor/Dressmaker
8297-11	p	Sales and Service Trainee					
8211-79	p	Sales Assistants nec	621211	ICT Sales Assistant	33152	p	Technical Representative
8297-11	p	Sales and Service Trainee			52111	p	Sales Assistant
6212-11		Motor Vehicle and Caravan Salesperson	621311	Motor Vehicle or Caravan Salesperson	12263	p	Retail Manager
					52111	p	Sales Assistant
6212-13		Motor Vehicle Parts Interpreter	621312	Motor Vehicle Parts Interpreter (Aus) / Automotive Parts Salesperson (NZ)	12261	p	Supply and Distribution Manager
					52111	p	Sales Assistant
8211-15	p	Sales Assistant (Other Personal and Household Goods)	621411	Pharmacy Sales Assistant	32252	p	Retail Dispensary Assistant
8297-11	p	Sales and Service Trainee			52111	p	Sales Assistant
6213-11	p	Retail Supervisor	621511	Retail Supervisor	42112	p	Checkout Operator
6213-13		Checkout Supervisor			52111	p	Sales Assistant
8296-11		Service Station Attendant	621611	Service Station Attendant	52113		Forecourt Attendant
8293-13		Cash Van Salesperson	621711	Cash Van Salesperson	52211	p	Street Vendor and Related Worker
8293-15		Door-to-Door Salesperson	621712	Door-to-door Salesperson	24134	p	Fundraiser
					33153	p	Sales Representative
					52112	p	Demonstrator
					52211	p	Street Vendor and Related Worker
8293-11		Street Vendor	621713	Street Vendor	52211	p	Street Vendor and Related Worker
8299-13		Materials Recycler	621911	Materials Recycler	12263	p	Retail Manager
					72312	p	Motor Mechanic
					91311	p	Refuse Collector
8299-11		Rental Salesperson	621912	Rental Salesperson	52111	p	Sales Assistant
8299-79		Elementary Sales Workers nec	621999	Sales Assistants and Salespersons nec	24134	p	Fundraiser
					42113	p	Ticket-Seller
					52111	p	Sales Assistant
					52211	p	Street Vendor and Related Worker
8291-11		Checkout Operator	631111	Checkout Operator	42112	p	Checkout Operator
8291-13		Office Cashier	631112	Office Cashier	42111	p	Cashier
8295-13		Model	639111	Model	52311		Fashion and Other Model
6211-11	p	Sales Representative (Personal and Household Goods)	639112	Sales Demonstrator	52112	p	Demonstrator
6211-15	p	Sales Representative (Builder's and Plumber's Supplies)					
6211-17	p	Sales Representative (Motor Vehicle Parts and Accessories)					
6211-79	p	Sales Representatives nec					
8295-11		Sales Demonstrator					
3996-11		Retail Buyer	639211	Retail Buyer	33161	p	Wholesale and/or Retail Buyer
					52111	p	Sales Assistant
3212-17	p	Commodities Trader	639212	Wool Buyer	33164		Wool Buyer/Merchant
8294-11		Telemarketer	639311	Telemarketer	41223	p	Survey Interviewer
8292-11		Ticket Seller	639411	Ticket Seller	33141	p	Travel Consultant
					42113	p	Ticket-Seller
8292-13		Transport Conductor	639412	Transport Conductor	41331	p	Transport Clerk
5999-15		Visual Merchandiser	639511	Visual Merchandiser	33633		Display and Window Dresser
3399-79	p	Managing Supervisors (Sales and Service) nec	639911	Other Sales Support Worker	52111	p	Sales Assistant
8319-79	p	Elementary Service Workers nec					
			**7**	**MACHINERY OPERATORS AND DRIVERS**			
7297-01	p	Supervisor, Clay, Stone and Concrete Processing Machine Operators	711111	Clay Products Machine Operator	81312	p	Clay Product Plant Operator
7297-11		Clay Products Machine Operator			81313	p	Pottery and Porcelain Mould Maker
7297-01	p	Supervisor, Clay, Stone and Concrete Processing Machine Operators	711112	Concrete Products Machine Operator	82121	p	Concrete Worker
7297-15		Concrete Products Machine Operator					
7296-01		Supervisor, Glass Production Machine Operators	711113	Glass Production Machine Operator	73211	p	Glass Cutter and Beveller
7296-11		Glass Production Machine Operator			81311	p	Non-Metallic Mineral Products Kiln or Furnace Operator
					81321	p	Glass Pressing and Drawing Machine Operator

ASCO Second Edition			ANZSCO		NZSCO 1999		
7297-01	p	Supervisor, Clay, Stone and Concrete Processing Machine Operators	711114	Stone Processing Machine Operator	71112	p	Stonemason
7297-13		Stone Processing Machine Operator			81121	p	Mineral and Stone Treater
7297-01	p	Supervisor, Clay, Stone and Concrete Processing Machine Operators	711199	Clay, Concrete, Glass and Stone Processing Machine Operators nec	81312	p	Clay Product Plant Operator
7297-79		Clay, Stone and Concrete Processing Machine Operators nec			81313	p	Pottery and Porcelain Mould Maker
					81323		Glass and Ceramics Painter and Decorator
					82121	p	Concrete Worker
7299-11		Industrial Spray Painter	711211	Industrial Spraypainter	71242	p	Spray Painter
7295-11		Paper Products Machine Operator	711311	Paper Products Machine Operator	82511		Paper Products Machine Operator
					82512		Cardboard Forme Maker and Finisher
					82513		Guillotine Operator
7294-01		Supervisor, Wood Processing Machine Operators	711312	Wood Processing Machine Operator	74211	p	Cabinetmaker
7294-11		Wood Processing Machine Operator			81411	p	Timber Processing Machine Operator
					81413	p	Timber Grader, Classer
					82411	p	Woodworking Machinist
					82412	p	Wood Panel Production Worker
					82941	p	Wood and Related Materials Products Assembler
7298-11		Photographic Developer and Printer	711411	Photographic Developer and Printer	82231	p	Photographic Darkroom Operator
7291-01	p	Supervisor, Plastics Production Machine Operators	711511	Plastic Cablemaking Machine Operator	82322	p	Plastics Machine Operator
7291-17	p	Plastic Cablemaking Machine Operator					
7291-01	p	Supervisor, Plastics Production Machine Operators	711512	Plastic Compounding and Reclamation Machine Operator	82322	p	Plastics Machine Operator
7291-13	p	Plastics Compounding and Reclamation Machine Operator					
7291-01	p	Supervisor, Plastics Production Machine Operators	711513	Plastics Fabricator or Welder	82322	p	Plastics Machine Operator
7291-79	p	Plastics Production Machine Operators nec			82932	p	Plastic and Rubber Goods Assembler
7291-01	p	Supervisor, Plastics Production Machine Operators	711514	Plastics Production Machine Operator (General)	82322	p	Plastics Machine Operator
7291-11	p	General Plastics Production Machine Operator			82323	p	Plastics Laminator
7291-01	p	Supervisor, Plastics Production Machine Operators	711515	Reinforced Plastic and Composite Production Worker	81321	p	Glass Pressing and Drawing Machine Operator
7291-15	p	Reinforced Plastic and Composite Production Worker			82322	p	Plastics Machine Operator
7292-01		Supervisor, Rubber Production Machine Operators	711516	Rubber Production Machine Operator	82311		Tyre Moulder and Builder
7292-11		Tyre Production Machine Operator			82313	p	Tyre Retreader
7292-79		Rubber Production Machine Operators nec			82321	p	Rubber Machine Operator
					82932	p	Plastic and Rubber Goods Assembler
7291-01	p	Supervisor, Plastics Production Machine Operators	711599	Plastics and Rubber Production Machine Operators nec	82322	p	Plastics Machine Operator
7291-79	p	Plastics Production Machine Operators nec					
7211-01		Supervisor, Sewing Machinists	711611	Sewing Machinist	74311	p	Tailor/Dressmaker
7211-11		Sewing Machinist			74321	p	Furniture Upholsterer
					74323	p	Canvas Worker
					82631	p	Sewing Machinist
					82632		Embroiderer
					82634		Stuffed Toy Maker
7212-01	p	Supervisor, Textile and Footwear Production Machine Operators	711711	Footwear Production Machine Operator	82954	p	Footwear Production Machine Operator
7212-21		Footwear Production Machine Operator					
7212-01	p	Supervisor, Textile and Footwear Production Machine Operators	711712	Hide and Skin Processing Machine Operator	82811	p	Hide and Pelt Processor
7212-19		Hide and Skin Processing Machine Operator			82812	p	Tanner, Splitter and Dyer
9219-19	p	Hide and Skin Processing Labourer			82953	p	Leather Goods Assembler
7212-01	p	Supervisor, Textile and Footwear Production Machine Operators	711713	Knitting Machine Operator	82624	p	Knitter, Knitting Machinist
7212-15		Knitting Machine Operator					
7212-01	p	Supervisor, Textile and Footwear Production Machine Operators	711714	Textile Dyeing and Finishing Machine Operator	82642		Bleacher and Dyer
7212-17		Textile Dyeing and Finishing Machine Operator			82645		Textile Finisher
					82652		Wool Scourer

ASCO Second Edition			ANZSCO		NZSCO 1999		
7212-01	p	Supervisor, Textile and Footwear Production Machine Operators	711715	Weaving Machine Operator	82622	p	Cloth Weaver
7212-13		Weaving Machine Operator			82623	p	Carpet Weaver
7212-01	p	Supervisor, Textile and Footwear Production Machine Operators	711716	Yarn Carding and Spinning Machine Operator	82611	p	Spinner and Winder
7212-11		Yarn Carding and Spinning Machine Operator			82651		Fibre Preparer
7212-01	p	Supervisor, Textile and Footwear Production Machine Operators	711799	Textile and Footwear Production Machine Operators nec	82611	p	Spinner and Winder
7212-79		Textile and Footwear Production Machine Operators nec			82622	p	Cloth Weaver
					82623	p	Carpet Weaver
					82631	p	Sewing Machinist
7293-01		Supervisor, Chemical Production Machine Operators	711911	Chemical Production Machine Operator	81511	p	Chemical Crushing, Grinding and Mixing Operator
7293-11		Chemical Production Machine Operator			81521	p	Filtering and Separating Equipment Operator
					81531	p	Still and Reactor Operator
					81541	p	Other Chemical Processing Plant Operator
					82211	p	Pharmaceutical and Toiletry Products Machine Operator
4992-19		Motion Picture Projectionist	711912	Motion Picture Projectionist	31323		Cinema Projectionist
7299-79	p	Intermediate Machine Operators nec	711913	Sand Blaster	81221	p	Metal Caster
					84110	p	Building Exterior Cleaner
8319-15		Sterilisation Technology Worker	711914	Sterilisation Technician	82641	p	Launderer
7299-79	p	Intermediate Machine Operators nec	711999	Machine Operators nec	72122	p	Sheet-Metal Worker
					82111	p	Machine Tool Operator
					82112	p	Automated Machine Operator
					82231	p	Photographic Darkroom Operator
					82932	p	Plastic and Rubber Goods Assembler
					91411	p	Packer
					91412	p	Loader and/or Checker
					91514	p	General Labourer
7122-11		Crane, Hoist or Lift Operator	712111	Crane, Hoist or Lift Operator	83111	p	Railway Locomotive Driver
					83323		Pile Driver, Driller Operator
					83331		Crane Operator
4986-01		Supervisor, Drillers	712211	Driller	81133	p	Driller
4986-11		Driller					
7911-11		Miner	712212	Miner	81111	p	Quarry and Mine Worker
					81112		Mining Plant Operator
					83321	p	Excavating Machine Operator
7912-11		Blasting Worker	712213	Shot Firer	81111	p	Quarry and Mine Worker
7123-01		Supervisor, Engineering Production Systems Workers	712311	Engineering Production Systems Worker	72122	p	Sheet-Metal Worker
7123-11		Engineering Production Systems Worker			72311	p	Machinery Mechanic
					81121	p	Mineral and Stone Treater
					81211		Metallic Furnace Operator
					81212		Drop Hammer and Forging Press Operator
					81221	p	Metal Caster
					81231	p	Welder and Flame-Cutter
					81241		Metal Drawer and/or Extruder
					81311	p	Non-Metallic Mineral Products Kiln or Furnace Operator
					81521	p	Filtering and Separating Equipment Operator
					82111	p	Machine Tool Operator
					82112	p	Automated Machine Operator
					82114	p	Tool Grinder and Sharpener
					82115		Power Shear Operator
7121-11		Engine or Boiler Operator	712911	Boiler or Engine Operator	81612		Boiler Attendant
7129-01	p	Supervisor, Other Intermediate Stationary Plant Operators	712912	Bulk Materials Handling Plant Operator	91412	p	Loader and/or Checker
7129-23		Bulk Materials Handling Plant Operator					
7129-01	p	Supervisor, Other Intermediate Stationary Plant Operators	712913	Cement Production Plant Operator	81511	p	Chemical Crushing, Grinding and Mixing Operator
7129-17		Cement Production Plant Operator			82121	p	Concrete Worker
7129-01	p	Supervisor, Other Intermediate Stationary Plant Operators	712914	Concrete Batching Plant Operator	82121	p	Concrete Worker
7129-19		Concrete Batching Plant Operator					
7129-01	p	Supervisor, Other Intermediate Stationary Plant Operators	712915	Concrete Pump Operator	82121	p	Concrete Worker
7129-15		Concrete Pump Operator					
7124-11		Pulp Mill Operator	712916	Paper and Pulp Mill Operator	81421	p	Pulp Production Worker
7124-13		Paper Mill Operator			81431	p	Paper Production Worker

ASCO Second Edition			ANZSCO		NZSCO 1999		
7129-01	p	Supervisor, Other Intermediate Stationary Plant Operators	712917	Railway Signal Operator	91413		Railway Shunter
7129-11		Railway Signal Operator					
7129-01	p	Supervisor, Other Intermediate Stationary Plant Operators	712918	Train Controller	41331	p	Transport Clerk
7129-13		Train Controller					
7129-01	p	Supervisor, Other Intermediate Stationary Plant Operators	712921	Waste Water or Water Plant Operator	81522		Water Treatment Plant Operator
7129-21		Water and Waste Water Plant Operator			81613	p	Pumping-Station Operator
7129-01	p	Supervisor, Other Intermediate Stationary Plant Operators	712922	Weighbridge Operator	41313	p	Weighing and Tally Clerk
7129-79	p	Intermediate Stationary Plant Operators nec					
7129-01	p	Supervisor, Other Intermediate Stationary Plant Operators	712999	Stationary Plant Operators nec	51431	p	Funeral Director
7129-79	p	Intermediate Stationary Plant Operators nec			81541	p	Other Chemical Processing Plant Operator
					81614		Other Stationary Engine Operator
					82112	p	Automated Machine Operator
					82421		Preservation Plant Operator
					82422		Wood Seasoning Kiln Operator
					82751	p	Fruit, Vegetable and Nut Processing Machine Operator
					82792		Distillery Worker (Alcoholic Beverages)
					91514	p	General Labourer
7119-11		Agricultural and Horticultural Mobile Plant Operator	721111	Agricultural and Horticultural Mobile Plant Operator	83311	p	Farm Machinery Operator, Including Contractor
					83312	p	Ground Spraying and/or Dusting Contractor
7119-17		Logging Plant Operator	721112	Logging Plant Operator	61311	p	Logger
7111-11		General Construction Plant Operator	721211	Earthmoving Plant Operator (General)	83324	p	Earthmoving Machine Operator
7111-15		Backhoe Operator	721212	Backhoe Operator	83324	p	Earthmoving Machine Operator
7111-13		Bulldozer Operator	721213	Bulldozer Operator	83324	p	Earthmoving Machine Operator
7111-21		Excavator Operator	721214	Excavator Operator	83321	p	Excavating Machine Operator
7111-19		Grader Operator	721215	Grader Operator	83325	p	Roading and/or Paving Machine Operator
7111-17		Loader Operator	721216	Loader Operator	83324	p	Earthmoving Machine Operator
					91412	p	Loader and/or Checker
7112-11		Forklift Driver	721311	Forklift Driver	83341		Forklift Operator
					83342	p	Straddle-Truck Operator
7119-79	p	Mobile Plant Operators nec	721911	Aircraft Baggage Handler and Airline Ground Crew	91412	p	Loader and/or Checker
7119-79	p	Mobile Plant Operators nec	721912	Linemarker	71242	p	Spray Painter
7111-23		Paving Plant Operator	721913	Paving Plant Operator	83325	p	Roading and/or Paving Machine Operator
7119-13		Railway Track Repair Mobile Plant Operator	721914	Railway Track Plant Operator	91514	p	General Labourer
7111-25		Road Roller Operator	721915	Road Roller Operator	83324	p	Earthmoving Machine Operator
					83325	p	Roading and/or Paving Machine Operator
7119-15		Streetsweeper Operator	721916	Streetsweeper Operator	91312		Street or Park Cleaner
7119-79	p	Mobile Plant Operators nec	721999	Mobile Plant Operators nec	51551	p	Armed Forces
					82112	p	Automated Machine Operator
					83311	p	Farm Machinery Operator, Including Contractor
					83321	p	Excavating Machine Operator
					83342	p	Straddle-Truck Operator
					83412	p	Other Ship or Boat Hand
					91412	p	Loader and/or Checker
					91514	p	General Labourer
7313-11	p	Automobile Driver	731111	Chauffeur	83211	p	Taxi Driver
7313-11	p	Automobile Driver	731112	Taxi Driver	83211	p	Taxi Driver
7313-11	p	Automobile Driver	731199	Automobile Drivers nec	83211	p	Taxi Driver
7312-11	p	Bus Driver	731211	Bus Driver	83221	p	Passenger Coach Driver
7312-11	p	Bus Driver	731212	Charter and Tour Bus Driver	83221	p	Passenger Coach Driver
7312-11	p	Bus Driver	731213	Passenger Coach Driver	83221	p	Passenger Coach Driver
7315-11		Train Driver	731311	Train Driver	83111	p	Railway Locomotive Driver
7315-13		Train Driver's Assistant					
7312-13		Tram Driver	731312	Tram Driver	83221	p	Passenger Coach Driver
7314-11	p	Delivery Driver	732111	Delivery Driver	41443	p	General Clerk
					51542	p	Security Officer
					83211	p	Taxi Driver
					83212	p	Light Truck or Van Driver
					91211	p	Courier and Deliverer
7311-11	p	Heavy Truck Driver	733111	Truck Driver (General)	51542	p	Security Officer
					83231	p	Heavy Truck or Tanker Driver
4987-13	p	Petroleum and Gas Plant Operator	733112	Aircraft Refueller	81613	p	Pumping-Station Operator

ASCO Second Edition			ANZSCO		NZSCO 1999		
7311-13		Furniture Removalist	733113	Furniture Removalist	91412	p	Loader and/or Checker
7311-11	p	Heavy Truck Driver	733114	Tanker Driver	83231	p	Heavy Truck or Tanker Driver
7311-11	p	Heavy Truck Driver	733115	Tow Truck Driver	83343		Tow Truck Operator
7993-01	p	Supervisor, Storepersons	741111	Storeperson	41311	p	Stock Clerk
7993-11	p	Storeperson			41312	p	Dispatch and Receiving Clerk
					51231	p	Bartender
					82712	p	Slaughterer
					83212	p	Light Truck or Van Driver
					91411	p	Packer
					91412	p	Loader and/or Checker
			**8**	**LABOURERS**			
9111-17		Vehicle Cleaner	811111	Car Detailer	91111	p	Cleaner
9111-11	p	Commercial Cleaner	811211	Commercial Cleaner	84110	p	Building Exterior Cleaner
					91111	p	Cleaner
9111-13		Domestic Cleaner	811311	Domestic Cleaner	51312	p	Health Assistant
					91111	p	Cleaner
8319-79	p	Elementary Service Workers nec	811411	Commercial Housekeeper	51212	p	Housekeeper (Not Private)
9111-11	p	Commercial Cleaner					
8313-11		Domestic Housekeeper	811412	Domestic Housekeeper	51211	p	Housekeeper (Private Service)
					51212	p	Housekeeper (Not Private)
8315-11	p	Laundry Worker	811511	Laundry Worker (General)	82641	p	Launderer
					82643	p	Dry-Cleaner
					82644	p	Presser
8315-11	p	Laundry Worker	811512	Drycleaner	82643	p	Dry-Cleaner
8315-11	p	Laundry Worker	811513	Ironer or Presser	82644	p	Presser
9111-15		Carpet Cleaner	811611	Carpet Cleaner	82646		Carpet Cleaner
9111-19		Window Cleaner	811612	Window Cleaner	91111	p	Cleaner
9111-79		Cleaners nec	811699	Cleaners nec	91111	p	Cleaner
9999-79	p	Labourers and Related Workers nec					
9916-11		Construction Assistant	821111	Builder's Labourer	82413		Joiner's Benchhand
					84111	p	Drainlayer
					84112	p	Pipe Fitter
					91512	p	Builder's Labourer
9999-17		Stormwater, Drainage and Sewerage System Labourer	821112	Drainage, Sewerage and Stormwater Labourer	91514	p	General Labourer
9912-11		Earthmoving Labourer	821113	Earthmoving Labourer	91514	p	General Labourer
9916-13		Plumber's Assistant	821114	Plumber's Assistant	91512	p	Builder's Labourer
9917-11		Concreter	821211	Concreter	82121	p	Concrete Worker
9919-15		Fence Erector	821311	Fencer	82944		Fencer
					91514	p	General Labourer
7914-11		Building Insulation Installer	821411	Building Insulation Installer	84119	p	Insulator
7914-13	p	Home Improvements Installer	821412	Home Improvement Installer	52111	p	Sales Assistant
					71122	p	Builder (including Contractor)
					74323	p	Canvas Worker
					91512	p	Builder's Labourer
9913-11		Paving and Surfacing Labourer	821511	Paving and Surfacing Labourer	83325	p	Roading and/or Paving Machine Operator
					91514	p	General Labourer
9915-11		Railway Labourer	821611	Railway Track Worker	91514	p	General Labourer
7913-17		Construction Rigger	821711	Construction Rigger	84115	p	Rigger and Cable Splicer
7913-11		Scaffolder	821712	Scaffolder	84114		Scaffolder
7913-13		Steel Fixer	821713	Steel Fixer	84113		Steel Fixer
7913-15		Structural Steel Erector	821714	Structural Steel Erector	84116		Steel Erector, Construction
9919-13		Crane Chaser	821911	Crane Chaser	91514	p	General Labourer
9911-13		Driller's Assistant	821912	Driller's Assistant	81133	p	Driller
9919-11		Lagger	821913	Lagger	84119	p	Insulator
9911-11		Mining Support Worker	821914	Mining Support Worker	81111	p	Quarry and Mine Worker
9914-11		Survey Hand	821915	Surveyor's Assistant	91511		Surveyor's Assistant
9214-11	p	Food and Drink Processing Machine Attendant	831111	Baking Factory Worker	82741	p	Baked Goods and Cereals Producing Machine Operator
9214-13		Baking Factory Hand			82742	p	Baker's Assistant
9214-11	p	Food and Drink Processing Machine Attendant	831112	Brewery Worker	82791		Brewery Worker
9214-79	p	Food Factory Hands nec					
4519-15		Confectioner	831113	Confectionery Maker	82762		Confectionery Maker
4519-85		Apprentice Confectioner			82773	p	Other Food Products Processing Machine Operator
9214-11	p	Food and Drink Processing Machine Attendant					

ASCO Second Edition			ANZSCO		NZSCO 1999		
4519-13		Buttermaker or Cheesemaker	831114	Dairy Products Maker	82721	p	Milk and Other Dairy Products Maker
4519-83		Apprentice Buttermaker or Cheesemaker			82722		Cheese Maker
9214-11	p	Food and Drink Processing Machine Attendant					
9214-15		Dairy Factory Hand					
9214-11	p	Food and Drink Processing Machine Attendant	831115	Fruit and Vegetable Factory Worker	82751	p	Fruit, Vegetable and Nut Processing Machine Operator
9214-17		Fruit and Vegetable Factory Hand			82794	p	Wine Making Machine Operator
4519-11		Miller	831116	Grain Mill Worker	82731	p	Grain Miller
4519-81		Apprentice Miller					
9214-11	p	Food and Drink Processing Machine Attendant					
9214-19	p	Food Products Millhand					
9214-11	p	Food and Drink Processing Machine Attendant	831117	Sugar Mill Worker	82761		Sugar Processor and Refiner
9214-19	p	Food Products Millhand					
9214-79	p	Food Factory Hands nec	831118	Winery Cellar Hand	51231	p	Bartender
					82794	p	Wine Making Machine Operator
9214-11	p	Food and Drink Processing Machine Attendant	831199	Food and Drink Factory Workers nec	82721	p	Milk and Other Dairy Products Maker
9214-79	p	Food Factory Hands nec			82731	p	Grain Miller
					82741	p	Baked Goods and Cereals Producing Machine Operator
					82751	p	Fruit, Vegetable and Nut Processing Machine Operator
					82773	p	Other Food Products Processing Machine Operator
					82781		Tobacco Product Process Worker
9213-13		Meat Boner and Slicer	831211	Meat Boner and Slicer	82712	p	Slaughterer
4511-01	p	Supervisor, Meat Tradespersons	831212	Slaughterer	82712	p	Slaughterer
4511-15		Slaughterperson					
4511-85		Apprentice Slaughterperson					
9213-11		Meatworks Labourer	831311	Meat Process Worker	82712	p	Slaughterer
9214-11	p	Food and Drink Processing Machine Attendant			82713	p	Smallgoods Maker
					82717	p	Meat Processing Worker
9213-15		Poultry Process Worker	831312	Poultry Process Worker	82712	p	Slaughterer
					82717	p	Meat Processing Worker
9213-17	p	Fish Process Worker	831313	Seafood Process Worker	82715		Oyster Opener and Canner
9214-11	p	Food and Drink Processing Machine Attendant			82718		Fish Processing Worker
9221-11	p	Hand Packer	832111	Chocolate Packer	91411	p	Packer
9222-11	p	Packager and Container Filler					
9221-11	p	Hand Packer	832112	Container Filler	91411	p	Packer
9222-11	p	Packager and Container Filler					
9221-11	p	Hand Packer	832113	Fruit and Vegetable Packer	91411	p	Packer
9222-11	p	Packager and Container Filler					
9221-11	p	Hand Packer	832114	Meat Packer	91411	p	Packer
9222-11	p	Packager and Container Filler					
9213-17	p	Fish Process Worker	832115	Seafood Packer	91411	p	Packer
9221-11	p	Hand Packer					
9222-11	p	Packager and Container Filler					
9221-11	p	Hand Packer	832199	Packers nec	91411	p	Packer
9222-11	p	Packager and Container Filler					
9212-11		Product Assembler	832211	Product Assembler	71221	p	Glazier
					82911	p	Machinery Assembler
					82922		Electric and Electronic Equipment Assembler
					82931		Metal Goods Assembler
					82932	p	Plastic and Rubber Goods Assembler
					82941	p	Wood and Related Materials Products Assembler
					82953	p	Leather Goods Assembler
					84118		Aluminium Joiner
9211-11		Engineering Production Process Worker	839111	Metal Engineering Process Worker	72211	p	Blacksmith
					81221	p	Metal Caster
					82911	p	Machinery Assembler
9219-17	p	Rubber and Plastics Factory Hand	839211	Plastics Factory Worker	82323	p	Plastics Laminator
					82932	p	Plastic and Rubber Goods Assembler
9219-17	p	Rubber and Plastics Factory Hand	839212	Rubber Factory Worker	82321	p	Rubber Machine Operator
					91411	p	Packer
7992-11		Product Examiner	839311	Product Examiner	74311	p	Tailor/Dressmaker
					82622	p	Cloth Weaver
					97000		Response Unidentifiable

ASCO Second Edition			ANZSCO		NZSCO 1999		
7992-13		Product Grader	839312	Product Grader	61112	p	Market Gardener and Related Worker
					61121	p	Fruit Grower, Worker
					74112		Meat Grader
					82811	p	Hide and Pelt Processor
7992-15		Product Tester	839313	Product Tester	72316	p	Mechanical Products Inspector and Tester
9215-17		Pulp and Paper Mill General Hand	839411	Paper and Pulp Mill Worker	81421	p	Pulp Production Worker
9215-11		Sawmill Labourer	839412	Sawmill or Timber Yard Worker	81411	p	Timber Processing Machine Operator
9215-13		Timberyard Labourer			81413	p	Timber Grader, Classer
					91513	p	Sawmill Labourer
9215-15		Wood and Wood Products Factory Hand	839413	Wood and Wood Products Factory Worker	81431	p	Paper Production Worker
					82412	p	Wood Panel Production Worker
					82941	p	Wood and Related Materials Products Assembler
					91513	p	Sawmill Labourer
9219-11		Cement and Concrete Plant Labourer	839911	Cement and Concrete Plant Worker	82121	p	Concrete Worker
9219-15		Chemical Plant Labourer	839912	Chemical Plant Worker	81541	p	Other Chemical Processing Plant Operator
9219-13		Clay Processing Factory Hand	839913	Clay Processing Factory Worker	81312	p	Clay Product Plant Operator
9219-23		Fabric and Textile Factory Hand	839914	Fabric and Textile Factory Worker	74313	p	Textile Products Marker and Cutter
					74323	p	Canvas Worker
					82611	p	Spinner and Winder
					82622	p	Cloth Weaver
					82624	p	Knitter, Knitting Machinist
9219-25		Footwear Factory Hand	839915	Footwear Factory Worker	82954	p	Footwear Production Machine Operator
9219-21		Glass Processing Labourer	839916	Glass Processing Worker	81321	p	Glass Pressing and Drawing Machine Operator
9219-19	p	Hide and Skin Processing Labourer	839917	Hide and Skin Processing Worker	82811	p	Hide and Pelt Processor
					82812	p	Tanner, Splitter and Dyer
9219-79		Process Workers nec	839999	Factory Process Workers nec	82942	p	Basket and Wicker Worker
4611-11	p	Farm Overseer	841111	Aquaculture Worker	61413	p	Shell Fisher
9921-79	p	Farm Hands nec			61421	p	Fish Farmer, Worker
9929-13	p	Rural Trainee			61422	p	Mussel and Oyster Farmer, Worker
9929-79	p	Agricultural and Horticultural Labourers nec					
4611-11	p	Farm Overseer	841211	Fruit or Nut Farm Worker	61121	p	Fruit Grower, Worker
9921-13	p	Fruit, Vegetable or Nut Farm Hand			61251	p	Crop and Livestock Farmer, Worker
9929-13	p	Rural Trainee					
9921-13	p	Fruit, Vegetable or Nut Farm Hand	841212	Fruit or Nut Picker	61121	p	Fruit Grower, Worker
4611-11	p	Farm Overseer	841213	Grain, Oilseed or Pasture Farm Worker (Aus) / Field Crop Farm Worker (NZ)	61111	p	Field Crop Grower and Related Worker
9921-11	p	General Farm Hand					
9929-13	p	Rural Trainee					
4611-11	p	Farm Overseer	841214	Vegetable Farm Worker (Aus) / Market Garden Worker (NZ)	61111	p	Field Crop Grower and Related Worker
9921-13	p	Fruit, Vegetable or Nut Farm Hand			61112	p	Market Gardener and Related Worker
9929-13	p	Rural Trainee					
9921-13	p	Fruit, Vegetable or Nut Farm Hand	841215	Vegetable Picker	61112	p	Market Gardener and Related Worker
4611-11	p	Farm Overseer	841216	Vineyard Worker	61122	p	Grape Grower and/or Wine Maker, Worker
9921-13	p	Fruit, Vegetable or Nut Farm Hand					
9929-13	p	Rural Trainee					
4611-11	p	Farm Overseer	841299	Crop Farm Workers nec	61111	p	Field Crop Grower and Related Worker
9921-11	p	General Farm Hand			61251	p	Crop and Livestock Farmer, Worker
9921-13	p	Fruit, Vegetable or Nut Farm Hand					
9921-79	p	Farm Hands nec					
9929-13	p	Rural Trainee					
9929-79	p	Agricultural and Horticultural Labourers nec					
7995-01	p	Supervisor, Forestry and Logging Workers	841311	Forestry Worker	61312		Forest Hand
7995-13		Forestry Worker			61313	p	Forestry Contractor
7995-15		Logging Assistant	841312	Logging Assistant	61311	p	Logger
					61313	p	Forestry Contractor
7995-01	p	Supervisor, Forestry and Logging Workers	841313	Tree Faller	61311	p	Logger
7995-11		Tree Faller					
9922-13	p	Garden Labourer	841411	Garden Labourer	61133	p	Grounds or Green Keeper
					61134	p	Gardener
9922-11		Horticultural Nursery Assistant	841412	Horticultural Nursery Assistant	61131	p	Nursery Grower, Nursery Worker
9922-13	p	Garden Labourer					
4611-11	p	Farm Overseer	841511	Beef Cattle Farm Worker	61213	p	Cattle Farmer, Cattle Farm Worker
9921-11	p	General Farm Hand					
9921-15	p	Stud Hand or Stable Hand					
9929-13	p	Rural Trainee					

APPENDIX A SUMMARY CORRESPONDENCE BETWEEN ANZSCO AND ASCO SECOND EDITION AND NZSCO 1999 *continued*

ASCO Second Edition			ANZSCO		NZSCO 1999		
4611-11	p	Farm Overseer	841512	Dairy Cattle Farm Worker	61211	p	Dairy Farmer, Dairy Farm Worker
9921-11	p	General Farm Hand					
9921-15	p	Stud Hand or Stable Hand					
9929-13	p	Rural Trainee					
4611-11	p	Farm Overseer	841513	Mixed Livestock Farm Worker	61221	p	Mixed Livestock Farmer, Mixed Livestock Farm Worker
9921-11	p	General Farm Hand					
9929-13	p	Rural Trainee					
4611-11	p	Farm Overseer	841514	Poultry Farm Worker	61231	p	Poultry Farmer and Poultry Farm Worker
9921-79	p	Farm Hands nec					
9929-13	p	Rural Trainee					
4611-11	p	Farm Overseer	841515	Sheep Farm Worker	61212	p	Sheep Farmer, Sheep Farm Worker
9921-11	p	General Farm Hand			61261	p	Shepherd or Musterer
9921-15	p	Stud Hand or Stable Hand					
9929-13	p	Rural Trainee					
4611-11	p	Farm Overseer	841516	Stablehand	33691	p	Professional Sportsperson
9921-15	p	Stud Hand or Stable Hand			61217	p	Stud Racehorse Breeder, Stud Worker
9929-13	p	Rural Trainee			61265	p	Horse Trainer, Groom or Stable Hand
9921-17		Shearing Shed Hand	841517	Wool Handler	61264		Shearing Shed Hand
4611-11	p	Farm Overseer	841599	Livestock Farm Workers nec	61214	p	Pig Farmer, Pig Farm Worker
9921-11	p	General Farm Hand			61215	p	Goat Farmer, Goat Farm Worker
9921-15	p	Stud Hand or Stable Hand			61216	p	Deer Farmer, Deer Farm Worker
9921-79	p	Farm Hands nec			61218	p	Other Livestock Farmer, Other Livestock Farm Worker
9929-13	p	Rural Trainee			61241	p	Apiarist and Apiary Worker
4611-11	p	Farm Overseer	841611	Mixed Crop and Livestock Farm Worker	61261	p	Shepherd or Musterer
9921-11	p	General Farm Hand					
9929-13	p	Rural Trainee					
9929-11		Shooter-Trapper	841911	Hunter-Trapper	61431	p	Hunter and Trapper
6399-17		Pest and Weed Controller	841912	Pest or Weed Controller	61431	p	Hunter and Trapper
					83312	p	Ground Spraying and/or Dusting Contractor
					91113		Pest Control Worker
9929-79	p	Agricultural and Horticultural Labourers nec	841999	Farm, Forestry and Garden Workers nec	61251	p	Crop and Livestock Farmer, Worker
					61261	p	Shepherd or Musterer
					91514	p	General Labourer
9932-11		Fast Food Cook	851111	Fast Food Cook	51222	p	Cook
9933-11		Pastrycook's Assistant	851211	Pastrycook's Assistant	82742	p	Baker's Assistant
9933-79		Food Trades Assistants nec	851299	Food Trades Assistants nec	51234	p	Catering Counter Assistant
					74111	p	Butcher
9931-11		Kitchenhand	851311	Kitchenhand	51235		Kitchenhand
9992-11		Freight Handler (Road and Rail)	891111	Freight Handler (Rail or Road)	91412	p	Loader and/or Checker
					91514	p	General Labourer
9992-15		Truck Driver's Offsider	891112	Truck Driver's Offsider	91514	p	General Labourer
9992-13		Waterside Worker	891113	Waterside Worker	91412	p	Loader and/or Checker
7993-01	p	Supervisor, Storepersons	891211	Shelf Filler	41311	p	Stock Clerk
7993-11	p	Storeperson			52111	p	Sales Assistant
					91412	p	Loader and/or Checker
8314-11		Caretaker	899111	Caretaker	91112		Building Caretaker
7994-11		Seafarer	899211	Deck Hand	83411	p	Deck Rating
					83412	p	Other Ship or Boat Hand
7994-13		Fishing Hand	899212	Fishing Hand	61411	p	Fishing Skipper, Fisherperson
					61413	p	Shell Fisher
					83411	p	Deck Rating
					83412	p	Other Ship or Boat Hand
9993-11		Handyperson	899311	Handyperson	91514	p	General Labourer
7991-11	p	Motor Vehicle Parts and Accessories Fitter	899411	Motor Vehicle Parts and Accessories Fitter (General)	72421	p	Electronics Servicer
7991-11	p	Motor Vehicle Parts and Accessories Fitter	899412	Autoglazier	71221	p	Glazier
					72312	p	Motor Mechanic
7991-11	p	Motor Vehicle Parts and Accessories Fitter	899413	Exhaust and Muffler Fitter	72312	p	Motor Mechanic
7991-11	p	Motor Vehicle Parts and Accessories Fitter	899414	Radiator Fitter	72312	p	Motor Mechanic
7991-11	p	Motor Vehicle Parts and Accessories Fitter	899415	Tyre Fitter	82313	p	Tyre Retreader
7996-13		Printer's Assistant	899511	Printer's Assistant	73317	p	Printing Machinist
7996-11		Printing Table Hand	899512	Printing Table Worker	73317	p	Printing Machinist
					73321	p	Bookbinder
9991-11		Garbage Collector	899611	Recycling or Rubbish Collector	91311	p	Refuse Collector
9999-11		Vending Machine Attendant	899711	Vending Machine Attendant	91514	p	General Labourer
9999-79	p	Labourers and Related Workers nec	899911	Bicycle Mechanic	72315	p	Small Engine Mechanic

ASCO Second Edition			ANZSCO		NZSCO 1999		
8319-17		Car Park Attendant	899912	Car Park Attendant	42113	p	Ticket-Seller
8319-19		Crossing Supervisor	899913	Crossing Supervisor	91514	p	General Labourer
9918-11		Electrical or Telecommunications Trades Assistant	899914	Electrical or Telecommunications Trades Assistant	91514	p	General Labourer
8319-23		Leaflet and Newspaper Deliverer	899915	Leaflet or Newspaper Deliverer	91211	p	Courier and Deliverer
9999-15		Mechanic's Assistant	899916	Mechanic's Assistant	91514	p	General Labourer
8319-11		Railways Assistant	899917	Railways Assistant	41331	p	Transport Clerk
					91412	p	Loader and/or Checker
					91514	p	General Labourer
9999-13		Sign Erector	899918	Sign Erector	91514	p	General Labourer
8312-11		Ticket Collector or Usher	899921	Ticket Collector or Usher	41331	p	Transport Clerk
					42113	p	Ticket-Seller
					51236	p	Usher and Cloakroom Attendant
8319-21		Trolley Collector	899922	Trolley Collector	52111	p	Sales Assistant
8319-79	p	Elementary Service Workers nec	899999	Labourers nec	91211	p	Courier and Deliverer
9999-79	p	Labourers and Related Workers nec			91514	p	General Labourer

The five skill levels in the Australian and New Zealand Standard Classification of Occupations (ANZSCO) are defined in terms of formal education and training, previous experience and on-the-job training.

In Australia, the formal education and training component is measured in terms of educational qualifications as set out in the Australian Qualifications Framework (the AQF). This appendix outlines the AQF in practise at the time of the development of ANZSCO.

The AQF is a single national and comprehensive system of qualifications in Australia. The AQF is endorsed by the Australian Government and therefore an AQF qualification is recognised all around Australia.

AQF qualifications link with each other, creating learning pathways between school, vocational education and training and university as education and career ambitions change or develop.

PURPOSES OF AQF

The AQF has a number of key purposes. They are:

- provide nationally consistent recognition of outcomes achieved in post-compulsory education
- help with developing flexible pathways which assist people to move more easily between education and training sectors and between those sectors and the labour market by providing the basis for recognition of prior learning including credit transfer and work and life experience
- integrate and streamline the requirements of participating providers, employers and employees, individuals and interested organisations
- offer flexibility to suit the diversity of purposes of education and training
- encourage individuals to progress through the levels of education and training by improving access to qualifications, clearly defining avenues for achievements, and generally contributing to lifelong learning
- encourage the provision of more and higher quality vocational education and training through qualifications that normally meet workforce requirements and vocational needs, thus contributing to national economic performance
- promote national and international recognition of qualifications offered in Australia.

AQF GUIDELINES

The AQF incorporates qualification titles and guidelines. The guidelines specify the characteristics of learning outcomes as well as explaining the responsibilities for assessment, issuance and certification. They are set out in a common format to enable comparisons and assist in distinguishing between qualifications. This common format covers the following areas:

- Purpose – aim of the guideline
- Context – why the guideline is necessary
- Learning Outcomes:
  - Authority – the ultimate determinants of the qualification
  - Characteristics – a description of learning outcomes or competencies to be achieved for this particular qualification
  - Distinguishing Features – the features that distinguish adjacent qualifications
- Responsibilities of Assessment – who is ultimately responsible for assessment
- Pathways to the Qualification – how the qualification can be achieved
- Authority to Issue the Qualification – who issues the qualification
- Certification Issued – when the qualification is issued and what is issued.

AQF QUALIFICATIONS

The qualifications are:

- Senior Secondary Certificate of Education
- Certificate I
- Certificate II
- Certificate III
- Certificate IV
- Diploma
- Advanced Diploma, Associate Degree
- Bachelor Degree
- Vocational Graduate Certificate
- Vocational Graduate Diploma
- Graduate Certificate
- Graduate Diploma
- Masters Degree
- Doctoral Degree

FURTHER INFORMATION

Guidelines for each qualification are provided in the *AQF Implementation Handbook* (2002) or by visiting the AQF web site <http:www.aqf.edu.au>.

APPENDIX C NEW ZEALAND REGISTER OF QUALITY ASSURED QUALIFICATIONS

The five skill levels in the Australian and New Zealand Standard Classification of Occupations (ANZSCO) are defined in terms of formal education and training, previous experience and on-the-job training.

In New Zealand, the formal education and training component is measured in terms of educational qualifications as set out in the New Zealand Register of Quality Assured Qualifications, Te Āhurutanga (the Register). This appendix outlines the Register in practise at the time of the development of ANZSCO.

The Register is a comprehensive list of all quality assured qualifications in New Zealand. Qualifications are quality assured by a recognised approval and accreditation body, and are automatically included on the Register. They can be viewed and compared on the KiwiQuals web site <http://www.kiwiquals.govt.nz>.

The Register is the result of extensive consultation and has the support of the New Zealand Vice-Chancellors Committee, the Association of Polytechnics in New Zealand and the Association of Colleges of Education in New Zealand.

PURPOSES OF THE REGISTER

The Register has a number of key purposes. They are to:

- identify clearly all quality assured qualifications in New Zealand
- ensure that all qualifications have a purpose and relation to each other that students and the public can understand
- maintain and enhance learners' ability to transfer credit by the establishment of a common system of credit
- enhance and build on the international recognition of New Zealand qualifications.

INFORMATION HELD ON THE REGISTER

The following information is held on the Register:

- title of the qualification
- level at which the qualification is registered
- outcome statement attached to the qualification
- credit requirements of the qualification
- subject classification
- qualification developer/provider details.

THE REGISTER'S QUALIFICATIONS

The Register has ten levels and comprises qualifications that are registered according to an agreed set of title definitions.

Level	Naming Sequence
10	Doctorates
9	Masters Degrees
8	Postgraduate Diplomas and Certificates,<br>Bachelors Degrees with Honours
7	Bachelors,<br>Graduate Diplomas
6	Graduate Certificates
5	Diplomas
4	Certificates
3	
2	
1	

In developing the skill specialisation criteria for the Australian and New Zealand Standard Classification of Occupations (ANZSCO), employability skills were considered as a possible additional dimension. Employers are increasingly using employability skills in conjunction with technical or job-specific skills when assessing the suitability of an individual for a particular occupation.

The employability skills considered for ANZSCO were those published by the Australian Government Department of Education, Science and Training in March 2002 in the report *Employability Skills for the Future*. This report discusses the employability skill needs of industry in Australia. It is based on research undertaken with industry.

The report identified two facets to employability skills: 'personal attributes' and 'generic skills'.

PERSONAL ATTRIBUTES

The 'personal attributes' component of employability skills includes the following qualities: loyalty, commitment, honesty and integrity, enthusiasm, reliability, personal presentation, commonsense, positive self-esteem, sense of humour, balanced attitude to work and home life, ability to deal with pressure, motivation and adaptability. As they relate to the individual rather than the occupation, they are not appropriate as classification criteria.

GENERIC SKILLS

There were eight 'generic skills' identified:

- communication skills that contribute to productive and harmonious relations between employees and customers
- team work skills that contribute to productive working relationships and outcomes
- problem-solving skills that contribute to productive (enterprise) outcomes
- initiative and enterprise skills that contribute to innovative outcomes
- planning and organisation skills that contribute to long-term and short-term strategic planning
- self-management skills that contribute to employee satisfaction and growth
- learning skills that contribute to ongoing improvement and expansion in employee and company operations and outcomes
- technology skills that contribute to the effective execution of tasks.

The generic skills have not been incorporated into ANZSCO because they are applicable to most occupations and therefore have limited value as classification criteria.

INDEX OF PRINCIPAL TITLES, ALTERNATIVE TITLES AND SPECIALISATIONS

This index lists occupation titles (principal titles, alternative titles and specialisations) used in the Australian and New Zealand Standard Classification of Occupations (ANZSCO), together with the relevant occupation (6-digit) code.

Alternative titles are denoted by an (A) and specialisations are denoted by an (S) following the index entry. Titles relating to occupations which are included in not elsewhere classified (nec) categories are denoted by (N) following the index entry.

This index is intended to help users find occupation definitions. As such, it is not suitable for use as the principal means of assigning ANZSCO codes to occupation information obtained from sources such as statistical collections, human resource management records or job vacancy advertisements.

The titles used in ANZSCO are intended to convey the clearest possible idea of the nature of the particular occupation. However, in some instances, the same job titles are used by different industries to describe different occupations (e.g. business analyst). The publication index is also not an exhaustive list of all titles used by people to describe an occupation (e.g. brickie).

Users wanting to assign ANZSCO codes to occupation information are advised to use the relevant computer coding systems developed by the Australian Bureau of Statistics and Statistics New Zealand.

For information about these coding systems, contact:

Australian Bureau of Statistics: Andrew Woolley on Canberra (02) 6252 7073

Statistics New Zealand: Andrew Hancock on Christchurch (03) 964 870

A

399911 Abalone Diver (S)
831313 Abalone Sheller (S)
149999 Abattoir Manager (N)
422111 Aboriginal and Torres Strait Islander Education Worker
411511 Aboriginal and Torres Strait Islander Health Worker
272211 Aboriginal Ceremonial Celebrant (Aus) (S)
111399 Aboriginal Community Council Member (Aus) (N)
241599 Aboriginal Education Teacher (Aus) (N)
422111 Aboriginal Education Worker Coordinator (S)
422111 Aboriginal Home-School Liaison Officer (S)
224912 Aboriginal Liaison Officer (S)
711199 Abrasive Wheel Maker (N)
452215 Abseiling Instructor (S)
141999 Accommodation and Hospitality Managers nec
221111 Accountant (General)
512299 Accounting Practice Manager (N)
551111 Accounts Clerk
551111 Accounts Payable or Receivable Clerk (A)
441111 Acoustic Warfare Analyst (Navy) (S)
224611 Acquisitions Librarian (S)
711513 Acrylic Fabricator (S)
411311 Activities Coordinator (S)
411311 Activities Officer (S)
211111 Actor
211199 Actors, Dancers and Other Entertainers nec
552314 Actuarial Clerk (S)
224111 Actuary
252211 Acupuncturist
132111 Administration Manager (A)
271213 Administrative Appeals Tribunal Member (S)
271299 Administrative Lawyer (N)
111212 Admiral (Navy) (S)
542112 Admissions Clerk
253411 Adolescent Psychiatrist (S)
242211 Adult Education Teacher (S)
452215 Adventure Challenge Instructor (S)
225111 Advertising Account Executive (A)
225111 Advertising Account Manager (A)
131111 Advertising and Public Relations Manager
225111 Advertising Specialist
312116 Aerial Survey Technician (S)
452111 Aerobics Instructor (S)
233911 Aeronautical Engineer
233911 Aeronautical Engineering Officer (Navy) (S)
233914 Aeronautical Engineering Technologist (S)
231111 Aeroplane Pilot
233911 Aerospace Engineer (S)
233911 Aerospace Engineer Officer - Aeronautical (Air Force) (S)
233911 Aerospace Engineer Officer - Armament (Air Force) (S)
233911 Aerospace Engineer Officer - Electronics (Air Force) (S)
423111 Aged or Disabled Carer
234111 Agricultural Adviser (A)
721111 Agricultural and Horticultural Mobile Plant Operator
234111 Agricultural Consultant
224311 Agricultural Economist (S)
233912 Agricultural Engineer
233914 Agricultural Engineering Technologist (S)
234111 Agricultural Extension Officer (S)
234112 Agricultural Scientist
311111 Agricultural Technical Officer (A)
311111 Agricultural Technician
311111 Agriculture Laboratory Technician (S)
234112 Agronomist (S)
111212 Air Chief Marshal (Air Force) (S)
111212 Air Commodore (Air Force) (S)
712999 Air Compressor Operator (N)
139111 Air Defence Officer (Air Force) (Aus) (S)
441111 Air Dispatcher (Army) (S)
111212 Air Force Senior Officer (A)
111212 Air Marshal (Air Force) (S)
231199 Air Observer (Rescue) (N)
234313 Air Pollution Analyst (S)
441111 Air Surveillance Operator (Air Force) (S)
231112 Air Traffic Controller
231199 Air Transport Professionals nec
111212 Air Vice Marshal (Air Force) (S)
399999 Airborne Electronics Analyst (Air Force) (N)
334112 Airconditioning and Mechanical Services Plumber
342111 Airconditioning and Refrigeration Mechanic
312511 Airconditioning Drafting Officer (S)
233512 Airconditioning Engineer (S)
712911 Airconditioning Plant Operator (S)
721911 Aircraft Baggage Handler and Airline Ground Crew
811211 Aircraft Cabin Cleaner (S)
312999 Aircraft Detail Draftsperson (N)
312412 Aircraft Electronics Technical Officer (S)
441111 Aircraft Life Support Fitter (Air Force) (S)
441111 Aircraft Life Support Fitter (Army) (S)
591211 Aircraft Load Controller (S)
323214 Aircraft Machinist (S)
323112 Aircraft Maintenance Engineer (Airframes) (S)
323111 Aircraft Maintenance Engineer (Avionics)
323111 Aircraft Maintenance Engineer (Electrical) (S)
323112 Aircraft Maintenance Engineer (Engines) (S)
323111 Aircraft Maintenance Engineer (Instruments) (S)
323112 Aircraft Maintenance Engineer (Mechanical)
323111 Aircraft Maintenance Engineer (Radio) (S)
323113 Aircraft Maintenance Engineer (Structures)
231199 Aircraft Navigator (N)
733112 Aircraft Refueller
323113 Aircraft Structural Fitter (Air Force, Army) (S)
312999 Aircraft Systems Technician (Air Force) (N)
323112 Aircraft Technician (Air Force, Army) (S)
441111 Aircrewman (Navy) (S)
441111 Airfield Defence Guard (Air Force) (S)
233211 Airfield Engineer Officer (Air Force) (S)
312999 Airframe Technical Officer (N)
139999 Airport Manager (N)
231199 Airworthiness Surveyor (N)
442211 Alarm, Security or Surveillance Monitor

111311 Alderman (Aus) (A)
121399 Alpaca Farmer (N)
712311 Alumina Refinery Operator (S)
139999 Ambassador (N)
411111 Ambulance Officer
411112 Ambulance Paramedic (NZ)
149111 Amusement Centre Manager
311211 Anaesthetic Technician
253211 Anaesthetist
261311 Analyst Programmer
234211 Analytical Chemist (S)
234512 Anatomist or Physiologist
361199 Animal Attendants and Trainers nec
234599 Animal Behaviourist (N)
361311 Animal Nurse (A)
232412 Animator (S)
322112 Anodiser (S)
272499 Anthropologist (N)
142112 Antique Dealer
394111 Antique Furniture Reproducer (S)
394111 Antique Furniture Restorer (S)
121311 Apiarist
393211 Apparel Cutter
261312 Applications Developer (A)
313199 Applications Packager (N)
452413 Apprentice Jockey (S)
234399 Aquaculture Consultant (N)
121111 Aquaculture Farmer
841111 Aquaculture Worker
361114 Aquarist (S)
149113 Aquatic Centre Manager (S)
252511 Aquatic Physiotherapist (S)
362212 Arborist
712311 Arc Welder (S)
331111 Arch Builder (S)
272499 Archaeologist (N)
139999 Archbishop (N)
232111 Architect
312111 Architectural Associate (A)
312111 Architectural Draftsperson
399999 Architectural Model Maker (N)
512299 Architectural Practice Manager (N)
224211 Archivist
341111 Armature Winder (S)
442212 Armoured Car Escort
139111 Armoured Corps Officer (Army) (S)
111212 Army Senior Officer (A)
451511 Aromatherapist (S)
234911 Art Conservator (S)
212311 Art Director (Film, Television or Stage)
139911 Art Gallery Director (S)
272411 Art Historian (S)
272314 Art Psychotherapist or Therapist (S)
249211 Art Teacher (Private Tuition)
311111 Artificial Insemination Technical Officer (S)
139111 Artillery Officer (Army) (S)
212111 Artistic Director
139911 Arts Administrator or Manager
711999 Asbestos Remover (N)
224511 Asset Manager (Land and Property) (S)
111211 Assistant Commissioner (Police) (S)
134212 Assistant Director of Nursing (S)
423312 Assistant in Nursing (A)
212412 Associate Editor (A)
251911 Asthma Educator (S)
451899 Astrologer (N)
234914 Astronomer (S)
452499 Athlete (N)
611111 Auctioneer
399516 Audio Operator (S)
252711 Audiologist
311299 Audiometrist (N)
224611 Audiovisual Librarian (S)
551111 Audit Clerk (S)
221214 Audit Officer (A)
452411 Australian Rules Footballer (S)
212211 Author
899412 Autoglazier
394213 Automatic Profile Sander Operator (S)
321211 Automatic Transmission Mechanic (S)
233513 Automation and Control Engineer (S)
731199 Automobile Drivers nec
321211 Automotive Airconditioning Mechanic (S)
621911 Automotive Dismantler (S)
321111 Automotive Electrical Fitter (A)
321111 Automotive Electrician
323214 Automotive Machinist (S)
621312 Automotive Parts Interpreter (A)
621312 Automotive Parts Salesperson (NZ)
323112 Aviation Technician (Navy) (S)
233911 Avionics Systems Engineer (S)
312999 Avionics Systems Technician (Air Force) (N)
323111 Avionics Technician (Defence) (S)
821412 Awning Installer (S)

B

261111 BA (ICT) (A)
721212 Backhoe Operator
141999 Backpackers Manager (N)
234517 Bacteriologist (Non-medical) (S)
561112 Bagman/woman (Aus) (S)
351111 Baker
851211 Bakery Assistant (S)
831111 Baking Factory Worker
211112 Ballet Dancer (S)
249212 Ballet Teacher (Private Tuition) (S)
231199 Balloonist (N)
211212 Band Leader (S)
711312 Band Saw Operator (S)
211214 Band Singer (S)
149914 Bank Manager (S)
552111 Bank Worker
431111 Bar Attendant
431911 Bar Back (A)

431111 Bar Steward (A)
431911 Bar Useful or Busser
391111 Barber (S)
899211 Barge Hand (S)
431112 Barista
271111 Barrister
452317 Basketball Coach (S)
711312 Beam Saw Operator (S)
711715 Beamer (S)
451111 Beauty Therapist
141911 Bed and Breakfast Operator
121312 Beef Cattle Farm Manager (A)
841511 Beef Cattle Farm Worker
121312 Beef Cattle Farmer
121312 Beef Cattle Grazier (A)
121311 Beekeeper (A)
241511 Behaviour Support Teacher (S)
561111 Betting Agency Counter Clerk
142113 Betting Agency Manager
561199 Betting Clerks nec
712999 Beverage Distiller (N)
224611 Bibliographer (S)
561211 Bicycle Courier (S)
899911 Bicycle Mechanic
899911 Bicycle Repairer (A)
899911 Bicycle Technician (A)
392111 Binder and Finisher
561199 Bingo Caller (N)
234513 Biochemist
233913 Bioengineer (S)
311413 Biological Technical Officer (S)
234511 Biologist (General) (A)
233913 Biomedical Engineer
312999 Biomedical Engineering Associate (N)
233914 Biomedical Engineering Technologist (S)
224113 Biometrician (S)
311313 Biosecurity Officer (Ministry of Agriculture and Forestry) (NZ) (A)
599514 Biosecurity Officer (Weeds and Pests) (A)
234514 Biotechnologist
831111 Biscuit Factory Worker (S)
139999 Bishop (N)
141111 Bistro Manager (S)
712999 Bitumen Plant Operator (N)
322111 Blacksmith
712916 Bleach Plant Operator (S)
331111 Blocklayer (A)
212499 Blogger (N)
451899 Blood Bank Aide (N)
711514 Blow Moulding Machine Operator (S)
141999 Boarding House Manager (N)
149911 Boarding Kennel or Cattery Operator
399111 Boat Builder and Repairer
441111 Boatswains Mate (Navy) (S)
721216 Bobcat Operator (S)
612112 Body Corporate Manager (S)
442299 Bodyguard (N)
712212 Bogger Operator (S)
712911 Boiler or Engine Operator
312512 Boiler Testing Technician (S)
839111 Boilermaker's Assistant (S)
322311 Boilermaker-Welder (S)
312611 Boilers and Pressure Vessels Inspector (S)
441312 Bomb Squad Officer (S)
591212 Bond Clerk (S)
212212 Book or Script Editor
639411 Booking Clerk (S)
551211 Bookkeeper
552311 Bookmaker
561112 Bookmaker's Clerk
599521 Boring Inspector (S)
311413 Botanical Technical Officer (S)
234515 Botanist
621911 Bottle Dealer (S)
832112 Bottling Attendant (S)
442213 Bouncer (A)
899999 Bowling Alley Attendant (N)
712111 Braceperson (S)
532112 Braille Transcriber (S)
711199 Brake Lining Maker (N)
321211 Brake Mechanic (S)
712311 Brake Press Operator (S)
225113 Brand Manager (S)
322311 Brass Finisher (S)
831111 Bread Room Hand (S)
831112 Brewery Worker
711111 Brick Extruder Operator (S)
839913 Brick Handler (S)
331111 Bricklayer
821111 Bricklayer's Assistant (S)
149111 Bridge Club Manager (S)
111212 Brigadier (Army) (S)
399511 Broadcast Transmitter Operator
149999 Brothel Keeper (N)
711999 Brush Maker (N)
821111 Builder's Labourer
133111 Building and Construction Manager (A)
312999 Building and Engineering Technicians nec
312112 Building Associate
312113 Building Certifier (A)
312112 Building Construction Supervisor (S)
312111 Building Drafting Officer (S)
233213 Building Economist (A)
312114 Building Estimator (A)
312113 Building Inspector
821411 Building Insulation Installer
149913 Building Manager (A)
149914 Building Society Manager (S)
312113 Building Surveyor (A)
712912 Bulk Fluids Handler (S)
712912 Bulk Materials Handling Plant Operator
721213 Bulldozer Operator
711911 Bullet Maker (S)
452211 Bungy Jump Master

149413 Bus Company Manager (S)
731211 Bus Driver
451899 Bus Escort (N)
599518 Bus Inspector (S)
452216 Bushwalking Guide (A)
612111 Business Agent (A)
224711 Business Analyst (S)
612111 Business Broker
224711 Business Consultant (A)
261111 Business Consultant (ICT) (A)
131112 Business Development Manager (S)
224912 Business Liaison Officer (S)
342311 Business Machine Mechanic
132111 Business Services Manager (A)
261111 Business Systems Analyst (S)
451612 Business Travel Consultant (S)
211113 Busker (S)
351211 Butcher or Smallgoods Maker
851299 Butcher's Assistant (N)
451899 Butler (N)
831114 Butter Maker (S)

C

451799 Cabin Steward (N)
451711 Cabin Supervisor (Aircraft) (S)
394111 Cabinetmaker
721999 Cable Ferry Operator (N)
342411 Cabler (Data and Telecommunications)
232212 Cadastral Surveyor (S)
431211 Cafe Assistant (A)
431211 Cafe Attendant (A)
141111 Cafe or Restaurant Manager
431211 Cafe Worker
351112 Cake Decorator (S)
541111 Call Centre Supervisor (A)
541111 Call or Contact Centre Coach (S)
149211 Call or Contact Centre Manager
541112 Call or Contact Centre Operator
541111 Call or Contact Centre Team Leader
541111 Call or Contact Centre Workforce Planner (S)
452312 Callisthenics Instructor (S)
399512 Camera Operator (Film, Television or Video)
323314 Camera Repairer (S)
711911 Candle Maker (S)
394299 Cane Furniture Maker (N)
832112 Cannery Worker (S)
399999 Canoe Maker (N)
711312 Cant Gang Sawyer (S)
431211 Canteen Attendant (S)
141111 Canteen Manager (S)
393111 Canvas Goods Maker
111212 Captain (Navy) (S)
712999 Car Compactor Operator (N)
811111 Car Detailer
899912 Car Park Attendant
149413 Car Rental Agency Manager (S)
621912 Car Rental Sales Assistant (S)
141211 Caravan Park and Camping Ground Manager
311212 Cardiac Technician
311212 Cardiac Technologist (S)
253312 Cardiologist
252511 Cardiothoracic Physiotherapist (S)
253512 Cardiothoracic Surgeon
272111 Careers Adviser (A)
272111 Careers Counsellor
899111 Caretaker
839913 Carousel Minder (S)
331212 Carpenter
331211 Carpenter and Joiner
821111 Carpenter's Assistant (S)
811611 Carpet Cleaner
332111 Carpet Layer (S)
621999 Carpet Measurer (N)
711715 Carpet Weaver (S)
821412 Carport Erector (S)
232211 Cartographer
711311 Carton Making Machinist (S)
232412 Cartoonist (S)
224213 Casemix Coordinator (S)
621711 Cash Van Salesperson
631112 Cashier (A)
141999 Casino Duty Manager (N)
431311 Casino Gaming Inspector (S)
223112 Casting Agent (S)
212399 Casting Director (N)
224611 Cataloguer (S)
225113 Category Manager (S)
141111 Caterer (S)
121411 Cattle and Wheat Farmer (S)
452299 Caving Guide (N)
234514 Cell Geneticist (S)
839211 Cellular Plastics Cutter (S)
839911 Cement and Concrete Plant Worker
712913 Cement Crusher Operator (S)
712913 Cement Despatch Operator (S)
712913 Cement Grinding Mill Operator (S)
712913 Cement Kiln Operator (S)
733111 Cement Mixer Driver (S)
712913 Cement Production Plant Operator
232312 Ceramic Designer (S)
333411 Ceramic Tiler (S)
234999 Ceramics Scientist (N)
254111 Certified Midwife (A)
321214 Chainsaw Mechanic (S)
394111 Chair and Couch Maker (S)
712111 Chairlift Operator (S)
271212 Chamber Magistrate (A)
224712 Change Management Facilitator (S)
451399 Chapel or Memorial Attendant (N)
272211 Chaplain (S)
254311 Charge Nurse (A)
731212 Charter and Tour Bus Driver
731111 Chauffeur
631111 Checkout Operator

621511 Checkout Supervisor (A)
831114 Cheese Factory Worker (S)
831114 Cheese Maker (S)
832199 Cheese Packer (N)
351311 Chef
351311 Chef de Partie (S)
851299 Chef's Assistant (N)
233111 Chemical Engineer
233914 Chemical Engineering Technologist (S)
311411 Chemical Instrumentation Officer (S)
399211 Chemical Plant Operator
839912 Chemical Plant Worker
311411 Chemical Process Analyst (S)
711911 Chemical Production Machine Operator
399211 Chemicals Distiller (S)
399211 Chemicals Fermentation Operator (S)
234211 Chemist
311411 Chemistry Laboratory Technician (S)
311411 Chemistry Technical Officer (A)
311411 Chemistry Technician
712111 Cherry Picker Operator (S)
121321 Chicken Meat Producer (S)
111111 Chief Executive Officer (A)
111111 Chief Executive or Managing Director
132211 Chief Financial Officer (A)
135111 Chief Information Officer
111312 Chief Minister (Aus) (S)
111211 Chief Operating Officer (A)
135111 Chief Technology Officer (A)
253411 Child and Adolescent Psychiatrist (S)
421111 Child Care Aide (A)
134111 Child Care Centre Director (A)
134111 Child Care Centre Manager
134111 Child Care Coordinator (A)
421111 Child Care Worker
423411 Child or Youth Residential Care Assistant
253411 Child Psychiatrist (S)
251911 Childbirth Educator (S)
271212 Children's Court Magistrate (S)
224611 Children's Librarian (S)
421111 Children's Nursery Assistant (S)
741111 Chiller Hand (S)
331111 Chimney Builder (S)
811699 Chimney Sweep (N)
411611 Chinese (Tui-Na) Masseur (S)
252214 Chinese Herbalist (S)
252214 Chinese Medicine Practitioner (A)
512211 Chiropractic Practice Manager (S)
252111 Chiropractor
831113 Chocolate Maker (S)
832111 Chocolate Packer
211212 Choral Director (S)
211214 Chorister (S)
149912 Cinema or Theatre Manager
212313 Cinematographer (A)
211113 Circus Artist (S)
451811 Civil Celebrant
233211 Civil Engineer
312212 Civil Engineering Assistant (S)
312211 Civil Engineering Design Draftsperson (S)
312211 Civil Engineering Draftsperson
312212 Civil Engineering Technician
312212 Civil Laboratory Technician (S)
561911 Classified Advertising Clerk
839913 Clay Processing Factory Worker
839913 Clay Processing Labourer (A)
711111 Clay Products Machine Operator
711199 Clay, Concrete, Glass and Stone Processing Machine Operators nec
811699 Cleaners nec
399911 Clearance Diver (Navy) (S)
599999 Clerical and Administrative Workers nec
561999 Clerical and Office Support Workers nec
599211 Clerk of Court
312112 Clerk of Works (S)
149212 Client Service Manager (A)
234913 Climatologist (S)
452214 Climbing Guide (S)
253399 Clinical Allergist (N)
599911 Clinical Coder (S)
253915 Clinical Cytopathologist (S)
411212 Clinical Dental Technician (A)
233913 Clinical Engineer (S)
253399 Clinical Geneticist (N)
253313 Clinical Haematologist
253399 Clinical Immunologist (N)
254211 Clinical Nurse Educator (A)
253314 Clinical Oncologist
253399 Clinical Pharmacologist (N)
272311 Clinical Psychologist
224213 Clinical Trial Data Manager (S)
431999 Cloakroom Attendant (N)
393212 Clothing Patternmaker
621111 Clothing Sales Assistant (S)
393299 Clothing Trades Workers nec
211113 Clown (S)
141411 Club Licensee (A)
324211 Coach Builder (S)
731212 Coach Tour Driver (S)
712212 Coal Cutter (S)
839313 Coal Sample Tester (S)
599911 Coding Clerk
121299 Coffee Grower (Aus) (N)
841299 Coffee Plantation Worker (Aus) (N)
394111 Coffin Maker (S)
599311 Collection Agent (S)
599311 Collection Officer (S)
111212 Colonel (Army) (S)
212413 Columnist (S)
441111 Combat Engineer (Army) (S)
441111 Combat Systems Operator (Navy) (S)
441111 Combat Systems Operator Mine Warfare (Navy) (S)
211113 Comedian (S)
111212 Commander (Navy) (S)

441111 Commando (Army) (S)
212114 Commentator (Television) (A)
811211 Commercial Cleaner
811411 Commercial Housekeeper
232511 Commercial Interior Designer (S)
211214 Commercial Singer (Advertising) (S)
351311 Commis Chef (S)
139111 Commissioned Defence Force Officer
139112 Commissioned Fire Officer
139113 Commissioned Police Officer
222111 Commodities Broker (A)
222111 Commodities Trader
111212 Commodore (Navy) (S)
342314 Communication Electronic Technician (Air Force) (S)
342312 Communication Information Systems Sailor (Navy) (S)
312411 Communications and Data Systems Drafting Officer (S)
342312 Communications and Information Systems Controller (Air Force) (S)
263312 Communications Consultant (A)
599914 Communications Controller (A)
233411 Communications Engineer (Army) (S)
312412 Communications Engineering Technical Officer (S)
312412 Communications Engineering Technician (S)
342312 Communications Operator
261312 Communications Programmer (Systems) (S)
263312 Communications Specialist (ICT) (A)
342414 Communications Technician (A)
272611 Community Artist (A)
139911 Community Arts Centre Manager (S)
272611 Community Arts Officer (A)
272611 Community Arts Worker
111311 Community Board Member (NZ) (S)
272611 Community Cultural Development Officer (S)
411711 Community Development Officer (S)
251911 Community Health Worker (A)
224912 Community Liaison Officer (S)
254111 Community Midwife (S)
251513 Community Pharmacist (A)
272399 Community Psychologist (N)
131111 Community Relations Manager (S)
411711 Community Support Worker (S)
411711 Community Worker
733111 Compactor Driver (Rubbish Collection) (S)
221211 Company Secretary
212114 Compere (Television) (A)
252299 Complementary Health Therapists nec
211211 Composer
399111 Composite Boat Builder (S)
263111 Computer Network and Systems Engineer
263111 Computer Network Engineer (S)
712311 Computer Numeric Control Machine Operator (S)
323211 Computer Numeric Control Setter (S)
312412 Computer Numeric Control Technical Officer (S)
263211 Computer Systems Auditor (S)
263111 Computer Systems Integrator (S)
313199 Computer Systems Technician (N)
712914 Concrete Batching Plant Operator
712915 Concrete Boom Operator (S)
711112 Concrete Pipe Machine Operator (S)
711112 Concrete Precast Moulder (S)
711112 Concrete Products Machine Operator
712915 Concrete Pump Operator
711112 Concrete Tile Machine Operator (S)
821211 Concrete Worker (A)
821211 Concreter
831113 Confectionery Maker
149311 Conference and Event Organiser
234311 Conservation Officer
232111 Conservation or Heritage Architect (S)
234911 Conservator
233213 Construction Economist (A)
312114 Construction Estimator
721211 Construction Plant Operator (General) (A)
133111 Construction Project Manager
821711 Construction Rigger
331112 Construction Stonemason (S)
821111 Construction Worker (A)
541111 Contact Centre Supervisor (A)
832112 Container Filler
211112 Contemporary or Modern Dancer (S)
252511 Continence and Women's Health Physiotherapist (S)
399599 Continuity Person (N)
511111 Contract Administrator
511111 Contract Officer (A)
599914 Control Room Operator (A)
312411 Control Systems Drafting Officer (S)
599111 Conveyancer
712912 Conveyor Belt Operator (S)
351411 Cook
851299 Cook's Assistant (N)
394299 Cooper (N)
711411 Copy Camera Operator (S)
394213 Copy Lathe Operator (S)
212411 Copywriter
841999 Coral Collector (N)
711799 Cord Making Machine Operator (N)
322114 Coremaker (S)
225311 Corporate Affairs Officer (A)
111211 Corporate General Manager
224611 Corporate Librarian (S)
132411 Corporate Planning Manager (S)
131111 Corporate Relations Manager (S)
132111 Corporate Services Manager
221212 Corporate Treasurer
241599 Correspondence School Teacher (NZ) (N)
621111 Cosmetic Sales Assistant (S)
711911 Cosmetics Machine Operator (S)
221112 Cost Accountant (A)
551112 Cost Clerk
232311 Costume Designer (S)
393213 Costume Maker (S)
711716 Cotton Ginner (S)
121211 Cotton Grower
721111 Cotton Picking Machine Operator (Aus) (S)

111311 Councillor (A)
111399 Councillor, Aboriginal Land Council (Aus) (N)
272399 Counselling Psychologist (N)
272199 Counsellors nec
561211 Courier
599213 Court Attendant (A)
599212 Court Bailiff or Sheriff (Aus)
599212 Court Collections Officer (NZ)
599213 Court Officer (A)
599213 Court Orderly (Aus)
599213 Court Registry Officer (NZ)
532112 Court Reporter (S)
599213 Court Usher (S)
821911 Crane Chaser
712111 Crane, Hoist or Lift Operator
899212 Cray Fishing Hand (S)
225111 Creative Director (Advertising) (A)
421111 Crèche Attendant (S)
552211 Credit Clerk (A)
552211 Credit or Loans Officer
149914 Credit Union Manager (S)
712999 Crematorium Operator (N)
451711 Crew Attendant (Air Force) (S)
441111 Crewman Armoured Personnel Carrier M113 (Army) (S)
441111 Crewman Australian Light Armoured Vehicle (ASLAV) (Army) (S)
441111 Crewman Main Battle Tank (Army) (S)
452317 Cricket Coach (S)
452499 Cricketer (N)
224411 Criminal Intelligence Analyst (S)
272499 Criminologist (N)
212499 Critic (N)
121399 Crocodile Farmer (Aus) (N)
841299 Crop Farm Workers nec
121299 Crop Farmers nec
899913 Crossing Supervisor
431311 Croupier (A)
442213 Crowd Controller
361199 Crutching Contractor (N)
139911 Cultural Centre Manager (S)
272411 Cultural Historian (S)
249111 Curriculum Advisory Teacher (S)
821412 Curtain Fitter (S)
442111 Custodial Officer (A)
591111 Customer Orders Clerk (A)
149212 Customer Service Manager
591212 Customs Agent (S)
591212 Customs Broker (A)
599511 Customs Inspector (A)
599511 Customs Investigator (S)
599511 Customs Officer
452299 Cycle Touring Guide (N)
452499 Cyclist (N)

D

121313 Dairy Cattle Farm Manager (A)
841512 Dairy Cattle Farm Worker
121313 Dairy Cattle Farmer
311411 Dairy Laboratory Technician (S)
831114 Dairy Products Maker
311399 Dairy Quality Assurance Officer (N)
311111 Dairy Technician (S)
249212 Dance Teacher (Private Tuition)
252299 Dance Therapist (N)
211112 Dancer or Choreographer
711411 Dark Room Attendant (S)
532111 Data Entry Operator
532111 Data Processing Operator (A)
262111 Database Administrator
262111 Database Analyst (S)
261313 Database Designer (S)
261312 Database Developer (S)
262111 Database Operator (A)
261312 Database Programmer (Systems) (S)
262111 Database Specialist (A)
262111 Database Support (A)
262111 DBA (A)
134411 Dean (University) (S)
711312 Debarker Operator (S)
599311 Debt Collector
599311 Debt Recovery Officer (A)
899211 Deck Hand
231214 Deck Officer (A)
841599 Deer Farm Worker (N)
121314 Deer Farmer
441111 Defence Force Member - Other Ranks
111212 Defence Force Senior Officer
224411 Defence Intelligence Analyst (S)
591112 Delivery Clerk (S)
732111 Delivery Driver
351311 Demi Chef (S)
224113 Demographer (S)
423211 Dental Assistant
423211 Dental Chairside Assistant (A)
411211 Dental Hygienist
423211 Dental Nurse (A)
512211 Dental Practice Manager (S)
252312 Dental Practitioner (A)
411212 Dental Prosthetist
252311 Dental Specialist
252312 Dental Surgeon (A)
411213 Dental Technician
411214 Dental Therapist
252312 Dentist
134212 Deputy Director of Nursing (S)
222211 Derivatives Dealer (S)
253911 Dermatologist
821912 Derrick Hand (S)
392211 Desktop Publishing Operator (S)
591211 Despatch Clerk (A)
591211 Despatching and Receiving Clerk
441311 Detective
441311 Detective Sergeant (S)
261312 Developer Programmer

712211 Development Driller (S)
251911 Diabetes Educator (S)
253315 Diabetologist (S)
253916 Diagnostic Radiologist (S)
311299 Dialysis Technician (N)
399411 Diamond Cutter (S)
323412 Die Caster (S)
323412 Die Sinker (S)
323211 Diesel Fitter-Mechanic (S)
321212 Diesel Motor Mechanic
251111 Dietitian
312412 Digital Controls Technical Officer (S)
232413 Digital Media Designer (A)
711411 Digital Photographic Printer (S)
392312 Digital Printer (S)
712211 Directional Driller (S)
212312 Director (Film, Television, Radio or Stage)
134211 Director of Clinical Services (S)
134211 Director of Medical Services (S)
134212 Director of Nursing (A)
134299 Director of Pharmacy (N)
212313 Director of Photography
134299 Director of Physiotherapy Services (N)
134299 Director of Speech Pathology (N)
111111 Director-General (S)
411712 Disabilities Services Officer
224912 Disability Liaison Officer (S)
211199 Disc Jockey (Nightclub) (N)
212113 Disc Jockey (Radio) (S)
851311 Dishwasher (S)
311215 Dispensary Technician (A)
399913 Dispensing Optician (NZ)
241599 Distance Education Teacher (Aus) (N)
271211 District Court Judge (S)
452311 Dive Master (S)
399911 Diver
411311 Diversional Therapist
423314 Diversional Therapist's Assistant (S)
452315 Diving Coach (S)
452311 Diving Instructor (Open Water)
711312 Docking Saw Operator (S)
599599 Dog Catcher (N)
361111 Dog Handler or Trainer
452318 Dog or Horse Racing Official
451899 Dog Walker (N)
821911 Dogman/woman (S)
811311 Domestic Cleaner
811412 Domestic Housekeeper
451612 Domestic Travel Consultant (S)
451813 Dominatrix (S)
431912 Doorperson or Luggage Porter
621712 Door-to-door Fundraising Collector (S)
621712 Door-to-door Salesperson
351111 Doughmaker (S)
712212 Dragline Operator (S)
312115 Drainage Inspector (S)
821112 Drainage, Sewerage and Stormwater Labourer
334113 Drainer (Aus)
334113 Drainlayer (NZ)
249213 Drama Teacher (Private Tuition)
252299 Drama Therapist (N)
212212 Dramaturge (S)
231213 Dredge Master (S)
721999 Dredge Operator (N)
452313 Dressage Instructor (S)
393213 Dressmaker or Tailor
249299 Dressmaking Teacher (Private Tuition) (N)
712211 Driller
821912 Driller's Assistant
821912 Driller's Offsider (A)
712211 Drilling Plant Operator (A)
431511 Drink Waiter (S)
621611 Driveway Attendant (A)
451211 Driving Instructor
272112 Drug and Alcohol Counsellor
211213 Drummer (S)
333211 Dry Wall Plasterer (S)
811512 Drycleaner
712916 Dryerman/woman (Paper Mill) (S)
399516 Dubbing Machine Operator (S)
841299 Duboisia Farm Worker (Aus) (N)
121299 Duboisia Farmer (Aus) (N)
334112 Ductfixing Plumber (S)
141311 Duty Manager (Hotel) (S)
312912 Dye Penetrant Testing Technician (S)

E

253515 Ear, Nose and Throat Specialist (A)
241111 Early Childhood (Pre-primary School) Teacher
311412 Earth Science Laboratory Technician (S)
311412 Earth Science Technical Officer (A)
311412 Earth Science Technician
821113 Earthmoving Labourer
721211 Earthmoving Plant Operator (General)
311413 Ecological Technical Officer (S)
234313 Ecologist (S)
224311 Econometrician (S)
224311 Economic Analyst (A)
224311 Economic Forecaster (S)
272411 Economic Historian (S)
224311 Economist
394213 Edge Bander Operator (S)
212499 Editorial Assistant (N)
249111 Education Adviser
134499 Education Managers nec
223311 Education Officer (Air Force and Army) (S)
249111 Education Officer (S)
249112 Education Reviewer
272312 Educational Psychologist
831199 Egg Factory Worker (N)
832199 Egg Packer (N)
121321 Egg Producer (S)
224999 Electoral Officer (N)
224911 Electorate Officer

731311 Electric Train Driver (S)
832211 Electrical and Electronic Assembler (S)
341111 Electrical Contractor (S)
233311 Electrical Design Engineer (S)
233311 Electrical Engineer
312311 Electrical Engineering Design Draftsperson (S)
312312 Electrical Engineering Detail Draftsperson (S)
312311 Electrical Engineering Drafting Officer (S)
312311 Electrical Engineering Draftsperson
312312 Electrical Engineering Laboratory Technician (S)
312312 Electrical Engineering Technical Officer (A)
312312 Electrical Engineering Technician
341111 Electrical Fitter (A)
312113 Electrical Installation Inspector (S)
312312 Electrical Instrument Technician (S)
342211 Electrical Line Mechanic (Distribution) (NZ) (S)
342211 Electrical Line Mechanic (NZ)
342211 Electrical Line Mechanic (Transmission) (NZ) (S)
342211 Electrical Linesworker (Aus)
342211 Electrical Linesworker (Distribution) (Aus) (S)
342211 Electrical Linesworker (Transmission) (Aus) (S)
899914 Electrical or Telecommunications Trades Assistant
341111 Electrician (General)
341112 Electrician (Special Class)
311212 Electrocardiographic Technician (S)
311299 Electroencephalographic Technician (N)
322112 Electroformer (S)
451111 Electrologist (Hair Remover) (S)
312411 Electronic Engineering Draftsperson
312412 Electronic Engineering Technician
342313 Electronic Equipment Trades Worker
342314 Electronic Instrument Trades Worker (General)
342315 Electronic Instrument Trades Worker (Special Class)
342314 Electronic Technician (Navy) (S)
441111 Electronic Warfare - Linguist (Navy) (S)
441111 Electronic Warfare - Technical (Navy) (S)
312411 Electronics Detail Draftsperson (S)
233411 Electronics Engineer
322112 Electroplater
712111 Elevated Work Platform Operator (S)
249213 Elocution Teacher (S)
451399 Embalmer (N)
711311 Embosser (S)
711611 Embroiderer (S)
234512 Embryologist (S)
253912 Emergency Medicine Specialist
253912 Emergency Physician (A)
441211 Emergency Response Officer (A)
441211 Emergency Service Worker
223112 Employment Consultant (Aus) (A)
599411 Employment Office Clerk (A)
841599 Emu Farm Worker (Aus) (N)
121399 Emu Farmer (Aus) (N)
253315 Endocrinologist
252311 Endodontist (S)
222111 Energy Trader (S)
133211 Engineering Manager
323411 Engineering Patternmaker
712311 Engineering Production Systems Worker
233999 Engineering Professionals nec
591114 Engineering Sales Clerk (S)
312116 Engineering Survey Drafting Technician (S)
233914 Engineering Technologist
249311 English as a Second Language Teacher (A)
323311 Engraver
411411 Enrolled Nurse
211113 Entertainer or Variety Artist
149111 Entertainment Centre Manager (A)
899921 Entertainment Usher (S)
234518 Entomologist (S)
234312 Environmental Adviser (A)
234312 Environmental Analyst (A)
234399 Environmental Auditor (N)
234312 Environmental Consultant
232511 Environmental Designer (S)
224311 Environmental Economist (S)
233915 Environmental Engineer
251311 Environmental Health Officer
139912 Environmental Manager
234311 Environmental Officer (A)
234313 Environmental Research Scientist
234313 Environmental Scientist (A)
234399 Environmental Scientists nec
311413 Environmental Technical Officer (S)
234513 Enzyme Chemist (S)
211499 Ephemeral Artist (N)
224113 Epidemiologist (S)
149999 Equipment Hire Manager (N)
222299 Equities Analyst (N)
212499 Essayist (N)
272499 Ethnographer (N)
211299 Ethnomusicologist (N)
149311 Event Management Consultant (A)
149311 Event Planner (S)
599999 Examination Supervisor (N)
721214 Excavator Operator
111111 Executive Director (S)
134212 Executive Director of Nursing (S)
212112 Executive Producer (S)
234999 Exercise Physiologist (N)
899413 Exhaust and Muffler Fitter
232411 Exhibition Designer (S)
149311 Exhibition Organiser (S)
211112 Exotic Dancer (S)
712211 Exploration Driller (S)
711911 Explosives Mixer Operator (S)
221213 External Auditor
711514 Extruding Machine Operator (Plastics) (S)
253914 Eye Specialist (A)
253914 Eye Surgeon (A)

F
839914 Fabric and Textile Factory Worker
399411 Faceter (S)

149913 Facilities Manager
839999 Factory Process Workers nec
134411 Faculty Head
149111 Fairground Operator (S)
272113 Family and Marriage Counsellor
272113 Family Court Counsellor (S)
271211 Family Court Justice (S)
271299 Family Court Registrar (Aus) (N)
421112 Family Day Care Worker
421112 Family Day Carer (A)
272113 Family Mediator (S)
411713 Family Support Worker
841999 Farm, Forestry and Garden Workers nec
121411 Farmer and Grazier (A)
322113 Farrier
232311 Fashion Designer
211311 Fashion Photographer (S)
851111 Fast Food Cook
732111 Fast Food Delivery Driver (S)
621111 Fast Food Sales Assistant (S)
212413 Feature Writer (S)
212412 Features Editor (S)
711712 Fellmongering Machine Operator (S)
839917 Fellmongery Worker (S)
711799 Feltmaker (N)
821311 Fence Erector (A)
821311 Fencer
899211 Ferry Hand (S)
711199 Fibre Cement Moulder (N)
342412 Fibre Optic Cable Splicer (S)
342412 Fibre Optics Jointer (S)
311499 Fibre Technologist (N)
711515 Fibreglass Gun Hand (S)
711515 Fibreglass Hand Laminator (S)
839211 Fibreglass Lay Up Worker (S)
333211 Fibrous Plasterer
841213 Field Crop Farm Worker (NZ)
121214 Field Crop Grower (NZ)
311111 Field Crop Technical Officer (S)
561311 Filing or Registry Clerk
212314 Film and Video Editor
232411 Film and Video Graphics Designer (S)
224211 Film Archivist (S)
711999 Film Cutter (N)
839311 Film Examiner (S)
711411 Film Process Operator (S)
212112 Film Producer (S)
711999 Film Spooler (N)
212399 Film, Television, Radio and Stage Directors nec
222112 Finance Broker
552211 Finance Clerk (A)
132211 Finance Director (A)
132211 Finance Manager
221112 Financial Accountant (A)
551211 Financial Administration Officer (S)
221111 Financial Analyst (S)
222199 Financial Brokers nec
132211 Financial Controller (A)
222299 Financial Dealers nec
149914 Financial Institution Branch Manager
222311 Financial Investment Adviser
222312 Financial Investment Manager
222211 Financial Market Dealer
222311 Financial Planning Adviser (A)
221212 Financial Risk Manager (A)
399999 Fire Alarm Technician (N)
441212 Fire Engineer (Army) (S)
399999 Fire Extinguisher Technician (N)
441212 Fire Fighter
139112 Fire Investigator (S)
841311 Fire Lookout (S)
441212 Fire Prevention Officer (S)
334111 Fire Services Plumber (S)
731311 Fireperson (Railway) (S)
451899 First Aid Attendant (N)
121111 Fish Farmer (S)
399911 Fisheries Diver (S)
311311 Fisheries Inspector (A)
311311 Fisheries Officer
311413 Fisheries Technical Officer (S)
899212 Fishing Boat Mate (A)
452212 Fishing Guide
899212 Fishing Hand
149112 Fitness Centre Manager
452111 Fitness Instructor
323211 Fitter (General)
323212 Fitter and Turner
323212 Fitter Armament (Army) (S)
323211 Fitter-Machinist (S)
323211 Fitter-Mechanic (S)
839111 Fitter's Assistant (S)
323213 Fitter-Welder
222211 Fixed Interest Dealer (S)
331212 Fixing Carpenter (S)
711713 Flat Bed Knitter (S)
149411 Fleet Manager
621311 Fleet Salesperson (S)
392311 Flexographic Printing Machinist (S)
451711 Flight Attendant
231199 Flight Engineer Inspector (N)
312412 Flight Surveyor (S)
332111 Floor Finisher
121212 Floriculturist (A)
362111 Florist
841299 Flower Buncher or Picker (N)
121212 Flower Grower
452212 Fly Fishing Guide (S)
231113 Flying Instructor
399512 Focus Puller (Film) (S)
811511 Folding Machine Operator (S)
399516 Foley Artist (S)
431511 Food and Beverage Attendant (A)
141111 Food and Beverage Manager (A)
831199 Food and Drink Factory Workers nec

251311 Food Safety Auditor (S)
251311 Food Safety Officer (S)
234212 Food Scientist (A)
234212 Food Technologist
851299 Food Trades Assistants nec
452317 Football Coach (S)
452411 Footballer
839915 Footwear Factory Worker
711711 Footwear Production Machine Operator
222211 Foreign Exchange Dealer (S)
253915 Forensic Pathologist (S)
253411 Forensic Psychiatrist (S)
272311 Forensic Psychologist (S)
133511 Forest Logistics Manager (NZ) (S)
133511 Forest Manager (A)
234113 Forest Scientist (NZ)
234113 Forester (Aus)
234113 Forestry Adviser (S)
234113 Forestry Consultant (S)
311413 Forestry Technical Officer (S)
841311 Forestry Worker
721311 Fork Truck Operator (A)
721311 Forklift Driver
721311 Forklift Operator (A)
431511 Formal Service Waiter (S)
224999 Forms Designer (N)
331212 Formwork Carpenter (S)
721112 Forwarder Operator (S)
712311 Foundry Operator (S)
712916 Fourdrinier Machine Operator (S)
612111 Franchise Broker (S)
224214 Freedom of Information Officer (S)
591211 Freight Clerk (A)
891111 Freight Handler (Rail or Road)
891111 Freight Loader (A)
394211 French Polisher (S)
431411 Front Office Manager (Hotel) (S)
721216 Front-end Loader Operator (S)
839312 Fruit and Vegetable Classer (S)
831115 Fruit and Vegetable Factory Worker
311399 Fruit and Vegetable Inspector (N)
832113 Fruit and Vegetable Packer
121213 Fruit or Nut Farm Manager (A)
841211 Fruit or Nut Farm Worker
121213 Fruit or Nut Grower
841212 Fruit or Nut Picker
841912 Fumigator (S)
451311 Funeral Director
451399 Funeral Director's Assistant (N)
451399 Funeral Workers nec
393299 Fur Cutter (N)
393299 Fur Matcher (N)
712311 Furnace Operator (Metals) (S)
232312 Furniture Designer (S)
394211 Furniture Finisher
733113 Furniture Removalist
891112 Furniture Removalist's Assistant (S)
393311 Furniture Upholsterer (S)
393299 Furrier (N)
222212 Futures Trader

G

451411 Gallery or Museum Attendant (A)
224212 Gallery or Museum Curator
451411 Gallery or Museum Guide
399311 Gallery or Museum Technician
272199 Gambling Counsellor (N)
141411 Gaming Manager (S)
431311 Gaming Pit Boss (S)
431311 Gaming Worker
899611 Garbage Depot Worker (S)
841411 Garden Labourer
362211 Gardener (General)
399212 Gas Compressor Turbine Operator (S)
312611 Gas Examiner (S)
334114 Gas Main and Line Fitter (S)
399212 Gas or Petroleum Operator
839912 Gas Plant Worker (S)
312115 Gas Plumbing Inspector (S)
334114 Gasfitter
253316 Gastroenterologist
899921 Gatekeeper (S)
399411 Gem Setter (S)
111212 General (Army) (S)
531111 General Clerk
841611 General Farm Hand (A)
253111 General Medical Practitioner
253111 General Practitioner (A)
139111 General Service Officer (Army) (Aus) (S)
251999 Genetic Counsellor (N)
311412 Geochemical Laboratory Technician (S)
232212 Geodetic Surveyor (S)
272499 Geographer (N)
272411 Geographical Historian (S)
821915 Geological Survey Field Assistant (S)
311412 Geological Technical Officer (S)
234411 Geologist
232212 Geomatic Engineer (A)
232212 Geomatician (A)
234412 Geophysicist
311412 Geoscience Laboratory Technician (S)
441111 Geospatial Imagery Intelligence Analyst (Air Force) (S)
233212 Geotechnical Engineer
312212 Geotechnical Laboratory Technician (S)
252511 Gerentological Physiotherapist (S)
253411 Geriatric Psychiatrist (S)
253399 Geriatrician (N)
711716 Gill Box Operator (S)
121299 Ginger Farmer (N)
333111 Glass Beveller (S)
399999 Glass Blower (N)
232312 Glass Designer (S)
333111 Glass Embosser (S)
333111 Glass Etcher (S)

711113 Glass Furnace Operator (S)
711113 Glass Laminating Operator (S)
711113 Glass Maker (S)
711113 Glass Melt Operator (S)
839916 Glass Mould Cleaner (S)
839916 Glass Processing Worker
711113 Glass Production Machine Operator
333111 Glass Silverer (S)
711113 Glass Toughening Operator (S)
431911 Glassie (A)
711113 Glassware Maker (S)
839313 Glassware Verifier (S)
333111 Glazier
231113 Gliding Pilot Instructor (S)
121315 Goat Farmer
841599 Goat Herder (N)
399411 Goldsmith (S)
149113 Golf Course Manager (S)
452412 Golfer
421113 Governess (S)
111312 Government Minister (S)
721215 Grader Operator
811699 Graffiti Cleaner (N)
222111 Grain Buyer (S)
712912 Grain Handler (S)
831116 Grain Mill Worker
121214 Grain, Oilseed or Pasture Farm Manager (Aus) (A)
841213 Grain, Oilseed or Pasture Farm Worker (Aus)
121214 Grain, Oilseed or Pasture Grower (Aus)
121215 Grape Grower
232411 Graphic Artist (A)
232411 Graphic Designer
392211 Graphic Pre-press Trades Worker
821113 Grave Digger (S)
392311 Gravure Printing Machinist (S)
362311 Greenkeeper
272199 Grief Counsellor (N)
899999 Grip (N)
732111 Grocery Deliverer (S)
733112 Ground Crewman Aircraft Support (Army) (S)
441111 Ground Crewman Mission Support (Army) (S)
139111 Ground Defence Officer (Air Force) (Aus) (S)
231113 Ground School Instructor (A)
321211 Ground Support Equipment Fitter (Air Force) (S)
111212 Group Captain (Air Force) (S)
141999 Guest House Manager (N)
211213 Guitarist (S)
441111 Gun Number (Army) (S)
323312 Gunsmith
452111 Gym Instructor (S)
451899 Gymnasium Attendant (N)
452312 Gymnastics Coach or Instructor
253913 Gynaecological Oncologist (S)

H

451812 Hair or Beauty Salon Assistant
142114 Hair or Beauty Salon Manager
391111 Hairdresser
322111 Hammer Smith (S)
839917 Hand Flesher (S)
452318 Handicapper (Racing) (S)
249299 Handicrafts Teacher (Private Tuition) (N)
899311 Handyperson
452215 Hang-gliding Instructor (S)
532112 Hansard Reporter (S)
139999 Harbour Master (N)
839413 Hardboard Factory Worker (S)
621111 Hardware Sales Assistant (S)
313111 Hardware Technician
841313 Hardwood Faller (S)
133511 Harvest Manager (Forestry) (S)
721111 Harvester Operator (S)
121111 Hatchery Manager (Fish) (S)
121321 Hatchery Manager (Poultry) (S)
733111 Haulpak Driver (S)
253515 Head and Neck Surgeon (A)
431411 Head Housekeeper (S)
431411 Head Porter (Hotel) (S)
134411 Head Teacher (TAFE) (Aus) (S)
134311 Headmaster/mistress (A)
134299 Health and Welfare Services Managers nec
224213 Health Data Administrator (S)
251999 Health Diagnostic and Promotion Professionals nec
224311 Health Economist (S)
251911 Health Educator (A)
224213 Health Information Manager
552312 Health Insurance Assessor (S)
512211 Health Practice Manager
251911 Health Promotion Officer
272311 Health Psychologist (S)
312912 Heat Treatment Technician (S)
312511 Heating and Ventilating Technical Officer (S)
233512 Heating and Ventilation Engineer (S)
341111 Heavy Coil Winder (S)
231114 Helicopter Pilot
231113 Helicopter Pilot Instructor (S)
451511 Herbalist (Western) (S)
311111 Herd Tester (S)
272499 Heritage Consultant (N)
399999 Hide and Skin Classer (N)
711712 Hide and Skin Fleshing Machine Operator (S)
711712 Hide and Skin Processing Machine Operator
839917 Hide and Skin Processing Worker
271211 High Court Justice (S)
731111 Hire Car Driver (S)
272411 Historian
451511 Holistic Pulser (S)
821412 Home Improvement Installer
423111 Home Support Worker (A)
249111 Home-School Liaison Officer (S)
252212 Homoeopath
841299 Hop Farm Worker (N)
121299 Hop Farmer (N)
361112 Horse Breaker (S)

121316 Horse Breeder
451899 Horse Racing Analyst (N)
452313 Horse Riding Coach or Instructor
121316 Horse Stud Manager (A)
841516 Horse Stud Worker (S)
361112 Horse Trainer
452299 Horse Trekking Guide (N)
841412 Horticultural Nursery Assistant
311111 Horticultural Technical Officer (S)
111211 Hospital Administrator (S)
423311 Hospital Orderly
251511 Hospital Pharmacist
234611 Hospital Scientist (A)
542112 Hospital Ward Clerk (A)
431999 Hospitality Trainee (N)
431999 Hospitality Workers nec
149999 Hospitality, Retail and Service Managers nec
141999 Hostel Manager (N)
423412 Hostel Parent
431999 Hotel Cellar Hand (N)
431411 Hotel Concierge (S)
431411 Hotel Office Manager (S)
141311 Hotel or Motel Manager
542113 Hotel or Motel Receptionist
431411 Hotel Service Manager
431411 Hotel Service Supervisor (A)
899311 Hotel Useful (S)
899311 Hotel Yardperson (S)
141311 Hotelier (A)
423412 House Parent (A)
411711 Housing Officer (S)
223111 Human Resource Adviser
599411 Human Resource Clerk
223111 Human Resource Consultant (A)
132311 Human Resource Manager
599411 Human Resources Records Clerk (A)
841911 Hunter (A)
841911 Hunter-Trapper
452213 Hunting Guide
312512 Hydraulic Controls Technician (S)
721214 Hydraulic Rockbreaker Operator (S)
233211 Hydraulics Engineer (S)
399213 Hydro-electric Station Operator (S)
231213 Hydrofoil Captain (S)
234411 Hydrogeologist (S)
311412 Hydrographer (S)
232212 Hydrographic Survey Operator (Navy) (S)
232212 Hydrographic Surveyor (S)
311412 Hydrographical Technical Officer (S)
311412 Hydrological Technical Officer (S)
234912 Hydrometallurgical Engineer (S)
399911 Hyperbaric Welder Diver (S)
252299 Hypnotherapist (N)

I

831199 Ice-cream Maker (N)
621711 Ice-cream Van Vendor (S)
225211 ICT Account Manager
261111 ICT Business Analyst
225212 ICT Business Development Manager
313112 ICT Customer Support Officer
261312 ICT Developer (A)
135112 ICT Development Manager (S)
223211 ICT Educator (A)
313112 ICT Help Desk Officer (A)
313112 ICT Help Desk Technician (A)
135199 ICT Managers nec
261312 ICT Programmer (A)
135112 ICT Project Manager
263211 ICT Quality Assurance Engineer
621211 ICT Sales Assistant
225213 ICT Sales Representative
262112 ICT Security Specialist
263299 ICT Support and Test Engineers nec
263212 ICT Support Engineer
313199 ICT Support Technicians nec
263213 ICT Systems Test Engineer
223211 ICT Trainer
232412 Illustrator
272211 Imam (S)
224913 Immigration Consultant (NZ)
599512 Immigration Officer
253915 Immunologist (S)
133311 Importer or Exporter
591212 Import-Export Clerk
841999 Indoor Plant Technician (N)
149113 Indoor Sports Centre Manager (S)
234211 Industrial Chemist (S)
271212 Industrial Court Magistrate (S)
232312 Industrial Designer
233511 Industrial Engineer
233914 Industrial Engineering Technologist (S)
399211 Industrial Gas Production Operator (S)
621912 Industrial Hire Sales Assistant (S)
342315 Industrial Measurement and Control Technician (A)
253399 Industrial Medicine Specialist (N)
441211 Industrial Paramedic (S)
251512 Industrial Pharmacist
272313 Industrial Psychologist (A)
271213 Industrial Relations Commissioner (S)
223113 Industrial Relations Officer (A)
334115 Industrial Roof Plumber (S)
711211 Industrial Spraypainter
224712 Industry Analyst (S)
139111 Infantry Officer (Army) (S)
253399 Infectious Diseases Specialist (N)
224999 Information and Organisation Professionals nec
541211 Information Clerk (S)
262112 Information Technology Security Manager (S)
711514 Injection Moulding Machine Operator (Plastics) (S)
541211 Inquiry Clerk
541211 Inquiry Officer (Aus) (A)
221111 Insolvency Practitioner (S)
139112 Inspector (Fire Services) (S)

599599 Inspectors and Regulatory Officers nec
392312 Instant Printer Operator (A)
232413 Instructional Designer (S)
342314 Instrument and Control Service Person (A)
211213 Instrumentalist (A)
711511 Insulation Extruder Operator (S)
611211 Insurance Agent
222113 Insurance Broker
552312 Insurance Clerk (A)
552312 Insurance Consultant
599611 Insurance Investigator
599612 Insurance Loss Adjuster
599612 Insurance Loss Assessor (A)
599613 Insurance Risk Surveyor
611211 Insurance Underwriter (S)
422112 Integration Aide
224411 Intelligence Officer
411112 Intensive Care Ambulance Paramedic (Aus)
253211 Intensive Care Anaesthetist (S)
253317 Intensive Care Medicine Specialist (A)
253317 Intensive Care Specialist
253317 Intensivist (A)
232413 Interactive Media Designer (A)
399912 Interior Decorator
232511 Interior Designer
241311 Intermediate School Teacher (NZ)
221214 Internal Auditor
253399 Internal Medicine Specialists nec
591114 Internal Salesperson (Aus) (A)
451612 International Travel Consultant (S)
141111 Internet Cafe Manager (S)
135199 Internet Service Provider (N)
272412 Interpreter
561511 Interviewer (A)
591115 Inventory Clerk (S)
712916 Inverform Machine Operator (S)
551111 Investment Accounting Clerk (S)
222199 Investment Broker (N)
222299 Investment Dealer (N)
451511 Iridologist (S)
839313 Iron Pellet Tester (S)
811513 Ironer or Presser
841999 Irrigationist (N)

J

899111 Janitor (S)
211214 Jazz Singer (S)
399411 Jeweller
232313 Jewellery Designer
323412 Jigmaker (Metal) (S)
394213 Jigmaker (Wood) (S)
452413 Jockey
331213 Joiner
839413 Joinery Factory Worker (S)
331213 Joinery Machinist (S)
331213 Joinery Patternmaker (S)
331213 Joinery Setter-out (S)
212499 Journalists and Other Writers nec
271211 Judge
271299 Judicial and Other Legal Professionals nec
271299 Judicial Registrar (Aus) (N)
712211 Jumbo Operator (S)
411716 Juvenile Justice Officer (S)

K

241112 Kaiako Kōhanga Reo (Māori Language Nest Teacher)
241211 Kaiako Kura Kaupapa Māori (Māori-medium Primary School Teacher)
411512 Kaiāwhina (Hauora) (Māori Health Assistant)
422113 Kaiāwhina Kōhanga Reo (Māori Language Nest Assistant)
422114 Kaiāwhina Kura Kaupapa Māori (Māori-medium School Assistant)
399999 Kayak Maker (N)
841999 Kelp or Seagrass Gatherer (N)
361199 Kennel Hand (N)
561199 Keno Terminal Operator (N)
839913 Kiln Labourer (S)
712311 Kiln Operator (Metals) (S)
422115 Kindergarten Assistant (S)
241111 Kindergarten Teacher (A)
451511 Kinesiologist (S)
851311 Kitchen Steward (A)
851311 Kitchenhand
711713 Knitting Machine Operator
224999 Knowledge Manager (N)

L

392311 Label Printing Machinist (S)
832112 Labelling Machine Operator (S)
139913 Laboratory Manager
224311 Labour Market Economist (S)
899999 Labourers nec
821913 Lagger
711514 Lamination Machine Operator (S)
263112 LAN Administrator (S)
234313 Land Degradation Analyst (S)
224511 Land Economist
232611 Land Planner (S)
234311 Landcare Facilitator (S)
234111 Landcare Officer (S)
232112 Landscape Architect
362213 Landscape Gardener
211311 Landscape Photographer (S)
399411 Lapidary (S)
253515 Laryngologist (S)
839212 Latex Foam Worker (S)
149999 Laundrette Owner (N)
811511 Laundry Worker (General)
841299 Lavender Farm Worker (N)
599214 Law Clerk
271299 Law Researcher (N)
841411 Lawn Mower (S)
321214 Lawnmower Mechanic (S)
591114 Lay-by Clerk (Aus) (S)

212413 Leader Writer (S)
211499 Leadlighter (N)
899915 Leaflet or Newspaper Deliverer
452315 Learn to Swim Instructor (S)
222112 Lease Broker (S)
393112 Leather Goods Maker
711712 Leather Production Machine Operator (A)
224211 Legal Archivist (S)
599214 Legal Clerk (A)
599112 Legal Executive
224611 Legal Librarian (S)
271299 Legal Officer (N)
512299 Legal Practice Manager (N)
521212 Legal Secretary
111399 Legislators nec
232311 Leisurewear Designer (S)
552211 Lending Consultant (A)
392311 Letterpress Printing Machinist (S)
224912 Liaison Officer
224611 Librarian
599711 Library Assistant
599711 Library Attendant (A)
599711 Library Clerk (A)
399312 Library Technician
323112 Licensed Aircraft Maintenance Engineer (Airframes) (S)
323111 Licensed Aircraft Maintenance Engineer (Electrical) (S)
323112 Licensed Aircraft Maintenance Engineer (Engines) (S)
323111 Licensed Aircraft Maintenance Engineer (Instruments) (S)
323111 Licensed Aircraft Maintenance Engineer (Radio) (S)
141411 Licensed Club Manager
712922 Licensed Weigher (S)
271212 Licensing Court Magistrate (S)
111212 Lieutenant Colonel (Army) (S)
111212 Lieutenant General (Army) (S)
611211 Life Assurance Representative (S)
272199 Life Coach (N)
311413 Life Science Technical Officer (A)
311413 Life Science Technician
234511 Life Scientist (General)
234599 Life Scientists nec
452414 Lifeguard
341113 Lift Electrician (A)
341113 Lift Mechanic
312611 Lifts and Cranes Inspector (S)
832211 Light Coil Winder (S)
399513 Light Technician
212399 Lighting Director (N)
731111 Limousine Driver (S)
721912 Linemarker
811511 Linen Sorter (S)
452322 Linesperson (Sport) (S)
272499 Linguist (N)
711999 Linoleum Maker (N)
334114 Liquefied Petroleum Gasfitter (S)
223112 Literary Agent (S)
392311 Lithographic Printing Machinist (S)
841599 Livestock Farm Workers nec
121399 Livestock Farmers nec
733111 Livestock Haulier (S)
222111 Livestock Trader (S)
121399 Llama Farmer (N)
721216 Loader Operator
552211 Loans Consultant (A)
552211 Loans Officer (A)
224999 Lobbyist (N)
111311 Local Government Legislator
212399 Location Manager (Film or Television) (N)
712999 Lock Master (Water Transport) (N)
323313 Locksmith
731311 Locomotive Driver (A)
599517 Locomotive Inspector (S)
731311 Locomotive Observer (S)
399112 Loftsman/woman (Marine) (S)
711312 Log Preparer (S)
841312 Logging Assistant
721112 Logging Plant Operator
733111 Logging Truck Driver (S)
591112 Logistics Clerk (S)
133611 Logistics Manager (S)
133611 Logistics Officer (Air Force) (S)
711715 Loom Operator (A)
323215 Loom Tuner (S)
621999 Lotteries Agent (N)
899916 Lube Attendant (S)
121214 Lucerne Farmer (S)

M

711999 Machine Operators nec
532112 Machine Shorthand Reporter
211113 Magician/Illusionist (S)
271212 Magistrate
251211 Magnetic Resonance Technologist (S)
312912 Magnetic Testing Technician (S)
561411 Mail Clerk
561411 Mail Officer (A)
591111 Mail Order Clerk (S)
392111 Mailhouse Operator (Aus) (S)
323211 Maintenance Fitter (S)
312911 Maintenance Planner
312911 Maintenance Scheduler (A)
111212 Major General (Army) (S)
399514 Make Up Artist
234518 Mammologist (S)
221112 Management Accountant
224711 Management Consultant
134299 Manager of Allied Health Services (N)
111211 Managing Editor (S)
451111 Manicurist (S)
133411 Manufacturer
741111 Manufacturing Storeperson (S)
224211 Manuscripts Archivist (S)
225311 Māori Liaison Officer (NZ) (S)
252511 Māori Physiotherapist (NZ) (S)
232211 Map Maker (A)

711114 Marble Cutter (S)
831199 Margarine Maker (N)
149999 Marina Manager (N)
234516 Marine Biologist
231299 Marine Certification and Surveillance Manager (N)
233916 Marine Designer (NZ)
712911 Marine Engine Driver (S)
231212 Marine Engineer (A)
231215 Marine Engineer Surveyor (A)
234411 Marine Geologist (S)
231299 Marine Safety Officer (N)
441111 Marine Specialist (Army) (S)
451799 Marine Steward (N)
321211 Marine Technician (Navy) (S)
231299 Marine Transport Professionals nec
841214 Market Garden Worker (NZ)
121212 Market Gardener (Flowers) (Aus) (S)
121213 Market Gardener (Fruit) (Aus) (S)
121221 Market Gardener (NZ)
121221 Market Gardener (Vegetables) (Aus) (S)
225112 Market Research Analyst
561511 Market Research Interviewer (S)
131112 Market Research Manager (S)
621713 Market Stall Vendor (S)
225113 Marketing Consultant (A)
225113 Marketing Coordinator (A)
225113 Marketing Officer (A)
225113 Marketing Specialist
411611 Massage Therapist
231211 Master Fisher
233112 Materials Engineer
621911 Materials Recycler
224112 Mathematician
393311 Mattress Maker (S)
111311 Mayor (S)
732111 Meals on Wheels Driver (S)
831211 Meat Boner and Slicer
839312 Meat Grader (S)
311312 Meat Inspector
832114 Meat Packer
831311 Meat Process Worker
831211 Meat Trimmer (S)
323211 Mechanic (Diesel and Heavy Earthmoving Equipment) (S)
733115 Mechanic Recovery (Army) (S)
233512 Mechanical Engineer
312511 Mechanical Engineering Draftsperson
231212 Mechanical Engineering Officer (Navy) (S)
312512 Mechanical Engineering Technician
312512 Mechanical Laboratory Technician (S)
899916 Mechanic's Assistant
233999 Mechatronics Engineer (N)
222111 Media Buyer (S)
225311 Media Liaison Officer (S)
561999 Media Monitor (Aus) (N)
212112 Media Producer (excluding Video)
134211 Medical Administrator (Aus)
411411 Medical Assistant (Defence) (S)
134299 Medical Corps Officer (Army) (N)
251211 Medical Diagnostic Radiographer
233913 Medical Engineer (S)
393114 Medical Grade Shoemaker (S)
253916 Medical Imaging Specialist (S)
251211 Medical Imaging Technologist (A)
253112 Medical Intern (S)
234611 Medical Laboratory Scientist
311213 Medical Laboratory Technical Officer (A)
311213 Medical Laboratory Technician
134211 Medical Manager (A)
253111 Medical Officer (Navy) (S)
234914 Medical Physicist (S)
512211 Medical Practice Manager (S)
253999 Medical Practitioners nec
253411 Medical Psychotherapist (S)
251212 Medical Radiation Therapist
542114 Medical Receptionist
599911 Medical Record Clerk (S)
224213 Medical Records Administrator (A)
225412 Medical Representative (S)
234611 Medical Scientific Officer (A)
134211 Medical Superintendent (NZ)
311299 Medical Technicians nec
111312 Member of Parliament
111312 Member of the Legislative Assembly (Aus) (S)
111312 Member of the Legislative Council (Aus) (S)
254416 Mental Retardation Nurse (A)
599311 Mercantile Agent (Aus) (A)
639211 Merchandise Planner (S)
639112 Merchandiser (A)
141111 Mess Supervisor (S)
322114 Metal Casting Trades Worker
839111 Metal Engineering Process Worker
322311 Metal Fabricator
322311 Metal Fabricator-Welder (S)
323299 Metal Fitters and Machinists nec
839111 Metal Forger's Assistant (S)
323214 Metal Machine Setter (S)
323214 Metal Machinist (First Class)
322114 Metal Moulder (S)
839111 Metal Moulder's Assistant (S)
322115 Metal Polisher
839311 Metal Products Viewer (S)
712311 Metal Rolling Mill Operator (S)
322211 Metal Spinner (S)
322311 Metal Template Maker (S)
323214 Metal Turner (S)
234912 Metallographer (S)
312912 Metallurgical or Materials Technician
234912 Metallurgist
312912 Metallurgy Laboratory Technician (S)
311412 Meteorological Observer (S)
234913 Meteorologist
561912 Meter Reader
234517 Microbiologist
399999 Micrographic Technician (N)

399599 Microphone Boom Operator (N)
134311 Middle or Intermediate School Principal (S)
241311 Middle School Teacher (Aus)
254111 Midwife
254111 Midwife Practitioner (S)
241599 Migrant Teacher (N)
224913 Migration Agent (Aus)
224913 Migration Consultant (A)
139111 Military Police Officer (Air Force) (NZ) (S)
139111 Military Police Officer (Army) (S)
139111 Military Police Officer (Navy) (NZ) (S)
839312 Milk and Cream Grader (S)
831114 Milk Processing Worker (S)
899999 Milk Runner (N)
733114 Milk Tanker Driver (S)
831114 Milk Treater (S)
621711 Milk Vendor (S)
399999 Milking Machine Technician (N)
393299 Milliner (N)
323214 Milling Machinist (S)
211111 Mime Artist (S)
312913 Mine Deputy
133513 Mine Manager (A)
133513 Mine Superintendent (A)
712212 Miner
224311 Mineral Economist (S)
821914 Mineral Ore Processing Labourer (A)
712311 Mineral Ore Treatment Plant Operator (S)
234999 Mineral Processing Engineer (N)
312611 Mines Inspector (S)
731211 Minibus Driver (S)
711411 Minilab Operator (S)
312999 Mining Detail Draftsperson (N)
233611 Mining Engineer (excluding Petroleum)
233914 Mining Engineering Technologist (S)
712212 Mining Plant Operator (A)
821914 Mining Support Worker
312913 Mining Technician (S)
272211 Minister of Religion
841611 Mixed Crop and Livestock Farm Worker
121411 Mixed Crop and Livestock Farmer
121216 Mixed Crop Farmer
841513 Mixed Livestock Farm Worker
121317 Mixed Livestock Farmer
442217 Mobile Patrol Officer (S)
621211 Mobile Phone Salesperson (S)
721999 Mobile Plant Operators nec
639111 Model
234514 Molecular Biologist (S)
234514 Molecular Geneticist (S)
552313 Money Market Clerk
222211 Money Market Dealer (A)
272211 Monk (S)
331112 Monumental Stonemason (S)
222112 Mortgage Broker (S)
451311 Mortician (A)
311299 Mortuary Technician (N)
333411 Mosaic Tiler (S)
411412 Mothercraft Nurse
711912 Motion Picture Projectionist
211199 Motivational Speaker (N)
321211 Motor Mechanic (General)
599513 Motor Vehicle Licence Examiner
621311 Motor Vehicle or Caravan Salesperson
899411 Motor Vehicle Parts and Accessories Fitter (General)
621312 Motor Vehicle Parts Interpreter (Aus)
621311 Motor Vehicle Salesperson (A)
561211 Motorbike Courier (S)
321213 Motorcycle Mechanic
451211 Motorcycle Riding Instructor (S)
712911 Motorman/woman (Fluids Drilling) (S)
452214 Mountain or Glacier Guide
441312 Mounted Police Officer (S)
233612 Mud Engineer (S)
721999 Mulcher Operator (N)
361199 Muleser (N)
224611 Multicultural Services Librarian (S)
211499 Multimedia Artist (N)
232413 Multimedia Designer
261211 Multimedia Developer (A)
261211 Multimedia Programmer (A)
261211 Multimedia Specialist
839912 Munitions Factory Worker (S)
252511 Musculoskeletal Physiotherapist (S)
253399 Musculoskeletal Specialist (NZ) (N)
224999 Museum Registrar (N)
211211 Music Arranger (S)
211299 Music Copyist (N)
211212 Music Director
211299 Music Professionals nec
211299 Music Researcher (N)
249214 Music Teacher (Private Tuition)
252299 Music Therapist (N)
399515 Musical Instrument Maker or Repairer
211213 Musician (Instrumental)
121111 Mussel Farmer (S)
639911 Mystery Shopper (S)

N

451111 Nail Technician (S)
421113 Nanny
234999 Natural and Physical Science Professionals nec
451511 Natural Remedy Consultant
233912 Natural Resources Engineer (A)
252213 Naturopath
233916 Naval Architect (Aus)
441111 Naval Police Coxswain (Navy) (S)
231214 Navigating Officer (Ship's) (S)
231199 Navigator (Air Force) (N)
111212 Navy Senior Officer (A)
253321 Neonatologist (S)
253322 Nephrologist (A)
711799 Net Maker (N)
263112 Network Administrator

263113 Network Analyst
263113 Network Architect (S)
263113 Network Consultant (A)
263113 Network Designer (A)
135199 Network Manager (N)
261312 Network Programmer (S)
263112 Network Specialist (A)
263113 Network Strategist (A)
263112 Network Support (A)
313112 Network Support Technician (S)
234512 Neuroanatomist (S)
252511 Neurological Physiotherapist (S)
253318 Neurologist
311299 Neurophysiological Technician (N)
272311 Neuropsychologist (S)
253513 Neurosurgeon
211499 New Media Artist (N)
212412 News Editor (S)
211311 News Photographer (S)
142111 Newsagent (S)
212412 Newspaper or Periodical Editor
212413 Newspaper Reporter (S)
891211 Night Filler (A)
141411 Nightclub Manager (S)
711911 Nitrocellulose Maker (S)
312912 Non-destructive Testing Technician (S)
212211 Novelist (S)
599514 Noxious Weeds and Pest Inspector
253916 Nuclear Medicine Specialist (S)
251213 Nuclear Medicine Technologist
254211 Nurse Educator
254311 Nurse Manager
254411 Nurse Practitioner
254212 Nurse Researcher
254311 Nurse Supervisor (A)
254311 Nurse Unit Manager (A)
841412 Nursery Hand (Horticulture) (A)
362411 Nurseryperson
423312 Nurses' Aide (NZ) (A)
149999 Nursing Agency Manager (N)
411411 Nursing Assistant (NZ) (A)
134212 Nursing Clinical Director
254499 Nursing Officer (Defence Forces) (N)
423312 Nursing Support Worker
251111 Nutritionist (A)

O

139111 Observer (Navy) (S)
253211 Obstetric Anaesthetist (S)
253913 Obstetrician and Gynaecologist
251312 Occupational Health and Safety Adviser
251312 Occupational Health and Safety Coordinator (A)
312611 Occupational Health and Safety Inspector (S)
132311 Occupational Health and Safety Manager (S)
251312 Occupational Health and Safety Officer (A)
252511 Occupational Health Physiotherapist (S)
251312 Occupational Hygienist (S)
253999 Occupational Medicine Specialist (N)
272313 Occupational Psychologist (A)
252411 Occupational Therapist
423314 Occupational Therapist's Assistant (S)
452212 Ocean Fishing Guide (S)
234213 Oenologist (A)
831311 Offal Separator (S)
631112 Office Cashier
512111 Office Manager
733113 Office Mover (S)
399911 Offshore Diver (S)
399212 Oil and Gas Well Treatment Operator (A)
399212 Oil, Gas and Pipe Tester (A)
712999 Oilseed Processing Operator (N)
121213 Olive Grower (S)
399911 Onshore Diver (S)
712212 Opal Miner (S)
399411 Opal Polisher (S)
312913 Open Cut Examiner (S)
211214 Opera Singer (S)
254423 Operating Room Nurse (A)
311214 Operating Theatre Technician
133511 Operations Manager (Forestry) (S)
133512 Operations Manager (Production) (S)
224112 Operations Research Analyst (S)
441111 Operator Artillery Meteorology and Surveyor (Army) (S)
342413 Operator Bearer Systems (Army) (S)
313112 Operator Command Support Systems (Army) (S)
441111 Operator Electronic Warfare (Army) (S)
441111 Operator Ground Based Air Defence (Army) (S)
342312 Operator Specialist Communications (Army) (S)
741111 Operator Supply (Army) (S)
441111 Operator Weapon Locating Radar (Army) (S)
253914 Ophthalmologist
711511 Optic Fibre Drawer (S)
399913 Optical Dispenser (Aus)
399914 Optical Mechanic
311499 Optics Technical Officer (N)
251411 Optometrist
252311 Oral and Maxillofacial Surgeon (S)
411214 Oral Health Therapist (S)
252311 Oral Pathologist (S)
841211 Orchard Worker (S)
121213 Orchardist (S)
211212 Orchestra Conductor (S)
211211 Orchestrator (S)
591111 Order Clerk
741111 Order Picker/Assembler (S)
133611 Ordnance Corps Officer (Army) (S)
224712 Organisation and Methods Analyst
272313 Organisational Psychologist
252214 Oriental Medicine Practitioner (A)
234518 Ornithologist (S)
252311 Orthodontist (S)
253514 Orthopaedic Surgeon
251412 Orthoptist
311299 Orthotic and Prosthetic Technician (N)

251912 Orthotist or Prosthetist
252112 Osteopath
841599 Ostrich Farm Worker (N)
121399 Ostrich Farmer (N)
639911 Other Sales Support Worker
452317 Other Sports Coach or Instructor
452323 Other Sports Official
253515 Otologist (S)
253515 Otorhinolaryngologist
421114 Out of School Hours Care Worker
321214 Outboard Motor Mechanic (S)
452299 Outdoor Adventure Guides nec
452215 Outdoor Adventure Instructor
452215 Outdoor Adventure Leader (A)
452215 Outdoor Education Teacher (S)
452215 Outdoor Pursuits Instructor (S)
731199 Oversize Load Pilot Escort (N)
121111 Oyster Farmer (S)
831313 Oyster Opener (S)

P

832199 Packers nec
252511 Paediatric Physiotherapist (S)
253516 Paediatric Surgeon
253321 Paediatric Thoracic Physician (S)
253321 Paediatrician
252311 Paedodontist (S)
253211 Pain Management Specialist (S)
839912 Paint Factory Worker (S)
399211 Paint Maker (S)
711911 Paint Tinter (S)
211411 Painter (Artistic) (A)
211411 Painter (Visual Arts)
332211 Painting Trades Worker
234411 Palaeontologist (S)
712912 Palletiser Operator (S)
253399 Palliative Medicine Specialist (N)
394213 Panel Saw Operator (S)
324111 Panelbeater
851311 Pantry Attendant (S)
712916 Paper and Pulp Mill Operator
839411 Paper and Pulp Mill Worker
711311 Paper Bag Making Machinist (S)
392111 Paper Guillotine Operator (Bookbinding) (S)
712916 Paper Machine Operator (A)
712916 Paper Maker (A)
711311 Paper Products Machine Operator
712916 Paper Rewinder Operator (S)
712916 Paperboard Machine Operator (S)
332211 Paperhanger (S)
399999 Parachute Rigger (N)
452215 Paragliding Instructor (S)
411111 Paramedic (Aus) (A)
423312 Paramedical Aide (S)
234599 Parasitologist (N)
561211 Parcel Contractor (S)
561412 Parcel Post Officer (S)
234314 Park Ranger
561913 Parking Inspector
111312 Parliamentarian (A)
224211 Parliamentary Archivist (S)
271299 Parliamentary Counsel (N)
224611 Parliamentary Librarian (S)
272499 Parole Board Member (N)
411714 Parole or Probation Officer
332111 Parquetry Layer (S)
839413 Particleboard Factory Worker (S)
621712 Party Plan Salesperson (S)
731213 Passenger Coach Driver
831114 Pasteuriser Operator (S)
351112 Pastrycook
851211 Pastrycook's Assistant
271299 Patent Attorney (N)
224914 Patents Examiner
253915 Pathologist
423311 Patient Services Assistant (A)
411111 Patient Transport Officer (Aus) (S)
393212 Pattern Grader (Clothing) (S)
393212 Patternmaker-Grader (S)
821511 Paving and Surfacing Labourer
721913 Paving Plant Operator
551311 Pay Clerk (A)
551311 Payroll Clerk
551311 Payroll Officer (A)
399911 Pearl Diver (S)
399999 Pearl Technician (N)
711512 Pelletising Extruder Operator (S)
561112 Penciller (A)
211199 Performance Artist (N)
399599 Performing Arts Road Manager (N)
399599 Performing Arts Technicians nec
311299 Perfusionist (N)
252311 Periodontist (S)
521111 Personal Assistant
423313 Personal Care Assistant
423111 Personal Care Worker (A)
423111 Personal Carer (A)
451899 Personal Service Workers nec
639911 Personal Shopper (S)
132311 Personnel and Employee Relations Manager (A)
223111 Personnel Officer (A)
599411 Personnel Records Clerk (A)
841912 Pest or Weed Controller
361113 Pet Groomer
733114 Petrol Tanker Driver (S)
399212 Petroleum and Gas Refining and Pumping Operator (A)
399212 Petroleum Blending Plant Operator (S)
233612 Petroleum Engineer
311411 Petroleum Laboratory Technician (S)
312912 Petroleum Products Laboratory Technician (S)
312912 Petroleum Refinery Laboratory Technician (S)
399212 Petroleum Terminal Plant Operator (S)
233612 Petrophysical Engineer (S)
251511 Pharmaceutical Officer (Army) (S)

399211 Pharmaceutical Plant Operator (S)
234599 Pharmacologist (Non-clinical) (N)
621411 Pharmacy Sales Assistant
311215 Pharmacy Technician
232212 Photogrammetric Surveyor (S)
312116 Photogrammetrist (S)
211311 Photographer
399915 Photographer's Assistant
211311 Photographic Artist (S)
711411 Photographic Developer and Printer
711411 Photographic Enlarger Operator (S)
452111 Physical Fitness Trainer (S)
234412 Physical Oceanographer (S)
252511 Physical Therapist (A)
234914 Physicist
311499 Physics Technical Officer (N)
252511 Physiotherapist
423314 Physiotherapist's Assistant (S)
512211 Physiotherapy Practice Manager (S)
211213 Pianist (S)
733113 Piano Removalist (S)
399515 Piano Tuner (S)
394212 Picture Framer
212412 Pictures Editor (S)
121318 Pig Breeder (S)
121318 Pig Farm Manager (A)
121318 Pig Farmer
841599 Piggery Worker (N)
712111 Pile Driver (S)
399211 Pilot Plant Operator (S)
312512 Pipe Testing Technician (S)
821914 Pit Crew Support Worker (S)
441311 Plain Clothes Police Officer (A)
224512 Plant and Machinery Valuer (S)
133512 Plant Manager (Manufacturing) (Aus) (S)
323211 Plant Mechanic (S)
234515 Plant Morphologist (S)
234515 Plant Pathologist (S)
234515 Plant Physiologist (S)
362411 Plant Propagator (S)
234515 Plant Taxonomist (S)
711199 Plaster Caster (N)
711199 Plaster Machine Operator (N)
253517 Plastic and Reconstructive Surgeon
711511 Plastic Cablemaking Machine Operator
711512 Plastic Compounding and Reclamation Machine Operator
323412 Plastic Mould Maker (S)
711514 Plastic Production Machine Setter (S)
711599 Plastics and Rubber Production Machine Operators nec
711513 Plastics Fabricator or Welder
839211 Plastics Factory Worker
399916 Plastics Fitter (A)
839211 Plastics Process Hand (A)
711514 Plastics Production Machine Operator (General)
399916 Plastics Technician
212211 Playwright (S)
334111 Plumber (General)
821114 Plumber's Assistant
312211 Plumbing Engineering Draftsperson (S)
312115 Plumbing Inspector
711312 Plywood and Veneer Repairer (S)
839413 Plywood Factory Worker (S)
252611 Podiatric Surgeon (S)
252611 Podiatrist
212211 Poet (S)
899711 Poker Machine Attendant (S)
224912 Police Liaison Officer (S)
441312 Police Officer
224412 Policy Adviser (A)
224412 Policy Analyst
132411 Policy and Planning Manager
132411 Policy Development Manager (S)
272499 Political Scientist (N)
452313 Polo Coach (S)
234999 Polymer Scientist (N)
134499 Polytechnic Registrar (NZ) (N)
242211 Polytechnic Teacher (NZ)
211214 Pop Singer (S)
711111 Porcelain Turner (S)
712111 Portainer Operator (S)
222312 Portfolio Manager (A)
211311 Portrait Photographer (S)
142115 Post Office Manager
561212 Postal Delivery Officer
561412 Postal Sorting Officer
561212 Postie (A)
211412 Potter or Ceramic Artist
241212 Pouako Kura Kaupapa Māori (Māori-medium Primary School Senior Teacher)
831312 Poultry Boner (S)
121321 Poultry Farm Manager (A)
841514 Poultry Farm Worker
121321 Poultry Farmer
831312 Poultry Process Worker
831312 Poultry Slaughterer (S)
311111 Poultry Technical Officer (S)
711211 Powder Coater (S)
711512 Powder Hand (Plastics) (S)
712213 Powder Monkey (Aus) (A)
399213 Power Generation Plant Operator
399213 Power Generation Turbine Room Operator (S)
712211 Power Tong Operator (S)
512299 Practice Managers nec
254421 Practice Nurse (A)
899212 Prawn Trawler Hand (S)
323314 Precision Instrument Maker and Repairer
272499 Prehistorian (N)
111312 Premier (Aus) (S)
712914 Premix Concrete Batcher (S)
249111 Preschool Adviser (S)
422115 Preschool Aide
241111 Preschool Director (S)
225311 Press Officer (S)
323412 Press-tool Maker (S)

312912 Pressure Testing Technician (S)
322312 Pressure Welder
711999 Pressurised Container Filler (N)
272211 Priest (S)
254421 Primary Health Care Nurse (NZ) (A)
134213 Primary Health Organisation Manager
311399 Primary Products Inspectors nec
134311 Primary School Principal (S)
241213 Primary School Teacher
241213 Primary School Teacher-Librarian (S)
111312 Prime Minister (S)
392111 Print Finisher (A)
212413 Print Journalist
312412 Printed Circuit Board Designer (S)
899511 Printer's Assistant
899512 Printing Bindery Assistant (A)
323299 Printing Engineer (N)
392311 Printing Machinist
899512 Printing Table Hand (A)
899512 Printing Table Worker
442111 Prison Officer
442214 Private Inquiry Agent (A)
442214 Private Investigator
249299 Private Tutors and Teachers nec
224712 Procedures Analyst (A)
312412 Process Control Technician (S)
233511 Process Engineer (Industrial) (S)
233611 Process Engineer (Mining) (S)
591113 Procurement Clerk (A)
832211 Product Assembler
233999 Product Design Engineer (N)
232312 Product Designer (A)
839311 Product Examiner
839312 Product Grader
225113 Product Manager (S)
839313 Product Tester
599912 Production Assistant (Film, Television, Radio or Stage)
591112 Production Clerk
212311 Production Designer (A)
133511 Production Manager (Forestry)
133512 Production Manager (Manufacturing)
133513 Production Manager (Mining)
233513 Production or Plant Engineer
591112 Production Recorder (A)
133112 Professional Builder (A)
212315 Program Director (Television or Radio)
511112 Program or Project Administrator
261311 Programmer Analyst (A)
133112 Project Builder
511112 Project Coordinator (A)
134499 Project Coordinator (Education) (N)
225311 Promotions Officer (S)
599913 Proof Reader
331212 Prop and Scenery Maker (S)
224511 Property Economist (A)
612112 Property Manager
612115 Property Portfolio Officer (S)
431999 Property Steward (N)
224512 Property Valuer (S)
252311 Prosthodontist (S)
451813 Prostitute (A)
234513 Protein Chemist (S)
254422 Psychiatric Nurse (A)
253411 Psychiatrist
599411 Psychological Examiner (Army) (S)
272399 Psychologists nec
254422 Psychopaedic Nurse (NZ) (S)
272314 Psychotherapist
131111 Public Affairs Manager (S)
225311 Public Affairs Officer (S)
253999 Public Health Physician (N)
132411 Public Policy Manager (A)
225311 Public Relations Consultant (S)
225311 Public Relations Officer (S)
225311 Public Relations Professional
211199 Public Speaker (N)
141311 Publican (A)
232411 Publication Designer (S)
253324 Pulmonary Specialist (S)
839411 Pulp, Paper Making and Paper Products Labourer (A)
211113 Puppeteer (S)
591113 Purchasing Officer
899212 Purse Seining Hand (S)
234912 Pyrometallurgical Engineer (S)
399999 Pyrotechnician (N)

Q

263211 Quality Analyst (ICT) (A)
839311 Quality Assurance Assessor (A)
139914 Quality Assurance Manager
224712 Quality Auditor (S)
139914 Quality Certification Manager (NZ) (S)
839311 Quality Control Assessor (A)
139914 Quality Facilitator (A)
263211 Quality Manager (ICT) (A)
263211 Quality Specialist (ICT) (A)
233213 Quantity Surveyor
311313 Quarantine Inspector (A)
311313 Quarantine Officer
133513 Quarry Manager (S)
271111 Queen's Counsel (S)

R

272211 Rabbi (S)
452318 Race Steward (A)
899999 Racecourse Barrier Attendant (N)
452499 Racing Driver (N)
253916 Radiation Oncologist (S)
899414 Radiator Fitter
212113 Radio Announcer (A)
599914 Radio Despatcher
212414 Radio Journalist
212113 Radio Presenter

212414 Radio Reporter (A)
313211 Radiocommunications Technician
234912 Radiological Metallurgist (S)
253916 Radiologist
731311 Rail Car Driver (S)
821611 Railway Fettler (A)
442217 Railway Patrol Officer (S)
712917 Railway Shunter (S)
341111 Railway Signal Electrician (S)
712917 Railway Signal Operator
233311 Railway Signalling Engineer (S)
451799 Railway Sleeping Car Conductor (N)
149412 Railway Station Manager
451799 Railway Steward (N)
712917 Railway Switching and Signalling Operator (A)
721914 Railway Track Plant Operator
821611 Railway Track Worker
342211 Railway Traction Line Worker (S)
712917 Railway Yard Assistant (S)
899917 Railways Assistant
712211 Raise Drill Operator (S)
721911 Ramp Agent (S)
272199 Rape Crisis Counsellor (N)
721311 Reach Truck Operator (S)
612113 Real Estate Agency Licensee (NZ)
612113 Real Estate Agency Principal (Aus)
612114 Real Estate Agent
612115 Real Estate Representative
612115 Real Estate Salesperson (A)
612115 Real Estate Subagent (A)
224512 Real Estate Valuer (S)
532112 Realtime Reporter (S)
111212 Rear Admiral (Navy) (S)
141999 Reception Centre Manager (N)
542111 Receptionist (General)
561311 Records Clerk (A)
224214 Records Manager
272612 Recreation Adviser (A)
272612 Recreation Coordinator (NZ)
272612 Recreation Officer (Aus)
411311 Recreational Therapist (A)
223112 Recruitment Consultant
899611 Recycling or Rubbish Collector
451899 Red Cross Aide (N)
392311 Reel Fed Printer (S)
452322 Referee (A)
224611 Reference Librarian (S)
399212 Refinery Pipeline Operator (S)
451511 Reflexologist (S)
331111 Refractory Bricklayer (S)
712911 Refrigeration Plant Operator (S)
423413 Refuge Worker
134412 Regional Education Manager
451412 Regional Guide (S)
254111 Registered Midwife (A)
254415 Registered Nurse (Acute Care) (S)
254412 Registered Nurse (Aged Care)
254423 Registered Nurse (Anaesthetic) (S)
254422 Registered Nurse (Child and Adolescence Mental Health) (S)
254413 Registered Nurse (Child and Family Health)
254414 Registered Nurse (Community Health)
254415 Registered Nurse (Critical Care and Emergency)
254416 Registered Nurse (Developmental Disability)
254417 Registered Nurse (Disability and Rehabilitation)
254422 Registered Nurse (Drug and Alcohol) (S)
254415 Registered Nurse (Emergency/Trauma) (S)
254412 Registered Nurse (Gerontology) (S)
254414 Registered Nurse (Health Education and Promotion) (S)
254415 Registered Nurse (High Dependency) (S)
254499 Registered Nurse (Infection Control) (N)
254421 Registered Nurse (Medical Practice)
254418 Registered Nurse (Medical)
254422 Registered Nurse (Mental Health)
254415 Registered Nurse (Neonatal Intensive Care) (S)
254423 Registered Nurse (Operating Room) (S)
254415 Registered Nurse (Paediatric Intensive Care) (S)
254499 Registered Nurse (Paediatrics) (N)
254423 Registered Nurse (Perioperative)
254422 Registered Nurse (Psychiatric Rehabilitation) (S)
254422 Registered Nurse (Psychogeriatric Care) (S)
254414 Registered Nurse (Public Health) (S)
254423 Registered Nurse (Recovery) (S)
254417 Registered Nurse (Rehabilitation) (S)
254499 Registered Nurse (Remote or Rural Area) (N)
254414 Registered Nurse (School Nurse) (S)
254424 Registered Nurse (Surgical)
254499 Registered Nurses nec
272114 Rehabilitation Counsellor
253399 Rehabilitation Medicine Physician (Aus) (N)
711515 Reinforced Plastic and Composite Production Worker
131111 Relationship Manager (S)
312311 Relays Draftsperson (S)
411611 Remedial Masseur (S)
241511 Remedial Teacher (S)
711999 Remotely Operated Vehicle (ROV) Pilot (N)
253322 Renal Medicine Physician (A)
253322 Renal Medicine Specialist
311299 Renal Technician (N)
731199 Rental Car Ferry Driver (N)
621912 Rental Clerk (A)
621912 Rental Salesperson
599311 Repossession Agent (S)
253913 Reproductive Endocrinologist (S)
399516 Re-recording Mixer (S)
711312 Resawyer (S)
132511 Research and Development Manager
224412 Research and Evaluation Analyst (NZ) (S)
639411 Reservations Clerk (S)
253112 Resident Medical Officer
411715 Residential Care Officer
423411 Residential Care Worker (A)
232511 Residential Interior Designer (S)
711515 Resin Transfer Moulding Machine Operator (S)
141311 Resort Manager (S)

232611 Resource Management Planner (NZ) (S)
253324 Respiratory Medicine Physician (A)
253324 Respiratory Physician (S)
141111 Restaurateur (A)
639211 Retail Buyer
232511 Retail Interior Designer (S)
442215 Retail Loss Prevention Officer
142111 Retail Manager (General)
251513 Retail Pharmacist
621111 Retail Sales Assistant (A)
142111 Retail Store Manager (A)
621511 Retail Supervisor
141999 Retirement Village Manager (N)
331111 Retort Setter (Bricklaying) (S)
253323 Rheumatologist
253515 Rhinologist (S)
452312 Rhythmic Gymnastics Coach (S)
441111 Rifleman (Army) (S)
712211 Rig Manager (S)
399111 Rigger (Boat) (S)
399411 Ring Maker (S)
711312 Ripsaw Operator (S)
312211 Road Design Draftsperson (S)
899999 Road Maintenance Traffic Controller (N)
721912 Road Marker (S)
721915 Road Roller Operator
733111 Road Train Driver (S)
233215 Roading Engineer (NZ) (S)
452215 Rock Climbing Instructor (S)
712211 Rock Drill Operator (S)
211214 Rock Singer (S)
333311 Roof Fixer (S)
334115 Roof Plumber
333311 Roof Shingler (S)
333311 Roof Slater (S)
333311 Roof Tiler
711799 Rope Making Machine Operator (N)
599411 Roster Clerk (S)
721111 Rotary Hoe Operator (S)
711599 Rotational Moulding Operator (Plastics) (N)
821912 Roughneck (S)
821912 Roustabout (Oil and Gas) (A)
711516 Rubber Belt Splicer (S)
711516 Rubber Compounder (S)
711516 Rubber Extrusion Machine Operator (S)
839212 Rubber Factory Worker
711516 Rubber Knitting and Reinforcing Machine Operator (S)
711516 Rubber Moulding Machine Operator (S)
839212 Rubber Process Hand (A)
711516 Rubber Production Machine Operator
711516 Rubber Roller Grinder Operator (S)
452411 Rugby League Footballer (S)
452411 Rugby Union Footballer (S)
561211 Rural Mail Contractor (S)
711211 Rust Proofer (S)

S

393112 Saddler (S)
323313 Safemaker (S)
312611 Safety Inspector
393113 Sail Maker
131112 Sales and Marketing Manager
621111 Sales Assistant (General)
621999 Sales Assistants and Salespersons nec
591114 Sales Clerk
639112 Sales Demonstrator
621511 Sales Department Supervisor (A)
225113 Sales Promotion Officer (S)
611312 Sales Representative (Advertising) (S)
611311 Sales Representative (Building and Plumbing Supplies)
611312 Sales Representative (Business Services)
225499 Sales Representative (Educational Products and Services) (N)
225411 Sales Representative (Industrial Products)
611399 Sales Representative (Jewellery and Watches) (N)
225412 Sales Representative (Medical and Pharmaceutical Products)
611313 Sales Representative (Motor Vehicle Parts and Accessories)
611399 Sales Representative (Musical Goods) (N)
611314 Sales Representative (Personal and Household Goods)
611399 Sales Representative (Photographic Equipment and Supplies) (N)
611312 Sales Representative (Printing) (S)
611399 Sales Representatives nec
272211 Salvation Army Officer (S)
711712 Sammying Machine Operator (S)
711913 Sand Blaster
851311 Sandwich Hand (S)
334111 Sanitary Plumber (S)
312115 Sanitary Plumbing and Water Supply Inspector (S)
399911 Saturation Diver (S)
323315 Saw Maker and Repairer
323315 Saw Sharpener (S)
711312 Sawmill Moulder Operator (S)
839412 Sawmill or Timber Yard Worker
821712 Scaffolder
323314 Scalemaker (S)
591112 Schedule Clerk (A)
731211 School Bus Driver (S)
451899 School Bus Warden (N)
811211 School Cleaner (S)
272115 School Counsellor (A)
249112 School Inspector (S)
311414 School Laboratory Technician
134311 School Principal
422116 School Services Officer (S)
134412 Schools Director (S)
311499 Science Technicians nec
399911 Scientific Diver (S)
323314 Scientific Instrument Maker and Repairer (S)
621911 Scrap Materials Buyer (A)
721213 Scraper Operator (Earthmoving) (S)
392112 Screen Printer
392112 Screen Printing Stencil Preparer (S)

212211	Screenwriter (S)
552313	Scrip Clerk (Stockbroking) (A)
212212	Script Coordinator (S)
212211	Script Writer (S)
452311	Scuba Instructor (S)
211413	Sculptor
452299	Sea Kayaking Guide (N)
899211	Seafarer (A)
121111	Seafood Farmer (A)
832115	Seafood Packer
831313	Seafood Process Worker
231214	Seaman Officer (Navy) (S)
441312	Search and Rescue Officer (S)
351311	Second Chef (S)
134311	Secondary School Principal (S)
241411	Secondary School Teacher
241411	Secondary School Teacher-Librarian (S)
521211	Secretary (General)
111111	Secretary (Government Department) (Aus) (S)
552313	Securities Clerk (A)
222211	Securities Dealer (S)
262112	Security Administrator (A)
442216	Security Consultant
821412	Security Door Installer (S)
442217	Security Guard (A)
442217	Security Officer
442299	Security Officers and Guards nec
441111	Security Police (Air Force) (S)
139111	Security Police Officer (Air Force) (Aus) (S)
841999	Seed Collector (N)
821915	Seismic Survey Assistant (S)
712213	Seismograph Shooter (S)
234412	Seismologist (S)
311412	Seismology Technical Officer (S)
111312	Senator (Aus) (S)
271111	Senior Counsel (Aus) (S)
139211	Senior Non-commissioned Defence Force Member
134212	Senior Nurse Manager (A)
334113	Septic Tank Installer (S)
149212	Service Manager (A)
621611	Service Station Attendant
631111	Service Station Console Operator (S)
599111	Settlement Agent (A)
312211	Sewage Reticulation Drafting Officer (S)
323215	Sewing Machine Mechanic (S)
711611	Sewing Machinist
451813	Sex Worker or Escort
253399	Sexual Health Physician (N)
121313	Share Dairy Farmer (Aus) (S)
222213	Sharebroker (A)
121313	Sharemilker (NZ) (S)
361211	Shearer
841517	Shearing Shed Hand (A)
841517	Shearing Shed Worker (A)
121411	Sheep and Wheat Farmer (S)
121322	Sheep Farm Manager (A)
841515	Sheep Farm Worker
121322	Sheep Farmer
392311	Sheet Fed Printer (S)
322211	Sheetmetal Patternmaker (S)
322211	Sheetmetal Trades Worker
712311	Sheetmetal Worker (Second Class) (S)
891211	Shelf Filler
839999	Sheltered Workshop Worker (N)
599212	Sheriff's Officer (Aus) (S)
411611	Shiatsu Therapist (S)
312999	Shipbuilding Draftsperson (N)
591211	Shipping and Receiving Clerk (S)
231213	Ship's Captain (A)
399112	Ship's Carpenter (S)
231212	Ship's Engineer
231213	Ship's Master
231214	Ship's Officer
231213	Ship's Pilot (S)
231299	Ship's Purser (N)
231215	Ship's Surveyor
399112	Shipwright
393114	Shoe Repairer (S)
393114	Shoemaker
711711	Shoemaking Cutter (S)
711711	Shoemaking Finisher (S)
841911	Shooter (A)
142111	Shop Manager (A)
331211	Shopfitter (S)
149913	Shopping Centre Manager (S)
851111	Short Order Cook (S)
712213	Shot Firer
452313	Show Jumping Instructor (S)
821412	Shower Screen Installer (S)
711512	Shredder/Granulator Operator (S)
312911	Shutdown Coordinator (A)
312911	Shutdown Planner (A)
711999	Sideshow Ride Operator (N)
899918	Sign Erector
399611	Sign Manufacturer (S)
441111	Signal Operator Linguist (Air Force) (S)
441111	Signal Operator Technical (Air Force) (S)
233311	Signalling and Communications Engineer (S)
263311	Signals Corps Officer (Army) (Aus) (S)
263311	Signals Officer (Army) (NZ) (S)
399611	Signwriter
711411	Silver Recovery Operator (Aus) (S)
431511	Silver Service Waiter (S)
399411	Silversmith (S)
234113	Silviculturist (S)
211214	Singer
249214	Singing Teacher (Private Tuition) (S)
452214	Ski Guide (S)
721112	Skidder Operator (S)
452314	Skiing Instructor (S)
224712	Skills Auditor (S)
831212	Slaughterer
253399	Sleep Medicine Specialist (N)
311299	Sleep Technician (N)

841312 Sleeper Cutter (S)
711411 Slide Developer (S)
821911 Slinger (S)
711111 Slip Caster (S)
321214 Small Engine Mechanic
392312 Small Offset Printer
142111 Snack Bar Manager (S)
452311 Snorkelling Instructor (S)
721999 Snow Groomer (N)
712999 Snow Maker (N)
452314 Snowboarding Instructor (S)
452314 Snowsport Instructor
452411 Soccer Player (S)
272499 Social Professionals nec
599515 Social Security Assessor
272511 Social Worker
272499 Sociologist (N)
261399 Software and Applications Programmers nec
261313 Software Architect (A)
261313 Software Designer (A)
261312 Software Developer (S)
261313 Software Engineer
261312 Software Programmer (S)
261399 Software Tester (N)
223211 Software Trainer (S)
841313 Softwood Faller (S)
311412 Soil Science Technical Officer (S)
234399 Soil Scientist (N)
271311 Solicitor
333212 Solid Plasterer
431511 Sommelier (S)
211211 Songwriter (S)
251214 Sonographer
399516 Sound Editor (S)
399516 Sound Effects Person (S)
399516 Sound Mixer (S)
399516 Sound Recordist (S)
399516 Sound Technician
351311 Sous Chef (S)
121214 Soybean Grower (Aus) (S)
399111 Sparmaker (S)
322313 Special Class Welder (S)
241599 Special Education Teachers nec
399599 Special Effects Person (N)
224611 Special Librarian (S)
224611 Special Needs Librarian (S)
241511 Special Needs Teacher
139111 Special Service Officer (Army) (Aus) (S)
139999 Specialist Managers nec
253311 Specialist Physician (General Medicine)
252712 Speech Language Therapist (NZ)
252712 Speech Pathologist (Aus)
252712 Speech Therapist (A)
272399 Sport Psychologist (N)
139915 Sports Administrator
149113 Sports Centre Manager
452321 Sports Development Officer
411611 Sports Medicine Masseur (S)
211311 Sports Photographer (S)
253999 Sports Physician (N)
252511 Sports Physiotherapist (S)
234999 Sports Scientist (N)
452317 Sports Trainer (S)
452322 Sports Umpire
452499 Sportspersons nec
322111 Spring Maker (S)
149113 Squash Centre Manager (S)
841516 Stablehand
149113 Stadium Manager (S)
254211 Staff Development Nurse (A)
212316 Stage Manager
212112 Stage Producer (S)
899999 Stagehand (N)
712999 Stationary Plant Operators nec
552314 Statistical Clerk
224113 Statistician
731311 Steam Train Driver (S)
821713 Steel Fixer
452413 Steeplechase Jockey (S)
532112 Stenocaptioner (S)
711914 Sterilisation Technician
891113 Stevedore (A)
141111 Steward (Navy) (S)
611112 Stock and Station Agent
591115 Stock Clerk
591115 Stock Control Clerk (A)
222213 Stockbroker (A)
222213 Stockbroking Dealer
831116 Stockfeed Miller (S)
621999 Stockyard Salesperson (N)
711114 Stone Polisher (S)
711114 Stone Processing Machine Operator
711114 Stone Sawyer (S)
331112 Stonemason
712211 Stope Miner (S)
741111 Storeperson
741111 Stores Assistant (A)
591115 Stores Clerk (A)
741111 Stores Despatch Hand (S)
741111 Stores Naval (Navy) (S)
721999 Straddle Carrier Operator (N)
132411 Strategic Planning Manager (S)
599521 Stream Control Officer (S)
621713 Street Vendor
721916 Streetsweeper Operator
233214 Structural Engineer
312211 Structural Engineering Drafting Officer (S)
821714 Structural Steel Erector
322311 Structural Steel Trades Worker (S)
121312 Stud Beef Cattle Farmer (S)
121313 Stud Dairy Cattle Farmer (S)
121316 Stud Master/Mistress (S)
121322 Stud Sheep Farmer (S)
272115 Student Counsellor

422116 Student Liaison Officer (S)
899999 Studio Hand (N)
831212 Stunner and Shackler (Abattoir) (S)
211199 Stunt Performer (N)
212412 Subeditor (S)
312311 Substation Design Draftsperson (S)
121217 Sugar Cane Farm Manager (A)
121217 Sugar Cane Grower
841299 Sugar Cane Planter (Aus) (N)
311411 Sugar Laboratory Assistant (S)
831117 Sugar Mill Worker
552312 Superannuation Clerk (S)
222312 Superannuation Funds Manager (S)
712916 Supercalender Operator (Papermaking) (S)
133611 Supply and Distribution Manager
133611 Supply Chain Manager (S)
591115 Supply Clerk (S)
133611 Supply Officer (Navy) (S)
263212 Support Analyst (A)
263212 Support Architect (A)
271211 Supreme Court Judge (S)
452311 Surface Supply Breathing Apparatus (SSBA) Instructor (S)
399999 Surfboard Maker (N)
452499 Surfer (N)
253511 Surgeon (General)
561511 Survey Interviewer
312116 Surveying or Cartographic Technician
312116 Surveying Technologist (A)
232212 Surveyor
821915 Surveyor's Assistant
452315 Swimming Coach or Instructor
811699 Swimming Pool Cleaner (N)
621999 Swimming Pool Salesperson (N)
899999 Swimming Pool Serviceperson (N)
561611 Switchboard Operator
262113 Systems Administrator
261112 Systems Analyst
261313 Systems Architect (S)
263211 Systems Auditor (ICT) (S)
262113 Systems Manager (A)
313112 Systems Support Officer (A)
263213 Systems Tester (A)

T

711911 Tablet Making Machine Operator (S)
441312 Tactical Response Group Officer (S)
242211 TAFE Lecturer (S)
134499 TAFE Registrar (Aus) (N)
242211 TAFE Teacher (S)
839412 Tailer-out (S)
212113 Talkback Host (S)
712912 Tank Farm Operator (Petroleum) (S)
733114 Tanker Driver
711712 Tanner (S)
839917 Tannery Worker (S)
451899 Tattoo Artist (N)
221113 Taxation Accountant
221113 Taxation Agent (A)
221113 Taxation Consultant (A)
224311 Taxation Economist (S)
599516 Taxation Inspector
731112 Taxi Driver
149999 Taxi Proprietor (N)
732111 Taxi Truck Driver (S)
399311 Taxidermist (S)
841299 Tea Plantation Worker (Aus) (N)
841299 Tea Tree Farm Worker (N)
121299 Tea Tree Farmer (N)
249311 Teacher of English to Speakers of Other Languages
241511 Teacher of Gifted Students (S)
241512 Teacher of the Hearing Impaired
241513 Teacher of the Sight Impaired
422116 Teachers' Aide
422116 Teachers' Assistant (S)
342212 Technical Cable Jointer
212317 Technical Director
232412 Technical Illustrator (S)
211311 Technical Photographer (S)
212317 Technical Producer (A)
225499 Technical Sales Representatives nec
212415 Technical Writer
599599 Technician Preventative Medicine (Army) (N)
342414 Technician Telecommunication Systems (Army) (S)
399999 Technicians and Trades Workers nec
342412 Telecommunications Cable Jointer
313199 Telecommunications Computer Systems Technician (N)
263312 Telecommunications Consultant (A)
263311 Telecommunications Engineer
313212 Telecommunications Field Engineer
342413 Telecommunications Line Mechanic (NZ)
342413 Telecommunications Linesworker (Aus)
263312 Telecommunications Network Engineer
313213 Telecommunications Network Planner
263312 Telecommunications Specialist (A)
313214 Telecommunications Technical Officer or Technologist
342414 Telecommunications Technician
639311 Telemarketer
312412 Telemetry Technician (S)
561113 Telephone Betting Clerk
561611 Telephone Operator (A)
451813 Telephone Sex Worker (S)
399517 Television Equipment Operator
212416 Television Journalist
212114 Television Presenter
212112 Television Producer (S)
212416 Television Reporter (A)
149113 Ten Pin Bowling Centre Manager (S)
149113 Tennis Centre Manager (S)
452316 Tennis Coach
452499 Tennis Player (N)
263213 Test Analyst (ICT) (A)
711799 Textile and Footwear Production Machine Operators nec
211499 Textile Artist (N)
232312 Textile Designer (S)

711714 Textile Dyeing and Finishing Machine Operator
711714 Textile Dyer (S)
839311 Textile Examiner (S)
711714 Textile Finisher (S)
711713 Textile Knitter (A)
323215 Textile Machine Mechanic (S)
311499 Textile Technical Officer (N)
323215 Textile, Clothing and Footwear Mechanic
392112 Textiles Printer (S)
399599 Theatrical Dresser (N)
423314 Therapist's Assistant (A)
423314 Therapy Aide
711599 Thermoforming Machine Operator (N)
253324 Thoracic Medicine Physician (A)
253324 Thoracic Medicine Specialist
899921 Ticket Collector or Usher
639411 Ticket Seller
821111 Tiler's Assistant (S)
733111 Tilt Tray Driver (S)
839312 Timber Grader (S)
839412 Timber Mill Worker (A)
712999 Timber Treatment Plant Operator (N)
452323 Timekeeper (Sports) (S)
841299 Tobacco Farm Worker (N)
121299 Tobacco Grower (N)
312511 Tool Design Draftsperson (S)
312511 Tool Designer (S)
712211 Tool Pusher (S)
712311 Tool Setter (S)
322111 Tool Smith (S)
323412 Toolmaker
312116 Topographic Drafting Officer (S)
451412 Tour Escort (A)
451412 Tour Guide
451412 Tour Leader (A)
212113 Tourism Radio Presenter (S)
451611 Tourist Adviser (A)
451611 Tourist Information Officer
733115 Tow Truck Driver
712111 Tower Crane Operator (S)
232611 Town Planner (S)
234599 Toxicologist (N)
821611 Track Inspector (S)
841516 Track Rider (S)
271299 Trade Mark Attorney (N)
599599 Trade Mark Examiner (Aus) (N)
223113 Trade Union Official (S)
111211 Trade Union Secretary (S)
222213 Trading Floor Operator (Stock Exchange) (S)
252214 Traditional Chinese Medicine Practitioner
252215 Traditional Māori Health Practitioner
232611 Traffic and Transport Planner (S)
712918 Train Controller
731311 Train Driver
599517 Train Examiner
599999 Train Planner (N)
132311 Training and Development Manager (S)
223311 Training and Development Professional
223311 Training Officer (A)
223311 Training Systems Officer (Navy) (S)
731312 Tram Driver
599518 Tram Inspector (S)
272413 Translator
272499 Transport Analyst (N)
149413 Transport Company Manager
639412 Transport Conductor
133611 Transport Corps Officer (Army) (S)
233215 Transport Engineer
599518 Transport Operations Inspector
272199 Trauma Counsellor (N)
599599 Travel Accommodation Inspector (N)
142116 Travel Agency Manager
451612 Travel Agent (A)
451799 Travel Attendants nec
599999 Travel Clerk (N)
451612 Travel Consultant
841313 Tree Faller
121299 Tree Farmer (N)
841313 Tree Feller (A)
721112 Tree Feller Operator (S)
841311 Tree Planter (S)
362212 Tree Surgeon (A)
452216 Trekking Guide
721214 Trench Digging Machine Operator (S)
271213 Tribunal Member
899922 Trolley Collector
591211 Truck Despatcher (S)
733111 Truck Driver (General)
891112 Truck Driver's Offsider
599215 Trust Clerk (A)
599215 Trust Officer
331111 Tuckpointer (S)
711799 Tufting Machine Operator (N)
899211 Tug Hand (S)
231213 Tug Master (S)
721999 Tunnelling Plant Operator (N)
841299 Turf Farm Worker (N)
121218 Turf Farmer (A)
121218 Turf Grower
362311 Turf Keeper (A)
899921 Turnstile Attendant (S)
712311 Turret Punch Operator (S)
532113 Typist (A)
711516 Tyre Builder (S)
839311 Tyre Finisher and Examiner (S)
899415 Tyre Fitter
711516 Tyre Retreader (S)

U

251214 Ultrasonographer (A)
312912 Ultrasound Technician (S)
712212 Underground Truck Operator (S)
451311 Undertaker (A)
431999 Uniform Room Attendant (N)

223113 Union Organiser (S)
222312 Unit Trust Manager (S)
242112 University Demonstrator (S)
242111 University Lecturer
134499 University Registrar (N)
242112 University Tutor
393311 Upholsterer
811611 Upholstery Cleaner (S)
232611 Urban and Regional Planner
253913 Urogynaecologist (S)
253518 Urologist
263299 Usability Specialist (N)

V

224512 Valuer
732111 Van Driver (A)
253521 Vascular Surgeon
121221 Vegetable Farm Manager (A)
841214 Vegetable Farm Worker (Aus)
121221 Vegetable Grower (Aus)
841215 Vegetable Picker
832211 Vehicle Assembler (S)
839311 Vehicle Assembly Inspector (S)
324211 Vehicle Body Builder
324211 Vehicle Body Maker (A)
811111 Vehicle Detailer (A)
321211 Vehicle Mechanic (Army) (S)
324311 Vehicle Painter
324111 Vehicle Refinisher (A)
324212 Vehicle Trimmer
324212 Vehicle Upholsterer (S)
899412 Vehicle Window Tinter (S)
899711 Vending Machine Attendant
899711 Vending Machine Refiller (A)
711312 Veneer Production Machine Operator (S)
711999 Venetian Blind Machine Operator (N)
211113 Ventriloquist (S)
899921 Venue Attendant (A)
323214 Vertical Borer (S)
234711 Veterinarian
361311 Veterinary Assistant (A)
361311 Veterinary Nurse
234711 Veterinary Parasitologist (S)
234711 Veterinary Pathologist (S)
512299 Veterinary Practice Manager (N)
234711 Veterinary Surgeon (A)
111212 Vice Admiral (Navy) (S)
399516 Video and Sound Recorder (S)
149111 Video Arcade Manager (S)
621912 Video Library Assistant (S)
212318 Video Producer
841216 Vineyard Worker
711513 Vinyl Welder and Fabricator (S)
211213 Violinist (S)
399517 Vision Mixer (S)
211499 Visual Arts and Crafts Professionals nec
639511 Visual Merchandiser
121215 Viticulturist (S)
211214 Vocalist (A)
242211 Vocational Education Teacher (Aus)
211111 Voice-over Artist (S)

W

271213 Waitangi Tribunal Member (NZ) (S)
431511 Waiter
333411 Wall and Floor Tiler
393213 Wardrobe Assistant (S)
393213 Wardrobe Coordinator (S)
423311 Wardsperson (A)
591116 Warehouse Administrator
741111 Warehouse Assistant (A)
741111 Warehouse Storeperson (S)
711713 Warp Knitter (S)
711715 Warper (S)
821914 Wash Plant Attendant (S)
712999 Wash Plant Operator (N)
621911 Waste Recycler (S)
899611 Waste Removalist (A)
712921 Waste Water or Water Plant Operator
323316 Watch and Clock Maker and Repairer
599521 Water Inspector
334111 Water Plumber (S)
234313 Water Quality Analyst (S)
311412 Water Resources Technical Officer (S)
733114 Water Tanker Driver (S)
891113 Waterside Worker
233911 Weapons Aeronautical Engineering Officer (Navy) (S)
231212 Weapons Electrical Engineering Officer (Navy) (S)
234913 Weather Forecaster (S)
711715 Weaving Machine Operator
313113 Web Administrator
232414 Web Designer
261212 Web Developer
313113 Web Master (A)
261212 Web Programmer (A)
212412 Web Site/Blog Editor (S)
149311 Wedding Coordinator (S)
712922 Weighbridge Operator
149999 Weight Loss Centre Manager (N)
451512 Weight Loss Consultant
599599 Weights and Measures Inspector (N)
322313 Welder (First Class) (Aus)
322313 Welder (NZ)
272613 Welfare Case Worker (A)
134214 Welfare Centre Manager
134214 Welfare Project Manager (A)
272613 Welfare Worker
821912 Well Treatment Offsider (S)
891113 Wharf Labourer (A)
591212 Wharf Tally Clerk (S)
121214 Wheat Farmer (S)
452217 Whitewater Rafting Guide
133312 Wholesaler
712111 Winch Operator (S)

712111 Winding Engine Driver (S)
811612 Window Cleaner
639511 Window Dresser (A)
821411 Window Tinter (Building) (S)
899412 Windscreen Fitter (A)
452317 Windsurfing Instructor (S)
234213 Wine Maker
431511 Wine Steward (S)
831118 Winery Cellar Hand
111212 Wing Commander (Air Force) (S)
711511 Wire Drawer (Plastics) (S)
839413 Wood and Wood Products Factory Worker
839413 Wood and Wood Products Labourer (A)
394213 Wood Machinist
394299 Wood Machinists and Other Wood Trades Workers nec
394299 Wood Model Maker (N)
711312 Wood Processing Machine Operator
839412 Wood Processing Worker (A)
311413 Wood Technologist (S)
394214 Wood Turner
399111 Wooden Boat Builder (S)
394213 Woodworking Machine Setter (S)
222111 Wool Broker (S)
639212 Wool Buyer
399917 Wool Classer
121322 Wool Grower (S)
841517 Wool Handler
532113 Word Processing Operator
223111 Workforce Planning Analyst (S)
251312 Workplace Rehabilitation Officer (S)
223113 Workplace Relations Adviser
132311 Workplace Relations Manager (S)
242211 Workplace Trainer and Assessor (S)
133512 Works Manager (Manufacturing) (Aus) (S)

Y

399111 Yacht Builder (S)
711716 Yarn Carding and Spinning Machine Operator
711716 Yarn Comber (S)
711716 Yarn Texture Machine Operator (S)
831114 Yoghurt Maker (S)
411716 Youth Accommodation Support Worker (S)
271211 Youth Court Judge (NZ) (S)
411716 Youth Liaison Officer (S)
411716 Youth Officer (A)
411716 Youth Support Worker (A)
411716 Youth Worker

Z

361114 Zookeeper
234518 Zoologist
311413 Zoology Technical Officer (S)

FOR MORE INFORMATION . . .

INTERNET **www.abs.gov.au** the ABS web site is the best place for data from our publications and information about the ABS.

LIBRARY A range of ABS publications are available from public and tertiary libraries Australia wide. Contact your nearest library to determine whether it has the ABS statistics you require, or visit our web site for a list of libraries.

INFORMATION AND REFERRAL SERVICE

Our consultants can help you access the full range of information published by the ABS that is available free of charge from our web site, or purchase a hard copy publication. Information tailored to your needs can also be requested as a 'user pays' service. Specialists are on hand to help you with analytical or methodological advice.

PHONE 1300 135 070

EMAIL client.services@abs.gov.au

FAX 1300 135 211

POST Client Services, ABS, GPO Box 796, Sydney NSW 2001

FREE ACCESS TO STATISTICS

All ABS statistics can be downloaded free of charge from the ABS web site.

WEB ADDRESS www.abs.gov.au

FOR MORE INFORMATION . . .

INTERNET	**www.stats.govt.nz**
PHONE	0508 525 525 (toll free in New Zealand)<br>+64 4 931 4600 (outside of New Zealand)
EMAIL	info@stats.govt.nz
FAX	+64 4 931 4079
POST	Private Bag 92003<br>Auckland
	PO Box 2922<br>Wellington
	Private Bag 4741<br>Christchurch

FREE ACCESS TO PUBLICATIONS

All Statistics NZ publications can be downloaded free of charge from the Statistics New Zealand web site.

WEB ADDRESS www.stats.govt.nz

2122000001066
ISBN 0 642 23200 8

Recommended retail price AUD $125.00
© Commonwealth of Australia 2006
© Crown Copyright New Zealand 2006
Produced by the Australian Bureau of Statistics
